# Putative Concussion Biomarkers Identified in Adolescent Male Athletes Using Targeted Plasma Proteomics

**DOI:** 10.3389/fneur.2021.787480

**Published:** 2021-12-20

**Authors:** Michael R. Miller, Michael Robinson, Lisa Fischer, Alicia DiBattista, Maitray A. Patel, Mark Daley, Robert Bartha, Gregory A. Dekaban, Ravi S. Menon, J. Kevin Shoemaker, Eleftherios P. Diamandis, Ioannis Prassas, Douglas D. Fraser

**Affiliations:** ^1^Department of Pediatrics, Western University, London, ON, Canada; ^2^Children's Health Research Institute, London, ON, Canada; ^3^School of Health Studies, Western University, London, ON, Canada; ^4^School of Kinesiology, Western University, London, ON, Canada; ^5^Department of Family Medicine, Western University, London, ON, Canada; ^6^Children's Hospital of Eastern Ontario Research Institute, Ottawa, ON, Canada; ^7^Neurolytixs Inc., Toronto, ON, Canada; ^8^Department of Epidemiology, Western University, London, ON, Canada; ^9^Department of Computer Science, Western University, London, ON, Canada; ^10^Department of Medical Biophysics, Western University, London, ON, Canada; ^11^Robarts Research Institute, London, ON, Canada; ^12^Department of Microbiology and Immunology, Western University, London, ON, Canada; ^13^Department of Pathology and Laboratory Medicine, University of Toronto, Toronto, ON, Canada; ^14^Department of Physiology and Pharmacology, Western University, London, ON, Canada; ^15^Depatment of Clinical Neurological Sciences, Western University, London, ON, Canada

**Keywords:** concussion, protein, biomarker, diagnosis, athlete

## Abstract

Sport concussions can be difficult to diagnose and if missed, they can expose athletes to greater injury risk and long-lasting neurological disabilities. Discovery of objective biomarkers to aid concussion diagnosis is critical to protecting athlete brain health. To this end, we performed targeted proteomics on plasma obtained from adolescent athletes suffering a sports concussion. A total of 11 concussed male athletes were enrolled at our academic Sport Medicine Concussion Clinic, as well as 24 sex-, age- and activity-matched healthy control subjects. Clinical evaluation was performed and blood was drawn within 72 h of injury. Proximity extension assays were performed for 1,472 plasma proteins; a total of six proteins were considered significantly different between cohorts (*P* < 0.01; five proteins decreased and one protein increased). Receiver operating characteristic curves on the six individual protein biomarkers identified had areas-under-the-curves (AUCs) for concussion diagnosis ≥0.78; antioxidant 1 copper chaperone (ATOX1; AUC 0.81, *P* = 0.003), secreted protein acidic and rich in cysteine (SPARC; AUC 0.81, *P* = 0.004), cluster of differentiation 34 (CD34; AUC 0.79, *P* = 0.006), polyglutamine binding protein 1 (PQBP1; AUC 0.78, *P* = 0.008), insulin-like growth factor-binding protein-like 1 (IGFBPL1; AUC 0.78, *P* = 0.008) and cytosolic 5'-nucleotidase 3A (NT5C3A; AUC 0.78, *P* = 0.009). Combining three of the protein biomarkers (ATOX1, SPARC and NT5C3A), produced an AUC of 0.98 for concussion diagnoses (*P* < 0.001; 95% CI: 0.95, 1.00). Despite a paucity of studies on these three identified proteins, the available evidence points to their roles in modulating tissue inflammation and regulating integrity of the cerebral microvasculature. Taken together, our exploratory data suggest that three or less novel proteins, which are amenable to a point-of-care immunoassay, may be future candidate biomarkers for screening adolescent sport concussion. Validation with protein assays is required in larger cohorts.

## Introduction

Concussions are frequent in sport, resulting from either a direct blow to the head or as a consequence of transmitted forces from a blow to the body (1–3). Diagnosis of concussion relies on an injury event, followed by standardized clinical testing with concussion tools (4). Concussion diagnoses are often uncertain as self-reporting of symptoms can be inaccurate (5) and symptoms can be exacerbated by other factors such as chronic pain (6). Accurate concussion diagnosis is critical to optimize medical care, to provide timely interventions and to prevent repeat injury (i.e., second impact syndrome) (7).

Accurate concussion diagnosis is critical for adolescents, as they are particularly susceptible to concussions (3) and to their potentially long-lasting neurological effects (8, 9). While self-reported headache, head pressure, fatigue, and/or noise and light sensitivity have been identified as useful discriminators of injury, they are imperfect (10, 11). A number of blood protein and lipid biomarkers for concussion diagnoses have also been investigated (4, 12–15); however, current concussion guidelines do not recommend the use of any blood biomarkers for diagnosis in adolescents due to insufficient evidence (4, 16), illustrating the need for additional biomarker exploratory studies.

Novel protein biomarker identification using plasma is possible with antibody-, gel- and chromatography-based techniques. Proximity extension assay (PEA) is a relatively new form of targeted proteomics that combines protein-specific antibodies with unique deoxyribonucleic acid (DNA) tags, followed by amplification with either quantitative polymerase chain reaction or next generation sequencing (17, 18). The result is a targeted proteomic approach to biomarker discovery with excellent sensitivity and specificity over a broad dynamic range. Indeed, PEA offers a high level of precision, and is well-suited for large-scale studies due to minimal matrix interference and cross reactivity. Indeed, conventional immunoassays suffer from cross-reactivity due to unspecific binding of antibodies, whereas, the unique DNA oligonucleotide sequences used in PEA result in matched DNA-pairs that greatly diminish non-specific antibody binding (17, 18). To the best of our knowledge, PEA has not yet been applied to biomarker discovery for concussion.

In this exploratory study, we hypothesized that the expression of numerous plasma proteins would be altered by concussion in adolescent athletes, thereby aiding identification of objective biomarkers either alone or in combinations. We report directional changes in six plasma proteins after concussion in adolescent athletes and provide a simplified diagnostic model utilizing three protein biomarkers.

## Methods

This study was approved by Western University Human Ethics Review Board. Male adolescent ice hockey athletes (Bantam Division; aged 12–14 years) participated in this study (12, 15, 19). Athletes suspected to have suffered a concussion were clinically evaluated at our academic Sports Medicine Clinic within 72 h of the injury; a time frame used to account for weekend injuries. Injured athletes were either referred by other Health Care providers, including emergency physicians, family physicians, coaches and/or trainers, or they had booked an appointment by self-referral. Control patients were recruited via posters in hockey arenas or by coaches and/or trainers whom provided athletes and their families with a study information package. To be considered as controls, the athletes were non-injured hockey players who were age- and sex-matched, and who had not suffered a diagnosed concussion in the past 6 months. All patients with a suspected concussion, as well as non-injured control athletes, including their parents/guardians, completed a Sports Concussion Assessment Tool−3rd Edition [SCAT3 (20); 13–14 years of age]. A complete history, physical and neurologic examination were also conducted by an experienced sport medicine physician. All prescription medications were recorded. Our patient evaluation was in keeping with the Berlin Consensus guidelines (4). Any subject with a reported neurological disease was excluded. All injured athletes were provided with standardized concussion care.

All athletes on their first clinic visit had 20 ml of blood drawn into EDTA Vacutainer tubes. No restrictions were placed on the time-of-day collection by intent and design, to better represent the natural state of the athlete. The blood was centrifuged, the plasma aliquoted into cryovials at a volume of 500 μl and stored at −80^o^C. Freeze/thaw cycles were avoided. Plasma was collected by strict standard operating procedures (21, 22), with equal processing times between cohorts.

A total of 1,472 plasma proteins were measured using PEA technology (OLINK EXPLORE 1536; Olink Proteomics, Sweden) (23). The Explore 1,536 panel consists of four 384 panels, with protein biomarkers related to: inflammation, oncology, cardiometabolic and neurology (1,472 specific protein targets and 64 controls). Each of the 1,472 specific proteins were targeted with two antibodies, labeled with one oligonucleotide each, and having a region of complementarity to each other. The PEA was performed in three steps: (1) antibody pairs, labeled with unique DNA oligonucleotides, were attached to their target antigen in plasma; (2) oligonucleotides that were brought into proximity hybridized and were extended by a DNA polymerase; and (3) the newly formed DNA barcode was amplified for high-sensitivity, high-specificity readout with next generation sequencing (NovaSeq Platform; Illumina Inc., San Diego, CA). Individual samples were screened based on quality controls for immunoassay and detection, as well as degree of hemolysis. Intra-assay variability was minimized via robotic pipetting for volume accuracy, and through normalization using three specifically engineered internal controls that were added to each sample; including one control for the incubation, one for the extension and one for the amplification. External negative control and plate control samples were included in each sample plate in triplicate to improve inter-assay precision. Following proteomic quality control, plasma measurements from all 35 participants were deemed suitable for analysis. The data generated were expressed as relative quantification on the log2 scale of normalized protein expression (NPX) values. NPX values were rank-based normal transformed for further analyses (Supplementary Table 1).

Plasma glial fibrillary acidic protein (GFAP) was measured with the enzyme-linked immunosorbent assay technique (24) via a commercially available test kit, as per manufacturer's instructions (Millipore/Sigma; NS830). Standard GFAP concentration curves were completed with all sample GFAP measurements and yielded linear correlation coefficients. All samples were measured in triplicate and values were reported in ng/ml.

Continuous data were reported as median (interquartile range [IQR]) and categorical data were reported as a number (percent); continuous variables were compared using Mann-Whitney U tests and categorical variables were compared using Fisher exact chi-square tests. For subject demographics, clinical data and plasma GFAP measurements, a *P*-value <0.05 was used to determine statistical significance. For targeted proteomic data, receiver operating characteristic (ROC) curves were conducted and area-under-the-curve (AUC) was calculated to determine sensitivity and specificity of all individual proteins for predicting concussion. Typically, an AUC ≥ 0.70 with a *P* < 0.05 is considered as acceptable (25); however, given the repeated measures, an AUC cutoff value of ≥ 0.78 and a *P* < 0.01 were considered significant (26). The coordinates of the curves were then analyzed to identify protein expression cut-off values based on the highest sensitivity and specificity for predicting concussion. Logistic regression analyses were also conducted with concussion as the outcome and combinations or ratios of those proteins with AUC ≥ 0.78 (*P* < 0.01) entered as predictors; the predicted values from the logistic regression models, which were independent of directional change, were then saved for use in ROC curve analyses to determine the most parsimonious combination of proteins with the greatest combined AUC. All analyses were conducted using SPSS version 25 (IBM Corp., Armonk, NY, USA).

To allow for 2D visualization, a Principal Component Analysis (PCA) was conducted to project the high-dimensional dataset onto two independent components, to investigate if a lower-dimensional representation can preserve the original structure and relationships between features (Python version 3.9.7, Sklearn decomposition PCA version 1.0.1). In addition, Pearson pairwise correlation coefficients (product-momentum coefficients) were computed to compare protein profiles between subjects to yield a correlation matrix, visualized as a heatmap (Numpy.corrcoef = 1.21).

## Results

Subject demographic and injury data are presented in Table 1. We investigated a total of 11 concussed athletes (median years of age 13; *IQR* = 13, 14) and 24 age-, sex- and activity-matched athlete control subjects (median years of age 13, *IQR* = 12.3, 14.0; *P* = 0.406). The predominant mechanisms of injuries were a body check and tripped/fell. One concussion patient had brief loss of consciousness, whereas 4 concussion patients reported amnesia. Headache was the most prevalent self-reported symptom, occurring in 91% of concussion patients. Self-reported symptom evaluation as per SCAT3 (*n* = 11) revealed a median total symptom score and a median total symptom severity of 13 (*IQR* = 7, 16) and 25 (*IQR* = 12, 49), respectively. In contrast, the non-concussed athletes had a median total symptom score and a median total symptom severity of 0 (IQR 0, 0). All concussed adolescent athletes underwent brain multiparametric magnetic resonance imaging (19), which demonstrated diffusion abnormalities within multiple white matter tracts and functional hyper-connectivity, thereby confirming concussion status.

**Table 1:** Subject demographics and clinical data.

	**Concussion patients** **(***n*** = 11)**	**Control subjects** **(***n*** = 24)**	***P*** **-value**
Age in years	13.0 (13.0, 14.0)	13.0 (12.3, 14.0)	0.406
Sex	11 M:0 F	24 M:0 F	1.000
Medical history			
Concussion(s)	3 (27)	6 (25)	1.000
Anxiety	1 (9)	0	0.314
Depression	1 (9)	0	0.314
Mood disorder	1 (9)	0	0.314
Pre-existing condition	4 (36)	3 (13)	0.171
Medications	3 (27)	4 (17)	0.652
Mechanism of injury			
Body checked	4 (36)	-	-
Tripped/Fell	4 (36)	-	-
Head into boards	1 (9)	-	-
Elbowed	1 (9)	-	-
Unknown	1 (9)	-	-
Injury details			
Loss of consciousness	1 (9)	-	-
Amnesia	4 (36)	-	-
SCAT3			
Number of symptoms	13 (7, 16)	0 (0, 0)	**<0.001**
Symptom severity score	25 (12, 49)	0 (0, 0)	**<0.001**

*Continuous data are presented as medians (IQRs), and categorical data are presented as frequency (percent). SCAT3, Sports Concussion Assessment Tool−3rd Edition*.

*Bold for statistical significance*.

Medical and medication history are also reported in Table 1. Three concussion patients reported at least one previous concussion, while 6 control subjects had suffered previous concussions (*P* = 1.000). There were no significant differences between groups with respect to anxiety, depression, mood disorders or other pre-existing medical conditions. Three concussion patients had been prescribed routine medications prior to their injury, including salbutamol, methylphenidate and fluoxetine, while four control subjects had been prescribed routine medications, including salbutamol, methylphenidate, cetirizine and loratadine (*P* = 0.279). None of the patients had been prescribed anti-inflammatories or analgesics.

**Figure 1:**
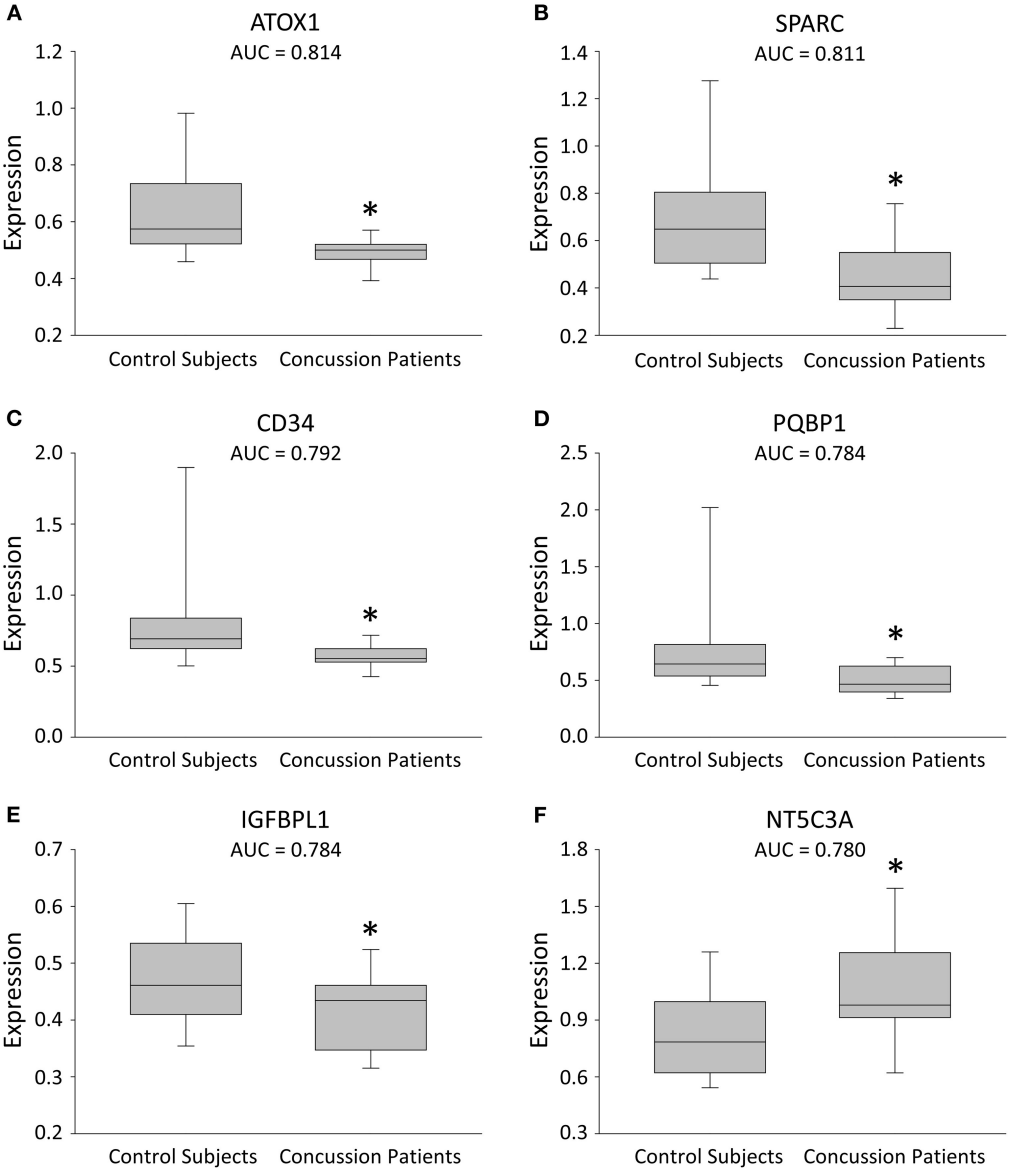
Box plots illustrating expression changes in the leading 6 proteins for concussion diagnosis. **(A–F)** Box plots comparing plasma expression in concussion patients and their matched healthy control subjects. The line within the box marks the median, the boundaries of the box indicate the 25th and 75th percentile, and the error bars indicate the 90th and 10th percentiles. All six proteins were selected based on a cut-off (AUC ≥ 0.78), with a more conservative *P* value (^*^P < 0.01).

The median time from concussion occurrence to blood draw at the first clinic visit was 2.0 days (IQR = 1, 3). A total of 1,472 plasma proteins were measured, of which six met our cut-off and statistical significance criteria with a change in expression after concussion (*P* < 0.01; Figure 1; Supplementary Table 2). Of the six proteins that changed significantly, five proteins decreased expression, whereas one protein increased expression. A ROC curve analysis was completed for each of the six identified proteins and yielded AUCs ≥ 0.78 (Figure 1; Supplementary Table 2): the AUCs [cut off values] were: antioxidant 1 copper chaperone (ATOX1; 0.81 [<0.52; *P* = 0.003]); secreted protein acidic and rich in cysteine (SPARC 0.81 [<0.56; *P* = 0.004]); cluster of differentiation 34 (CD34; 0.79 [<0.62; *P* = 0.006]); polyglutamine binding protein 1 (PQBP1; 0.78 [<0.58; *P* = 0.008]); insulin-like growth factor-binding protein-like 1 (IGFBPL1; 0.78 [<0.46; *P* = 0.008]); and cytosolic 5'-nucleotidase 3A (NT5C3A; 0.78 [>0.90; *P* = 0.009]).

**Figure 2:**
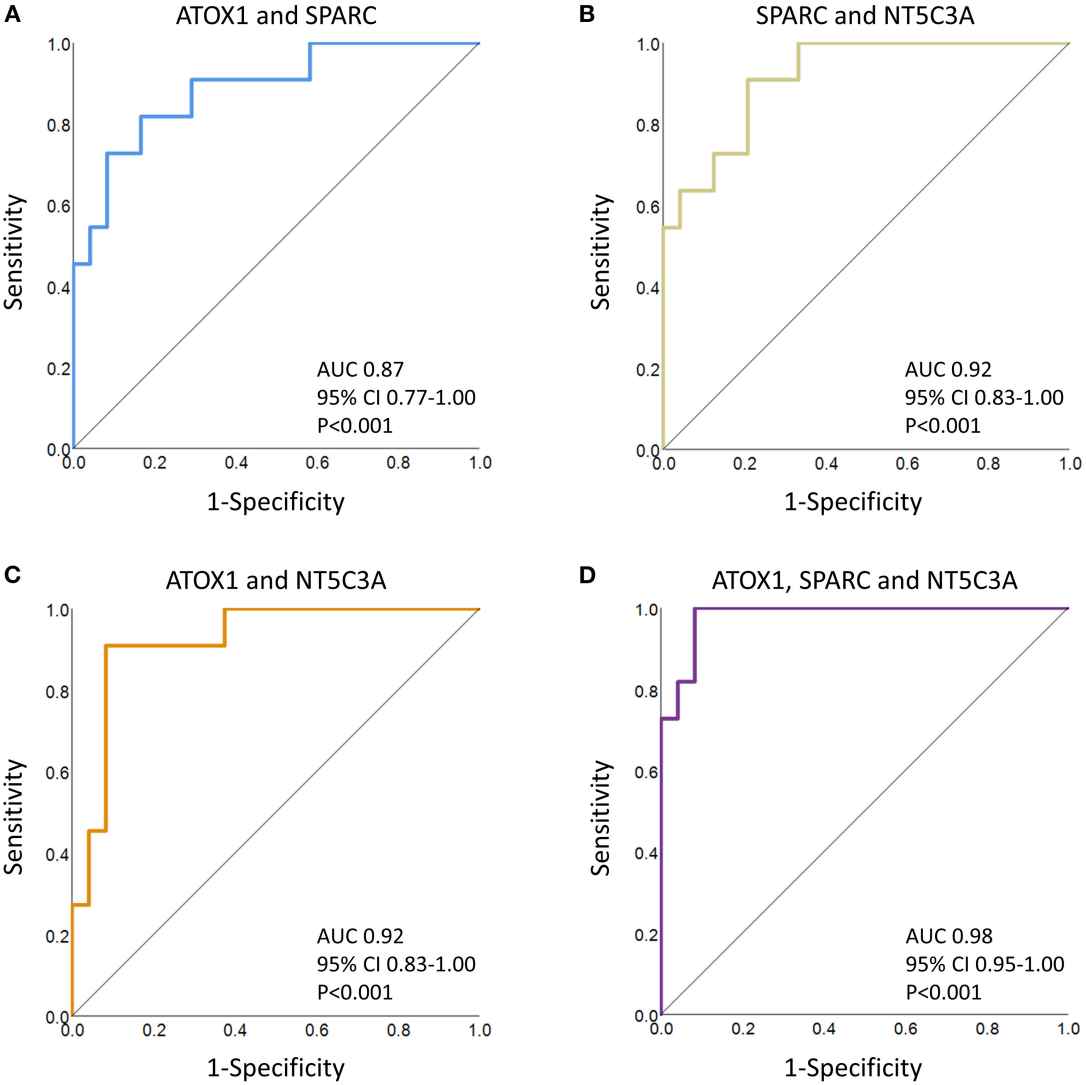
ROC curve analyses of several protein combinations for concussion diagnosis. **(A)** ATOX1 and SPARC; **(B)** SPARC and NT5C3A; **(C)** ATOX1 and NT5C3A; **(D)** ATOX1, SPARC and NT5C3A. The AUCs, confidence intervals (CI) and *P* values are indicated. When predicted values for the three leading proteins, as determined by regression analyses, were combined, the AUC increased to 0.98 (*P* < 0.001). The addition or substitution of other proteins failed to significantly improve the model.

Regression analyses identified the best protein combinations for concussion prediction (Figure 2) (15). A combination of ATOX1 and SPARC yielded an AUC of 0.87 (*P* < 0.001), while combinations of either ATOX1 and NT5C3A or SPARC and NT5C3A yielded AUCs of 0.92 (*P* < 0.001). A combination of ATOX1, SPARC and NT5C3A yielded the best AUC at 0.98 (*P* < 0.001). The addition of further proteins to the model were not helpful, and lowered the AUC slightly to 0.96 (*P* < 0.001; 95% CI 0.86-1.00; data not shown).

**Figure 3:**
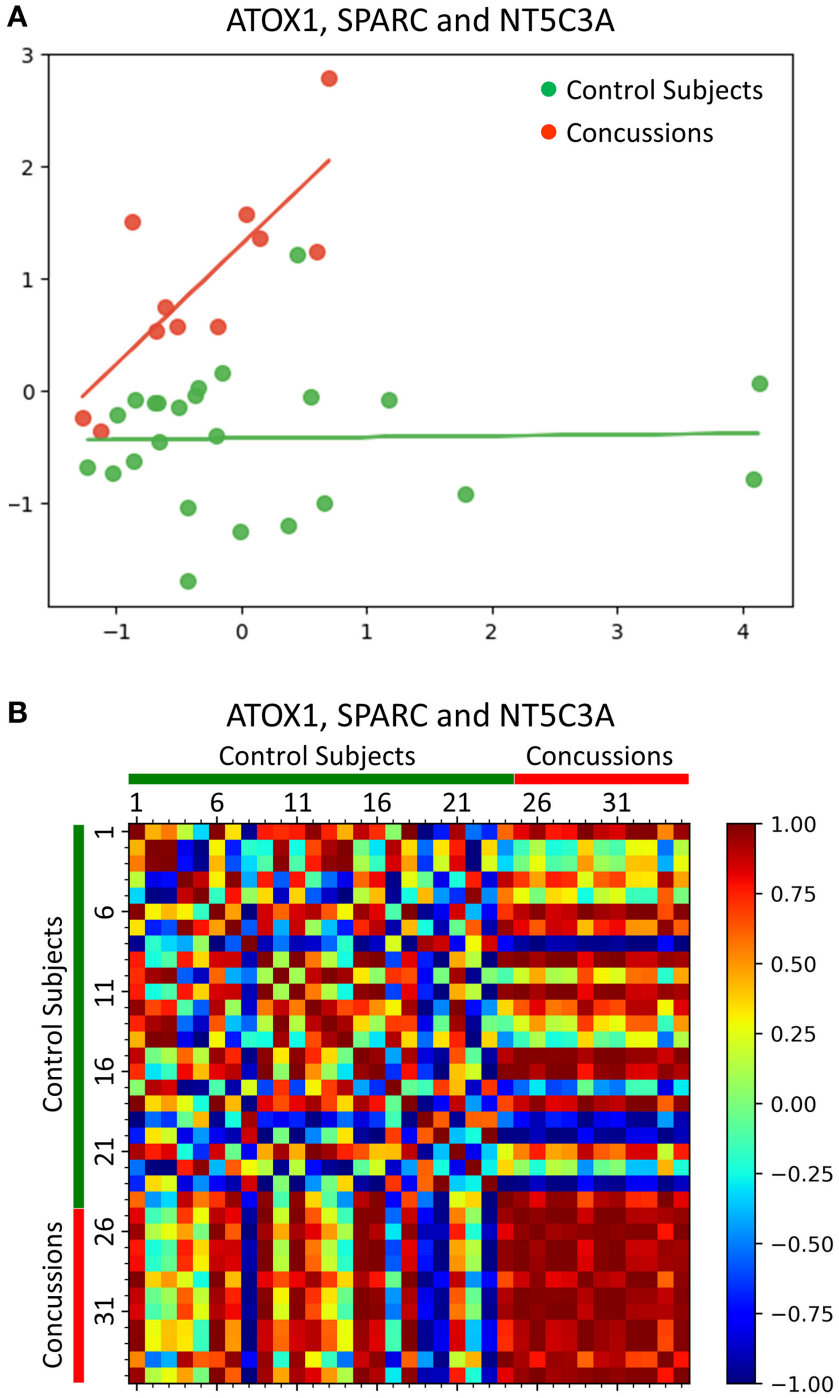
Machine learning analyses of a 3-protein combination for concussion diagnosis. **(A)** PCA showed a distinct separation of the concussed athletes when compared to their matched healthy control subjects, based on the reduction of the 3-protein feature set (ATOX1, SPARC and NT5C3A) down to two dimensions. **(B)** Pearson correlation coefficients heatmap using the 3-protein feature set (ATOX1, SPARC and NT5C3A) showed that concussed athletes were highly similar (red) when compared to their matched healthy control subjects, which were highly dissimilar.

The reduction of the 3-protein feature set (ATOX1, SPARC and NT5C3A) down to two dimensions using PCA showed a distinct separation of the concussed athletes when compared to their matched healthy control subjects (Figure 3A). In addition, using the 3-protein combination (ATOX1, SPARC and NT5C3A), the concussed athletes were shown to be highly similar as indicated by their near 1 Pearson correlation coefficients (Figure 3B). In contrast, the matched healthy control subjects were highly dissimilar.

For comparison, we measured plasma GFAP, a frequently investigated concussion biomarker, with a commercially available ELISA kit. Our measurements demonstrated a trend toward elevated plasma GFAP within the concussion cohort (median 0.6 ng/ml; IQR 23.1; *n* = 11), when compared to their matched healthy control subjects (median 0.0 ng/ml, IQR 6.2; *n* = 17; *P* = 0.072). The AUC was 0.70.

## Discussion

In this exploratory study, we identify a number of plasma proteins that change after concussion, with six proteins having good to very good diagnostic potential (AUC 0.78–0.81). Combining two plasma proteins displayed very good diagnostic potential with AUCs of 0.87–0.92, while combining three of the leading plasma proteins resulted in a potentially excellent diagnostic test with an AUC of 0.98 (ATOX1, SPARC and NT5C3A).

Accurate sport concussion diagnosis remains elusive for several reasons. First, concussion diagnostics are complicated by patient and injury heterogeneity. Second, many patients struggle with the recognition of injury-induced deficits, as well as quantification of their symptoms. Third, many athletes are influenced by secondary gain (e.g., an athlete who wants to continue play) resulting in injury symptom underreporting (27, 28). Finally, concussions are diagnosed by clinical criteria, but clinical diagnostics tools are imperfect. The SCAT is the most widely used tool, and it is reported to have good diagnostic accuracy (10), but certain self-reported symptoms are unhelpful with initial concussion diagnosis in adolescents (10, 11).

Protection of concussed athletes requires early and accurate diagnoses. The protein biomarkers discovered here appear to objectively diagnose adolescent concussion within the first 72 h of injury and would help inform and protect patients. Indeed, once concussed, a graded return to activities with symptom recovery is recommended (4). Medical clearance requires resolution in self-reported symptoms and clinical signs. If return to sport occurs prematurely for any reason, the athlete is at increased risk of injury, with potentially serious consequences, including second impact syndrome (7). Our previous studies investigating these same adolescent injured athletes utilizing both plasma metabolomics and multiparametric magnetic resonance imaging demonstrated abnormalities that persisted for up to 3 months post-injury (12, 15, 19).

ATOX1, a small cytosolic protein with an essential role in copper homeostasis (29, 30) was the top performing protein biomarker to diagnose concussion with an *AUC* = 0.81. Copper concentration is greatest in the human brain compared to other organs (30), with the highest levels found in neurons of the cerebral cortex, hippocampus and cerebellum (31). Copper homeostasis is essential for brain development, as well as normal cellular function, regulation of inflammation and maintenance of myelination (31, 32). ATOX1 mediates copper trafficking and storage, and modulates transcription and antioxidant defense (33). In neurons, ATOX1 transfection offers cellular protection from stress (34). Concussion decreased plasma ATOX1 expression, perhaps reflecting alterations in brain copper homeostasis.

SPARC, also known as osteonectin or basement-membrane protein 40 (BM-40), is an acidic extracellular matrix glycoprotein with expression in endothelium, fibroblasts, and macrophages. A top performer for diagnosing concussion, SPARC yielded an *AUC* = 0.81. SPARC is constitutively expressed, with expression up-regulated in tissue regions undergoing remodeling, repair, and proliferation (35). In brain, SPARC is reported to be highly expressed in developing blood vessels and is important for angiogenesis and blood brain barrier establishment and function (36). SPARC has been reported to be induced in mature blood vessels close to an injury site and in new blood vessels that develop following injury (37). Paradoxically, SPARC expression in plasma was acutely depressed after concussion, perhaps due to earlier sampling time frame and/or early sequestration after injury.

NT5C3A, a member of the 5′-nucleotidase family, mediates nucleotide catabolism. NT5C3A degrades RNA by dephosphorylating the pyrimidine 5′ monophosphates UMP and CMP to their corresponding nucleosides. Plasma NT5C3A expression is elevated after concussion, yielding an *AUC* = 0.78. NT5C3A functions as a negative feedback regulator of inflammatory cytokine signaling (38), but when 5-nucleotidase activity is excessive, it results in a syndrome characterized by ataxia, hyperactivity, short attention span and poor social interaction (39). This latter syndrome is improved by oral supplementation of UMP and CMP (39), which are believed to be neuroprotective. Indeed, pyrimidine supplementation is currently being tested as a therapeutic agent in pediatric patients after traumatic brain injury (40).

We demonstrated changes in plasma proteins after concussion that may be useful for injury diagnosis in adolescent athletes, but other plasma biomarkers have been proposed (13). For example, several protein biomarkers are released after injury and are relatively specific to a wide variety of brain cells, including neurons (UCH-L1, NF-L, Tau, NSE, SNTF), astrocytes (GFAP, S100β) and oligodendrocytes (MBP). To date, only a handful of brain injury protein biomarkers have shown some degree of diagnostic accuracy, such as a combination of GFAP and UCH-L1 (41). These latter two biomarkers are FDA approved to identify concussed adults in need of computerized tomography scanning, but their usefulness to diagnose concussion in adolescents is unclear (42, 43). With the exception of neurofilament light chain (NEFL, Supplementary Tables 1, 2), the brain injury biomarkers listed above were not part of our targeted panels, and therefore not measured in this study. Nonetheless, we did measure plasma GFAP levels for a comparison. Plasma GFAP was elevated after concussion, but fell short of significance, perhaps due to the post-injury blood sampling time frame (42, 43) and/or insufficient ELISA kit sensitivities. An alternative to protein measurements is mass spectrometry measurements of plasma glycerophospholipids, yielding AUCs for adolescent concussion diagnosis equal or greater to the classical protein biomarkers (12, 15).

The putative concussion biomarkers discovered here may serve well as a standalone point of care screening tool, or as part of a multimodal concussion diagnostic model. For the former, the identified proteins would be amenable to immunoassay technology, including lateral flow. For the latter, protein measurements may be combined with other approaches including electroencephalography, neurocognitive tests and standard concussion assessment tools (44), as well as multiparametric advanced imaging (19, 45).

Our study has several limitations. First, our study evaluated a limited number of adolescent athletes. Despite this caveat, a strong predictive model was identified with high statistical significance illustrating the potential of these proteins for diagnostic utility. Second, we did not have baseline measurements from each athlete and, therefore, we compared concussed athletes to a control cohort who were age-, sex- and activity-matched. Third, our matched control group was uninjured; further studies should add an additional control group consisting of matched athletes with musculoskeletal injuries. Fourth, our study population was only male. Follow-up studies should investigate protein changes in both sexes. Finally, the temporal threshold for accurately measuring changes in the identified proteins is unclear at present, but certainly falls within our 72-h post-injury time period. Despite these caveats listed above, we emphasize that these exact cohorts are extremely well-characterized having been studied with both multi-parametric MRI (19) and metabolomics (12, 15).

In summary, we identify a number of plasma proteins that change after concussion in adolescent athletes. Importantly, a combination of up to three novel plasma proteins (ATOX1, SPARC and NT5C3A), which are amenable to point of care immunoassay testing, have been identified as putative concussion biomarkers. Despite a paucity of studies on these three identified proteins, the available evidence points to their roles in modulating tissue inflammation and regulating integrity of the cerebral microvasculature. Future studies should endeavor to have a larger cohort of athletes, comprised of both sexes, with measurements at baseline, post-injury and at multiple intervals during recovery.

## Data Availability Statement

The original contributions presented in the study are included in the article/Supplementary Material further inquiries can be directed to the corresponding author.

## Ethics Statement

The studies involving human participants were reviewed and approved by Human Ethics Review Board, Western University. Written informed consent to participate in this study was provided by the participants' legal guardian/next of kin.

## Author Contributions

DF: concept, methods design, data collection, data analysis, data interpretation, manuscript writing, and submission. MM, MP, and MD: data analysis and manuscript writing. MR, LF, AD, RB, GD, RM, JS, ED, and IP: data collection, and critical review of the manuscript. All authors contributed to the article and approved the submitted version.

## Funding

DF received funding for subject recruitment and sample procurement from the Children's Health Foundation (London, Ontario, Canada; https://childhealth.ca/), and funding for targeted proteomics from Neurolytixs Inc. (Toronto, Ontario, Canada; https://www.neurolytixs.com/). The Children's Health Foundation was not involved in the study design, collection, analysis, interpretation of data, the writing of this article or the decision to submit it for publication.

## Conflict of Interest

DF discloses a provisional patent and the licensing of technology to Neurolytixs Inc. Given the commercial interests of DF, all data and analyses were independently reviewed for accuracy by an impartial Institutional Scientist prior to journal submission. AD and DF are employed by Neurolytixs Inc. The remaining authors declare that the research was conducted in the absence of any commercial or financial relationships that could be construed as a potential conflict of interest.

## Publisher's Note

All claims expressed in this article are solely those of the authors and do not necessarily represent those of their affiliated organizations, or those of the publisher, the editors and the reviewers. Any product that may be evaluated in this article, or claim that may be made by its manufacturer, is not guaranteed or endorsed by the publisher.
