# Supplementary material for: Putative Concussion Biomarkers Identified in Adolescent Male Athletes Using Targeted Plasma Proteomics
Source: Front Neurol. 2021 Dec 20;12:787480. doi: 10.3389/fneur.2021.787480 (PMC8721148; doi:10.3389/fneur.2021.787480)
Supplement: Supplementary file 1 [file Data_Sheet_1.PDF]

	P20333,TNFRSF1B,NEUROLOGY	Q6UXH9,PAMR1,NEUROLOGY	P16871,IL7R,NEUROLOGY	Q8N6Q3,CD177,NEUROLOGY	Q9NRG1,PRTFDC1,NEUROLOGY
**UniPort**	P20333	Q6UXH9	P16871	Q8N6Q3	Q9NRG1
**Assay**	TNFRSF1B	PAMR1	IL7R	CD177	PRTFDC1
**Panel**	NEUROLOGY	NEUROLOGY	NEUROLOGY	NEUROLOGY	NEUROLOGY
**Control-1**	0.295555301	0.280836041	0.694862775	0.233598028	0.477773917
**Control-10**	0.454420555	0.333001366	0.97036539	0.022189177	1.12482456
**Control-11**	0.441351498	0.367674238	0.643227057	0.180228616	0.769077025
**Control-12**	0.32666867	0.394938522	0.681223448	0.259894859	1.242632765
**Control-13**	0.358811989	0.445686139	0.828457733	0.184411432	0.774104371
**Control-14**	0.344481879	0.373220465	0.566756132	0.220859378	0.393790442
**Control-15**	0.365463695	0.336388432	1.091112606	0.616340714	0.729712463
**Control-16**	0.428658305	0.38209413	0.711975746	0.782900028	0.863399403
**Control-17**	0.248066702	0.324929807	0.536407011	0.188103683	0.891372279
**Control-18**	0.296766469	0.23379241	0.251599333	0.169152922	0.425844907
**Control-19**	0.315366211	0.303359417	0.598448913	0.07117429	0.957006083
**Control-2**	0.42445985	0.42513708	1.134376853	0.828113259	0.927102014
**Control-20**	0.25003466	0.276221974	0.554554056	0.158164749	0.758804427
**Control-21**	0.253138702	0.27289162	0.240631931	0.214775301	0.478237777
**Control-22**	0.18780404	0.202557021	0.561438538	0.174065235	0.533108097
**Control-23**	0.230893586	0.337743521	0.4557454	0.178934068	0.549808075
**Control-24**	0.332586151	0.449035061	0.874299816	0.437726005	0.88227481
**Control-3**	0.285665754	0.356902027	0.741439047	0.212981496	0.735501404
**Control-4**	0.321970407	0.443467413	0.707842357	0.051990794	1.098625741
**Control-5**	0.2946757	0.327235234	0.839847913	0.182643207	0.689776078
**Control-6**	0.317449718	0.276470988	0.292153808	0.128202829	0.363719959
**Control-7**	0.319126429	0.283083585	0.371053726	0.022127741	0.756546152
**Control-8**	0.282025985	0.252245424	0.635824274	0.069449109	0.774587433
**Control-9**	0.250954896	0.323200201	0.362260648	0.053175297	0.832891226
**Concussion-1**	0.376990481	0.296067903	0.221718337	0.228442028	0.993023662
**Concussion-10**	0.31355708	0.495961487	0.795591628	0.226958433	0.639980565
**Concussion-11**	0.396062496	0.307402329	0.775877073	0.022511406	0.607013286
**Concussion-12**	0.309111664	0.298044571	0.558604848	0.136096324	0.596006512
**Concussion-13**	0.316548837	0.430176315	0.873270191	0.605920323	0.762706553
**Concussion-2**	0.364073086	0.349775223	0.665633524	0.101587866	0.599985691
**Concussion-3**	0.308876068	0.226314355	0.57383187	0.101884042	0.421294212
**Concussion-4**	0.316790285	0.294532757	0.638783964	0.04528572	0.743291492
**Concussion-5**	0.300200857	0.288491357	0.329419988	0.135428192	0.47711204
**Concussion-7**	0.264804584	0.247517082	0.30333839	0.088603039	0.864417389
**Concussion-8**	0.322461762	0.404216467	1.087111561	0.169129474	0.694621995

**Q15043,SLC39A14,NEUROLOGY**	**Q9NQ38,SPINK5,NEUROLOGY**	**Q92765,FRZB,NEUROLOGY**	**Q6P4E1,CASC4,NEUROLOGY**	**O60242,ADGRB3,NEUROLOGY**	**Q969Z4,RELT,NEUROLOGY**
Q15043	Q9NQ38	Q92765	Q6P4E1	O60242	Q969Z4
SLC39A14	SPINK5	FRZB	CASC4	ADGRB3	RELT
NEUROLOGY	NEUROLOGY	NEUROLOGY	NEUROLOGY	NEUROLOGY	NEUROLOGY
0.947632774	0.442423523	0.479864835	0.39112455	0.425579334	0.368771738
0.778516758	0.454452054	0.570144692	0.709807636	0.594850898	0.418906449
0.898879729	0.518566171	0.511604195	0.569394317	0.600484953	0.571014783
0.935040008	0.577943353	0.311240132	0.784692872	0.658337827	0.358240413
1.085530303	0.412452532	0.467293217	0.732398124	0.481864693	0.402566772
0.712815197	0.577222723	0.506312497	0.423313968	0.56007813	0.46532158
0.646310798	0.612847489	0.60869862	0.554823192	0.590537262	0.379586345
0.561049513	0.492638061	0.521919786	0.41884838	0.507401609	0.42699764
0.908652142	0.459711289	0.474703963	0.505786347	0.264951463	0.305321269
0.50754231	0.447915967	0.421498675	0.339127575	0.279689888	0.24865201
0.753719697	0.446459124	0.440526282	0.420011286	0.351988451	0.335712928
0.844693585	0.622609718	0.43603021	0.625985003	0.482466273	0.676799309
0.625854847	0.489302989	0.220080008	0.525477173	0.439489316	0.249757516
0.524894723	0.431310876	0.383792919	0.322394715	0.311693505	0.328234775
0.453696678	0.409206226	0.336738364	0.350357576	0.328621779	0.199091012
0.516700434	0.48036402	0.825820421	0.287333876	0.248979696	0.290075429
0.79366385	0.512136397	0.522100701	0.601734925	0.444298138	0.352208102
0.859577723	0.535255641	0.458470242	0.496408595	0.428390977	0.389528286
0.731180753	0.486968379	0.530454164	0.641046093	0.361508131	0.378850354
1.067399322	0.403181123	0.477409771	0.461883207	0.506101972	0.483001642
0.514770042	0.471065637	0.33847003	0.468818043	0.384272063	0.365843872
0.832198735	0.509410287	0.393490306	0.44596426	0.257652769	0.416503349
0.470673978	0.482466273	0.428123817	0.343956971	0.256725766	0.392292049
0.733617521	0.428598884	0.293290043	0.287971911	0.298851357	0.341841628
0.886688949	0.485283596	0.425844907	0.39713461	0.402399384	0.40114619
0.787908498	0.570144692	0.568408487	0.412996082	0.314384065	0.405872922
0.69515182	0.457200853	0.506172137	0.462556018	0.47293047	0.353088074
0.518063196	0.432088878	0.375061738	0.495652187	0.388799967	0.334690614
0.695585614	0.4799979	0.374386417	0.610896551	0.433919703	0.437756347
0.699259571	0.56864493	0.49086561	0.311067591	0.327825504	0.313079294
0.558759748	0.459456442	0.432298579	0.375764323	0.435788491	0.290900968
0.589473965	0.449377563	0.521305144	0.452032999	0.499826743	0.33980995
0.579708683	0.545934585	0.315563008	0.345031504	0.239384227	0.362009635
0.790643918	0.566756132	0.328644558	0.40934807	0.324502163	0.300180049
1.446634036	0.469338267	0.476748399	0.427767861	0.635559897	0.346277367

O14625,CXCL11,NEUROLOGY	Q9NPH3,IL1RAP,NEUROLOGY	Q15814,TBCC,NEUROLOGY	Q06323,PSME1,NEUROLOGY	P20774,OGN,NEUROLOGY	P00749,PLAU,NEUROLOGY
O14625	Q9NPH3	Q15814	Q06323	P20774	P00749
CXCL11	IL1RAP	TBCC	PSME1	OGN	PLAU
NEUROLOGY	NEUROLOGY	NEUROLOGY	NEUROLOGY	NEUROLOGY	NEUROLOGY
0.13637017	0.308298548	0.637191974	1.0132569	0.419109753	0.556903784
0.225640822	0.514128182	0.616255277	0.687199075	0.539651523	0.576942721
0.326148297	0.715141187	0.668453912	1.047971876	0.577582926	0.694140687
0.279786838	0.292518546	0.466387168	0.741850302	0.439093475	0.685438912
0.30149375	0.394500764	0.894590907	1.513721713	0.446397236	0.691164009
0.436362793	0.284223944	0.827768928	1.020446113	0.527850029	0.60924736
0.211246596	0.33641175	0.647027977	0.846099947	0.344076198	0.629334963
0.156919875	0.303317365	0.39066394	0.588861395	0.523006217	0.653246774
0.292619943	0.229951259	0.483772276	0.937831082	0.330243026	0.51192345
0.301870148	0.208497212	0.4555559	0.869284304	0.237072463	0.378535366
0.118593759	0.233954519	0.562139462	0.604242682	0.322327682	0.425903946
0.539539317	0.388799967	1.14480351	1.462665627	0.840313752	0.55413139
0.14014576	0.264621099	0.47976506	0.437483345	0.439855025	0.414717261
0.125677649	0.160128277	0.578945721	0.701930452	0.405423045	0.504385952
0.121598724	0.177451909	0.466969426	0.731231437	0.240431862	0.358389432
0.21915143	0.486024181	0.615999037	1.088695123	0.338235502	0.308683441
0.418152183	0.300700673	0.952373907	1.804000827	0.344290911	0.655242118
0.156691628	0.569512732	0.843874287	0.943045968	0.408582693	0.586376829
0.202108233	0.236317766	0.529902926	0.922614582	0.372522637	0.663928598
0.253050986	0.481430684	0.550189305	0.627418511	0.487035892	0.512811315
0.243838865	0.4768806	0.501145002	0.675299783	0.454137161	0.50662845
0.266757363	0.418036263	0.724471077	1.297199165	0.605752349	0.353039129
0.160985204	0.475033117	0.449845033	0.686675312	0.538493188	0.46274843
0.174851248	0.21453724	0.492842986	0.631913941	0.366656241	0.394774306
0.212200506	0.582770599	0.505786347	0.693371287	0.400618236	0.533108097
0.23855602	0.308576478	0.604284566	0.912249272	0.401285241	0.466064005
0.175397489	0.602694999	0.675346593	1.055553718	0.364073086	0.457486159
0.270518657	0.396062496	0.765195344	1.386165964	0.434491543	0.340800652
0.280758187	0.305363599	0.594191553	1.201885949	0.629465843	0.733363312
0.118273601	0.234555299	0.656606074	0.936661713	0.306529961	0.505330793
0.20735863	0.452503231	0.57327529	0.656788149	0.253226448	0.353749497
0.179916574	0.269451963	0.633009914	0.768011596	0.586011142	0.657744872
0.394145443	0.404889462	0.554630938	0.74046323	0.344171609	0.365463695
0.231807637	0.317317722	0.607897504	0.985002984	0.382200084	0.371542718
0.184373089	0.304454808	0.820741609	1.41656814	0.360332329	0.428420672

Q9HA65,TBC1D17,NEUROLOGY	P14384,CPM,NEUROLOGY	P51452,DUSP3,NEUROLOGY	Q14112,NID2,NEUROLOGY	P21217_Q11128,FUT3_FUT5,NEUROLOGY
Q9HA65	P14384	P51452	Q14112	P21217_Q11128
TBC1D17	CPM	DUSP3	NID2	FUT3_FUT5
NEUROLOGY	NEUROLOGY	NEUROLOGY	NEUROLOGY	NEUROLOGY
0.707253835	0.273592389	0.771747067	0.531668906	0.306381268
0.758226088	0.496615088	0.610896551	0.500797755	0.20770387
0.73417709	0.249290533	1.102287073	0.613740205	0.198044975
0.983774797	0.454515059	3.253291284	0.387965427	0.488286569
1.038283197	0.290598669	0.930320679	0.455524325	0.215730191
0.861367025	0.306912647	0.888781073	0.49267221	0.224315311
0.974274357	0.383314373	2.139539034	0.483370053	0.131096402
0.853226098	0.310873598	0.927551956	0.455903377	0.260183251
0.96727633	0.297590422	0.706127202	0.449035061	0.149684838
0.961061061	0.210939327	0.820400343	0.593944487	0.285764775
0.932580391	0.2859431	0.505926601	0.41847113	0.156192816
1.374588696	0.403572563	0.99972278	0.792619299	0.362888942
0.814959347	0.234929534	0.903627449	0.488997841	0.226942702
1.107341337	0.223043962	2.52223028	0.360132573	0.153488237
0.623430222	0.215969576	0.944943516	0.382200084	0.130488991
1.005909142	0.261521235	3.164768562	0.351135557	0.346061415
1.126150778	0.256387885	1.479797739	2.576656105	0.139428752
1.170317518	0.262611136	0.705686834	0.539352359	0.195792852
0.793058942	0.544838289	1.338020296	0.473619373	0.198884121
1.033544137	0.281713382	1.465100875	0.376468223	0.242154547
0.89564567	0.216884672	0.907393355	0.434009943	0.16007279
1.008562158	0.338916082	0.765938254	0.484510555	0.274085897
0.70290421	0.219790358	0.900251498	0.338118298	0.216824547
1.063190412	0.207992008	0.984252243	0.354338467	0.133037051
0.924727373	0.197072722	0.716679503	0.350333292	0.155189193
1.464390176	0.251826149	0.506488002	0.758120982	0.341226122
0.933485812	0.319458404	0.773889773	0.485485462	0.193834866
1.225185332	0.252542832	0.78176126	0.938676535	0.174911858
1.028612792	0.351744556	0.856485068	0.608951823	0.346133384
0.934651216	0.317031918	0.972250516	0.414717261	0.172217064
0.58548333	0.270781297	0.577743088	0.471196262	0.208526118
0.907519155	0.283162083	0.920825697	0.483537606	0.356308795
0.707793295	0.227052842	0.926202785	0.374204808	0.218015105
1.056944776	0.256441205	1.014029765	0.431729626	0.294267476
1.649581434	0.263851849	1.8303252	0.422288247	0.222611496

Q9Y240,CLEC11A,NEUROLOGY	Q6UX27,VSTM1,NEUROLOGY	P06734,FCER2,NEUROLOGY	P52798,EFNA4,NEUROLOGY	P13385,TDGF1,NEUROLOGY	Q9H5V8,CDCP1,NEUROLOGY
Q9Y240	Q6UX27	P06734	P52798	P13385	Q9H5V8
CLEC11A	VSTM1	FCER2	EFNA4	TDGF1	CDCP1
NEUROLOGY	NEUROLOGY	NEUROLOGY	NEUROLOGY	NEUROLOGY	NEUROLOGY
0.648105238	0.466969426	0.554707832	0.525404332	0.147378706	0.352501182
0.790041312	0.668500248	0.908085472	0.417572903	0.78627179	0.319746395
1.347887417	0.698194066	0.797634657	0.492057903	1.362352462	0.402232065
0.786326293	0.494656863	0.552329083	0.370745221	0.825648715	0.436786448
0.904065998	0.774963356	0.5749068	0.55953489	1.99612214	0.378509129
0.564756157	0.649859615	0.939197192	0.540437616	0.166431452	0.309712174
0.679572793	0.795536484	0.892670713	0.401591322	0.81988871	0.203655121
0.767160317	0.882152509	0.718669318	0.424401012	0.913894794	0.291143033
0.869585627	0.551678629	0.452565966	0.294328673	0.381194709	0.227635899
0.636794597	0.864417389	0.632395935	0.303044171	0.578344092	0.229521308
0.473159993	0.632922166	0.430027253	0.317779948	0.160239308	0.257171023
1.550571443	0.67604913	1.135714334	0.657015814	0.147419574	0.465999399
0.737645729	0.68700857	0.601401345	0.343980813	0.140427754	0.220920622
0.532849494	0.941347957	0.467325608	0.288411381	0.149249707	0.214477766
0.485485462	0.726835102	0.291789525	0.215520947	0.684584247	0.300700673
0.464870248	0.821083017	0.435818698	0.335433806	0.535070168	0.322417063
0.754765299	0.755236293	0.553056968	0.358936365	0.416272454	0.283672853
0.540550009	0.597744146	0.665541254	0.336528361	0.599486845	0.388126811
0.729661885	0.643316234	0.508669325	0.386060806	0.65161874	0.278413293
0.628506687	1.309213152	0.700812299	0.438363627	0.162284824	0.300888318
0.934845592	0.436453542	0.546805629	0.323783192	0.170553941	0.298809931
1.172428546	0.731839911	0.699598936	0.377225733	0.16559144	0.41091161
0.949078944	0.680940194	0.682594171	0.290638957	0.191604055	0.329054852
0.559728844	0.800736791	0.624771262	0.265098424	0.15127023	0.24618248
0.636838738	1.116745158	0.71946679	0.445037867	0.110728615	0.356827819
0.757175691	0.917067607	0.710053679	0.296540282	0.962861368	0.226471278
0.534069722	0.3515983	0.733058379	0.31709785	0.643316234	0.237302631
0.602068691	0.47853621	0.499515032	0.351330322	0.646131627	0.272702532
0.910796088	0.569354851	0.803906711	0.282926654	0.184603268	0.295493848
0.537821748	0.481330584	0.598697852	0.367878177	0.153105711	0.328325794
0.309776584	0.929353908	0.66287098	0.34018702	0.149622599	0.171383485
0.630251696	0.536927797	0.79366385	0.43077308	0.675721189	0.27308084
0.470413054	0.546313129	0.301974786	0.278336111	0.146350543	0.257902918
0.472733825	0.501527252	0.351768938	0.360032737	0.203232072	0.271495464
0.575225684	1.104734738	0.625681347	0.34393313	0.639581449	0.299577255

A1E959,ODAM,NEUROLOGY	Q9NZD4,AHSP,NEUROLOGY	P06733,ENO1,NEUROLOGY	Q96RD9,FCRL5,NEUROLOGY	O43927,CXCL13,NEUROLOGY	P15336,ATF2,NEUROLOGY
A1E959	Q9NZD4	P06733	Q96RD9	O43927	P15336
ODAM	AHSP	ENO1	FCRL5	CXCL13	ATF2
NEUROLOGY	NEUROLOGY	NEUROLOGY	NEUROLOGY	NEUROLOGY	NEUROLOGY
1.32106367	1.169830898	1.244356614	0.212553806	0.249186877	0.484040611
0.824790717	1.676555218	1.180174343	0.278934831	0.482700424	0.706176149
0.690397909	2.378908859	1.697133436	0.352256931	0.673103377	0.734737085
1.035480224	1.05270412	2.021883679	0.240015586	0.331228794	0.77335354
1.575489348	1.456191406	2.002774511	0.303296341	0.504001524	0.791521257
0.758278646	1.311483812	0.739232454	0.189175872	0.265834453	0.786544339
0.999099315	1.282625505	1.01094184	0.573156093	0.351354675	1.070288698
0.92242275	1.193087622	1.194245963	0.349290667	0.268594189	0.735756353
0.690302206	1.505664148	1.497857107	0.313817998	0.333810213	0.809835056
0.900875721	1.999029829	0.99972278	0.238489888	0.372832621	0.801347555
1.150770386	1.334315654	1.359805208	0.149871711	0.22768324	0.721764454
1.003332647	3.177076877	2.692226891	0.366072169	0.353039129	0.65161874
0.740668558	0.906701766	0.793773883	0.259301058	0.170305862	0.742673497
0.882030225	2.393132041	0.929869394	0.214240034	0.332655317	0.735093668
0.926588062	1.841140851	0.826106679	0.17836445	0.199422487	0.919167696
0.875634073	2.675484183	1.136108011	0.323603698	0.293005571	0.94258851
1.819951494	4.353271502	1.445932295	0.241099406	0.222534358	0.837696761
1.10060745	1.735799123	1.174706219	0.332540048	0.39166714	0.749447851
1.338855257	1.459829621	3.102647935	0.414401175	0.42401876	1.165460412
1.573415826	0.956873423	0.858327425	0.226769733	0.367241244	1.129434026
1.214279049	0.76785191	1.528588413	0.261521235	0.271890943	1.540394631
0.957736042	1.868136664	1.793154752	0.2124507	0.226864063	0.492126121
0.798243054	1.049934989	1.606587994	0.229616783	0.358737384	0.946713631
1.007095153	0.743085437	0.669195663	0.241366942	0.246609452	2.145628046
0.880503107	1.567102993	0.910101905	0.274237924	0.284361883	0.896701676
1.022499402	1.940327258	0.860412268	0.389042588	0.339927739	0.876362708
0.648509674	1.981921745	0.800958833	0.304602566	0.27559087	0.576423077
1.114966219	3.031643263	1.550141593	0.485014573	0.303296341	4.060901404
0.898443696	2.572194967	1.126619228	0.646266001	0.413081971	2.952219518
0.840372	1.291099332	0.866396908	0.234555299	0.219775123	0.821652345
1.04906204	1.330436819	0.600484953	0.265521391	0.344983675	0.521305144
0.753980961	1.220777265	1.24289119	0.246284886	0.263395025	0.54002571
0.833122185	0.636529817	0.923830447	0.381379711	0.201828246	0.744529026
0.908652142	0.962861368	0.935688353	0.378430428	0.247637207	0.630732422
0.818979929	4.329498732	1.304774055	0.31325295	0.444359735	0.970096385

P58417,NXPH1,NEUROLOGY	P15018,LIF,NEUROLOGY	O14594,NCAN,NEUROLOGY	Q96GP6,SCARF2,NEUROLOGY	Q96B86,RGMA,NEUROLOGY	Q9H3R2,MUC13,NEUROLOGY
P58417	P15018	O14594	Q96GP6	Q96B86	Q9H3R2
NXPH1	LIF	NCAN	SCARF2	RGMA	MUC13
NEUROLOGY	NEUROLOGY	NEUROLOGY	NEUROLOGY	NEUROLOGY	NEUROLOGY
0.900501135	0.642915037	1.874882261	0.589882698	0.377461132	0.630951055
1.198641309	0.571608787	1.284760996	1.012976005	0.461307291	0.841654481
0.88154126	0.630994791	1.088921534	0.799128825	0.539053362	0.441933132
1.270503192	0.61668258	2.714525431	0.954025676	0.766203754	0.720514815
1.32408891	1.39126766	1.358109684	0.582810995	0.519933854	0.879710051
0.905445681	0.516091938	0.432328545	0.514555999	0.435033981	0.941739534
0.936077575	0.715042054	0.882397128	0.458502022	0.542953292	0.815694031
0.832775771	0.917957967	1.055187954	0.658702988	0.522462719	0.480031172
1.048625839	0.594397519	0.923318309	0.380719402	0.318728515	0.264621099
1.007025349	0.768970416	0.63055757	0.34757591	0.264676131	0.552252519
1.013046221	0.795646776	0.787963113	0.564756157	0.358091456	0.952637998
1.124512735	0.63931551	1.568080908	0.936402051	0.644164026	0.984320468
0.704611536	0.435788491	0.915733686	0.561516375	0.282417227	0.651889797
1.100988958	0.364451817	0.708627815	0.407875286	0.336225255	0.404468709
0.43077308	0.622393975	0.943111337	0.478602554	0.253753566	0.463711689
0.713853533	0.614463832	0.657425809	0.296972243	0.239782787	0.384618483
1.293786891	0.612932454	1.684125907	0.597329964	0.559573676	0.588983858
1.101905116	1.021790905	1.623604226	0.465805636	0.43906304	0.7944344
0.934456881	0.66287098	1.593722005	0.825305409	0.630776142	0.689967351
1.075643496	0.575744249	0.967745769	0.612465294	0.44028207	0.791960291
0.959929261	0.561321802	0.916114609	0.453822486	0.266332427	0.427116045
0.948552809	0.600901322	0.747683707	0.429580377	0.257331505	0.847743663
0.885460589	0.407931834	0.845689517	0.455208689	0.276796959	0.599237577
0.972452711	0.525222272	0.527484278	0.348613417	0.318485589	0.461019602
1.275532802	0.621273318	0.99833783	0.493082173	0.394418739	0.880014988
1.038139271	0.655696455	0.740206649	0.41549413	0.391830064	0.603196516
0.90450476	0.542878028	0.989588061	0.541900544	0.366402182	0.49134218
0.809442217	0.520835612	0.882702996	0.520727318	0.335829297	0.449190711
0.811070934	0.660714999	0.766575609	0.555130937	0.565186927	0.586092386
1.036485547	0.511143401	1.351910833	0.345462256	0.353921179	0.460125718
0.595304622	0.464934697	0.660577621	0.308127638	0.412738522	0.364047852
0.753876445	0.465902507	1.36717697	0.652206173	0.414114034	0.435546905
0.6943813	0.380798579	0.527228403	0.359833148	0.324524657	0.791411536
1.465507143	0.341652124	0.840255508	0.391938717	0.344696845	0.760805721
1.179683625	0.448599526	0.943242089	0.377330337	0.375920631	0.711926397

Q99426,TBCB,NEUROLOGY	Q8TCZ2,CD99L2,NEUROLOGY	Q96FE7,PIK3IP1,NEUROLOGY	P15151,PVR,NEUROLOGY	P16278,GLB1,NEUROLOGY	Q08345,DDR1,NEUROLOGY
Q99426	Q8TCZ2	Q96FE7	P15151	P16278	Q08345
TBCB	CD99L2	PIK3IP1	PVR	GLB1	DDR1
NEUROLOGY	NEUROLOGY	NEUROLOGY	NEUROLOGY	NEUROLOGY	NEUROLOGY
1.126306907	0.372367741	0.410114882	0.402343603	0.81966142	0.468623108
0.457898581	0.40745143	0.499549657	0.809273915	0.655560121	0.489811992
0.995780728	0.33028881	0.421849413	0.55351718	0.738106039	0.514306396
0.81524184	0.353161505	0.385846788	1.695369808	0.668685621	0.875330654
1.353598617	0.353724977	0.44723345	0.647341993	0.77819305	0.413741047
1.042754834	0.386194628	0.350697731	0.410342361	0.652839385	0.430952271
0.520258307	0.382359069	0.425874425	0.541637676	0.656833675	0.433679153
0.480131002	0.331872272	0.545858908	0.548780073	0.619166791	0.465482876
0.734788015	0.322439412	0.307444947	0.389609295	0.765991347	0.408809322
0.984661667	0.273308077	0.378771582	0.296704765	0.492126121	0.310141824
0.753562982	0.294430697	0.279922624	0.409717097	0.652160967	0.337158763
1.672724673	0.492262587	0.670031116	0.560194607	0.866937564	0.618823547
0.570856486	0.328098295	0.366707074	0.280369244	0.64711768	0.397795814
0.816033339	0.304054111	0.335387308	0.205043228	0.74277646	0.352990191
0.933032992	0.279864422	0.246763343	0.29982654	0.489811992	0.295370981
0.911680359	0.283319145	0.4009516	0.277450056	0.636882881	0.339833504
1.13115764	0.44664484	0.350697731	0.407394949	0.59435632	0.444144183
0.791795624	0.354240238	0.533440771	0.588290237	0.410257041	0.388046111
1.068287529	0.509622189	0.386435623	2.274264419	0.631388549	0.976573138
0.734024438	0.290538247	0.41748608	0.327167195	0.450375421	0.349896466
0.68724671	0.273573425	0.341960122	0.536965015	0.682404942	0.369616227
1.0966476	0.427293714	0.384671806	0.340470097	0.648959342	0.301619164
0.547943862	0.327076497	0.360232437	0.347118461	0.534328917	0.322193658
0.624771262	0.250641984	0.329602708	0.403041416	0.694188803	0.308876068
0.704172113	0.326736606	0.400534939	0.567778451	0.670681634	0.3898254
0.713408347	0.419691166	0.391368623	0.317603783	0.690063007	0.371336748
0.866937564	0.324524657	0.488591273	0.473783546	0.563661135	0.34837186
1.143455325	0.309862484	0.363241262	0.423313968	0.761122197	0.351330322
0.861665604	0.327076497	0.413827091	0.372574283	0.709955252	0.43419048
0.964063446	0.346926031	0.382465097	0.486024181	0.78154454	0.38320811
0.783388579	0.312537238	0.408780987	0.454767166	0.461115479	0.344458002
0.844049784	0.310335361	0.379323327	0.367572311	0.687484933	0.420768906
0.686151948	0.262138289	0.314362274	0.248893421	0.676002272	0.369283319
0.549465193	0.321658117	0.358488812	0.401034984	0.743703775	0.405338748
1.26654646	0.332932127	0.445222991	0.468688077	0.641890894	0.386918067

Q9UNZ2,NSFL1C,NEUROLOGY	Q9P232,CNTN3,NEUROLOGY	Q9NR71,ASAH2,NEUROLOGY	P78423,CX3CL1,NEUROLOGY	Q16740,CLPP,NEUROLOGY	O96013,PAK4,NEUROLOGY
Q9UNZ2	Q9P232	Q9NR71	P78423	Q16740	O96013
NSFL1C	CNTN3	ASAH2	CX3CL1	CLPP	PAK4
NEUROLOGY	NEUROLOGY	NEUROLOGY	NEUROLOGY	NEUROLOGY	NEUROLOGY
0.884724389	0.385525983	0.592751782	0.582487906	1.221200428	0.774855931
0.924983798	0.199381022	0.255430021	0.877456798	0.868441154	0.904818292
1.109108111	0.290900968	0.441871871	0.795536484	1.146789023	0.984047595
1.33172851	0.123261999	0.206354957	0.594603558	0.807648806	1.774484534
0.755498083	0.229712298	0.343670995	0.830757893	0.965333934	1.159256648
0.658657332	0.245313749	0.439794053	0.669938236	1.062969351	0.864417389
0.716331853	0.299203716	0.393653988	0.48424196	0.948355583	0.944419673
0.501318716	0.287274133	0.323805636	0.505856469	0.756860856	0.916813377
0.933485812	0.253841525	0.222195268	0.215520947	0.816938851	1.092929241
0.959064667	0.270593671	0.161342677	0.23868834	0.838626312	0.919677532
0.69674372	0.264437741	0.174609021	0.43603021	0.746337458	0.858684467
1.681792831	0.390718101	0.38536568	0.81988871	1.145279719	0.865136691
0.740411906	0.169481534	0.19994845	0.582972607	0.856485068	1.221454396
0.753824192	0.196622455	0.26967618	0.446799662	0.828687462	0.981935386
0.781869643	0.178179097	0.231647015	0.383500403	0.691020301	1.048843917
0.906701766	0.184718466	0.13969962	0.341628443	1.761494536	0.901687858
1.665205236	0.183162999	0.213839459	0.662549431	0.93789609	0.789931796
0.736368593	0.266074102	0.289995014	0.459775023	0.921783597	6.322959593
1.512672844	0.210603308	0.133934543	0.447543556	0.799461243	0.98268436
0.631694975	0.295965311	0.217562226	0.678490255	0.662641287	1.291188827
0.579547976	0.206312051	0.269788358	0.386649968	0.770784786	1.262777097
0.934975198	0.21962284	0.435728082	0.467196056	1.424642565	0.990068329
0.729459608	0.208526118	0.323401887	0.428272219	0.804296864	0.997922719
0.525404332	0.220889998	0.334806629	0.465999399	0.80865711	1.010521488
0.581358502	0.228220454	0.379244457	0.470739232	0.958200851	0.996816601
0.879588105	0.246336105	0.246438574	0.585889297	0.859637307	1.12826034
0.605416542	0.207057017	0.192309242	0.475560238	1.028113827	1.099158927
2.234574276	0.123441551	0.384885173	0.357025741	1.070140335	0.874421028
1.481953315	0.256441205	0.383420665	0.575983744	1.056798263	0.729914811
0.762336575	0.195711441	0.413999233	0.559767643	1.290830883	1.040011874
0.67324336	0.209308093	0.241584534	0.438059881	0.578023479	0.633053792
0.833122185	0.252647884	0.182579918	0.664527128	0.730066608	0.85340354
0.969424198	0.223926938	0.138168438	0.270687468	0.696695428	0.953166399
0.675159373	0.270724996	0.564599595	0.470902406	1.045215193	0.946779255
1.533895288	0.311175418	0.315082165	0.382438587	0.928452495	0.999514915

Q9H3S3,TMPRSS5,NEUROLOGY	Q6ZMC9,SIGLEC15,NEUROLOGY	P52943,CRIP2,NEUROLOGY	Q9Y6E0,STK24,NEUROLOGY	Q13426,XRCC4,NEUROLOGY	Q9NRW1,RAB6B,NEUROLOGY
Q9H3S3	Q6ZMC9	P52943	Q9Y6E0	Q13426	Q9NRW1
TMPRSS5	SIGLEC15	CRIP2	STK24	XRCC4	RAB6B
NEUROLOGY	NEUROLOGY	NEUROLOGY	NEUROLOGY	NEUROLOGY	NEUROLOGY
0.668824685	0.688104699	0.534588238	1.115894007	0.83052759	0.760752988
0.47813834	0.974206828	0.490899635	1.057237864	0.774372701	0.879466177
0.555823986	0.879893	0.52624262	1.198724396	0.792729187	0.45584018
0.480863724	0.672916779	0.509445598	0.815015838	0.932321861	0.616768077
0.429610155	1.086132416	1.130687303	1.588208146	1.185667921	0.99896082
0.582164995	0.85577296	0.660577621	1.090054298	0.912628745	0.79680577
0.379691603	1.416764532	0.585848688	1.708227353	1.079153433	1.000207966
0.277450056	1.100531165	0.34992072	0.938156166	0.793058942	1.017973496
0.24449892	0.96072804	0.314493041	1.513197187	0.747631883	0.907456253
0.324344752	0.724521296	0.358414274	1.004724526	0.874784765	0.867418429
0.359708461	0.700860878	0.373065279	1.141000956	1.107341337	0.826393035
0.900938167	0.920187651	0.893971039	1.221793102	0.809610553	0.843874287
0.418906449	0.8005703	0.291122853	1.199638726	1.081100018	0.713556711
0.542201121	0.790315167	0.359858091	1.266283117	1.02129525	0.854291303
0.425697346	0.801680896	0.2382916	0.956210399	0.857435467	2.558503133
0.289452796	0.882397128	0.441933132	1.419221726	0.961260928	1.105347503
0.747424624	0.929353908	0.296211591	1.21959319	0.859756486	0.897696702
0.242843706	1.043839571	0.464161896	0.933679945	1.180828952	0.758068435
0.380271046	0.872181322	0.542276291	1.546171124	1.073111511	0.77292482
0.574309368	1.72955393	0.341202471	1.504203751	1.252317174	0.857851599
0.597909898	0.985685973	0.326442319	1.432862328	0.992335589	0.74046323
0.310012867	0.838103312	0.536518566	0.901937894	0.740104042	0.745096918
0.361959453	0.809386112	0.418094219	1.302605298	0.864417389	0.809386112
0.290216208	0.987600861	0.295780736	1.267776121	1.207731706	1.03857111
0.495927111	0.883070178	0.420739741	1.171372557	1.160462578	0.750643603
0.448381917	1.171859818	0.336038864	1.261202557	1.212428777	0.860352631
0.345821627	0.826622192	0.394692224	1.467336746	0.816712379	0.637280314
0.173198699	0.795481343	0.397492625	1.172753657	0.803795274	0.558449991
0.402510968	1.139183377	0.28614137	1.26111514	1.102363481	0.929804943
0.437726005	1.125604499	0.491069798	0.995918783	0.882091365	1.131941971
0.248617542	0.676986983	0.367929179	0.988422666	0.804966138	0.622739199
0.371336748	0.684869017	0.358339752	0.922294884	0.701346846	0.675252977
0.399647508	0.814112457	0.546729831	1.157811186	0.645862965	0.613357453
0.441229146	0.815807118	0.392047401	1.187641984	0.876848802	0.863519104
0.319702072	0.962928111	0.518781882	1.067695309	0.732296599	0.805747661

O94985,CLSTN1,NEUROLOGY	Q16288,NTRK3,NEUROLOGY	P48740,MASP1,NEUROLOGY	P22894,MMP8,NEUROLOGY	P29466,CASP1,NEUROLOGY	Q07011,TNFRSF9,NEUROLOGY
O94985	Q16288	P48740	P22894	P29466	Q07011
CLSTN1	NTRK3	MASP1	MMP8	CASP1	TNFRSF9
NEUROLOGY	NEUROLOGY	NEUROLOGY	NEUROLOGY	NEUROLOGY	NEUROLOGY
0.563895604	0.306997753	0.68700857	0.468265937	0.250364167	1.076837085
1.001803808	0.477409771	0.794985251	0.487880592	0.333810213	1.279162899
0.727036651	0.501701098	1.102592735	0.591028662	0.282260665	1.59604353
0.767905135	0.829664522	0.552941975	0.420331651	0.325290366	0.556595056
0.720165304	0.450469084	1.477952599	0.449252987	0.282985493	0.857851599
0.594603558	0.49086561	0.989725257	0.47243901	0.269956713	1.018679346
1.52467315	0.417341415	0.586214273	0.449533333	0.246063061	0.935364124
1.145835547	0.428925799	0.55144924	0.391016123	0.201060279	1.202052577
0.427975466	0.296211591	0.689489267	0.599196042	0.178934068	0.664527128
1.215710738	0.340092713	0.633580571	0.256352345	0.181281047	0.669659675
0.726683976	0.383367515	0.558527414	0.576862745	0.388853869	0.627201102
1.118682008	0.452158346	0.833295446	0.736266517	0.37837797	1.631501145
0.701006633	0.436181353	0.688963758	0.305046271	0.279302424	0.950659101
0.955150515	0.208989157	0.670728123	0.264621099	0.14919799	0.815976778
0.610981245	0.352427889	0.508316864	0.475000191	0.292376649	0.558179096
0.61839476	0.283731847	0.60924736	0.596543811	0.191378411	0.59828301
0.968081222	0.426968043	0.688677286	0.851925985	0.259498841	0.732245841
0.467228441	0.43615112	0.691020301	0.308148997	0.341723176	0.784910465
0.862083788	0.654425105	0.670031116	0.467163673	0.40983071	0.78192384
1.96577745	0.348227007	0.741336269	0.479100426	0.480797067	0.957669659
0.514235103	0.375842469	0.813097351	0.316483019	0.331274716	0.874542257
0.532221981	0.258117525	0.565461224	0.411510173	0.290196093	0.943307472
0.760963943	0.301765546	0.554323471	0.538194667	0.276777774	0.997922719
1.065625123	0.329191731	0.644744739	0.324727169	0.280699811	0.64967946
1.017691293	0.319524841	0.612847489	0.376311687	0.23411674	1.348074287
1.447035183	0.452597337	0.658246568	0.364426556	0.228743079	0.888534685
0.65747138	0.347070343	0.931546699	0.416763258	0.228220454	0.689059276
0.560155779	0.31892741	0.729307937	0.489642265	0.215700287	0.73163703
1.129277464	0.350673423	0.770784786	0.736266517	0.145470649	0.969693017
0.751684937	0.336062157	1.140605583	0.288551353	0.448941696	0.49338987
0.508035071	0.306508715	0.749603711	0.21581993	0.080626617	0.513095757
0.658429098	0.330174361	0.784094802	0.446551972	0.215446266	1.429390396
0.457010748	0.287214402	0.612083338	0.297796768	0.244515868	0.762547969
0.349654021	0.306636215	0.755131602	0.235712472	0.254017536	0.642514091
1.37249415	0.305427104	0.501318716	0.709512497	0.374023287	0.679290225

P01178,OXT,NEUROLOGY	P50135,HNMT,NEUROLOGY	O00214,LGALS8,NEUROLOGY	P31948,STIP1,NEUROLOGY	Q8TCT1,PHOSPHO1,NEUROLOGY	P30086,PEBP1,NEUROLOGY
P01178	P50135	O00214	P31948	Q8TCT1	P30086
OXT	HNMT	LGALS8	STIP1	PHOSPHO1	PEBP1
NEUROLOGY	NEUROLOGY	NEUROLOGY	NEUROLOGY	NEUROLOGY	NEUROLOGY
0.501422973	0.36680876	0.487306037	1.230035539	0.530380633	0.632352102
0.733820951	0.23607219	0.48454414	0.969289817	0.635075491	0.481130446
0.04360387	0.29211331	0.259786794	1.197810762	0.52007803	0.648644541
0.0568656	0.254828757	0.511356024	1.130608932	0.418239144	0.514555999
0.05419111	0.447016503	0.354412158	1.00556058	0.706763774	0.59238212
0.106579361	0.299245198	0.368388518	1.420796566	0.744529026	0.598490395
0.126516723	0.158318308	0.418152183	1.383957845	0.316088401	0.573672792
0.3188832	0.163232473	0.323065814	0.964063446	0.414286295	0.406943384
0.053652901	0.189044791	0.309926925	1.398228358	0.330884588	0.511994423
0.055042889	0.190994103	0.316285649	1.377450046	0.28839139	0.541900544
0.104140273	0.176813459	0.387562261	1.106804182	0.436272064	0.497821347
0.898007874	0.531374168	0.458184322	1.444629964	0.71276579	1.172916247
0.083666854	0.123219287	0.348951878	0.988696753	0.336808394	0.34193642
0.272004043	0.165821157	0.341344403	1.299088755	0.307189328	0.548209791
0.083037107	0.12583455	0.239185195	1.357262716	0.236629197	0.420768906
0.061158634	0.086497365	0.463808125	1.055261097	0.231117755	0.627592493
0.033013502	0.229871578	0.518314622	2.080456322	0.382889499	1.18304094
0.159773495	0.30168189	0.489574391	1.146630055	0.645773435	0.579668502
0.311758327	0.300159243	0.594768439	1.192426217	0.496787232	0.601734925
0.047405557	0.217562226	0.218741673	1.052558194	0.412281034	0.42281545
0.292174059	0.260634505	0.351647045	0.776253622	0.343980813	0.452816992
0.264712825	0.428005132	0.419603902	1.647524592	0.378902877	0.811633321
0.11208771	0.318750608	0.430385089	1.050080551	0.395458991	0.447357466
0.610938896	0.185128639	0.214136109	0.977860112	0.394500764	0.416849931
0.065661886	0.230669634	0.418355121	0.967947027	0.414458628	0.454105684
0.075065822	0.171811677	0.412595502	1.551861709	0.329419988	0.671472397
0.538418542	0.171205387	0.472177107	1.233450654	0.25396472	0.538082764
0.104046475	0.139148764	0.530417397	1.918794222	0.252560338	0.936986392
0.312299031	0.152237956	0.494348377	1.475291457	0.342933322	0.739181216
0.689537061	0.179866698	0.370668134	1.187724308	0.419516657	0.508528311
0.073986199	0.162386094	0.314144451	1.071996355	0.458724542	0.5163782
0.516199268	0.230286222	0.475692109	1.199971382	0.469598596	0.442791674
0.091454629	0.160428237	0.316110311	1.038859103	0.29278225	0.447574578
0.064762388	0.187738963	0.389285361	1.06873191	0.287752427	0.461371246
0.04047851	0.182883903	0.428688018	2.097833239	0.296622512	1.002845945

O60240,PLIN1,NEUROLOGY	Q9Y2W6,TDRKH,NEUROLOGY	P07108,DBI,NEUROLOGY	Q6UWL6,KIRREL2,NEUROLOGY	Q2MKA7,RSPO1,NEUROLOGY	O15117,FYB1,NEUROLOGY
O60240	Q9Y2W6	P07108	Q6UWL6	Q2MKA7	O15117
PLIN1	TDRKH	DBI	KIRREL2	RSPO1	FYB1
NEUROLOGY	NEUROLOGY	NEUROLOGY	NEUROLOGY	NEUROLOGY	NEUROLOGY
1.042321254	0.511781535	0.421761701	1.295851141	0.468330857	0.377330337
1.033687427	0.936596791	0.280719269	2.345019631	0.384431911	0.43642329
0.780732374	0.857732683	0.308833252	2.342095657	0.435124453	0.40595733
0.934068332	0.830354904	0.338563887	1.114425365	0.315082165	0.412881591
1.09065892	1.191104507	0.487373596	1.70491522	0.386837618	0.565970985
0.977047089	0.753615216	0.435365804	1.624842634	0.390474435	0.33940977
0.863938187	0.972789795	0.35026045	1.41460572	0.424813053	0.462267551
0.950856806	1.105807299	0.292072817	1.744846351	0.425225494	0.395897812
1.091717815	1.102287073	0.261793285	1.310756772	0.41989485	0.65012994
0.823077395	0.918785504	0.248979696	1.178457721	0.300180049	0.496821668
1.011993483	0.972250516	0.344672954	1.324823345	0.371800341	0.467228441
1.489264494	1.29020472	0.924855576	3.176856667	0.607602622	0.695440986
0.938286231	0.77840884	0.215013627	2.207479439	0.347889249	0.289492926
1.179683625	0.788072356	0.310658191	1.345460466	0.309261683	0.455619058
0.950263815	0.782303324	0.18833852	1.172834949	0.198292223	0.229823782
0.754503762	1.10343374	0.300096833	1.01094184	0.364906815	0.505856469
1.151089491	0.797358265	0.327212553	1.20505584	0.622998243	0.614038066
1.417157397	1.112727246	0.403684472	2.007221738	0.356728899	0.489676206
1.129199191	0.942980603	0.368056716	1.65278607	0.414631032	0.773031978
1.080276034	0.915479826	0.307359717	2.054375639	0.316132223	0.349823715
0.867238073	0.996954798	0.368158777	1.514246419	0.299514966	0.503128912
1.150291893	0.958532996	0.586864766	1.228246391	0.468395785	0.425166549
1.272353895	1.017550221	0.411766966	1.669944319	0.253929515	0.56663829
1.008072919	0.950066234	0.241953213	1.556817681	0.309561938	0.276221974
1.221793102	1.125994671	0.256316809	2.035382586	0.404749162	0.436060434
1.306855835	1.313121125	0.299037849	1.057677648	0.33081579	0.421148228
0.904567458	0.867298188	0.258852113	1.292711197	0.357397141	0.482299092
0.949144731	0.657289116	0.33010571	1.229609315	0.322260663	0.74442582
0.96761162	0.970432653	0.295473367	2.536255344	0.354805431	0.463197702
1.049207481	0.718370494	0.526060269	1.28788163	0.400673778	0.348154602
0.665633524	0.896080346	0.264694477	1.106727467	0.396144863	0.373349835
0.686104389	0.648824409	0.255270725	1.651182974	0.378141999	0.340588116
0.683683257	0.673383371	0.254387555	1.073185896	0.310163323	0.415235012
1.020375384	0.985207831	0.276796959	1.407953805	0.230477848	0.399509025
0.920251435	1.095735813	0.265742338	1.269534851	0.249031475	0.905320169

O76076,WISP2,NEUROLOGY	Q9H477,RBKS,NEUROLOGY	P07741,APRT,NEUROLOGY	P38484,IFNGR2,NEUROLOGY	Q14126,DSG2,NEUROLOGY	Q01469,FABP5,NEUROLOGY
O76076	Q9H477	P07741	P38484	Q14126	Q01469
WISP2	RBKS	APRT	IFNGR2	DSG2	FABP5
NEUROLOGY	NEUROLOGY	NEUROLOGY	NEUROLOGY	NEUROLOGY	NEUROLOGY
0.058338819	0.481597564	1.22069265	0.519105616	0.430444757	0.389258379
0.259570799	0.374490234	1.163926534	0.426494783	0.39329943	0.437574327
0.094509293	0.519321551	1.431373326	0.433288545	0.59238212	0.383075323
0.129157202	0.348734258	0.864237657	0.374983755	0.440099	0.64429799
0.091810311	0.44844408	1.27041513	0.504980646	0.315563008	0.600110468
0.241082695	0.52660751	1.307580711	0.45875634	0.443897965	0.34642141
0.287493252	0.368643954	1.431373326	0.325831954	0.416907722	0.318022336
0.138082271	0.261811432	0.79874118	0.266313967	0.330426202	0.316088401
0.060033018	0.392319242	1.283070106	0.305914416	0.266092546	0.336855089
0.185205648	0.308234446	1.370022892	0.56420838	0.247568557	0.26197481
0.106608916	0.232386795	0.830470024	0.348903506	0.298458035	0.400368396
0.273933953	0.708431369	2.567919541	1.001387256	0.499134316	0.942065973
0.121102454	0.218954044	0.695537401	0.352110462	0.331205836	0.235990387
0.15824151	0.274275944	1.243752995	0.302582404	0.291365103	0.275476279
0.014970851	0.317339717	0.837812898	0.276720226	0.235957674	0.315716157
0.131132755	0.456662427	1.529754348	0.590128075	0.279651117	0.362310871
0.107551533	0.784149153	3.454315841	0.675861716	0.361357815	0.267145941
0.125182084	0.3188832	0.904065998	0.341841628	0.485317235	0.418007288
0.070106888	0.291991849	1.056578531	0.362411339	0.334087984	0.757858283
0.179654876	0.358737384	0.934586433	0.267906223	0.317405713	0.35769454
0.029805038	0.219546737	0.698775049	0.376337772	0.276356031	0.413282448
0.076590008	0.42699764	1.846892634	0.424106941	0.295616766	0.546275263
0.133443413	0.274637398	0.867598822	0.346661614	0.315278785	0.373815942
0.139100547	0.25151215	0.953959551	0.257670628	0.254070363	0.224501968
0.099669783	0.392645699	1.212008654	0.356803086	0.40323702	0.314100904
0.136824643	0.460157612	1.435347442	0.313622289	0.399453645	0.303275319
0.300200857	0.317978252	1.308668778	0.320967694	0.402455172	0.232951254
0.099249249	0.576103529	2.755664019	0.308298548	0.314907495	0.402706315
0.176067435	0.508035071	1.868525173	0.34719065	0.419342221	0.316417215
0.096521892	0.262174632	1.283603831	0.382147103	0.345007589	0.396639428
0.364957405	0.262556533	0.879953992	0.553018635	0.432118829	0.24884167
0.096856991	0.481197149	1.272706715	0.371336748	0.264786229	0.312212456
0.212701189	0.292640226	1.344528187	0.279360509	0.276394344	0.241099406
0.115711166	0.255376911	1.042610287	0.290921132	0.37845666	0.337112026
0.071813555	0.744580635	2.903111535	0.596295767	0.347046287	0.318000293

Q8N474,SFRP1,NEUROLOGY	Q6NW40,RGMB,NEUROLOGY	Q9UK53,ING1,NEUROLOGY	O15197,EPHB6,NEUROLOGY	P02771,AFP,NEUROLOGY	Q96GW7,BCAN,NEUROLOGY
Q8N474	Q6NW40	Q9UK53	O15197	P02771	Q96GW7
SFRP1	RGMB	ING1	EPHB6	AFP	BCAN
NEUROLOGY	NEUROLOGY	NEUROLOGY	NEUROLOGY	NEUROLOGY	NEUROLOGY
0.556595056	0.43149029	0.620670723	0.620799801	0.245977797	1.268303484
0.398430501	0.544951597	0.775769521	0.598448913	0.244532817	1.05416449
0.965668552	0.471653736	0.625854847	0.627418511	0.157235621	0.815920221
0.326940498	0.59184857	0.658109704	0.551372798	0.211598309	0.934716004
0.404973665	0.493868891	0.791411536	0.62876813	0.227194529	1.57210764
0.895087111	0.469208157	0.606466558	0.595510974	0.269321255	0.549503281
0.447729723	0.406633224	0.741387656	0.461755164	0.161443359	0.7275912
0.50375704	0.496511831	0.930256196	0.553478814	0.141669392	0.5522908
0.345294677	0.376833727	0.576862745	0.325019909	0.157094002	0.538008175
0.367546834	0.270706231	0.758278646	0.386034047	0.13473532	0.504980646
0.515591362	0.397409978	0.887611337	0.415522931	0.336738364	0.674504512
1.202469249	0.646669288	0.855120715	0.705491204	0.31914855	0.973666763
0.672870138	0.379612657	1.035193167	0.387105846	0.244397257	0.596957447
0.886443141	0.391694289	0.911996378	0.393381223	0.091056133	0.852871325
0.328485137	0.317383713	0.609712065	0.226565485	0.294941349	0.532960309
0.467487599	0.255164583	0.903439564	0.272494686	0.173426949	0.537635385
0.835319487	0.360882227	0.629334963	0.565069411	0.120608208	0.799627503
0.651122095	0.383234673	0.732347359	0.514984173	0.121767413	0.930836701
0.813604746	0.449751501	0.813548353	0.463422501	0.212774918	0.831852705
0.469403336	0.353724977	1.056212412	0.357793727	0.156517947	0.64166847
0.351549561	0.29498224	0.811014717	0.351842094	0.155987248	0.560505331
0.501075533	0.317691853	0.906513242	0.368516214	0.128817455	0.703489112
0.528765517	0.361984543	0.777438253	0.376077005	0.192202632	0.765991347
0.213809817	0.310421416	0.759330572	0.314362274	0.256530096	0.549846186
0.708971727	0.422962012	1.068805991	0.507718242	0.184923439	0.508598813
1.219508657	0.318264908	0.959796196	0.397409978	0.136767751	0.826106679
0.299847323	0.433468782	0.734686159	0.506698689	0.204206402	0.590496331
0.466678206	0.429401757	0.712074453	0.342671948	0.141130333	0.769290288
0.743600683	0.502501567	0.866336856	0.417833479	0.325380568	0.558875951
0.612805011	0.336225255	0.642335973	0.579990028	0.5749068	0.920506619
0.287154684	0.39696948	0.562256368	0.40796011	0.376233443	0.60098463
0.647656162	0.410484599	0.333856492	0.394637511	0.359085674	0.92312633
0.357149498	0.3304033	0.408951029	0.346205368	0.173920512	0.539614118
0.309519026	0.332816762	0.936207352	0.480097723	0.113165021	0.657927263
0.430952271	0.312472255	1.062822003	0.454389058	0.20389524	0.529756027

**Q6ZMJ2,SCARA5,NEUROLOGY**	**P30039,PBLD,NEUROLOGY**	**Q8N149,LILRA2,NEUROLOGY**	**P04179,SOD2,NEUROLOGY**	**P15289,ARSA,NEUROLOGY**	**Q9NQ88,TIGAR,NEUROLOGY**
Q6ZMJ2	P30039	Q8N149	P04179	P15289	Q9NQ88
SCARA5	PBLD	LILRA2	SOD2	ARSA	TIGAR
NEUROLOGY	NEUROLOGY	NEUROLOGY	NEUROLOGY	NEUROLOGY	NEUROLOGY
0.387481678	0.212303491	0.535292743	0.427441829	0.466322517	0.620455652
0.677503357	0.425431865	0.581318207	0.460987648	0.51845835	0.692938874
0.42658348	0.459838766	0.586417475	0.424695286	0.57125231	0.544234379
0.541900544	0.401535654	0.485653748	0.56178889	0.49806295	0.476120945
0.28973382	0.499965344	0.444791154	0.428361285	0.41942943	1.010171329
0.454798689	0.614932516	0.371723036	0.491785124	0.257420704	0.931288456
0.516987033	0.464451546	0.39052857	0.595510974	0.534551184	1.081549728
0.509480912	0.597288562	0.40779048	0.420535646	0.56007813	1.359616712
0.374853818	0.557985679	0.268184916	0.290921132	0.399342909	0.828457733
0.245602985	0.385793302	0.392183298	0.338352745	0.285705359	0.856722569
0.347961598	0.511958935	0.245637035	0.452503231	0.405872922	0.570065659
0.6816958	0.335340817	0.562178428	0.494040082	0.483470578	0.475098975
0.41091161	0.330334601	0.353259435	0.299431935	0.29233612	0.728196645
0.38371312	0.41313924	0.290719551	0.350965226	0.87151657	0.928388141
0.174294627	0.439397937	0.204347996	0.313057593	0.309798057	0.895211206
0.239865903	0.480164284	0.426110646	0.293290043	0.369001862	0.792784137
0.288991707	0.460030047	0.51845835	0.605290662	0.448661719	1.110338834
0.346541491	0.592505315	0.47883483	0.462523957	0.581237625	0.60625641
0.394118124	0.420389925	0.380824975	0.473094404	0.339621571	0.8630404
0.459042616	0.558721019	0.546313129	0.333278464	0.358141101	0.878734962
0.2880917	0.937441131	0.306317565	0.302309873	0.711285175	1.267600382
0.37030861	0.409773899	0.508563561	0.309841007	0.855476423	0.827768928
0.336201951	0.360007783	0.484644908	0.344147754	0.64429799	0.742107452
0.206440795	0.494416913	0.260201286	0.346829856	0.371130893	0.947961255
0.359808207	0.523622864	0.543141498	0.396309649	0.43970261	1.21369002
0.281186649	0.588575747	0.381300413	0.42889607	0.383739718	1.100760038
0.548399819	0.451438073	0.601192951	0.517596584	0.486226355	0.669102899
0.421878655	0.415177452	0.472668294	0.365489027	0.935947816	0.859518144
0.580673862	0.451782408	0.650851357	0.526972653	1.282803326	1.203720131
0.312385631	0.430265777	0.270875159	0.369257723	0.422083401	0.860770178
0.33641175	0.31822079	0.348178735	0.33854042	0.344649063	0.63529563
0.442055679	0.471457621	0.365641062	0.431191308	0.469305736	0.761491585
0.278722234	0.395513817	0.391287248	0.325019909	0.391612847	0.65012994
0.343075974	0.381697065	0.433498829	0.484913727	0.426435663	0.767639045
0.26611099	0.532258873	0.472504508	0.378719077	0.452095668	0.754033225

Q9UHV9,PFDN2,NEUROLOGY	P39905,GDNF,NEUROLOGY	P19438,TNFRSF1A,NEUROLOGY	P07196,NEFL,NEUROLOGY	P41208,CETN2,NEUROLOGY	Q03393,PTS,NEUROLOGY
Q9UHV9	P39905	P19438	P07196	P41208	Q03393
PFDN2	GDNF	TNFRSF1A	NEFL	CETN2	PTS
NEUROLOGY	NEUROLOGY	NEUROLOGY	NEUROLOGY	NEUROLOGY	NEUROLOGY
0.76684133	0.476385035	0.437574327	0.3187948	0.863519104	0.780407745
1.063190412	0.750747671	0.536221139	0.410370804	1.089298991	1.0966476
0.935623498	0.620584685	0.432088878	0.443129415	1.136738178	1.02002181
0.827310043	0.610050255	0.435456345	0.465353834	1.287167674	0.794985251
1.07840568	0.688200097	0.515913105	0.597537019	0.92108104	0.727742514
0.893661266	1.558653238	0.496099016	0.349024448	1.213858285	0.982752477
0.918085232	0.70120102	0.409234591	0.325403123	1.135871789	0.703391594
1.339876472	0.643360827	0.500658924	0.302561432	0.995021773	0.707351888
1.19052672	0.78561806	0.292883739	0.21453724	0.675299783	1.253185515
1.182549029	0.577983415	0.298271905	0.381564802	1.104505039	0.561243991
0.692794797	0.739693755	0.335201381	0.370822323	0.910101905	0.557599047
1.237732885	1.274472285	0.679996865	0.550341871	1.084552582	1.541355879
0.905696759	0.636927028	0.263979902	0.268575573	0.848684362	0.654878876
1.35106773	0.31024933	0.325719049	0.211173396	0.496511831	0.746389192
0.930578654	0.580030231	0.211994685	0.380666627	1.199056799	0.653382627
1.666937485	0.609796595	0.285962921	0.494896931	0.967075211	0.630819866
0.88976731	0.842179696	0.460732093	0.447202451	0.940500099	0.773782497
1.214110726	0.651483254	0.395486403	0.382597672	1.333206263	0.803906711
1.510158524	0.728247122	0.379849545	0.36366954	1.122099026	0.699211103
1.860254489	0.870852325	0.402929684	0.271740217	0.95210989	0.67007756
1.507230433	0.957802429	0.41529258	0.347094401	0.981527096	0.684252165
1.029040669	0.562178428	0.457200853	0.289352497	1.178621101	0.947567091
1.429984986	0.562139462	0.360607173	0.387401112	0.96279463	0.70759708
1.382423838	0.788454824	0.319502694	0.286478743	1.110184919	0.673150035
1.403666302	0.822564092	0.40228783	0.310313851	0.870188587	0.63529563
2.108621276	0.821994132	0.311693505	0.433679153	1.007444246	0.858684467
1.186572292	0.682783453	0.444883655	0.263048367	1.145756126	0.986916546
0.913894794	0.429104222	0.370077672	0.49297965	1.247552043	0.891001645
1.419516875	0.897012503	0.415062357	0.440007494	1.083575741	1.135714334
0.935623498	0.502048971	0.378692827	0.443836432	0.848625538	0.504420915
0.561360711	0.441382091	0.335759471	0.567424363	0.479299719	0.373168729
0.540175458	0.455208689	0.383792919	0.391070333	0.914782074	0.840313752
0.604787405	0.544913825	0.302792211	0.391612847	0.743961568	0.438880477
0.816599166	0.606760889	0.313057593	0.5538242	1.138630776	0.641846403
1.475700551	0.672543738	0.357347599	0.572481112	1.109492565	1.096343587

O15394,NCAM2,NEUROLOGY	O60609,GFRA3,NEUROLOGY	Q9Y279,VSIG4,NEUROLOGY	Q13451,FKBP5,NEUROLOGY	Q6YHK3,CD109,NEUROLOGY	Q96FQ6,S100A16,NEUROLOGY
O15394	O60609	Q9Y279	Q13451	Q6YHK3	Q96FQ6
NCAM2	GFRA3	VSIG4	FKBP5	CD109	S100A16
NEUROLOGY	NEUROLOGY	NEUROLOGY	NEUROLOGY	NEUROLOGY	NEUROLOGY
0.400423902	0.636794597	0.241032568	0.506488002	0.649544377	0.633492744
0.428806892	0.571054364	0.295227701	0.383234673	0.733668373	0.68477408
0.661860923	0.54559412	0.250398877	0.574150158	0.908778116	0.429491058
0.390799357	0.566088688	0.249325094	0.534254848	0.611659221	0.861367025
0.383872735	0.665725807	0.27814325	0.693563557	0.790096075	0.71227191
0.380323766	0.74385844	0.356061906	0.686675312	0.49239909	0.500520131
0.646938286	0.472963252	0.267164458	0.565814087	0.674224052	0.635339667
0.586986814	0.402176308	0.306742505	0.50278029	0.697178507	1.437338636
0.337041933	0.341723176	0.203232072	0.431280981	0.559263468	0.509728173
0.421294212	0.30266631	0.214715761	0.489710149	0.472340779	0.353724977
0.372445181	0.399647508	0.213602435	0.451939011	0.726180452	0.411396094
0.607602622	0.706714787	0.449564493	0.698048895	0.770731361	1.232425125
0.404132421	0.418674222	0.198553542	0.500971348	0.503407983	0.49297965
0.297507924	0.406605039	0.075107459	0.377565801	0.432358513	0.275380823
0.303191245	0.256530096	0.141905263	0.437392382	0.425992519	0.363216085
0.323177799	0.29197161	0.210224104	0.51270469	0.38315499	0.580593369
0.403684472	0.455334917	0.253138702	0.760436665	0.540400157	0.64015803
0.73417709	0.636838738	0.197990073	0.453130969	1.004933475	0.873694007
0.478204629	0.396584446	0.233517083	0.556479328	0.66840758	1.114657127
0.518638065	0.531153222	0.216749414	0.487846776	0.693563557	0.683067473
0.388611365	0.442392858	0.259283086	0.399924619	0.695007282	0.687580245
0.250920109	0.39727227	0.289412673	0.803962436	0.446026088	0.614634221
0.413196517	0.371439719	0.219958003	0.41302471	0.607644739	1.104351932
0.447357466	0.295944797	0.174234231	0.33464422	0.513665114	0.933097667
0.417862442	0.442699608	0.309841007	0.484913727	0.569394317	0.664158738
0.351038215	0.353381887	0.237516559	0.420244254	0.651392945	1.121399242
0.424989764	0.375087737	0.248893421	0.494965543	0.583659958	0.318309032
0.333694543	0.43708931	0.190254173	0.586824089	0.726029463	0.539763752
0.424695286	0.484644908	0.307040315	0.502292626	0.755707581	0.536853368
0.292498271	0.453413735	0.311844777	0.653382627	0.408780987	0.789877044
0.413110605	0.338516955	0.297260567	0.49373198	0.497304021	0.508211174
0.502710595	0.480997066	0.21459673	0.465515142	0.810115772	0.827654183
0.252245424	0.300533975	0.210749337	0.399121527	0.404805276	0.788072356
0.410484599	0.62563798	0.193365189	0.528106206	0.502571233	0.449657987
0.386087567	0.34077703	0.293635845	0.713260013	0.440526282	0.509798841

O00399,DCTN6,NEUROLOGY	O95466,FMNL1,NEUROLOGY	Q99727,TIMP4,NEUROLOGY	P00352,ALDH1A1,NEUROLOGY	O95994,AGR2,NEUROLOGY	O43505,B4GAT1,NEUROLOGY
O00399	O95466	Q99727	P00352	O95994	O43505
DCTN6	FMNL1	TIMP4	ALDH1A1	AGR2	B4GAT1
NEUROLOGY	NEUROLOGY	NEUROLOGY	NEUROLOGY	NEUROLOGY	NEUROLOGY
0.367827181	0.347262854	0.283201341	0.531521517	0.217818742	0.439580715
0.464097554	0.645325974	0.313470155	0.329146098	0.268780429	0.396364593
0.389501287	0.726583244	0.324862247	0.508316864	0.793553833	0.376703149
0.447636629	0.376129144	0.337626488	0.493526686	0.689154806	0.523405141
3.418823632	0.492740513	0.333602036	0.401257427	0.940043876	0.422991331
0.418935487	0.329283014	0.335201381	0.386141093	0.368720619	0.22131912
0.322528823	0.349484409	0.308277179	0.350042015	0.662319849	0.342624447
0.837812898	0.336248561	0.210968572	0.25233286	0.647252259	0.34630137
0.434762677	0.637280314	0.225703391	0.449502174	0.002358851	0.27612626
0.799904681	0.49373198	0.198113624	0.341723176	0.076510417	0.249584457
0.528289265	0.630120652	0.276049712	0.340942416	0.336598348	0.252472822
0.526060269	0.617366883	0.472504508	0.675206174	0.331596343	0.416792147
0.23594132	0.596998826	0.193955824	0.249722895	0.14958112	0.28631993
0.545216074	0.464805808	0.237056031	0.335503565	0.392618484	0.286220716
0.544272104	0.186649041	0.181847374	0.334922685	0.316483019	0.235467524
0.438272481	0.42552034	0.300888318	0.380455599	0.421761701	0.302477555
2.572551574	0.370231615	0.342743212	0.734227981	0.860829844	0.473947775
3.559809404	0.711581052	0.327280602	0.369821242	0.253859121	0.503233545
0.277661682	0.638031696	0.290820324	0.418297129	0.949539552	0.358986128
0.587108887	0.481630947	0.263614202	0.316483019	0.809554437	0.254493374
0.483001642	0.145047768	0.193298185	0.357471468	0.383287804	0.261430614
0.763658749	0.647162537	0.178636649	0.559690048	0.141649754	0.221718337
0.482299092	0.398844973	0.195142511	0.348323569	0.773728864	0.264511069
0.322685353	0.347070343	0.195697876	0.311024471	0.572481112	0.259085468
0.733414147	0.617024637	0.325132572	0.322439412	0.64743174	0.293982055
0.624165274	0.62563798	0.293188414	0.518494288	0.778840602	0.355691894
0.415695778	0.388638303	0.265135177	0.378797838	0.281245126	0.330999284
0.617880608	0.721414337	0.160383763	0.646669288	0.6943813	0.360707169
0.509728173	0.365767805	0.243991027	0.316307573	0.463454624	0.380033894
0.486125258	0.362763196	0.352550052	0.348758431	0.825820421	0.348565092
0.418935487	0.227888496	0.208222807	0.232209677	0.411025555	0.22520332
0.358314914	0.399924619	0.222333924	0.429610155	0.381617702	0.322126666
0.416676604	0.672357295	0.162521219	0.363014731	0.982480038	0.259696774
0.434009943	0.345390426	0.231936214	0.335457057	0.561127296	0.317295727
0.407084445	0.703489112	0.322171326	0.70514898	1.245305751	0.47083713

P23280,CA6,NEUROLOGY	Q9Y4K4,MAP4K5,NEUROLOGY	P02533,KRT14,NEUROLOGY	O60462,NRP2,NEUROLOGY	Q92932,PTPRN2,NEUROLOGY	P53539,FOSB,NEUROLOGY
P23280	Q9Y4K4	P02533	O60462	Q92932	P53539
CA6	MAP4K5	KRT14	NRP2	PTPRN2	FOSB
NEUROLOGY	NEUROLOGY	NEUROLOGY	NEUROLOGY	NEUROLOGY	NEUROLOGY
0.337696703	0.895149156	0.574229757	0.414889773	1.305045403	0.766469346
0.206069086	0.730218437	0.788837477	0.47355372	1.436243138	0.893165852
0.130561369	0.794819956	0.567896529	0.737543476	0.970029145	0.768863821
0.271533104	0.625507895	0.530748392	0.365818515	1.672029151	1.040300267
0.211642314	0.922167035	0.638429845	0.458819941	1.77743894	0.758120982
0.688247801	0.970096385	0.553402091	0.457105791	0.576702827	0.640246781
0.341131527	0.808320869	0.586417475	0.454263092	0.845220697	0.708185888
0.210355289	1.901713612	0.582649428	0.567306382	0.848272678	0.688152396
0.263979902	0.713705107	0.579708683	0.494554013	1.029040669	0.554784736
0.246592359	0.983024992	0.658520382	0.413741047	0.580191072	0.740052743
0.206211972	0.981595132	0.692842819	0.367470413	0.806585849	0.659388212
0.397988873	1.008282564	0.501909793	0.590414476	1.180992661	0.90000193
0.420186	0.85660381	0.78024548	0.480997066	0.935040008	0.723367156
0.272230384	0.659936903	0.516020398	0.313752748	0.843640348	0.777061129
0.201534677	0.606466558	0.590946733	0.372910158	0.94579538	0.770250703
0.319414121	0.828515159	0.776791866	0.327870953	1.032255425	0.830412462
0.763870509	1.161750286	0.69011084	0.393708564	1.526999934	0.792344646
0.352427889	0.721414337	0.643583837	0.774694821	1.143851685	0.767000807
0.712815197	0.611913656	0.662182138	0.394418739	1.281825611	1.072814023
0.277757929	1.421387582	0.79366385	0.51505557	0.652703644	0.906199123
0.22250351	0.676283472	0.698387673	0.479200062	0.558411284	0.750539549
0.234587818	0.992748375	0.853876899	0.496374188	0.978334688	1.003054503
0.39166714	0.708333166	0.603531092	0.449315271	0.886566036	0.942065973
0.3075302	0.579467639	0.66062341	0.30919738	0.580392186	0.786162798
0.416416748	0.819718237	0.581963267	0.52576864	1.138788635	1.068435635
0.60638249	0.774694821	0.652567933	0.453476596	0.839207805	0.787089719
0.221672237	0.842763653	0.618094786	0.376337772	0.918212514	1.032326978
0.457771642	0.83497216	0.716480825	0.46339038	0.825591487	0.792289726
0.40351662	0.858089479	0.523586571	0.666141238	0.748928554	0.682310347
0.186946841	1.099311313	0.700375246	0.381723524	1.008282564	0.976031761
0.444421341	0.592053724	0.941543725	0.387911647	0.739642485	0.656879205
0.360707169	0.704660378	0.824219213	0.506593334	0.867057755	0.710546022
0.281518181	0.628855302	0.600526577	0.353774017	0.596874697	0.699114179
0.53119004	0.696888619	0.672357295	0.373065279	0.975355462	0.484745698
0.198086161	0.746596163	0.778732639	0.436332548	1.058337668	0.755917136

P11215,ITGAM,NEUROLOGY	O14944,EREG,NEUROLOGY	Q9Y680,FKBP7,NEUROLOGY	P16444,DPEP1,NEUROLOGY	Q86WV1,SKAP1,NEUROLOGY	Q02083,NAAA,NEUROLOGY
P11215	O14944	Q9Y680	P16444	Q86WV1	Q02083
ITGAM	EREG	FKBP7	DPEP1	SKAP1	NAAA
NEUROLOGY	NEUROLOGY	NEUROLOGY	NEUROLOGY	NEUROLOGY	NEUROLOGY
1.22901285	0.408554373	1.226119845	0.295493848	0.86934456	0.438242103
0.851099672	0.406915178	1.857291162	0.344052349	0.870067961	0.397961287
1.022782939	0.485418164	0.737850276	0.447016503	0.608909615	0.459838766
0.912881814	0.465999399	1.299809322	0.296972243	0.711087992	0.389015623
0.921336453	0.50736644	1.195654032	0.466064005	0.857554342	0.574030779
0.908778116	0.599279114	1.231656538	0.299577255	0.492330833	0.34238704
0.832660332	0.541074817	0.996540263	0.368669507	0.656606074	0.291506508
0.726633608	0.418442125	1.215289478	0.262702166	0.499515032	0.335247853
0.81524184	0.375842469	0.994608041	0.343742466	0.571688035	0.303801311
0.677597285	0.414889773	1.041887855	0.296437527	0.801958785	0.239782787
0.766044443	0.472471758	0.988354157	0.333440211	0.585523914	0.902000414
1.074376758	0.594850898	1.505351087	0.287931992	1.046375016	0.398955572
0.66342257	0.408554373	1.700076893	0.258135417	0.575225684	0.265024934
0.76785191	0.362486708	1.386262049	0.372806779	0.428123817	0.361082398
0.724119647	0.365058607	1.157891442	0.286379474	0.34745547	0.479166848
0.691739143	0.384192164	1.420402692	0.301849225	0.760225857	0.283103208
1.112264572	0.494108576	1.477338064	0.426849679	1.144168872	0.343123537
1.243063503	0.432958305	0.923510328	0.375217755	0.636176947	0.495240086
1.166834542	0.42717526	1.104428483	0.289232184	0.882335967	0.308897479
0.778678663	0.341628443	1.786703201	0.179281683	0.828859801	0.295432408
0.958665886	0.314667482	0.776684188	0.31281899	0.53998828	0.329009238
0.841012996	0.526680518	1.069547088	0.224984887	0.663146718	0.270724996
0.880259014	0.35030901	1.113730367	0.116507925	0.832083375	0.259013644
0.654289036	0.344028502	1.299268859	0.271495464	0.399342909	0.32302103
0.856663188	0.453696678	1.243752995	0.414803508	0.854942916	0.281635285
0.91783072	0.395404172	1.360465149	0.424960307	0.756651039	0.341178822
0.770090551	0.474177792	2.797038283	0.459010799	0.611023596	0.295944797
0.693275172	0.418877414	1.195074038	0.277027288	0.803962436	0.49120597
0.903251719	0.717325588	1.520451714	0.22020208	0.457295935	0.328963631
0.841479482	0.551372798	1.013186669	0.534736478	0.452001667	0.37845666
0.834104473	0.389312346	0.720365004	0.379007946	0.374594079	0.394200087
0.888165231	0.399398273	1.587437729	0.283122831	0.532443372	0.305978036
0.76154437	0.400534939	0.916241618	0.332125408	0.498132001	0.267757706
0.772817678	0.43603021	1.257797785	0.210486557	0.465289327	0.29641698
0.69495911	0.487711535	1.291815468	0.281889178	0.93212801	0.632746707

P53582,METAP1,NEUROLOGY	Q9HAW4,CLSPN,NEUROLOGY	P05067,APP,NEUROLOGY	O15389,SIGLEC5,NEUROLOGY	P11717,IGF2R,NEUROLOGY	P37023,ACVRL1,NEUROLOGY
P53582	Q9HAW4	P05067	O15389	P11717	P37023
METAP1	CLSPN	APP	SIGLEC5	IGF2R	ACVRL1
NEUROLOGY	NEUROLOGY	NEUROLOGY	NEUROLOGY	NEUROLOGY	NEUROLOGY
1.066659713	0.932451117	0.597164372	0.340116287	0.428569177	0.588738957
1.048771219	0.976370086	0.630426463	0.624424911	0.412281034	0.546805629
1.085154152	0.784529717	0.655832817	0.571173123	0.47430928	0.603572927
1.22577994	1.373255433	0.616041736	0.562568235	0.389771362	0.496718367
1.155005723	1.270855499	0.703684188	0.381749984	0.436211588	0.647027977
0.90000193	0.888842681	0.638252859	0.258242794	0.359210145	0.793278855
1.083350441	1.146312186	0.597081594	0.024974507	0.391477148	0.466581173
0.995918783	1.255271991	0.546048121	0.291728856	0.317890102	0.411995362
1.258320997	1.372969903	0.578985852	0.516987033	0.321524371	0.477674577
1.520662507	1.155245925	0.666233591	0.523441422	0.256796955	0.286657514
1.207564291	1.120855268	0.672310693	0.150194095	0.35774413	0.38788476
1.178376039	1.285473616	0.777114992	0.530270355	0.379218171	0.692794797
1.147345583	0.957603281	0.513238037	0.309047393	0.369078601	0.459106257
1.174217774	0.824676384	0.693467415	0.267090395	0.33457464	0.480131002
1.143217574	0.949210523	0.604619746	0.022901724	0.319325573	0.359160351
1.059291757	1.113730367	0.625811467	0.177279792	0.333648287	0.438211728
1.092701996	1.049934989	1.890935299	0.629727685	0.364957405	0.361207562
1.014310952	1.204888796	0.712420038	0.41529258	0.552788689	0.570935629
0.954356374	1.214110726	0.563856519	0.476649273	0.435728082	0.534106742
1.267248978	1.17609125	0.730978055	0.411139531	0.346109393	0.449751501
1.065477406	1.190279182	0.52850902	0.16130913	0.46274843	0.426613049
0.983093132	1.074376758	0.745561878	0.247757391	0.389609295	0.49267221
1.209323312	0.913451477	0.56097174	0.188077608	0.385525983	0.381670609
1.437039781	0.992748375	0.595882589	0.326510208	0.33401852	0.425962992
1.397259516	1.559301599	0.577863239	0.361407913	0.381459025	0.60924736
1.351723431	1.806879128	0.758909626	0.519717664	0.445562586	0.455776991
1.197395705	1.059365184	0.576782781	0.636750459	0.381670609	0.352183689
0.817505306	0.968685331	1.021082899	0.326555475	0.365514362	0.492057903
1.138630776	1.334870696	0.61288997	0.023436485	0.38291604	0.477773917
1.275444392	1.305045403	0.531042783	0.393790442	0.382544636	0.579226695
0.901063072	0.932451117	0.694140687	0.210574114	0.247191322	0.373168729
0.788782801	0.976099416	0.625161136	0.230621672	0.364527611	0.4499074
1.160301715	1.030682513	0.745096918	0.348516774	0.295309567	0.412738522
1.189289547	1.418140036	0.585767477	0.356086587	0.341960122	0.514841409
1.175765214	1.014029765	0.618223328	0.196540699	0.430295602	0.447295454

Q6UXG3,CD300LG,NEUROLOGY	Q9Y6Y9,LY96,NEUROLOGY	Q99731,CCL19,NEUROLOGY	Q96CD2,PPCDC,NEUROLOGY	P09769,FGR,NEUROLOGY	P02749,APOH,NEUROLOGY
Q6UXG3	Q9Y6Y9	Q99731	Q96CD2	P09769	P02749
CD300LG	LY96	CCL19	PPCDC	FGR	APOH
NEUROLOGY	NEUROLOGY	NEUROLOGY	NEUROLOGY	NEUROLOGY	NEUROLOGY
0.665356752	0.494862628	0.156963389	0.66646453	0.69380397	0.89260884
0.74649267	0.466742906	0.304096265	0.866396908	0.945533186	0.946451183
0.774533744	0.543141498	0.559806444	0.688247801	0.809722796	1.121943481
0.73907875	0.447543556	0.311110717	0.626592758	0.961260928	0.90645041
0.79901805	0.638164384	0.568526696	0.72895416	1.299539062	0.621058038
0.640735131	0.526461523	0.218453784	0.632527452	0.637633795	0.88976731
0.623732786	0.520763414	0.254246532	0.659708226	0.923510328	0.885644734
0.600526577	0.578905593	0.323940331	0.504036459	0.727439918	0.955349154
0.470673978	0.488354264	0.402204185	0.455114041	0.876727253	0.848566718
0.528179422	0.446428179	3.171796034	0.462684284	0.834624977	0.981867326
0.577502861	0.418935487	0.174814893	0.587882607	0.900064315	0.944026982
0.985276123	0.547071005	0.394145443	1.093004999	0.710595275	0.974882331
0.499272725	0.452911162	0.193700556	0.545896745	0.873028103	0.824333482
0.506347593	0.431819411	0.636529817	0.47355372	0.823476851	0.684441907
0.514021283	0.479598815	0.152597158	0.574627921	0.673850287	0.829549514
0.439123911	0.558992178	0.331665303	0.530858769	0.960861234	0.762970933
0.596585162	0.436937853	0.310335361	0.931675848	0.939392513	0.819207029
0.718768954	0.537411836	0.312992502	0.803349679	0.803628147	1.011152081
0.628898892	0.489778042	0.312472255	0.85423209	0.887119277	0.921655819
0.470771863	0.502989434	0.369334516	0.470347845	1.029254674	0.851453708
0.69011084	0.456472546	0.256868164	0.647656162	0.895583591	0.819831882
0.479200062	0.478668907	0.205128521	0.77335354	0.752519045	0.811070934
0.522643822	0.419778447	0.229505399	0.609838864	0.955547834	0.745045273
0.602862125	0.459743155	0.327348665	0.374879801	0.898256889	0.848390282
0.669706094	0.499584285	0.164801354	0.435124453	0.927359097	0.893042042
0.784312229	0.502884851	0.630295383	0.528325885	1.043911927	0.976167077
0.60617237	0.488388116	0.175312406	0.59106963	0.739283695	0.928195109
0.63980315	0.530307111	0.274332984	0.745096918	0.917767103	0.8456309
0.704709223	0.418587171	0.338610825	0.829204586	0.909471289	0.897883392
0.718171347	0.742004582	0.218317548	0.42445985	0.940956543	0.919167696
0.483001642	0.424930852	0.236514412	0.398596238	0.449097314	0.734737085
0.601568112	0.331642315	0.221257766	0.429133966	0.54699517	0.863998073
0.532443372	0.438819639	0.222118275	0.404160434	0.723216752	0.719666296
0.732905959	0.440953979	0.145531161	0.463005103	0.845806762	1.023563069
0.551487465	0.454231606	0.210296975	0.745148566	1.316675654	0.940043876

Q9H446,RWDD1,NEUROLOGY	Q6ZMJ4,IL34,NEUROLOGY	O43278,SPINT1,NEUROLOGY	O14579,COPE,NEUROLOGY	Q02487,DSC2,NEUROLOGY	O76061,STC2,NEUROLOGY
Q9H446	Q6ZMJ4	O43278	O14579	Q02487	O76061
RWDD1	IL34	SPINT1	COPE	DSC2	STC2
NEUROLOGY	NEUROLOGY	NEUROLOGY	NEUROLOGY	NEUROLOGY	NEUROLOGY
1.092247649	1.150052722	0.544423029	0.844283837	0.352232516	0.34231585
1.091188239	1.564172907	0.462716356	0.739796305	0.405170207	0.404973665
0.810059621	1.150850154	0.643405423	0.698339266	0.545934585	0.326057882
0.894714933	1.604918459	0.456947397	0.832141053	0.315541136	0.330838721
0.774265358	1.014803218	0.514128182	0.926331193	0.301891073	0.371955001
0.841712822	1.132255855	0.540512542	1.131314463	0.392428031	0.401897637
0.999376362	0.94579538	0.570579571	0.984934711	0.425638336	0.389636301
0.619596114	1.056358844	0.53950192	0.606929142	0.510895453	0.377068882
0.832718049	1.490297131	0.376155216	0.637015331	0.236793273	0.290316807
0.851453708	0.814338209	0.388315177	1.055041684	0.248118291	0.270687468
0.716629829	1.684476148	0.333370881	1.069250588	0.253349344	0.291425697
1.551861709	1.323263158	0.689298127	0.690637224	0.522498934	0.504036459
0.711679705	1.072516617	0.491955593	0.96520012	0.264364433	0.296458075
0.77362161	1.230632501	0.360457232	0.713161141	0.247688707	0.23903603
0.939587874	1.011152081	0.258081744	0.678678399	0.170022785	0.238242054
0.935818075	1.389629225	0.360882227	0.966271155	0.305914416	0.22326051
1.755400228	0.935234464	0.517237937	0.766522476	0.258135417	0.423079298
0.815354864	1.244874235	0.559767643	0.704269739	0.407875286	0.418442125
0.93400359	1.410982407	0.497821347	1.09992107	0.314275127	0.392972429
0.650580732	2.097251677	0.3898254	1.54735047	0.351744556	0.298437348
0.503582481	1.546599873	0.379191886	0.861904541	0.311952873	0.358265245
0.817788681	1.461145654	0.393272169	0.80770479	0.319724233	0.360057694
0.790205613	1.513826639	0.423167284	0.703001659	0.325200189	0.311520714
0.641312752	1.4063932	0.453068156	1.232339703	0.274104896	0.2997642
0.55953489	1.391074802	0.50396659	0.671472397	0.408951029	0.418674222
1.137920683	0.895769842	0.46432279	0.950461438	0.357199013	0.328416838
0.912881814	1.333483524	0.389447294	0.881113637	0.421615555	0.362788342
1.765895535	1.041093758	0.380271046	0.680657058	0.263413283	0.364426556
1.173078859	1.531133418	0.490083677	0.866276809	0.376259523	0.393735855
0.721264339	1.148300315	0.3739196	0.94842132	0.267201498	0.316088401
0.743806881	1.056358844	0.370334279	0.524858342	0.269302588	0.281167159
0.84188787	1.747630271	0.524240239	0.647027977	0.3103999	0.380772185
0.662687219	0.852103156	0.496202187	0.5498843	0.291931137	0.431669779
0.759646434	0.748098426	0.502675751	1.329975805	0.317581769	0.311024471
1.701727459	1.301341855	0.480563839	1.24099732	0.279108893	0.262847879

Q6XZF7,DNMBP,NEUROLOGY	Q9BYC5,FUT8,NEUROLOGY	Q9HD42,CHMP1A,NEUROLOGY	Q9HCK4,ROBO2,NEUROLOGY	P17405,SMPD1,NEUROLOGY	Q5T2D2,TREML2,NEUROLOGY
Q6XZF7	Q9BYC5	Q9HD42	Q9HCK4	P17405	Q5T2D2
DNMBP	FUT8	CHMP1A	ROBO2	SMPD1	TREML2
NEUROLOGY	NEUROLOGY	NEUROLOGY	NEUROLOGY	NEUROLOGY	NEUROLOGY
0.43277828	0.70305039	1.581068638	0.918085232	0.298333936	0.637501216
0.445284716	0.446211623	1.124278924	1.041599023	0.558953433	0.824905065
0.442055679	0.670124008	0.897136864	0.853995279	0.41838412	1.022712047
0.395404172	0.451719782	1.218241368	1.330067995	0.611277767	0.575584641
0.493458273	0.580110646	1.8957911	0.848978545	0.535812447	0.541675221
0.611023596	0.514841409	1.350599567	0.725677276	0.240481864	0.628724549
0.389987557	0.466872332	1.225270258	0.622523412	0.29717816	0.866877474
0.363392362	0.391938717	1.022215945	0.790150842	0.380060237	1.065920617
0.238638712	0.39978604	0.895769842	0.687580245	0.340399306	0.462203471
0.286717129	0.444236549	1.250062303	0.627853555	0.258314404	0.472766593
0.352232516	0.414372452	1.151648139	0.738413073	0.531153222	0.529645878
0.890939887	0.67581487	1.393390855	1.002290004	0.358215582	0.815467904
0.212863427	0.534921835	0.968953945	0.819207029	0.23306431	0.583215109
0.320034646	0.273478628	1.135793059	0.62994597	0.308298548	0.395568651
0.150152458	0.198374707	1.126150778	0.608066072	0.390420307	0.372393553
0.358190753	0.316987971	1.331451613	0.431969094	0.269414611	0.455998189
0.353088074	2.050392348	1.20405392	0.761227718	0.340895155	0.68037404
0.362612358	0.362562093	1.200886664	0.78561806	0.387025358	0.808713164
0.334969118	0.44537732	0.932257239	1.142425431	0.65170908	0.338001136
0.328416838	0.512562558	1.089298991	0.702173765	0.242658617	0.533477748
0.353063601	0.279767445	1.25989194	0.660302952	0.305215472	0.647521499
0.454074209	0.435667681	0.970432653	0.566873998	0.238853844	0.536518566
0.325245274	0.360307353	0.975355462	0.839789701	0.176568513	0.537896311
0.253525014	0.256245753	1.241943894	0.429520829	0.264456071	0.432958305
0.331734278	0.412795744	0.849096246	0.714398026	0.21851436	0.753824192
0.270856384	0.587801115	1.258582685	0.586254908	0.291324714	0.710496772
0.255288419	0.312797308	1.25292495	0.590946733	0.318242849	0.71015212
0.303191245	0.58743454	1.259804614	0.661310631	0.308576478	0.566402681
0.249290533	0.708431369	1.23841942	0.728550054	0.408101523	0.539352359
0.386864432	0.307317111	1.282003322	0.492945481	0.351086883	0.558875951
0.408214689	0.480430617	1.103510227	0.551334582	0.254563944	0.514449012
0.311132283	0.514021283	1.222894545	0.907644973	0.325786787	0.425874425
0.275094652	0.472701058	0.866757308	0.482767345	0.22768324	0.315300639
0.246216611	0.387079015	1.315945736	0.559341004	0.328553451	0.478337234
0.312039376	0.504350992	1.05270412	0.791521257	0.574827106	0.701833151

O15232,MATN3,NEUROLOGY	P63098,PPP3R1,NEUROLOGY	Q9HAN9,NMNAT1,NEUROLOGY	Q96IU4,ABHD14B,NEUROLOGY	O15496,PLA2G10,NEUROLOGY	P14207,FOLR2,NEUROLOGY
O15232	P63098	Q9HAN9	Q96IU4	O15496	P14207
MATN3	PPP3R1	NMNAT1	ABHD14B	PLA2G10	FOLR2
NEUROLOGY	NEUROLOGY	NEUROLOGY	NEUROLOGY	NEUROLOGY	NEUROLOGY
11.60279119	0.543555781	0.979420298	0.634899435	0.856722569	0.316219886
8.305114164	0.582245706	0.655469247	0.659388212	0.737236804	0.545405064
12.75154366	0.809105649	0.884050078	0.735858358	1.0181852	0.309368883
7.319778538	0.695200006	0.836652248	0.637677994	0.793993994	0.60202696
8.108865033	1.095280204	1.267951885	1.151887642	0.636927028	0.388207528
6.879959155	0.701687224	0.923382311	0.679619899	1.568624458	0.360207469
6.274497831	0.56616717	0.685961732	0.66287098	0.359110564	0.294206291
7.598949552	0.711729036	0.625985003	0.420273384	0.322886718	0.344577403
6.525552248	0.573474006	0.589800929	0.578905593	0.282201977	0.265245467
3.963019318	0.429639934	0.983502074	0.582326428	0.568605516	0.217125338
7.713576823	0.658109704	0.774211692	0.395321959	0.888965909	0.280894445
15.83121415	0.785128119	1.318959257	0.899440654	0.772282187	0.440587357
7.639085824	0.675159373	0.686151948	0.315563008	0.681884833	0.389879445
5.487313493	0.592176851	0.802459229	0.521594297	0.338376199	0.255005452
5.683186264	0.516127712	0.592300004	0.578664883	0.402204185	0.190478491
2.988662526	0.526972653	1.315398564	0.637147809	0.51652139	0.25774208
6.602443268	0.657836061	0.772817678	0.764029368	0.708824315	0.41359768
7.40602798	0.814056029	1.126619228	0.683920245	0.864057963	0.262483747
7.39218058	0.794654695	1.106497353	0.499515032	1.25292495	0.518817842
8.707750071	0.560544184	0.795536484	0.449471018	0.5253315	0.363442742
7.839165045	0.696309205	0.761174956	0.438850057	0.543254453	0.260923719
7.919997638	0.614804658	0.798021765	0.578464368	0.757228177	0.414889773
9.5672215	0.670588664	0.622652875	0.516987033	1.315033908	0.243298613
4.919801131	0.654016981	0.787144277	0.421294212	0.742724977	0.228394529
11.1178044	0.789165614	0.659891162	0.417572903	0.715091619	0.406858772
7.165662029	0.586824089	0.556170836	0.833006697	0.380956982	0.254528657
2.46160623	0.427293714	0.88154126	0.691307748	0.470739232	0.43419048
10.63169708	0.561438538	0.973734255	0.591971654	0.418964526	0.23702317
8.266060941	0.53950192	0.732601216	0.675159373	0.390501501	0.378876615
6.018483452	0.688772764	0.779002574	1.289489477	0.648150163	0.302960161
1.154045416	0.512384948	0.697226834	0.545480678	0.2946757	0.277046491
9.7967281	0.530601258	0.591233532	0.534365955	0.532074438	0.484745698
4.902779913	0.441382091	0.680326881	0.43470241	0.347310998	0.279321785
6.098692601	0.586580087	1.015999717	0.928130773	0.52355028	0.317339717
7.908477604	0.421060661	1.108954367	0.707646129	0.664158738	0.380745793

Q8WV92,MITD1,NEUROLOGY	Q07812,BAX,NEUROLOGY	Q9UBB4,ATXN10,NEUROLOGY	P08962,CD63,NEUROLOGY	P50225,SULT1A1,NEUROLOGY	P10636,MAPT,NEUROLOGY
Q8WV92	Q07812	Q9UBB4	P08962	P50225	P10636
MITD1	BAX	ATXN10	CD63	SULT1A1	MAPT
NEUROLOGY	NEUROLOGY	NEUROLOGY	NEUROLOGY	NEUROLOGY	NEUROLOGY
0.756546152	0.245943699	0.116120935	0.288731417	0.68448935	1.045939932
0.658337827	0.476385035	0.249670972	0.263961605	0.581479405	1.216469375
0.689776078	0.195752142	0.195942193	0.276087983	0.618866442	0.983638426
0.589678296	0.844459419	0.155987248	0.293330705	0.859041658	0.653110949
1.027401439	0.576103529	0.198512258	0.536890581	0.665310635	1.313576297
0.636397468	0.499618914	0.162374839	0.22332242	0.587149584	1.378787381
0.960262005	0.563895604	0.140398556	0.262847879	0.479200062	1.194577124
0.625507895	0.716182911	0.117978837	0.143527591	0.52373176	1.585788095
0.840197268	0.66701911	0.092924318	0.192976891	0.613144917	0.850981692
0.756493714	0.389042588	0.106956792	0.148434669	0.632834431	1.051610171
0.514056913	0.18780404	0.152702967	0.177378124	0.453476596	1.238591113
0.704416203	0.77938064	0.152544281	0.576183399	0.745303531	0.455429611
0.512136397	0.951845945	0.137833645	0.184500931	0.598490395	1.048335138
0.512917962	0.33106812	0.132687098	0.121970148	0.320079015	1.160623463
0.495755265	0.485317235	0.13061568	0.10820957	0.501040803	0.653292055
0.69025436	0.443867197	0.16868458	0.215446266	0.378745329	1.134455485
0.893661266	0.866396908	0.186455079	1.083350441	0.72638182	1.516557291
0.779921053	0.379849545	0.108322136	0.210778555	0.674598025	1.526259209
0.722164797	0.772335719	0.119269743	0.239085742	0.890322549	1.281292625
0.504281079	0.651347796	0.079975394	0.111467883	0.438910899	1.206727561
0.564403954	0.594685993	0.16700926	0.148661193	0.472864912	2.048829598
0.816655771	0.853344389	0.153233113	0.25605045	0.499065126	1.316310644
0.555361857	0.431580025	0.156117049	0.250364167	0.463583139	1.077957277
0.458978984	0.468006347	0.210763946	0.172444019	0.337884014	1.084251922
0.520763414	0.371336748	0.119286278	0.188090645	0.494965543	1.10297493
0.547412392	0.576822762	0.097213467	0.330265917	0.357768928	1.59416394
0.526936127	0.63564801	0.183315413	0.142013501	0.559224704	1.12537046
0.730420924	0.77313915	0.120842513	0.255979468	0.768863821	0.946385582
0.558333877	0.563778357	0.212553806	0.150298238	0.549617559	1.65484948
0.577863239	0.537188379	0.063804412	0.215565768	0.747683707	1.489883991
0.398430501	0.19356634	0.038313589	0.106085545	0.583255536	1.145676711
0.547716026	0.229999081	0.121826509	0.140944589	0.420477352	0.8005703
0.698968817	0.398762044	0.043248683	0.167833196	0.411253539	1.090961356
0.49120597	0.625117805	0.139554447	0.09113822	0.61920971	1.081774654
0.800958833	0.76530143	0.06279093	0.240999156	0.60924736	1.090507733

Q9Y6D9,MAD1L1,NEUROLOGY	Q92752,TNR,NEUROLOGY	O95256,IL18RAP,NEUROLOGY	Q8NBI3,DRAXIN,NEUROLOGY	Q9BW30,TPPP3,NEUROLOGY	O14763,TNFRSF10B,NEUROLOGY
Q9Y6D9	Q92752	O95256	Q8NBI3	Q9BW30	O14763
MAD1L1	TNR	IL18RAP	DRAXIN	TPPP3	TNFRSF10B
NEUROLOGY	NEUROLOGY	NEUROLOGY	NEUROLOGY	NEUROLOGY	NEUROLOGY
0.443129415	0.394719583	0.433498829	1.565148993	0.721014411	0.652206173
0.5253315	0.724370651	0.319724233	1.946523821	0.733973561	0.540325247
0.581157054	0.777007269	0.341723176	2.484748787	1.031611671	0.602945705
0.343171108	0.631607409	0.431131536	1.267161142	0.94842132	0.437483345
0.479266498	0.583619503	0.564638732	1.147584192	1.491950841	0.645504921
0.547412392	0.527996401	0.319192797	1.236532364	0.936596791	0.804352616
0.478735269	0.524131238	0.573990992	1.4037636	0.804129633	0.56327057
0.358687656	0.436060434	0.398983226	1.158613998	0.84621725	0.51644979
0.33143549	0.751424468	0.278336111	0.919550046	0.792509426	0.377906176
0.3441239	0.4399465	0.256868164	1.04015606	1.060687741	0.335852576
0.346709675	0.267201498	26.78400876	0.818412452	0.787799278	0.404384611
0.592587459	0.589473965	0.963929808	2.01279466	1.491433859	0.848390282
0.384112282	0.430683512	0.149622599	1.336815161	0.643494624	0.436847004
0.338986565	0.354387593	0.373091139	0.798298386	0.898007874	0.383925955
0.305617699	0.343147322	0.189241447	0.535404066	0.801125405	0.353994782
0.436574569	0.448879464	0.234799298	0.610896551	1.115275396	0.320745293
0.400146446	0.415004821	0.26528224	1.265932077	0.854468966	0.443129415
0.457042427	0.641623995	9.954162921	1.480515916	0.736317553	0.395321959
0.586620747	0.557985679	0.759225314	2.295486584	1.070808132	0.556170836
0.474112061	0.378168211	0.481597564	1.312757101	1.061276075	0.557637698
0.385927031	0.512384948	0.299473448	1.2894001	0.548133798	0.400507177
0.514841409	0.484510555	0.245892562	1.032183877	0.757543164	0.518063196
0.387079015	0.329511335	0.569197015	1.069769517	0.842997349	0.444482955
0.33980995	0.429639934	0.367725212	0.788017733	0.785400271	0.36296441
0.515698588	0.799904681	1.191434797	2.06179372	0.895459445	0.499376557
0.385232146	0.683114822	0.180967182	0.787144277	1.346486719	0.483135577
0.362536963	0.72956074	0.307168036	1.029896957	0.921591937	0.346349381
0.437301439	0.420943935	1.934686757	1.008701984	0.672636979	0.351013884
0.439824538	0.752466886	0.550036781	0.820627838	0.713358899	0.482968164
0.577102706	0.452534598	0.968752478	0.735399449	0.876788025	0.527886618
0.39112455	0.35732283	0.819320604	0.580915408	0.960395135	0.402762146
0.451939011	0.745096918	0.41425758	1.093383872	0.664895722	0.544876056
0.461979263	0.340257767	0.215282059	0.738413073	0.606130355	0.407028015
0.386971709	0.397685537	0.328052814	0.712370658	1.088468759	0.431909214
0.417283563	0.488218883	0.927551956	1.115971358	0.77067794	0.352037251

Q06830,PRDX1,NEUROLOGY	P48047,ATP5PO,NEUROLOGY	Q96JP9,CDHR1,NEUROLOGY	Q8WTV0,SCARB1,NEUROLOGY	P36269,GGT5,NEUROLOGY	P45452,MMP13,NEUROLOGY
Q06830	P48047	Q96JP9	Q8WTV0	P36269	P45452
PRDX1	ATP5PO	CDHR1	SCARB1	GGT5	MMP13
NEUROLOGY	NEUROLOGY	NEUROLOGY	NEUROLOGY	NEUROLOGY	NEUROLOGY
1.170560904	1.315489743	0.387750353	0.41116803	0.660257184	5.301130293
0.786871521	1.373921903	0.651302649	0.685914187	0.422903381	7.839708433
1.03332924	0.977453516	0.436302305	0.70207643	0.779272602	10.44832664
0.837870973	0.693034943	0.522607596	0.486394898	0.560699621	4.01333059
1.043550198	0.845279285	0.415811049	0.464934697	0.825305409	5.161176614
1.128573203	0.679949733	0.488388116	0.308662046	0.613867842	5.989767483
1.361408479	1.316858197	0.573792096	0.747994724	0.464676954	7.93318394
0.625681347	0.998614666	0.583215109	0.438363627	0.400673778	11.01730915
0.872241779	1.393970471	0.520871715	0.632834431	0.361508131	3.748725011
0.931934199	0.830297351	0.461467196	0.481864693	0.338822127	3.740678562
0.776845711	1.086282996	0.393735855	0.585442749	0.424960307	4.143069438
1.294055954	0.78051594	0.773514371	0.439215234	0.657608112	11.82689945
0.713507253	1.352566943	0.454578073	0.619553168	0.422083401	7.833190258
0.730471555	1.132962414	0.689393691	0.482432833	0.306870102	6.101652423
0.712469421	1.22010051	0.397354889	0.624468195	0.355100673	6.825335672
1.527846918	1.232425125	0.428094142	0.513309192	0.313122699	4.033130255
2.354466181	0.853166959	0.490831587	0.500901904	0.965936329	4.166396091
1.123577781	1.18624335	0.593738677	0.325200189	0.52898547	5.826380816
0.745148566	0.873512347	0.487137179	1.201469479	0.646042061	4.893612953
1.038499125	1.006327574	0.680751424	0.737236804	0.533736656	7.597896187
0.660577621	1.067843333	0.385098658	0.380350129	0.286876163	6.887116112
0.874421028	1.098625741	0.456599125	0.657562531	0.278066143	7.830475952
0.700860878	1.308033962	0.722415123	0.589841812	0.295883264	9.983875889
0.736164456	1.429390396	0.67717471	0.400118711	0.45429458	4.765078562
0.912249272	1.145994405	0.458978984	0.685201398	0.493971599	5.58671413
1.094521277	1.045432562	0.772068094	0.806865439	0.455334917	4.557997079
0.976505449	0.873209663	0.457454449	0.641979885	0.343432862	3.822191335
1.350693187	0.966740106	0.504106339	0.361633441	0.313600551	5.543126762
1.362635785	0.970432653	0.795536484	0.595799988	0.325922306	6.857582151
0.995780728	0.854528195	0.518817842	0.549960535	0.597495602	4.266934605
0.802848679	0.887488297	0.400590469	0.594315124	0.453696678	2.067661472
0.838277609	0.733007569	0.575624539	0.477376681	0.41065535	8.379127343
0.816938851	0.94710744	0.643316234	3.476655215	0.362888942	5.167620052
1.29486348	1.46286841	0.525695759	0.539913427	0.429848447	5.109209168
1.19325303	0.792729187	0.810508938	0.408554373	0.411766966	5.146173152

P12644,BMP4,NEUROLOGY	P23276,KEL,NEUROLOGY	Q02747,GUCA2A,NEUROLOGY	Q02643,GHRHR,NEUROLOGY	P30043,BLVRB,NEUROLOGY	P01215,CGA,NEUROLOGY
P12644	P23276	Q02747	Q02643	P30043	P01215
BMP4	KEL	GUCA2A	GHRHR	BLVRB	CGA
NEUROLOGY	NEUROLOGY	NEUROLOGY	NEUROLOGY	NEUROLOGY	NEUROLOGY
0.381882311	0.537113914	0.35128162	0.646758942	1.392521884	0.118289998
0.39526716	0.614676826	0.315191383	0.799904681	1.099463721	0.595882589
0.641268301	0.74298243	0.39052857	0.905257419	1.612277414	0.312537238
0.523622864	0.504350992	0.438059881	0.949605371	0.878795873	0.165798171
0.448723921	1.034045738	0.333555792	1.053142018	0.797302999	0.282045534
1.185174918	0.413311096	0.397382432	0.813491964	1.155245925	0.311628697
0.527045712	0.507155476	0.380350129	1.455585919	1.279783703	0.330999284
0.538306593	0.805859369	0.273839031	1.393101138	0.880564141	0.26624014
0.226047834	0.479332943	0.212730677	1.320331319	1.031182725	0.236235879
0.318728515	0.725224717	0.2059263	1.225864907	1.309031669	0.285784584
0.547222707	0.67245051	0.395212368	1.139104418	0.918467132	0.19372741
0.821367632	0.671332783	0.573195822	1.003471749	1.48596486	0.321279314
0.811689581	0.364527611	0.197729495	1.271119794	0.986984956	0.204475515
0.544121221	0.921719706	0.195223685	1.237475532	1.1076484	0.303990891
0.251233369	0.530380633	0.182112266	1.245133127	0.979013053	0.236809687
0.265797603	0.776522698	0.217894245	1.264441244	1.418533282	0.227100061
0.573394511	0.695682049	0.261557492	0.574309368	2.064653955	0.186442155
0.619166791	0.621316382	0.288491357	1.486376915	1.628902215	0.315300639
0.552827006	0.63366841	0.277969789	1.304683618	1.069621226	0.26110464
0.754085493	0.588331016	0.33214843	1.460740594	0.85731661	0.466193243
0.283653191	0.467034166	0.238903517	1.149574528	0.916559218	0.129166155
0.359259945	0.52635206	0.362461583	1.036054575	1.500975054	0.243214307
0.554707832	0.50547092	0.42581539	1.542531552	0.87151657	0.207373004
0.201912201	0.766894485	0.239799408	1.052923046	1.0453601	0.234669134
0.530748392	0.585239885	0.30945467	1.106497353	1.271207904	0.240498533
0.46648416	1.001179045	0.234896969	1.577128269	1.56884193	0.248496941
0.34191272	0.625204471	0.28973382	1.16764361	1.387704124	0.522317881
0.386408839	0.574707587	0.216719369	1.102363481	2.178748997	0.205883483
0.583336398	0.783225695	0.27912824	1.462767015	1.490917057	0.211217313
0.334412342	0.472013491	0.239749548	1.157730935	0.931223906	0.27675859
0.254458096	0.488354264	0.288751431	0.817391984	1.268215575	0.334412342
0.671053642	0.664619257	0.280563648	0.865376591	1.120000984	0.241116118
0.411082539	0.416070527	0.206426487	0.835377389	1.089827651	0.25605045
0.419400359	0.563778357	0.254193668	1.044635763	1.157891442	0.294532757
0.256885969	0.529352262	0.40287383	1.221115784	2.113157061	0.146655187

O00220,TNFRSF10A,NEUROLOGY	O43155,FLRT2,NEUROLOGY	P50453,SERPINB9,NEUROLOGY	P22105,TNXB,NEUROLOGY	P06748,NPM1,NEUROLOGY	O95407,TNFRSF6B,NEUROLOGY
O00220	O43155	P50453	P22105	P06748	O95407
TNFRSF10A	FLRT2	SERPINB9	TNXB	NPM1	TNFRSF6B
NEUROLOGY	NEUROLOGY	NEUROLOGY	NEUROLOGY	NEUROLOGY	NEUROLOGY
0.51416382	0.439824538	0.843464936	0.769823704	1.263214818	0.298996396
0.475263661	0.619424349	0.699162639	0.723617899	1.525307376	0.654651951
0.453633786	0.816995479	0.918085232	0.849979529	1.782250345	0.404440674
0.369872514	0.764453154	0.760331254	0.683541104	1.682025993	0.347841025
0.513487122	0.534217818	0.969491396	0.658429098	1.871506424	0.343099755
0.554746283	0.715240334	0.889643971	0.790479525	1.476212079	0.450781433
0.491240019	0.586905446	0.791795624	0.512029913	1.166106859	0.445871534
0.460795969	0.572997202	0.53733734	0.700909459	0.821139932	0.33401852
0.325538482	0.318066426	0.609120684	0.595469698	1.107111096	0.234978392
0.305787216	0.444914493	0.701930452	0.511072546	1.477440468	0.289914621
0.444575392	0.59563482	0.526206144	0.711679705	0.954025676	0.177206079
0.51181701	0.802125565	1.21326946	1.150371628	2.435469642	0.585158759
0.32483973	0.641846403	0.605962323	0.717077025	1.085003728	0.1971957
0.247860452	0.352403461	0.62455477	0.666834199	1.056578531	0.19030693
0.278722234	0.371955001	0.527776859	0.431879278	0.657334677	0.172109663
0.311067591	0.364881522	0.671472397	0.347985718	2.905930099	0.39088063
0.547905883	0.537635385	0.733820951	0.724822678	2.145033234	0.392292049
0.408158102	0.610177125	0.825877665	1.333945755	1.900132468	0.461915224
0.339927739	0.58743454	0.6012763	0.818072154	2.413120569	0.329100472
0.316088401	0.425254969	0.682073917	0.616340714	2.27016947	0.452754222
0.31892741	0.619166791	0.50754231	0.809722796	1.027401439	0.364401297
0.489133439	0.424342181	1.027615104	0.610811868	1.393873851	0.423460703
0.399038541	0.452064333	0.605542448	0.708775185	0.963729385	0.256370115
0.345390426	0.364401297	0.572084436	0.582730206	1.028470206	0.38646241
0.433258512	0.660440272	0.751580739	0.682972787	1.470900849	0.308490935
0.421031477	0.505260744	0.642870475	0.575824069	1.169182386	0.274523203
0.364224531	0.39069102	0.555361857	0.512953516	2.444941591	0.332655317
0.453822486	0.43875881	0.814620486	0.687484933	1.833118436	0.2914661
0.488692883	0.490151621	0.726583244	0.63785482	1.072070662	0.485014573
0.428509769	0.511852487	0.600401714	0.565186927	0.949539552	0.332125408
0.347118461	0.372006568	0.534662352	0.347600003	1.023066554	0.278799523
0.381485466	0.536072488	0.689967351	0.792344646	0.983161277	0.293839449
0.322148995	0.487880592	0.555939579	0.500971348	0.906136312	0.306444985
0.32983125	0.542765152	0.435245112	0.602736777	1.067399322	0.199920733
0.414803508	0.489404747	0.703440352	0.579467639	3.195186185	0.323917878

P16284,PECAM1,NEUROLOGY	P50895,BCAM,NEUROLOGY	P20936,RASA1,NEUROLOGY	Q9H2W6,MRPL46,NEUROLOGY	Q9HCN6,GP6,NEUROLOGY	P22676,CALB2,NEUROLOGY
P16284	P50895	P20936	Q9H2W6	Q9HCN6	P22676
PECAM1	BCAM	RASA1	MRPL46	GP6	CALB2
NEUROLOGY	NEUROLOGY	NEUROLOGY	NEUROLOGY	NEUROLOGY	NEUROLOGY
0.689441477	0.518099106	0.57164841	0.722966147	0.315038489	0.740976657
0.769930432	0.434581902	0.682688805	0.887734395	0.484913727	1.227055071
0.675252977	0.715190759	0.865376591	0.527557408	0.291567132	0.727843408
0.561594223	0.461307291	1.136344283	0.753458523	0.261430614	0.905885113
0.776468875	0.583579051	0.744477421	0.947698461	0.288911593	1.027615104
0.583255536	0.504141282	0.812139802	0.740719899	0.198622367	0.76950361
0.69341935	0.534958914	0.910732959	0.853699358	0.333879634	1.24323584
0.587638164	0.513771939	1.022924736	0.806809513	0.299037849	1.016563263
0.562334318	0.404132421	0.951252338	0.618051944	0.331114019	0.584186126
0.566088688	0.399093863	1.247119749	0.876484206	0.209787409	0.894342909
0.566952589	0.416387885	0.998407032	0.853758534	0.208714104	0.750643603
0.686770512	0.564169274	0.648195091	0.755969534	0.39680442	2.0277784
0.624641358	0.407903559	0.855002178	0.92993385	0.223663229	0.79526082
0.47207893	0.439002177	0.65374504	0.787089719	0.14060307	1.030039741
0.609289591	0.302477555	0.584388624	0.885706125	0.128879973	0.600734739
0.485653748	0.394610158	0.643762301	0.78176126	0.300804906	1.236018211
0.60336378	0.444390537	0.769983801	1.017197626	0.484644908	0.869887055
0.681317893	0.571093948	0.861187927	1.235418637	0.287812269	0.864537231
0.67633035	0.484779299	0.734482489	0.842004588	0.338047996	1.435446936
0.522861229	0.337884014	0.967947027	0.817052111	0.407536166	1.427311276
0.518494288	0.451093999	0.785563607	0.684109894	0.205455805	0.856306986
0.505891534	0.266757363	0.595428425	0.724923167	0.198842768	1.003750009
0.600568204	0.317757922	0.758488914	0.64166847	0.260580314	0.907456253
0.414401175	0.313339814	0.792070087	0.866757308	0.222380162	0.985481026
0.466872332	0.483470578	0.945074522	0.977114815	0.334783423	1.678299274
0.611235398	0.419865746	1.106113937	1.086734863	0.403041416	1.423260753
0.777707739	0.402036948	0.919868793	0.805356804	0.44319085	0.903001319
0.553133644	0.438090246	0.732702783	1.026760713	0.366351391	0.535070168
0.732601216	0.572520795	0.93400359	1.044129025	0.354756248	1.216553697
0.751945497	0.39077227	1.035623782	0.862621751	0.257278	1.304774055
0.534254848	0.38734741	1.093535457	0.771051966	0.228426194	0.634591455
0.672636979	0.505996742	0.718519891	0.645012936	0.231374215	0.787744674
0.469403336	0.360282379	0.897945631	0.577222723	0.198374707	0.747735534
0.594150368	0.552750373	0.896204577	0.817278677	0.264327787	0.763870509
0.642024385	0.4118526	0.865196659	0.878065215	0.304159506	0.953761201

Q8IU54,IFNL1,NEUROLOGY	Q16864,ATP6V1F,NEUROLOGY	Q9BRF8,CPPED1,NEUROLOGY	O76070,SNCG,NEUROLOGY	Q9Y624,F11R,NEUROLOGY	Q8N2G4,LYPD1,NEUROLOGY
Q8IU54	Q16864	Q9BRF8	O76070	Q9Y624	Q8N2G4
IFNL1	ATP6V1F	CPPED1	SNCG	F11R	LYPD1
NEUROLOGY	NEUROLOGY	NEUROLOGY	NEUROLOGY	NEUROLOGY	NEUROLOGY
0.729611311	0.699453474	0.703440352	0.272419145	0.340895155	0.959463613
0.707204814	1.196648963	0.446118846	0.231245949	0.283417353	1.140368426
0.575185814	0.861904541	0.654289036	0.371568473	0.303506643	1.625518528
0.711285175	0.862083788	0.591233532	0.333139886	0.311110717	1.381657472
0.808152801	1.314304901	0.503722123	0.501701098	0.336108749	1.21849472
0.693900158	0.862382615	0.314602055	0.232676917	0.313774496	0.914782074
0.796584879	1.243408201	0.482165389	0.215416401	0.279515463	1.282625505
1.158051971	1.019456347	0.436998429	0.191391677	0.22993532	1.332559544
0.825419828	1.066142293	0.493116352	0.102280286	0.262920766	1.015366101
1.094521277	0.96339544	0.432058928	0.072323084	0.216254191	1.070437082
1.178376039	1.217312859	0.587760373	0.137261602	0.234783023	1.085906584
2.526079423	1.229438867	0.788728129	0.489913856	0.397189668	1.118682008
0.808713164	0.954025676	0.355445432	0.216089369	0.227352063	1.059365184
0.616340714	1.006467091	0.530638037	0.227178782	0.163912752	1.060099734
0.664987902	0.916051111	0.29569874	0.094863703	0.191166284	0.887365273
1.059512053	1.118682008	0.492296709	0.206211972	0.212671704	1.412548105
1.09497657	1.204304322	1.38098725	0.16927021	0.324952331	1.301161464
0.834567127	1.05270412	0.379770566	0.34596548	0.302267967	1.178294363
1.026547226	0.762495115	0.688057004	0.207459266	0.278085418	1.069769517
0.768544126	1.504516574	0.526315577	0.314057363	0.262775012	1.470493085
1.264879542	1.088619663	0.290316807	0.245143769	0.227336305	1.175520746
1.291636397	1.356040249	0.791576123	0.395788061	0.308512318	1.120699895
0.744787105	0.867298188	0.414056629	0.26585288	0.241500821	1.11860447
0.857792139	0.98684814	0.321412958	0.15965172	0.206211972	1.586337782
0.749759603	1.443228767	0.430623811	0.226691154	0.247071413	0.927102014
0.560699621	1.260241305	0.381882311	0.177784321	0.280310949	1.085831318
0.688343219	1.035264924	0.462684284	0.146726361	0.269395937	1.228501824
0.722164797	0.816995479	0.75126823	0.16400367	0.261140839	0.986984956
0.748409616	0.99391887	0.492330833	0.137385343	0.247020041	1.06201195
1.189619334	0.747787365	0.29982654	0.420710579	0.216014491	0.852575794
0.823533932	0.609754329	0.524312919	0.149404965	0.238953201	1.162555829
0.578624774	0.803962436	0.425933468	0.190994103	0.244143284	0.889458994
0.80865711	0.712469421	0.381300413	0.111963469	0.218771999	0.819775057
0.67562752	0.90663892	0.494348377	0.162645183	0.2761454	1.139420288
0.849979529	1.036629245	0.762495115	0.13476334	0.318242849	1.14092187

P09104,ENO2,NEUROLOGY	Q16762,TST,NEUROLOGY	P01732,CD8A,NEUROLOGY	Q6P1N0,CC2D1A,NEUROLOGY	P04155,TFF1,NEUROLOGY	Q8WWN9,IPCEF1,NEUROLOGY
P09104	Q16762	P01732	Q6P1N0	P04155	Q8WWN9
ENO2	TST	CD8A	CC2D1A	TFF1	IPCEF1
NEUROLOGY	NEUROLOGY	NEUROLOGY	NEUROLOGY	NEUROLOGY	NEUROLOGY
0.953562892	0.538866573	0.306848833	0.544725005	0.251826149	0.367419474
1.039579435	0.880686222	0.683162173	0.467358002	0.242339251	0.304919432
0.916432165	0.796750542	0.325538482	0.447915967	0.498270131	0.352843417
1.472226862	0.68577157	0.684109894	0.355913855	0.39680442	0.439916006
0.88785747	1.104045783	0.466516496	0.587475259	0.317846036	0.812027223
0.872423175	0.616554358	0.33420379	0.560893978	0.256796955	0.523840678
0.866697231	0.794159118	0.412338192	0.419167858	0.358613076	0.358861734
0.823819397	0.770197316	0.466937059	0.351695797	0.318618071	0.228299563
0.935040008	0.612125765	0.551067137	0.378771582	0.249653667	0.429908041
0.649949711	0.846921406	0.475494316	0.480197567	0.262592934	0.50547092
0.860114123	0.483604643	0.353185985	0.459138081	0.385659619	0.490933663
1.456494244	0.697758645	0.569631171	0.78138204	0.506698689	0.588086387
0.864836909	0.658566029	0.377461132	0.371568473	0.287752427	0.3103999
0.790424735	0.831276308	0.45328804	0.41402793	0.214418308	0.272475799
0.670031116	0.585402171	0.328530678	0.266554049	0.248187094	0.235434883
0.719367058	0.379823217	0.742467613	0.481630947	0.251285617	0.566520474
0.952505944	0.894776952	0.508211174	0.799793799	0.39001459	0.49520576
0.824390623	1.041382451	0.299701872	0.417457143	0.466548833	0.328234775
1.684943248	0.763499967	0.548970298	0.525149466	0.426110646	0.720464874
0.724069457	1.223912143	0.434581902	0.351427745	0.308384038	0.477707688
0.870490223	0.778786618	0.44031259	0.363921704	0.39447342	0.11526289
0.918339815	0.732651997	0.495927111	0.596626515	0.371723036	0.486462331
0.784964873	0.602569686	0.495274415	0.428005132	0.275552668	0.36938572
0.623948993	0.740873943	0.440190526	0.297920644	0.344147754	0.295432408
0.701784505	0.588983858	0.517991382	0.418761292	0.370052021	0.135203087
0.851866936	0.667389086	0.364805655	0.455177137	0.367903677	0.344267047
0.778354887	0.567424363	0.743291492	0.366885044	0.259660775	0.37428263
1.102516311	0.540250347	0.346229366	0.684536797	0.240882251	0.229409971
0.828802351	0.956475553	0.292214566	0.593121675	0.368311921	0.454798689
0.710940141	0.427827167	0.44322157	0.358314914	0.322059689	0.38890778
0.651483254	0.423607489	0.494999852	0.435003827	0.259498841	0.333740806
0.76966364	0.635383707	0.570302792	0.468688077	0.481530805	0.258654823
0.822222069	0.628855302	0.345797657	0.432178737	0.184449783	0.287274133
0.684821547	0.566952589	0.361332768	0.289512993	0.287015389	0.336878439
0.843874287	0.951186405	0.545858908	0.813153713	0.244109441	1.10519428

Q8IWV2,CNTN4,NEUROLOGY	Q8WUW1,BRK1,NEUROLOGY	Q9BXS1,IDI2,NEUROLOGY	Q96F46,IL17RA,NEUROLOGY	P09211,GSTP1,NEUROLOGY	P14209,CD99,NEUROLOGY
Q8IWV2	Q8WUW1	Q9BXS1	Q96F46	P09211	P14209
CNTN4	BRK1	IDI2	IL17RA	GSTP1	CD99
NEUROLOGY	NEUROLOGY	NEUROLOGY	NEUROLOGY	NEUROLOGY	NEUROLOGY
0.783225695	0.754399173	0.84264683	0.721514353	0.825820421	0.484745698
0.509480912	0.581963267	1.249889019	0.954356374	0.643539229	0.428747451
0.741387656	1.083350441	1.281292625	0.396035044	0.822108092	0.519141599
0.532332665	0.798021765	0.711482413	0.672543738	0.831506819	0.376468223
0.629684037	0.993023662	1.17356683	0.50662845	1.058484395	0.470445661
0.502327443	1.078629951	0.949934536	0.328325794	0.519501565	0.497062786
0.602778557	0.798464405	0.827596816	0.524785586	1.034619293	0.405085963
0.506312497	0.76583208	0.884050078	0.479200062	0.817561973	0.419778447
0.382226577	0.703732965	0.776361241	0.373168729	0.949013161	0.36213512
0.421732468	0.597537019	0.968416792	0.40649232	1.01038141	0.299805759
0.480031172	0.64510236	0.975287857	0.364881522	0.590414476	0.437726005
0.736725968	1.428399961	0.789384447	0.52188361	0.959663149	0.701735863
0.430176315	0.952637998	0.727288667	0.544347562	0.821196851	0.420827241
0.419574819	0.436907568	0.759857082	0.488354264	0.778948579	0.364881522
0.398071641	0.499722818	0.776953413	0.478072061	0.586946128	0.282065085
0.395102807	0.867839405	0.940891323	0.270087729	0.734227981	0.283220971
0.665448997	0.817958753	0.710004464	0.771319238	0.707940492	0.451688472
0.889335697	0.905508444	1.03053964	0.68216848	1.128025749	0.527045712
0.534958914	1.194659929	0.733363312	0.616127143	0.714645661	0.462171435
0.538829222	0.685438912	1.104734738	0.626462475	1.148220724	0.387052185
0.460795969	0.933938852	0.671984565	0.615999037	0.957006083	0.402790064
0.386301718	0.699501958	0.866577089	0.790369949	1.077359696	0.44077063
0.473816387	0.632966039	0.936921447	0.772871247	0.830930662	0.442454191
0.419284092	0.595345886	0.771693575	0.702660644	0.671239723	0.358190753
0.559457328	0.964263938	0.885153765	0.494725442	1.559085449	0.430027253
0.455587478	1.392521884	1.280049854	0.467390398	1.149096533	0.459998161
0.52007803	0.444513765	0.906764616	1.000970877	0.769450274	0.369975079
0.544347562	0.724119647	0.705540106	0.595965202	0.626288807	0.354092944
0.632746707	0.687389634	0.876301966	0.408582693	1.357168641	0.457644739
0.424989764	0.89180488	0.775339462	0.908085472	0.784420965	0.413827091
0.356877289	0.204390493	0.737032427	0.355470071	0.716878237	0.291143033
0.605878325	0.84621725	0.832025702	0.343075974	0.518997682	0.418558157
0.40615435	0.418239144	1.191352216	0.363543524	0.68448935	0.319082192
0.491887398	0.557289934	0.595717398	0.330311705	0.761333254	0.384005798
0.518997682	0.890199133	1.040949442	0.35365143	0.927744855	0.372625936

O95817,BAG3,NEUROLOGY	Q92917,GPKOW,NEUROLOGY	P30519,HMOX2,NEUROLOGY	O95630,STAMBP,NEUROLOGY	Q9NPH6,OBP2B,NEUROLOGY	Q92854,SEMA4D,NEUROLOGY
O95817	Q92917	P30519	O95630	Q9NPH6	Q92854
BAG3	GPKOW	HMOX2	STAMBP	OBP2B	SEMA4D
NEUROLOGY	NEUROLOGY	NEUROLOGY	NEUROLOGY	NEUROLOGY	NEUROLOGY
0.497269551	0.603112901	0.66342257	0.888781073	0.289312387	0.395815496
0.535292743	0.892052176	0.778786618	0.809891191	0.343551908	0.556865184
0.431580025	0.851217667	0.722365051	0.835145805	0.403432719	0.484880117
0.435275282	0.907644973	0.583174685	0.621187197	0.263048367	0.655878278
0.392999668	0.877882646	0.624338354	1.035049669	0.298540797	0.556517901
0.643583837	0.662273942	0.519861781	0.876241227	0.316176051	0.344052349
0.226282983	0.938546416	0.76583208	0.662090346	0.38483182	0.392156114
0.426820093	0.792784137	0.581116772	0.438363627	0.361984543	0.318507666
0.281811033	0.769450274	0.516628809	0.554092982	0.474901428	0.554361895
0.204093197	0.827596816	0.815976778	0.6012763	0.300471487	0.413655021
0.461691155	0.819320604	0.787690073	0.521269011	0.416099367	0.378535366
0.710398283	0.842471624	0.705491204	1.30035001	0.476285984	0.401257427
0.323357057	0.747839199	0.706176149	0.438606774	0.228378699	0.361257639
0.458343144	0.874724132	0.845220697	0.504385952	0.311261706	0.289613348
0.153307481	0.627548993	0.490457489	0.503233545	0.26359593	0.352256931
0.219592396	0.844517954	0.576223339	0.643093316	0.411253539	0.441473884
0.424871948	0.965534691	0.563153453	1.279783703	0.224891338	0.485350876
0.418587171	0.711087992	0.839964348	0.708136802	0.222041308	0.413167878
0.240782092	0.686104389	1.07937786	0.763499967	0.412795744	0.850627853
0.272608037	0.824276346	0.733414147	0.536965015	0.312905734	0.423842452
0.341533737	0.798907291	0.653699727	0.474506581	0.215715239	0.297136965
0.38291604	0.88337628	0.530748392	0.855654333	0.275705509	0.425903946
0.333093706	0.798409062	0.699986983	0.52850902	0.236088553	0.344625175
0.319901575	0.88902753	0.800237421	0.417515019	0.409149502	0.319369844
0.467552411	0.858505927	1.288149465	0.600193666	0.503652297	0.452973953
0.289573202	0.886750412	1.119302509	0.649319301	0.361157491	0.305660069
0.219546737	0.759541132	0.414114034	0.600276877	0.272211515	0.369821242
0.248015123	0.763976412	0.644610682	1.101294259	0.31718578	0.374646012
0.332955205	0.77630743	0.741439047	0.863998073	0.451125268	0.392890721
0.379454813	0.856663188	0.648824409	0.555400353	0.396859432	0.399176861
0.303191245	0.593080565	0.590128075	0.276950491	0.424224545	0.33028881
0.349629785	0.688152396	0.573156093	0.657334677	0.360882227	0.398734405
0.264859654	0.698339266	0.771212318	0.487306037	0.371465466	0.362260648
0.202052205	0.661035657	0.687532587	0.501110266	0.541449991	0.304708151
0.216284173	0.877152747	0.787144277	1.16934448	0.202332503	0.377958568

P14625,HSP90B1,NEUROLOGY	Q92851,CASP10,NEUROLOGY	Q10588,BST1,NEUROLOGY	P17538,CTRB1,NEUROLOGY	P00918,CA2,NEUROLOGY	P05455,SSB,NEUROLOGY
P14625	Q92851	Q10588	P17538	P00918	P05455
HSP90B1	CASP10	BST1	CTRB1	CA2	SSB
NEUROLOGY	NEUROLOGY	NEUROLOGY	NEUROLOGY	NEUROLOGY	NEUROLOGY
0.458692747	0.34077703	0.597495602	0.244736299	1.057750964	0.954554847
0.360232437	1.060320199	0.386355275	0.467520004	1.032613239	1.198973689
0.532000682	0.334041673	0.587556706	0.362034728	1.299448988	1.654161391
0.758857025	1.22010051	1.908448162	0.332102388	0.756179163	1.291725929
0.515305537	0.86298058	0.588902213	0.263468063	0.775070796	1.214447395
0.51192345	0.677597285	0.288251496	0.356926766	0.736777035	1.278099363
0.504350992	0.911427623	0.55632506	0.412938832	1.322254605	1.484935225
0.782900028	0.230829577	0.507999858	0.2731187	0.582245706	1.368883813
0.538269282	2.551242436	0.72076457	0.282730612	0.856010264	1.435844982
0.52278875	0.916432165	0.388288262	0.200823499	1.434750622	1.660709809
0.516127712	1.011993483	0.192642777	0.401257427	0.572084436	1.218325813
0.389420301	0.504176227	0.739488696	0.407225554	1.892902366	2.41579829
0.70144408	0.938351271	0.38958229	0.250920109	0.562997336	1.085756056
0.438363627	0.569354851	0.234376528	0.355174522	1.023208391	1.36717697
0.41884838	0.674457761	0.536667341	0.215416401	0.777384367	1.543601125
0.420973113	1.064591535	0.470902406	0.415638154	1.39929486	1.955449122
0.889150784	0.649184293	0.659114037	0.343028417	3.481478225	1.282181057
0.54231388	0.467649646	0.516020398	0.338024565	1.334778173	1.557033516
0.542765152	0.704562698	1.815541616	0.177796644	0.677879149	2.389816754
1.210665238	1.016351897	0.293574792	0.259660775	0.648689504	2.13154572
0.390176826	0.747321016	0.244736299	0.206583939	0.560583039	1.843311638
0.534477085	0.88687335	0.444482955	0.318132573	1.244011653	1.518976974
0.640202404	0.636176947	0.352354611	0.302351785	0.575863984	1.174543382
0.56923647	1.399876932	0.379034218	0.25107669	0.660211421	2.177088417
0.603531092	0.601985232	0.437453022	0.237220402	0.692410736	1.397065828
0.529792748	0.535107257	0.421235812	0.333833352	1.751875195	2.309050939
0.491580639	1.023066554	0.19185656	0.548665969	1.228927664	1.193335743
0.502362263	0.932774336	0.390636862	0.292234822	2.824117276	0.729712463
0.621833394	1.198807488	0.462043312	0.290196093	1.851635336	1.569494529
0.431879278	0.464741376	0.505646133	0.344386382	0.68249955	0.937636086
0.307551518	0.441810619	0.437149908	0.446799662	1.225100411	1.025196174
0.38799232	0.520546879	0.312407285	0.551908114	0.754660673	1.382903033
0.386034047	0.70305039	0.412624102	0.320456403	0.830354904	0.861008867
0.508951469	0.845924024	0.455619058	0.338516955	0.717773219	1.38386192
0.464515937	1.008562158	0.447450501	0.221349804	2.820791437	1.598479232

Q9UJ72,ANXA10,NEUROLOGY	P19440,GGT1,NEUROLOGY	Q4LE39,ARID4B,NEUROLOGY	Q92597,NDRG1,NEUROLOGY	Q99497,PARK7,NEUROLOGY	P61244,MAX,NEUROLOGY
Q9UJ72	P19440	Q4LE39	Q92597	Q99497	P61244
ANXA10	GGT1	ARID4B	NDRG1	PARK7	MAX
NEUROLOGY	NEUROLOGY	NEUROLOGY	NEUROLOGY	NEUROLOGY	NEUROLOGY
0.186545569	0.357124743	0.791466394	0.444667848	0.785345833	0.384245428
0.209860129	0.349969233	0.736725968	0.344267047	0.629858647	1.384245661
0.688486371	0.27211719	1.165945214	0.355963199	0.710349044	0.831852705
0.470869767	0.369436931	1.242202176	0.504735687	0.611023596	1.230973752
0.36938572	0.335573338	1.294594249	0.554554056	0.567227742	1.11829437
0.555554364	0.248445273	1.007583918	0.596171783	0.505260744	1.132962414
0.480164284	0.398900269	1.522666508	0.347889249	0.582366793	1.734476146
0.26967618	0.340635334	1.655193632	0.455966583	0.469435874	1.528058737
0.38291604	0.230845578	1.275974944	0.36645298	0.521919786	1.779164615
0.583538602	0.2516691	1.139657249	0.362662631	0.517524834	0.967209285
0.361859111	0.251547019	0.933097667	0.39012274	0.475988954	1.300440147
0.607686859	0.396694417	1.544350266	0.699695928	1.060026256	1.413233644
0.823362701	0.23702317	0.793113915	0.361959453	0.47976506	1.750418632
0.267906223	0.255553986	1.370402796	0.540812349	0.459297234	1.153085907
0.324682155	0.23617039	1.230291345	0.399342909	0.407705691	0.782900028
0.1965952	0.190478491	0.815015838	0.364426556	0.664757475	2.080600533
0.401702682	0.322752461	1.336907825	0.339645113	1.349383101	1.159578107
0.854883658	0.369283319	0.876059037	0.398817328	0.64478943	0.635603952
0.238176008	0.336481712	1.097560145	0.504805663	0.532443372	1.389243991
0.51687954	0.341983826	1.725123922	0.582366793	0.525040275	1.766017942
0.547298573	0.276931295	1.134298227	0.413511684	0.373997362	1.037563763
0.865736566	0.277450056	1.093990341	0.380481971	0.740411906	1.448038539
0.558682293	0.256458981	1.180992661	0.415436534	0.491989694	0.800847805
0.325515918	0.257759946	1.4037636	0.403376796	0.446397236	1.450449433
0.374905787	0.272343625	1.259193501	0.432448429	0.385098658	1.351817129
0.479034013	0.347262854	1.630709728	0.367419474	0.698581334	1.355852275
0.320012464	0.286478743	1.215457965	0.362285759	0.46836332	1.24893639
0.312234097	0.274961208	1.197893791	0.388719126	1.163684528	1.086810192
0.357372369	0.300596476	1.684359393	0.422258977	0.876301966	1.175683719
0.279903222	0.251843605	1.041526827	0.426613049	0.477707688	1.055041684
0.339315679	0.29617053	0.939978719	0.333972218	0.481530805	0.560427634
0.22326051	0.300429836	1.214110726	0.706078259	0.493218904	1.233450654
0.230781583	0.217652727	0.847038823	0.240165362	0.528399131	0.880075988
0.519465557	0.266480154	0.975017488	2.806943452	0.47883483	1.309485423
0.259678774	0.30665747	1.253359256	0.410257041	1.311302014	1.284493865

Q9P0K1,ADAM22,NEUROLOGY	P28799,GRN,NEUROLOGY	Q5JZY3,EPHA10,NEUROLOGY	Q86Z14,KLB,NEUROLOGY	Q6ISS4,LAIR2,NEUROLOGY	Q6UXK2,ISLR2,NEUROLOGY
Q9P0K1	P28799	Q5JZY3	Q86Z14	Q6ISS4	Q6UXK2
ADAM22	GRN	EPHA10	KLB	LAIR2	ISLR2
NEUROLOGY	NEUROLOGY	NEUROLOGY	NEUROLOGY	NEUROLOGY	NEUROLOGY
0.438850057	0.588657346	0.739693755	0.207186226	0.285744968	1.019597683
0.615700225	0.507612675	0.423754326	0.716133271	0.352794506	1.561681229
0.685819105	0.78719884	0.29889279	0.111560638	0.422083401	0.961860781
0.400479417	0.552558838	0.428301905	0.311455942	0.200003895	0.67881954
0.662779093	0.584793832	0.477840155	0.49720062	0.131223681	1.516767545
0.582972607	0.475329551	0.385552706	0.373272208	0.113644518	0.664711399
0.481130446	0.529132156	0.551105336	0.290236325	0.252315371	1.007863318
0.488455825	0.438272481	0.473882076	0.320900958	1.016774673	0.996264001
0.245041838	0.410626887	0.451876363	0.302603378	0.182947296	0.858208443
0.283908904	0.427827167	0.351354675	0.309883963	0.28269142	0.949078944
0.304834902	0.331091069	0.308769038	0.136900536	0.217501914	0.789439165
0.90802253	0.635119513	0.553478814	0.230445899	0.480330725	1.344714591
0.385125352	0.373712312	0.500971348	0.41085465	0.026692903	0.788345528
0.396831925	0.415321367	0.354707072	0.342576953	0.278664282	1.345926847
0.175300255	0.303569762	0.449221848	0.218226771	0.064475729	0.822336061
0.26288432	0.365463695	0.311758327	0.235826868	0.34169949	1.013467623
0.359384477	0.509269069	0.322417063	0.531595206	0.130660955	1.256229453
0.251442426	0.715289912	0.376389947	0.507260947	0.353921179	0.760331254
0.329419988	0.460700159	0.598324482	0.416301309	0.030713144	1.100760038
0.309948408	0.423695585	0.52660751	0.750227473	0.336271869	1.242632765
0.29641698	0.480763742	0.405338748	0.206870524	0.275075585	0.878795873
0.283103208	0.559884055	0.319391982	0.258923892	0.172002328	0.678302164
0.301577353	0.440007494	0.435516716	0.945140032	0.030738701	0.754242317
0.359035897	0.282985493	0.294839148	0.495514782	0.095914982	0.842413231
0.377958568	0.530785182	0.534069722	0.656105627	1.864772973	0.880747268
0.417978315	0.528069602	0.603698449	0.661218961	0.496959435	1.27641724
0.565970985	0.406689599	0.374178871	0.361859111	0.31355708	0.924727373
0.393899639	0.454893271	0.612635129	0.192629425	0.230429926	0.680940194
0.384138908	0.603949572	0.505085664	0.611235398	0.278567721	0.996402123
0.520655135	0.317383713	0.257206677	0.184002848	0.255465433	1.018396947
0.346205368	0.374620045	0.276662689	0.313731001	0.273990922	0.7040257
0.43080294	0.534106742	0.378063375	0.954224081	0.226518376	1.013537874
0.318110523	0.384032416	0.333047533	0.19225593	0.206598259	0.576143463
0.477509056	0.492262587	0.35128162	0.406041755	0.243653018	0.574428805
0.301117821	0.456282744	0.303780254	0.77335354	0.350066278	1.32951495

Q96B36,AKT1S1,NEUROLOGY	P01833,PIGR,NEUROLOGY	P14780,MMP9,NEUROLOGY	Q96PP9,GBP4,NEUROLOGY	P22749,GNLY,NEUROLOGY	Q9UM07,PADI4,NEUROLOGY
Q96B36	P01833	P14780	Q96PP9	P22749	Q9UM07
AKT1S1	PIGR	MMP9	GBP4	GNLY	PADI4
NEUROLOGY	NEUROLOGY	NEUROLOGY	NEUROLOGY	NEUROLOGY	NEUROLOGY
1.310484237	0.367343079	1.107571626	1.370022892	0.096261319	0.518063196
1.873972783	0.308426792	0.623689554	1.399197872	0.174451753	0.540362701
2.491647533	0.408809322	1.375351141	1.390978384	0.220415869	0.718470088
1.649238448	0.442945161	0.330632397	1.288149465	0.105872514	0.632132984
2.241244434	0.426317446	0.708971727	1.57385213	0.14733785	0.8489197
1.901713612	0.307359717	0.735603373	1.162314108	0.155339863	0.631432315
2.130511738	0.406266975	0.745561878	1.496922987	0.20514274	0.997023905
1.726200446	0.327939139	0.482198811	1.1045816	0.111197788	0.536853368
2.171963713	0.294124731	0.529829471	1.377354572	0.153042049	0.782682992
2.413622415	0.249601758	0.566991888	1.320880544	0.059854354	0.68491649
1.39784074	0.309068815	0.606971213	1.210077963	0.106660655	0.806306356
3.137248916	0.382253072	2.281053051	1.224930588	0.247911998	0.770517698
2.210541777	0.252280395	0.657015814	1.129199191	0.099063678	0.828974713
1.988940337	0.26135814	0.628550254	1.20957481	0.094797972	0.89428092
1.53166416	0.219288187	0.743497605	1.002081605	0.055391177	0.699938466
2.203199291	0.26266575	1.240051468	1.531770331	0.228743079	0.707793295
5.12872423	0.41702333	1.435944511	1.629692755	0.084711403	0.946057647
1.386358141	0.649769531	0.677409442	1.458514774	0.11005526	0.338587355
1.53485248	0.442730294	0.531153222	1.650954088	0.119683817	0.661769176
3.212507895	0.288911593	0.647566384	1.66197652	0.069685389	1.02072908
1.212512819	0.286260397	0.768597399	1.107187837	0.125738643	0.537672653
3.18413168	0.271495464	0.775877073	1.247292648	0.067479823	0.709070018
1.779411276	0.26434611	0.838219506	1.420993544	0.072579202	0.671612041
1.009891289	0.346181372	0.355938526	2.330759262	0.132769898	1.05709131
1.114657127	0.438880477	0.524567379	1.37783201	0.155598494	0.855179989
2.655162208	0.34992072	0.689823891	1.615857528	0.162600094	0.770143931
1.42050115	0.313274664	0.744890362	1.71166456	0.182871226	0.758699241
5.135127152	0.338986565	0.916051111	1.171453753	0.14058358	0.862442393
3.156006114	0.316351425	0.771479646	1.412254404	0.109613698	0.738873863
1.862576911	0.31281899	0.469403336	1.220354249	0.13187107	0.519033657
1.719632164	0.301702802	0.495240086	1.355382454	0.175713872	0.69140359
2.637004439	0.30729581	0.77335354	1.216047852	0.0547461	0.710890864
1.346206754	0.375764323	0.459711289	0.986984956	0.088541646	0.550608963
1.317132058	0.330243026	0.549503281	1.169101347	0.114919856	0.816146473
4.911283148	0.354584161	1.40746593	1.141396465	0.153711819	0.797579371

Q9Y2V2,CARHSP1,NEUROLOGY	Q8IUN9,CLEC10A,NEUROLOGY	Q10589,BST2,NEUROLOGY	Q15126,PMVK,NEUROLOGY	P26440,IVD,NEUROLOGY	P10145,CXCL8,NEUROLOGY
Q9Y2V2	Q8IUN9	Q10589	Q15126	P26440	P10145
CARHSP1	CLEC10A	BST2	PMVK	IVD	CXCL8
NEUROLOGY	NEUROLOGY	NEUROLOGY	NEUROLOGY	NEUROLOGY	NEUROLOGY
0.873875706	0.418877414	0.45350803	0.456884055	0.131770561	0.296314268
1.626307426	0.318684333	0.400757104	0.44596426	0.165648839	0.23906917
1.358015551	0.314253344	0.407423189	0.49993069	0.148826155	0.339386245
0.870007655	0.371053726	0.39077227	0.498857614	0.158208608	0.290437571
1.523827926	0.564912763	0.505050656	0.475263661	0.201604535	0.526936127
0.6341957	0.410313919	0.317383713	0.315759928	0.14482675	0.53418079
1.01550687	0.215595654	0.304940568	0.73285516	0.209758329	0.330265917
0.643182474	0.24348419	0.273383864	0.40584479	0.268129154	0.270125174
0.716083634	0.227336305	0.303443537	0.292518546	0.220583992	0.245960747
1.153405654	0.214344009	0.207387378	0.442362195	0.219470661	0.323446723
0.853699358	0.181281047	0.286121537	0.274466124	0.234035615	0.245228745
2.977909685	0.371362488	0.580311732	0.502118575	0.205726565	0.580512887
0.76742624	0.228568738	0.426849679	0.257652769	0.190148703	0.208150655
1.736882308	0.233549458	0.245534899	0.408695992	0.147746924	0.306190197
0.590578197	0.200295234	0.191909761	0.299618788	0.139042709	0.301326613
0.910228081	0.231310073	0.252297882	0.503128912	0.125904347	0.211451691
3.012998595	0.352794506	0.442055679	0.912249272	0.115719187	0.501596783
1.257449098	0.413081971	0.324704661	0.433168428	0.150527606	0.302582404
0.999930688	0.237368434	0.529719308	0.274009914	0.196363678	0.247654373
1.838462819	0.251721439	0.35769454	0.303548721	0.224051144	0.280388678
0.81552443	0.253507441	0.302142283	0.316110311	0.213676477	0.324884766
1.709648807	0.447760758	0.318573904	0.530454164	0.245790319	0.700181088
0.826679491	0.314645671	0.309175949	0.368439591	0.127264328	0.334621025
1.376781866	0.196826994	0.178203799	0.303401474	0.242188119	0.275820196
1.47641674	0.288511354	0.271608399	0.478436711	0.175190931	0.423695585
3.020526408	0.227320547	0.330128592	0.54536726	0.256885969	0.354117489
1.124278924	0.281537695	0.334806629	0.50121448	0.138216332	0.393054153
1.462665627	0.204915355	0.364906815	0.425107613	0.141286938	0.598490395
1.746540382	0.293839449	0.423490056	0.371362488	0.156887248	0.441290318
0.763923459	0.550570799	0.228473699	0.363997387	0.23940082	1.35162974
0.745045273	0.228347041	0.270443663	0.506488002	0.138523246	0.257010641
0.938936827	0.204943764	0.403712455	0.413569013	0.174427571	0.205313443
0.787963113	0.186687857	0.244719336	0.303401474	0.197168365	0.257492087
0.656833675	0.258780354	0.308234446	0.416359025	0.124515737	0.583457712
4.017783985	0.259193241	0.406266975	0.496236583	0.175726052	0.326148297

Q9UJY5,GGA1,NEUROLOGY	P01236,PRL,NEUROLOGY	Q2TAL6,VWC2,NEUROLOGY	Q9H0C8,ILKAP,NEUROLOGY	Q6UX15,LAYN,NEUROLOGY	P63313,TMSB10,NEUROLOGY
Q9UJY5	P01236	Q2TAL6	Q9H0C8	Q6UX15	P63313
GGA1	PRL	VWC2	ILKAP	LAYN	TMSB10
NEUROLOGY	NEUROLOGY	NEUROLOGY	NEUROLOGY	NEUROLOGY	NEUROLOGY
1.350037985	0.120041071	0.52090782	0.625507895	0.715934744	0.305596516
1.521822397	0.584955994	0.556093739	0.157148456	0.613952948	0.240681974
1.299899421	0.406295136	0.598697852	0.44723345	0.578103615	0.19133862
1.144010267	0.601317979	0.399038541	0.54231388	0.451876363	0.376155216
1.954500563	0.77271055	0.36213512	0.677268593	0.689680461	0.636397468
0.88227481	0.377434969	0.441626913	0.382014684	0.70637197	0.333047533
1.119380096	0.248979696	0.399536718	1.118759552	0.493116352	0.297446065
1.06201195	0.278027598	0.39302691	0.648824409	0.564169274	0.266277051
1.474269217	0.120007793	0.245688119	0.631563631	0.305617699	0.164436218
1.136659388	0.147307215	0.175239511	0.666418336	0.339645113	0.202416668
0.947567091	0.111259467	0.211554312	0.659479628	0.436635096	0.315322495
1.656800622	0.387669731	0.585037092	0.832544908	0.817165386	0.929740496
1.399003915	0.226816893	0.253577738	0.575784158	0.44217826	0.173378871
1.24841708	0.100425671	0.273971931	0.594974606	0.391260127	0.234539042
1.299088755	0.189425177	0.139148764	0.338071429	0.215088158	0.13651203
1.22748041	0.213203053	0.210617906	0.869947353	0.232918962	0.257420704
1.318959257	0.152914805	0.325087503	0.566402681	0.519105616	0.176862489
2.80247208	0.165258914	0.289131961	0.556595056	0.449969774	0.414918532
1.549174866	0.329214549	0.373013565	0.632089169	0.429967643	0.314776556
1.654161391	0.543103852	0.255447726	0.792179899	0.422054146	0.271834411
1.159095952	0.27486593	0.305257786	0.657015814	0.429074479	0.333393989
1.24039533	0.309647778	0.27912824	0.652929894	0.456630775	0.585321022
1.050153339	0.385018587	0.260959893	0.451563255	0.369975079	0.322528823
1.795393404	0.241718534	0.255766638	0.474901428	0.317713875	0.138513644
1.582164931	0.316878131	0.529022138	1.096419582	0.496546248	0.165007099
1.338205798	0.155060164	0.337743521	0.661998567	0.359533972	0.201227586
1.636030903	0.376520416	0.369334516	0.335643126	0.400646006	0.204404661
1.626307426	0.223864861	0.240215309	0.496408595	0.314405857	0.206583939
1.451354553	0.217924454	0.371645746	0.475626169	0.405872922	0.192082767
0.985481026	0.023149101	0.358613076	0.470445661	0.389636301	0.279437975
1.000762753	0.21848407	0.286995496	0.43452166	0.295125401	0.179244406
1.32565007	0.14958112	0.238093477	0.622135183	0.494005839	0.231438374
1.319782323	0.248410834	0.221580066	0.496821668	0.291163214	0.179356259
1.348915522	0.402176308	0.316812244	0.710743054	0.417457143	0.179070551
0.858148959	0.226738298	0.312299031	1.794398103	0.378194425	0.211202673

P13647,KRT5,NEUROLOGY	P20827,EFNA1,NEUROLOGY	Q14696,MESD,NEUROLOGY	Q9UKK9,NUDT5,NEUROLOGY	Q9NS71,GKN1,NEUROLOGY	O14523,C2CD2L,NEUROLOGY
P13647	P20827	Q14696	Q9UKK9	Q9NS71	O14523
KRT5	EFNA1	MESD	NUDT5	GKN1	C2CD2L
NEUROLOGY	NEUROLOGY	NEUROLOGY	NEUROLOGY	NEUROLOGY	NEUROLOGY
0.43615112	0.622998243	0.747165632	0.519933854	0.653699727	0.498132001
0.83676824	0.464194071	0.816655771	0.509975555	1.020658331	0.845689517
0.66595657	0.64792557	1.193170323	0.502292626	1.189784262	0.530858769
0.555246385	0.389096525	0.795922574	0.469956784	1.132883885	0.788728129
0.609754329	0.647386865	0.788564134	0.498132001	1.207313211	0.650175005
0.809498325	0.468136124	0.92993385	0.594150368	0.785236968	0.652658404
0.951977908	0.476318999	0.945467649	0.494965543	1.310938494	0.821481505
0.659936903	0.455934979	0.839964348	0.459711289	1.31348525	0.60865643
0.707204814	0.41754396	0.821766258	0.452973953	0.690206517	0.445469944
0.46432279	0.468688077	0.874057442	0.370976576	0.855179989	0.810452759
0.889274055	0.345270744	0.769983801	0.454357563	1.159497734	0.779002574
0.946910515	0.661081478	0.76540753	0.925112036	1.254054459	0.605080921
0.794159118	0.340729792	0.74046323	0.357868136	0.767958364	0.563817436
0.711482413	0.400257405	0.780353653	0.387857875	1.219846824	0.782194881
0.49520576	0.332470905	0.806362247	0.323626129	0.703489112	0.451625859
0.451062733	0.357669747	0.698871926	0.460221408	0.920634237	0.800958833
0.719167634	0.444359735	1.018114627	0.803572445	0.865016765	0.834682831
0.685961732	0.60202696	1.01494391	0.477807035	0.993092495	0.90463016
0.589269704	0.397465074	0.829607016	0.413454363	1.12015626	0.620197665
1.404736952	0.438850057	0.610811868	0.325922306	1.605808661	0.729156297
0.449377563	0.437968799	0.807816769	0.453256621	1.123422031	0.866817389
0.936207352	0.447978066	0.975625925	0.580915408	0.822678132	0.935299292
0.535181444	0.464999155	0.692458732	0.43569788	0.901125531	0.560116953
0.683493726	0.379560035	0.597495602	0.381247558	0.960395135	0.474375038
1.065034378	0.415839872	0.822450069	0.452221033	1.818816504	0.964731916
0.916876928	0.47463816	0.713161141	0.491989694	1.63104886	1.173892257
0.544573996	0.483537606	0.768437591	0.483437067	1.013608129	0.70514898
0.52278875	0.395705768	0.732702783	0.671937988	0.730623468	0.75917269
0.817788681	0.620627703	0.929418328	0.611362514	1.419221726	0.781057139
0.679667008	0.544762764	0.886074556	0.467293217	0.928903092	0.751893378
0.506733811	0.440495749	0.700375246	0.331527396	0.555169417	0.646758942
0.599237577	0.470935048	0.740309271	0.399259876	1.364809884	0.529058808
0.65012994	0.362738052	0.821139932	0.431969094	0.450281778	0.672170904
0.669334833	0.60252792	1.08809159	0.35697625	0.988148656	0.807760778
0.564012875	0.397685537	0.932386486	0.67708084	1.230461911	0.947632774

Q8TDQ0,HAVCR2,NEUROLOGY	P35237,SERPINB6,NEUROLOGY	Q08708,CD300C,NEUROLOGY	Q08629,SPOCK1,NEUROLOGY	Q13093,PLA2G7,NEUROLOGY	O00308,WWP2,NEUROLOGY
Q8TDQ0	P35237	Q08708	Q08629	Q13093	O00308
HAVCR2	SERPINB6	CD300C	SPOCK1	PLA2G7	WWP2
NEUROLOGY	NEUROLOGY	NEUROLOGY	NEUROLOGY	NEUROLOGY	NEUROLOGY
0.420448208	0.554515618	0.352232516	0.494622578	0.201534677	0.623862501
0.514377699	0.767798688	0.493800431	0.544498508	0.24760288	0.488692883
0.492296709	0.549655657	0.585767477	0.603949572	0.25151215	0.590455402
0.404384611	1.170885498	0.85245761	0.642024385	0.526936127	0.439916006
0.380745793	0.571133534	0.355273011	0.83294896	0.30934744	0.790369949
0.364426556	0.547146851	0.526571009	0.516342408	0.347696392	0.603071097
0.336178648	0.52453102	0.353945711	0.517130392	0.365185149	0.479831575
0.448910579	0.577222723	0.469956784	0.376885971	0.275304482	0.34642141
0.325786787	0.494177078	0.395733197	0.419167858	0.32983125	0.414861016
0.283063964	0.536072488	0.312342328	0.35774413	0.128291723	0.443467413
0.323738309	0.561399623	0.386221397	0.438789224	0.245909606	0.436514051
0.607434182	0.720315074	0.523151245	0.613059923	0.247894815	0.783388579
0.301891073	0.510966283	0.274923093	0.464838027	0.221027839	0.392945191
0.281654807	0.438150983	0.288411381	0.430116684	0.244109441	0.401507822
0.210122127	0.552750373	0.300492315	0.361984543	0.190412487	0.281908718
0.282495541	0.502083772	0.348516774	0.426317446	0.259193241	0.529058808
0.382809888	0.565226104	0.409802304	0.486597226	0.26359593	0.699938466
0.408695992	0.537486342	0.34820287	0.438485184	0.41402793	0.41647448
0.41549413	1.102057883	0.79526082	0.504525817	0.261321911	1.133119486
0.343385256	0.552137694	0.40598547	0.403824403	0.255678012	0.416157055
0.353063601	0.503059168	0.409546736	0.380165627	0.324907286	0.755236293
0.393544859	0.576423077	0.305935621	0.478270927	0.181117769	0.579829243
0.323805636	0.556903784	0.333509555	0.393272169	0.192429248	0.502048971
0.283358424	0.507260947	0.310163323	0.371413974	0.325967492	0.335526821
0.4144299	0.452189689	0.41148165	0.370205953	0.21638914	0.433288545
0.257563488	0.408384496	0.279709275	0.47467106	0.352208102	0.454987874
0.423490056	0.487508744	0.425579334	0.431550112	0.27572462	0.317625798
0.328075553	0.558101721	0.261666293	0.365413034	0.199284305	0.651483254
0.41091161	0.610515573	0.425785876	0.405732282	0.268650048	0.431580025
0.355617937	0.526461523	0.286022392	0.606508597	0.31281899	0.381802909
0.27918629	0.519141599	0.249342377	0.402120558	0.262775012	0.30038819
0.383473822	0.551219947	0.335643126	0.485620086	0.229648617	0.433709215
0.275419001	0.457613018	0.333833352	0.32778006	0.161555302	0.355494711
0.385606159	0.56864493	0.338963069	0.381379711	0.222472667	0.434762677
0.355297637	0.465805636	0.40796011	0.507753435	0.339786397	0.574428805

P08648,ITGA5,NEUROLOGY	P12429,ANXA3,NEUROLOGY	O94813,SLIT2,NEUROLOGY	Q13308,PTK7,NEUROLOGY	P04118,CLPS,NEUROLOGY	P56279,TCL1A,NEUROLOGY
P08648	P12429	O94813	Q13308	P04118	P56279
ITGA5	ANXA3	SLIT2	PTK7	CLPS	TCL1A
NEUROLOGY	NEUROLOGY	NEUROLOGY	NEUROLOGY	NEUROLOGY	NEUROLOGY
0.614421242	0.244939949	0.534773544	0.669659675	0.211789064	3.567219522
0.70705777	0.264474402	0.759383207	1.172103525	0.291304522	5.020043968
0.695537401	0.29441029	0.720065475	0.896204577	0.252507825	5.731449314
0.60098463	0.35294126	0.557058212	0.742724977	0.645504921	4.013052416
0.751007906	0.395157583	1.265756594	0.537486342	0.22332242	6.538229417
0.617666504	0.255819829	0.422962012	0.64403009	0.247071413	5.029099161
0.614847274	0.264181253	1.255881201	1.256926248	0.191976284	4.0552757
0.587638164	0.197332433	0.958200851	0.787853886	0.174899734	2.054660456
0.626549327	0.283397709	0.779002574	0.810846089	0.222164468	2.338040628
0.458216082	0.232741438	0.667527881	0.821823221	0.126543034	6.218212845
0.555554364	0.302330828	0.667574152	0.934716004	0.294961794	1.439133072
0.688247801	0.468688077	1.0977884	1.062822003	0.274199909	5.74218575
0.496339783	0.200920963	0.724370651	0.98677974	0.232612414	2.273949161
0.388530564	0.164083264	0.485653748	1.050663001	0.315978872	1.13115764
0.402734229	0.239832653	0.734635236	0.557289934	0.114015352	1.049134758
0.484208396	0.311326438	0.536667341	0.666002732	0.34393313	8.824411082
0.480497224	0.21459673	1.905011887	0.930385166	0.297941295	5.330238177
0.722314982	0.237879029	0.420856411	0.789329733	0.111197788	7.571084657
0.656651588	0.439458854	0.517596584	1.069991993	0.131050976	7.989471098
0.774748521	0.250260065	0.777007269	0.907707888	0.325132572	6.225544396
0.447605603	0.224906927	0.690828736	1.132883885	0.11481635	1.880478745
0.558798479	0.246148354	0.88779593	0.678631358	0.232967401	2.378084535
0.500312014	0.202683422	0.608614242	0.70144408	0.242087417	2.444941591
0.314449446	0.250781008	0.742107452	0.696309205	0.239849278	3.166962973
0.648509674	0.186287141	0.494314112	0.576742803	0.181431895	1.470900849
0.558372579	0.22131912	0.905445681	0.843464936	0.405198293	2.030028526
0.578945721	0.176752191	0.632658996	0.76742624	0.152851223	6.204435693
0.54638887	0.343694817	0.556402189	0.938936827	0.290477837	2.957544754
0.649409322	0.287572973	1.062674674	0.909660428	0.285250239	1.160864833
0.652522702	0.200142575	0.649094303	0.919550046	0.142467031	1.924922017
0.383846128	0.189937938	0.334829837	0.677644254	0.400229663	2.651483921
0.630513865	0.205356142	0.422405346	0.923702386	0.290558386	2.944861941
0.4309224	0.174415481	0.458883552	0.878674055	0.289995014	1.314122712
0.441810619	0.178463383	0.947632774	1.037060457	0.204801757	2.899290713
0.509339673	0.359608743	1.067917353	0.906701766	0.17496036	6.964404506

P23588,EIF4B,NEUROLOGY	P25774,CTSS,NEUROLOGY	P28906,CD34,NEUROLOGY	Q08431,MFGE8,NEUROLOGY	Q14739,LBR,NEUROLOGY	P01258,CALCA,NEUROLOGY
P23588	P25774	P28906	Q08431	Q14739	P01258
EIF4B	CTSS	CD34	MFGE8	LBR	CALCA
NEUROLOGY	NEUROLOGY	NEUROLOGY	NEUROLOGY	NEUROLOGY	NEUROLOGY
1.083275352	0.355297637	0.774855931	0.348154602	0.535329848	0.30367499
0.919550046	0.388126811	1.323446614	0.330334601	0.557715008	0.346709675
1.226374836	0.440373634	0.669056522	0.361959453	0.556710809	0.375764323
0.708578698	0.646624466	2.473064728	0.404440674	0.696164426	0.4984083
1.046157451	0.401062783	0.85104068	0.50754231	0.867177963	0.573394511
1.137368694	0.318110523	0.718818777	0.448412997	0.497028333	0.280991813
0.862442393	0.361157491	0.622782366	0.278297528	0.532997252	0.195128985
0.718619505	0.288771446	0.662365759	0.2059263	0.504840655	0.163289055
0.880320031	0.281615764	0.70828407	0.271928638	0.449938586	0.193459033
0.911933166	0.249342377	0.545518489	0.253929515	0.541712768	0.474144925
0.705980382	0.222287696	0.797579371	0.26866867	0.506207224	0.368695062
1.060540709	0.568960341	1.017832385	0.432748283	0.678960712	0.260905633
0.558063038	0.283201341	0.690637224	0.218681033	0.41116803	0.22939407
1.184271612	0.164299501	0.508422577	0.162645183	0.355864518	0.175579948
0.582689816	0.146584046	0.683067473	0.177095566	0.343242476	0.16868458
0.801569767	0.279070203	0.494896931	0.131971655	0.572243073	0.316746372
2.692600139	0.364679245	0.678490255	0.272324748	0.553632292	0.310206323
1.260153954	0.304138424	0.923766415	0.361182525	0.496752798	0.523840678
0.665495124	0.483537606	2.754136378	0.361483074	0.637280314	0.288171587
0.859994894	0.339362721	0.694044465	0.259157311	0.510010905	0.502188188
0.645818198	0.250728865	0.70637197	0.226314355	0.357843331	0.332010322
1.194577124	0.341013321	0.43615112	0.27177789	0.587027502	0.324772189
0.620670723	0.346589535	0.569907625	0.355519353	0.530417397	0.19249595
0.700084029	0.231550696	0.624987829	0.198884121	0.355889186	0.140798122
0.914528478	0.305490622	0.589392252	0.190994103	0.462876749	0.340658946
1.090961356	0.249100531	0.59234106	0.234344039	0.455776991	0.615700225
1.066585781	0.325696473	0.542426663	0.23023834	0.456757398	0.179443305
1.978353177	0.344792429	0.622437118	0.275170935	0.627157629	0.143826358
0.973194451	0.341036959	0.66840758	0.295309567	0.424518697	0.449845033
0.914274952	0.214879536	0.728701568	0.283535248	0.396914452	0.35963367
0.77689956	0.237516559	0.533995689	0.205883483	0.490729532	0.63437156
0.671193198	0.329716959	0.552673751	0.206970922	0.40410441	0.173992859
0.721114372	0.289753903	0.404132421	0.182478703	0.413827091	0.279903222
0.692218786	0.287931992	0.528142813	0.175093812	0.350333292	0.150924612
2.764272802	0.311931251	0.513060193	0.427649275	0.481464055	0.204291347

Q9UL46,PSME2,NEUROLOGY	Q9BS40,LXN,NEUROLOGY	P13473,LAMP2,NEUROLOGY	P35442,THBS2,NEUROLOGY	P48052,CPA2,NEUROLOGY	Q16881,TXNRD1,NEUROLOGY
Q9UL46	Q9BS40	P13473	P35442	P48052	Q16881
PSME2	LXN	LAMP2	THBS2	CPA2	TXNRD1
NEUROLOGY	NEUROLOGY	NEUROLOGY	NEUROLOGY	NEUROLOGY	NEUROLOGY
1.272442091	0.880320031	0.557715008	0.662503509	0.550494479	0.620799801
1.000069317	0.775930854	1.151249077	0.783442881	0.802626113	0.515734335
1.279251567	0.723668059	0.622480263	0.744167866	0.708578698	0.759962428
0.96279463	0.867057755	1.800752616	0.594397519	0.646355598	0.377906176
1.112727246	0.858208443	0.622868708	0.484980955	0.460955696	0.691883001
1.056651769	1.187148161	0.582689816	0.602402641	0.339456826	0.607602622
0.824905065	0.924727373	0.563348661	0.694862775	0.188625941	0.601026289
0.540662425	0.563661135	0.470087101	0.757438154	0.315104006	0.54329211
0.79526082	0.566873998	0.728095703	0.550570799	0.308512318	0.607097442
0.784149153	0.73820837	0.510258424	0.485754747	0.370668134	0.602653225
0.547981844	0.570263263	0.682972787	0.468298395	0.820855396	0.572243073
1.958297565	0.809835056	0.797579371	1.141317352	0.519105616	0.832429501
0.522100701	0.723617899	0.546729831	0.754922264	0.424577552	0.677409442
0.59828301	0.80068129	0.472766593	0.533366826	0.408299583	0.480230853
0.659342508	0.686294644	0.723567744	0.337884014	0.237302631	0.408809322
1.056871517	0.812759264	0.471784524	0.347407306	0.2906994	0.46532158
2.541887182	1.017127122	0.602319136	0.823648106	0.369872514	0.776522698
0.81966142	0.793498829	0.684536797	0.578945721	0.332378738	0.794159118
0.879466177	0.642692259	1.883609625	0.675533865	0.645012936	0.677926137
0.629858647	0.87751762	0.5253315	0.581197338	0.240698657	0.59139748
0.616939105	0.614676826	0.459456442	0.589760049	0.274618362	0.584550674
1.298728621	0.875330654	0.477509056	0.59357408	0.53950192	0.695923196
0.727944315	0.662044455	0.424165739	0.45952014	0.666603132	0.780840614
0.682688805	0.539053362	0.504211175	0.504525817	0.431101653	0.403852395
0.729965406	0.855179989	0.538978638	0.515948867	0.64175743	0.567857167
0.896701676	0.919996323	0.472111654	0.735144623	0.42445985	0.554515618
0.970230878	0.603949572	0.494314112	0.560583039	0.537635385	0.581036218
1.811267966	0.928388141	0.504420915	0.501944584	0.247259867	0.621531752
1.262164543	0.948224122	0.580875143	0.562490252	0.398844973	0.511533277
0.690350056	0.970163629	0.577983415	0.408667664	0.278027598	0.496511831
0.677127773	0.842413231	0.347166585	0.279012178	0.31822079	0.361432965
0.724521296	0.630339073	0.482499717	0.529168834	0.271796729	0.531742616
0.675393406	0.585564501	0.432568345	0.687484933	0.467358002	0.494725442
0.724973416	0.737594601	0.441902501	0.478635729	0.415350156	0.606508597
2.010145595	1.081549728	0.542802774	0.403153178	1.233279674	0.735654363

P28908,TNFRSF8,NEUROLOGY	Q14108,SCARB2,NEUROLOGY	P14778,IL1R1,NEUROLOGY	P08254,MMP3,NEUROLOGY	P52823,STC1,NEUROLOGY	Q9UBT3,DKK4,NEUROLOGY
P28908	Q14108	P14778	P08254	P52823	Q9UBT3
TNFRSF8	SCARB2	IL1R1	MMP3	STC1	DKK4
NEUROLOGY	NEUROLOGY	NEUROLOGY	NEUROLOGY	NEUROLOGY	NEUROLOGY
0.723517592	0.480230853	0.558333877	0.038080599	0.443928735	0.517560708
1.13713221	0.46664586	0.453130969	0.05335621	0.656651588	0.464838027
1.715108683	0.495995866	0.572481112	0.041254634	0.631563631	0.44319085
0.851807891	0.375582045	0.306933921	0.05205931	1.00751408	0.343123537
0.677503357	0.467682062	0.518889771	0.053020717	0.96828255	0.470543498
0.660852405	0.526936127	0.429788862	0.081763457	0.727944315	0.442853062
1.264003098	0.382147103	0.550036781	0.071456051	0.44590244	0.755079262
1.390110917	0.364881522	0.480930391	0.049005925	0.525185868	0.379428512
0.680468366	0.370873733	0.465095859	0.043242688	0.554554056	0.287473325
0.72865106	0.313383255	0.248893421	0.052647178	0.681270669	0.352916797
0.523405141	0.325448236	0.40584479	0.043812915	0.4399465	0.476946715
1.330713504	0.531042783	0.700181088	0.050702476	0.719616414	0.676283472
1.192674201	0.356926766	0.327325976	0.051900779	0.470739232	0.446892581
0.669010148	0.316987971	0.333810213	0.043155852	0.414114034	0.270293739
0.501875004	0.280136136	0.30834129	0.037126677	0.415206231	0.243146883
1.020375384	0.323828081	0.428717734	0.045657637	0.399121527	0.317515736
0.827998466	0.370539693	0.491682871	0.04793755	0.45350803	0.332793694
0.885460589	0.389015623	0.531263683	0.039002219	0.466710555	0.384351978
0.677268593	0.359683529	0.425844907	0.050720051	0.869766471	0.419778447
0.960594864	0.407564415	0.375321801	0.036696849	0.530086609	0.315038489
0.644566002	0.319591291	0.262556533	0.04143518	0.499376557	0.237072463
0.631694975	0.392074576	0.304961706	0.049703801	0.718569696	0.310356873
0.646355598	0.397988873	0.391178775	0.041357707	0.552061156	0.887119277
0.593450663	0.315191383	0.323155399	0.065244495	0.352330189	0.489710149
0.985207831	0.413282448	0.348758431	0.045952909	0.44714046	0.461787171
0.769183649	0.374204808	0.504770674	0.051392441	0.30510971	0.412338192
0.748098426	0.369104185	0.489370825	0.062305356	0.369001862	0.360207469
0.789603341	0.329648404	0.399342909	0.039857637	0.310916697	0.34596548
1.243580586	0.371594229	0.540812349	0.065194768	0.536704541	0.53211132
0.387965427	0.365666407	0.30587201	0.049463221	0.399869182	0.474539472
0.414947293	0.354215684	0.395102807	0.115143111	0.786544339	0.43615112
1.096267597	0.38958229	0.526534511	0.048207452	0.482834276	0.355790526
0.815863667	0.322528823	0.34035212	0.075289892	0.234604079	0.401535654
0.890137431	0.349363308	0.428509769	0.05350435	0.561282895	0.339527421
0.957470538	0.338352745	0.443990281	0.04470562	0.507718242	0.339151082

Q9UKJ1,PILRA,NEUROLOGY	P09466,PAEP,NEUROLOGY	Q53H47,SETMAR,NEUROLOGY	P15311,EZR,NEUROLOGY	P28325,CST5,NEUROLOGY	P04233,CD74,NEUROLOGY
Q9UKJ1	P09466	Q53H47	P15311	P28325	P04233
PILRA	PAEP	SETMAR	EZR	CST5	CD74
NEUROLOGY	NEUROLOGY	NEUROLOGY	NEUROLOGY	NEUROLOGY	NEUROLOGY
0.410968579	0.569197015	0.45640927	0.791356681	0.207732666	0.36912977
0.459743155	0.556672222	0.447016503	0.937571096	0.235369616	0.521919786
0.555169417	0.604326453	0.456156253	0.821026105	0.348492618	0.536927797
0.465870215	1.148379912	0.406520497	1.473758363	0.448040173	0.976234742
0.409206226	0.641579522	0.543631139	0.918403471	0.275954057	0.544121221
0.331665303	1.009331441	0.520871715	0.872786081	0.603196516	0.405394944
0.404693056	0.532775631	0.337860594	0.724872921	0.184347531	0.430593964
0.557058212	0.393681275	0.382836423	0.728701568	0.218969221	0.333463324
0.317581769	0.509551546	0.367343079	0.664895722	0.328121037	0.323536414
0.377670499	0.59857337	0.292904041	0.825877665	0.215013627	0.315082165
0.212318207	0.719317197	0.424077545	0.929611615	0.208193943	0.339315679
0.548513867	0.567896529	0.759067454	0.948750075	0.689489267	0.543668822
0.272740339	0.677409442	0.408780987	0.85577296	0.282084637	0.308704838
0.270031572	0.569789128	0.331849269	0.677926137	0.376624824	0.258117525
0.171252862	0.554054577	0.214492633	0.761438805	0.283554902	0.228157186
0.146360687	0.622437118	0.327257917	0.842354841	0.246643641	0.571965487
0.42398937	0.349387524	0.435637484	0.849802799	0.558295178	0.29617053
0.374101071	0.507507131	0.473356816	0.772228658	0.394500764	0.301870148
0.425372891	0.477012838	0.537560859	1.398906947	0.334481889	0.751737042
0.344171609	0.467390398	0.374594079	0.739950157	0.586702075	0.420710579
0.43612089	0.483571123	0.396831925	0.757700707	0.206455105	0.270069009
0.309240247	0.616896343	0.442699608	0.898256889	0.200781743	0.329899844
0.291284331	0.637810609	0.394829037	0.681979368	0.301514649	0.315388071
0.258117525	0.499792099	0.277854209	0.737441238	0.33980995	0.211027073
0.465611953	0.522317881	0.442730294	0.741798883	0.579066122	0.392618484
0.192562676	0.449035061	0.39937059	0.520258307	0.442423523	0.303443537
0.475725083	0.466031701	0.279341146	0.671146676	0.190703073	0.377173442
0.38969032	0.486226355	0.342695701	0.826450318	0.278934831	0.283751515
0.22481341	0.702952933	0.368950711	0.859398998	0.255731184	0.427027238
0.25371839	0.652115764	0.373117	0.768810529	0.260869467	0.340328529
0.344028502	0.341013321	0.228014899	0.755340998	0.225359473	0.453979797
0.194144131	0.588494159	0.316680513	0.837929052	0.374957764	0.336388432
0.330449107	0.502362263	0.392047401	0.635912424	0.60638249	0.257206677
0.280194395	0.567935894	0.330426202	0.798519752	0.495274415	0.30266631
0.30729581	0.472340779	0.325470795	0.615572207	0.441933132	0.312407285

P40222,TXLNA,NEUROLOGY	Q02790,FKBP4,NEUROLOGY	Q6P1M0,SLC27A4,NEUROLOGY	Q9NZQ7,CD274,NEUROLOGY	Q9NXA8,SIRT5,NEUROLOGY	Q8NBJ7,SUMF2,NEUROLOGY
P40222	Q02790	Q6P1M0	Q9NZQ7	Q9NXA8	Q8NBJ7
TXLNA	FKBP4	SLC27A4	CD274	SIRT5	SUMF2
NEUROLOGY	NEUROLOGY	NEUROLOGY	NEUROLOGY	NEUROLOGY	NEUROLOGY
0.897696702	0.463037197	0.336878439	0.429610155	0.75912007	0.469240681
0.7850737	0.407903559	0.9507909	0.56097174	0.991304373	0.724571517
0.783823102	0.628288901	0.847097537	0.532369564	0.804241116	0.336108749
0.517524834	0.511675124	0.916114609	0.390285021	0.859279868	0.468785548
0.954951918	0.789767552	0.465708784	0.585077645	1.18846548	0.397906122
0.752258287	0.695007282	0.638075922	0.532627934	0.826335756	0.420885584
0.785454712	0.514555999	0.863399403	0.534328917	0.852162222	0.439458854
0.571093948	0.353161505	0.541074817	0.470739232	0.855891604	0.484980955
0.574747424	0.313774496	0.591151575	0.397933703	0.937246215	0.295104945
0.717623978	0.340706175	0.525877982	0.354731659	0.897074682	0.194063405
0.718968266	0.383979182	0.740822592	0.360757177	0.980643049	0.27789273
1.188383105	0.90463016	0.904065998	0.542125961	3.086346413	0.568369089
0.712321282	0.311888011	1.255620075	0.34687794	0.816089904	0.422258977
0.554285049	0.365185149	0.735654363	0.278471193	0.880381052	0.350357576
0.370950863	0.282985493	0.85340354	0.327325976	0.891063406	0.176911532
0.865496566	0.392428031	0.508352099	0.366859614	1.036916699	0.224299763
0.999584198	0.960927838	1.11211039	0.407507919	0.788345528	0.599071456
0.772228658	0.386515989	0.672776864	0.284184544	0.863339559	0.244719336
0.812421317	0.441565695	0.407169104	0.442515532	0.986301067	0.796088099
0.771051966	0.306360032	0.491682871	0.403041416	0.823762296	0.381432585
0.587923357	0.318331096	0.778732639	0.333787076	1.042032302	0.247225592
1.025196174	0.676846222	0.917957967	0.370873733	0.699114179	0.320545264
0.591766528	0.35030901	1.059071506	0.288791463	0.678208138	0.302226066
0.568920905	0.265061676	0.734533401	0.443283018	0.988833825	0.334505074
0.83225642	0.379823217	0.625551254	0.453068156	0.760278553	0.272702532
0.609416303	0.334621025	0.727036651	0.377121158	0.952704032	0.268222097
0.624468195	0.276988887	0.76212524	0.377304183	0.849272829	0.453162379
1.033186	0.617623692	0.950659101	0.355149904	0.89688816	0.24961906
0.742364692	0.405619805	0.541074817	0.476120945	0.96513322	0.423020651
0.729156297	0.301326613	0.774909642	0.368976285	0.97481476	0.517991382
0.550990748	0.316812244	0.495892737	0.500346694	0.744167866	0.572044783
0.530932367	0.338282394	0.376050938	0.373168729	0.662182138	0.628550254
0.581882596	0.39280903	0.513309192	0.284401307	0.969693017	0.491103837
0.589514825	0.216629256	0.905257419	0.405310653	0.892485107	0.300700673
1.202385903	0.808320869	0.522861229	0.312927423	1.08259978	0.623646324

P42892,ECE1,NEUROLOGY	P57087,JAM2,NEUROLOGY	O75509,TNFRSF21,NEUROLOGY	Q9UPY6,WASF3,NEUROLOGY	Q9Y5P4,CERT,NEUROLOGY	P22223,CDH3,NEUROLOGY
P42892	P57087	O75509	Q9UPY6	Q9Y5P4	P22223
ECE1	JAM2	TNFRSF21	WASF3	CERT	CDH3
NEUROLOGY	NEUROLOGY	NEUROLOGY	NEUROLOGY	NEUROLOGY	NEUROLOGY
0.504665721	0.674457761	0.5163782	1.292532001	1.081100018	0.449533333
0.903376945	0.871335362	0.711482413	1.577128269	1.246860445	0.828228068
0.488083538	0.731941372	0.579066122	0.872241779	1.421584642	0.560466481
0.674457761	0.643137893	0.583174685	1.027187819	0.540699902	0.960461706
0.764718139	0.760278553	0.533145051	1.048407806	1.580192151	0.404552824
0.334690614	0.651889797	0.476517136	0.669474031	1.218072495	0.546616154
0.584307616	0.549274796	0.423137954	0.460380936	1.083125188	0.56703119
0.568093381	0.720964436	0.523151245	1.329699273	0.669566847	0.645997282
0.408639339	0.399425958	0.328394074	1.824625013	0.649544377	0.460412849
0.42788648	0.369590608	0.280777649	0.936856507	1.139894259	0.347985718
0.52624262	0.638341346	0.489506526	1.83961007	1.682725673	0.525695759
0.805468458	1.263565104	0.77335354	0.960128893	0.772068094	0.741953151
0.478702087	0.649589402	0.376677039	0.533071146	1.061423209	0.424136339
0.794104073	0.505786347	0.321881151	0.564912763	1.155726478	0.414889773
0.581197338	0.360857214	0.352721152	1.620006947	0.396694417	0.328849641
0.509834179	0.363619128	0.301451957	1.29477373	0.89180488	0.326895178
0.53155836	0.56003931	0.52624262	0.840255508	1.437438268	0.480131002
0.479731807	0.601026289	0.395788061	1.942480344	0.756651039	0.462908834
0.61429349	0.65492427	0.486630955	1.983570947	1.18468212	0.948158399
0.898319154	0.558101721	0.443528895	0.652251382	1.890280064	0.471196262
0.42658348	0.587801115	0.423578128	0.970836329	0.932709683	0.42717526
0.416330166	0.571054364	0.337112026	1.095432053	1.035623782	0.363291622
0.473619373	0.656833675	0.405507359	1.682142586	2.175127545	0.376755375
0.536704541	0.437999158	0.319702072	1.055261097	1.336722503	0.380244688
0.507964648	0.71717644	0.437483345	2.316103942	0.657425809	0.385900281
1.163039423	0.593697523	0.403572563	1.333760843	1.280227319	0.34077703
0.698726615	0.589147181	0.448848351	1.019809724	0.992266808	0.548285794
0.538194667	0.618051944	0.380534721	1.591734814	1.510577287	0.3672667
0.762072415	0.701152418	0.49986139	2.088547565	1.667399722	0.428747451
0.514306396	0.466193243	0.437938442	2.051671849	1.496819232	0.357124743
0.192709554	0.469631147	0.333139886	0.863219883	1.339783602	0.510683021
0.439458854	0.677926137	0.589596555	0.220293678	1.100988958	0.530711604
0.407394949	0.41142461	0.286220716	0.495961487	1.355758298	0.388799967
0.360257407	0.431071773	0.368567304	0.921975296	1.249109541	0.47384923
0.76530143	0.466839972	0.454011265	0.864896857	1.212092667	0.391097441

P04216,THY1,NEUROLOGY	Q15633,TARBP2,NEUROLOGY	P49789,FHIT,NEUROLOGY	P21757,MSR1,NEUROLOGY	Q9UKV5,AMFR,NEUROLOGY	P12081,HARS,NEUROLOGY
P04216	Q15633	P49789	P21757	Q9UKV5	P12081
THY1	TARBP2	FHIT	MSR1	AMFR	HARS
NEUROLOGY	NEUROLOGY	NEUROLOGY	NEUROLOGY	NEUROLOGY	NEUROLOGY
0.588494159	0.578504465	0.655696455	0.26533741	0.534958914	0.839498702
0.932515752	1.045577499	0.971240172	0.398789685	1.013467623	0.744580635
0.531963808	0.661494011	0.753197439	0.353528885	0.513843168	1.075643496
1.102287073	0.871395761	0.961127679	0.482232236	0.699453474	0.737492355
0.660486052	0.914528478	1.030682513	0.419284092	1.111185748	0.825705946
0.488185043	0.333417099	1.006606626	0.180729009	0.654561203	0.833699861
0.453445164	0.794049032	0.958998192	0.284697159	0.775124522	0.967947027
0.6042008	1.051172909	0.623689554	0.220813457	0.987737781	0.802347993
0.434943527	0.675112577	0.95019795	0.319569139	0.840721574	0.780407745
0.368286393	0.607855369	0.923574343	0.140671308	1.011081996	0.65533296
0.498270131	1.247292648	0.960994447	0.242037082	0.736215485	0.787690073
0.979759798	0.60344743	1.289310728	0.409177863	0.745561878	0.857019538
0.491410299	0.836072525	0.704562698	0.159928615	0.654969667	0.626983769
0.45081268	0.84077985	0.990136957	0.087389272	1.46886316	0.712420038
0.325561047	0.661402315	1.22603486	0.163074148	0.704123306	0.712716387
0.335712928	1.07750906	1.112264572	0.213395255	0.520402573	0.820855396
0.498684753	0.673850287	1.986322667	0.293819082	0.641712949	0.882152509
0.44664484	0.753980961	1.151648139	0.357719334	2.235658761	0.908148418
1.0428994	1.036557394	0.890260839	0.383872735	0.814112457	0.927680551
0.545858908	0.932192622	1.120855268	0.165786679	0.662503509	0.752623374
0.539090727	0.893971039	1.141554707	0.229855645	1.027615104	0.791466394
0.516199268	0.404665006	1.464796247	0.651392945	1.252143578	0.868019886
0.437634992	0.735195581	0.789931796	0.395842933	0.631476084	0.771693575
0.363921704	0.642781361	0.383261238	0.138821218	1.016563263	0.697468516
0.47853621	0.782845763	1.462057448	0.299639557	1.028042566	0.7622309
0.446366295	0.727692073	1.034117415	0.266664928	0.923446317	0.730724761
0.561827832	0.927102014	0.99633306	0.252158017	0.558411284	0.962260891
0.472177107	0.485350876	1.439432363	0.249014214	0.517202086	0.821196851
0.639359826	0.988833825	1.073260286	0.300950892	1.000762753	1.302695591
0.599653081	0.934133079	0.352183689	0.358687656	0.646086842	0.847743663
0.402092686	0.562139462	0.276739407	0.172073877	0.538455864	0.622739199
0.455776991	0.823362701	0.703245344	0.297034003	0.551525693	0.683446351
0.347479555	1.133983776	0.654152994	0.187102404	0.963929808	0.728196645
0.398651499	0.575345312	0.985207831	0.204220557	0.731992108	0.940109037
0.416792147	0.974476973	1.113730367	0.284756366	0.751059963	0.895521516

Q15155,NOMO1,NEUROLOGY	Q9UBW5,BIN2,NEUROLOGY	P05231,IL6,NEUROLOGY	P23381,WARS,NEUROLOGY	O00559,EBAG9,NEUROLOGY	P11464,PSG1,NEUROLOGY
Q15155	Q9UBW5	P05231	P23381	O00559	P11464
NOMO1	BIN2	IL6	WARS	EBAG9	PSG1
NEUROLOGY	NEUROLOGY	NEUROLOGY	NEUROLOGY	NEUROLOGY	NEUROLOGY
0.365970686	0.845748137	0.604703569	0.533847654	0.855120715	0.051470871
0.412395358	0.799793799	0.474802685	0.396859432	0.636265146	0.090420895
0.413311096	0.942392524	0.807257027	0.482399394	0.68619951	0.071555179
0.286896048	0.794709778	0.76742624	0.732753572	0.627375023	0.050108529
0.404721108	1.056505297	1.46094311	0.506804065	1.154525469	0.083158065
0.337649892	0.847567398	0.811014717	0.474473691	1.022782939	0.09383045
0.349000256	0.943896121	0.463069294	0.372729264	0.705100105	0.069007645
0.325448236	0.853640186	0.335899138	0.494554013	0.543631139	0.053459865
0.316636615	1.163442572	0.454735645	0.421878655	0.628986082	0.050656809
0.240398533	1.186325577	0.83613048	0.402510968	0.602945705	0.035854549
0.296375891	1.121787957	0.434431313	0.58394322	0.522317881	0.049879818
0.41371237	0.79680577	1.323079728	0.618651997	0.922806455	0.053105312
0.314994818	0.738617833	0.526315577	0.334621025	0.529976392	0.049921324
0.282260665	0.946976152	0.325899716	0.348782606	0.48333655	0.027942405
0.179816835	1.033257618	0.422229709	0.292680797	0.510789226	0.03794622
0.343694817	0.779866995	0.383473822	0.564012875	0.894714933	0.038099081
0.233938303	0.823077395	0.532923368	0.673336697	0.957138761	0.087856936
0.361232599	1.350037985	1.182303151	0.542878028	0.60886741	0.074935856
0.382438587	1.063043033	1.884262548	0.582851394	0.614549021	0.052705598
0.232274068	0.75612675	0.636353357	0.351038215	0.782791502	0.024077009
0.264602757	1.057897609	1.04131027	0.498097474	0.465611953	0.03847593
0.378037171	0.68164855	1.282358818	0.65788166	0.751841262	0.018051969
0.352550052	0.922103118	0.545934585	0.566952589	0.466710555	0.037652775
0.198250993	1.150610866	0.365058607	0.255341511	0.469696256	0.067311649
0.243467313	1.034404173	0.414286295	0.676283472	0.565931757	0.017809613
0.332447861	0.796198468	0.391775748	0.481230504	0.614463832	0.058756815
0.379191886	0.720514815	0.459711289	0.352208102	0.816429377	0.096789878
0.344052349	0.752206146	0.444760324	0.459838766	0.73693026	0.05335621
0.449969774	0.954422527	0.604033303	0.415321367	0.647656162	0.058108781
0.267906223	0.776845711	0.862203306	0.396749415	0.998753113	0.057063023
0.317846036	0.871999976	0.305914416	0.238737979	0.575305433	0.047765077
0.332217505	0.934133079	0.513202463	0.409489964	0.632790568	0.060274851
0.282946265	0.769770346	0.322082013	0.365514362	0.483705216	0.076855909
0.296663636	1.10343374	0.313470155	0.344171609	0.760963943	0.05844
0.379297035	0.551334582	0.388611365	0.4768806	0.782682992	0.074899505

O43557,TNFSF14,NEUROLOGY	O95727,CRTAM,NEUROLOGY	O94779,CNTN5,NEUROLOGY	P14868,DARS,NEUROLOGY	Q8NBS9,TXNDC5,NEUROLOGY	P60484,PTEN,NEUROLOGY
O43557	O95727	O94779	P14868	Q8NBS9	P60484
TNFSF14	CRTAM	CNTN5	DARS	TXNDC5	PTEN
NEUROLOGY	NEUROLOGY	NEUROLOGY	NEUROLOGY	NEUROLOGY	NEUROLOGY
0.527374602	0.419400359	1.020941357	0.962194195	0.53998828	0.749967509
0.534810613	0.556170836	0.959796196	0.749447851	0.738310714	0.842822071
0.413855777	0.543216799	1.215289478	1.095735813	0.562997336	1.111493876
0.531595206	0.56848729	0.891001645	0.940891323	1.56092368	0.599486845
0.527593977	0.451939011	1.051318643	0.875816175	0.499584285	1.176906737
0.40983071	0.620240656	0.367012219	1.072888387	0.68491649	0.907393355
0.550303725	0.383846128	0.517273791	1.295042999	0.672963424	0.879100493
0.327348665	0.499238119	0.636132852	1.004376376	0.505050656	0.770304095
0.460349026	0.471261588	0.77819305	1.058117616	0.471915349	1.00291546
0.342743212	0.62772301	0.71976607	1.106880902	0.464097554	0.903940676
0.482399394	0.404693056	0.77335354	0.968215436	0.688772764	0.805189353
0.72597914	0.78583591	1.438734115	1.394743666	0.588616545	1.219762273
0.435335628	0.387186351	1.044490956	0.955415376	0.59020989	1.286632468
0.257278	0.398762044	0.574986504	1.034189097	0.555823986	0.853521855
0.329785529	0.239749548	0.311218559	1.342386396	0.462011286	0.789384447
0.607644739	0.382544636	0.416561092	1.221369734	0.473389627	1.264353603
0.744529026	0.490899635	0.49120597	1.421092043	0.536332655	0.902125466
0.321502085	0.396666922	1.174869079	0.966539099	0.726180452	1.104887897
0.553248677	0.417428208	0.748461493	0.901187995	1.117751901	0.9151626
0.416705486	0.557444469	1.125136471	1.560382799	0.585889297	0.542351472
0.380877772	0.221533994	1.187477354	1.166511071	0.488218883	0.933679945
0.666233591	0.346133384	0.640646313	0.995918783	0.520655135	1.00291546
0.392591271	0.443774907	0.662044455	0.929353908	0.481430684	0.90450476
0.289392613	0.235990387	0.457676461	1.184600007	0.418239144	0.883070178
0.38858443	0.753197439	0.973059547	1.525624588	0.581116772	0.927423379
0.322082013	0.277738677	0.585848688	1.444830246	0.374983755	0.871818667
0.434732543	0.510081612	0.468753056	0.919996323	0.484074163	0.877578447
0.476682313	0.266535573	0.630601278	2.031717758	0.452942556	0.836942259
0.446211623	0.424930852	0.926202785	0.994125571	0.415235012	1.084327079
0.348855141	0.510470678	0.567267061	1.210245727	0.581963267	0.959397111
0.360507205	0.397685537	0.397768242	0.822678132	0.5214497	0.799073436
0.320700832	0.351890873	0.577262735	0.853581019	0.558953433	0.974747193
0.397988873	0.320145581	0.419080704	1.026760713	0.414171446	0.874906044
0.382624192	0.417399275	0.454231606	0.960461706	0.394172764	0.892670713
0.557251307	0.586295545	0.449097314	1.484214905	0.565304466	0.811014717

P29218,IMPA1,NEUROLOGY	O14737,PDCD5,NEUROLOGY	P29474,NOS3,NEUROLOGY	P53985,SLC16A1,NEUROLOGY	P30740,SERPINB1,NEUROLOGY	P14174,MIF,NEUROLOGY
P29218	O14737	P29474	P53985	P30740	P14174
IMPA1	PDCD5	NOS3	SLC16A1	SERPINB1	MIF
NEUROLOGY	NEUROLOGY	NEUROLOGY	NEUROLOGY	NEUROLOGY	NEUROLOGY
0.77781556	0.552635444	1.060834794	0.629465843	0.561983625	0.782845763
0.959663149	0.532443372	1.815667464	0.499065126	0.510506063	0.615742903
0.981391037	0.509975555	1.467641902	0.50785903	0.621187197	0.745820315
0.848155091	0.8400808	2.111400117	0.439093475	0.497131698	0.697323497
1.344807802	0.654515834	2.647810731	0.736419636	0.62772301	0.748202141
1.010661586	0.760963943	1.88334852	0.337696703	0.543405096	0.634327591
0.662779093	0.396254713	2.138204736	0.421060661	0.397988873	0.843932782
0.760805721	0.250537767	1.565257485	0.499722818	0.483236053	0.558798479
0.735297508	0.366351391	1.778917987	0.479034013	0.45546118	0.661264794
0.713408347	0.51544843	1.491744027	0.618309038	0.416821038	0.708136802
0.657653695	0.562919293	1.62394188	0.445315582	0.400118711	0.660394495
0.99819944	1.149176185	1.377927517	0.434371093	0.746285727	1.449243485
0.705491204	0.357372369	1.706452196	0.43612089	0.411025555	0.482432833
0.587801115	0.454830214	1.385589594	0.611065951	0.408611015	0.733464985
0.665818102	0.521521993	1.050444544	0.486192654	0.408327885	0.5749068
0.721664404	0.546085971	1.299539062	0.698000512	0.527264949	0.813097351
0.675908564	0.777276606	1.043694875	0.679290225	0.631213515	1.874752309
0.876301966	0.537188379	1.593390634	0.93212801	0.631957744	0.69260274
0.738157203	0.777438253	2.422001905	0.39680442	0.521269011	0.62994597
0.709610863	0.657927263	1.067843333	0.581680965	0.411881148	0.49875389
0.661310631	0.340493698	1.631161919	0.495823996	0.462427788	0.53067482
0.810396585	0.625161136	2.47546577	0.740668558	0.634503488	0.942000676
0.68249955	0.568290302	1.690910149	0.352476749	0.625594615	0.452127006
0.661035657	0.338399654	0.880259014	0.459806893	0.438850057	0.451782408
0.827539453	0.360357306	1.596707444	0.463904582	0.493595108	0.611913656
0.850215226	0.565853307	1.493295831	0.890075733	0.532849494	0.885767519
0.909345219	0.377095019	1.923721562	0.395705768	0.379113044	0.77716886
0.857078944	0.822222069	1.960334706	0.481330584	0.565226104	1.507857403
0.768863821	0.40745143	1.292263255	0.471751824	0.567188427	0.938871747
1.067843333	0.440129507	1.355570363	0.563231528	0.352721152	0.478337234
0.760963943	0.415580538	0.943699864	0.631432315	0.41402793	0.538045468
0.591192552	0.47853621	1.308850211	0.445624359	0.416821038	0.562217396
0.556595056	0.57271925	1.323813602	0.46771448	0.446335357	0.624078753
0.634503488	0.405479252	1.406198246	0.498442849	0.579226695	0.506804065
0.602235643	0.955216724	3.462466229	0.627679502	0.568329694	1.638414059

P55291,CDH15,NEUROLOGY	P13611,VCAN,NEUROLOGY	P01138,NGF,NEUROLOGY	P18031,PTPN1,NEUROLOGY	Q86T13,CLEC14A,NEUROLOGY	P08134,RHOC,NEUROLOGY
P55291	P13611	P01138	P18031	Q86T13	P08134
CDH15	VCAN	NGF	PTPN1	CLEC14A	RHOC
NEUROLOGY	NEUROLOGY	NEUROLOGY	NEUROLOGY	NEUROLOGY	NEUROLOGY
0.607265789	0.635736136	1.076314727	1.04891662	0.621402521	0.60869862
1.052047614	1.16191135	1.148300315	1.069472955	0.861426732	0.460157612
0.96761162	0.797911144	1.165056564	1.057824284	0.706127202	0.585402171
1.022924736	1.869950396	1.032183877	0.87575547	0.510860041	0.450656467
0.625507895	0.694092575	0.944485138	1.23387821	0.66595657	0.818639395
0.453256621	0.623430222	1.194328745	0.933550518	0.667204073	0.60634046
0.555092459	0.917067607	1.185174918	0.724119647	0.486765897	0.56178889
0.806138707	0.758278646	1.146868515	0.7031966	0.605206757	0.521341279
0.399342909	0.948026965	1.005281818	0.764400168	0.441351498	0.500277336
0.454326071	0.690350056	1.025338306	0.903063912	0.377251881	0.328576225
0.454326071	0.676236597	1.08809159	0.792509426	0.533736656	0.397850964
1.567320255	1.119069781	1.043116288	1.090810128	1.048117166	0.910101905
0.80865711	0.671937988	1.087789948	0.811239609	0.530748392	0.473061612
0.495514782	0.459902517	0.9885597	0.663376587	0.555130937	0.339574493
0.537672653	0.652929894	1.028470206	0.563973781	0.287333876	0.316922062
0.378981676	0.518745924	1.012835586	0.807033239	0.440373634	0.482767345
0.594397519	0.808040775	1.053726166	0.882947767	0.75351075	0.579146403
0.584307616	0.73882265	1.01494391	0.957470538	0.606802948	0.470641355
1.371923464	1.495678399	1.028042566	0.709463319	0.581277915	0.461243345
0.741541839	0.59238212	1.034117415	0.844576494	0.570342323	0.367903677
0.623170999	0.596833326	1.064739129	1.085154152	0.441657526	0.462011286
0.577462833	0.621230256	1.07601635	0.78181545	0.523223774	0.635692071
0.776468875	0.574588092	1.041743429	0.752258287	0.584793832	0.458724542
0.295473367	0.557676352	1.032183877	0.802737389	0.52365916	0.298727094
0.549503281	0.695682049	1.113730367	0.978538148	0.692170807	0.452942556
0.440648439	0.729358491	1.079527504	0.817165386	0.532664855	0.370026372
0.417746602	0.612295506	0.979759798	0.90884111	0.489031737	0.345031504
0.621273318	0.623559874	0.970903624	0.888288365	0.562568235	0.47883483
0.70383053	0.862322841	0.955216724	1.024769896	0.581680965	0.422405346
0.376520416	0.76212524	1.267073312	1.506917046	0.393081399	0.451719782
0.303422505	0.457486159	1.106420659	0.825877665	0.436090661	0.294777844
0.798851916	0.657243557	0.987600861	0.798187726	0.58192293	0.415465331
0.446830633	0.508739846	1.017550221	0.848037519	0.427530722	0.370899441
0.426524347	0.429967643	1.046592627	0.93809114	0.522824988	0.386194628
0.462395736	0.698968817	1.05110005	0.965735489	0.509657515	0.478038924

Q8NFP4,MDGA1,NEUROLOGY	Q6PIL6,KCNIP4,NEUROLOGY	Q04900,CD164,NEUROLOGY	P15509,CSF2RA,NEUROLOGY	Q96NZ8,WFIKKN1,NEUROLOGY	Q9H1C3,GLT8D2,NEUROLOGY
Q8NFP4	Q6PIL6	Q04900	P15509	Q96NZ8	Q9H1C3
MDGA1	KCNIP4	CD164	CSF2RA	WFIKKN1	GLT8D2
NEUROLOGY	NEUROLOGY	NEUROLOGY	NEUROLOGY	NEUROLOGY	NEUROLOGY
0.384751805	1.165702788	0.633141558	0.615614876	0.364729804	0.802570482
0.260039015	1.069028266	0.621704101	0.6596625	1.008352455	1.053142018
0.332839832	0.899939549	0.705735751	0.529425651	0.546654043	0.935299292
0.259103427	0.88779593	0.593039457	0.317361714	0.517776	0.655923741
0.559030925	1.605697359	0.61459162	0.890939887	0.579266846	0.890816385
0.663008835	0.690541488	0.752988637	0.78605382	0.532775631	0.997576925
0.490593492	1.035193167	0.627897076	0.74046323	0.652658404	0.816768991
0.17783362	1.130765679	0.680091139	0.930062775	0.585402171	1.705033399
0.380719402	0.804129633	0.466290195	0.464580337	0.700958044	0.640779545
0.269377265	0.972520118	0.515662844	0.465418351	0.277238592	0.665448997
0.526899604	0.744374222	0.627635996	0.455114041	0.407903559	1.020870593
0.333879634	1.144248182	0.745458529	0.50375704	0.549503281	0.975558302
0.157366461	1.357827303	0.605794338	0.429312475	0.23411674	0.987121791
0.26751654	0.964665048	0.569118113	0.199408664	0.438728399	0.83497216
0.254722799	1.075941769	0.46557968	0.469208157	0.323670997	0.918148871
0.230749592	0.972048363	0.516199268	0.292214566	0.535738173	0.828457733
0.707646129	1.00793318	0.622307699	0.574229757	0.335899138	0.928130773
0.520186189	1.773132073	0.897385638	1.100531165	0.454326071	2.177541177
0.400396148	1.029254674	0.55632506	0.785563607	0.568369089	2.937115501
0.462491899	1.803000754	0.567857167	0.800736791	0.834277938	1.042176768
0.337626488	1.139420288	0.608361179	0.437453022	0.461307291	0.91376811
0.313600551	0.910732959	0.569315388	0.764294207	0.447667658	0.644655364
0.358712519	1.028684092	0.616169852	0.478270927	0.36721579	0.687770909
0.362009635	1.015718061	0.543028567	0.448350838	0.598946896	1.022286802
0.471130945	0.98951947	0.61796627	0.564795305	0.586092386	1.213437666
0.735807354	1.645584374	0.642959602	0.560738487	0.664204775	0.843289561
0.181608043	0.991785474	0.577342766	0.520150133	0.584955994	0.97874165
0.466290195	1.22357285	0.543405096	0.383872735	0.334226956	0.97326191
0.219425029	0.96346222	0.632790568	0.585118201	0.613952948	2.272846102
0.566324167	1.298188607	0.529719308	0.821823221	0.593532938	1.001109651
0.209380646	0.695296388	0.466839972	0.348927691	0.571014783	0.726633608
0.297672943	0.90789666	0.552214241	0.548475849	0.590905773	0.65397165
0.241768803	0.976031761	0.474605262	0.538157363	0.422024892	0.779272602
0.471130945	2.123287897	0.578143688	0.599944105	0.531890067	1.049207481
0.176152884	1.023421183	0.553555548	0.281967346	0.461947242	0.854824404

P22079,LPO,NEUROLOGY	Q9NSK7,C19orf12,NEUROLOGY	Q99972,MYOC,NEUROLOGY	Q9BZM5,ULBP2,NEUROLOGY	P78333,GPC5,NEUROLOGY	P00995,SPINK1,NEUROLOGY
P22079	Q9NSK7	Q99972	Q9BZM5	P78333	P00995
LPO	C19orf12	MYOC	ULBP2	GPC5	SPINK1
NEUROLOGY	NEUROLOGY	NEUROLOGY	NEUROLOGY	NEUROLOGY	NEUROLOGY
0.864057963	0.263870139	0.223059423	0.953761201	0.564873607	0.440953979
0.834856416	0.228220454	0.134791367	0.601985232	0.495377415	0.369872514
0.531005975	0.261484983	0.196241218	0.902876145	0.264749525	0.442055679
0.726633608	0.30855509	0.111699916	0.60886741	0.633404929	0.373505139
1.108954367	0.829549514	0.135137502	1.112573	0.971442157	0.447481517
1.018114627	0.275629078	0.114149781	0.896515232	0.65492427	0.50930437
0.833295446	0.27115694	0.276030578	0.665033997	0.525440751	0.395897812
0.722264917	0.328325794	0.20517118	0.761597158	0.394063491	0.385713087
0.766203754	0.212907695	0.324682155	0.547867907	0.342434508	0.318154625
0.69399636	0.330769932	0.094876855	0.679101912	0.352965725	0.383633337
0.512562558	0.260471964	0.183162999	0.670681634	0.499618914	0.470184863
0.972520118	0.228996904	0.217245771	1.210581324	0.710792321	0.459902517
0.612550206	0.300971753	0.152946606	0.636706325	0.696792017	0.342481983
0.472996037	0.331642315	0.107001283	0.701979108	0.371903441	0.283869548
0.632395935	0.251686545	0.020170631	0.51192345	0.389015623	0.337837177
0.573553512	0.240898948	0.100907128	0.474769776	0.394692224	0.361057371
1.067473311	0.633492744	0.192709554	0.536592948	0.988285652	0.413769727
1.234305914	0.665264521	0.158768873	0.632220622	0.440556818	0.457708186
1.01931503	0.300846609	0.168252518	0.627331539	0.673616788	0.392781803
0.393435761	0.369949435	0.085679864	0.729965406	0.344243185	0.346974129
1.04441856	0.313470155	0.115071303	0.567149113	0.310744336	0.324749678
0.560427634	0.344816329	0.109819032	0.761808347	0.301786463	0.308983135
0.714992493	0.258010199	0.209235565	0.493287283	0.227367822	0.427856822
0.569947129	0.337392545	0.1241882	0.511533277	0.362687769	0.349557089
1.02002181	0.261430614	0.120032751	0.756283999	0.535886731	0.426968043
1.00542119	0.352330189	0.156452867	0.688009314	0.435395982	0.41879032
0.620885868	0.307572836	0.128451888	0.360907243	0.384671806	0.385900281
0.853699358	0.216359144	0.158945051	0.668453912	0.490015741	0.359434302
1.372589287	0.260399756	0.385980535	0.735960377	0.555978115	0.480397317
0.278201094	0.247740218	0.188103683	0.525477173	0.425107613	0.423167284
0.554092982	0.266997844	0.166016668	0.539090727	0.830067175	0.499341944
1.033400868	0.250815776	0.185822873	0.794269219	0.257206677	0.365185149
1.034404173	0.295064037	0.176617475	0.576662855	0.275438092	0.323155399
1.628337779	0.2731187	0.094594493	0.665310635	0.215520947	0.380534721
1.097864496	0.284460453	0.085864167	0.633932	0.306253874	0.443836432

Q9UGT4,SUSD2,NEUROLOGY	P22692,IGFBP4,NEUROLOGY	P78560,CRADD,NEUROLOGY	Q9P126,CLEC1B,NEUROLOGY	Q9NP79,VTA1,NEUROLOGY	P01375,TNF,NEUROLOGY
Q9UGT4	P22692	P78560	Q9P126	Q9NP79	P01375
SUSD2	IGFBP4	CRADD	CLEC1B	VTA1	TNF
NEUROLOGY	NEUROLOGY	NEUROLOGY	NEUROLOGY	NEUROLOGY	NEUROLOGY
0.286042218	0.209671111	1.351442378	0.253806338	0.405338748	0.357818529
0.318507666	0.281401125	1.211420726	0.290417441	0.461947242	0.552750373
0.374360468	0.258242794	1.161750286	0.155814349	0.628855302	0.78051594
0.323424304	0.200907037	0.838975159	0.261684431	0.498719321	0.685724037
0.325561047	0.192469267	1.252577614	0.24033189	0.411995362	0.871697816
0.298458035	0.252402832	1.016492803	0.0982976	0.278432592	0.599071456
0.324997382	0.250711486	1.021790905	0.169516781	0.516342408	0.52679005
0.280758187	0.328030076	0.792344646	0.133591489	0.316548837	0.556247943
0.222441827	0.214983822	0.81529835	0.321257046	0.589147181	0.583498155
0.235761492	0.138792354	0.859279868	0.252490323	0.690397909	0.65012994
0.247345576	0.200434116	0.84886086	0.202599146	0.416792147	0.578304006
0.502745441	0.377513463	1.859609885	0.359011012	1.352004544	0.598822361
0.246951562	0.168322507	0.882580636	0.176936059	0.260887549	0.49383466
0.22090531	0.181960852	1.03053964	0.135794787	0.528875482	0.587475259
0.221933599	0.116217562	0.541337411	0.198360958	0.433198454	0.459998161
0.244600626	0.154127906	1.189371986	0.304940568	0.591766528	0.433228482
0.38858443	0.202781789	2.312574752	0.931998798	1.489677463	0.799960128
0.769610293	0.205797876	0.88227481	0.200323003	0.372083933	0.806306356
0.233452347	0.207891114	0.729004689	0.309841007	0.446923558	0.515591362
0.236416069	0.23230627	0.86934456	0.166293075	0.348710086	0.682925448
0.219060307	0.22095125	0.623300597	0.149591489	0.267460918	0.541187342
0.172324532	0.194764143	1.426816693	0.23903603	0.530380633	0.734737085
0.233646608	0.179455743	0.727742514	0.241283305	0.354780839	0.538493188
0.274294956	0.180253603	0.67604913	0.130942016	0.297652311	0.533588693
0.649994763	0.205441565	0.754190038	0.181130324	0.237977981	0.643985451
0.533403797	0.22321409	0.733414147	0.256210232	0.668546586	0.793883931
0.288271477	0.227604344	0.653156221	0.166824143	0.515770084	0.736266517
0.266203233	0.21540147	2.086811082	0.296581394	1.025338306	0.471555668
0.278722234	0.308683441	1.46886316	0.240665292	0.655469247	0.706959758
0.257581342	0.218287285	0.687199075	0.16145455	0.283044344	0.415839872
0.264364433	0.258117525	0.821538448	0.259822811	0.402622583	0.406238816
0.567385033	0.230046913	1.105730653	0.270649945	0.306614961	0.587190283
0.222827624	0.159574275	0.702465852	0.235059843	0.366046796	0.52220928
0.25833231	0.179405994	0.810396585	0.275266319	0.374568115	0.690876622
0.248738201	0.214076746	1.742187621	0.330380399	1.224675898	0.645773435

P08758,ANXA5,NEUROLOGY	P55103,INHBC,NEUROLOGY	Q15818,NPTX1,NEUROLOGY	P49023,PXN,NEUROLOGY	P78325,ADAM8,NEUROLOGY	P05164,MPO,NEUROLOGY
P08758	P55103	Q15818	P49023	P78325	P05164
ANXA5	INHBC	NPTX1	PXN	ADAM8	MPO
NEUROLOGY	NEUROLOGY	NEUROLOGY	NEUROLOGY	NEUROLOGY	NEUROLOGY
0.732753572	0.255731184	0.714249487	0.575903901	0.338376199	0.330953401
0.543555781	0.546237399	0.626853405	0.673056723	0.504595764	0.336178648
0.628593823	0.394036178	0.647072827	0.4799979	0.671612041	0.513522715
0.927423379	0.411966805	0.607686859	0.64478943	0.422991331	0.570460935
0.809498325	0.187751976	0.700326702	0.87569477	0.430235955	0.390041625
0.538194667	0.321167987	0.517237937	0.846569255	0.371208075	0.392020227
0.801236473	0.239450607	0.785672517	0.505611086	0.441902501	0.43178948
0.620369644	0.521377417	0.669242049	2.149051417	0.536481378	0.354215684
0.671146676	0.352867875	0.55290365	0.394746943	0.406858772	0.447481517
0.532443372	0.124593439	0.351915265	0.743652227	0.400257405	0.478204629
0.831968032	0.423724954	0.52635206	0.400646006	0.312970807	0.490117648
0.67722165	0.356902027	0.658657332	0.830470024	0.494794031	0.424548123
0.931159361	0.4040764	0.617837781	0.853521855	0.444821985	0.351354675
0.576902732	0.375295787	0.532812561	0.482232236	0.142654781	0.353577898
0.46557968	0.195915032	0.61459162	0.29876851	0.195725007	0.396392068
0.531632055	0.349532861	0.383394089	0.490185597	0.41014331	0.338704721
0.787307977	0.256814755	0.606760889	0.910291175	0.226864063	0.460221408
0.737850276	0.248686483	0.653880996	1.884785049	0.377330337	0.315738042
0.964999433	0.379796891	0.543028567	0.519933854	0.42065226	0.297507924
0.664573191	0.380139277	0.453036753	1.247292648	0.463615273	0.336248561
0.834740688	0.20652667	0.417920375	0.435637484	0.315716157	0.365387706
0.856960136	0.545518489	0.474605262	0.859220309	0.495480436	0.37138823
0.529902926	0.360682167	0.474934347	0.450562766	0.399619808	0.329465658
0.512455984	0.400729327	0.430534275	0.530417397	0.256707971	0.233274417
0.480863724	0.361157491	0.317361714	0.378850354	0.470087101	0.285685556
0.906387582	0.477012838	0.715190759	0.630513865	0.475000191	0.369513762
0.652070564	0.549046407	0.579507806	0.556093739	0.430593964	0.319901575
0.818752891	0.319259178	0.569552209	0.608614242	0.38111545	0.418500137
0.620885868	0.369360117	0.847626149	0.407818747	0.474835597	0.40666141
0.515984631	0.52679005	0.542614686	0.563583	0.258601043	0.30065899
0.452597337	0.25815331	0.398568611	0.478204629	0.323424304	0.239899158
0.474144925	0.315366211	0.536816157	0.398375271	0.515698588	0.267535084
0.477310507	0.373220465	0.491751037	0.382677239	0.298085892	0.266074102
0.571331508	0.353210467	0.601151281	0.406126198	0.391694289	0.276566822
0.651257506	0.371105169	0.538120062	1.158533691	0.354436725	0.355248386

P51531,SMARCA2,NEUROLOGY	O14618,CCS,NEUROLOGY	P13500,CCL2,NEUROLOGY	P30533,LRPAP1,NEUROLOGY	P29475,NOS1,NEUROLOGY	P05060,CHGB,NEUROLOGY
P51531	O14618	P13500	P30533	P29475	P05060
SMARCA2	CCS	CCL2	LRPAP1	NOS1	CHGB
NEUROLOGY	NEUROLOGY	NEUROLOGY	NEUROLOGY	NEUROLOGY	NEUROLOGY
1.012344274	1.832483236	0.30582961	0.24023196	0.152872414	0.831622098
1.538900548	1.562872405	0.317075871	0.262611136	0.096274664	0.897074682
1.166106859	2.081610292	0.287931992	0.212038773	0.177046472	0.691739143
1.203303026	1.03332924	0.242490478	0.288311442	0.155598494	2.120934401
1.44412938	1.953552463	0.507436781	0.248479718	0.168778144	1.378214079
1.432763013	1.889494082	0.491716953	0.21067631	0.145087989	0.518997682
1.259280785	2.234109658	0.290759856	0.188168886	0.091264652	0.341297086
1.222640278	0.811689581	0.306126533	0.169059149	0.18975371	0.427619634
1.341456247	1.045432562	0.298954949	0.229362271	0.036199159	0.610896551
1.806503437	1.42968766	0.297301779	0.151574608	0.114395325	0.38979838
1.355194571	1.362919167	0.317053894	0.26434611	0.0620941	0.405479252
1.554122257	2.484921023	0.476946715	0.280738727	0.256121452	0.725677276
1.531982694	1.437039781	0.287553041	0.207286778	0.120650015	0.442147611
1.276594202	1.099997313	0.278239664	0.343266268	0.054424498	0.43491338
1.115894007	1.239449937	0.186429232	0.12616643	0.078031476	0.691164009
1.112573	1.966731482	0.252105588	0.171086757	0.097490132	0.477641468
1.108877502	3.38792045	0.405254469	1.00159551	0.116032431	0.685438912
1.437936533	1.110338834	0.477178187	0.236858935	0.09031441	0.70275806
1.352191984	1.057237864	0.43178948	0.558682293	0.130281126	1.822349912
1.184189527	1.060981867	0.318485589	0.184718466	0.132494098	0.421557111
1.600807687	0.938676535	0.376859848	0.157366461	0.151165414	0.45040664
1.678881029	1.41705917	0.494862628	0.197359791	0.166627682	0.516270833
0.980643049	0.992335589	0.332102388	0.218741673	0.097098983	0.529425651
1.322988022	0.639049682	0.291344908	0.149996423	0.118684217	0.283672853
0.831103467	0.965936329	0.353381887	0.141522172	0.112188756	0.446768693
1.387127114	1.959247967	0.343123537	0.191126536	0.096134628	0.559069676
1.202719321	1.476007447	0.262592934	0.214641359	0.059754866	0.599279114
0.556440757	3.676925638	0.340658946	0.248910673	0.139738358	0.486900876
1.785217681	2.475808966	0.344720739	0.305702446	0.089715434	0.499792099
1.140684647	1.795517856	0.305935621	0.209366134	0.074624853	0.360557186
1.143059102	1.227565495	0.23855602	0.216014491	0.052661776	0.552865327
0.819945543	0.935688353	0.270969053	0.21851436	0.13320314	0.519141599
1.390689168	1.068435635	0.269975426	0.224719932	0.041726277	0.23079758
1.121088367	1.184846363	0.286796635	0.156496251	0.148537592	0.464741376
1.628789312	3.61000291	0.222642358	0.189503973	0.083846827	0.491512496

P07306,ASGR1,NEUROLOGY	Q86VW0,SESTD1,NEUROLOGY	O94907,DKK1,NEUROLOGY	Q13145,BAMBI,ONCOLOGY	O00592,PODXL,ONCOLOGY	Q9UHF1,EGFL7,ONCOLOGY
P07306	Q86VW0	O94907	Q13145	O00592	Q9UHF1
ASGR1	SESTD1	DKK1	BAMBI	PODXL	EGFL7
NEUROLOGY	NEUROLOGY	NEUROLOGY	ONCOLOGY	ONCOLOGY	ONCOLOGY
0.390826446	0.458947171	0.544687249	1.442328714	0.911553982	0.334621025
0.519177584	0.619252632	0.590701017	1.572870616	1.049571172	0.406971593
0.398623868	0.867117857	1.038643101	1.562872405	0.862801147	0.406266975
0.379349621	0.855950932	0.56097174	2.013631932	0.689537061	0.388638303
0.689537061	1.480515916	0.958865256	1.483597765	1.108877502	0.444482955
0.477509056	1.22858698	0.746440929	1.368504331	0.935818075	0.435184778
0.324412204	0.845396474	0.88441782	1.008632068	0.673150035	0.356037227
0.273876996	0.621488672	0.708431369	1.136738178	0.565618024	0.336551689
0.323828081	0.778948579	0.517345505	0.959064667	0.615785585	0.34793748
0.217366271	0.777923395	0.722264917	1.042321254	0.700812299	0.452221033
0.338376199	0.651031836	0.451093999	1.144010267	0.692170807	0.359434302
0.553977774	0.979352412	1.446433503	1.64992449	0.969289817	0.503268428
0.341983826	0.804185372	0.793003974	2.025109615	0.805747661	0.370000724
0.238076974	0.643985451	0.532701778	1.14092187	0.448848351	0.329671254
0.220691046	0.896763833	0.52166661	0.859696895	0.481998312	0.250242719
0.323088208	1.323905365	0.387750353	0.937571096	0.571331508	0.406689599
0.263358513	1.28422679	1.71225788	1.17299755	0.825419828	0.793278855
0.299182978	1.793651989	0.716878237	1.261377409	0.6012763	0.477674577
0.397878542	1.052631155	0.633844124	1.156207231	0.691116103	0.462523957
0.386248169	0.962727896	0.533145051	1.307490079	0.717425037	0.382756823
0.288851522	0.766416221	0.487576332	1.006746181	0.632220622	0.280310949
0.493013822	0.607139524	0.688916005	1.203469851	0.588045625	0.354731659
0.365463695	1.082074627	0.818015452	1.769694094	0.520943928	0.310227826
0.336365116	0.582084296	0.370411296	1.067399322	0.542464262	0.341746863
0.316746372	0.989450884	0.400618236	1.230717805	0.744322628	0.385713087
0.367292158	1.041960076	1.328870022	0.953959551	0.844752137	0.384325338
0.36948815	0.918212514	0.329899844	1.351255041	0.740514556	0.28813164
0.301431063	0.896328826	1.030182544	1.345833558	0.600734739	0.544611745
0.484644908	1.059218335	0.95972967	1.813152158	0.699986983	0.479366169
0.371851887	0.693323228	0.324142478	0.811127156	0.802459229	0.340540903
0.309068815	0.627766522	0.527082245	0.78051594	0.682736127	0.556247943
0.308426792	0.772175133	1.068657834	1.387511761	0.724621742	0.338704721
0.255607133	0.681176231	0.510647625	1.104351932	0.508387337	0.353970246
0.359160351	0.697758645	0.865676559	1.254054459	0.558992178	0.219607617
0.341723176	0.864537231	0.93646696	1.064001364	0.734482489	0.389852421

Q9BSL1,UBAC1,ONCOLOGY	P82980,RBP5,ONCOLOGY	Q7Z5R6,APBB1IP,ONCOLOGY	P09110,ACAA1,ONCOLOGY	O43895,XPNPEP2,ONCOLOGY	Q96I82,KAZALD1,ONCOLOGY
Q9BSL1	P82980	Q7Z5R6	P09110	O43895	Q96I82
UBAC1	RBP5	APBB1IP	ACAA1	XPNPEP2	KAZALD1
ONCOLOGY	ONCOLOGY	ONCOLOGY	ONCOLOGY	ONCOLOGY	ONCOLOGY
0.990068329	0.332516999	0.618952241	0.180967182	0.207574338	0.872907084
1.021224461	0.431819411	0.583498155	0.220385315	0.437028721	1.433855857
1.101905116	0.483571123	0.59234106	0.341533737	0.156333623	1.527952824
0.77716886	0.314384065	0.536369832	0.141875757	0.208945704	0.671658595
0.983842989	0.533477748	1.074227828	0.235696134	0.199574597	0.753667455
1.222894545	0.25278802	0.51687954	0.292619943	0.145168465	1.208652907
0.750019495	0.18367154	0.476748399	0.075493697	0.357248535	0.918849191
0.711531731	0.290598669	0.591643486	0.21851436	0.196010114	0.978063473
0.660898213	0.383606747	0.345151103	0.141453522	0.141012993	0.626636191
0.905006463	0.203655121	0.543706507	0.189622229	0.166108753	0.665587387
0.818412452	0.2310697	0.533218965	0.318618071	0.145369851	0.708136802
1.564498201	0.410683816	0.497752339	0.245211747	0.197798035	1.727876375
0.786217292	0.329922712	0.550227442	0.113306301	0.275801078	1.192756873
0.912249272	0.142803179	0.457962064	0.171015619	0.293941303	0.893909076
0.68387284	0.284894564	0.330311705	0.194750643	0.397465074	0.534551184
0.974139304	0.278606341	0.46852567	0.365463695	0.075656089	0.550494479
1.752118073	0.234652868	0.63055757	0.267368239	0.430057062	0.385766561
0.784801661	0.24815269	0.39727227	0.180291089	0.256032703	0.670263371
0.870671256	0.399121527	0.605080921	0.204461342	0.389933497	0.619467286
0.666649339	0.29781741	0.842413231	0.155846753	0.232209677	0.92242275
0.638474099	0.199754513	0.424960307	0.180303587	0.128906775	0.927873476
0.856900738	0.245807357	0.686960951	0.40287383	0.085870119	1.016140574
0.589514825	0.194723647	0.570105174	0.222580637	0.116944831	0.872786081
0.54129989	0.181759163	0.409433201	0.23903603	0.294532757	0.80229238
0.656287564	0.244702373	0.567699746	0.198429716	0.156876374	0.866517025
0.997300377	0.349000256	0.372910158	0.203937643	0.431879278	0.409972771
0.816768991	0.318838997	0.382518121	0.251390145	0.420506498	0.856247633
1.596486109	0.327099169	0.467358002	0.104523555	0.075399564	0.909408252
1.462665627	0.328576225	0.531595206	0.247020041	0.090402094	0.659616778
0.911238116	0.225343853	0.505330793	0.180103734	0.202767734	0.671937988
0.760331254	0.238093477	0.368388518	0.184808113	0.365311734	0.488083538
0.979488188	0.395980145	0.459966278	0.250017329	0.176936059	1.253011799
0.815467904	0.159541096	0.272362503	0.209250068	0.072554052	0.705197859
0.698968817	0.183506109	0.276183684	0.121405021	0.188181929	0.676564789
1.881391381	0.27494215	0.534662352	0.348710086	0.323536414	0.664481068

Q9NX58,LYAR,ONCOLOGY	Q14508,WFDC2,ONCOLOGY	Q6UWW8,CES3,ONCOLOGY	Q9Y265,RUVBL1,ONCOLOGY	Q14241,ELOA,ONCOLOGY	Q9BUE0,MED18,ONCOLOGY
Q9NX58	Q14508	Q6UWW8	Q9Y265	Q14241	Q9BUE0
LYAR	WFDC2	CES3	RUVBL1	ELOA	MED18
ONCOLOGY	ONCOLOGY	ONCOLOGY	ONCOLOGY	ONCOLOGY	ONCOLOGY
1.381753244	0.268129154	0.189872122	0.472013491	0.36948815	0.710102898
1.234990548	0.259013644	0.316066492	0.362210431	0.398264833	0.733363312
1.568950678	0.268203506	0.253525014	0.356012549	0.539015999	1.018114627
1.074600191	0.266831335	0.178822478	0.750279477	0.504595764	0.721714427
1.336537207	0.286637645	0.306253874	0.444452147	0.471196262	0.894963035
1.731353119	0.261032256	0.291122853	1.268303484	0.525003884	0.739437441
1.142267068	0.301326613	0.3459415	0.191537662	0.379218171	0.823077395
1.608147793	0.331642315	0.375243764	0.228552895	0.274561263	0.587923357
1.650153234	0.202080217	0.213720914	0.369539375	0.333856492	0.872483649
1.224166675	0.248290333	0.299992846	0.27110056	0.609162906	0.743497605
1.181238267	0.202374581	0.235843215	0.948487062	0.388746071	0.583457712
1.960742389	0.380587478	0.295452887	0.33028881	0.60202696	0.798243054
2.69708315	0.22422204	0.395431581	0.260544192	0.342363309	0.871154192
1.402693692	0.20389524	0.127803566	0.320723062	0.234913251	0.71484383
1.384053777	0.168299174	0.213750544	0.522100701	0.22777795	0.746285727
1.097864496	0.2516691	0.206784507	0.983570248	0.506593334	0.663928598
1.227991011	0.271307343	0.234539042	1.376114009	0.47900081	0.667805555
1.39910089	0.251424998	0.265410987	7.277787866	0.501631552	0.79940583
0.884050078	0.224190958	0.277507756	0.430623811	0.573195822	0.675908564
1.389629225	0.240531876	0.236137652	0.454641095	0.507401609	0.590005374
1.519714165	0.212024076	0.218423502	0.311024471	0.294634852	0.83961509
1.507021501	0.269265257	0.314623862	0.24449892	0.424577552	0.718071794
2.952628811	0.234360283	0.189031688	0.2584756	0.288451366	0.604158922
1.714395538	0.214299442	0.261249467	0.249307813	0.198333461	0.677832163
2.044857061	0.308020868	0.318110523	0.762072415	0.31983506	0.602778557
1.107034359	0.24779174	0.266517099	0.548095806	0.491887398	0.494211333
1.241771736	0.249567158	0.160394881	0.210428206	0.408497739	0.670681634
1.601362581	0.241768803	0.247362721	0.645057647	0.504385952	0.858565436
1.662783111	0.298333936	0.268575573	0.306168974	0.732195088	0.764665135
1.363769665	0.178117355	0.457295935	0.287812269	0.339598031	0.834914286
0.926331193	0.285626155	0.204787562	0.254211288	0.310335361	0.656287564
1.475291457	0.228347041	0.433919703	0.520294369	0.374983755	0.770624522
0.881357967	0.202655326	0.303359417	0.77448006	0.338165175	0.54329211
1.447637113	0.212524342	0.209366134	0.287114879	0.328963631	0.471261588
1.36679796	0.291223766	0.241802321	0.635515845	0.701395461	0.574508443

P01375,TNF,ONCOLOGY	O95786,DDX58,ONCOLOGY	P50579,METAP2,ONCOLOGY	Q14790,CASP8,ONCOLOGY	Q9NSA1,FGF21,ONCOLOGY	Q9H4P4,RNF41,ONCOLOGY
P01375	O95786	P50579	Q14790	Q9NSA1	Q9H4P4
TNF	DDX58	METAP2	CASP8	FGF21	RNF41
ONCOLOGY	ONCOLOGY	ONCOLOGY	ONCOLOGY	ONCOLOGY	ONCOLOGY
0.764135293	0.40598547	0.422229709	0.534477085	0.100802268	0.451406782
0.665448997	0.493150534	0.450125749	0.355815188	0.286677384	0.289894527
0.832891226	0.550876185	0.415522931	0.662457589	0.243788166	0.484107718
0.697710282	0.625941615	0.342339579	0.483035123	0.078237279	0.315191383
0.82857259	0.534958914	0.464258426	0.502222998	0.281362117	0.604452133
0.69495911	0.481230504	0.453602344	0.537859028	0.103866333	0.487306037
0.521847437	0.430832802	0.301577353	0.285645954	0.068147285	0.253771155
0.625377838	0.343599538	0.29441029	0.319126429	0.264309466	0.211319821
0.647162537	0.425402377	0.336528361	0.385445823	0.098714101	0.379428512
0.771265776	0.487103414	0.420127754	0.358886609	0.096823429	0.334435523
0.555015513	0.445192132	0.369206536	0.364982703	0.11393635	0.304264938
0.596543811	0.564834455	0.191033823	0.821652345	0.082618003	0.536035331
0.525040275	0.482265663	0.276413503	0.327848227	0.15256543	0.277161735
0.683446351	0.388988659	0.34793748	0.250641984	0.395458991	0.281186649
0.594109186	0.434943527	0.218635564	0.256619018	0.110805393	0.258708615
0.526936127	0.654515834	0.311823162	0.424342181	0.173378871	0.420419065
0.743755326	0.620842833	0.632658996	0.548361808	0.099125497	0.497166158
0.686675312	0.465934802	0.359683529	0.430027253	1.731233115	0.350430439
0.552329083	0.456599125	0.425903946	0.306338798	0.422200444	0.465837924
0.55856613	0.779704843	0.253208897	0.343528095	0.155415252	0.27559087
0.522462719	0.379113044	0.229155688	0.262320052	0.252665396	0.336481712
0.945598728	0.59147947	0.566206415	0.34276697	0.142121823	0.504560789
0.664481068	0.377278031	0.443959507	0.30155645	0.071798624	0.384618483
0.566402681	0.353872118	0.315431796	0.315584882	0.354953022	0.258798292
0.851099672	0.476352016	0.279244351	0.326012684	0.108947123	0.23442527
0.597661286	0.317207767	0.396447024	0.310507496	0.187335991	0.303717091
0.644342651	0.446335357	0.322528823	0.388288262	0.270312475	0.332540048
0.569631171	0.577903295	0.410997066	0.59225895	0.064726486	0.448910579
0.885031065	0.464129724	0.325019909	0.461179407	0.136635095	0.319037961
0.455587478	0.298602883	0.293839449	0.274808779	0.479864835	1.316310644
0.66595657	0.373556922	0.32506497	0.363543524	0.190636992	0.252280395
0.670495707	0.517130392	0.462588081	0.294920906	0.546350998	0.37079662
0.457264239	0.48380581	0.439976996	0.264364433	0.094254153	0.276202829
0.550990748	0.373738217	0.215999518	0.318507666	0.083608881	0.20903262
0.711482413	0.632352102	0.998268633	0.368107743	0.068052879	0.429193461

Q9UBX1,CTSF,ONCOLOGY	O75629,CREG1,ONCOLOGY	P14136,GFAP,ONCOLOGY	Q9UK85,DKKL1,ONCOLOGY	P55273,CDKN2D,ONCOLOGY	Q7L5Y9,MAEA,ONCOLOGY
Q9UBX1	O75629	P14136	Q9UK85	P55273	Q7L5Y9
CTSF	CREG1	GFAP	DKKL1	CDKN2D	MAEA
ONCOLOGY	ONCOLOGY	ONCOLOGY	ONCOLOGY	ONCOLOGY	ONCOLOGY
0.301305727	0.437999158	0.608572058	0.461211375	0.81253395	1.225270258
0.246011899	0.433709215	0.496408595	0.285546974	0.693034943	1.115739322
0.29508449	0.43708931	0.36905302	0.641490587	0.894838975	1.543922141
0.256245753	0.381300413	0.304264938	0.666972877	38.41239465	1.132255855
0.300117635	0.438515578	0.627418511	0.329511335	0.834162291	1.278808289
0.228394529	0.276030578	0.364653968	0.49373198	0.701930452	1.2894001
0.254634534	0.276777774	0.319724233	0.452503231	1.638868387	1.121166078
0.269358594	0.320589704	0.289452796	0.479798316	0.621790293	1.141475583
0.246848879	0.355815188	0.371233806	0.453822486	1.093611258	1.256839128
0.170388515	0.242978404	0.425431865	0.281420631	1.378596254	1.683775739
0.416416748	0.564286602	0.290437571	0.247654373	0.566402681	1.159337004
0.317383713	0.554784736	0.444852819	0.667111585	0.788892157	1.377927517
0.193298185	0.280446989	0.3459415	0.480630464	0.734584317	1.167562678
0.289292334	0.492535631	0.414085331	0.208699638	0.794819956	0.906199123
0.237401343	0.262993674	0.251198543	0.381247558	0.514484672	1.178866214
0.185822873	0.225656462	0.246131293	0.433739278	0.670356296	0.921719706
0.259049553	0.381776445	0.426110646	0.693034943	1.145597302	1.117209696
0.337720111	0.343075974	0.378929142	0.507577492	0.921400317	1.363864198
0.215924672	0.399204531	0.41091161	0.731484906	0.414717261	1.227565495
0.278760876	0.322506468	0.257010641	0.455082495	0.461371246	1.064960558
0.171990407	0.395404172	0.316724418	0.37079662	0.619123875	0.970432653
0.249117798	0.398540985	0.370642443	0.435516716	0.767213494	1.120000984
0.206254857	0.316724418	0.435637484	0.39069102	0.671146676	1.07601635
0.189661664	0.274789732	0.485350876	0.24431257	0.793718865	1.469677897
0.191418212	0.32778006	0.34276697	0.39077227	1.171210181	1.144010267
0.241517561	0.318662244	0.46901306	0.558875951	0.586742744	0.865016765
0.19020143	0.323626129	0.289492926	0.225797278	0.740565887	1.316036954
0.306551209	0.429401757	0.414602293	0.508316864	0.667111585	1.496922987
0.360057694	0.51181701	0.423490056	0.489981777	0.830930662	1.56178948
0.316614668	0.359060784	0.472111654	0.33877516	1.395323845	1.037923418
0.222364748	0.327484834	0.416301309	0.272475799	1.023563069	1.271824848
0.226911243	0.353921179	0.428480068	0.303233279	0.737032427	1.228672143
0.191020582	0.290377183	0.306933921	0.235418565	0.97874165	0.996954798
0.169575541	0.250572501	0.534254848	0.396309649	0.787744674	1.203469851
0.566402681	0.485283596	0.969760233	0.63437156	0.802236771	1.037419937

Q9NTU7,CBLN4,ONCOLOGY	Q14213_Q8NEV9,EBI3_IL27,ONCOLOGY	Q9C0C4,SEMA4C,ONCOLOGY	P31994,FCGR2B,ONCOLOGY	Q9BTE6,AARSD1,ONCOLOGY	P47992,XCL1,ONCOLOGY
Q9NTU7	Q14213_Q8NEV9	Q9C0C4	P31994	Q9BTE6	P47992
CBLN4	EBI3_IL27	SEMA4C	FCGR2B	AARSD1	XCL1
ONCOLOGY	ONCOLOGY	ONCOLOGY	ONCOLOGY	ONCOLOGY	ONCOLOGY
0.597288562	0.315825595	0.669938236	0.461371246	1.548101432	0.239467205
0.686627717	0.404524784	0.893909076	1.036485547	1.794149363	0.772389255
0.673430048	0.448102289	0.716679503	0.648734469	2.326401331	0.285408459
0.613102418	0.466257876	0.859279868	0.540737382	1.186572292	0.255908505
0.493458273	0.494759735	1.112033307	0.813266448	1.514141463	0.633888061
0.342126083	0.357719334	0.9568071	0.570105174	2.212074539	0.241651524
0.323805636	0.328416838	0.642113395	0.538381223	2.342582732	0.285171162
0.391151662	0.280680356	0.530343871	0.585118201	1.111493876	0.241567789
0.306593709	0.337182134	0.519321551	0.149156629	1.134298227	0.189071
0.553939376	0.388019214	0.661769176	0.54699517	2.087245067	0.227620121
0.524894723	0.298106554	0.622868708	0.426672194	1.112341671	0.206655548
0.469891638	0.410997066	0.846686622	0.588045625	3.572663409	0.273971931
0.532849494	0.291264141	0.775769521	0.316724418	1.043984288	0.267757706
0.419632988	0.249705586	0.408695992	0.407847015	1.379456535	0.211656984
0.355889186	0.260887549	0.576063598	0.428420672	1.374779267	0.135653671
0.293737629	0.27559087	0.627679502	0.538679848	1.869950396	0.235010969
0.479100426	0.305215472	0.756388849	0.263760421	5.682398462	0.306020456
0.498201061	0.341368064	0.672823499	0.219897026	1.863351696	0.251616773
0.562178428	0.371645746	0.796253658	0.319037961	1.255446021	0.81270293
0.528106206	0.530086609	0.966137211	0.11088991	1.438235574	0.480397317
0.570223736	0.296375891	0.627462002	0.268687293	0.680657058	0.196377289
0.385659619	0.343337656	0.629334963	0.606004327	1.831848255	0.252420327
0.385713087	0.345797657	0.51128514	0.323334644	1.400750493	0.299494206
0.375920631	0.303044171	0.486327474	0.488659011	0.761861153	0.260706779
0.330151476	0.366300608	0.874299816	1.083275352	1.451253956	0.3103999
0.37298771	0.420273384	0.96306161	0.394364064	1.846892634	0.210355289
0.409376445	0.321212513	0.683493726	0.653563808	1.603139534	2.048119651
0.409518349	0.198223512	0.623905745	0.606929142	4.542542461	0.238622171
0.343099755	0.378981676	0.853581019	0.497166158	2.916220888	0.329694106
0.398817328	0.274846879	0.873209663	0.522752514	0.999792077	0.170731362
0.473914924	0.416503349	0.776253622	0.235418565	1.490917057	0.289412673
0.458184322	0.419603902	0.801292012	0.552099424	1.987011194	0.234100513
0.346685644	0.371877663	0.535663909	0.388207528	1.394840346	0.224501968
0.382465097	0.231743375	0.533699661	0.18854751	1.28395977	0.24170178
0.443528895	0.362185326	0.615060401	0.782140665	4.03340982	0.210136692

O14713,ITGB1BP1,ONCOLOGY	P02760,AMBP,ONCOLOGY	P09958,FURIN,ONCOLOGY	P55789,GFER,ONCOLOGY	P80075,CCL8,ONCOLOGY	P26447,S100A4,ONCOLOGY
O14713	P02760	P09958	P55789	P80075	P26447
ITGB1BP1	AMBP	FURIN	GFER	CCL8	S100A4
ONCOLOGY	ONCOLOGY	ONCOLOGY	ONCOLOGY	ONCOLOGY	ONCOLOGY
1.176335837	0.492126121	0.250676733	0.345989461	0.011561721	0.933032992
1.344155456	0.518350551	0.329305839	0.421294212	0.011459596	0.927359097
1.216132144	0.468818043	0.277757929	0.406971593	0.02001326	1.391556996
1.202552601	0.536555756	0.316504957	0.447636629	0.019837857	0.861785064
0.88196909	0.591889595	0.257171023	0.57271925	0.021588398	0.734075318
0.49383466	0.446892581	0.228109747	0.421878655	0.019758264	0.995573683
0.894652918	0.394118124	0.217321076	0.428005132	0.0147842	0.964531326
0.543781886	0.418239144	0.230094755	0.399564413	0.011197341	0.843114222
0.679714121	0.295616766	0.263906721	0.512775771	0.013544022	0.962594443
1.241255405	0.354780839	0.274542233	0.347889249	0.022984419	1.099844832
0.654198338	0.394801671	0.315453661	0.445161274	0.015821169	0.957736042
0.619596114	0.561633151	0.387965427	0.538679848	0.017583913	1.137526378
0.652613167	0.304412604	0.193633436	0.340210601	0.013887204	0.861426732
0.833757651	0.288951647	0.195670748	0.362813489	0.012119718	1.304231528
0.877700114	0.227352063	0.216014491	0.305236628	0.005346461	0.9151626
0.928066442	0.283162083	0.198470983	0.438211728	0.011509748	1.161830815
0.597329964	0.367750702	0.377095019	0.553939376	0.025619746	1.979176122
0.694670145	0.356679449	0.290759856	0.610473256	0.014280596	1.273412649
0.751997619	0.421206615	0.434220577	0.368771738	0.018135999	1.06873191
0.430982143	0.410826173	0.192309242	0.321680413	0.016122285	0.700860878
0.621187197	0.355741206	0.362536963	0.333417099	0.012765554	0.881785709
1.079602333	0.417891407	0.394965898	0.559573676	0.018693752	1.332651913
0.674831863	0.321301584	0.166235453	0.305596516	0.012223489	0.924983798
0.642781361	0.331504417	0.190835305	0.400784883	0.007731157	0.707842357
0.527374602	0.388234438	0.184258107	0.462203471	0.016777831	0.933356412
0.90789666	0.412996082	0.30477152	0.343623355	0.014172126	1.271736694
0.875087995	0.408865999	0.224735509	0.359608743	0.010515053	1.06984367
0.753719697	0.401007187	0.248720961	0.401090583	0.014460888	1.577893682
0.897821158	0.471359595	0.289894527	0.506382692	0.014505059	1.698663397
0.960794635	0.35502684	0.323828081	0.511107972	0.014317268	0.891125172
1.015084621	0.404356582	0.197387153	0.334134302	0.016357544	0.788126983
1.230376625	0.389744346	0.313100995	0.50736644	0.016472462	0.956077849
0.823362701	0.366046796	0.197400835	0.389393309	0.016256945	0.878430468
1.154445447	0.352379036	0.196458977	0.405366845	0.012819644	0.907833732
0.799295017	0.440862295	0.304518124	0.4118526	0.013979921	1.636598007

Q96NA2,RILP,ONCOLOGY	Q9BS26,ERP44,ONCOLOGY	O95498,VNN2,ONCOLOGY	P30041,PRDX6,ONCOLOGY	Q9UBG3,CRNN,ONCOLOGY	Q07954,LRP1,ONCOLOGY
Q96NA2	Q9BS26	O95498	P30041	Q9UBG3	Q07954
RILP	ERP44	VNN2	PRDX6	CRNN	LRP1
ONCOLOGY	ONCOLOGY	ONCOLOGY	ONCOLOGY	ONCOLOGY	ONCOLOGY
1.534746096	0.661356472	0.678067123	1.154205411	1.133276581	0.40801667
1.198973689	0.646131627	0.443959507	0.594933367	0.614421242	0.527850029
2.590085998	0.718221129	0.708185888	1.21167266	1.402985404	0.461179407
1.153885442	0.409206226	0.124463964	1.30161249	0.354387593	0.349969233
1.399779903	0.678866594	0.340966049	0.689059276	0.492023797	0.61890934
1.370592787	0.704074501	0.344434127	0.949802856	0.500450749	0.554285049
1.217650417	0.379481116	0.40598547	1.31494276	0.303296341	0.3509409
0.833410973	0.479332943	0.382783354	0.57990963	0.613059923	0.334783423
1.581945612	0.376964351	0.526571009	0.669752516	0.595139591	0.238986329
1.962373967	0.423108625	0.406182503	1.232083472	0.633141558	0.304708151
0.797137221	0.357372369	0.33049492	1.159417366	0.465999399	0.289111921
2.689802041	0.879283316	0.780137323	1.360088001	0.793058942	0.511746062
1.380700112	0.528325885	0.36271291	0.978538148	0.533255927	0.621273318
1.747266899	0.433198454	0.336225255	0.596543811	0.487982055	0.396749415
1.428102965	0.257849294	0.30065899	0.617709319	0.303527681	0.232419013
2.354139805	0.321101209	0.365590377	0.923190319	0.161555302	0.19914622
5.776917934	0.519861781	0.541412462	1.783857039	1.370497788	0.49134218
1.235247384	0.461947242	0.455303356	0.735858358	1.523933554	0.415897524
1.568515733	0.737236804	0.669891801	0.708971727	0.467649646	0.515234106
1.184189527	0.436847004	0.343670995	0.470935048	0.654425105	0.321791919
0.887611337	0.393845037	0.544876056	0.737492355	0.325358015	0.283731847
2.329628645	0.438333243	0.446551972	0.831679744	0.490083677	0.329191731
1.105577377	0.430206134	0.503163787	0.746958502	0.461083518	0.294185899
0.876301966	0.411025555	0.186895016	0.589841812	0.603196516	0.316504957
0.908715127	0.424695286	0.316373354	0.732245841	0.703537876	0.32778006
1.827282888	0.542802774	0.454704126	0.94927632	1.203970464	0.349242248
1.363675139	0.456504188	0.350430439	0.988148656	0.432628316	0.337720111
4.781953164	0.355839852	0.486496051	1.901318202	0.747372819	0.309905443
2.898687885	0.657289116	0.803461054	1.647867221	0.421323415	0.305321269
1.098549593	0.551525693	0.407847015	0.446180695	0.551258156	0.419807545
1.390496391	0.461851193	0.34279073	0.523985938	0.500658924	0.2584756
1.543387151	0.492262587	0.567306382	0.882397128	0.523840678	0.384751805
1.595269313	0.301472853	0.240132071	0.641801915	0.277738677	0.222812179
0.990617491	0.441535089	0.312623904	1.186736798	0.228996904	0.39713461
5.968217034	0.528106206	0.536890581	1.379839055	0.713457798	0.325470795

P06756,ITGAV,ONCOLOGY	P35813,PPM1A,ONCOLOGY	P13726,F3,ONCOLOGY	Q16772,GSTA3,ONCOLOGY	P29017,CD1C,ONCOLOGY	P49788,RARRES1,ONCOLOGY
P06756	P35813	P13726	Q16772	P29017	P49788
ITGAV	PPM1A	F3	GSTA3	CD1C	RARRES1
ONCOLOGY	ONCOLOGY	ONCOLOGY	ONCOLOGY	ONCOLOGY	ONCOLOGY
1.142108727	1.099311313	0.408101523	0.289553131	0.647835755	0.570856486
0.900064315	1.108646942	0.542802774	0.3139703	0.637899034	0.721264339
0.991098259	1.11195623	0.359658598	0.345462256	0.657425809	0.705686834
1.404834325	1.035264924	0.375425877	0.482733884	0.602736777	0.688009314
1.211000953	1.222640278	0.420360787	0.494314112	0.56327057	0.661677442
1.037779541	1.174787646	0.461947242	0.267609271	0.464902471	0.691307748
0.679619899	1.251102508	0.37950742	0.171146062	0.573553512	0.659433918
0.878917709	1.061496784	0.35370046	0.174899734	0.515341257	0.555862515
0.816429377	1.120466876	0.294696126	0.276605165	0.596254436	0.650400378
0.717425037	1.139420288	0.256370115	0.281186649	0.483705216	0.810677496
0.971038229	1.119224928	0.306423745	0.370103325	0.599486845	0.622005827
1.036701101	0.982888725	0.442668923	0.23594132	0.586905446	0.650400378
0.790260388	1.125448468	0.259372962	0.267016352	0.514128182	0.622825535
0.693563557	1.334593146	0.234279074	0.234279074	0.477211263	0.518961709
0.678255149	1.052631155	0.265687084	0.400868233	0.48727226	0.669056522
0.567306382	0.914338327	0.228204635	0.345270744	0.523223774	0.571014783
1.01395948	0.960461706	0.412481123	0.538717188	0.539539317	0.486900876
1.235675562	1.059805853	0.352867875	0.470869767	0.630513865	0.751059963
1.120699895	0.969693017	0.338024565	0.38969032	0.653609111	0.696840316
0.8229633	1.016492803	0.409433201	0.420448208	0.633404929	0.529902926
1.091490823	1.128182137	0.256299044	0.263358513	0.550380019	0.527228403
0.692314754	1.073409082	0.370693828	0.373323958	0.62135945	0.62135945
0.959995801	0.785890382	0.319768559	0.274618362	0.689489267	0.438971749
0.632132984	1.113035803	0.349629785	0.321725011	0.461499183	0.575026361
0.889335697	0.908337282	0.311996122	0.181973465	0.514021283	0.671891414
0.819207029	1.021578452	0.333440211	0.167461342	0.664527128	0.627331539
0.611786425	1.172103525	0.396254713	0.242911046	0.544649496	0.654016981
0.723066379	1.146630055	0.366757913	0.284164847	0.505646133	0.92999831
0.899191311	1.466421659	0.438394013	0.340729792	0.58548333	0.782791502
0.686151948	1.333575958	0.411282046	0.137938778	0.588983858	0.600651466
0.724069457	1.31303011	0.536741744	0.469143116	0.438880477	0.500485439
0.90909313	1.199971382	0.363241262	0.451751094	0.541449991	0.602569686
0.792124991	0.844986384	0.283044344	0.19468316	0.534662352	0.529645878
0.703342841	1.115043505	0.343361455	0.246063061	0.569433786	0.604577838
0.683493726	0.989862471	0.287114879	0.337720111	0.622221435	0.665495124

O00221,NFKBIE,ONCOLOGY	A4D1B5,GSAP,ONCOLOGY	Q7Z4W1,DCXR,ONCOLOGY	Q9Y243,AKT3,ONCOLOGY	Q86SJ2,AMIGO2,ONCOLOGY	Q7LG56,RRM2B,ONCOLOGY
O00221	A4D1B5	Q7Z4W1	Q9Y243	Q86SJ2	Q7LG56
NFKBIE	GSAP	DCXR	AKT3	AMIGO2	RRM2B
ONCOLOGY	ONCOLOGY	ONCOLOGY	ONCOLOGY	ONCOLOGY	ONCOLOGY
0.838917008	0.045241796	0.385472541	0.917576279	0.58743454	1.07758375
0.913578117	0.061018901	0.360307353	0.896204577	0.744013137	0.996885697
0.786980612	0.057571555	0.475988954	1.420008928	0.810508938	1.160462578
0.967745769	0.049046704	0.432178737	0.900313901	0.313796246	1.015014263
1.136423051	0.066500085	0.402315716	1.12537046	0.654198338	1.249109541
0.920187651	0.048214136	0.364249778	1.081474763	0.630951055	1.008632068
0.563856519	0.08700246	0.358017	0.712025098	0.509374979	1.109723302
0.696357471	0.049490657	0.255058485	0.590823862	0.465837924	0.487982055
0.788618795	0.052749455	0.560893978	0.951318276	0.363216085	0.74241615
0.863998073	0.057404196	0.276375187	1.145438499	0.475758059	1.003402196
0.697275164	0.063817681	0.324209889	1.091188239	0.306933921	1.188959852
1.008212677	0.047435139	0.398762044	0.943438251	0.875512693	1.273500918
0.698532914	0.045004092	0.253665637	0.890569433	0.472733825	0.668222284
0.442699608	0.052269023	0.277065695	0.792894047	0.363392362	0.857851599
0.540699902	0.048826223	0.576862745	0.772549886	0.340895155	0.84264683
0.997715228	0.046791815	0.349775223	0.547260638	0.455208689	0.838277609
0.747321016	0.068607019	0.421907898	1.175439268	0.464934697	0.69375588
0.708480475	0.060765657	0.385392393	1.179601858	0.473947775	1.453770968
0.908337282	0.049063705	0.322037366	1.33783482	0.46904557	1.009961291
0.976843939	0.04389803	0.366402182	0.748824737	0.553478814	0.688725023
0.762019594	0.034194308	0.383580158	0.771693575	0.377958568	0.764453154
1.057384439	0.036251889	0.445655248	0.817731998	0.328553451	0.994814886
1.018891197	0.039827259	0.292417184	0.707008762	0.428361285	1.196483084
0.594150368	0.046125233	0.323200201	0.95019795	0.343385256	0.642647712
0.929353908	0.047382561	0.322976253	1.109184991	0.513415942	0.563856519
0.709512497	0.111878134	0.351208581	0.695778498	0.530527706	0.608150374
0.698290863	0.04917947	0.360282379	0.596626515	0.397107083	1.020799834
1.116745158	0.040004331	0.46706654	1.092701996	0.420186	0.984184022
0.909156145	0.076441505	0.400063246	1.072665309	0.601151281	1.432167268
0.593162789	0.078606913	0.357273298	0.945140032	0.397244734	0.928388141
0.687770909	0.049703801	0.266609483	0.979556083	0.547791961	0.745406859
0.983229427	0.051214637	0.465676505	0.938741601	0.684299596	0.900064315
0.650761136	0.042938036	0.298189218	0.783497187	0.317515736	0.304792646
0.398900269	0.061943641	0.37785379	0.787690073	0.359758331	0.905571211
1.120233906	0.04273909	0.458025555	0.608234687	0.510470678	0.676236597

P80511,S100A12,ONCOLOGY	P80303,NUCB2,ONCOLOGY	Q9HAT2,SIAE,ONCOLOGY	Q9BXY4,RSPO3,ONCOLOGY	O60259,KLK8,ONCOLOGY	Q6UX82,LYPD8,ONCOLOGY
P80511	P80303	Q9HAT2	Q9BXY4	O60259	Q6UX82
S100A12	NUCB2	SIAE	RSPO3	KLK8	LYPD8
ONCOLOGY	ONCOLOGY	ONCOLOGY	ONCOLOGY	ONCOLOGY	ONCOLOGY
0.27289162	0.273763118	0.538343907	0.439824538	0.40354459	0.719616414
0.082721147	0.419138805	0.469500956	0.427352954	0.39025797	0.739181216
0.112726608	0.351939659	0.465192583	0.490219575	0.414602293	0.521521993
0.105294355	0.335201381	0.561049513	0.362838638	0.379139323	1.004794171
0.441198564	0.433078363	0.458597374	0.440831738	0.440801183	0.894590907
0.17702193	0.346661614	0.435818698	0.33985706	0.514235103	0.570026146
0.115960069	0.298023913	0.479731807	0.368592852	0.629160499	0.507155476
0.123279088	0.208641782	0.449128445	0.366122921	0.598448913	0.658520382
0.153435051	0.211906537	0.451281643	0.296910496	0.384059036	0.76731986
0.662825035	0.251721439	0.510046257	0.332056352	0.288331427	1.063706401
0.171883147	0.203881107	0.484812903	0.392264858	0.507120324	0.41142461
0.172551629	0.463583139	0.595799988	0.579427475	0.772335719	0.921336453
0.065739304	0.260471964	0.402929684	0.323357057	0.447822835	0.454042736
0.04136344	0.251215955	0.407310243	0.355815188	0.374568115	0.508880918
0.078275249	0.152692383	0.424665849	0.285923281	0.357273298	0.677644254
0.372290317	0.200851341	0.441504485	0.269769659	0.314144451	0.542765152
0.182251172	0.334597831	0.572798651	0.536630143	0.541449991	1.186078913
0.192229279	0.245790319	0.466937059	0.415062357	0.365767805	1.347046824
0.150840945	0.313383255	0.525987347	0.416416748	0.43587912	0.759330572
0.105440425	0.273535503	0.427323333	0.351403387	0.366707074	0.723918906
0.075913485	0.23607219	0.514841409	0.312472255	0.317846036	0.49342407
0.278027598	0.336271869	0.521160627	0.36389648	0.470282646	0.760911198
0.205299213	0.222441827	0.439855025	0.336155346	0.382014684	0.768970416
0.100133735	0.208352743	0.383580158	0.352721152	0.394227412	0.468168574
0.116080698	0.287612842	0.456124636	0.398762044	0.293025881	0.825934912
0.341273429	0.278876834	0.460285213	0.46448374	0.459392752	0.698775049
0.055641302	0.262520138	0.347310998	0.399315229	0.381406147	0.607013286
0.181658402	0.26577918	0.487508744	0.384991901	0.361658509	0.60336378
0.289212137	0.373401596	0.576622885	0.493287283	0.48310209	0.495343079
0.111699916	0.190214614	0.40570416	0.354657903	0.348492618	0.527447717
0.087522636	0.327689193	0.41039925	0.443528895	0.471719125	1.832864329
0.070106888	0.370822323	0.432208695	0.386998532	0.31709785	0.768171317
0.078296954	0.251250783	0.374152936	0.33929216	0.503442878	0.636971178
0.139158409	0.31325295	0.398402885	0.305003986	0.422405346	0.771426173
0.095795387	0.229903447	0.499618914	0.325358015	0.347841025	0.411738426

P42331,ARHGAP25,ONCOLOGY	O00186,STXBP3,ONCOLOGY	O14828,SCAMP3,ONCOLOGY	Q9BQ51,PDCD1LG2,ONCOLOGY	Q9Y6A5,TACC3,ONCOLOGY	Q9UKR3,KLK13,ONCOLOGY
P42331	O00186	O14828	Q9BQ51	Q9Y6A5	Q9UKR3
ARHGAP25	STXBP3	SCAMP3	PDCD1LG2	TACC3	KLK13
ONCOLOGY	ONCOLOGY	ONCOLOGY	ONCOLOGY	ONCOLOGY	ONCOLOGY
0.666372145	0.899877172	0.620756772	0.68619951	1.110031025	0.857911063
0.632571297	0.764718139	0.553056968	0.771747067	1.381657472	0.891681259
0.896142459	0.94487802	0.461051559	0.8229633	0.833237688	1.093535457
0.776199818	1.076314727	0.509410287	0.483370053	1.427113422	0.649949711
1.08658422	0.832371804	0.705735751	0.572163749	1.32592576	0.680421201
0.83098826	0.731484906	0.590701017	0.723818557	1.421190549	1.142821434
0.792454496	0.917957967	0.304243849	0.581479405	0.851217667	0.698678185
0.676799309	1.070808132	0.372187111	0.678349182	1.181483925	0.890384263
0.72206469	0.504141282	0.307040315	0.406999803	0.676377231	0.845572287
0.783823102	0.86106855	0.365894593	0.630819866	1.072219293	0.55194637
0.873875706	0.69193096	0.41879032	1.1045816	0.930062775	0.668963777
1.104811315	0.609374063	0.907141807	0.841596144	1.297109253	0.832141053
0.737492355	0.738106039	0.399952341	0.601609811	0.968752478	0.757963352
0.679619899	0.688295508	0.235108728	0.407169104	1.046737725	0.757595675
0.811689581	0.960861234	0.275877557	0.354830026	1.097179826	0.631957744
0.724471077	0.66342257	0.443067988	0.449471018	0.746182277	0.471261588
0.980779004	0.924406943	0.8933516	0.609669805	0.876666485	0.680562706
0.917512679	0.528618932	0.460923746	0.634855429	1.08417677	0.842413231
0.844459419	0.681081807	0.382995674	0.655242118	1.29092036	0.776738025
0.592217899	0.754922264	0.354657903	0.66296288	0.802459229	0.856425703
0.726079789	0.585645683	0.61552954	0.353088074	0.663146718	0.985822627
0.862801147	0.576822762	0.608192529	0.484812903	0.800514811	0.918021597
0.811295842	0.831968032	0.513629511	0.503303313	1.038283197	0.996540263
0.784856061	0.76302382	0.352623371	0.417978315	0.739129981	0.802069968
0.825248205	0.760173164	0.39253685	0.373013565	1.637846326	0.781165424
0.846510577	0.525295088	0.426968043	0.568920905	1.097255879	0.730269053
0.907016059	0.77781556	0.459456442	0.641001661	1.026049263	0.703537876
0.660760797	0.847214978	0.505786347	0.427086441	1.004933475	0.572243073
1.168048355	0.904128665	0.362788342	0.498788463	1.00430676	0.881052565
1.279783703	0.78333428	0.33401852	0.4768806	1.246860445	0.666741762
0.664435011	0.932386486	0.56420838	0.458089055	1.080350916	0.720265147
0.861904541	0.628593823	0.373634609	0.617538077	1.044925438	0.833064439
0.441902501	0.699598936	0.289252233	0.362034728	0.56268523	0.474868512
0.965333934	0.729965406	0.326374444	0.659571058	1.122721422	1.015225351
0.936596791	0.78138204	0.63454747	0.491444362	0.737901421	0.589188019

O75493,CA11,ONCOLOGY	Q8IXJ6,SIRT2,ONCOLOGY	P48307,TFPI2,ONCOLOGY	P18084,ITGB5,ONCOLOGY	P38936,CDKN1A,ONCOLOGY	P06850,CRH,ONCOLOGY
O75493	Q8IXJ6	P48307	P18084	P38936	P06850
CA11	SIRT2	TFPI2	ITGB5	CDKN1A	CRH
ONCOLOGY	ONCOLOGY	ONCOLOGY	ONCOLOGY	ONCOLOGY	ONCOLOGY
0.798907291	0.857792139	0.435818698	0.882397128	0.611871242	0.206040521
0.846393234	0.847156255	0.475593202	0.734380675	0.774265358	0.724370651
1.042321254	0.930191718	0.672217497	0.932192622	0.756336422	0.27918629
0.775608221	0.842296455	0.491819213	0.664895722	1.61540958	0.492603915
0.979759798	1.080500695	0.551372798	0.826163942	0.992060493	0.299618788
0.980575078	0.813491964	0.427827167	0.824333482	0.807816769	0.457010748
0.739283695	0.564756157	0.391585704	0.64478943	0.825248205	0.275667291
0.700666585	0.684536797	0.43452166	0.724671971	0.731789185	0.314929323
0.939327401	0.537896311	0.332309628	0.783768773	0.465870215	0.26186588
0.769716991	0.7850737	0.491376238	0.519609603	0.966807118	0.270368691
0.711975746	0.453539466	0.407507919	0.861904541	0.621273318	0.190003777
0.656651588	1.271913007	0.630076977	0.953827313	0.533477748	0.177919926
0.680987395	0.614208337	0.298913508	0.556247943	0.670542184	0.258780354
0.824504916	0.660119902	0.355199142	0.544536251	0.676189725	0.24961906
0.651754254	0.616768077	0.224610924	0.529829471	0.498823037	0.202444731
0.748409616	0.869525354	0.278760876	0.418703244	0.831910367	0.26266575
0.865016765	1.589639911	0.615870957	0.799350422	0.429074479	0.51505557
0.825419828	0.590128075	0.583700415	1.26733682	0.628593823	0.269938002
0.905445681	0.655923741	0.489438671	0.951120476	0.701055225	0.373945519
0.724521296	0.745613559	0.427589995	0.68037404	0.708480475	0.293147773
0.751997619	0.599029933	0.461531173	0.657061356	0.490321524	0.169047431
0.823248567	1.155245925	0.570975204	0.540437616	0.726986259	0.117652183
0.735246543	0.720215224	0.465870215	0.622135183	0.702417162	0.266960833
0.562178428	0.417746602	0.228568738	0.553478814	0.57256048	0.275629078
0.932580391	0.530858769	0.505926601	0.652658404	0.496993883	0.211246596
0.817845368	0.93342111	0.452660084	0.735807354	0.562958313	0.374204808
0.605878325	0.708480475	0.323962785	0.55064713	0.853462695	0.397024516
0.889212417	1.387127114	0.391395751	0.607855369	0.746544415	0.366249831
0.855179989	1.10872379	0.430653661	0.881052565	0.547298573	0.313144403
0.515376979	1.039291243	0.401090583	0.673476728	0.685438912	0.212553806
0.89328968	0.755550452	0.354953022	0.473061612	0.512349433	0.69317907
1.136895774	0.746182277	0.524640105	0.644476652	0.528106206	0.184628861
0.650400378	0.570184213	0.328143782	0.678161129	0.731484906	0.209540352
0.800514811	0.535070168	0.281128184	0.544460767	0.789329733	0.206841847
0.707940492	1.681909408	0.562139462	0.548285794	0.809330012	0.325899716

O43464,HTRA2,ONCOLOGY	P07948,LYN,ONCOLOGY	O94760,DDAH1,ONCOLOGY	P37108,SRP14,ONCOLOGY	Q3B7J2,GFOD2,ONCOLOGY	Q13561,DCTN2,ONCOLOGY
O43464	P07948	O94760	P37108	Q3B7J2	Q13561
HTRA2	LYN	DDAH1	SRP14	GFOD2	DCTN2
ONCOLOGY	ONCOLOGY	ONCOLOGY	ONCOLOGY	ONCOLOGY	ONCOLOGY
0.480730419	1.031754693	0.667944436	0.445469944	0.066967272	0.839324152
0.496030247	1.072665309	0.796529666	0.489608327	0.081587956	0.783279986
0.54178787	1.028612792	0.685296394	0.502710595	0.126052794	0.697371833
0.564325716	0.946648012	0.69025436	0.538605176	0.080900925	1.196234308
0.396556957	1.469983539	0.708726058	0.529388955	0.075750541	0.926459618
0.42199564	0.841479482	0.757490657	0.690445765	0.073510798	1.834898169
0.356877289	0.901937894	0.51584159	0.552061156	0.088523236	1.347233577
0.461787171	0.872483649	0.523114984	0.358339752	0.064172545	0.605206757
0.566873998	0.949539552	0.368158777	0.429580377	0.089318325	0.47293047
0.388261349	0.854705908	0.488963947	0.61869488	0.09777436	0.910985502
0.654742712	0.656924738	0.507190631	0.494759735	0.178191448	0.581036218
0.593820992	1.135242102	0.544385294	0.839033314	0.069883709	1.082749871
0.485350876	0.72271563	0.5893514	0.51666462	0.803461054	0.737645729
0.458819941	0.740976657	0.462139401	0.451438073	0.077015893	0.59279287
0.423519411	0.509198474	0.426051578	0.279554214	0.070111747	0.70275806
0.693082982	0.94881584	0.381432585	0.628637395	0.076818628	0.64510236
0.525805085	1.009611326	0.56405197	0.704709223	0.069362514	0.714942935
0.485317235	0.685248894	0.676564789	0.537560859	0.219060307	0.95568031
0.466872332	1.044563357	0.618566239	0.650175005	0.084564737	0.824790717
0.492126121	1.037060457	0.470739232	0.611744021	0.076229858	1.007025349
0.465611953	0.537262854	0.551678629	0.433528877	0.075169958	0.590087172
0.489574391	0.978538148	0.575345312	0.503407983	0.058274155	0.606550638
0.48424196	0.821310701	0.459998161	0.484577727	0.072017933	0.693659712
0.340800652	0.594150368	0.357050489	0.387052185	0.07722437	0.710201346
0.501979377	0.716431164	0.574110362	0.390609786	0.083475694	0.796529666
0.43283828	0.834567127	0.587841859	0.60638249	0.126692234	0.939132094
0.389042588	0.83052759	0.56311442	0.53998828	0.055544967	0.597412778
0.490389502	1.024343795	0.484745698	0.639138279	0.120307626	0.937961102
0.526169672	0.940239373	0.627462002	0.505505958	0.096850278	1.265844333
0.477475959	1.065108203	0.650400378	0.457644739	0.08732872	0.668268603
0.428777171	0.589841812	0.520943928	0.500277336	0.084389072	0.555669901
0.443990281	0.958267271	0.5465025	0.416647723	0.080928968	1.040300267
0.398844973	0.607644739	0.344100048	0.308512318	0.08482892	0.590128075
0.352965725	0.75675594	0.617152957	0.418703244	0.120016112	0.904316693
0.53211132	0.885092413	0.593779833	0.960994447	0.362486708	0.574229757

P49441,INPP1,ONCOLOGY	Q6P2H3,CEP85,ONCOLOGY	Q2VWP7,PRTG,ONCOLOGY	Q9BSW2,CRACR2A,ONCOLOGY	O43570,CA12,ONCOLOGY	O95388,WISP1,ONCOLOGY
P49441	Q6P2H3	Q2VWP7	Q9BSW2	O43570	O95388
INPP1	CEP85	PRTG	CRACR2A	CA12	WISP1
ONCOLOGY	ONCOLOGY	ONCOLOGY	ONCOLOGY	ONCOLOGY	ONCOLOGY
0.957006083	1.170155289	0.750435509	0.61552954	0.382120616	0.715637057
1.187971314	0.850745783	0.863638821	0.484913727	0.64011366	0.995780728
1.06355895	0.735501404	0.900438719	0.686484951	0.54737445	0.955349154
1.248503616	0.778624691	0.629465843	0.468590626	0.398375271	0.413884464
1.09186917	0.858505927	0.765619776	0.819775057	0.454546565	0.666510727
1.31494276	0.565461224	0.682452244	0.553632292	0.582003607	0.894466899
0.696792017	0.804854553	0.579427475	0.681176231	0.52624262	0.698823486
0.903690086	1.043984288	0.569039221	0.626636191	0.493903124	0.933679945
0.576343173	0.869224052	0.515555626	0.484040611	0.192122714	0.652341809
0.456314372	1.08132485	0.558024357	0.675768028	0.299618788	0.616340714
0.827596816	0.879283316	0.622393975	0.893599324	0.324457181	0.598407433
1.795766784	0.998753113	0.88227481	0.710644531	0.522028327	1.273589194
0.64936431	0.862561961	0.556710809	0.760489377	0.363039895	0.689967351
0.679855478	1.093838692	0.599819363	0.526644013	0.358687656	0.567660397
0.571925842	1.044925438	0.463872427	0.505015649	0.189425177	0.375868521
0.76286517	0.750851754	0.382412079	0.736777035	0.295862755	0.402790064
0.822792187	0.845806762	0.730117214	0.67245051	0.394993276	0.770304095
1.419320102	0.769823704	0.809778924	0.303759198	0.30891889	0.666187413
0.773728864	0.804241116	0.728095703	0.593697523	0.296704765	0.515591362
0.69375588	0.649184293	0.670867612	0.339456826	0.327076497	0.674364268
0.7622309	0.626028395	0.506558221	0.635559897	0.282652233	0.74597542
0.603112901	0.821994132	0.52011408	0.635207565	0.313361534	0.486698421
0.621445595	0.748513375	0.649229293	0.672124314	0.249532563	0.456662427
0.471653736	0.885215121	0.593779833	0.538120062	0.274104896	0.574627921
0.901937894	1.045577499	0.70144408	0.332701436	0.384245428	0.70588252
0.760436665	0.641579522	0.542389066	0.791850509	0.439733089	0.662779093
0.636618064	0.993643336	0.563309614	0.541487523	0.361107427	0.502536399
0.842062954	0.802570482	0.65469733	0.762600827	0.332655317	0.898817426
0.880320031	1.100378609	0.588657346	0.689584858	0.49393736	0.836594257
0.730572827	0.705833593	0.438789224	0.331022228	0.435667681	0.522028327
0.48260006	0.939978719	0.477442864	0.7850737	0.488794515	0.508352099
0.713705107	0.790917982	0.71182771	0.690063007	0.46836332	0.935234464
0.71449707	0.834682831	0.442055679	0.394582807	0.439245679	0.667250321
0.572798651	0.778354887	0.547071005	0.420011286	0.328371313	0.631957744
0.627027229	0.962661167	0.55632506	0.50117974	0.38106262	0.463936738

P06127,CD5,ONCOLOGY	P21810,BGN,ONCOLOGY	Q9UJ68,MSRA,ONCOLOGY	P98082,DAB2,ONCOLOGY	Q9H3G5,CPVL,ONCOLOGY	P15328,FOLR1,ONCOLOGY
P06127	P21810	Q9UJ68	P98082	Q9H3G5	P15328
CD5	BGN	MSRA	DAB2	CPVL	FOLR1
ONCOLOGY	ONCOLOGY	ONCOLOGY	ONCOLOGY	ONCOLOGY	ONCOLOGY
0.823020345	0.247345576	0.410769224	0.984184022	0.506488002	0.408951029
1.12482456	0.253191346	0.383473822	0.73417709	0.734788015	0.511001701
1.08417677	0.248204297	0.391151662	0.987600861	0.466322517	0.512598087
0.737441238	0.682073917	0.372341931	1.149176185	0.802681749	0.419255031
0.945598728	0.285052587	0.612125765	1.065920617	0.599154511	0.459902517
0.826622192	0.257759946	0.374853818	0.932451117	0.292457725	0.430116684
0.849331699	0.456093021	0.321479801	0.846510577	0.60583633	0.394418739
0.853876899	0.552329083	0.2880917	0.814846378	0.469240681	0.437483345
0.764082329	0.775017074	0.295391455	0.70061802	0.541750318	0.277989057
1.195488291	1.023917871	0.230110704	0.996816601	0.2769121	0.338634296
0.818582653	0.359060784	0.250260065	1.013046221	0.44028207	0.326012684
0.926331193	0.277757929	0.580392186	0.717773219	0.343551908	0.567621051
0.756283999	0.443805669	0.268966798	0.841771167	0.557521752	0.404468709
0.604955111	0.402343603	0.218590105	0.789767552	0.524931108	0.379165604
0.398071641	0.189451439	0.242692259	0.857911063	0.284105763	0.296355348
0.701541327	0.43467228	0.323872976	0.891495858	0.168977141	0.249100531
0.788126983	0.793113915	0.433048345	0.837580639	0.558256481	0.543857276
0.778786618	0.663238656	0.319546989	0.935493802	0.403041416	0.43951978
0.679667008	0.333995368	0.475857	1.026049263	0.284322465	0.403600537
0.866937564	0.270743761	0.337766933	0.766203754	0.475428403	0.356432304
0.648060317	0.475066045	0.289533061	0.926010206	0.431221196	0.347792807
0.726180452	0.487846776	0.361934365	0.868862627	0.531816336	0.214240034
0.623387011	0.172169322	0.349605552	0.933162346	0.658931317	0.331320643
0.518997682	0.39763041	0.247431314	0.714645661	0.490593492	0.278606341
0.916940484	0.423431352	0.247499926	0.774855931	0.602319136	0.547867907
0.783279986	1.222809783	0.280446989	0.793278855	0.375712234	0.441076255
0.735705356	0.455998189	0.306529961	0.855179989	0.558798479	0.469175635
0.767639045	0.231679131	0.435848908	0.89211401	0.268929514	0.326080483
0.545291662	0.504071398	0.365818515	0.952704032	0.522897472	0.373427479
0.42788648	0.553670668	0.299909681	0.886566036	0.291506508	0.402594676
0.817165386	0.341131527	0.284736629	0.746854959	0.305405934	0.370616752
0.844166802	0.29629373	0.338165175	1.003054503	0.80457566	0.388369013
0.8525167	0.422698237	0.270537408	0.739539956	0.727742514	0.320234356
0.633536656	0.299452691	0.214581856	1.059732395	0.473094404	0.466937059
0.829204586	0.210661707	0.323895426	0.780299565	0.40983071	0.469696256

P26842,CD27,ONCOLOGY	Q9NS68,TNFRSF19,ONCOLOGY	Q9NZT2,OGFR,ONCOLOGY	Q00796,SORD,ONCOLOGY	Q496F6,CD300E,ONCOLOGY	Q8IUK5,PLXDC1,ONCOLOGY
P26842	Q9NS68	Q9NZT2	Q00796	Q496F6	Q8IUK5
CD27	TNFRSF19	OGFR	SORD	CD300E	PLXDC1
ONCOLOGY	ONCOLOGY	ONCOLOGY	ONCOLOGY	ONCOLOGY	ONCOLOGY
0.317625798	0.619682014	0.465418351	1.199805043	0.254811095	0.676002272
0.423313968	0.655242118	0.590905773	0.590619134	0.379349621	0.946648012
0.411624283	0.813153713	0.440221039	0.656014678	0.369590608	1.057677648
0.280836041	0.52011408	0.438242103	1.250842375	0.385766561	1.080425803
0.345438311	0.64015803	0.576423077	0.662595357	0.444513765	0.673896996
0.281069731	0.618737766	0.718669318	0.361357815	0.260580314	0.602694999
0.400063246	0.584712768	0.293656199	0.438363627	0.20656962	0.537300096
0.437362066	0.677785181	0.380323766	0.252980835	0.319768559	0.940891323
0.26067064	0.371903441	0.253173797	0.984525173	0.267164458	0.586539429
0.260562252	0.443682637	0.39433673	0.2545463	0.468428253	0.919613787
0.251407571	0.546199538	0.480830394	0.459042616	0.216929776	0.616639837
0.524276578	1.14480351	0.737645729	1.652442418	0.381776445	0.845220697
0.286816515	0.521738933	0.306827564	0.246114233	0.198939271	0.65161874
0.245534899	0.506909462	0.32273009	0.356432304	0.164106012	0.614549021
0.159662786	0.313578815	0.228616272	0.420710579	0.196922518	0.40584479
0.299369676	0.382518121	0.412338192	0.766203754	0.191153033	0.365438363
0.365210462	0.54699517	0.543970379	0.837522585	0.252210457	1.116899983
0.306105315	0.689871707	0.459329071	0.595139591	0.304201675	1.054895434
0.42146946	0.484678503	0.500728334	0.435275282	0.288291459	0.743961568
0.325673898	0.531742616	0.35507606	0.60667678	0.280019655	0.883988802
0.306806297	0.558837214	0.457739913	0.294655275	0.2584756	0.604703569
0.240949047	0.579990028	0.468688077	1.183286972	0.434822952	0.700375246
0.245092799	0.534551184	0.455208689	0.43915435	0.362863789	0.7040257
0.270743761	0.562724234	0.251059287	0.468201026	0.24865201	0.670867612
0.414401175	0.658018477	0.463261919	0.313470155	0.275399911	0.789493887
0.369821242	0.433468782	0.329328666	0.296622512	0.248101094	0.733160009
0.359434302	0.561711015	0.404328555	0.535701039	0.233581837	0.393735855
0.276701045	0.536146808	0.386355275	1.297289083	0.278297528	0.723467443
0.394938522	0.803962436	0.372367741	0.704318557	0.37822064	0.933162346
0.245892562	0.38966331	0.38947429	0.362185326	0.388046111	0.464676954
0.258529354	0.423783699	0.287174589	0.257420704	0.225453217	0.440099
0.357199013	0.6341957	0.408469426	0.962727896	0.257046273	0.798851916
0.267497998	0.535701039	0.311974496	0.651934984	0.255323812	0.359733395
0.315541136	0.534402996	0.315738042	0.435577096	0.20755995	0.600568204
0.330449107	0.431909214	0.436181353	1.022924736	0.263194273	0.689871707

Q9NS15,LTBP3,ONCOLOGY	P50583,NUDT2,ONCOLOGY	Q96PQ0,SORCS2,ONCOLOGY	Q8N129,CNPY4,ONCOLOGY	Q86SJ6,DSG4,ONCOLOGY	P56159,GFRA1,ONCOLOGY
Q9NS15	P50583	Q96PQ0	Q8N129	Q86SJ6	P56159
LTBP3	NUDT2	SORCS2	CNPY4	DSG4	GFRA1
ONCOLOGY	ONCOLOGY	ONCOLOGY	ONCOLOGY	ONCOLOGY	ONCOLOGY
0.56311442	1.373921903	0.666418336	0.556440757	0.827367389	0.459934397
0.733820951	1.190444201	0.737339014	0.71484383	0.65533296	0.61869488
0.776738025	1.207229529	0.907833732	0.595222101	0.45350803	0.561088403
0.636838738	0.764824159	0.553056968	0.458851745	0.930901224	0.491580639
0.700666585	1.276240304	1.082749871	0.63423966	0.441167983	0.533145051
0.432058928	1.546599873	0.443283018	0.732702783	0.898817426	0.434883236
0.668500248	0.876848802	0.465128098	0.412281034	0.500971348	0.499376557
0.577823186	1.093156533	0.484107718	0.490593492	0.487982055	0.511178832
0.540699902	0.804742985	0.627592493	0.428390977	0.367012219	0.34757591
0.748046573	1.008562158	0.409631907	0.446737727	0.507612675	0.317515736
0.598905381	0.899565352	0.550303725	0.417920375	0.438120614	0.300846609
0.795536484	1.374683978	0.861844801	0.956342967	1.012905793	0.783225695
1.721540355	3.55906924	0.482968164	0.519429552	0.448412997	0.379533727
0.340210601	0.91383145	0.608740813	0.451782408	0.641490587	0.3747499
21.38163098	0.802681749	0.454735645	0.394555457	0.381749984	0.273858013
0.754817617	0.901125531	0.523368863	0.431849343	0.528216034	0.307210621
0.635559897	1.375923252	0.70828407	0.816768991	0.50291971	0.410228605
0.579186548	1.096571589	0.651302649	0.546767729	0.779866995	0.476913656
0.646131627	1.211504699	0.715637057	0.901875378	0.683446351	0.418616186
0.686104389	0.82410496	0.419167858	0.633712334	0.308811846	0.47243901
8.804859604	0.964397623	0.409915941	0.308640652	0.345031504	0.407507919
0.724320443	0.770838214	0.683635869	0.537933597	0.368720619	0.471392268
0.541149831	0.801458653	0.676377231	0.471228924	0.560544184	0.455871777
0.528435758	1.161750286	0.542351472	0.51788368	0.418152183	0.372393553
0.423548769	0.800403843	0.525586455	0.620541671	0.507999858	0.397713103
0.432478405	1.203052832	0.409489964	0.589269704	0.542652298	0.432148782
0.554707832	1.318319449	0.519285555	0.401674839	0.596419776	0.404665006
0.914908898	0.966874134	0.478038924	0.520619047	0.706665803	0.408158102
0.88423393	0.766628746	0.715686663	0.673150035	0.608150374	0.535366956
0.56703119	1.235589914	0.656060151	0.567345706	0.680515534	0.478735269
0.70110382	1.049571172	0.641357206	0.454326071	0.655060472	0.348033962
0.881846831	1.111185748	0.779056572	0.595057093	0.823876502	0.480397317
0.542276291	0.784094802	0.416359025	0.441412687	0.615444215	0.40142434
0.653563808	0.722565361	0.572481112	0.447202451	0.591766528	0.410029609
0.647611271	0.945467649	0.531337337	0.574269561	0.54002571	0.393681275

O15263,DEFB4A_DEFB4B,ONCOLOGY	P35318,ADM,ONCOLOGY	P35052,GPC1,ONCOLOGY	Q9Y5K8,ATP6V1D,ONCOLOGY	P35475,IDUA,ONCOLOGY	P29317,EPHA2,ONCOLOGY
O15263	P35318	P35052	Q9Y5K8	P35475	P29317
DEFB4A_DEFB4B	ADM	GPC1	ATP6V1D	IDUA	EPHA2
ONCOLOGY	ONCOLOGY	ONCOLOGY	ONCOLOGY	ONCOLOGY	ONCOLOGY
0.043546483	0.190544517	0.739591218	1.119380096	1.065477406	0.969424198
0.047330042	0.293249387	0.70275806	1.359616712	1.070808132	1.16360387
0.028348181	0.252980835	0.730471555	1.350225153	0.865076726	1.618884433
0.053951243	0.186765515	0.865196659	1.015225351	0.648689504	0.705442304
0.755707581	0.31837523	0.62381926	1.181483925	1.181074524	1.022712047
0.166685441	0.312190816	0.631476084	1.152366799	0.438545975	1.14092187
0.110017124	0.206111942	0.480963727	0.986164346	0.530895567	0.707842357
0.021097268	0.208078527	0.618651997	0.981050971	0.605290662	1.213017194
0.071485775	0.138811596	0.384431911	1.095507985	0.665818102	0.550723471
0.117196387	0.126271416	0.333972218	1.309757751	0.568369089	0.642291451
0.010226101	0.188691325	0.414918532	1.015788468	1.114966219	0.550341871
0.060266496	0.313687512	1.07601635	1.235761215	1.238076105	2.02202383
0.038983299	0.111916915	0.572997202	0.992060493	0.590782911	0.87091269
0.044044326	0.101630124	0.513950029	0.894033007	0.752571208	0.775661984
0.035949113	0.080386665	0.33376394	0.98173122	0.475329551	0.501422973
0.061579758	0.142052881	0.220813457	0.929418328	0.499203516	0.471980775
0.393544859	0.163651644	0.729358491	0.898256889	0.989245155	0.892175849
0.620670723	0.216164272	0.608994034	1.098778053	0.800459325	0.869645904
0.066187381	0.20064262	0.781978041	0.951845945	0.816882227	0.885767519
0.056246222	0.243940296	0.500624222	1.138472939	0.590332633	0.810902295
0.02138881	0.139689937	0.449066186	0.622955062	0.544687249	0.87260461
0.019657178	0.263486326	0.356704173	0.979148782	0.714199981	0.786653384
0.107506813	0.143507695	0.390934822	1.075196241	0.796971478	0.881357967
0.046465379	0.119476601	0.355125288	1.033830736	0.717126731	0.683446351
0.077112043	0.170885276	0.463968897	1.292352831	0.502884851	1.265581135
0.09182304	0.168801543	0.463005103	1.558329159	0.545896745	0.683541104
0.082166835	0.200851341	0.362888942	1.057164585	0.659753955	0.673103377
0.01487878	0.146634857	0.448009118	1.192426217	0.757280665	0.770891647
0.134455437	0.173775909	0.617281303	1.059805853	0.866517025	1.124200997
0.015695557	0.181255917	0.488726758	1.354912795	0.99281719	0.600734739
0.0648927	0.180341084	0.394145443	1.147027516	0.46888304	0.465708784
0.026174395	0.164516022	0.706420933	1.305769275	0.920251435	1.027900059
0.059643139	0.112266547	0.40430053	1.09065892	0.681176231	0.571133534
0.005056658	0.182909257	0.539427135	1.046375016	0.386944887	0.645370706
0.071184157	0.1615665	0.444236549	1.044997869	0.817675319	0.737083516

Q96J42,TXNDC15,ONCOLOGY	Q5JTD0,TJAP1,ONCOLOGY	O43699,SIGLEC6,ONCOLOGY	Q15116,PDCD1,ONCOLOGY	Q8NCC3,PLA2G15,ONCOLOGY	Q9P1Z2,CALCOCO1,ONCOLOGY
Q96J42	Q5JTD0	O43699	Q15116	Q8NCC3	Q9P1Z2
TXNDC15	TJAP1	SIGLEC6	PDCD1	PLA2G15	CALCOCO1
ONCOLOGY	ONCOLOGY	ONCOLOGY	ONCOLOGY	ONCOLOGY	ONCOLOGY
0.508845647	0.803850991	0.573672792	0.585442749	0.38600729	0.49120597
1.042610287	0.867177963	0.636353357	0.667157827	0.456504188	0.569078666
0.856781955	0.896577376	0.588820579	0.805189353	0.518099106	0.79487505
0.634503488	0.908148418	0.510046257	0.51652139	0.468753056	0.49127407
0.78138204	0.852634892	0.640557506	0.59737137	0.442914459	0.581157054
0.542501864	0.859279868	0.46771448	0.456219494	0.355864518	0.499584285
0.645818198	0.859816082	0.453633786	0.522173084	0.345605959	0.598158614
0.636838738	0.813491964	0.513238037	0.463069294	0.371619986	0.465934802
0.575026361	0.843757309	0.533329857	0.476682313	0.391450014	0.625377838
0.504840655	0.787799278	0.454168641	0.654062316	0.299328178	0.627331539
0.576982713	0.827826306	0.495927111	0.417688695	0.449969774	0.465224829
0.92242275	0.987053371	0.626332219	0.70730286	0.483403559	0.589719171
0.805245166	0.859279868	0.384485208	0.635824274	0.370334279	0.652206173
0.732347359	0.933615229	0.386034047	0.332424818	0.348420158	0.473488076
0.40745143	0.979080915	0.41205248	0.264859654	0.302561432	0.40351662
0.442484861	0.85660381	0.464548136	0.460253309	0.289693657	0.620455652
0.557328564	1.005978868	0.555438852	0.768171317	0.346205368	1.465608728
0.664527128	0.924214738	0.597827016	0.993574464	0.380481971	0.560272272
0.719367058	0.904567458	0.650039819	0.451500659	0.41017174	0.448661719
0.568526696	0.851925985	0.444544577	0.501562016	0.321323856	0.376389947
0.672497123	0.866817389	0.418761292	0.40745143	0.360132573	0.327939139
0.533477748	0.897323438	0.55351718	0.350187623	0.337860594	0.931740429
0.601985232	0.895149156	0.562217396	0.547298573	0.330999284	0.451219087
0.453570904	0.817958753	0.409461582	0.372910158	0.258511434	0.493560895
0.55632506	0.849096246	0.343432862	0.814056029	0.338610825	0.409461582
0.51584159	0.83497216	0.43587912	0.448755025	0.359783268	0.515770084
0.929869394	0.833179934	0.493663539	0.443836432	0.392428031	0.580472651
0.571371111	0.79344383	0.377749042	0.363266441	0.369334516	1.09832118
0.686770512	0.868260586	0.507999858	0.61429349	0.413884464	1.035767359
0.565539619	0.983502074	0.523368863	0.373686409	0.368822864	0.587149584
0.365159837	0.898194629	0.443651884	0.433769344	0.296026862	0.563426763
0.839382331	0.793333843	0.522281678	0.510399917	0.430653661	0.618094786
0.43718021	0.858565436	0.417572903	0.409660302	0.336668349	0.50291971
0.446613882	0.785890382	0.445593471	0.427797513	0.287194495	0.480230853
0.553248677	0.998753113	0.514520334	0.489540458	0.404973665	0.968215436

P08397,HMBS,ONCOLOGY	Q16653,MOG,ONCOLOGY	Q16543,CDC37,ONCOLOGY	Q8WYN0,ATG4A,ONCOLOGY	O14974,PPP1R12A,ONCOLOGY	P41439,FOLR3,ONCOLOGY
P08397	Q16653	Q16543	Q8WYN0	O14974	P41439
HMBS	MOG	CDC37	ATG4A	PPP1R12A	FOLR3
ONCOLOGY	ONCOLOGY	ONCOLOGY	ONCOLOGY	ONCOLOGY	ONCOLOGY
1.73868912	0.825648715	1.008911759	1.025978145	0.705540106	1.138551855
1.40415286	0.761491585	1.094824785	1.03304278	0.601860066	0.029763748
2.147413471	0.495102795	1.733154179	1.116745158	0.812027223	0.020418207
1.211000953	0.873451801	1.116512962	0.947764153	0.682026641	1.47856739
1.354255547	1.102439893	0.889150784	1.219508657	0.816825607	2.380063393
1.404250192	0.321702711	1.004585252	1.210749158	0.779272602	0.019336895
1.801376817	0.423754326	0.825076617	0.877152747	0.741644646	0.020411132
0.991235663	0.488185043	0.825191005	0.8005703	0.62494451	0.017087676
1.283159044	0.541224855	1.190774308	0.884601749	0.960195447	0.014425849
1.809762022	0.442668923	0.918148871	1.379456535	0.783442881	3.402039755
1.519398182	0.506207224	0.907770808	0.801125405	0.606929142	0.015446245
3.025345688	0.709119169	1.221115784	1.539754132	0.682026641	1.678531952
1.000624027	0.494931236	0.777761647	0.777546036	0.919103986	0.017490314
1.687514619	0.571767293	0.73989887	1.450851639	0.636794597	0.014848901
1.741342511	0.480297432	0.821424566	0.988080165	0.741901725	0.011143143
2.318834732	0.478370391	0.891866698	1.111647973	0.77035749	1.001317847
4.416795389	0.681601304	1.217566019	1.798008698	0.86585659	0.025354752
1.230376625	0.662641287	1.531557997	1.211840646	0.772121612	0.018003235
1.210917015	0.678349182	1.157490217	1.077359696	0.860710516	0.021604865
1.353223372	0.494896931	0.677315539	0.971105539	0.721164357	0.019231299
0.750435509	0.31214754	1.155005723	0.752571208	0.769343613	0.017017937
1.960606485	0.558411284	1.248590159	0.8962667	0.803071307	0.018478609
1.118449409	0.496443005	0.77196107	0.838568184	0.740155344	0.015802537
1.093004999	0.356803086	1.152446677	0.729459608	0.713161141	0.013913218
1.104275387	0.511426918	0.707351888	0.754399173	0.707940492	0.024690502
1.717964232	0.498788463	0.921911392	0.896328826	0.769343613	0.016106647
1.464593197	0.609289591	0.848096303	1.189784262	0.729105758	0.025909051
4.463576029	0.522281678	1.303147149	2.539773779	0.752936446	0.01330212
2.554781662	0.491921494	1.099235117	1.74001531	0.761597158	0.019521396
0.847097537	0.512384948	1.216975395	0.727944315	0.745303531	0.018492704
2.092750033	0.616041736	0.835609037	0.940891323	0.644208678	0.014684114
1.201719343	0.691835045	0.984866443	1.0238469	0.631519856	1.526576619
1.038427144	0.382703765	0.861128236	0.783660128	0.744941995	0.014550374
1.073185896	0.508140725	1.153805464	1.007095153	0.599860941	0.019762373
3.652033502	0.600859672	1.081624698	1.831086569	0.937181253	0.022705726

P43629,KIR3DL1,ONCOLOGY	Q9Y653,ADGRG1,ONCOLOGY	Q9UHD8,SEPTIN9,ONCOLOGY	Q08AG7,MZT1,ONCOLOGY	P20851,C4BPB,ONCOLOGY	Q9P0J1,PDP1,ONCOLOGY
P43629	Q9Y653	Q9UHD8	Q08AG7	P20851	Q9P0J1
KIR3DL1	ADGRG1	SEPTIN9	MZT1	C4BPB	PDP1
ONCOLOGY	ONCOLOGY	ONCOLOGY	ONCOLOGY	ONCOLOGY	ONCOLOGY
0.115334817	0.247980743	1.034045738	0.930320679	0.309519026	0.669798941
0.211803744	0.333717674	1.163684528	0.752623374	0.320900958	0.689154806
0.493355672	0.341675806	1.076389334	0.967410432	0.340328529	0.697613566
0.257242336	0.257956553	1.045215193	0.570105174	0.385018587	0.897012503
1.266809859	0.350211897	1.201719343	0.759751751	0.54062495	0.544158938
0.119883083	0.338282394	1.215289478	0.901500377	0.312515576	0.583862274
1.116512962	0.260381707	0.941347957	0.790096075	0.458947171	0.631038529
0.401814073	0.266350889	0.939392513	0.593944487	0.295248165	0.812590273
0.287393632	0.219744658	0.834393602	0.938286231	0.385820044	0.631038529
0.203105329	0.24238965	1.150929928	0.843289561	0.292214566	0.532406467
0.189097212	0.202669374	0.96346222	0.657790465	0.321948091	0.604661656
0.377984767	0.353504381	0.993781093	0.905759539	0.376520416	0.620842833
0.356704173	0.249705586	1.06341152	1.003471749	0.555631386	0.788181613
0.096689296	0.23254793	0.97063447	0.661494011	0.19586072	0.578584669
0.504770674	0.134520691	1.018891197	0.878978633	0.189740558	0.737850276
0.235728811	0.219729427	0.87569477	0.835551119	0.248445273	0.63564801
0.180791656	0.216269181	1.272971394	0.903752727	0.44537732	0.491751037
0.268780429	0.231614905	1.134062381	0.955084312	0.357149498	0.742004582
0.395760628	0.222796736	1.145756126	0.617409677	0.390420307	0.536444194
0.318507666	0.234669134	1.066659713	0.780948869	0.306699985	0.909912675
0.281967346	0.205783612	0.874906044	0.534143765	0.303590805	0.498961359
0.243686798	0.174125572	1.093004999	0.967745769	0.594727215	0.826450318
0.202108233	0.213854282	0.886074556	0.544272104	0.322797207	0.49134218
0.485149066	0.201590562	0.887488297	0.713804054	0.322864338	0.640202404
0.289814162	0.215073249	1.042032302	0.86904332	0.332470905	0.571450326
0.069946709	0.25012133	1.036916699	0.686294644	0.362637494	0.510789226
0.205228074	0.214195489	0.992404375	0.860173744	0.312558903	0.555746938
0.259408921	0.192990268	0.971778891	0.975828822	0.307700779	0.712568197
0.808040775	0.258726548	1.005978868	0.981186983	0.348879323	0.689680461
0.473422441	0.21968374	1.153325709	0.714051482	0.315475528	0.455366479
0.583498155	0.192789716	0.822792187	1.16199189	0.336365116	0.898879729
0.234815574	0.215954607	1.078480432	1.110184919	0.241852608	0.617709319
0.223740759	0.163606276	0.98479818	0.908400245	0.258171204	0.466581173
0.132090627	0.225531367	0.950263815	0.915543285	0.246080117	0.741644646
0.574229757	0.232193582	1.051245773	0.764082329	0.307082882	0.522498934

P01242,GH2,ONCOLOGY	P42658,DPP6,ONCOLOGY	Q7Z5L0,VMO1,ONCOLOGY	Q9BSG5,RTBDN,ONCOLOGY	P29459_P29460,IL12A_IL12B,ONCOLOGY	Q99717,SMAD5,ONCOLOGY
P01242	P42658	Q7Z5L0	Q9BSG5	P29459_P29460	Q99717
GH2	DPP6	VMO1	RTBDN	IL12A_IL12B	SMAD5
ONCOLOGY	ONCOLOGY	ONCOLOGY	ONCOLOGY	ONCOLOGY	ONCOLOGY
0.309411774	1.222386063	0.494794031	1.176580475	0.590496331	0.304201675
0.597909898	1.137053392	0.349339092	1.284048771	1.378309613	0.645773435
0.462363687	0.596874697	0.623646324	0.692026889	1.020092515	0.375712234
0.92012387	0.998753113	0.600734739	1.141475583	0.744374222	0.33641175
0.413454363	1.384053777	0.621402521	1.636371142	1.10343374	0.408299583
0.37192922	0.657106902	0.471719125	0.636265146	1.30658411	0.452221033
0.501283968	0.625985003	0.494382644	0.494691152	0.638783964	0.280174974
0.390312075	0.587312399	0.529095481	0.548057816	0.585767477	0.311240132
0.701638589	0.606004327	0.397906122	0.543480433	0.732550437	0.26110464
0.355691894	0.612040913	0.766788178	0.638341346	0.705393409	0.319325573
0.384618483	0.54733651	0.306721244	0.594109186	0.477641468	0.279476716
0.467293217	0.877639279	0.762019594	0.542953292	1.081399804	0.637280314
0.329579862	0.806529943	0.386408839	0.760225857	0.3778276	0.30855509
0.185835754	0.746027129	0.465031388	0.762706553	0.642335973	0.410228605
0.31910431	0.600901322	0.19218931	0.54638887	0.616725327	0.179841764
0.56276324	0.542765152	0.601568112	0.548171793	0.579387313	0.288591357
0.301034345	0.882580636	0.664803554	0.920187651	1.238505264	0.231791569
1.558869329	0.997507781	0.500867185	0.829377032	0.571291908	0.22642419
1.887138097	0.840488509	0.386864432	1.04478059	0.771586603	0.419109753
0.406013612	0.489404747	0.599403744	0.731434205	1.160543018	0.227352063
0.240032223	0.734940826	0.38858443	0.51680789	0.856781955	0.226486977
0.447884921	0.567935894	0.397189668	0.645997282	0.847214978	0.388853869
0.244939949	0.998614666	0.348927691	0.65879431	0.786816981	0.269526681
0.452754222	0.504735687	0.343623355	0.324434692	0.241350212	0.225938182
0.303275319	0.633361027	0.634723428	0.777977319	0.812139802	0.223833829
0.549008351	0.722815826	0.517847784	0.78333428	0.844869253	0.240365209
0.522390295	0.75712321	0.361082398	0.585077645	0.713754578	0.339245128
11.97040451	0.777977319	0.222534358	0.564756157	0.630382767	0.283083585
1.125526481	0.706763774	0.294880024	0.690206517	0.625334492	0.371645746
43.03981585	0.616297994	0.684062477	0.774641125	0.356037227	0.327643769
0.426672194	0.616810829	0.310098833	0.6816958	0.393081399	0.25843977
0.198182297	1.058337668	0.403376796	0.653518508	0.775823295	0.432688295
6.015980956	0.655015068	0.48260006	0.475362499	0.464580337	0.290578527
0.433709215	0.779326619	0.518997682	0.463294031	0.637368666	0.308983135
3.317278183	0.683209528	0.332401777	0.911680359	0.758699241	0.268259283

Q7Z434,MAVS,ONCOLOGY	P07332,FES,ONCOLOGY	P31350,RRM2,ONCOLOGY	P21802,FGFR2,ONCOLOGY	Q8N5S9,CAMKK1,ONCOLOGY	Q9H8J5,MANSC1,ONCOLOGY
Q7Z434	P07332	P31350	P21802	Q8N5S9	Q9H8J5
MAVS	FES	RRM2	FGFR2	CAMKK1	MANSC1
ONCOLOGY	ONCOLOGY	ONCOLOGY	ONCOLOGY	ONCOLOGY	ONCOLOGY
0.544498508	1.365661562	0.336948498	0.696164426	0.633932	0.466710555
0.748772835	1.082899982	0.400590469	0.758383773	0.658292196	0.574588092
0.552367369	1.531876509	0.39763041	0.810115772	0.882641814	0.684821547
0.615999037	1.079003841	0.458692747	0.719167634	1.269974914	0.476021948
0.645191796	0.997023905	0.531595206	0.755602825	0.600776381	0.46612862
0.674504512	0.934262586	0.386274943	0.757858283	0.954091807	0.429044739
0.469273208	0.90062598	0.320189965	0.70740092	0.638075922	0.479931363
0.435999988	0.969222633	0.23607219	0.780407745	0.788618795	0.734380675
0.578664883	1.248157506	0.326804556	0.593985657	0.521630452	0.35060051
0.614080629	1.278010775	0.538082764	0.56703119	0.543480433	0.368337452
0.606130355	1.24841708	0.369513762	0.692170807	0.820343479	0.40323702
0.567660397	1.311847483	0.34844431	0.909660428	0.907644973	0.678396203
0.497097241	1.445531453	0.423842452	0.764082329	0.630951055	0.481330584
0.608572058	1.18213926	0.287154684	0.580834881	0.660394495	0.382995674
0.592751782	1.134612765	0.35137903	0.577382786	0.992886009	0.310615128
0.438789224	1.005351502	1.248590159	0.537039459	0.472209837	0.349508634
0.732245841	1.048262476	0.435245112	0.769343613	0.992748375	0.622825535
0.633756261	1.068139443	0.358861734	0.734788015	0.788345528	0.440221039
0.512775771	0.845865391	0.45565064	0.700423794	0.661448161	0.492057903
0.486732158	1.043984288	0.271194533	0.737952571	0.805580128	0.43467228
0.387965427	0.894218935	1.40551612	0.68060988	0.649274296	0.341770552
0.670309832	1.445431259	0.323289823	0.533958677	0.942849887	0.371130893
0.601901785	1.481439798	0.294430697	0.582124644	0.848743191	0.4466758
0.444914493	0.98268436	0.256690178	0.597868456	0.867899561	0.364982703
0.410997066	1.16247525	0.395102807	0.671193198	0.566756132	0.508281631
0.41702333	0.823705199	0.270443663	0.77067794	0.693948257	0.467876606
0.525732198	1.222386063	0.337158763	0.742930932	0.701784505	0.56557882
0.763552891	1.097940596	0.337065296	0.628288901	0.77819305	0.364653968
0.666510727	1.217903646	0.554630938	0.723266883	0.47813834	0.526571009
0.459392752	1.102745597	0.258726548	0.733973561	0.722565361	0.434732543
0.458343144	1.135556902	0.279728664	0.49270636	0.603614765	0.491819213
0.58406466	1.372779582	0.448599526	0.764559137	0.590291716	0.490049708
0.368388518	0.515198394	0.252385337	0.615060401	0.505540998	0.392264858
0.545253866	1.197478705	0.323783192	0.60663473	0.595015848	0.415407739
0.580875143	1.189619334	0.345007589	0.609796595	0.964063446	0.445995173

Q9UKS7,IKZF2,ONCOLOGY	Q9H6S3,EPS8L2,ONCOLOGY	Q9Y5K2,KLK4,ONCOLOGY	P36888,FLT3,ONCOLOGY	O00451,GFRA2,ONCOLOGY	P01275,GCG,ONCOLOGY
Q9UKS7	Q9H6S3	Q9Y5K2	P36888	O00451	P01275
IKZF2	EPS8L2	KLK4	FLT3	GFRA2	GCG
ONCOLOGY	ONCOLOGY	ONCOLOGY	ONCOLOGY	ONCOLOGY	ONCOLOGY
0.699598936	0.455808584	7.260153243	0.729358491	0.72693587	0.063742526
1.373921903	0.378037171	2.764272802	1.28761385	0.896453092	0.088492561
0.994952806	0.462139401	0.540100579	5.218011053	0.645594414	0.061154395
1.133747995	0.500728334	2.826075483	0.863219883	0.810789887	0.040450462
1.255794153	0.487643929	3.245858256	0.734278875	0.729712463	0.088027616
0.802737389	0.468201026	2.246376892	0.831449185	0.941086996	0.07163458
1.195736912	0.277873469	3.251037055	0.623387011	0.505015649	0.047428564
5.024569525	0.405901056	3.074176504	0.681176231	0.532148204	0.039113217
1.036772962	0.304412604	1.110107969	0.65533296	0.401869781	0.008376713
0.95568031	0.259624781	2.807138021	0.61501777	0.350697731	0.037856898
1.304502763	0.45009455	1.836806942	0.639404144	0.486260059	0.046610539
0.484510555	0.830009641	3.88038554	0.566795418	0.768650676	0.029671054
3.18413168	0.277046491	1.579535104	0.679384402	0.604368344	0.017767691
0.828342892	0.252700426	1.429786762	0.427530722	0.45350803	0.023472251
1.073037131	0.173655499	4.655060764	0.809049568	0.348927691	0.032417126
0.700812299	0.240381871	1.944501031	0.848037519	0.372238711	0.026687352
2.771755498	0.340895155	8.219211515	0.607644739	0.849743897	0.026036873
1.038787098	0.52188361	8.434485106	0.864357475	0.541449991	0.061048514
18.30797092	0.429878243	3.041957506	0.695296388	0.590332633	0.062365847
7.656048664	0.317603783	3.930464992	0.837058292	0.518494288	0.062847536
0.686389791	0.391993055	2.478899874	0.504420915	0.494416913	0.027003683
3.207835141	0.492774668	1.313940549	0.524967494	0.329534176	0.014620131
1.08192463	0.456630775	2.841002301	0.909156145	0.567306382	0.070472295
0.612380394	0.32273009	0.659708226	0.674364268	0.423137954	0.078655966
0.752466886	0.33420379	4.357800043	0.825763182	0.626115187	0.025040375
0.995849753	0.294430697	3.876621817	0.664711399	0.558256481	0.048546127
1.605919971	0.218681033	2.291671094	0.743394541	0.529315571	0.036602855
1.670754778	0.280039065	7.810419294	0.7944344	0.403432719	0.025721169
0.910669834	0.296807613	2.767340179	0.828515159	0.502745441	0.018866884
0.905320169	0.327144518	3.009867545	0.576343173	0.608909615	0.01803196
0.822906258	0.276873714	1.682842315	0.692890845	0.500485439	0.050164132
1.000277297	0.440678984	2.014190307	0.644566002	0.567306382	0.059210629
1.007374418	0.381697065	3.037111764	0.404665006	0.474868512	0.025460419
1.176743595	0.285111868	2.784657705	0.844283837	0.476946715	0.028811728
0.961394197	0.235043551	2.377590077	0.826507605	0.601151281	0.047927583

Q9Y5V3,MAGED1,ONCOLOGY	Q9BYE9,CDHR2,ONCOLOGY	Q49AH0,CDNF,ONCOLOGY	Q9Y639,NPTN,ONCOLOGY	P15121,AKR1B1,ONCOLOGY	Q7Z7D3,VTCN1,ONCOLOGY
Q9Y5V3	Q9BYE9	Q49AH0	Q9Y639	P15121	Q7Z7D3
MAGED1	CDHR2	CDNF	NPTN	AKR1B1	VTCN1
ONCOLOGY	ONCOLOGY	ONCOLOGY	ONCOLOGY	ONCOLOGY	ONCOLOGY
1.032684817	0.475988954	0.324704661	1.245996486	1.251102508	0.986984956
1.066733651	0.764135293	0.262574733	1.859609885	0.351842094	0.887549815
0.943830697	0.409575124	0.264859654	1.8276629	0.878430468	1.079752008
1.049498424	0.494040082	0.332816762	1.602472945	0.55290365	1.031540168
1.224166675	0.465741066	0.308448172	1.004237149	0.645504921	0.977589029
0.873028103	0.457771642	0.450125749	1.052266404	0.489811992	0.709610863
0.937376154	0.35128162	0.300804906	1.228672143	0.41942943	0.821937158
0.851158668	0.33847003	0.302477555	0.971105539	0.25191344	0.784149153
0.979080915	0.327802781	0.196813352	1.384245661	0.627679502	1.229098042
0.897696702	0.514306396	0.22642419	1.77706937	0.74046323	0.731687745
1.040372377	0.341983826	0.235712472	0.786217292	0.353185985	0.936921447
0.939457629	0.405142124	0.349169633	2.183889711	1.113421618	1.230803115
0.951054551	0.8019032	0.345989461	1.340526742	1.218241368	0.753197439
0.891001645	0.374568115	0.2249537	0.933097667	0.379165604	0.81292829
1.117751901	0.455303356	0.200211951	1.160703914	0.62563798	1.068583762
0.980982972	0.524094909	0.219653288	1.936028244	0.735756353	0.910101905
0.894094978	0.482968164	0.247757391	1.569059433	0.496374188	0.711531731
0.973936758	0.453665231	0.232209677	1.341735224	0.776146018	1.071773463
0.934975198	0.551028941	0.290457704	1.842034395	0.666233591	1.052339344
0.717673721	0.444051835	0.333324669	0.98268436	0.616340714	0.911680359
0.814168889	0.429401757	0.161331494	1.244529131	0.537374586	0.70329409
0.906890329	0.590864816	0.2761454	2.02763785	0.86465709	0.930062775
0.811014717	0.349435963	0.209889223	3.641921939	0.540812349	0.968081222
0.920698053	0.359334659	0.318838997	0.757543164	0.487542537	0.681601304
0.899752431	0.408469426	0.303086185	1.631953556	0.488963947	0.837522585
0.680232575	0.407253782	0.352281349	0.769290288	0.41302471	0.866997657
1.058264312	0.704220925	0.196309242	0.700375246	0.486091563	1.252056789
1.13500606	0.453445164	0.177169234	1.541676428	1.258931686	0.976099416
1.22196249	0.641446123	0.381406147	1.694312511	0.612805011	1.021436841
0.976234742	0.550074908	0.292356384	0.675440223	0.422639643	0.897261243
1.310211757	0.586417475	0.397354889	0.434762677	0.667250321	1.328685814
1.171291366	0.372316124	0.237417799	1.792906185	0.578664883	1.088167013
0.829032175	0.356803086	0.277584708	0.646310798	0.293676555	0.552673751
1.069472955	0.383526986	0.322439412	1.217734821	0.533182007	1.063853872
0.969424198	0.489336906	0.252840591	1.247292648	0.737134609	0.753354078

Q8IWL2,SFTPA1,ONCOLOGY	P05783,KRT18,ONCOLOGY	Q8WXI7,MUC16,ONCOLOGY	P05187,ALPP,ONCOLOGY	O75380,NDUFS6,ONCOLOGY	Q9UBM4,OPTC,ONCOLOGY
Q8IWL2	P05783	Q8WXI7	P05187	O75380	Q9UBM4
SFTPA1	KRT18	MUC16	ALPP	NDUFS6	OPTC
ONCOLOGY	ONCOLOGY	ONCOLOGY	ONCOLOGY	ONCOLOGY	ONCOLOGY
0.911743554	0.115238924	0.451406782	0.448910579	0.201422953	0.619295557
0.905257419	0.131123666	0.546843532	0.310701261	0.280797111	0.527667122
0.950527321	0.554669384	0.804408371	0.359210145	0.408979377	0.327076497
1.100760038	0.280836041	0.547298573	0.203358895	0.347238784	0.520366503
0.881480158	0.197935186	0.928581214	0.228695518	0.301870148	0.420798072
0.556055195	0.242574533	0.497372966	0.406633224	0.287473325	0.444236549
0.663008835	0.191232548	0.410769224	0.53067482	0.337743521	0.527740277
0.711975746	0.116451409	0.361458018	0.13142394	0.211334469	0.527484278
1.14750465	0.122402082	0.281225632	0.149819779	0.29257938	0.41722572
0.849802799	0.083025597	0.639492791	0.237879029	0.307061598	0.237500096
0.803015645	0.142023345	0.59225895	0.200156448	0.923894485	0.347142522
0.770090551	0.192469267	0.239417415	0.287154684	0.505225723	0.592012688
0.743755326	0.078498016	0.149508561	0.780732374	0.328075553	0.358488812
1.11049277	0.090609115	0.391802905	0.228679667	0.290296684	0.280952862
0.799516659	0.069815927	0.297239963	0.208728571	0.251337875	0.326193514
0.672217497	0.110613548	0.237533023	0.138187593	0.172097733	0.39033913
0.791685866	0.182213278	0.481664332	0.418616186	0.370899441	0.544687249
0.906387582	0.118478731	0.43431088	0.416561092	3.879847641	0.426465222
0.909156145	0.121640875	0.429491058	0.041760998	0.252053169	0.491103837
0.625204471	0.38920442	0.531890067	0.278838176	0.171407246	0.392945191
0.720065475	0.100676579	0.642068889	0.176275026	0.132035703	0.427382577
0.699259571	0.15106067	0.503791959	0.26561343	0.216599227	0.259283086
0.623127805	0.094725719	0.378115789	0.501110266	0.233403807	0.286796635
0.55198463	0.077675315	0.26814774	0.348058087	0.119866465	0.336155346
0.672636979	0.177907594	0.895211206	0.053006019	0.412910211	0.435033981
0.568723767	0.100251797	0.380719402	0.337299013	0.190016947	0.585199321
0.690780853	0.10137684	0.851335679	0.203626891	0.162487428	0.22394246
0.816203046	0.136796194	0.182655867	0.101792276	0.499792099	0.786762445
1.00751408	0.163006342	0.519033657	0.26005704	0.50792944	0.291809751
0.837754828	0.20823724	0.512775771	0.253033446	0.202444731	0.578504465
1.6545054	0.127405547	0.336878439	0.132880379	0.213794997	0.412738522
0.775393206	0.247620043	1.312666111	0.467131293	3.635868445	0.883743743
0.76684133	0.185475431	0.270443663	0.34035212	1.886484178	0.437453022
1.263477524	0.106262171	0.267924794	0.256245753	0.169787246	0.391395751
0.875573381	0.127874455	0.412910211	0.431460383	0.298127218	0.381406147

O60763,USO1,ONCOLOGY	Q8N9I9,DTX3,ONCOLOGY	O15116,LSM1,ONCOLOGY	Q9HAV7,GRPEL1,ONCOLOGY	P20138,CD33,ONCOLOGY	Q96JA1,LRIG1,ONCOLOGY
O60763	Q8N9I9	O15116	Q9HAV7	P20138	Q96JA1
USO1	DTX3	LSM1	GRPEL1	CD33	LRIG1
ONCOLOGY	ONCOLOGY	ONCOLOGY	ONCOLOGY	ONCOLOGY	ONCOLOGY
0.986232704	0.524022259	0.778462797	0.404552824	0.154384519	0.52453102
1.400071009	0.721014411	0.882702996	0.389420301	0.972317909	0.448412997
0.944550607	0.633712334	1.484832301	0.493697758	0.627897076	0.453633786
0.96132756	0.533255927	1.875532158	0.381168288	0.452064333	0.423548769
1.590521639	0.518566171	0.976031761	0.370745221	0.455177137	0.555939579
1.07117931	0.653156221	0.766256865	0.51015233	0.502536399	0.451000207
1.302695591	0.446799662	0.819775057	0.270387432	0.506347593	0.365869232
0.581721286	0.589024684	0.864177755	0.370231615	0.601901785	0.428005132
1.464085696	0.366021424	0.838917008	0.295289098	0.581277915	0.32034536
1.271824848	0.448848351	1.078256191	0.294777844	0.485451812	0.421849413
1.086207703	0.447760758	0.951318276	0.396611936	0.102913204	0.302330828
1.812147012	1.08192463	0.930578654	0.625768091	0.173727735	0.671658595
1.196648963	0.500346694	0.986506184	0.355987873	0.544838289	0.324254837
0.505611086	0.404132421	0.782900028	0.225859891	0.511639658	0.282201977
1.138551855	0.256530096	0.980846989	0.210661707	0.185642637	0.203626891
2.976052547	0.388046111	0.955746555	0.309068815	0.285665754	0.271664885
2.026794754	0.514841409	0.872665096	0.360432248	0.515126977	0.379954877
1.089147993	0.390285021	1.565583005	0.363341988	0.249965345	0.399702915
0.893971039	0.560272272	0.962394298	0.449284128	0.242843706	0.338822127
1.439033323	0.543593458	0.737287907	0.289773988	0.366885044	0.477575258
0.984934711	0.509763506	0.847743663	0.321925776	0.372574283	0.306062883
3.133988748	0.574309368	1.392908026	0.402232065	0.244194058	0.55144924
1.67098641	0.50375704	0.77938064	0.366072169	0.274789732	0.38788476
1.309031669	0.438789224	1.004724526	0.212907695	0.306360032	0.359957878
0.813040994	0.548551889	0.782140665	0.450344204	0.48454414	0.46047668
1.647067865	0.579427475	0.693515485	0.324952331	0.502501567	0.417949344
2.001109343	0.419923956	0.887119277	0.283083585	0.723417298	0.342838254
1.491227117	0.446830633	1.028470206	0.260399756	0.547564188	0.350066278
1.56384768	0.50557604	1.364620694	0.298602883	0.510081612	0.566913292
1.204721774	0.465515142	1.53378897	0.380297405	0.244295636	0.200309118
1.281292625	0.286955713	1.471002807	0.230765587	0.318816897	0.320478616
1.233792687	0.530454164	1.389436594	0.40779048	0.469956784	0.219455449
0.52507667	0.3687973	0.751737042	0.306678727	0.434732543	0.332586151
1.069250588	0.373815942	1.395130425	0.300742361	0.513522715	0.416647723
1.413429573	0.486732158	0.821253774	0.368056716	0.454924803	0.500901904

Q96SM3,CPXM1,ONCOLOGY	P35916,FLT4,ONCOLOGY	Q96EK5,KIFBP,ONCOLOGY	P09105,HBQ1,ONCOLOGY	Q9Y223,GNE,ONCOLOGY	O60828,PQBP1,ONCOLOGY
Q96SM3	P35916	Q96EK5	P09105	Q9Y223	O60828
CPXM1	FLT4	KIFBP	HBQ1	GNE	PQBP1
ONCOLOGY	ONCOLOGY	ONCOLOGY	ONCOLOGY	ONCOLOGY	ONCOLOGY
0.72271563	0.585118201	0.982888725	1.850608856	1.107878753	0.51419946
0.603154707	0.427738212	0.957802429	1.093535457	0.683635869	0.569197015
0.870490223	0.5488942	0.890260839	1.156367526	1.524039188	0.840488509
0.561399623	0.285190929	0.800403843	1.155005723	0.900813279	0.652703644
0.511497821	0.403880389	1.121710203	1.505768517	1.17096666	2.413120569
0.672730233	0.456979071	1.542852346	1.332836671	1.097103778	0.834567127
0.568723767	0.557289934	0.8525167	0.965467767	0.91770349	0.657699282
0.783931771	0.503687209	1.331451613	0.908589161	0.899503001	0.690350056
0.657927263	0.52595089	0.826335756	1.04608494	0.699889951	0.600901322
0.703928108	0.378404198	0.844635038	1.299809322	0.910732959	0.62981499
0.588167919	0.429491058	0.63327323	1.12654114	1.333483524	0.473389627
1.071105064	0.734686159	0.833526516	2.298511684	1.134691413	0.634899435
0.729257387	0.472570016	0.986027644	1.039291243	0.871395761	0.341817935
0.625507895	0.253894316	0.933032992	0.655514682	0.677973129	0.584955994
0.58743454	0.332816762	0.95323247	1.205222908	1.003402196	0.75644128
0.726734348	0.447884921	0.373194596	1.347233577	1.142108727	0.907707888
2.285484445	0.471326924	0.750435509	8.24088912	1.143772402	1.627435087
0.632176802	0.478370391	0.682357643	1.866065983	1.033257618	3.046599799
0.651528413	0.540437616	0.916940484	1.215879283	0.940043876	0.527338049
0.676611686	0.408101523	0.861247622	1.022428531	0.903878022	0.436877285
0.558140407	0.294859585	0.915797162	1.062601018	0.908274323	0.486664687
0.529792748	0.509904862	1.078929052	1.206811208	1.188218372	0.680657058
0.594438721	0.42670177	0.841421157	1.991561457	1.077658445	0.595469698
0.664987902	0.365185149	0.664435011	0.957271458	0.87091269	0.699404993
0.588249462	0.44070953	0.977589029	1.15700893	1.262952167	0.330930461
0.816089904	0.520150133	0.872665096	1.513721713	0.724923167	0.419923956
0.595180844	0.381644155	0.935623498	1.015788468	0.733719229	0.465063622
0.804631431	0.444667848	0.862382615	1.873323426	1.224506133	0.376703149
1.120932962	0.52507667	0.716133271	1.328041288	0.98951947	0.62481457
0.678302164	0.493253092	1.13241283	1.051027196	0.768917117	0.582810995
0.737032427	0.496408595	1.062159186	1.099539932	1.305226334	0.711877052
0.586702075	0.572997202	0.935104822	1.268215575	1.214615765	0.397437525
0.719117787	0.4555559	0.967745769	1.162717005	2.067231559	0.40410441
0.592423182	0.38858443	1.089450011	1.262164543	0.789603341	0.650310219
0.572243073	0.404749162	1.126775422	2.00861352	1.327489086	0.526425033

Q6UXD5,SEZ6L2,ONCOLOGY	P61218,POLR2F,ONCOLOGY	P22307,SCP2,ONCOLOGY	O95831,AIFM1,ONCOLOGY	P39900,MMP12,ONCOLOGY	P62166,NCS1,ONCOLOGY
Q6UXD5	P61218	P22307	O95831	P39900	P62166
SEZ6L2	POLR2F	SCP2	AIFM1	MMP12	NCS1
ONCOLOGY	ONCOLOGY	ONCOLOGY	ONCOLOGY	ONCOLOGY	ONCOLOGY
1.154365429	0.505540998	0.805691813	0.750123477	0.300180049	0.25564257
1.026760713	0.705735751	0.75912007	0.636176947	0.339362721	0.493868891
1.192013025	0.561088403	0.940826108	0.665080096	0.43593955	0.52507667
1.048262476	0.572084436	0.837696761	0.787799278	0.240765403	0.269938002
1.13241283	0.679196062	0.705197859	0.673010072	0.260562252	0.322215991
1.031754693	0.843698827	0.835203695	0.767585838	0.231807637	0.298913508
0.699356515	0.481898094	0.469631147	0.472537261	0.188286308	0.234003173
0.837464534	0.474769776	0.741541839	0.774587433	0.166870403	0.286121537
0.600734739	0.384138908	0.717375311	0.632615145	0.245160762	0.173198699
0.648060317	0.519033657	0.88141906	0.61429349	0.393954249	0.273649286
0.671425856	0.488218883	0.569275927	0.467973908	0.180591263	0.252262908
1.492261115	0.891248717	0.56178889	0.417312488	0.295842249	0.440221039
1.123110595	0.473061612	0.694814612	0.713161141	0.258618969	0.486192654
0.63542775	0.431550112	0.555592874	0.540812349	0.231647015	0.165144405
0.560699621	0.413511684	0.840896415	0.586011142	0.102173998	0.156756807
0.46664586	0.509798841	0.588902213	0.619553168	0.228489536	0.140642059
0.9507909	0.498650188	0.779704843	0.760436665	0.356679449	0.261430614
1.044273782	0.625334492	1.476723784	0.538530515	0.304096265	0.262556533
0.893165852	0.678255149	0.523586571	0.625291149	0.277757929	0.372083933
0.710349044	0.511887968	0.764771147	0.975896463	0.287572973	0.402343603
0.793003974	0.546691936	0.505435884	0.793828905	0.226000834	0.178574749
0.64936431	0.678302164	0.867418429	0.800403843	0.151732286	0.180967182
0.777923395	0.520222246	0.705246741	0.882825373	0.26005704	0.315519265
0.629902307	0.433528877	0.421966391	0.61221063	0.12603532	0.210253249
0.834509281	0.569473258	0.692890845	0.66342257	0.287214402	0.268743171
0.683493726	0.584105146	0.524421958	0.616468892	0.353602407	0.191047065
0.618180478	0.482131969	0.423079298	0.516056167	0.244549768	0.169975651
0.670031116	0.501631552	0.760752988	0.709020871	0.272494686	0.247980743
0.715141187	0.515627102	1.111879158	0.447884921	0.280836041	0.334365986
0.745613559	0.61339997	0.669984674	0.522245478	0.182491352	0.198718763
0.597081594	0.43080294	1.026404926	0.621574834	0.174330874	0.229425873
1.085154152	0.504840655	0.688057004	0.774104371	0.230126655	0.371491215
0.515734335	0.502257811	0.796253658	0.534588238	0.273440719	0.159751347
0.801569767	0.439458854	0.830412462	0.589188019	0.184628861	0.14860968
0.839673289	0.645415442	0.497510886	0.505540998	0.258117525	0.230190468

**Q9UKR0,KLK12,ONCOLOGY**	**P35070,BTC,ONCOLOGY**	**Q9Y5L3,ENTPD2,ONCOLOGY**	**Q6NXT1,ANKRD54,ONCOLOGY**	**P55008,AIF1,ONCOLOGY**	**Q9BZR6,RTN4R,ONCOLOGY**
Q9UKR0	P35070	Q9Y5L3	Q6NXT1	P55008	Q9BZR6
KLK12	BTC	ENTPD2	ANKRD54	AIF1	RTN4R
ONCOLOGY	ONCOLOGY	ONCOLOGY	ONCOLOGY	ONCOLOGY	ONCOLOGY
1.114966219	0.900376308	0.788400174	0.600526577	0.459042616	0.355839852
0.7040257	0.870791965	0.954885728	0.625247808	0.619467286	0.547222707
1.95789039	0.696550569	1.060540709	0.826221209	0.401285241	0.321056698
0.695440986	1.407758635	0.716778863	0.686627717	0.458629163	0.339904178
1.31084763	1.12537046	1.025693723	0.627548993	0.711877052	0.310012867
0.826393035	1.022712047	0.68448935	0.56003931	0.521269011	0.270218808
0.238986329	1.601362581	0.662365759	0.726583244	0.41302471	0.416214751
0.672730233	0.612635129	0.584955994	0.564012875	0.301431063	0.351257272
0.43976357	1.106113937	0.786980612	0.576183399	0.354756248	0.265981904
1.151568316	1.468354179	0.799073436	0.753980961	0.506768937	0.228854093
0.620326645	0.898879729	0.653020415	0.66646453	0.344004657	0.20080958
1.117906865	0.670263371	0.930901224	0.761966777	0.464129724	0.453068156
0.298789219	0.68164855	0.63542775	0.433138404	0.864417389	0.247122795
0.671239723	0.965869378	0.566952589	0.60491318	0.216569202	0.220691046
0.56420838	0.95323247	0.525222272	0.579427475	0.342363309	0.161947713
0.553363733	0.749759603	0.629902307	0.600776381	0.398817328	0.233290587
0.895149156	0.76742624	0.601526416	1.124902529	0.315016653	0.194117219
0.610854208	0.854765154	0.754974593	0.701687224	0.497821347	0.310055847
0.737134609	0.866036659	0.759593782	0.859935286	0.30245659	0.418413121
0.943961549	0.680657058	0.739539956	0.622696036	0.423167284	0.335643126
0.275323565	0.724571517	0.307402329	0.458978984	0.391802905	0.141590856
0.414746008	0.673663482	0.578704994	0.686247075	0.423548769	0.285388677
0.38858443	0.71015212	0.762389418	0.418964526	0.22543759	0.222904864
0.33662168	0.875634073	0.546350998	1.018749958	0.43951978	0.272249254
0.843464936	0.754242317	0.649454338	0.643137893	0.318022336	0.220003747
0.646893445	0.949342121	0.525805085	0.620541671	0.280913916	0.26533741
0.589882698	1.114966219	0.764612134	0.674598025	0.360907243	0.41529258
0.7040257	0.664481068	0.720864496	1.207062183	0.761174956	0.284322465
0.404440674	1.293697215	0.988080165	0.808545014	0.502048971	0.257367181
0.642113395	1.016704198	0.689632658	0.843289561	0.468655591	0.402483069
0.250572501	1.326109585	0.658566029	0.824276346	0.434100202	0.303906619
0.919167696	0.813266448	0.885521966	0.684347029	0.303780254	0.295596276
0.24556894	0.739591218	0.723417298	2.464679406	0.329876978	0.219836066
1.10030234	0.955746555	0.785182541	0.704172113	0.403684472	0.262338236
0.388099909	0.853344389	0.500971348	0.917449084	0.433588981	0.329168913

Q16790,CA9,ONCOLOGY	P34130,NTF4,ONCOLOGY	P43490,NAMPT,ONCOLOGY	P10145,CXCL8,ONCOLOGY	P40198,CEACAM3,ONCOLOGY	P08069,IGF1R,ONCOLOGY
Q16790	P34130	P43490	P10145	P40198	P08069
CA9	NTF4	NAMPT	CXCL8	CEACAM3	IGF1R
ONCOLOGY	ONCOLOGY	ONCOLOGY	ONCOLOGY	ONCOLOGY	ONCOLOGY
0.433408694	0.917385494	0.553478814	0.266037219	0.748980467	0.64792557
0.427293714	0.757438154	0.57383187	0.222827624	0.793773883	0.706763774
0.525695759	1.214784158	0.533255927	0.280466429	0.808376899	0.601818349
0.324524657	1.289847049	0.562217396	0.317251744	0.917957967	0.55194637
0.319569139	1.129903842	1.031969262	0.48036402	0.876059037	0.726734348
0.431639859	0.908400245	0.897758928	0.477343593	0.692938874	0.62981499
0.446211623	0.734889885	0.435003827	0.349823715	0.869163804	0.499896039
0.337182134	0.839906128	0.462844666	0.346373389	0.722164797	0.446954538
0.279224996	0.894156954	0.283967947	0.203034951	0.645594414	0.420215126
0.385579432	0.927551956	0.669891801	0.349702496	0.764135293	0.454263092
0.264437741	0.767532635	0.551105336	0.285013073	0.577422808	0.443528895
0.40332088	0.859160755	0.877821798	0.627244578	0.659296807	0.570223736
0.368950711	0.977927894	1.017691293	0.242810043	0.575225684	0.51359391
0.225156495	0.759277941	0.589106346	0.299681099	0.500693628	0.439306576
0.212259348	3.02807303	0.354363029	0.302876175	0.709364973	0.383633337
0.214150953	0.911869957	0.757385654	0.187180234	0.734227981	0.497752339
0.401313057	1.004376376	0.673476728	0.454137161	0.803795274	0.67324336
0.457200853	0.886320262	0.583579051	0.307423637	0.618309038	0.599611517
0.254299406	1.126619228	0.511994423	0.243146883	0.443990281	0.64166847
0.262065619	0.772549886	0.933356412	0.306381268	0.87812608	0.509551546
0.477012838	0.73417709	0.640513108	0.314253344	0.806977302	0.419225971
0.270706231	0.710546022	0.761122197	0.603154707	0.762442265	0.457739913
0.381353276	0.689919527	0.707989564	0.39172144	0.699792933	0.480463919
0.255837562	0.818072154	0.63270285	0.273706196	0.665448997	0.423959982
0.366859614	0.821481505	0.699017268	0.350430439	0.792894047	0.471130945
0.272872706	0.849861705	1.572434584	0.393845037	0.557947004	0.532997252
0.26027344	0.733007569	0.608403349	0.423901213	0.771426173	0.586986814
0.198457226	0.983911186	0.840721574	0.588616545	0.793553833	0.432388483
0.301159568	1.457504159	0.886566036	0.455871777	0.893847117	0.626636191
0.297652311	0.995573683	0.491512496	1.306131361	0.820059219	0.5829322
0.414315012	0.88313139	0.576502992	0.309390328	1.008352455	0.592998352
0.401730527	0.87206042	0.654152994	0.252700426	0.53211132	0.574229757
0.373246335	0.725425819	1.001387256	0.334041673	0.710693791	0.377434969
0.245347759	0.775500706	0.895583591	0.575305433	0.88337628	0.525805085
0.293269715	1.05270412	0.704513863	0.408271283	0.708382266	0.536518566

P05937,CALB1,ONCOLOGY	P25685,DNAJB1,ONCOLOGY	O43827,ANGPTL7,ONCOLOGY	P00519,ABL1,ONCOLOGY	Q9UJM8,HAO1,ONCOLOGY	Q10471,GALNT2,ONCOLOGY
P05937	P25685	O43827	P00519	Q9UJM8	Q10471
CALB1	DNAJB1	ANGPTL7	ABL1	HAO1	GALNT2
ONCOLOGY	ONCOLOGY	ONCOLOGY	ONCOLOGY	ONCOLOGY	ONCOLOGY
0.625247808	0.805300983	0.548285794	0.524894723	0.147143937	0.547526235
0.864297564	0.926716523	0.646131627	0.663744543	0.12648165	0.644119378
0.722465199	0.648150163	0.583538602	0.571727662	0.388153715	0.52090782
0.956608158	0.705393409	0.480763742	0.649859615	0.329671254	0.626245397
1.262864629	0.78008325	0.401090583	0.668222284	0.148805525	0.575624539
0.662549431	0.826564896	0.625031151	0.697033548	0.218347815	0.619467286
0.591930623	0.668963777	0.594850898	0.451719782	0.121228432	0.498511952
0.588534951	0.609712065	0.52220928	0.424106941	0.191790079	0.527411159
0.428836616	0.506593334	0.382332567	0.3515983	0.329808389	0.354731659
0.497959391	0.815637493	0.336971854	0.475758059	0.083765501	0.50930437
0.5302336	0.442638241	0.331573359	0.441198564	0.126806447	0.428272219
0.813210078	0.622955062	0.720964436	0.98541272	0.192469267	0.74571693
0.6341957	0.470217455	0.454515059	0.458947171	0.060404507	0.56268523
0.574747424	0.621445595	0.381036208	0.338704721	0.082612277	0.33198731
0.341557411	0.566284913	0.289573202	0.394856406	0.148898384	0.224891338
0.511746062	0.624078753	0.253261555	0.434401202	0.118060642	0.372780939
0.984047595	0.812871944	0.543706507	0.564795305	0.112632884	0.709266641
0.651528413	0.580633614	0.544838289	0.423901213	0.15793469	0.527557408
0.685201398	0.588371797	0.469761375	0.421411036	0.113565773	0.459424596
0.495892737	0.55118174	0.440160015	0.513558311	0.136568816	0.380271046
0.496821668	0.554438751	0.342838254	0.395321959	0.553440451	0.518170935
0.54062495	0.532332665	0.308961719	0.603782145	0.565186927	0.426760927
0.609923412	0.59279287	0.420943935	0.525113066	0.093823947	0.380613859
0.427708564	0.46664586	0.440099	0.418152183	0.384965216	0.442331534
0.898381423	0.458311376	0.493629322	0.465837924	0.16508718	0.545896745
0.676471003	0.745561878	0.378273076	0.336201951	0.067433066	0.523913303
0.5522908	0.635207565	0.433889627	0.457200853	0.12131249	0.520799511
0.592546385	0.97481476	0.384085658	0.384245428	0.127088025	0.500624222
0.387911647	1.072070662	0.496752798	0.501283968	0.129112447	0.507753435
0.563192489	0.77035749	0.340210601	0.533071146	0.19583357	0.423842452
0.507788631	0.768490857	0.196214015	0.412681308	0.249722895	0.375477926
0.678208138	0.561321802	0.520691225	0.506804065	0.286419178	0.61501777
0.397052036	0.517345505	0.384138908	0.51584159	0.175385332	0.485115439
0.529315571	0.511746062	0.511497821	0.455776991	0.169164647	0.576782781
1.0604672	1.119457688	0.320323156	0.612932454	0.101257453	0.46047668

O60760,HPGDS,ONCOLOGY	P30260,CDC27,ONCOLOGY	O60825,PFKFB2,ONCOLOGY	P01229,LHB,ONCOLOGY	P04626,ERBB2,ONCOLOGY	O00233,PSMD9,ONCOLOGY
O60760	P30260	O60825	P01229	P04626	O00233
HPGDS	CDC27	PFKFB2	LHB	ERBB2	PSMD9
ONCOLOGY	ONCOLOGY	ONCOLOGY	ONCOLOGY	ONCOLOGY	ONCOLOGY
0.761069442	0.691499446	0.518674015	0.054082288	0.492262587	1.083050114
0.575385193	0.626940311	0.518889771	0.065050322	0.446861606	1.189949212
0.745872013	0.720115387	0.520582962	0.042792447	0.479565573	1.41735387
0.832544908	0.601109614	0.460444763	0.063826529	0.422054146	0.981459064
0.605962323	0.688772764	0.980846989	0.052298015	0.448848351	1.073409082
0.594191553	0.679996865	0.575784158	0.035906778	0.385312261	1.353598617
0.391504284	0.594809667	0.374464277	0.040296546	0.353112549	0.925689331
0.599694647	0.604703569	0.311844777	0.049696911	0.352818961	0.609585292
0.511675124	0.692266769	0.382650715	0.052829955	0.313274664	0.517811891
0.825992164	0.535366956	0.463229809	0.060942817	0.335108457	1.216638025
0.679667008	0.721214346	0.594273931	0.043724933	0.345031504	0.73153561
0.850215226	0.674831863	1.048771219	0.050021773	0.624338354	1.675393522
0.511604195	0.729307937	0.317075871	0.053522896	0.460317118	1.076463946
0.541825426	0.787744674	0.376311687	0.052457759	0.311736718	0.927744855
0.432688295	0.607897504	0.233500897	0.059206525	0.261068446	0.579748867
0.52453102	0.655196702	0.733668373	0.094162733	0.296232123	1.009541348
0.629509476	0.707744236	0.82742474	0.063262746	0.411082539	2.11696879
0.68558146	0.952307896	0.259624781	0.060845737	0.403264971	0.856306986
0.796143281	0.701492702	0.788454824	0.098372577	0.439976996	0.848213882
0.425549836	0.685819105	0.467682062	0.286617777	0.37579037	0.851630781
0.567935894	0.778031246	0.352208102	0.063192625	0.38966331	0.518925739
0.506101972	0.634767425	0.507260947	0.323245009	0.386248169	1.03907515
1.114966219	0.427560357	0.430564118	0.062869321	0.349193837	0.715240334
0.486125258	0.452346433	0.29754917	0.086419458	0.295739735	0.548970298
0.728196645	0.70305039	0.386944887	0.064632338	0.354657903	0.61221063
0.457295935	0.553939376	0.478934411	0.044895044	0.466419496	1.048989328
0.47976506	0.436604831	0.506768937	0.048589891	0.354436725	0.774211692
0.791356681	0.616597096	0.606929142	0.298582186	0.386623169	2.066372
0.562919293	0.730674113	0.425638336	0.165018537	0.367444943	1.527529244
0.551105336	0.883192607	0.322841959	0.040604966	0.44614977	0.67581487
0.739591218	0.50145773	0.299141505	0.035319286	0.231823705	1.040083965
0.545480678	0.542464262	0.46577335	0.061460359	0.414516088	0.928259448
0.624381631	0.477939529	0.259624781	0.05097381	0.291688416	0.709610863
0.582326428	0.609543041	0.284322465	0.161230881	0.236334147	0.659525342
0.581318207	0.59147947	1.192343567	0.049056904	0.421849413	2.126086066

Q8N386,LRRC25,ONCOLOGY	P15514,AREG,ONCOLOGY	P09486,SPARC,ONCOLOGY	Q9Y2Z0,SUGT1,ONCOLOGY	P49767,VEGFC,ONCOLOGY	O75695,RP2,ONCOLOGY
Q8N386	P15514	P09486	Q9Y2Z0	P49767	O75695
LRRC25	AREG	SPARC	SUGT1	VEGFC	RP2
ONCOLOGY	ONCOLOGY	ONCOLOGY	ONCOLOGY	ONCOLOGY	ONCOLOGY
0.48169772	0.240498533	0.648779437	0.680845803	0.317251744	0.75691332
0.693659712	0.315147692	0.651392945	0.901937894	0.262738587	1.114966219
0.697371833	0.360682167	1.146391645	0.873270191	0.485889446	0.979488188
0.475362499	0.240631931	0.443897965	1.079303045	0.463454624	1.02484093
0.65012994	0.276317722	0.505540998	1.0977884	0.492296709	0.851453708
0.319015848	0.44319085	0.728045237	0.714051482	0.453382308	1.070437082
0.45015695	0.394063491	0.446242553	0.734126202	0.452848379	0.947238746
0.367470413	0.246677836	0.70275806	0.729864219	0.326374444	0.833179934
0.377775226	0.193391997	0.493560895	0.931288456	0.389231399	0.70637197
0.290457704	0.220767545	0.755026926	1.106497353	0.553133644	0.710447526
0.306955197	0.221856696	0.647745952	0.775661984	0.418877414	0.752832074
0.674504512	0.321747312	1.404736952	1.131236049	0.553095305	0.934262586
0.468298395	0.226282983	0.821310701	0.62981499	0.329191731	0.835203695
0.27325125	0.171039329	0.571173123	0.717623978	0.325019909	0.76955695
0.329716959	0.158625872	0.504316034	0.856010264	0.30317023	0.810115772
0.33049492	0.197031746	0.431580025	0.737083516	0.371671508	0.896515232
0.464934697	0.191803373	3.413850782	1.132177376	1.483083679	0.905508444
0.393054153	0.221211762	0.602694999	0.911617168	0.474177792	0.91092236
0.481097097	0.196200415	0.56268523	0.73321083	0.528179422	0.86609669
0.432418455	0.213691288	0.673710178	0.730724761	0.527264949	0.884601749
0.289713737	0.240132071	0.621790293	0.71449707	0.481864693	0.798907291
0.486192654	0.257385021	0.83058516	0.670170459	0.41847113	1.048044518
0.510612231	0.325470795	0.857554342	0.785672517	0.307956824	0.74675143
0.277584708	0.238341156	0.352354611	0.580231289	0.328690121	0.789712811
0.631257269	0.200434116	0.201898206	0.801569767	0.383022222	0.989382303
0.42552034	0.304856033	0.517130392	0.659936903	0.542351472	1.002151066
0.524785586	0.167159818	0.337907435	0.745303531	0.429997447	0.845455074
0.308170357	0.200531391	0.365134527	1.256229453	0.501353466	0.961727448
0.476913656	0.268854961	0.789275023	0.780299565	0.348685917	0.933291719
0.431999037	0.307231916	0.406689599	0.734533401	0.342434508	0.923382311
0.253999929	0.202669374	0.377147299	0.718420289	0.432658305	0.733109192
0.48256661	0.329602708	0.622998243	0.925561012	0.393681275	0.810621306
0.350527612	0.256334577	0.468038788	0.500624222	0.420448208	0.737134609
0.415955183	0.14563207	0.349872214	0.675206174	0.341841628	0.897821158
0.344052349	0.260815226	0.549427109	1.253619912	0.517524834	0.910796088

Q15797,SMAD1,ONCOLOGY	Q86SF2,GALNT7,ONCOLOGY	P06870,KLK1,ONCOLOGY	O00622,CCN1,ONCOLOGY	P12931,SRC,ONCOLOGY	O00548,DLL1,ONCOLOGY
Q15797	Q86SF2	P06870	O00622	P12931	O00548
SMAD1	GALNT7	KLK1	CCN1	SRC	DLL1
ONCOLOGY	ONCOLOGY	ONCOLOGY	ONCOLOGY	ONCOLOGY	ONCOLOGY
0.953298545	0.646042061	0.234604079	0.037856898	0.715885121	0.411709887
1.171210181	0.821994132	0.201116033	0.162431124	0.76164995	0.549274796
1.149654213	0.722815826	0.283574557	0.103981588	0.659936903	0.549350947
1.061570364	0.630863593	2.888659186	0.06164809	0.76774547	0.422844758
0.922998367	0.78333428	1.737845708	0.254193668	1.602584024	0.498339211
1.153565561	0.731839911	0.306529961	0.168813244	0.688486371	0.466581173
1.110184919	0.514984173	2.744798038	0.017888795	0.705197859	0.505295767
0.776953413	0.427827167	0.234409022	0.018765155	0.565304466	0.457454449
0.907456253	0.329488496	0.152978414	0.011016426	0.619682014	0.327984604
1.111570922	0.476781446	0.09103089	0.026826451	0.680279726	0.355248386
0.814281765	0.432268616	0.317317722	0.043371766	0.633404929	0.372780939
1.07997656	0.703635414	0.271044192	0.028344252	0.818923163	0.582164995
0.851630781	0.769610293	0.404805276	0.080470288	0.748461493	0.329945581
0.896639524	0.314035595	0.189793173	0.039519267	0.445562586	0.310916697
0.929676053	0.298189218	0.141993815	0.023129854	0.679337312	0.2382916
0.899690067	0.296828186	0.842530022	0.008023792	0.619252632	0.346805816
0.789001528	0.768703957	2.963701195	0.028996046	1.382136401	0.44614977
1.078031998	0.72517445	2.858978954	0.082474961	0.791466394	0.340281353
1.084402241	0.55015117	2.283267673	0.121396607	0.609036248	0.386274943
0.816203046	0.550990748	0.209438707	0.064266023	0.574548266	0.389231399
0.951318276	0.568329694	0.286896048	0.021044689	0.706029319	0.418558157
1.074227828	0.473422441	0.068001011	0.024533553	0.694621995	0.340493698
0.785454712	0.422903381	0.124697116	0.055176586	0.745148566	0.359932929
0.921975296	0.248496941	0.234262835	0.056226732	0.538493188	0.296088426
0.858148959	0.594521134	0.239317865	0.029323477	0.707646129	0.434461427
0.791740743	0.589760049	1.836425029	0.027005555	0.661264794	0.393244911
0.845806762	0.514770042	0.181847374	0.012709931	0.73499177	0.464805808
1.009191528	0.487846776	0.285645954	0.018408297	0.683730648	0.369001862
1.16384586	0.631957744	0.296252657	0.041891461	0.727490342	0.458724542
0.690828736	0.414803508	0.200879187	0.057134263	0.527630548	0.311736718
0.812646599	0.358463964	0.214284589	0.043843294	0.49875389	0.380086582
0.966941155	0.601776636	0.178649032	0.053186356	0.668963777	0.442300875
0.800071034	0.290055323	0.173932568	0.017435844	0.550113038	0.302624354
1.075792622	0.556980992	0.126973558	0.013050962	0.609500792	0.285111868
1.25962998	0.598490395	1.968504486	0.033084516	0.741439047	0.360032737

O60907,TBL1X,ONCOLOGY	Q8NBZ7,UXS1,ONCOLOGY	P41586,ADCYAP1R1,ONCOLOGY	Q16674,MIA,ONCOLOGY	P07949,RET,ONCOLOGY	Q8NEZ2,VPS37A,ONCOLOGY
O60907	Q8NBZ7	P41586	Q16674	P07949	Q8NEZ2
TBL1X	UXS1	ADCYAP1R1	MIA	RET	VPS37A
ONCOLOGY	ONCOLOGY	ONCOLOGY	ONCOLOGY	ONCOLOGY	ONCOLOGY
0.646669288	0.434732543	0.971846252	0.927873476	0.696985235	0.947829849
0.302960161	0.495823996	0.909345219	0.669752516	1.470391162	0.912375746
0.637545406	0.476021948	0.886074556	1.02484093	0.967142246	0.797302999
0.341746863	0.461851193	0.709906043	0.678490255	0.752258287	0.911743554
0.859101205	1.474167032	1.136895774	1.336537207	0.914211581	1.133433697
0.497441922	0.489845944	0.87091269	0.788345528	1.112187479	1.00793318
0.516270833	0.355223763	0.64792557	0.689202576	0.595139591	0.966472106
0.237912009	0.358314914	0.639625783	0.911174956	0.738566638	0.887488297
0.529095481	0.23266079	1.073557899	0.655196702	0.564521331	0.789165614
0.552712061	0.48256661	1.150052722	0.516628809	0.563778357	0.869766471
0.499376557	0.670681634	0.74277646	1.05797094	0.679667008	0.7622309
0.404244486	0.520186189	1.122410181	2.065512799	1.068805991	0.970029145
0.662457589	0.355051449	1.24616923	0.756703488	0.94487802	1.009751298
0.419284092	0.338986565	0.656515055	0.68339898	0.740309271	0.872967591
1.048843917	0.331711285	0.945992073	0.596667872	0.855179989	0.934975198
0.516915368	0.27804687	0.929804943	0.601443033	0.301535549	0.77938064
0.859816082	0.569473258	0.972789795	0.844986384	0.788946841	1.003958754
1.437039781	0.594933367	0.898443696	0.937246215	0.682878113	1.330713504
0.460157612	0.430593964	0.938936827	0.924983798	0.720265147	0.72981363
0.523622864	0.273895981	0.869525354	0.798353722	0.870791965	0.932968321
0.920634237	0.326781904	0.614080629	0.685866644	0.961394197	0.794489468
0.918148871	0.406407801	0.962260891	0.790315167	0.737287907	0.965333934
0.352525616	0.348009839	0.657106902	0.927873476	0.876484206	1.616417639
0.513202463	0.461211375	0.586783415	0.621101088	0.654742712	0.796971478
0.502397085	0.479499096	1.981097659	0.747994724	0.837290406	0.970298132
0.295473367	0.559224704	0.79708197	0.599860941	0.817505306	0.829377032
0.558449991	0.435818698	1.030825406	0.426672194	0.513273613	0.79940583
0.421411036	0.846627937	1.135399491	0.90909313	0.914084853	0.963128367
0.629378587	0.411282046	0.927680551	0.823819397	0.790369949	1.002637431
0.450718946	0.456947397	1.535916728	0.713606173	0.607518396	0.993781093
0.419400359	0.263925015	0.844635038	0.422932696	0.353406382	0.601568112
0.732601216	0.391938717	1.30025988	0.795095467	1.074823671	0.820912295
0.225953843	0.290618812	0.859577723	0.525586455	0.578063546	0.754190038
0.457359334	0.258636895	0.959862726	0.539277594	0.785128119	0.830009641
0.659433918	0.663238656	0.992954833	0.71449707	0.548704001	1.170885498

P51580,TPMT,ONCOLOGY	Q9HAV5,EDA2R,ONCOLOGY	Q9GZY6,LAT2,ONCOLOGY	Q9H4F8,SMOC1,ONCOLOGY	Q92982,NINJ1,ONCOLOGY	P13521,SCG2,ONCOLOGY
P51580	Q9HAV5	Q9GZY6	Q9H4F8	Q92982	P13521
TPMT	EDA2R	LAT2	SMOC1	NINJ1	SCG2
ONCOLOGY	ONCOLOGY	ONCOLOGY	ONCOLOGY	ONCOLOGY	ONCOLOGY
0.671007129	0.435546905	1.060614223	0.103758397	0.816316204	0.649859615
0.552635444	0.433438737	0.923830447	0.206669873	1.109415664	0.687294348
0.619252632	0.318772703	0.963862996	0.200586998	0.82857259	0.646893445
0.649724494	0.462876749	1.382328019	0.174077301	0.915987617	0.960461706
0.475725083	0.458851745	1.591845148	0.321591237	1.71463322	1.199139914
0.458406689	0.353602407	0.829434522	0.169763711	0.77196107	0.560155779
0.632834431	0.357050489	0.95707242	0.139013799	0.89001404	0.449128445
0.613740205	0.394938522	0.704904637	0.137204528	1.256142381	0.460859853
0.841479482	0.263723859	0.754399173	0.118396636	0.965267025	0.36645298
0.947370071	0.240148716	1.259193501	0.119476601	1.080276034	0.383261238
0.635383707	0.351866483	0.947173091	0.123338918	1.074898175	0.527703698
0.769236967	0.518422414	0.993161334	0.212038773	0.874784765	0.552214241
0.588983858	0.363442742	0.853048693	0.169681361	1.109108111	0.586254908
0.464805808	0.322886718	0.730117214	0.123527144	0.664342907	0.479034013
0.596295767	0.185565446	0.839324152	0.073617879	0.784366595	0.385820044
0.589188019	0.320589704	0.895273259	0.099063678	1.083275352	0.346397398
1.410102465	0.299162241	1.044201401	0.164607275	1.069324705	0.703099123
0.875816175	0.324007699	0.826736794	0.122503935	0.780948869	0.764029368
0.4118526	0.286101705	0.976911651	0.164310889	0.860292998	0.78333428
0.824447767	0.372290317	0.99660934	0.160918266	1.804876346	0.495583479
0.778732639	0.275992315	0.646131627	0.125799666	0.676517894	0.379270745
0.676564789	0.28751318	0.945009017	0.122571885	0.943961549	0.544309832
0.492911314	0.312017748	0.750123477	0.11539879	1.104964485	0.586376829
0.425697346	0.367903677	0.43283828	0.13439953	0.800459325	0.35732283
0.706127202	0.453256621	0.794049032	0.105308953	0.859756486	0.513985655
0.662825035	0.470706604	1.105424122	0.124145167	0.607518396	0.453885404
0.494177078	0.30510971	0.679949733	0.108555144	0.785345833	0.431460383
1.454174095	0.224501968	0.847626149	0.090345716	1.209239491	0.424283359
0.925112036	0.513843168	0.819434193	0.147409356	1.028684092	0.493903124
0.58743454	0.317251744	0.817448643	0.097402324	0.758541491	0.397354889
0.847567398	0.343028417	0.793058942	0.129157202	1.275886504	0.42791614
0.619123875	0.370052021	0.78561806	0.145410162	1.051172909	0.596833326
0.547564188	0.297941295	0.548095806	0.110139205	0.727137447	0.33376394
0.689584858	0.278316819	0.827711553	0.076097876	0.846862704	0.499792099
0.966003285	0.348009839	1.239879572	0.127202594	1.148459515	0.724772439

P54727,RAD23B,ONCOLOGY	P32926,DSG3,ONCOLOGY	O43240,KLK10,ONCOLOGY	P09960,LTA4H,ONCOLOGY	P20472,PVALB,ONCOLOGY	P21589,NT5E,ONCOLOGY
P54727	P32926	O43240	P09960	P20472	P21589
RAD23B	DSG3	KLK10	LTA4H	PVALB	NT5E
ONCOLOGY	ONCOLOGY	ONCOLOGY	ONCOLOGY	ONCOLOGY	ONCOLOGY
0.698290863	0.365134527	0.310206323	1.152047339	0.493766204	0.484174834
0.745045273	0.442822367	0.343551908	1.434750622	0.521341279	0.285210698
0.802347993	0.473816387	0.532997252	1.245651071	0.300200857	0.303275319
0.805468458	0.362888942	0.310184822	1.348915522	0.703732965	0.285329338
0.759014841	0.301702802	0.298582186	1.519082265	0.801180937	0.408073237
0.665448997	0.437604658	0.248910673	1.421092043	0.535812447	0.26986317
0.560155779	0.470445661	0.356704173	0.937051341	0.353137026	0.287692596
0.660028396	0.494211333	0.386248169	1.920923413	0.402204185	0.241919673
0.611616825	0.27939924	0.315147692	1.045867435	0.436453542	0.314580249
0.917894341	0.309841007	0.393926943	2.050534476	0.359658598	0.215745145
0.608361179	0.276566822	0.314907495	1.505872892	0.374957764	0.283436999
0.887611337	0.662503509	0.691739143	1.423063461	0.660577621	0.457993808
0.559806444	0.313361534	0.361032345	0.728600555	0.41989485	0.187413918
0.778139111	0.326510208	0.428272219	1.845996733	0.182719182	0.151711252
0.524349263	0.167891372	0.328781266	0.580110646	0.289312387	0.199962309
0.59857337	0.278529106	0.508387337	1.195156877	0.455903377	0.250346814
0.853166959	0.409575124	0.549274796	1.437239011	0.691355667	0.230765587
0.996885697	0.403292925	0.400673778	0.54329211	0.401257427	0.288811481
0.680185426	0.389987557	0.438090246	2.147562324	0.345342549	0.459551993
0.545405064	0.459870641	0.260075067	1.076837085	0.208165083	0.217215657
0.569552209	0.187024606	0.360707169	2.740995577	0.407169104	0.295125401
0.819036698	0.219698968	0.449377563	1.791663867	0.461755164	0.259480854
0.642603169	0.304687031	0.605752349	1.324456077	0.410997066	0.204844349
0.664849636	0.261448736	0.315869381	1.387800316	0.2329997	0.226110517
0.676377231	0.267980513	0.278973502	1.745814167	0.449938586	0.223771778
0.468103676	0.339245128	0.286935823	1.200720197	0.59139748	0.273042986
0.674504512	0.349024448	0.293798717	2.8683081	0.38969032	0.215595654
0.734838948	0.308020868	0.444113398	1.050954347	0.370052021	0.190716292
1.053288025	0.476682313	0.395458991	1.976982362	0.425284447	0.259139348
0.550608963	0.398927919	0.515913105	1.011572695	0.258206997	0.304539232
1.268127672	0.485586427	0.425048684	1.490193835	0.3898254	0.169141197
0.604787405	0.364856232	0.489506526	1.409516142	0.504525817	0.159309036
0.521377417	0.503128912	0.367368543	0.562841261	0.363039895	0.174077301
0.692698762	0.435577096	0.335154916	1.586447743	0.278915497	0.160506096
0.676752398	0.277334692	0.396007594	2.199384743	0.623689554	0.316834205

Q8WWY7,WFDC12,ONCOLOGY	O95721,SNAP29,ONCOLOGY	O15123,ANGPT2,ONCOLOGY	O95274,LYPD3,ONCOLOGY	Q07960,ARHGAP1,ONCOLOGY	Q96D42,HAVCR1,ONCOLOGY
Q8WWY7	O95721	O15123	O95274	Q07960	Q96D42
WFDC12	SNAP29	ANGPT2	LYPD3	ARHGAP1	HAVCR1
ONCOLOGY	ONCOLOGY	ONCOLOGY	ONCOLOGY	ONCOLOGY	ONCOLOGY
0.32789368	0.333047533	0.43642329	0.419720257	0.835666959	0.156257788
0.380086582	0.338704721	0.413827091	0.49134218	0.915543285	0.159474759
0.408073237	0.40228783	0.842004588	0.457200853	0.923958526	0.187219161
0.200628713	0.397740672	0.406238816	0.475857	0.751528645	0.103120281
0.37030861	0.496890547	0.611108308	0.304096265	1.084101623	0.11354216
0.69433317	0.448226546	0.39052857	0.425254969	0.641001661	0.231101736
0.405310653	0.313470155	0.763923459	0.600776381	0.820627838	0.168240856
0.443713391	0.277757929	0.467422796	0.617452474	0.812871944	0.101559704
0.238622171	0.468558147	0.319569139	0.359608743	0.70730286	0.108442336
0.549808075	0.416041688	0.746958502	0.432238654	0.756651039	0.151102559
0.365590377	0.317757922	0.337158763	0.34992072	0.720265147	0.058924033
1.254402205	0.729156297	0.564677871	0.46904557	1.085981856	0.133424915
0.591643486	0.298520104	0.396447024	0.373401596	0.581277915	0.093947592
0.552750373	0.291486303	0.335410556	0.439276127	0.740925299	0.051696127
0.299951261	0.350527612	0.283712181	0.228647968	0.766522476	0.066638512
0.598241542	0.33847003	0.357967372	0.393981557	0.8962667	0.069603323
0.355051449	0.539165466	0.666834199	0.426287897	0.873512347	0.15540448
0.381036208	0.430176315	0.483939968	0.556363623	0.710743054	0.14325923
0.6029875	0.382624192	0.434491543	0.667389086	0.733719229	0.152586581
0.794599615	0.393763147	0.329854113	0.516270833	0.571212716	0.10444389
0.355938526	0.316132223	0.275438092	0.418413121	0.729914811	0.08884904
0.564951921	0.426199262	0.332447861	0.307210621	0.978809494	0.094587936
0.285013073	0.276835334	0.38947429	0.381406147	0.563426763	0.08969678
0.352647814	0.240365209	0.376285604	0.44168814	0.709168323	0.099752721
0.319768559	0.253331784	0.410513053	0.338869101	0.614975141	0.32273009
0.450718946	0.441779996	0.543781886	0.449813854	0.667898139	0.166281549
0.290679251	0.298789219	0.309390328	0.438910899	0.773943417	0.164436218
0.473061612	0.459042616	0.264382758	0.415350156	0.97874165	0.204433
0.670495707	0.429044739	0.509622189	0.441841244	0.916178111	0.200253588
0.445099566	0.357347599	0.325628753	0.405114043	0.65925111	0.145168465
0.575983744	0.326057882	0.381485466	0.477045903	0.73153561	0.123638503
0.395980145	0.342031238	0.567739097	0.415378946	0.718818777	0.102813385
0.61595634	0.263322006	0.310055847	0.387025358	0.594315124	0.120876023
0.58394322	0.237138202	0.37659872	0.423724954	0.789110915	0.125512243
0.416849931	0.682357643	0.662687219	0.404861398	1.058190961	0.215013627

Q14512,FGFBP1,ONCOLOGY	Q8TD06,AGR3,ONCOLOGY	Q02763,TEK,ONCOLOGY	P05089,ARG1,ONCOLOGY	O75787,ATP6AP2,ONCOLOGY	Q9P0V8,SLAMF8,ONCOLOGY
Q14512	Q8TD06	Q02763	P05089	O75787	Q9P0V8
FGFBP1	AGR3	TEK	ARG1	ATP6AP2	SLAMF8
ONCOLOGY	ONCOLOGY	ONCOLOGY	ONCOLOGY	ONCOLOGY	ONCOLOGY
0.304096265	0.495102795	0.528399131	1.249455916	0.504840655	0.419662076
0.229076282	0.701492702	0.511391469	0.734533401	0.470869767	0.491410299
0.223911418	0.520980038	0.579066122	0.837638698	0.512065405	0.38151191
0.414573556	0.646669288	0.389123496	0.632264446	1.204220849	0.414228867
0.316373354	1.424741317	0.55015117	0.921208737	0.58394322	0.500901904
0.309261683	0.650806245	0.47083713	0.870731608	0.552750373	0.255731184
0.338024565	0.218317548	0.534736478	1.199721881	0.50117974	0.401062783
0.236858935	0.507788631	0.443959507	0.639049682	0.523949619	0.520005937
0.242995247	0.441902501	0.416532219	0.742158893	0.451531956	0.674831863
0.218696191	1.071476345	0.362637494	1.690324225	0.497614352	0.669613259
0.33401852	0.646893445	0.495549129	0.556787991	0.305766021	0.316724418
0.266960833	0.312515576	0.583659958	1.510367891	0.519609603	0.666325957
0.210311552	0.336645013	0.365185149	0.845982661	0.401034984	0.303717091
0.218438642	0.401313057	0.39359942	0.908337282	0.745561878	0.33049492
0.176482862	0.297755487	0.286856278	0.914908898	0.536072488	0.350503316
0.20495797	0.257295833	0.374931774	1.136186763	0.621661009	0.425992519
0.371645746	0.448723921	0.504665721	2.633898955	0.647790852	0.408441114
0.342600699	0.348033962	0.448848351	0.736062409	0.469793937	0.329671254
0.292985262	0.499584285	0.394500764	0.98268436	0.499411172	0.436332548
0.266184782	0.518709968	0.402148432	0.779921053	0.500138649	0.48454414
0.20290833	0.46616093	0.407847015	0.500346694	0.418935487	0.200559193
0.192629425	1.010451446	0.456979071	1.20380357	0.62546454	0.439215234
0.184654458	1.183286972	0.43548653	0.746182277	0.596957447	0.312645574
0.197168365	0.449564493	0.240032223	0.86160588	0.402622583	0.365818515
0.146889176	0.554938576	0.370539693	0.603991436	0.460189509	0.364704524
0.271815569	0.39025797	0.489370825	1.245046824	0.27783495	0.298437348
0.218696191	0.366783336	0.441045683	0.735042717	0.518817842	0.419400359
0.205256526	0.618651997	0.336551689	2.173771053	0.53122686	0.278606341
0.294104344	0.239699699	0.404636958	1.470187336	0.750383495	0.556055195
0.200670437	0.414688516	0.375816418	0.522643822	0.401368694	0.390420307
0.247911998	0.428361285	0.386864432	1.856004232	0.43431088	0.50662845
0.206698525	0.4555559	0.495892737	1.282714412	0.476781446	0.403852395
0.253595315	0.481530805	0.282652233	0.810789887	0.453696678	0.390853537
0.161119163	0.645281245	0.357124743	0.695971435	0.665172302	0.386060806
0.235467524	1.054602996	0.360757177	2.131102523	0.623948993	0.414861016

O43524,FOXO3,ONCOLOGY	Q6BAA4,FCRLB,ONCOLOGY	P50120,RBP2,ONCOLOGY	P18627,LAG3,ONCOLOGY	Q96NB1,FOPNL,ONCOLOGY	P35968,KDR,ONCOLOGY
O43524	Q6BAA4	P50120	P18627	Q96NB1	P35968
FOXO3	FCRLB	RBP2	LAG3	FOPNL	KDR
ONCOLOGY	ONCOLOGY	ONCOLOGY	ONCOLOGY	ONCOLOGY	ONCOLOGY
1.031540168	0.293554444	0.201898206	0.449751501	0.240365209	0.494245591
1.868136664	0.564560462	0.241835845	0.768970416	0.577863239	0.511639658
0.73989887	0.452095668	0.261720711	0.688772764	0.443928735	0.414803508
1.112650121	0.430206134	0.198113624	0.531447837	0.536481378	0.358190753
0.870490223	0.367419474	0.460604369	0.527155319	0.251407571	0.493526686
0.657425809	0.456852388	0.387911647	0.436574569	0.418297129	0.368082228
0.767053973	0.351184238	0.247414164	0.683304248	0.263851849	0.317361714
5.325806453	0.454168641	0.249066	0.535849588	0.51054145	0.335154916
0.662273942	0.362888942	0.229473585	0.446026088	0.544725005	0.3304033
0.696936926	0.34231585	0.192002899	0.56120509	0.346974129	0.319790725
1.705033399	0.29565775	0.277450056	0.274485149	0.406041755	0.422874069
0.543555781	0.376912096	0.284204243	0.601109614	0.445593471	0.46771448
3.73497866	0.349944976	0.231245949	0.435305454	0.282006437	0.317581769
0.782954296	0.318309032	0.219333791	0.306785032	0.339433297	0.227935889
0.75675594	0.456377635	0.162915977	0.222673225	0.492842986	0.280077889
0.824276346	0.344434127	0.162194859	0.526717026	0.409007726	0.337486103
2.336420584	0.711482413	0.305236628	0.704123306	0.458311376	0.470250049
0.829089641	0.416185902	0.49127407	0.498235595	0.276317722	0.401869781
1.261989581	0.668731972	0.212494882	0.432058928	0.455114041	0.528838824
7.603164473	0.652748888	0.162060006	0.465418351	0.236924615	0.362486708
0.492501492	0.324682155	0.208107375	0.412223884	0.162814376	0.274180904
4.301381222	0.445284716	0.248204297	0.5538242	0.288751431	0.425785876
0.845689517	0.333648287	0.256565662	0.332102388	0.278818849	0.36180895
0.536853368	0.285151396	0.167380109	0.260039015	0.370180293	0.247654373
0.643985451	0.388073009	0.343742466	0.692986907	0.379638971	0.368260866
1.048625839	0.271627226	0.305511798	0.487103414	0.254228909	0.427086441
1.357356797	0.423079298	0.21997325	0.534328917	0.43149029	0.461627156
1.299358921	0.489201252	0.093434554	0.333856492	0.394883776	0.495308746
1.019173732	0.476517136	0.170294058	0.638783964	0.414717261	0.351890873
1.033974066	0.602903914	0.181998694	0.436483796	0.413741047	0.405001737
0.932063402	0.401758374	0.199560764	0.359758331	0.383979182	0.316022679
1.199472432	0.299701872	0.379981214	0.358389432	0.871335362	0.397437525
0.600693101	0.317339717	0.270724996	0.5030243	0.402455172	0.307828775
1.381082976	0.384805147	0.226518376	0.558837214	0.4695335	0.311348018
0.562646229	0.394309398	0.249670972	0.5163782	0.561944673	0.435909334

Q13576,IQGAP2,ONCOLOGY	Q15303,ERBB4,ONCOLOGY	Q06787,FMR1,ONCOLOGY	P98073,TMPRSS15,ONCOLOGY	Q01543,FLI1,ONCOLOGY	P48643,CCT5,ONCOLOGY
Q13576	Q15303	Q06787	P98073	Q01543	P48643
IQGAP2	ERBB4	FMR1	TMPRSS15	FLI1	CCT5
ONCOLOGY	ONCOLOGY	ONCOLOGY	ONCOLOGY	ONCOLOGY	ONCOLOGY
0.562919293	0.369846877	0.942457848	0.611659221	1.072888387	0.906261938
0.570777354	0.337532891	1.010801703	0.248238708	1.28422679	0.94809268
0.969693017	0.463037197	1.05277709	0.358787119	1.235504273	1.032971178
0.789329733	0.388692183	0.649049313	0.509940207	1.199056799	1.113498797
0.905069195	0.463518877	0.846569255	0.903001319	1.429291322	0.868140228
0.695923196	0.289874433	0.952373907	1.469372317	1.244615398	0.902813565
0.835435295	0.360032737	1.05709131	0.386194628	1.060173217	0.988491181
0.767107143	0.307935478	0.977453516	0.182415471	1.269622851	0.909660428
0.596378437	0.284835328	1.038283197	0.10984187	1.17723309	0.961594134
0.614165765	0.317031918	0.788290886	0.456472546	1.20280269	1.059658943
0.574588092	0.333648287	1.174380567	0.375295787	1.143613852	1.041165924
0.492433221	0.403153178	0.911869957	0.466710555	0.920442816	1.106574052
0.277854209	0.354879219	0.737032427	0.271740217	0.864956809	1.039795632
0.651934984	0.304687031	1.208736687	0.157072225	0.807592826	1.068287529
0.765142306	0.315759928	0.589800929	0.186661979	0.981799271	1.109108111
0.730319674	0.316987971	0.833006697	0.169810786	1.005630283	0.863638821
0.661723307	0.458533804	1.132648333	0.299701872	0.999099315	1.030325368
0.675018993	0.411139531	1.247119749	0.188403804	1.367271739	1.037635684
0.768171317	0.379612657	0.920634237	0.273327022	0.88172459	0.882947767
0.833122185	0.336015572	0.656924738	0.241182979	1.069472955	0.721014411
0.497752339	0.301870148	0.845220697	0.326329202	0.688916005	0.650986712
0.9151626	0.252700426	1.069621226	0.542990928	1.115661988	1.050735829
0.63366841	0.35858822	0.979895631	0.671984565	0.94789555	0.836942259
0.458692747	0.238225541	0.965869378	0.402510968	0.931611272	0.831045862
0.712469421	0.304982845	0.758699241	0.244566719	1.080500695	0.838335716
0.907267572	0.342933322	0.830297351	0.194211428	0.713161141	0.660348722
0.756808396	0.322394715	0.648329893	0.142427536	1.162394676	0.954621014
0.525040275	0.281440138	0.431191308	0.411595753	1.557357326	0.978402503
0.623862501	0.418616186	1.080500695	0.271702548	1.663820733	1.172103525
0.643360827	0.313122699	0.90293873	0.244363379	0.964665048	0.953166399
0.631694975	0.209380646	0.601568112	0.167972854	1.259717294	0.861247622
0.367801686	0.359334659	0.879222371	0.486934626	0.96781285	0.930707669
0.746130558	0.298851357	0.792289726	0.316900096	0.884908382	0.866036659
0.81140832	0.318551823	0.985276123	0.310227826	1.434850075	1.180419779
1.111185748	0.461755164	0.824390623	0.326193514	0.950527321	0.913324855

Q8WUX2,CHAC2,ONCOLOGY	Q99536,VAT1,ONCOLOGY	O00748,CES2,ONCOLOGY	P07947,YES1,ONCOLOGY	P39748,FEN1,ONCOLOGY	O14964,HGS,ONCOLOGY
Q8WUX2	Q99536	O00748	P07947	P39748	O14964
CHAC2	VAT1	CES2	YES1	FEN1	HGS
ONCOLOGY	ONCOLOGY	ONCOLOGY	ONCOLOGY	ONCOLOGY	ONCOLOGY
0.951384219	0.588943034	0.515198394	0.92678076	0.746596163	0.52453102
0.83479855	0.700812299	0.361057371	1.13981525	0.799627503	0.682925448
1.335518536	0.637633795	0.309862484	1.081624698	0.994745933	0.706029319
1.032255425	0.515412703	0.469631147	1.005072798	0.678067123	0.7031966
0.941935384	0.668037039	0.459042616	1.454879836	1.004445996	0.485418164
0.980439151	0.721814485	0.471130945	1.027686335	0.90162536	0.717425037
0.945992073	0.387320563	0.332010322	0.703684188	0.645415442	0.76833107
0.706861759	0.431729626	0.242944723	0.838219506	0.667805555	0.453633786
0.857613785	0.374620045	0.325200189	0.763288309	0.530270355	0.503722123
1.08192463	0.464644746	0.376729261	0.866336856	0.708136802	1.028327639
1.10297493	0.466322517	0.339033561	0.748254005	0.568093381	0.595841287
1.353973967	0.679337312	0.402343603	1.153805464	0.769130335	0.857078944
1.057018041	0.565186927	0.434732543	0.819547799	0.619381415	0.508316864
1.036844828	0.508951469	0.273592389	0.765036242	0.779488692	0.441841244
1.034332476	0.333671414	0.196513455	0.679902604	0.77781556	0.690397909
1.09847345	0.394446079	0.272948373	0.649139297	0.831795047	0.71434851
1.384821473	0.674925422	0.441045683	0.81490286	0.528069602	1.024911969
1.870468928	0.549579463	0.302414664	0.76774547	0.738515446	0.605080921
1.10175237	0.5069446	0.36698678	1.016915638	0.737799134	0.707008762
0.70329409	0.420389925	0.328121037	0.63454747	0.661494011	0.384165535
0.77938064	0.54559412	0.336995212	0.761069442	0.578504465	0.493766204
1.076463946	0.575943821	0.507788631	0.910859222	0.598822361	0.545821073
1.116512962	0.541262371	0.315322495	0.872120869	0.627679502	0.588902213
1.091415169	0.42670177	0.219090677	0.695440986	0.629684037	0.489065635
1.046012433	0.434431313	0.399619808	1.017268135	0.649274296	0.424106941
1.015647659	0.525295088	0.360432248	0.854528195	0.72865106	0.500346694
1.169101347	0.43080294	0.426938449	0.609796595	0.781707075	0.494382644
1.572652585	0.443559639	0.386971709	0.782628742	0.687341989	1.085605549
1.24616923	0.468720565	0.512775771	0.832083375	0.99075483	0.807368945
1.147902414	0.607855369	0.485653748	0.814394656	0.66328463	0.588575747
1.066955495	0.385232146	0.249636362	0.646714113	0.702806773	0.649004326
1.023704975	0.44220891	0.465902507	0.857673232	0.613570064	0.699356515
0.785182541	0.378561605	0.422376068	0.705197859	0.696888619	0.496099016
1.049498424	0.43149029	0.531521517	0.877700114	0.704513863	0.572243073
1.388088931	0.448941696	0.434973676	1.150451369	0.592053724	1.041887855

P34949,MPI,ONCOLOGY	Q05516,ZBTB16,ONCOLOGY	P37173,TGFBR2,ONCOLOGY	O75144,ICOSLG,ONCOLOGY	Q9UP79,ADAMTS8,ONCOLOGY	P09758,TACSTD2,ONCOLOGY
P34949	Q05516	P37173	O75144	Q9UP79	P09758
MPI	ZBTB16	TGFBR2	ICOSLG	ADAMTS8	TACSTD2
ONCOLOGY	ONCOLOGY	ONCOLOGY	ONCOLOGY	ONCOLOGY	ONCOLOGY
1.405905866	0.707695181	0.397244734	0.65094159	1.008212677	0.317603783
1.32408891	1.009751298	0.596998826	0.872544127	1.022782939	0.379823217
0.984184022	0.930062775	0.441443284	0.753458523	1.263477524	0.553939376
1.139420288	0.924983798	0.376624824	0.559379776	0.79454454	0.530895567
0.881602365	0.833353207	0.420623103	0.849802799	0.794764865	0.511107972
1.153405654	1.142663016	0.44723345	0.757595675	0.835609037	0.331274716
0.748617148	0.726432171	0.41359768	0.558333877	1.00751408	0.310163323
0.825191005	0.739283695	0.473685035	0.525513598	0.804296864	0.325786787
0.636838738	0.897696702	0.317471722	0.449221848	0.670356296	0.34837186
0.993230177	1.002706931	0.310378386	0.531263683	0.449377563	0.275399911
0.918594468	0.802181166	0.393735855	0.564521331	0.759962428	0.27524724
1.989353969	0.795812243	0.687484933	0.884540435	2.1022001	0.54646462
0.79636405	0.75236258	0.35566724	0.84797874	0.776092221	0.367852678
0.688772764	0.834856416	0.332563099	0.522969966	0.343718641	0.287154684
0.639359826	0.78008325	0.288611362	0.469598596	0.460604369	0.300180049
0.782411782	0.808152801	0.255235339	0.397823388	0.299972052	0.332586151
1.004445996	0.836072525	0.414976056	0.749759603	0.87575547	0.485653748
0.934133079	0.953100333	0.359608743	0.72733908	0.914148215	0.524094909
0.93342111	0.800126493	0.394774306	0.614719434	0.707106781	0.421966391
0.656151107	0.590087172	0.362939252	0.635163538	1.012203943	0.365463695
0.754712984	0.66646453	0.428955531	0.543103852	0.851512728	0.330724081
1.060246705	0.818582653	0.388476706	0.50557604	1.178131028	0.295289098
0.796971478	0.736164456	0.355642588	0.446737727	0.865556559	0.387535398
0.61221063	0.800237421	0.35343088	0.480730419	0.539651523	0.351964054
0.959596633	0.770143931	0.467325608	0.698339266	1.331267048	0.309948408
0.88423393	0.84844909	0.435999988	0.631694975	0.553440451	0.391477148
0.759962428	0.930772183	0.494759735	0.600360099	0.438302861	0.336948498
0.804798767	0.920442816	0.374230747	0.640557506	0.712568197	0.352770053
1.109338768	0.798243054	0.420739741	0.544234379	0.717673721	0.488557408
0.949934536	0.960395135	0.470673978	0.579146403	0.529058808	0.250156007
0.786871521	0.952968214	0.347816915	0.705931449	0.382332567	0.390853537
1.069324705	0.944157861	0.388288262	0.618952241	0.654107653	0.547867907
0.883008971	0.675393406	0.328826848	0.48350409	0.919805035	0.338728199
0.870309229	0.77781556	0.35055191	0.621058038	0.71946679	0.484208396
0.847214978	1.135320794	0.369104185	0.56750303	0.878065215	0.341770552

Q02742,GCNT1,ONCOLOGY	O60243,HS6ST1,ONCOLOGY	Q96DU3,SLAMF6,ONCOLOGY	P58499,FAM3B,ONCOLOGY	Q9P0G3,KLK14,ONCOLOGY	P43628,KIR2DL3,ONCOLOGY
Q02742	O60243	Q96DU3	P58499	Q9P0G3	P43628
GCNT1	HS6ST1	SLAMF6	FAM3B	KLK14	KIR2DL3
ONCOLOGY	ONCOLOGY	ONCOLOGY	ONCOLOGY	ONCOLOGY	ONCOLOGY
0.292863438	0.276356031	0.868561554	0.76418826	1.249022962	0.391314371
0.621661009	0.301284843	0.924727373	0.742107452	0.80865711	0.583579051
0.637147809	0.276068847	1.049862215	0.77362161	0.902750988	0.440373634
0.608951823	0.321546658	0.927744855	0.506312497	0.731992108	0.288611362
0.561010625	0.341628443	1.17299755	0.74421945	0.66328463	0.533145051
0.691211919	0.241986757	0.644208678	1.156127091	0.657015814	0.528106206
0.506031816	0.225844236	0.645057647	0.420856411	0.819831882	0.432388483
0.587841859	0.210093	0.845162113	0.36846513	0.963261894	0.328212024
0.414142739	0.16868458	0.737492355	0.262429171	0.94128271	0.20679884
0.582326428	0.144315684	0.968551052	0.493903124	0.536704541	0.25774208
0.32302103	0.188051537	0.41339705	0.404440674	0.681081807	0.528325885
0.600152066	0.310615128	1.296120633	0.808096786	1.520346327	1.064739129
0.470608734	0.179629973	0.462395736	0.344935854	0.816938851	0.18308684
0.463101392	0.194440412	0.365489027	0.261521235	0.764029368	0.189477704
0.269825761	0.115727209	0.919741281	0.211056329	0.458533804	0.379086767
0.465611953	0.168194216	0.983570248	0.132843542	0.62481457	0.273687225
0.543970379	0.241651524	0.917576279	0.360682167	1.165783591	0.451093999
0.430206134	0.252893174	0.746699671	0.464870248	0.596833326	0.312775627
0.439428394	0.24961906	0.664481068	0.558295178	0.989656656	0.259570799
0.399592109	0.22937817	0.486125258	0.297034003	0.646758942	0.985890962
0.450625231	0.155630853	0.664527128	0.271044192	0.482700424	0.231984448
0.430593964	0.217592389	0.620799801	0.479200062	1.042610287	0.34820287
0.369078601	0.199796055	0.812815602	0.552329083	0.943503647	0.28210419
0.203584552	0.159507924	0.856900738	0.337790346	0.769290288	0.245432805
0.435728082	0.156333623	0.848155091	0.685819105	0.573990992	0.443651884
0.548589913	0.205769349	0.41014331	0.252000762	0.791356681	0.403712455
0.292133558	0.206097656	0.77313915	0.308084926	0.83098826	0.53968893
0.348058087	0.203866976	0.405423045	0.214552111	0.660760797	0.316504957
0.717226153	0.270987836	0.872120869	0.329397155	0.977927894	0.651438098
0.409518349	0.18046613	0.563583	0.334853047	0.731028724	0.322148995
0.440099	0.219729427	0.710201346	0.402371492	1.017620755	0.363090226
0.299514966	0.239218356	0.924663278	0.458025555	1.077658445	0.663376587
0.430265777	0.171895061	0.606550638	0.426613049	1.063706401	0.229044528
0.340139863	0.184462569	0.752154009	0.292234822	0.655832817	0.308148997
0.566363423	0.316746372	0.997576925	0.544423029	0.930707669	0.324637148

Q9UQB8,BAIAP2,ONCOLOGY	Q02246,CNTN2,ONCOLOGY	P27695,APEX1,ONCOLOGY	P35637,FUS,ONCOLOGY	O00292,LEFTY2,ONCOLOGY	Q13490,BIRC2,ONCOLOGY
Q9UQB8	Q02246	P27695	P35637	O00292	Q13490
BAIAP2	CNTN2	APEX1	FUS	LEFTY2	BIRC2
ONCOLOGY	ONCOLOGY	ONCOLOGY	ONCOLOGY	ONCOLOGY	ONCOLOGY
0.7275912	0.771693575	0.310550544	0.7850737	0.159984052	0.735501404
0.542163539	0.593327271	0.292295597	0.717623978	0.401897637	0.496443005
0.888904293	0.558643569	0.381221132	0.882458293	0.338329293	0.965936329
0.908715127	0.571806926	0.245296746	0.766522476	0.264126324	0.650535639
1.059291757	0.784747265	0.522933718	0.980575078	0.505505958	1.447135488
0.829722032	0.349654021	0.265668669	0.885890321	0.316395284	0.634855429
0.530785182	0.397878542	0.242490478	0.714199981	0.203471692	0.666556928
0.451406782	0.737390124	0.153328735	0.553939376	0.170140676	0.880808319
0.392727356	0.399425958	0.256868164	0.877882646	0.115382793	0.682594171
0.457835107	0.424342181	0.490151621	0.820855396	0.198553542	0.940891323
0.523332587	0.359085674	0.242641798	0.808040775	0.248617542	0.554977043
0.845748137	0.519645621	0.693852062	0.800625793	0.193378593	0.78583591
0.517560708	0.459615705	0.273573425	0.607434182	0.214968921	0.746544415
0.408724322	0.434401202	0.227620121	0.67562752	0.173787955	0.801680896
0.501249223	0.369975079	0.155329096	0.82857259	0.106129674	0.80770479
0.327870953	0.266295508	0.533034198	0.684869017	0.109348096	0.633888061
0.469338267	0.486091563	0.458597374	0.778570723	0.142793281	1.066511853
0.4555559	0.598946896	0.451125268	1.064148876	0.347479555	1.361785994
0.682878113	0.551105336	0.435275282	0.926716523	0.247431314	0.526534511
0.338728199	0.730319674	0.419225971	0.7031966	0.244973908	0.690541488
0.589106346	0.4151199	0.281518181	0.728297602	0.094706024	0.611574433
1.082524743	0.374204808	0.297301779	0.852871325	0.121169626	0.766947644
0.595057093	0.352037251	0.206426487	0.610050255	0.246387334	0.709512497
0.363014731	0.222349336	0.245347759	0.734635236	0.264401085	0.587149584
0.30369604	0.383952567	0.214433171	0.891434066	0.24363613	0.63745703
0.2516691	0.649814571	0.259732779	0.478668907	0.166350718	0.476054945
0.41529258	0.342244675	0.352990191	0.677973129	0.085342016	0.656560563
0.689632658	0.741696055	0.45328804	0.912755271	0.184449783	0.68183757
0.685438912	1.045794944	0.391368623	0.840022572	0.248496941	0.841654481
0.316066492	0.3739196	0.264786229	0.690063007	0.179841764	0.607013286
0.400479417	0.335015558	0.251024486	0.863160051	0.44614977	0.599071456
0.345294677	0.925817668	0.188678246	0.719267339	0.241601279	0.727288667
0.409291327	0.40528256	0.210515738	0.60996569	0.12648165	0.587801115
0.300846609	0.449595656	0.300867463	0.984866443	0.095490432	0.726432171
0.546691936	0.653654418	0.762970933	0.716927929	0.213025789	0.714942935

P46060,RANGAP1,ONCOLOGY	P16562,CRISP2,ONCOLOGY	Q99683,MAP3K5,ONCOLOGY	Q9Y570,PPME1,ONCOLOGY	Q96I15,SCLY,ONCOLOGY	Q6EIG7,CLEC6A,ONCOLOGY
P46060	P16562	Q99683	Q9Y570	Q96I15	Q6EIG7
RANGAP1	CRISP2	MAP3K5	PPME1	SCLY	CLEC6A
ONCOLOGY	ONCOLOGY	ONCOLOGY	ONCOLOGY	ONCOLOGY	ONCOLOGY
0.838626312	0.246319031	0.717275869	2.808889751	0.386810805	0.240465195
0.96339544	0.268854961	0.783008568	2.329144263	0.398817328	0.383420665
0.833122185	0.30462368	1.378691814	4.089714122	0.477972659	0.511639658
0.938156166	0.328280281	0.828630024	1.715703197	0.478967609	0.609543041
1.012624995	0.236612796	0.755864742	1.599033318	0.351232926	0.40089602
0.882213658	0.460859853	0.83613048	2.295327478	0.385927031	0.333625161
0.909660428	0.213646857	0.621747195	2.135686666	0.270256271	0.268129154
1.530284613	0.277950523	1.646497133	1.45195828	0.253929515	0.210093
1.00542119	0.395294558	0.841946227	1.977804737	0.391070333	0.37051401
1.188383105	0.277046491	1.257013375	2.920266432	0.252525328	0.8489197
0.967544553	0.306614961	1.252490794	1.605252227	0.316263726	0.297879346
0.925946023	0.437695665	0.926010206	5.569701459	0.534143765	0.37221291
1.075867193	0.383181549	1.470391162	1.668324581	0.281245126	0.320767526
1	0.315869381	1.069547088	2.470837274	0.261847729	0.328507907
0.810115772	0.202781789	0.760542092	2.103074562	0.338282394	0.314122677
0.839498702	0.26605566	0.62161792	3.207835141	0.332586151	0.315759928
0.839324152	0.420011286	1.319142116	9.251581459	0.339786397	0.374775877
1.23105908	0.152290727	0.876484206	1.877873654	0.349726737	0.365514362
0.946123225	0.300533975	0.561360711	1.962646028	0.351354675	0.347118461
2.116235231	0.278722234	2.5752277	2.004857921	0.469566047	0.432448429
0.834624977	0.201940194	0.914972317	7.698620738	0.326781904	0.179555282
1.103051385	0.229346373	1.404250192	3.679475173	0.380851373	0.478668907
0.922742493	0.323626129	0.575345312	1.958976376	0.355963199	0.326351822
0.815976778	0.297404834	1.297379007	1.263127262	0.272022897	0.172467927
0.88172459	0.283358424	1.295042999	1.652327884	0.332747562	0.396447024
0.765778999	0.327394048	1.243752995	2.778102828	0.295616766	0.273801072
0.899627707	0.248996954	1.093762875	2.747653346	0.25491709	0.496133404
0.821481505	0.299141505	0.848155091	7.629560748	0.3021004	0.281771968
1.380413033	0.398402885	1.069695369	4.555154539	0.268017666	0.365742453
1.039435329	0.26288432	0.976640831	1.968640937	0.230254299	0.263194273
0.617666504	0.391585704	1.240825293	2.208856965	0.211554312	0.320167772
1.060834794	0.342600699	0.603865853	2.354302987	0.500277336	0.497338492
0.897634481	0.10328481	0.714645661	1.849582945	0.21751699	0.263760421
1.031754693	0.227620121	0.984729921	1.578550046	0.233436166	0.419865746
0.825934912	0.354682487	0.886443141	7.820712234	0.423666217	0.290357056

Q8TE58,ADAMTS15,ONCOLOGY	Q9Y662,HS3ST3B1,ONCOLOGY	O75054,IGSF3,ONCOLOGY	Q9NPH0,ACP6,ONCOLOGY	Q9H156,SLITRK2,ONCOLOGY	Q11201,ST3GAL1,ONCOLOGY
Q8TE58	Q9Y662	O75054	Q9NPH0	Q9H156	Q11201
ADAMTS15	HS3ST3B1	IGSF3	ACP6	SLITRK2	ST3GAL1
ONCOLOGY	ONCOLOGY	ONCOLOGY	ONCOLOGY	ONCOLOGY	ONCOLOGY
0.808376899	0.35769454	1.047971876	0.457074107	0.394145443	0.195318431
1.393680633	0.66328463	1.076687814	0.35259893	0.604452133	0.235663462
1.127166	0.350211897	0.915416372	0.311412768	0.416792147	0.23981603
0.61327243	0.706323009	0.888781073	0.41847113	0.372496816	0.246301958
0.881602365	0.495858365	0.860770178	0.29498224	0.305448275	0.258010199
1.324456077	0.418500137	0.987327078	0.152692383	0.35150083	0.324277313
0.570105174	0.287812269	0.609500792	0.420069516	0.292619943	0.13210894
0.639581449	0.408695992	0.830297351	0.376938222	0.439580715	0.207747065
0.410513053	0.239450607	0.552673751	0.282887434	0.365033304	0.132815921
0.445562586	0.352208102	0.568014632	0.235663462	0.251198543	0.205983403
0.623430222	0.29711637	0.643851552	0.282084637	0.313231237	0.154888292
1.154605498	0.419138805	1.338669665	0.23444152	0.617538077	0.233274417
0.68141235	0.3672667	0.786217292	0.43149029	0.277430825	0.254369923
0.699114179	0.233938303	0.556980992	0.094942642	0.267460918	0.245790319
0.281615764	0.229680455	0.480064447	0.170778705	0.276432663	0.140749334
0.296560838	0.235059843	0.464226247	0.061673734	0.144535922	0.191790079
0.463615273	0.349072836	0.715042054	0.344434127	0.497028333	0.206712853
0.730370297	0.417891407	0.749292024	0.240298575	0.572679553	0.208960187
1.028541496	0.558643569	0.842179696	0.442454191	0.348130471	0.669149279
0.725777884	0.461531173	0.713457798	0.386649968	0.336038864	0.157562924
0.551678629	0.354313907	0.858327425	0.240949047	0.324209889	0.172085805
0.700569459	0.418239144	0.787253406	0.206326352	0.314013829	0.183506109
0.72206469	0.269657488	0.767958364	0.211774384	0.21082239	0.191312096
0.753667455	0.307019033	0.447047489	0.185874401	0.356382895	0.125078003
0.688486371	0.344696845	0.83294896	0.347118461	0.358439118	0.228030704
0.671146676	0.361107427	0.837929052	0.17824086	0.298664982	0.21540147
0.392074576	0.348879323	0.499030535	0.225734682	0.350503316	0.148044213
0.569828624	0.306020456	0.655469247	0.247534239	0.425402377	0.163255104
0.755445718	0.423813074	0.934651216	0.215192544	0.504665721	0.240982452
0.632308273	0.380007553	0.596130461	0.267164458	0.233727598	0.157945638
0.369360117	0.359932929	0.36366954	0.17476643	0.270125174	0.178203799
1.050808663	0.49768334	1.021507644	0.308448172	0.750175473	0.21099782
0.418297129	0.256245753	0.461979263	0.361733721	0.23301585	0.181281047
0.475725083	0.20656962	0.448599526	0.2032039	0.379086767	0.174621124
0.570342323	0.563622066	0.729763045	0.393217654	0.325628753	0.241668275

Q9NRA1,PDGFC,ONCOLOGY	Q99795,GPA33,ONCOLOGY	P15848,ARSB,ONCOLOGY	O43752,STX6,ONCOLOGY	O75569,PRKRA,ONCOLOGY	O14558,HSPB6,ONCOLOGY
Q9NRA1	Q99795	P15848	O43752	O75569	O14558
PDGFC	GPA33	ARSB	STX6	PRKRA	HSPB6
ONCOLOGY	ONCOLOGY	ONCOLOGY	ONCOLOGY	ONCOLOGY	ONCOLOGY
0.382279568	0.339174591	0.596378437	0.711285175	0.628724549	0.279302424
0.510399917	0.23768125	0.942131274	0.953959551	0.735909365	0.255199959
0.530932367	0.092589987	0.541224855	0.735909365	0.410257041	0.29508449
0.635559897	0.160428237	0.636529817	0.888965909	0.559379776	0.200920963
0.61890934	0.221533994	0.831852705	0.626419053	1.089752112	0.332033336
0.411909698	1.78967795	0.460253309	0.678443227	1.089827651	0.297899994
0.503059168	0.168591067	0.550723471	0.589760049	0.408951029	0.134231949
0.38646241	0.129210928	0.499376557	2.387333304	0.648914361	0.145238919
0.434491543	0.270593671	0.377408808	0.453130969	0.642202417	0.105411195
0.520763414	0.280972336	0.420885584	0.658657332	0.700035505	0.129686481
0.43603021	0.234084287	0.667111585	0.987327078	0.68491649	0.204688223
0.49768334	0.029413046	0.751945497	0.821253774	0.985685973	0.409234591
0.385419107	0.137128467	0.577222723	1.701255705	0.438454791	0.149778245
0.403432719	0.373997362	0.52268005	0.58305343	0.47384923	0.160294852
0.35566724	0.202276412	0.475131908	0.497304021	0.718669318	0.095861811
0.610896551	0.133489669	0.428212852	0.727238257	0.302288919	0.114157694
0.59857337	0.316197968	1.120932962	1.479284969	1.151408685	0.170908967
0.629858647	0.198539779	0.457708186	1.03907515	1.36301364	0.237500096
0.398513361	0.117155777	0.454861742	0.800625793	0.803795274	0.230413955
0.416907722	0.294022813	0.990823506	2.671592557	0.602694999	0.180904475
0.350697731	0.213276956	0.435546905	0.404356582	0.535812447	0.150663306
0.474375038	1.408930063	0.836420311	1.502536461	0.630601278	0.243298613
0.385472541	0.121043709	0.520150133	0.960927838	0.962928111	0.195602945
0.455492751	0.176690944	0.428628594	0.534699414	0.885337846	0.142783384
0.44220891	0.326736606	0.407847015	0.47404634	0.570381858	0.147624083
0.387723477	0.197318756	0.525222272	0.718022023	0.824905065	0.122181689
0.368950711	0.185938832	0.713804054	1.047318321	0.763288309	0.12909455
0.360982299	0.58735311	0.895087111	0.8527531	0.891866698	0.119567732
0.53894128	0.451563255	0.759067454	0.77819305	1.55584679	0.141522172
0.548970298	0.433859553	0.58548333	1.720109014	0.373117	0.162194859
0.547943862	0.476418057	0.609162906	0.971240172	1.034045738	0.178921666
0.382014684	0.241819082	0.559884055	0.642113395	0.901187995	0.266572525
0.374152936	0.075824085	0.539539317	0.657380242	0.471653736	0.118454097
0.416936621	0.583215109	0.459010799	0.586376829	0.426938449	0.117774572
0.484174834	0.254828757	0.669938236	0.737952571	1.371828373	0.086227986

P04637,TP53,ONCOLOGY	Q9GZV9,FGF23,ONCOLOGY	Q8IX05,CD302,ONCOLOGY	Q99075,HBEGF,ONCOLOGY	Q9Y5W5,WIF1,ONCOLOGY	O60911,CTSV,ONCOLOGY
P04637	Q9GZV9	Q8IX05	Q99075	Q9Y5W5	O60911
TP53	FGF23	CD302	HBEGF	WIF1	CTSV
ONCOLOGY	ONCOLOGY	ONCOLOGY	ONCOLOGY	ONCOLOGY	ONCOLOGY
0.326193514	0.419022611	0.287274133	0.2640165	1.030182544	0.591725511
0.562919293	0.448848351	0.382014684	0.214819967	1.188795039	0.608192529
0.333347774	0.385419107	0.349435963	0.240898948	1.635917506	0.539053362
0.454830214	0.369693094	0.346325375	0.252507825	1.069621226	0.427827167
0.368286393	0.891990345	0.461211375	0.257295833	0.875876884	0.414573556
0.250746245	0.577863239	0.440740079	0.238754528	1.14750465	0.539053362
0.488997841	0.564325716	0.400673778	0.199215251	0.695055458	0.440556818
0.453696678	0.43080294	0.298044571	0.202571062	0.909408252	0.403209071
0.70305039	0.244194058	0.217713081	0.211363769	0.695248195	0.463518877
0.317493729	0.426081111	0.27475164	0.306933921	0.874057442	0.540212901
0.557753667	0.429967643	0.318022336	0.264071406	0.995918783	0.534440039
0.386649968	0.504071398	0.480763742	0.298685685	2.086377187	0.90293873
0.450750188	0.349896466	0.33010571	0.235092432	0.856010264	0.633932
0.471719125	0.293127454	0.220767545	0.189359538	0.84077985	0.563543937
0.673523412	0.332309628	0.204135642	0.20324616	0.789439165	0.364628693
0.435094293	0.261158941	0.250086658	0.216254191	0.571331508	0.40598547
0.559728844	0.393708564	0.302561432	0.899066665	1.014240648	0.535107257
0.523622864	0.415782228	0.296869338	0.227037104	0.821538448	0.602653225
0.575784158	0.610727198	0.33008283	0.244177132	1.017056622	0.706959758
0.44881724	0.320145581	0.355223763	0.208193943	1.208736687	0.6012763
0.425874425	0.523768064	0.381829374	0.178117355	0.729105758	0.319259178
0.408865999	0.500763044	0.330861654	0.283358424	0.98173122	0.422756839
0.316812244	0.425549836	0.330517829	0.19922906	1.065182034	0.549769966
0.458279609	0.304286029	0.26228369	0.157464662	0.645549666	0.486260059
0.469208157	0.302477555	0.378063375	0.198704989	0.893103945	0.480697098
0.467746901	0.360832202	0.337603087	0.232257969	0.667250321	0.635163538
0.355174522	0.275801078	0.316987971	0.168567697	0.810789887	0.527813443
0.498546508	0.34068256	0.223291463	0.379244457	0.734889885	0.498546508
0.649139297	0.351817707	0.393899639	0.245143769	1.533895288	0.730927389
0.274523203	0.383979182	0.349557089	0.191166284	0.532443372	0.436362793
0.410712283	0.399342909	0.233177421	0.270237539	0.557908331	0.612040913
0.248927927	0.406943384	0.427293714	0.253279111	0.748565259	0.621402521
0.371723036	0.425313926	0.27675859	0.214492633	0.702465852	0.576423077
0.513914406	0.324772189	0.321412958	0.202024196	0.53211132	0.315519265
0.368925138	0.36478037	0.33743932	0.193070547	0.960594864	0.538455864

Q13421,MSLN,ONCOLOGY	P01298,PPY,ONCOLOGY	Q86WD7,SERPINA9,ONCOLOGY	P23515,OMG,ONCOLOGY	Q86SR1,GALNT10,ONCOLOGY	P51858,HDGF,ONCOLOGY
Q13421	P01298	Q86WD7	P23515	Q86SR1	P51858
MSLN	PPY	SERPINA9	OMG	GALNT10	HDGF
ONCOLOGY	ONCOLOGY	ONCOLOGY	ONCOLOGY	ONCOLOGY	ONCOLOGY
0.257759946	0.113369149	1.314396005	0.825877665	0.493526686	0.627157629
0.32687252	0.380402861	2.613168221	1.720705261	0.559573676	0.731839911
0.458057304	0.154085178	2.514201014	0.576223339	0.504246126	0.965936329
0.382995674	0.25685036	0.486765897	1.204220849	0.422317519	0.410342361
0.422581056	0.552443949	0.528582292	1.631840441	0.492603915	1.059732395
0.552175966	0.251494717	0.730724761	0.280330379	0.494005839	0.70110382
0.556595056	0.145672454	0.678020124	0.470902406	0.510187692	0.356852553
0.416099367	0.138484844	0.949934536	0.501006074	0.456187873	0.417138969
0.171146062	0.127308442	0.790205613	0.411139531	0.359359567	0.793223871
0.264767877	0.321903462	1.463984217	0.291163214	0.280913916	0.498028428
0.197182032	0.176091845	0.679572793	0.345821627	0.302058523	0.863758555
0.395788061	0.119991158	0.70514898	0.504665721	0.604284566	0.934586433
0.449564493	0.23906917	1.207982873	1.014451575	0.557174061	0.307892792
0.22540634	0.12244451	0.282338935	1.253880622	0.369590608	0.761280484
0.267053371	0.157377369	0.363392362	0.617195736	0.270143898	0.488659011
0.250312111	0.104024842	0.213336097	0.398044049	0.380772185	1.067917353
0.386971709	0.133739729	1.354255547	0.587393823	0.502466737	4.804212614
0.282299798	0.197373472	2.291988809	0.486698421	0.420215126	1.131549738
0.401535654	0.176311685	1.170236401	0.721964598	0.440831738	1.111031715
0.413311096	0.257688489	0.828515159	0.430504433	0.47109829	0.266350889
0.303864491	0.28753311	0.409575124	0.469696256	0.40762092	0.219714197
0.158867949	0.2769121	0.841654481	1.036916699	0.417341415	0.361007321
0.213025789	0.276796959	1.543708123	0.68179031	0.27302406	0.367394007
0.210486557	0.105469664	0.262556533	0.230078806	0.29257938	0.494691152
0.552673751	0.116419126	2.838246712	0.484275526	0.435848908	0.555092459
0.470445661	0.128273939	1.028042566	0.506066893	0.456567477	0.941804813
0.250572501	0.170684032	1.00486382	0.89564567	0.391450014	0.646848608
0.333532673	0.147654783	0.797026722	0.509940207	0.471849932	1.950305326
0.508246401	0.131196397	0.564599595	0.451125268	0.521160627	1.273677475
0.174318791	0.128701431	0.496649512	0.792124991	0.431370672	0.574070569
0.327167195	0.147113342	0.741284885	0.614932516	0.384778475	0.956342967
0.253771155	0.098974453	1.381082976	1.147107024	0.449221848	0.662733155
0.300804906	0.101194305	0.306827564	0.347768701	0.163560921	1.028185093
0.232870533	0.112820411	0.283378066	0.490729532	0.42806447	0.245620009
0.49338987	0.243382948	0.934716004	0.573116366	0.463454624	1.137605228

P13688,CEACAM1,ONCOLOGY	O43715,TRIAP1,ONCOLOGY	Q7L5N7,LPCAT2,ONCOLOGY	P28907,CD38,ONCOLOGY	P32004,L1CAM,ONCOLOGY	O00244,ATOX1,ONCOLOGY
P13688	O43715	Q7L5N7	P28907	P32004	O00244
CEACAM1	TRIAP1	LPCAT2	CD38	L1CAM	ATOX1
ONCOLOGY	ONCOLOGY	ONCOLOGY	ONCOLOGY	ONCOLOGY	ONCOLOGY
0.488151206	0.390853537	0.949144731	0.352818961	0.721014411	0.53096917
0.484712099	0.323424304	1.071847755	0.589760049	0.650986712	0.489642265
0.466387168	0.351890873	0.836652248	0.420215126	0.770517698	0.714893381
0.460317118	0.305681257	0.910732959	0.472996037	0.604703569	0.534662352
0.527630548	0.475560238	1.068287529	0.478469875	0.66646453	0.91503574
0.431699701	0.325583614	0.917258326	0.504700703	0.671891414	0.540475078
0.381617702	0.304834902	0.902750988	0.329146098	0.574946651	0.676189725
0.310141824	0.327371356	0.894156954	0.319037961	0.459615705	0.732195088
0.415321367	0.2640165	0.712914021	0.235614462	0.665910411	0.607855369
0.305469448	0.289111921	0.827941075	0.314079133	0.497545372	0.39801646
0.385419107	0.309883963	0.745561878	0.22104316	0.522390295	0.734889885
0.594933367	0.558333877	0.935688353	0.393381223	0.589024684	1.262952167
0.300450661	0.286478743	0.956409258	0.397878542	0.630076977	0.475560238
0.240565223	0.274352	0.797302999	0.244244842	0.443928735	0.442576882
0.2946757	0.21804533	0.782194881	0.176446168	0.605122863	0.520655135
0.330311705	0.318971626	0.823819397	0.192549329	0.384618483	0.538381223
0.509480912	0.291587342	1.085906584	0.206040521	0.818355726	0.702417162
0.415321367	0.422668939	1.077135689	0.347527729	0.654152994	0.528728867
0.451062733	0.317229755	0.957404173	0.366021424	0.722815826	0.738106039
0.392509642	0.312949115	0.849390572	0.405760406	0.661081478	0.625117805
0.268854961	0.308897479	0.737492355	0.215999518	0.626636191	0.52595089
0.380481971	0.416878825	0.965267025	0.231213894	0.447357466	1.048843917
0.416301309	0.295350508	0.769610293	0.278741555	0.638828242	0.762072415
0.333001366	0.302729254	0.77201458	0.238010974	0.523876989	0.512065405
0.372419366	0.317537746	0.991922974	0.365083912	0.580512887	0.386408839
0.441259731	0.310529019	0.866997657	0.339574493	0.558024357	0.471621044
0.445408193	0.314405857	0.871818667	0.314384065	0.597827016	0.499722818
0.443252293	0.343171108	0.863998073	0.274275944	0.507964648	0.574787264
0.429401757	0.356506429	1.060908328	0.493321476	0.558411284	0.55179336
0.345031504	0.283201341	0.90331433	0.203909373	0.559496108	0.520222246
0.393381223	0.285824205	0.944157861	0.386837618	0.482332524	0.477509056
0.415580538	0.303759198	1.046375016	0.285507391	0.559224704	0.508281631
0.271175736	0.354780839	0.815694031	0.168264181	0.502466737	0.415177452
0.313883261	0.291688416	0.929740496	0.224035615	0.450188154	0.49986139
0.380666627	0.315563008	0.909912675	0.32273009	0.51132058	0.467617232

Q15427,SF3B4,ONCOLOGY	Q96PD2,DCBLD2,ONCOLOGY	P21741,MDK,ONCOLOGY	P78552,IL13RA1,ONCOLOGY	P51692,STAT5B,ONCOLOGY	Q14203,DCTN1,ONCOLOGY
Q15427	Q96PD2	P21741	P78552	P51692	Q14203
SF3B4	DCBLD2	MDK	IL13RA1	STAT5B	DCTN1
ONCOLOGY	ONCOLOGY	ONCOLOGY	ONCOLOGY	ONCOLOGY	ONCOLOGY
0.726180452	0.38920442	0.485822092	0.39937059	1.223064086	1.104198847
0.776845711	0.330655316	0.500034659	0.431191308	0.860531555	0.916178111
0.5829322	0.30107608	0.669288439	0.447419488	1.358486285	0.963729385
0.848566718	0.26585288	0.414602293	0.493903124	1.060981867	0.880014988
0.722014642	0.36902744	0.542915659	0.461147442	1.365661562	1.036844828
0.910732959	0.300596476	0.451782408	0.406210659	0.904442067	0.990274229
0.837232372	0.246370257	0.517417229	0.508140725	1.040805146	0.815920221
0.621014991	0.310701261	0.537076685	0.507753435	0.660028396	0.600443332
0.651257506	0.268929514	0.356605288	0.395239763	1.147425114	0.817731998
0.74406471	0.20679884	0.445686139	0.493868891	0.773728864	1.102134274
0.565696441	0.320323156	0.40584479	0.384751805	0.867538687	0.718818777
0.951450166	0.507014882	0.822336061	0.438424401	1.826269908	0.99896082
0.730724761	0.225125284	0.420389925	0.328576225	0.798077082	0.799793799
0.543631139	0.188364631	0.382809888	0.303149217	0.824733549	0.96989468
0.40983071	0.198360958	0.324120011	0.284342174	0.648329893	0.836884249
0.605878325	0.197086382	0.35566724	0.333925923	1.547994129	0.886996305
0.789931796	0.235059843	0.621230256	0.472864912	2.094636641	1.845740842
0.749759603	0.302184172	0.474769776	0.388826917	1.005142466	0.718569696
0.60634046	0.282809012	0.463615273	0.438181354	0.972722369	0.972924662
0.706420933	0.319790725	0.543367431	0.355889186	0.55194637	0.766469346
0.491069798	0.262829661	0.452095668	0.371259539	0.610727198	0.665495124
0.541825426	0.271307343	0.468460723	0.527813443	0.998822344	0.924535101
0.362185326	0.255288419	0.483906425	0.532000682	0.828457733	0.695537401
0.524931108	0.181658402	0.334528261	0.316132223	0.519681642	0.629378587
0.558179096	0.242793213	0.491103837	0.463808125	0.784366595	0.816429377
0.485283596	0.28029152	0.58666141	0.383367515	1.246514789	0.945336589
0.65747138	0.296375891	0.450344204	0.393681275	0.955812805	0.844810693
0.867478556	0.378876615	0.541938107	0.412281034	2.362149049	2.496315011
0.825191005	0.316680513	0.652658404	0.394200087	1.336166691	1.224336392
0.564677871	0.25003466	0.511497821	0.346133384	0.802014375	0.643137893
1.100988958	0.221656873	0.42625835	0.487035892	0.652296594	0.956144122
0.622868708	0.328895232	0.525222272	0.376729261	0.753771943	1.066142293
0.576582918	0.183048772	0.473947775	0.322394715	0.741439047	0.768650676
0.650039819	0.23768125	0.4695335	0.353332901	0.538418542	0.857851599
0.649499356	0.312342328	0.469175635	0.418935487	2.146074263	0.853995279

Q9NP84,TNFRSF12A,ONCOLOGY	Q9UJ71,CD207,ONCOLOGY	P26010,ITGB7,ONCOLOGY	P41271,NBL1,ONCOLOGY	P50749,RASSF2,ONCOLOGY	P05231,IL6,ONCOLOGY
Q9NP84	Q9UJ71	P26010	P41271	P50749	P05231
TNFRSF12A	CD207	ITGB7	NBL1	RASSF2	IL6
ONCOLOGY	ONCOLOGY	ONCOLOGY	ONCOLOGY	ONCOLOGY	ONCOLOGY
0.20488695	0.423255289	0.706518871	0.319768559	0.501701098	0.537225615
0.323379471	0.52595089	0.54973186	0.454672609	0.534440039	0.40351662
0.25003466	0.43080294	1.446634036	0.365615718	0.825305409	0.614123196
0.235696134	0.547981844	0.667342828	0.405647921	0.778570723	0.523405141
0.269900583	0.481564183	1.121943481	0.330838721	0.792289726	1.222809783
0.279534838	0.528655574	0.781057139	0.411339066	0.901750361	0.618437625
0.263851849	0.47421066	0.534921835	0.434973676	0.295821743	0.374723926
0.370180293	0.523006217	0.756860856	0.387669731	0.392455233	0.245449818
0.18854751	0.205043228	0.530160099	0.299162241	0.570619122	0.273763118
0.1818978	0.385606159	0.481397315	0.304665913	0.533477748	0.885890321
0.209932873	0.3459415	0.507225788	0.331389547	0.648644541	0.436635096
0.318154625	0.446428179	0.708431369	0.654833484	0.715637057	1.203386436
0.232129213	0.465450612	0.47204621	0.377958568	0.791576123	0.355815188
0.17034128	0.479200062	0.377382649	0.278471193	0.448630621	0.311801549
0.14423568	0.213632049	0.463872427	0.227131546	0.467455196	0.383181549
0.219394612	0.401173996	0.513380356	0.225453217	0.67562752	0.254264156
0.266794346	0.573553512	0.512491506	0.399093863	0.372161314	0.420302517
0.28138162	0.318772703	1.173810892	0.372290317	0.878856789	0.891681259
0.253771155	0.443713391	0.446490072	0.315388071	0.923702386	1.424642565
0.171752142	0.505050656	0.636529817	0.40000779	0.621273318	0.547033086
0.18922833	0.203077175	0.47109829	0.381723524	0.415004821	0.845337877
0.220614573	0.408327885	0.42552034	0.318640157	0.73877144	1.181402033
0.255306115	0.31718578	0.401396516	0.321725011	0.488388116	0.429908041
0.270612428	0.264053103	0.369769978	0.278606341	0.598075697	0.291183397
0.213572826	0.326917837	0.51501987	0.360307353	0.73989887	0.337065296
0.239533609	0.401730527	0.557715008	0.291749078	0.378482893	0.440984545
0.154448739	0.34393313	0.462267551	0.384245428	0.56178889	0.327711908
0.158603883	0.314798375	0.394090807	0.366885044	0.50923377	0.435758285
0.23499468	0.533884659	0.628071191	0.407903559	0.484275526	0.469175635
0.230286222	0.411453129	0.763235404	0.303044171	0.497545372	0.59291615
0.27475164	0.542464262	0.514948478	0.375425877	0.54231388	0.204390493
0.240265265	0.563465818	0.773031978	0.369436931	0.644253333	0.412852973
0.149415322	0.406379632	0.631782552	0.29233612	0.433138404	0.298954949
0.187504874	0.4118526	0.806306356	0.270574915	0.543706507	0.359658598
0.161197358	0.359533972	0.522897472	0.495583479	0.889458994	0.37822064

Q9C005,DPY30,ONCOLOGY	Q9BYH1,SEZ6L,ONCOLOGY	Q9Y336,SIGLEC9,ONCOLOGY	Q6PCB0,VWA1,ONCOLOGY	Q9ULX7,CA14,ONCOLOGY	Q9NZ53,PODXL2,ONCOLOGY
Q9C005	Q9BYH1	Q9Y336	Q6PCB0	Q9ULX7	Q9NZ53
DPY30	SEZ6L	SIGLEC9	VWA1	CA14	PODXL2
ONCOLOGY	ONCOLOGY	ONCOLOGY	ONCOLOGY	ONCOLOGY	ONCOLOGY
0.715785885	0.955282936	0.684726617	0.308512318	0.365311734	0.934780795
0.80068129	0.685724037	0.488997841	0.395815496	0.350114811	0.831449185
0.621101088	0.789001528	0.688725023	0.375660153	0.474539472	1.030039741
0.521558144	0.858030002	0.377932371	0.350527612	0.298147883	0.608403349
0.890631165	0.947829849	0.868802404	0.311563903	0.358439118	0.757490657
0.625811467	0.610007971	0.41549413	0.259085468	0.406858772	0.554477183
0.45948829	0.571608787	0.479831575	0.264272828	0.283712181	0.81298464
0.573593269	0.711433098	0.362788342	0.200434116	0.27325125	0.810115772
0.384965216	0.691547378	0.652160967	0.243113178	0.266037219	0.740206649
0.523114984	0.43452166	0.486968379	0.279360509	0.210369871	0.51788368
0.525367915	0.562529242	0.419778447	0.290759856	0.254493374	0.611744021
1.03053964	1.083951345	0.726885484	0.40615435	0.414056629	1.236532364
0.385205445	0.723567744	0.341817935	0.276049712	0.301117821	0.795150581
0.441290318	0.522571373	0.31718578	0.213025789	0.178921666	0.539576716
0.34393313	0.497441922	0.333486439	0.116969151	0.105214103	0.473685035
0.425638336	0.374646012	0.477840155	0.154202707	0.271514283	0.582164995
0.534958914	0.962928111	0.521124504	0.283319145	0.339598031	0.845279285
0.554323471	0.929740496	0.601609811	0.354191133	0.27486593	0.753928701
0.667574152	0.72895416	0.817448643	0.477542156	0.252490323	0.543781886
0.447109467	0.46223551	0.631476084	0.285072346	0.289914621	0.571529551
0.649724494	0.675533865	0.242053859	0.262957217	0.203866976	1.053945305
0.774265358	0.44537732	0.529058808	0.408894341	0.285863831	0.547905883
0.61839476	0.686342216	0.568172141	0.225421965	0.346805816	0.683020128
0.404272507	0.44537732	0.372832621	0.200017759	0.212907695	0.540887327
0.572441432	0.80860106	0.343432862	0.236612796	0.329465658	0.756283999
0.495240086	0.608994034	0.487880592	0.269788358	0.374697953	0.830354904
0.434130293	0.636529817	0.385713087	0.227289036	0.354461293	0.74262202
0.635339667	0.713655638	0.323603698	0.29982654	0.205199625	0.686865725
0.460572443	0.584469643	0.615657549	0.30036737	0.422756839	0.792894047
0.495034164	0.522969966	0.51359391	0.238589093	0.207344258	0.752884258
0.502118575	0.350843612	0.599237577	0.239002895	0.525841533	0.520655135
0.688200097	0.769930432	0.66342257	0.31930344	0.320989943	0.839556894
0.524276578	0.536258308	0.590660074	0.253982324	0.281986891	0.577462833
0.473192791	0.536481378	0.409149502	0.18640339	0.208699638	0.790315167
0.662273942	0.80647404	0.472701058	0.29189067	0.215745145	0.71182771

P10606,COX5B,ONCOLOGY	Q6FI81,CIAPIN1,ONCOLOGY	Q8IWL1,SFTPA2,ONCOLOGY	P06731,CEACAM5,ONCOLOGY	Q96NY8,NECTIN4,ONCOLOGY	Q6PGN9,PSRC1,ONCOLOGY
P10606	Q6FI81	Q8IWL1	P06731	Q96NY8	Q6PGN9
COX5B	CIAPIN1	SFTPA2	CEACAM5	NECTIN4	PSRC1
ONCOLOGY	ONCOLOGY	ONCOLOGY	ONCOLOGY	ONCOLOGY	ONCOLOGY
0.538791875	0.618266182	0.62676651	0.332102388	0.451375494	1.06355895
0.669056522	0.666325957	0.544347562	0.207301146	0.444421341	0.780299565
0.577863239	0.748980467	0.510753822	0.22147258	0.502222998	0.863160051
0.596709231	0.461787171	1.084327079	0.310163323	0.355494711	0.958599438
0.776576524	0.653609111	0.458406689	0.336341802	0.371851887	0.766416221
0.665587387	0.616084438	0.611150668	0.306211421	0.525586455	0.94586094
0.531890067	0.562373298	0.482499717	0.568369089	0.500034659	0.617067407
0.416099367	0.574468622	0.476054945	0.233452347	0.594068007	0.849861705
0.438728399	0.481564183	0.553862589	0.403432719	0.350503316	0.702660644
0.454420555	0.591602478	0.606088343	0.28813164	0.318331096	0.837464534
0.425726854	0.52635206	0.498926775	0.201143915	0.393708564	0.684204738
0.517094548	1.364809884	0.614889894	0.305194316	0.745458529	0.774909642
0.51975369	0.467001795	0.613527536	0.261285687	0.426406105	0.872423175
0.607055363	0.554438751	0.513487122	0.339339199	0.397052036	0.690685097
1.667053032	0.421119037	0.295596276	0.154502276	0.267182977	0.711334479
0.453476596	0.640468712	0.35379854	0.402064816	0.282769809	1.028684092
0.378299297	1.429291322	0.998130253	0.324884766	0.514056913	1.021153678
0.617324092	0.561321802	0.483169067	0.463133493	0.385659619	1.148459515
0.653382627	0.597951344	0.77271055	0.248634776	0.389339332	0.64015803
0.473356816	0.440831738	0.475988954	0.21715544	0.371362488	0.713853533
0.321814224	0.457644739	0.610896551	0.162161135	0.42889607	0.7944344
0.642246933	0.896515232	0.490423494	0.257617053	0.378692827	1.012274106
0.527411159	0.609543041	0.388126811	0.234522785	0.421440247	0.83098826
0.516628809	0.401396516	0.43875881	0.367648754	0.408412804	0.866697231
0.735093668	0.447884921	0.551105336	0.223198618	0.412938832	0.73163703
0.660944025	0.701298234	0.27524724	0.655651007	0.383394089	0.546957257
0.370565378	0.572005134	0.435305454	0.195792852	0.451531956	0.626288807
0.614762044	1.24971576	0.451531956	0.262775012	0.350746351	0.81292829
0.519501565	0.830757893	0.693707795	0.290457704	0.50754231	1.088242442
0.533182007	0.453979797	0.507014882	0.132063162	0.434491543	0.762072415
0.56003931	0.403041416	0.793993994	0.364653968	0.45394833	0.832025702
0.744941995	0.49909972	0.449066186	0.324299791	0.438789224	0.957802429
0.503268428	0.590496331	0.43593955	0.285883646	0.505085664	0.795315945
0.681317893	0.440129507	0.451907686	0.435607289	0.401396516	0.912881814
0.4799979	1.243925428	0.915606748	0.3459415	0.416214751	0.765991347

Q96RT1,ERBIN,ONCOLOGY	Q92832,NELL1,ONCOLOGY	P07237,P4HB,ONCOLOGY	Q5VIR6,VPS53,ONCOLOGY	P55039,DRG2,ONCOLOGY	P01303,NPY,ONCOLOGY
Q96RT1	Q92832	P07237	Q5VIR6	P55039	P01303
ERBIN	NELL1	P4HB	VPS53	DRG2	NPY
ONCOLOGY	ONCOLOGY	ONCOLOGY	ONCOLOGY	ONCOLOGY	ONCOLOGY
0.933291719	0.288051764	0.309883963	1.099768599	1.144882864	0.240915647
1.526365005	0.165339118	0.330678236	1.09179349	0.836826243	0.454326071
0.834856416	0.4575813	0.375061738	1.093459662	0.935688353	0.374983755
1.032398535	0.485115439	0.302603378	0.995159722	0.826908727	0.356160641
1.565908593	0.250694109	0.363972158	1.096875665	1.012484625	0.769716991
1.156207231	0.258923892	0.292214566	1.099616149	0.999792077	0.529939658
1.12747856	0.170766868	0.314405857	0.907707888	1.107034359	0.214864642
1.170885498	0.203824587	0.327008491	0.975017488	0.959796196	0.19356634
1.354631078	0.241534303	0.297693577	0.908463213	0.995090745	0.144365708
0.868922854	0.196254821	0.290437571	1.167805491	1.188877442	0.259696774
1.082975046	0.294430697	0.222164468	0.879344266	1.222640278	0.296006344
1.63104886	0.233193584	0.415551734	1.032827987	0.94881584	0.208786451
1.022924736	0.293025881	0.270031572	1.105960607	1.044997869	0.244770229
0.985481026	0.245773283	0.302205118	0.965735489	0.852339442	0.176201731
0.717425037	0.207876705	0.178822478	0.868380961	0.80994733	0.154117223
0.856306986	0.264474402	0.179219559	1.020658331	1.050517358	0.149280745
1.797884074	0.355100673	0.320567484	1.214615765	1.239106336	0.182326984
0.881174713	0.443252293	0.256796955	0.929676053	1.006536856	0.43419048
1.203553272	0.266387815	0.315716157	0.944485138	0.953695093	0.43669563
1.448138913	0.162138657	0.216104347	1.024769896	1.117674427	0.369283319
0.74102802	0.3455341	0.272815969	1.038211231	0.779542724	0.171252862
1.262077059	0.181973465	0.38844978	1.107878753	0.985002984	0.172599477
1.019385686	0.252525328	0.261938495	0.910796088	0.875937598	0.263815274
1.044563357	0.150465016	0.206641224	0.939653004	0.939262294	0.241350212
1.300620438	0.238126486	0.26186588	1.059585495	1.356322259	0.174210079
1.175520746	0.230765587	0.342173515	1.195074038	0.888965909	0.197086382
1.168210292	0.134390215	0.239849278	1.117209696	0.788236248	0.149218674
1.320422841	0.188286308	0.30665747	1.004445996	0.745045273	0.187999405
1.061717539	0.260941805	0.313644028	1.015366101	1.041382451	0.247311289
1.593832477	0.338681244	0.266480154	1.161428225	0.875634073	0.242423254
0.913388164	0.202332503	0.191126536	0.921208737	1.101141598	0.43431088
1.461246936	0.267535084	0.299598021	0.993367877	0.740719899	0.2651168
1.721659687	0.203768083	0.303296341	0.866517025	0.944812528	0.142635006
1.011011915	0.2946757	0.242255277	0.893537387	0.696792017	0.173896403
1.588318236	0.51505557	0.360257407	1.070585487	0.969357005	0.246951562

Q12846,STX4,ONCOLOGY	O15357,INPPL1,ONCOLOGY	Q9H6B4,CLMP,ONCOLOGY	Q16595,FXN,ONCOLOGY	Q9NUY8,TBC1D23,ONCOLOGY	Q16775,HAGH,ONCOLOGY
Q12846	O15357	Q9H6B4	Q16595	Q9NUY8	Q16775
STX4	INPPL1	CLMP	FXN	TBC1D23	HAGH
ONCOLOGY	ONCOLOGY	ONCOLOGY	ONCOLOGY	ONCOLOGY	ONCOLOGY
0.688104699	0.969424198	0.490185597	0.801458653	1.217312859	7.371202539
0.701152418	1.100454884	0.539128096	0.871576981	1.160864833	5.283155963
0.602277388	1.087563772	0.38788476	0.866396908	0.96727633	4.681919081
0.648419777	3.275919714	0.389744346	0.826679491	1.072070662	2.448503062
0.992060493	1.102822036	0.33263226	0.693611633	1.026404926	2.291988809
0.692266769	0.975558302	0.374386417	0.842880493	1.239879572	3.300535236
0.505505958	1.119535286	0.314820196	0.794269219	0.925047915	3.813458501
0.557483109	0.832371804	0.385178745	0.628158266	0.780840614	1.963326347
0.566716849	0.722565361	0.279786838	0.782357551	0.858743989	2.737198383
0.664066672	4.015000034	0.215341756	0.832775771	1.116280814	5.66823668
0.604033303	0.700375246	0.29565775	0.962060815	1.167077203	2.756237103
0.853521855	1.090810128	0.685628982	0.906576079	1.175683719	12.53769296
0.512349433	0.746389192	0.246267815	0.627331539	1.198059866	3.435452176
0.476913656	0.85340354	0.227667458	0.658474739	1.281736764	4.46202934
0.405394944	0.74675143	0.166350718	0.698145672	1.083125188	2.536606968
0.57256048	0.718320702	0.1902278	0.749967509	1.122254592	5.266702616
0.642157904	1.818690437	0.342126083	1.174950518	1.199306161	8.5084716
0.507788631	1.095128377	0.375477926	0.759330572	0.96727633	2.752609584
0.681270669	1.088695123	0.398983226	0.834277938	1.040372377	2.255895126
0.632615145	0.614080629	0.27289162	0.752727717	0.810396585	2.763123412
0.60996569	0.695585614	0.255412316	0.946254394	0.85862495	1.311120242
0.696888619	0.984252243	0.324907286	1.047318321	1.108646942	6.476884423
0.513344773	0.812421317	0.297322387	0.790369949	0.958665886	2.613892845
0.338704721	0.558295178	0.229108041	0.752519045	0.804352616	1.511310402
0.647521499	0.976302412	0.324097546	0.874057442	1.017479692	2.702322765
0.556440757	0.692554734	0.277642437	0.664204775	1.057164585	5.024569525
0.448661719	1.126463057	0.414631032	0.858030002	1.249975658	5.00371632
0.615657549	1.331543906	0.326397067	1.168372251	1.030611074	15.32377667
0.580110646	1.015647659	0.392672916	1.118992216	1.243925428	5.873009616
0.652296594	1.190939396	0.468655591	0.798851916	1.094445413	2.002496887
0.508880918	1.118526937	0.245330754	0.798519752	1.255359003	3.029542616
0.568211525	0.701833151	0.370231615	0.774265358	1.349570179	2.799559806
0.518745924	0.637943252	0.266627963	0.851925985	0.894714933	3.292081381
0.477608362	1.090810128	0.238622171	0.806753591	1.204888796	1.832356222
0.732448891	1.113344444	0.279612352	1.064517746	1.538367298	10.14150599

Q9H4A9,DPEP2,ONCOLOGY	P10747,CD28,ONCOLOGY	P47929,LGALS7_LGALS7B,ONCOLOGY	P08473,MME,ONCOLOGY	Q7Z6M1,RABEPK,ONCOLOGY	P17948,FLT1,ONCOLOGY
Q9H4A9	P10747	P47929	P08473	Q7Z6M1	P17948
DPEP2	CD28	LGALS7_LGALS7B	MME	RABEPK	FLT1
ONCOLOGY	ONCOLOGY	ONCOLOGY	ONCOLOGY	ONCOLOGY	ONCOLOGY
0.999168569	0.440343111	0.463808125	0.376363858	0.986095993	0.672963424
0.984593418	0.502710595	0.322215991	0.459010799	1.022215945	0.820457211
0.816712379	0.912059595	0.412138173	1.215710738	0.790698723	0.720864496
0.822735158	0.680704239	0.40666141	0.289894527	0.617152957	0.600152066
0.920825697	0.489947815	0.352061653	0.339268643	1.077285022	0.807312984
0.790808345	0.355420795	0.466193243	0.510860041	1.014100054	0.815694031
0.714992493	0.576463033	0.431759552	0.354953022	1.80325072	0.648689504
0.690158677	0.586417475	0.335015558	0.445624359	0.597040208	0.489201252
0.505190705	0.485149066	0.225000482	0.174306708	0.863938187	0.433408694
0.625291149	0.479565573	0.397961287	0.221411184	1.065698989	0.567463695
0.498028428	0.350819294	0.445315582	0.234360283	0.870852325	0.54916059
0.972992102	0.61021942	0.536741744	0.449564493	0.949868694	0.812139802
0.659753955	0.442484861	0.332378738	0.514520334	1.09179349	0.643048742
0.363568723	0.429788862	0.377749042	0.208352743	1.051974694	0.560311108
0.660348722	0.410029609	0.314471243	0.645281245	1.171941048	0.42720487
0.571886201	0.382730293	0.299514966	0.204079051	1.029825572	0.56899978
0.927873476	0.608066072	0.489676206	0.270106451	0.968752478	0.586417475
0.877395979	0.59423274	0.405788532	0.374983755	1.746056205	0.690828736
0.897447842	0.437271128	0.467358002	0.954819543	0.645997282	0.804910344
0.768917117	0.287174589	0.441382091	0.322841959	0.765248385	0.586133012
0.594068007	0.366300608	0.343171108	0.247191322	1.007374418	0.56007813
0.726835102	0.348251145	0.423284627	0.283005108	1.003750009	0.591725511
0.685486424	0.374853818	0.374957764	0.200378552	1.016633728	0.575584641
0.41529258	0.311823162	0.312342328	0.325358015	0.790150842	0.386569575
0.819150248	0.485384519	0.306997753	1.060908328	0.672264093	0.711679705
0.932709683	0.460508599	0.284993318	0.321012193	0.627592493	0.641890894
0.835609037	0.585321022	0.353112549	0.234327796	0.920315225	0.69380397
0.676752398	0.407084445	0.303906619	0.189543383	0.94809268	0.509410287
0.803962436	0.513558311	0.406915178	0.382226577	1.470798897	0.656287564
0.712963438	0.403740439	0.37221291	0.336528361	0.579869435	0.582851394
0.555823986	0.405366845	0.466096311	1.112187479	0.662090346	0.647072827
0.759909753	0.531742616	0.3842987	0.472963252	0.987669319	0.541938107
0.650265145	0.286578046	0.42117742	0.136502568	0.680798612	0.483470578
0.544083507	0.33690179	0.409319698	0.205983403	0.829319546	0.509480912
0.851925985	0.350357576	0.292275337	0.340281353	0.862143545	0.643360827

Q9UQQ2,SH2B3,ONCOLOGY	Q7Z5A7,TAFA5,ONCOLOGY	P16870,CPE,ONCOLOGY	P40121,CAPG,ONCOLOGY	Q92876,KLK6,ONCOLOGY	Q6UWN8,SPINK6,ONCOLOGY
Q9UQQ2	Q7Z5A7	P16870	P40121	Q92876	Q6UWN8
SH2B3	TAFA5	CPE	CAPG	KLK6	SPINK6
ONCOLOGY	ONCOLOGY	ONCOLOGY	ONCOLOGY	ONCOLOGY	ONCOLOGY
0.73189064	0.529902926	0.538381223	0.304982845	0.352794506	0.672124314
0.99529769	0.675768028	0.527959804	0.326578111	0.513736328	0.217818742
0.911238116	0.461243345	0.594892131	0.280058477	0.312407285	0.519141599
0.888473099	0.52445831	0.553402091	0.638474099	0.575225684	0.199533101
1.425037614	0.651528413	0.491887398	0.313948538	0.747942879	0.292680797
0.933550518	0.615188313	0.503477775	0.441167983	0.386060806	0.362486708
0.676705491	0.374490234	0.369693094	0.1859775	0.416821038	0.659068352
1.184271612	0.477376681	0.322104339	0.270856384	0.497235084	0.293982055
1.282358818	0.367674238	0.372238711	0.198994436	0.303927685	0.277661682
1.129668909	0.323334644	0.261648157	0.109416332	0.316987971	0.133323223
0.836072525	0.286339777	0.423519411	0.201926197	0.325719049	0.494759735
0.891063406	0.886688949	0.62494451	0.596171783	0.485384519	1.924255005
1.467438458	0.466775259	0.527557408	0.210647106	0.363871257	0.184462569
0.643137893	0.342244675	0.382597672	0.19694982	0.472144379	0.336155346
1.021720083	0.287075079	0.225296999	0.064960205	0.276643513	0.443559639
1.114966219	0.404608911	0.228901687	0.23715464	0.469793937	0.27524724
1.10175237	0.385419107	0.458724542	0.300513144	0.550113038	0.593862154
2.237519104	0.370231615	0.503373091	0.351890873	0.378929142	0.338563887
0.8272527	0.400229663	0.375503953	0.343694817	0.410228605	0.380877772
0.989862471	0.417081145	0.30317023	0.185321221	0.364174043	0.253033446
0.840721574	0.393899639	0.383819523	0.361307723	0.295268631	0.155027923
1.306855835	0.368286393	0.446397236	0.179904104	0.370462649	0.502849995
0.892856358	0.371980784	0.530417397	0.164630096	0.442975864	0.208989157
0.960528283	0.342980866	0.291627768	0.264126324	0.366300608	0.118191648
1.32408891	0.598490395	0.152100837	0.257581342	0.344028502	0.142951732
0.637147809	0.385392393	0.353504381	0.195792852	0.40559169	0.283987631
1.291278329	0.476649273	0.312537238	0.198953061	0.399453645	0.093954105
1.182876947	0.458311376	0.131360188	0.327530236	0.274409056	0.260959893
0.566756132	0.551602156	0.38371312	0.105887192	0.303738144	0.254811095
0.635692071	0.48524996	0.320545264	0.434943527	0.464741376	0.288231517
0.916368645	0.333625161	0.388665242	0.194818151	0.362059823	0.2639982
0.709364973	0.653473211	0.462363687	0.235239136	0.388288262	0.1393901
0.90682747	0.376781491	0.290800166	0.141091208	0.291122853	0.245654062
0.835666959	0.493013822	0.344864134	0.214656237	0.398817328	0.201800269
1.069176475	0.457264239	0.373401596	0.205014805	0.433258512	0.169199827

Q9UBX7,KLK11,ONCOLOGY	P25786,PSMA1,ONCOLOGY	P0CG37,CFC1,ONCOLOGY	O14662,STX16,ONCOLOGY	Q8TDQ1,CD300LF,ONCOLOGY	O00584,RNASET2,CARDIOMETABOLIC
Q9UBX7	P25786	P0CG37	O14662	Q8TDQ1	O00584
KLK11	PSMA1	CFC1	STX16	CD300LF	RNASET2
ONCOLOGY	ONCOLOGY	ONCOLOGY	ONCOLOGY	ONCOLOGY	CARDIOMETABOLIC
0.432208695	1.035480224	0.480197567	0.56848729	0.308213081	0.420710579
0.387938536	0.942327205	0.798409062	0.579708683	0.488083538	0.45040664
0.436604831	1.747993718	0.645639164	0.555939579	0.342363309	0.4280348
0.351086883	1.114579868	0.560544184	0.853226098	0.270875159	0.447388476
0.417283563	1.218748126	0.596461118	0.463037197	0.478072061	0.350430439
0.431699701	1.660940048	0.43548653	0.462973011	0.364249778	0.433829481
0.448288688	1.070066161	0.703586643	0.481464055	0.353137026	0.35343088
0.489099536	0.821766258	0.796308852	0.562295342	0.447543556	0.324794701
0.325042439	1.213521778	0.441902501	0.484644908	0.361583312	0.257349342
0.312125904	1.082374683	0.481630947	0.44590244	0.081684151	0.24691733
0.362536963	1.359334017	0.670728123	0.644521326	0.360757177	0.365565037
0.684536797	1.055773237	0.80446413	0.378850354	0.51054145	0.53562678
0.400729327	1.45276364	0.788564134	0.655741906	0.201032408	0.353357393
0.378535366	1.023917871	0.303464571	0.386435623	0.239932418	0.333463324
0.259984947	1.548423384	0.459329071	0.961460838	0.117701124	0.243264887
0.341533737	1.652213357	0.335852576	0.565186927	0.344601288	0.339433297
0.533440771	1.002706931	0.607981782	0.622696036	0.390609786	0.360132573
0.391314371	1.377450046	0.507964648	0.601776636	0.34455352	0.352208102
0.312645574	1.328685814	0.527996401	0.356926766	0.42217118	0.398182025
0.333972218	1.069250588	1.053215019	0.968081222	0.423313968	0.357868136
0.315475528	0.877882646	0.457391037	0.224906927	0.308448172	0.30149375
0.467001795	1.1076484	0.661494011	0.62772301	0.219394612	0.365008003
0.474868512	1.46571032	0.378719077	0.449128445	0.34837186	0.342553208
0.380719402	0.573951207	0.533588693	0.601568112	0.222997586	0.30120132
0.385900281	0.983842989	0.42699764	0.607476287	0.42513708	0.374308574
0.433198454	0.803628147	0.462363687	0.352452318	0.310701261	0.420215126
0.368005696	0.931675848	0.471392268	0.515091272	0.397189668	0.44070953
0.347118461	1.133355136	0.433829481	0.662319849	0.35055191	0.287174589
0.348782606	1.994324262	0.531153222	0.657562531	0.456377635	0.442454191
0.431012017	1.499935018	0.490491486	0.745872013	0.337509496	0.314057363
0.413769727	0.90928219	0.635956503	0.636706325	0.174923982	0.368541758
0.363014731	0.762336575	0.410997066	0.65925111	0.382279568	0.350649117
0.420535646	3.800001531	0.281030769	0.641357206	0.288451366	0.276720226
0.409007726	1.186325577	0.556595056	0.639936207	0.306444985	0.274085897
0.450000965	1.132177376	0.569039221	0.706176149	0.082956567	0.453916866

Q07108,CD69,CARDIOMETABOLIC	P35754,GLRX,CARDIOMETABOLIC	Q9H5Y7,SLITRK6,CARDIOMETABOLIC	P31431,SDC4,CARDIOMETABOLIC	P07585,DCN,CARDIOMETABOLIC
Q07108	P35754	Q9H5Y7	P31431	P07585
CD69	GLRX	SLITRK6	SDC4	DCN
CARDIOMETABOLIC	CARDIOMETABOLIC	CARDIOMETABOLIC	CARDIOMETABOLIC	CARDIOMETABOLIC
0.140233215	0.766469346	0.970096385	0.200851341	0.253296667
0.154085178	1.26242703	1.032183877	0.107261185	0.26215646
0.115927922	1.218832606	0.790315167	0.096207955	0.263796988
0.10300598	0.82262111	0.629422213	0.040978188	0.392455233
0.197743201	1.009471374	0.745872013	0.103643389	0.246660738
0.123947407	1.178866214	0.744580635	0.079159154	0.264419412
0.102884674	0.998476238	0.826393035	0.08101877	0.226612602
0.079632436	0.537598121	0.925689331	0.045635489	0.279592972
0.153967739	1.267776121	0.622350836	0.130416653	0.244448084
0.117701124	1.398034536	0.491819213	0.274961208	0.248858919
0.066164446	0.327189873	0.724872921	0.064141415	0.256975015
0.173162687	1.344341808	1.172022284	0.336295178	0.418355121
0.090747392	0.601734925	0.66062341	0.082018888	0.27177789
0.077573086	1.044635763	0.605416542	0.065757534	0.189609085
0.094732285	0.737747995	0.415407739	0.119932952	0.217411476
0.179742067	1.170885498	0.445099566	0.223384347	0.205128521
0.242927884	1.244270365	0.697130184	0.831449185	0.324682155
0.112422289	0.817788681	0.514520334	0.064663706	0.308597868
0.10698645	0.79603292	0.694477568	0.044758329	0.232080948
0.1241882	0.587760373	0.715686663	0.068336492	0.21581993
0.113235639	0.689489267	0.810340415	0.058233777	0.262065619
0.188247159	1.203136224	0.487035892	0.138657735	0.203838716
0.113557902	0.480730419	0.586214273	0.062171621	0.216269181
0.093908529	0.621402521	0.704367378	0.02824423	0.269134641
0.126376489	0.487035892	0.79680577	0.067671867	0.23338763
0.150757324	1.181483925	0.554746283	0.191484564	0.298333936
0.125625392	1.398906947	0.824962245	0.052272646	0.25268291
0.203457589	1.92145608	0.671239723	0.412338192	0.228394529
0.112757867	1.628337779	0.734024438	0.174294627	0.287015389
0.107670878	0.627679502	0.44168814	0.044304591	0.242709082
0.111367486	0.386060806	0.475791037	0.11406278	0.209250068
0.082497831	0.897447842	0.860114123	0.077058611	0.240415197
0.07531077	0.620842833	0.56848729	0.152016518	0.215879776
0.107134868	0.573672792	0.594397519	0.056218938	0.123279088
0.117554364	2.142507122	0.727742514	0.143001283	0.205427325

O75023,LILRB5,CARDIOMETABOLIC	P48745,CCN3,CARDIOMETABOLIC	P07359,GP1BA,CARDIOMETABOLIC	P41159,LEP,CARDIOMETABOLIC	Q9UK05,GDF2,CARDIOMETABOLIC
O75023	P48745	P07359	P41159	Q9UK05
LILRB5	CCN3	GP1BA	LEP	GDF2
CARDIOMETABOLIC	CARDIOMETABOLIC	CARDIOMETABOLIC	CARDIOMETABOLIC	CARDIOMETABOLIC
0.314754738	1.344434994	0.480330725	0.179829299	0.48801588
0.368337452	1.74787256	0.542201121	0.510860041	0.610134832
0.125216796	1.544457316	0.449346416	0.192843176	0.462620147
0.329557018	0.827023368	0.361508131	0.357397141	0.41612821
0.204702411	0.951911925	0.467422796	0.364098323	0.480730419
0.634503488	1.306855835	0.328826848	0.324389719	0.467228441
0.243264887	0.716034001	0.53418079	0.163617617	0.347551819
0.329123284	1.823107964	0.373815942	0.169505031	0.325764206
0.084952487	1.470798897	0.369206536	0.370488329	0.308448172
0.212347643	0.70578467	0.311304859	0.143816389	0.238986329
0.098851043	1.153805464	0.324120011	0.285903463	0.344171609
0.39359942	1.739050708	0.596957447	0.340422901	0.576223339
0.420477352	1.150451369	0.39001459	0.171823587	0.42637655
0.121152829	1.178784504	0.244295636	0.243501067	0.301431063
0.054146054	0.520980038	0.397602849	0.125660227	0.299598021
0.252507825	0.668314926	0.415436534	0.152227404	0.151837495
0.207732666	0.779975115	0.586905446	0.13351743	0.510576839
0.248514167	0.741387656	0.372806779	0.529866198	0.438941323
0.403069353	1.261377409	0.418355121	0.513487122	0.342078657
0.306912647	1.042249009	0.465224829	0.118486944	0.383766318
0.222441827	1.569603322	0.341581087	0.200211951	0.451970338
0.255430021	1.049643925	0.330655316	0.526279097	0.222997586
0.074650721	0.92108104	0.349993492	0.196977124	0.233824823
0.2555717	0.880564141	0.38533897	0.096408222	0.252630372
0.411652816	1.282003322	0.479100426	0.157017798	0.525550025
0.286737003	0.932645035	0.460508599	0.231101736	0.402371492
0.315475528	0.689537061	0.623343803	0.2310697	0.40615435
0.36938572	1.715465367	0.382041164	0.579347155	0.473455258
0.517632462	1.256839128	0.430474594	0.226864063	0.365970686
0.322171326	0.951977908	0.309948408	1.962509993	0.41425758
0.093732944	0.427441829	0.438637177	0.155415252	0.210180393
0.422024892	1.766262781	0.426317446	0.226785452	0.451187811
0.241852608	1.068213484	0.23964986	0.171466661	0.229092161
0.489778042	0.842880493	0.411938251	0.163345657	0.26559502
0.107001283	0.774587433	0.391151662	0.153403149	0.275361735

Q9GZM7,TINAGL1,CARDIOMETABOLIC	Q8NC01,CLEC1A,CARDIOMETABOLIC	Q86U17,SERPINA11,CARDIOMETABOLIC	P08887,IL6R,CARDIOMETABOLIC
Q9GZM7	Q8NC01	Q86U17	P08887
TINAGL1	CLEC1A	SERPINA11	IL6R
CARDIOMETABOLIC	CARDIOMETABOLIC	CARDIOMETABOLIC	CARDIOMETABOLIC
0.440953979	0.918912883	0.575704343	0.364578149
0.457708186	1.258146569	0.839964348	0.253244001
0.382836423	0.825763182	0.934651216	0.385713087
0.439550247	0.696164426	0.749863549	0.294267476
0.471228924	1.276771187	0.70759708	0.422522478
0.36296441	0.745768621	0.498650188	0.373401596
0.491887398	0.708382266	0.618952241	0.333001366
0.399259876	0.765991347	0.710792321	0.255997211
0.477807035	0.816995479	0.350187623	0.320834235
0.3139703	0.462684284	0.34006914	0.338493492
0.310120328	0.683020128	0.689632658	0.207013965
0.402622583	1.35388012	1.057604338	0.572084436
0.362235539	0.821367632	0.606508597	0.195711441
0.251094094	0.617238518	0.396337121	0.170660372
0.269451963	0.50117974	0.477906402	0.227935889
0.401062783	0.682215765	0.280369244	0.431280981
0.39055564	0.964531326	0.630907322	0.376781491
0.414516088	0.889828986	0.584712768	0.333347774
0.292477997	0.919550046	0.733414147	0.267794827
0.377696678	0.790205613	0.589555689	0.344601288
0.310313851	0.679148986	0.455019412	0.224175419
0.39329943	0.732347359	0.411396094	0.23266079
0.322998641	0.707842357	0.255076165	0.193191028
0.349290667	0.667157827	0.354510435	0.271589574
0.480630464	0.682830781	0.447605603	0.287892079
0.355174522	0.760278553	0.620068712	0.435426163
0.464065387	0.831103467	0.581963267	0.284086071
0.275629078	0.901187995	0.954157941	0.347744596
0.472144379	0.549960535	0.502640909	0.468330857
0.276701045	0.726986259	0.29375799	0.422141918
0.303086185	0.564795305	0.473258394	0.29993047
0.348468463	0.566677568	0.634767425	0.387965427
0.268985442	0.599944105	0.32005683	0.19533197
0.293941303	0.464354976	0.415062357	0.329123284
0.303590805	0.619510225	0.613102418	0.377330337

P13807,GYS1,CARDIOMETABOLIC	Q9Y4L1,HYOU1,CARDIOMETABOLIC	P98160,HSPG2,CARDIOMETABOLIC	P19320,VCAM1,CARDIOMETABOLIC	P16112,ACAN,CARDIOMETABOLIC
P13807	Q9Y4L1	P98160	P19320	P16112
GYS1	HYOU1	HSPG2	VCAM1	ACAN
CARDIOMETABOLIC	CARDIOMETABOLIC	CARDIOMETABOLIC	CARDIOMETABOLIC	CARDIOMETABOLIC
0.902313077	0.471261588	0.608825208	0.447574578	2.016844702
0.96828255	0.565382839	0.602862125	0.59106963	2.006804391
0.775554462	0.675955417	0.618266182	0.665495124	3.137466381
0.626636191	0.375243764	0.480697098	0.511994423	1.789305836
0.744116286	0.500277336	0.613059923	0.458565588	2.060650736
0.520582962	0.498201061	0.630994791	0.517632462	1.806002638
0.53329289	0.511887968	0.460636297	0.557908331	1.958433308
0.532332665	0.524022259	0.639271198	0.616639837	1.873583142
0.938026119	0.385766561	0.399924619	0.540475078	1.72751711
0.799627503	0.338963069	0.340493698	0.465289327	1.780891965
0.693371287	0.32873569	0.568960341	0.415666965	1.301432061
1.009191528	0.555862515	1.013678389	0.67156549	2.752800387
0.598034243	0.50291971	0.51132058	0.476946715	2.176937518
0.981595132	0.369308916	0.503861804	0.374801855	1.286900043
0.548171793	0.261684431	0.286975603	0.307658125	1.174624798
0.734482489	0.398458119	0.403041416	0.446397236	1.126775422
1.020092515	0.451845043	0.480597151	0.497510886	1.64832417
0.653382627	0.380429229	0.516056167	0.430116684	2.210541777
0.827539453	0.493971599	0.543066208	0.471915349	1.366229643
0.502048971	0.376389947	0.567463695	0.574946651	1.84894204
0.634151742	0.311585499	0.579990028	0.518242774	2.714713594
0.682404942	0.349460185	0.626723068	0.584955994	1.562872405
0.566559743	0.35566724	0.473061612	0.462107369	1.57210764
0.576303226	0.414803508	0.462139401	0.353381887	1.425828042
0.678490255	0.414544821	0.51501987	0.562958313	2.033267456
0.616426163	0.458406689	0.471555668	0.540812349	1.78967795
0.871395761	0.549350947	0.496030247	0.624122012	1.319965297
1.118759552	0.450344204	0.547905883	0.376781491	1.947198552
0.832660332	0.556749399	0.609500792	0.569749635	1.872674294
0.816372788	0.324749678	0.469598596	0.345893546	1.277390831
0.722565361	0.492842986	0.367572311	0.357496247	0.548209791
0.42281545	0.452848379	0.587312399	0.655832817	2.148455656
0.716331853	0.428985265	0.432028981	0.388395933	1.369548161
0.532664855	0.346829856	0.488760635	0.440801183	1.489677463
1.009121578	0.479465861	0.405563578	0.49520576	1.543601125

Q96A56,TP53INP1,CARDIOMETABOLIC	P09382,LGALS1,CARDIOMETABOLIC	P17813,ENG,CARDIOMETABOLIC	Q16627,CCL14,CARDIOMETABOLIC	P10586,PTPRF,CARDIOMETABOLIC
Q96A56	P09382	P17813	Q16627	P10586
TP53INP1	LGALS1	ENG	CCL14	PTPRF
CARDIOMETABOLIC	CARDIOMETABOLIC	CARDIOMETABOLIC	CARDIOMETABOLIC	CARDIOMETABOLIC
0.972183127	0.381432585	0.594727215	0.471490301	0.440678984
0.957736042	0.383580158	0.614123196	0.404048393	0.474473691
1.116745158	0.490321524	0.518530228	0.376912096	0.431939153
0.883988802	0.320834235	0.465837924	0.356407598	0.351086883
0.969155454	0.597081594	0.698000512	0.480730419	0.435214944
1.074153371	0.468265937	0.548171793	0.424606982	0.315409933
1.075419845	0.26811057	0.522245478	0.519105616	0.314972985
1.117751901	0.338986565	0.452409146	0.543480433	0.311563903
1.183204956	0.310270836	0.429193461	0.532923368	0.360432248
0.94487802	0.301891073	0.385098658	0.412509715	0.239600031
1.045215193	0.275075585	0.470771863	0.44260756	0.274789732
1.018044059	0.40287383	0.739642485	0.603991436	0.540063143
1.084026481	0.31985723	0.485048192	0.351232926	0.306168974
0.998891579	0.225078475	0.353088074	0.344100048	0.271288538
0.994676984	0.187154287	0.364401297	0.311175418	0.17640948
0.981254996	0.304243849	0.441565695	0.460285213	0.286856278
0.976302412	0.246319031	0.549846186	0.635692071	0.263650749
0.928130773	0.397823388	0.457486159	0.507260947	0.287553041
1.029468724	0.295309567	0.50445588	0.489845944	0.290820324
1.000970877	0.36389648	0.505260744	0.467131293	0.409603515
1.10297493	0.397382432	0.384671806	0.357347599	0.244804164
1.003332647	0.536890581	0.379428512	0.460253309	0.436483796
0.918594468	0.385927031	0.4499074	0.406351465	0.339151082
1.370497788	0.191577495	0.400784883	0.433288545	0.25535921
1.070437082	0.379665286	0.49520576	0.339880618	0.402790064
0.993161334	0.266443215	0.570777354	0.54092482	0.393926943
0.904943735	0.294104344	0.574030779	0.561243991	0.487441165
1.224760789	0.296334807	0.447419488	0.481630947	0.455082495
1.099387514	0.38536568	0.585889297	0.548970298	0.394774306
0.998130253	0.403824403	0.396831925	0.601234624	0.31281899
0.878552253	0.277642437	0.506839195	0.593820992	0.351232926
0.869284304	0.39829244	0.541187342	0.603782145	0.38536568
0.970769038	0.251581894	0.374594079	0.332102388	0.329557018
1.076837085	0.339127575	0.3842987	0.487474953	0.367037659
1.040805146	0.247122795	0.515162684	0.656196589	0.335224617

O14798,TNFRSF10C,CARDIOMETABOLIC	Q9H773,DCTPP1,CARDIOMETABOLIC	Q9NQ79,CRTAC1,CARDIOMETABOLIC	O95183,VAMP5,CARDIOMETABOLIC
O14798	Q9H773	Q9NQ79	O95183
TNFRSF10C	DCTPP1	CRTAC1	VAMP5
CARDIOMETABOLIC	CARDIOMETABOLIC	CARDIOMETABOLIC	CARDIOMETABOLIC
0.363846036	0.453791031	0.310658191	1.145517898
0.442699608	0.398485739	0.193324984	1.013818925
0.531963808	0.554784736	0.211363769	1.000485321
0.414746008	0.419836645	0.46998936	1.289936458
0.526096734	0.578103615	0.383686524	1.986735755
0.151995446	0.534477085	0.261032256	0.817731998
0.494828328	0.475263661	0.205598266	1.043839571
0.615060401	0.341226122	0.228394529	1.025480458
0.520582962	0.333972218	0.20562677	2.294214048
0.189714256	0.331688293	0.206412179	1.760884155
0.217065146	0.314798375	0.320589704	0.844635038
0.521269011	0.697226834	0.383739718	1.196483084
0.182731847	0.391558562	0.254158432	0.901375411
0.268836326	0.312645574	0.209279079	1.104198847
0.288211539	0.230046913	0.226173216	0.885521966
0.42581539	0.356679449	0.163221159	0.80179204
0.548513867	0.43452166	0.437756347	0.856781955
0.409575124	0.450125749	0.290296684	1.252664439
0.43615112	0.426022048	0.292640226	1.464897783
0.225328234	0.338235502	0.212686446	1.134298227
0.218544655	0.488049708	0.223709744	1.393777239
0.374101071	0.616169852	0.098789396	1.084853325
0.351744556	0.439611186	0.167217761	0.78747171
0.338001136	0.273725169	0.211217313	1.473554071
0.412652704	0.435848908	0.114689085	0.800625793
0.413110605	0.360907243	0.246712035	0.768277815
0.522716281	0.235565473	0.161790634	1.227650587
0.323043421	0.366656241	0.10628427	1.206476655
0.225765978	0.365159837	0.287912035	0.895769842
0.386301718	0.337065296	0.173920512	1.222470796
0.342814491	0.270631186	0.207574338	0.885276481
0.21137842	0.410484599	0.158340257	1.178866214
0.375087737	0.356358193	0.184385869	1.682725673
0.167507779	0.315781815	0.131651877	1.067177386
0.548399819	0.262138289	0.224891338	1.285117257

Q9Y2B0,CNPY2,CARDIOMETABOLIC	P27352,CBLIF,CARDIOMETABOLIC	P31146,CORO1A,CARDIOMETABOLIC	Q13740,ALCAM,CARDIOMETABOLIC	P16234,PDGFRA,CARDIOMETABOLIC
Q9Y2B0	P27352	P31146	Q13740	P16234
CNPY2	CBLIF	CORO1A	ALCAM	PDGFRA
CARDIOMETABOLIC	CARDIOMETABOLIC	CARDIOMETABOLIC	CARDIOMETABOLIC	CARDIOMETABOLIC
0.616768077	0.118988991	0.425019223	0.631344786	0.580231289
0.725928821	0.251285617	0.41838412	0.489438671	0.754399173
0.558488702	0.314427651	0.467228441	0.601151281	0.897323438
0.58422662	0.216509165	0.461531173	0.503373091	0.618523365
0.771533123	0.097382072	0.830873068	0.626419053	0.660028396
0.847332435	0.331205836	0.430534275	0.508916192	0.821196851
1.755400228	0.164071891	0.38969032	0.45015695	0.681932099
0.746699671	0.168930297	0.333278464	0.427145651	0.759699091
0.816768991	0.134483399	0.393872337	0.424960307	0.508880918
0.713705107	0.140846928	0.384885173	0.377199587	0.434160386
0.676752398	0.164516022	0.429371994	0.451563255	0.412738522
0.856900738	0.238572556	0.599196042	0.734075318	0.771586603
0.707548035	0.127776993	0.488828396	0.440343111	0.690732973
1.126072722	0.214507501	0.37428263	0.359334659	0.491614714
0.534254848	0.156398654	0.35697625	0.36759779	0.37298771
0.67633035	0.220660454	0.517668343	0.358190753	0.382306067
0.556749399	0.150809582	0.541487523	0.526315577	0.439397937
1.60402875	0.428777171	0.448475165	0.501422973	0.517381366
0.677503357	0.24878993	0.571569168	0.528216034	0.432688295
0.77201458	0.211921226	0.533403797	0.480897056	0.601443033
0.704465031	0.226298668	0.499515032	0.391694289	0.78583591
0.594273931	0.177624193	0.512491506	0.386810805	0.623257395
0.451845043	0.297982601	0.532738703	0.401452165	0.515376979
1.358297971	0.108217071	0.406266975	0.395212368	0.473094404
0.601734925	0.127043987	0.449471018	0.521955964	0.624684657
0.660806599	0.179306538	0.407112663	0.498995946	0.488218883
1.238333582	0.180441114	0.359957878	0.529315571	0.459392752
0.826335756	0.125451359	0.636000586	0.445933349	0.532258873
1.01832636	0.190082813	0.466775259	0.487238487	0.648734469
0.6012763	0.114419115	0.391830064	0.392428031	0.52471284
0.963796188	0.349532861	0.418152183	0.455619058	0.402148432
0.560699621	0.285448028	0.378509129	0.503373091	0.885521966
0.846862704	0.168696272	0.457042427	0.359011012	0.46998936
0.705393409	0.220767545	0.4248425	0.44217826	0.456915725
0.664481068	0.151669195	0.426051578	0.559845248	0.43259833

P55774,CCL18,CARDIOMETABOLIC	P55285,CDH6,CARDIOMETABOLIC	P18065,IGFBP2,CARDIOMETABOLIC	P21583,KITLG,CARDIOMETABOLIC	P20062,TCN2,CARDIOMETABOLIC
P55774	P55285	P18065	P21583	P20062
CCL18	CDH6	IGFBP2	KITLG	TCN2
CARDIOMETABOLIC	CARDIOMETABOLIC	CARDIOMETABOLIC	CARDIOMETABOLIC	CARDIOMETABOLIC
0.213380464	0.614889894	0.233824823	0.507471955	0.439184791
0.181457048	0.667527881	0.25518227	0.589678296	0.447512535
0.263669024	0.642514091	0.268166327	0.531411001	0.451782408
0.096964469	0.435365804	0.426849679	0.286796635	0.568369089
0.149373901	0.395870372	0.235190224	0.418355121	0.523949619
0.327053827	0.55194637	0.209453225	0.437392382	0.386730378
0.095120493	0.366681656	0.158878961	0.314231562	0.423431352
0.343766294	0.422610348	0.303548721	0.385953782	0.376024873
0.136030305	0.366199062	0.171157925	0.30120132	0.350989554
0.150799129	0.364224531	0.175033139	0.26235642	0.389177444
0.176813459	0.323133	0.324682155	0.223973508	0.277353916
0.176764442	0.932903655	0.285546974	0.548057816	0.53629548
0.126894373	0.393626703	0.184987539	0.322417063	0.406098049
0.167984497	0.359583817	0.147358277	0.303212261	0.415378946
0.090980426	0.291001804	0.177611881	0.232467349	0.256085949
0.471000338	0.333209168	0.232338477	0.273895981	0.344481879
0.239799408	0.367343079	0.290477837	0.56003931	0.526498016
0.232854392	0.356902027	0.231566747	0.428212852	0.34193642
0.309068815	0.439215234	0.159231757	0.360107612	0.456061408
0.157978485	0.634811426	0.352501182	0.338399654	0.389123496
0.156138693	0.460412849	0.206770174	0.557985679	0.359135457
0.206712853	0.39066394	0.121995514	0.39172144	0.431879278
0.122937759	0.318838997	0.224501968	0.264951463	0.34484023
0.130470903	0.420973113	0.169317148	0.323536414	0.333463324
0.327348665	0.550265582	0.271288538	0.319724233	0.475296604
0.170908967	0.330151476	0.412824357	0.371053726	0.411738426
0.125381813	0.46823348	0.27268363	0.476682313	0.439397937
0.216734391	0.358613076	0.13608689	0.369411325	0.446428179
0.138034423	0.60023527	0.445253853	0.45584018	0.481664332
0.280155554	0.332010322	0.169223285	0.432418455	0.372264513
0.130425693	0.287553041	0.280913916	0.290739703	0.438454791
0.095444111	0.507296109	0.343790122	0.448941696	0.326397067
0.147143937	0.394883776	0.112531437	0.25605045	0.452064333
0.116774729	0.288831501	0.169575541	0.350236173	0.319502694
0.296560838	0.406999803	0.339033561	0.307786103	0.589147181

Q9Y5C1,ANGPTL3,CARDIOMETABOLIC	O75356,ENTPD5,CARDIOMETABOLIC	P20718,GZMH,CARDIOMETABOLIC	P04066,FUCA1,CARDIOMETABOLIC	O60496,DOK2,CARDIOMETABOLIC
Q9Y5C1	O75356	P20718	P04066	O60496
ANGPTL3	ENTPD5	GZMH	FUCA1	DOK2
CARDIOMETABOLIC	CARDIOMETABOLIC	CARDIOMETABOLIC	CARDIOMETABOLIC	CARDIOMETABOLIC
0.448350838	0.688629552	0.113133649	0.348275284	0.742724977
0.36617368	0.53362568	0.186933884	0.104472852	0.875998315
0.456884055	0.595799988	0.131935069	0.428420672	0.877760954
0.439550247	0.443928735	0.227415107	0.349072836	0.907016059
0.450344204	0.693611633	0.137252088	0.682641487	0.819775057
0.355913855	0.460604369	0.106564587	0.60634046	1.427212346
0.398458119	0.626462475	0.1698461	0.74597542	1.150770386
0.375894575	0.416416748	0.108374707	0.712074453	0.701833151
0.38209413	0.555785461	0.25491709	0.419400359	0.785726977
0.277430825	0.462780506	0.279922624	0.187297039	0.572084436
0.434431313	0.505786347	0.16191404	0.697516863	0.742158893
0.674317526	0.730066608	0.27816253	0.431669779	0.826679491
0.375712234	0.714794283	0.185231325	0.752101876	0.828228068
0.321034444	0.414142739	0.234896969	0.397107083	0.815637493
0.2047166	0.528142813	0.122046261	0.34276697	0.762706553
0.184500931	0.481564183	0.497890364	0.217230713	0.728600555
0.486260059	0.772549886	0.159110395	0.403153178	0.939653004
0.2859431	0.737185705	0.174730093	0.931288456	0.574309368
0.309733643	0.705980382	0.112336604	0.284027003	0.88687335
0.324502163	0.590005374	0.147839122	0.580834881	0.960062345
0.41065535	0.455177137	0.122835545	0.381036208	0.808488972
0.360257407	0.402009082	0.09338923	0.538157363	0.966137211
0.351452105	0.408384496	0.087492308	0.257617053	0.714150478
0.13241147	0.968014122	0.120407737	0.507999858	0.463037197
0.269938002	0.438029518	0.188979284	0.639093979	1.099692372
0.352501182	0.564912763	0.206884863	0.515198394	0.979556083
0.356803086	0.615700225	0.18297266	0.107790355	1.31494276
0.346589535	0.571410717	0.17025865	0.42135262	1.326385371
0.555823986	0.765036242	0.184884989	0.607855369	0.904567458
0.377958568	0.519825748	0.092525831	0.39033913	0.879710051
0.281420631	0.423431352	0.130561369	0.441596303	0.872786081
0.408356189	0.511568735	0.076505114	0.685866644	0.623170999
0.306062883	0.5030243	0.184424215	0.682972787	0.632089169
0.2761454	0.499757457	0.104552539	0.335619861	0.737032427
0.425019223	0.739181216	0.534143765	0.555284873	0.44723345

Q9UBU3,GHRL,CARDIOMETABOLIC	O94903,PLPBP,CARDIOMETABOLIC	P48960,CD97,CARDIOMETABOLIC	Q14767,LTBP2,CARDIOMETABOLIC	P09668,CTSH,CARDIOMETABOLIC
Q9UBU3	O94903	P48960	Q14767	P09668
GHRL	PLPBP	CD97	LTBP2	CTSH
CARDIOMETABOLIC	CARDIOMETABOLIC	CARDIOMETABOLIC	CARDIOMETABOLIC	CARDIOMETABOLIC
0.399204531	0.890322549	0.428301905	0.309647778	0.290457704
0.251616773	0.816655771	0.391531422	0.371233806	0.43915435
0.318309032	0.978809494	0.424636415	0.409688698	0.383952567
0.34958132	0.652387028	0.473652203	0.237401343	0.241065985
0.70207643	0.818525916	0.646131627	0.304328215	0.272475799
0.42788648	0.892732591	0.463840275	0.289212137	0.330426202
0.151301689	0.78398611	0.438941323	0.310227826	0.210253249
0.208873301	0.491887398	0.408299583	0.294247079	0.203471692
0.342363309	0.651573575	0.367190337	0.266554049	0.211114855
0.298644281	0.998891579	0.324164947	0.178649032	0.224299763
0.385686352	0.667851846	0.271608399	0.223678733	0.288031799
0.658474739	1.132726845	0.529645878	0.499515032	0.453539466
0.313426702	0.74277646	0.315125848	0.345414368	0.18595172
0.268408079	0.742879438	0.287732482	0.245092799	0.197990073
0.184641659	0.67156549	0.268705918	0.211231954	0.241233137
0.190808851	1.016070143	0.267460918	0.177956927	0.199962309
0.249826773	1.5244618	0.361007321	0.303717091	0.252280395
0.365008003	0.621402521	0.412452532	0.222256883	0.344100048
0.154823889	0.516235049	0.399287552	0.243991027	0.282515123
0.198828986	0.872907084	0.432958305	0.243113178	0.346133384
0.147256171	0.670774616	0.372445181	0.241634775	0.306381268
0.093642029	1.01550687	0.333902778	0.26197481	0.460412849
0.122173221	0.647162537	0.379586345	0.257206677	0.305173163
0.112359966	0.576902732	0.285725163	0.201395032	0.583174685
0.310873598	0.769930432	0.40671779	0.265760758	0.320611927
0.21358763	1.023066554	0.3455341	0.293941303	0.325087503
0.187869139	0.880198001	0.289031773	0.26811057	0.175239511
0.339080565	1.374207631	0.311304859	0.299556491	0.53119004
0.284401307	1.127947563	0.426317446	0.436756173	0.249844091
0.134585977	0.892546971	0.350624813	0.222410993	0.253630474
0.362235539	0.658566029	0.313665769	0.177328951	0.324097546
0.327689193	0.787144277	0.462652214	0.328599001	0.286876163
0.114379468	0.676517894	0.322215991	0.225781628	0.472766593
0.157377369	0.698436083	0.357199013	0.212038773	0.430743222
0.24865201	1.438833844	0.360257407	0.212539074	0.43724082

P15907,ST6GAL1,CARDIOMETABOLIC	P59665,DEFA1_DEFA1B,CARDIOMETABOLIC	Q86VB7,CD163,CARDIOMETABOLIC	P08174,CD55,CARDIOMETABOLIC
P15907	P59665	Q86VB7	P08174
ST6GAL1	DEFA1_DEFA1B	CD163	CD55
CARDIOMETABOLIC	CARDIOMETABOLIC	CARDIOMETABOLIC	CARDIOMETABOLIC
0.290558386	0.432088878	0.361132458	0.377749042
0.405142124	0.444667848	0.609923412	0.439458854
0.349847964	0.615828269	0.526096734	0.436544309
0.43259833	0.380139277	0.420885584	0.394965898
0.43178948	0.55064713	0.548095806	0.372574283
0.257295833	0.427827167	0.333810213	0.51845835
0.316636615	0.475922973	0.332563099	0.379428512
0.311607098	0.394610158	0.259175275	0.467260827
0.330701157	0.451594556	0.345845598	0.354854621
0.301222199	0.359858091	0.291021975	0.30120132
0.199934591	0.375972748	0.256281279	0.350989554
0.451625859	0.39302691	0.338493492	0.54092482
0.285092106	0.36966747	0.193646858	0.393190399
0.19994845	0.312082637	0.264272828	0.29930743
0.215222378	0.316768328	0.221641509	0.235239136
0.290598669	0.475362499	0.342553208	0.275361735
0.464676954	0.379928541	0.336038864	0.447450501
0.339127575	0.338916082	0.475857	0.339245128
0.345342549	0.439428394	0.388799967	0.35697625
0.259948908	0.36645298	0.499896039	0.37822064
0.221365147	0.448288688	0.380851373	0.321992725
0.370334279	0.338986565	0.525877982	0.330128592
0.214002566	0.366732493	0.482332524	0.291365103
0.260508075	0.348347714	0.143467911	0.33028881
0.257492087	0.350066278	0.265963468	0.347841025
0.335899138	0.422376068	0.409376445	0.443467413
0.356086587	0.392047401	0.589433107	0.450281778
0.366605415	0.389393309	0.28651846	0.28779232
0.409206226	0.757858283	0.439885515	0.428242534
0.198017522	0.465805636	0.259462869	0.415350156
0.293635845	0.333972218	0.207200588	0.350649117
0.254881753	0.381591251	0.41402793	0.387427965
0.261394375	0.448226546	0.430206134	0.330243026
0.262938991	0.377304183	0.345845598	0.300533975
0.388046111	0.443436675	0.466290195	0.33401852

O60664,PLIN3,CARDIOMETABOLIC	P00568,AK1,CARDIOMETABOLIC	Q969P0,IGSF8,CARDIOMETABOLIC	P41222,PTGDS,CARDIOMETABOLIC	P24821,TNC,CARDIOMETABOLIC
O60664	P00568	Q969P0	P41222	P24821
PLIN3	AK1	IGSF8	PTGDS	TNC
CARDIOMETABOLIC	CARDIOMETABOLIC	CARDIOMETABOLIC	CARDIOMETABOLIC	CARDIOMETABOLIC
0.641446123	1.235418637	0.546313129	0.500381376	1.002220533
0.813210078	1.186736798	0.52278875	0.737543476	0.78905622
0.806529943	1.126775422	0.55856613	0.529535753	1.169830898
0.602360887	0.857197769	0.467973908	0.493287283	0.997646074
0.805747661	1.190939396	0.540100579	0.587597434	1.265844333
0.668592928	0.882458293	0.529939658	0.446273486	1.056432068
0.630513865	0.957205107	0.436362793	0.496580667	1.070140335
0.741901725	1.056432068	0.359309753	0.502571233	0.991304373
0.506277404	0.878430468	0.444205758	0.472275303	0.942065973
0.672310693	1.337371242	0.356555855	0.390826446	0.798851916
0.555130937	0.749811574	0.370565378	0.388342094	0.837116315
0.779002574	1.593722005	0.537896311	0.789165614	0.984934711
0.550685299	0.966941155	0.480697098	0.43869799	1.001179045
0.52188361	0.855002178	0.310507496	0.426642621	0.521052266
0.448910579	0.753197439	0.309240247	0.293615493	0.64470005
0.600818025	1.386165964	0.382571153	1.296659787	0.566010217
0.585929909	2.043440165	0.535738173	0.497028333	0.879466177
0.561633151	1.175276328	0.408327885	0.466678206	0.768917117
0.690876622	0.676377231	0.455240242	0.430832802	1.24616923
0.494416913	0.767000807	0.393981557	0.565343651	0.896950329
0.745613559	0.82262111	0.477012838	0.444236549	1.160543018
0.630426463	1.030611074	0.495137114	0.440099	0.736215485
0.480397317	0.558721019	0.410626887	0.441810619	0.726835102
0.484913727	0.565304466	0.326012684	0.387427965	0.666418336
0.757543164	0.808881348	0.491444362	0.422962012	0.820855396
0.541187342	1.440530294	0.4768806	0.504770674	1.43296165
0.600360099	1.009751298	0.359434302	0.610938896	0.511604195
0.609205132	1.662552617	0.463551007	0.49120597	0.80068129
0.679761237	1.574070327	0.435637484	0.49806295	0.916432165
0.706567845	0.874542257	0.38106262	0.490525486	0.642781361
0.530785182	0.60869862	0.449346416	0.435275282	0.491240019
0.653201496	1.048553156	0.476550167	0.537039459	0.968819629
0.465805636	0.666880422	0.396749415	0.41425758	0.597909898
0.584145634	0.894714933	0.375842469	0.501040803	0.880442077
0.515555626	1.884785049	0.482399394	0.445933349	0.834509281

P06681,C2,CARDIOMETABOLIC	Q13444,ADAM15,CARDIOMETABOLIC	P16581,SELE,CARDIOMETABOLIC	Q5VY43,PEAR1,CARDIOMETABOLIC	Q04760,GLO1,CARDIOMETABOLIC
P06681	Q13444	P16581	Q5VY43	Q04760
C2	ADAM15	SELE	PEAR1	GLO1
CARDIOMETABOLIC	CARDIOMETABOLIC	CARDIOMETABOLIC	CARDIOMETABOLIC	CARDIOMETABOLIC
0.389555287	0.603698449	1.008142796	0.56750303	0.265945033
0.337720111	0.618995145	1.134612765	0.556363623	0.616725327
0.288951647	0.632439771	1.00542119	0.493458273	0.481063751
0.313035895	0.477145113	0.37192922	0.452064333	0.361859111
0.342909553	0.933679945	0.83613048	0.622135183	0.508070287
0.284993318	0.617452474	0.55171687	0.467293217	0.530821974
0.303422505	0.512278412	0.508951469	0.49948041	0.597495602
0.260977982	0.455398044	0.505155689	0.483604643	0.408356189
0.329009238	0.414056629	1.365282973	0.428836616	0.46616093
0.22487575	0.53968893	0.531337337	0.349678258	0.707793295
0.207603116	0.567975262	0.804296864	0.449346416	0.374490234
0.408356189	0.986437807	1.102592735	0.567424363	0.257242336
0.218211645	0.572997202	0.932838993	0.42443043	0.492160234
0.213765361	0.41529258	0.463165596	0.399010883	0.506382692
0.174814893	0.580472651	0.575943821	0.343980813	0.553363733
0.273042986	0.460444763	0.646221206	0.429610155	0.488930056
0.305046271	0.639847499	1.042465761	0.461467196	1.629466848
0.277353916	0.710989422	0.601026289	0.47813834	0.383899344
0.32983125	0.644342651	1.455182401	0.508704584	0.401007187
0.25	0.473947775	1.761738748	0.536035331	0.312320679
0.30587201	0.607518396	0.440831738	0.434822952	0.296931077
0.355149904	0.29711637	1.150052722	0.369744348	0.826106679
0.219516304	0.35539616	1.011432471	0.394500764	0.547716026
0.202655326	0.414343731	0.460221408	0.440740079	0.622307699
0.247362721	0.566049451	0.538866573	0.51630662	0.43915435
0.429401757	0.489744094	0.983979388	0.555554364	0.822906258
0.262556533	0.543781886	0.792509426	0.575664439	0.580191072
0.244905996	0.459297234	1.06201195	0.476021948	1.267248978
0.308213081	0.551908114	1.458413681	0.511143401	0.755183946
0.311823162	0.621101088	0.838045222	0.471228924	0.44664484
0.253929515	0.337907435	0.509763506	0.449408713	0.406689599
0.294736982	0.402399384	1.021082899	0.538045468	0.42401876
0.193700556	0.39166714	0.458184322	0.370000724	0.402009082
0.218423502	0.448506252	0.232016611	0.369975079	0.325312914
0.405085963	0.4430987	0.971105539	0.444482955	1.479490056

Q13231,CHIT1,CARDIOMETABOLIC	P13686,ACP5,CARDIOMETABOLIC	P00441,SOD1,CARDIOMETABOLIC	P12111,COL6A3,CARDIOMETABOLIC	P15144,ANPEP,CARDIOMETABOLIC
Q13231	P13686	P00441	P12111	P15144
CHIT1	ACP5	SOD1	COL6A3	ANPEP
CARDIOMETABOLIC	CARDIOMETABOLIC	CARDIOMETABOLIC	CARDIOMETABOLIC	CARDIOMETABOLIC
0.123913047	0.557521752	0.998891579	0.548361808	0.592217899
0.11581548	0.669010148	0.967209285	0.800071034	0.615145672
0.176629718	0.544121221	1.069621226	0.732956762	0.623776021
0.240965749	0.504560789	0.889643971	0.469631147	0.59291615
0.157770568	0.484107718	0.887426783	0.581358502	0.473619373
0.0552225	0.485384519	0.94258851	0.621230256	0.600318486
0.235010969	0.531116406	1.054310638	0.522607596	0.711581052
0.213173498	0.525914435	0.792894047	0.625204471	0.677597285
0.152946606	0.403824403	0.968886784	0.405901056	0.460636297
0.071520469	0.271288538	0.982820599	0.338188616	0.481931498
0.122750431	0.438606774	0.882030225	0.437908088	0.465353834
0.139119832	0.715042054	1.293069662	1.298098626	0.628811715
0.173030708	0.543932675	0.842763653	0.621962714	0.731383508
0.08337161	0.393763147	0.992060493	0.555554364	0.467455196
0.053641746	0.397740672	0.837522585	0.265190316	0.37353103
0.138504043	0.312753948	1.04066087	0.380640242	0.434371093
0.212318207	0.415263795	1.916800248	0.378719077	0.586092386
0.062474012	0.431580025	0.915733686	0.512171897	0.523441422
0.19030693	0.409546736	0.842471624	0.465934802	0.679196062
0.133018609	0.585848688	0.798243054	0.691355667	0.624078753
0.043730995	0.40983071	0.778031246	0.541525057	0.406633224
0.245177756	0.508175948	1.174543382	0.623300597	0.43277828
0.275705509	0.43593955	0.86910356	0.477807035	0.378719077
0.075373437	0.320834235	0.739796305	0.532258873	0.478436711
0.150517173	0.515627102	0.802848679	0.550456323	0.431550112
0.293473064	0.423431352	1.22858698	0.499341944	0.579588149
0.123432995	0.692170807	1.301973424	0.513878786	0.602360887
0.201227586	0.55321033	1.586117885	0.713507253	0.47404634
0.381406147	0.569710144	1.481850597	0.693948257	0.639359826
0.255943984	0.420798072	0.834335768	0.37051401	0.540250347
0.078334953	0.391178775	0.886750412	0.375243764	0.469728814
0.158724859	0.680987395	0.910732959	0.791905398	0.455429611
0.080951409	0.374023287	0.906576079	0.457613018	0.380851373
0.074397606	0.446954538	0.898443696	0.463101392	0.437604658
0.003963251	0.506137053	1.695134796	0.333671414	0.497166158

P22748,CA4,CARDIOMETABOLIC	P07339,CTSD,CARDIOMETABOLIC	P48304,REG1B,CARDIOMETABOLIC	P24592,IGFBP6,CARDIOMETABOLIC	P12724,RNASE3,CARDIOMETABOLIC
P22748	P07339	P48304	P24592	P12724
CA4	CTSD	REG1B	IGFBP6	RNASE3
CARDIOMETABOLIC	CARDIOMETABOLIC	CARDIOMETABOLIC	CARDIOMETABOLIC	CARDIOMETABOLIC
0.410484599	0.587271691	0.281342615	0.28247596	0.142694338
0.32185884	0.738157203	0.285507391	0.375634115	0.250277413
0.395157583	0.592176851	0.397162138	0.227951688	0.295801239
0.350867932	0.686104389	0.256547878	0.267869086	0.204872749
0.446830633	0.607981782	0.310981357	0.241785561	0.281225632
0.42513708	0.596667872	0.434280777	0.349048641	0.30367499
0.419953064	0.527338049	0.302918165	0.285903463	0.139399762
0.302351785	0.551143537	0.309926925	0.321702711	0.174839129
0.299037849	0.643093316	0.196690611	0.218590105	0.486125258
0.26999414	0.569433786	0.322864338	0.198347209	0.206469416
0.281850103	0.711433098	0.328599001	0.267164458	0.217065146
0.484678503	0.680279726	0.272853792	0.501422973	0.226298668
0.281050249	0.61813763	0.175385332	0.21032613	0.230701614
0.327167195	0.597164372	0.171312224	0.243568589	0.16177942
0.298540797	0.623689554	0.144225683	0.104973713	0.159154516
0.37433452	0.64166847	0.229489492	0.244058685	0.610515573
0.367725212	0.615145672	0.322193658	0.255306115	0.200364663
0.329968452	0.585199321	0.194157588	0.245432805	0.443713391
0.373298082	0.616169852	0.200948818	0.24033189	0.210063877
0.330220136	0.660531835	0.310356873	0.233727598	0.335201381
0.266554049	0.651167229	0.39055564	0.229887512	0.300700673
0.264419412	0.661494011	0.307679451	0.270499906	0.40301348
0.280699811	0.655015068	0.390149782	0.232113124	0.332885976
0.354289349	0.589882698	0.388207528	0.256263515	0.13737582
0.37950742	0.647341993	0.391287248	0.273858013	0.26110464
0.273440719	0.731839911	0.276509317	0.231663073	0.187413918
0.432928295	0.594809667	0.27046241	0.379402213	0.180028847
0.359409388	0.508810377	0.22433086	0.204730791	0.197359791
0.451970338	0.660394495	0.336365116	0.340163441	0.228426194
0.344625175	0.655015068	0.329009238	0.286300084	0.205057441
0.292518546	0.511958935	0.270106451	0.500103983	0.096368135
0.31822079	0.562880276	0.261992969	0.225140889	0.138859713
0.312125904	0.599071456	0.978063473	0.240132071	0.197018089
0.332378738	0.572758949	0.24813549	0.20083742	0.146208592
0.347624098	0.717226153	0.239766167	0.2382916	0.610600214

P01241,GH1,CARDIOMETABOLIC	Q8NI22,MCFD2,CARDIOMETABOLIC	O75354,ENTPD6,CARDIOMETABOLIC	P19429,TNNI3,CARDIOMETABOLIC	P05231,IL6,CARDIOMETABOLIC
P01241	Q8NI22	O75354	P19429	P05231
GH1	MCFD2	ENTPD6	TNNI3	IL6
CARDIOMETABOLIC	CARDIOMETABOLIC	CARDIOMETABOLIC	CARDIOMETABOLIC	CARDIOMETABOLIC
1.053507073	0.470902406	0.660074148	0.221933599	0.516987033
34.44684627	0.514663009	0.672543738	0.242675438	0.423901213
1.225610022	0.706861759	0.616639837	0.113786397	0.775930854
0.549808075	0.518817842	0.570619122	0.061690836	0.541337411
3.378305976	0.454389058	0.655832817	0.18451372	1.222470796
8.228902333	0.708775185	0.499376557	0.10571119	0.595057093
41.16653167	0.508175948	0.590168981	0.06811895	0.40883766
0.863279719	0.55041817	0.480264141	0.111382926	0.241467344
2.64634288	0.42581539	0.515234106	0.048187408	0.358091456
0.487204715	0.515162684	0.523695459	0.324727169	0.72386873
0.421060661	0.441412687	0.498028428	0.064672671	0.386435623
4.469458341	0.622609718	0.833122185	0.242053859	1.089676579
23.92422026	0.58394322	0.343718641	0.142003658	0.229696376
58.65165854	0.308961719	0.54178787	0.09047732	0.347841025
28.3799472	0.300471487	0.503617388	0.151333155	0.393272169
25.59340156	0.390474435	0.465708784	0.19951927	0.279166939
1.487613762	0.39713461	0.677738202	0.096823429	0.453445164
0.961460838	0.357868136	0.669195663	0.13300017	1.023492124
13.42825562	0.470935048	0.582245706	0.281771968	1.543601125
77.36987297	0.333116795	0.494485458	0.125460055	0.578263922
1.006257823	0.416763258	0.546426744	0.106801218	0.83294896
2.460412141	0.652025368	0.500485439	0.129713451	1.041093758
1.796389258	0.394282067	0.390609786	0.106269537	0.656788149
1.911890635	0.404132421	0.659296807	0.078776001	0.382041164
121.3390407	0.751059963	0.709266641	0.284243645	0.334226956
0.683067473	0.476451081	0.462011286	0.094058361	0.339951302
1.496196851	0.47463816	0.521558144	0.163980935	0.523513991
3.190759785	0.355815188	0.627766522	0.100509237	0.45081268
3.143561526	0.446057005	0.46771448	0.127555764	0.591274515
0.315060326	0.323446723	0.576502992	0.179929045	0.739591218
1.396388132	0.433168428	0.418819349	0.099386933	0.22047699
2.287227704	0.398154426	0.574030779	0.071233515	0.304665913
2.81864151	0.194548262	0.507471955	0.130290157	0.326397067
2.909961367	0.41612821	0.43491338	0.146767048	0.372083933
4.472247404	0.453476596	0.565931757	0.146991028	0.357025741

Q14515,SPARCL1,CARDIOMETABOLIC	Q15831,STK11,CARDIOMETABOLIC	P16109,SELP,CARDIOMETABOLIC	Q15828,CST6,CARDIOMETABOLIC	P52888,THOP1,CARDIOMETABOLIC
Q14515	Q15831	P16109	Q15828	P52888
SPARCL1	STK11	SELP	CST6	THOP1
CARDIOMETABOLIC	CARDIOMETABOLIC	CARDIOMETABOLIC	CARDIOMETABOLIC	CARDIOMETABOLIC
0.655060472	0.910732959	0.339104069	0.425196021	0.469468414
0.537113914	0.791137301	0.38209413	0.405366845	0.46706654
0.616255277	0.75675594	0.37192922	0.443559639	0.453759578
0.4765832	0.775930854	0.293391708	0.416243601	0.447512535
0.502466737	0.965467767	0.522752514	0.30065899	0.505085664
0.595923894	0.55856613	0.291042148	0.701492702	0.56268523
0.50557604	0.943765278	0.324389719	0.663100754	0.360882227
0.439093475	0.656105627	0.178896864	0.510435297	0.403153178
0.37288431	0.952175887	0.400201922	0.280952862	0.414631032
0.315519265	0.655015068	0.311650299	0.356308795	0.34238704
0.542088385	0.717524501	0.240815474	0.311283282	0.359135457
0.763341218	0.827080695	0.626679628	0.850450988	0.651573575
0.45952014	0.419778447	0.290276563	0.386034047	0.430027253
0.448786132	0.995435677	0.236088553	0.435094293	0.308704838
0.26061644	0.637677994	0.229123922	0.277411596	0.250086658
0.327757341	0.694862775	0.389042588	0.451782408	0.368822864
0.766150647	0.835203695	0.650490548	0.201842236	0.544800525
0.472177107	0.631169764	0.302309873	0.336178648	0.432718288
0.269732263	0.778732639	0.333393989	0.372470998	0.476979775
0.535070168	0.504910645	0.372264513	0.411538697	0.387911647
0.44614977	0.929482753	0.270293739	0.195819996	0.49909972
0.441841244	0.852693994	0.398623868	0.335666392	0.504420915
0.47467106	0.775017074	0.448879464	0.422639643	0.44300657
0.414516088	0.451751094	0.301138694	0.465482876	0.273364916
0.377095019	0.398264833	0.306848833	0.437059015	0.489302989
0.495034164	0.598697852	0.405732282	0.260417806	0.450843929
0.560699621	0.707253835	0.306275103	0.484275526	0.304518124
0.489472598	1.020516848	0.363115394	0.362788342	0.411624283
0.449315271	0.713062283	0.343623355	0.608361179	0.404693056
0.586173641	0.645147077	0.304602566	0.298333936	0.421907898
0.398706768	0.537635385	0.319436262	0.702319793	0.227320547
0.495308746	0.827367389	0.379034218	0.422903381	0.394418739
0.351086883	0.897758928	0.296828186	0.468753056	0.287075079
0.428390977	0.524421958	0.268612808	0.577262735	0.321658117
0.443959507	0.908148418	0.39713461	0.334551449	0.386918067

O75015,FCGR3B,CARDIOMETABOLIC	Q9UEW3,MARCO,CARDIOMETABOLIC	Q16853,AOC3,CARDIOMETABOLIC	Q12860,CNTN1,CARDIOMETABOLIC
O75015	Q9UEW3	Q16853	Q12860
FCGR3B	MARCO	AOC3	CNTN1
CARDIOMETABOLIC	CARDIOMETABOLIC	CARDIOMETABOLIC	CARDIOMETABOLIC
0.278722234	0.623862501	0.870550563	0.499341944
0.350624813	0.748876644	0.440099	0.510895453
0.379954877	0.803405365	0.752519045	0.39052857
0.264639441	0.64478943	0.692506731	0.380271046
0.431041894	0.904191337	0.658839976	0.355199142
0.182883903	0.432568345	0.660714999	0.317251744
0.446459124	0.418587171	0.56923647	0.340234183
0.346229366	0.380112928	0.604661656	0.306806297
0.391911551	0.54532946	0.399647508	0.232097035
0.251599333	0.641401663	0.55064713	0.283653191
0.291223766	0.380798579	0.599196042	0.256014956
0.421527892	0.541675221	1.019668359	0.503268428
0.160940575	0.536258308	0.641223854	0.391205891
0.157399188	0.308148997	0.518745924	0.337649892
0.289693657	0.386730378	0.486125258	0.244109441
0.340989684	0.616468892	0.400174183	0.260093094
0.35379854	0.526133202	0.651167229	0.403740439
0.26879906	0.659205416	0.714942935	0.437059015
0.417978315	0.572243073	0.5069446	0.364982703
0.353455378	0.719267339	0.718519891	0.334760219
0.22032422	0.518494288	0.713705107	0.290457704
0.229092161	0.742210337	0.508246401	0.274523203
0.255926244	0.568172141	0.600152066	0.264786229
0.190359701	0.20044801	0.457803374	0.413368397
0.414918532	0.659525342	0.530748392	0.397961287
0.321568947	0.664250816	0.606046333	0.326940498
0.391070333	0.717972255	0.421937144	0.519321551
0.107053213	0.427797513	0.594933367	0.315694274
0.521811267	0.750019495	0.57990963	0.335852576
0.230973621	0.349314879	0.52679005	0.336062157
0.314558445	0.392945191	0.402678402	0.347648194
0.429015001	0.567975262	0.64166847	0.439489316
0.41847113	0.471849932	0.443467413	0.319259178
0.244939949	0.446273486	0.452785606	0.337884014
0.359359567	0.709364973	0.561282895	0.286796635

Q9BQR3,PRSS27,CARDIOMETABOLIC	P09601,HMOX1,CARDIOMETABOLIC	P08709,F7,CARDIOMETABOLIC	Q9NQX5,NPDC1,CARDIOMETABOLIC	P15086,CPB1,CARDIOMETABOLIC
Q9BQR3	P09601	P08709	Q9NQX5	P15086
PRSS27	HMOX1	F7	NPDC1	CPB1
CARDIOMETABOLIC	CARDIOMETABOLIC	CARDIOMETABOLIC	CARDIOMETABOLIC	CARDIOMETABOLIC
0.438850057	0.464097554	0.400757104	0.55290365	0.343052194
0.445717033	0.362738052	0.385606159	0.614975141	0.493116352
0.493253092	0.32371587	0.196010114	0.455682225	0.317361714
0.449720327	0.360482218	0.285586562	0.551105336	0.2967459
0.33743932	0.26235642	0.271401387	0.793938961	0.352818961
0.369949435	0.63270285	0.399038541	0.470315244	0.294247079
0.641001661	0.222657791	0.384991901	0.415897524	0.275915804
0.324952331	0.172455973	0.246421493	0.408101523	0.276221974
0.277027288	0.269060031	0.274675474	0.339151082	0.278432592
0.287353793	0.290115644	0.238357677	0.308512318	0.218378087
0.207631897	0.268315072	0.280544202	0.298520104	0.24348419
0.446397236	0.423490056	0.399342909	0.601234624	0.500832469
0.366707074	0.183340828	0.228632119	0.330380399	0.223787289
0.244787196	0.183023398	0.237730679	0.42065226	0.324727169
0.302729254	0.336691686	0.21041362	0.282299798	0.168579382
0.492945481	0.313926778	0.248910673	0.374049213	0.360832202
0.547526235	0.248514167	0.35611127	0.476021948	0.297281172
0.660028396	0.266591003	0.241517561	0.43669563	0.261285687
0.565265284	0.286637645	0.385713087	0.423167284	0.293168093
0.33662168	0.173210705	0.286578046	0.459106257	0.24686599
0.464838027	0.258063856	0.247465618	0.351135557	0.219912269
0.250676733	0.478304079	0.367495885	0.421849413	0.312623904
0.349072836	0.366859614	0.305766021	0.412252458	0.241752045
0.391287248	0.202388609	0.246883102	0.322461762	0.283103208
0.30513086	0.286538321	0.257153198	0.482499717	0.234279074
0.494485458	0.267794827	0.380165627	0.39452811	0.282495541
0.368133259	0.270706231	0.401229615	0.490117648	0.620068712
0.353332901	0.226173216	0.28138162	0.42513708	0.283574557
0.558527414	0.374308574	0.342102369	0.433528877	0.316900096
0.258977739	0.329009238	0.34075341	0.349435963	0.21709524
0.380613859	0.304539232	0.278490496	0.39359942	0.384938533
0.418587171	0.287353793	0.272381382	0.425962992	0.295821743
0.324547152	0.183137609	0.251477285	0.254140816	0.268017666
0.299868108	0.225953843	0.307914135	0.352647814	0.335387308
0.485653748	0.256210232	0.367444943	0.469826502	0.286915935

P23526,AHCY,CARDIOMETABOLIC	P19961,AMY2B,CARDIOMETABOLIC	P07204,THBD,CARDIOMETABOLIC	O95841,ANGPTL1,CARDIOMETABOLIC	P02462,COL4A1,CARDIOMETABOLIC
P23526	P19961	P07204	O95841	P02462
AHCY	AMY2B	THBD	ANGPTL1	COL4A1
CARDIOMETABOLIC	CARDIOMETABOLIC	CARDIOMETABOLIC	CARDIOMETABOLIC	CARDIOMETABOLIC
1.508484633	0.513238037	0.380745793	0.591151575	0.716034001
1.222470796	0.282299798	0.648240022	0.570065659	0.547222707
1.316675654	0.270312475	0.582528282	0.771640088	0.8630404
1.363864198	0.339456826	0.43915435	0.716878237	0.711679705
1.525518843	0.557483109	0.534588238	0.816882227	0.679007775
1.571018315	0.527886618	0.542765152	0.782411782	0.750851754
1.113807568	0.442086321	0.479698555	0.494862628	0.599528399
0.965467767	0.366021424	0.498892194	0.512136397	0.575026361
0.888411517	0.419749351	0.29392093	0.4200404	0.399619808
1.333483524	0.212804417	0.27939924	0.361708649	0.36243646
0.954687186	0.45546118	0.334690614	0.449066186	0.507788631
1.566342815	0.552712061	0.63529563	0.789931796	1.304774055
1.019739039	0.42699764	0.377068882	0.534254848	0.458057304
1.301883181	0.222149069	0.287413553	0.52949905	0.400451659
0.926395403	0.269788358	0.23361422	0.413511684	0.43178948
1.250842375	0.391558562	0.289954815	0.29982654	0.366681656
1.076463946	0.626940311	0.389717332	0.574866952	0.569986636
1.519819507	0.498201061	0.446737727	0.483906425	0.553287026
0.961994133	0.293534097	0.395952699	0.483839346	0.57271925
1.035982764	0.297693577	0.377278031	0.493116352	0.485721078
0.960395135	0.268501118	0.466419496	0.678725443	0.593203905
1.421781729	0.285744968	0.3304033	0.651573575	0.524676471
1.237990291	0.387481678	0.314514841	0.556787991	0.514627337
1.087337643	0.403264971	0.326963161	0.518530228	0.396447024
1.620119241	0.508316864	0.491751037	0.468753056	0.431550112
1.145994405	0.488252725	0.374957764	0.408214689	0.68216848
1.10182874	0.249342377	0.434732543	0.388126811	0.375946689
1.696427764	0.451031469	0.387857875	0.582649428	0.393408491
1.781632771	0.351403387	0.524312919	0.697710282	0.595098341
1.206225801	0.30515201	0.336155346	0.439885515	0.361357815
1.088846059	0.5	0.365261095	0.258870056	0.25566029
1.031754693	0.44614977	0.449221848	0.763394131	0.441871871
1.215121015	0.202094225	0.295473367	0.341604764	0.33645839
1.136423051	0.297838054	0.387589126	0.397299808	0.492945481
1.121166078	0.306211421	0.392373632	0.40522638	0.330380399

Q8IW75,SERPINA12,CARDIOMETABOLIC	P08263,GSTA1,CARDIOMETABOLIC	P12104,FABP2,CARDIOMETABOLIC	Q9NNX6,CD209,CARDIOMETABOLIC	P55808,XG,CARDIOMETABOLIC
Q8IW75	P08263	P12104	Q9NNX6	P55808
SERPINA12	GSTA1	FABP2	CD209	XG
CARDIOMETABOLIC	CARDIOMETABOLIC	CARDIOMETABOLIC	CARDIOMETABOLIC	CARDIOMETABOLIC
0.409319698	0.112765683	0.186093555	0.253314225	0.333486439
0.492740513	0.098679896	0.142171087	0.218287285	0.43915435
1.074376758	0.171561769	0.220232608	0.204022476	0.387079015
0.289051808	0.251756337	0.142279528	0.324997382	0.378430428
0.343218685	0.245449818	0.32005683	0.479532333	0.377042746
0.690445765	0.136843612	0.245807357	0.435094293	0.896018236
1.120389214	0.068379136	0.118782977	0.17506954	0.331918282
0.448350838	0.046539515	0.145017609	0.247843272	0.372470998
0.243940296	0.149612228	0.152904206	0.22357023	0.270499906
0.284677426	0.06037939	0.147286795	0.209671111	0.308533704
0.426790509	0.090496136	0.148753962	0.218923692	0.35645701
0.691835045	0.098625191	0.190109166	0.324502163	0.668685621
0.348830961	0.046607308	0.095961532	0.230190468	0.322662987
0.315628634	0.057803474	0.117880746	0.171217254	0.373013565
0.209105078	0.063932796	0.102287375	0.109378418	0.251233369
0.270762529	0.083974785	0.085679864	0.186830254	0.293086821
0.368822864	0.126648334	0.251041886	0.368873998	0.312363979
0.278529106	0.108412273	0.535144349	0.36366954	0.247208456
0.24363613	0.092090746	0.106291637	0.245943699	0.371130893
0.374983755	0.290880805	0.193311584	0.201576589	0.449876215
0.249307813	0.119717005	0.172097733	0.219090677	0.289874433
0.400174183	0.250312111	0.128300616	0.239865903	0.335712928
0.244041769	0.062305356	0.188038502	0.234913251	0.336878439
0.188181929	0.228442028	0.099448954	0.119154059	0.301472853
0.277200161	0.159099367	0.481864693	0.276796959	0.296314268
0.461275317	0.055973974	0.232161395	0.235027259	0.283436999
0.781165424	0.104125837	0.147368491	0.162735398	0.507577492
0.319192797	0.13093294	0.093066128	0.158088026	0.314645671
0.228442028	0.062699598	0.125199439	0.219501089	0.383420665
0.353063601	0.062808342	0.155825149	0.169059149	0.3028132
0.417167884	0.056289124	0.11105144	0.190122344	0.3747499
0.327734624	0.19026736	0.16127559	0.174089368	0.399509025
0.293107137	0.06586245	0.24018201	0.14860968	0.348903506
0.454767166	0.052534173	0.151280716	0.142269666	0.255394613
0.306763768	0.087686588	0.151354135	0.214819967	0.302603378

Q16769,QPCT,CARDIOMETABOLIC	Q96AP7,ESAM,CARDIOMETABOLIC	P15085,CPA1,CARDIOMETABOLIC	Q92692,NECTIN2,CARDIOMETABOLIC	P02452,COL1A1,CARDIOMETABOLIC
Q16769	Q96AP7	P15085	Q92692	P02452
QPCT	ESAM	CPA1	NECTIN2	COL1A1
CARDIOMETABOLIC	CARDIOMETABOLIC	CARDIOMETABOLIC	CARDIOMETABOLIC	CARDIOMETABOLIC
0.43149029	0.511037123	0.188038502	0.355149904	2.072252782
0.586824089	0.643673063	0.405451147	0.532295768	1.842672906
0.453822486	0.537039459	0.193244599	0.478436711	2.062937338
0.394500764	0.38667677	0.227241778	0.317713875	1.421880282
0.474276404	0.492330833	0.27325125	0.428390977	1.659559091
0.441045683	0.55015117	0.203020878	0.481530805	1.910433447
0.513629511	0.403824403	0.275667291	0.362662631	1.638981988
0.492877149	0.609331826	0.16681258	0.433949781	2.014748836
0.382014684	0.305575334	0.166396847	0.237944993	1.53815405
0.3509409	0.392727356	0.093053227	0.261412494	1.635577362
0.345079338	0.483035123	0.152597158	0.257706352	1.764916586
0.509798841	0.796253658	0.329032044	0.578945721	2.343232324
0.436332548	0.438637177	0.151396106	0.344076198	2.142507122
0.335526821	0.380191979	0.360457232	0.266350889	1.811895812
0.279089548	0.302288919	0.09111927	0.179430867	1.616081549
0.317273735	0.331826267	0.28210419	0.224284216	1.561140085
0.425078148	0.553670668	0.197100044	0.390420307	1.975201723
0.35769454	0.373375715	0.198278479	0.360807192	1.833880967
0.378509129	0.391694289	0.113597265	0.378430428	1.731233115
0.396364593	0.471751824	0.138436857	0.341297086	2.032703793
0.287294046	0.350187623	0.079676606	0.396007594	1.605363498
0.375477926	0.419982174	0.226816893	0.392863489	1.910168623
0.361257639	0.384885173	0.137442492	0.297404834	1.887138097
0.384805147	0.410285479	0.135465746	0.332655317	1.617762697
0.355371527	0.507507131	0.155598494	0.487610129	1.818816504
0.416041688	0.392319242	0.120968221	0.311585499	1.632971939
0.532701778	0.604955111	0.556903784	0.419632988	1.393004579
0.463808125	0.451531956	0.222256883	0.305681257	1.766997502
0.637191974	0.570658676	0.152893608	0.544385294	2.088113309
0.292376649	0.335712928	0.132972517	0.365058607	1.303779594
0.393353957	0.47384923	0.230685623	0.314427651	1.042032302
0.516915368	0.459647564	0.207229314	0.524022259	1.837698383
0.42806447	0.393845037	0.183023398	0.293696912	1.555199866
0.365767805	0.425372891	0.273611353	0.273099769	1.429489477
0.352745602	0.433528877	0.178426277	0.345869571	1.750904019

Q01973,ROR1,CARDIOMETABOLIC	P10451,SPP1,CARDIOMETABOLIC	Q8IZP9,ADGRG2,CARDIOMETABOLIC	Q16773,KYAT1,CARDIOMETABOLIC	P06858,LPL,CARDIOMETABOLIC
Q01973	P10451	Q8IZP9	Q16773	P06858
ROR1	SPP1	ADGRG2	KYAT1	LPL
CARDIOMETABOLIC	CARDIOMETABOLIC	CARDIOMETABOLIC	CARDIOMETABOLIC	CARDIOMETABOLIC
0.539464526	2.112571251	0.908526185	0.591643486	0.752049746
0.735450424	2.270484204	0.843581873	0.70110382	0.964865665
0.51128514	2.697644051	0.969491396	0.94809268	0.858089479
0.380719402	1.398131443	1.003750009	0.294267476	0.908526185
0.493253092	1.579425622	0.990548829	0.558295178	0.652794135
0.674411013	1.615297612	0.852398524	0.586458123	0.443590385
0.438333243	1.613842737	0.568250912	0.397382432	0.824847889
0.649904661	2.362476535	0.660074148	0.284243645	0.785454712
0.301953856	1.296120633	0.669706094	0.439824538	0.744632247
0.279360509	1.586557711	0.80770479	0.46448374	0.655832817
0.372548459	1.173973628	0.64166847	0.235924966	0.680940194
0.85340354	4.9588308	1.145359106	0.651483254	0.923766415
0.438029518	2.316585612	0.792015187	0.183302707	0.915670215
0.399121527	1.515506458	0.508140725	0.247054288	1.066585781
0.275552668	1.161508732	0.490661507	0.30369604	0.690015177
0.353406382	0.954621014	0.46054052	0.370257278	0.625898229
0.541412462	1.65783451	0.829377032	0.761333254	0.82393361
0.470608734	1.441629062	0.950988632	0.528399131	1.273236129
0.384032416	1.461854778	0.892361391	0.311218559	0.850333099
0.407338476	1.801127111	0.784529717	0.51505557	0.638341346
0.460604369	2.002496887	1.063706401	0.401897637	0.914401706
0.379954877	2.324144872	0.728247122	0.716778863	0.418761292
0.42566784	1.958297565	0.759067454	0.511214265	0.57125231
0.366275219	0.9568071	0.627635996	0.25518227	0.551258156
0.603991436	1.856647585	1.057604338	0.729965406	0.814789899
0.360182502	1.448741302	0.882335967	0.382730293	0.891990345
0.488455825	1.038139271	0.481263862	0.328895232	0.428955531
0.508316864	1.483083679	0.706714787	0.59184857	0.475857
0.468201026	2.506023598	0.729358491	0.418877414	0.631826345
0.341723176	1.311756556	0.665541254	0.523078726	0.69495911
0.298044571	0.69322712	0.380271046	0.245211747	0.430265777
0.514805724	2.153972752	0.820286619	0.389933497	0.624468195
0.351939659	1.558437178	0.485653748	0.332447861	0.526023807
0.315738042	1.195239722	0.584024177	0.269582733	0.586376829
0.378509129	1.802875784	0.719815962	0.695055458	0.803182644

O75340,PDCD6,CARDIOMETABOLIC	P61978,HNRNPK,CARDIOMETABOLIC	Q6PJW8,CNST,CARDIOMETABOLIC	Q9H7M9,VSIR,CARDIOMETABOLIC	P09496,CLTA,CARDIOMETABOLIC
O75340	P61978	Q6PJW8	Q9H7M9	P09496
PDCD6	HNRNPK	CNST	VSIR	CLTA
CARDIOMETABOLIC	CARDIOMETABOLIC	CARDIOMETABOLIC	CARDIOMETABOLIC	CARDIOMETABOLIC
0.602152161	1.221454396	0.587801115	0.564521331	0.861307322
0.651663908	1.185010629	0.910228081	0.563778357	0.785454712
0.784420965	1.049862215	0.925304428	0.539090727	1.000762753
0.613782748	0.892423247	0.835087919	0.550990748	0.836420311
0.727944315	1.094369555	0.651302649	0.637059487	0.650670928
0.345031504	0.844869253	1.017761836	0.763447047	1.159176297
0.22284307	0.778570723	0.574189956	0.540250347	1.053069023
0.540250347	0.946516788	0.665587387	0.502675751	0.940565291
0.533847654	0.982480038	0.878491359	0.388288262	1.383382393
0.233080465	0.962727896	0.866997657	0.549922416	0.68164855
0.411025555	0.991922974	0.699744429	0.413511684	0.636927028
0.905445681	1.098930387	0.931223906	0.763235404	0.985481026
0.595469698	1.160301715	0.799849238	0.558604848	0.670542184
0.455082495	1.201885949	0.538194667	0.362838638	0.919613787
0.317779948	1.24065329	0.645147077	0.391531422	0.922294884
0.457074107	1.378309613	0.784149153	0.497166158	0.646221206
0.805300983	1.127087874	0.889335697	0.54737445	0.679902604
0.556556477	1.048407806	0.720864496	0.614038066	1.278187957
0.60667678	1.031897734	0.642113395	0.587068193	0.956409258
0.52679005	1.177804426	0.851276671	0.539053362	0.911806754
0.595965202	1.037419937	0.797137221	0.506768937	1.019880415
0.542276291	0.964464472	0.870550563	0.500207987	0.820514082
0.498511952	0.665264521	0.460732093	0.465418351	0.774319028
0.346853897	1.020941357	0.655832817	0.406238816	0.887365273
0.672497123	0.817505306	0.97036539	0.52438561	0.67877249
0.294859585	0.790917982	0.87393628	0.543857276	0.991373087
0.49520576	1.265054904	0.754974593	0.435184778	0.861964286
0.206440795	1.515401415	0.74913623	0.513665114	0.676002272
0.639537118	0.803516748	0.676283472	0.694862775	0.636485697
0.254758114	1.046157451	0.939913567	0.590987696	1.048771219
0.448319762	0.912692006	0.735399449	0.477045903	0.726230789
0.463069294	0.755864742	0.710250575	0.655287538	0.736725968
0.377434969	0.879100493	0.771158864	0.441963766	0.903439564
0.407479674	1.29477373	0.92242275	0.465160339	0.884540435
0.520222246	1.282269935	0.918339815	0.321502085	1.166672795

P34947,GRK5,CARDIOMETABOLIC	P08670,VIM,CARDIOMETABOLIC	P34913,EPHX2,CARDIOMETABOLIC	P16860,NPPB,CARDIOMETABOLIC	P20160,AZU1,CARDIOMETABOLIC
P34947	P08670	P34913	P16860	P20160
GRK5	VIM	EPHX2	NPPB	AZU1
CARDIOMETABOLIC	CARDIOMETABOLIC	CARDIOMETABOLIC	CARDIOMETABOLIC	CARDIOMETABOLIC
0.574229757	0.921464186	0.930191718	0.272268125	0.362486708
0.668453912	0.542802774	0.623473437	0.147962142	0.284401307
0.621445595	0.442423523	0.636750459	0.27939924	0.421674008
1.046229968	0.857494902	0.686627717	0.264144633	0.313578815
0.820286619	1.510158524	0.920187651	0.662411672	0.334783423
0.922614582	0.392373632	0.678255149	0.252402832	0.209351622
0.991716731	0.546275263	0.730927389	0.772764112	0.218317548
0.875876884	1.774853566	0.712173175	0.422317519	0.243399819
0.722615447	0.968819629	0.766788178	0.558295178	0.487306037
0.637412846	0.746130558	0.713161141	0.48256661	0.2154612
0.743910002	0.804519893	0.695200006	0.502292626	1.067843333
0.699744429	0.967075211	0.838917008	0.590168981	0.392917955
0.725124186	0.720964436	0.619424349	0.46823348	0.231326107
0.701395461	1.504516574	0.889828986	0.440740079	0.241953213
0.747528247	0.963328665	0.964798788	0.335666392	0.253437164
1.12482456	0.426317446	0.621101088	0.315497395	0.43259833
0.829607016	0.772175133	0.751841262	0.443559639	0.46664586
0.801680896	1.873713013	1.260852926	0.249221424	0.182655867
0.650986712	1.582384281	0.941935384	0.575664439	0.271025406
0.800903317	1.592507316	0.520799511	0.299556491	0.300596476
0.70120102	0.994125571	0.581560021	0.4399465	0.360607173
0.783877434	1.21167266	0.59106963	0.323536414	0.227904292
0.617152957	0.541374935	0.737339014	0.386596371	0.234457771
0.643182474	0.911617168	0.789165614	0.515877346	0.244804164
0.768011596	1.478362431	0.401619159	0.7031966	0.232080948
0.830930662	0.303969821	0.65492427	0.616853585	0.281908718
0.859160755	0.717425037	0.808376899	0.23258017	0.267535084
1.193170323	1.004515622	0.501110266	0.400201922	0.276317722
0.886996305	3.091056447	0.577062705	0.197935186	0.266018779
0.936012694	1.537301353	1.025764821	0.566520474	0.177292081
0.716282202	1.77706937	0.774533744	0.205014805	0.180591263
0.795150581	0.925112036	1.179601858	1.282181057	0.204872749
0.624511481	0.687818583	0.763764622	0.81056512	0.251024486
0.706420933	0.883927531	0.587515981	0.429937841	0.253437164
0.799461243	0.820172911	0.902438173	0.949868694	0.427945802

Q9Y4X3,CCL27,CARDIOMETABOLIC	O95988,TCL1B,CARDIOMETABOLIC	P04275,VWF,CARDIOMETABOLIC	P40189,IL6ST,CARDIOMETABOLIC	P55058,PLTP,CARDIOMETABOLIC
Q9Y4X3	O95988	P04275	P40189	P55058
CCL27	TCL1B	VWF	IL6ST	PLTP
CARDIOMETABOLIC	CARDIOMETABOLIC	CARDIOMETABOLIC	CARDIOMETABOLIC	CARDIOMETABOLIC
0.01420458	1.416175438	0.632001549	0.486496051	0.507190631
0.095193046	1.47959261	0.283220971	0.431729626	0.365641062
0.109674497	1.246860445	0.451375494	0.593738677	0.427501089
0.037540717	2.365917881	0.56202258	0.393490306	0.467811749
0.197962627	1.277922193	1.077882561	0.574150158	0.385258849
0.202388609	1.72500435	0.428094142	0.551678629	0.571965487
0.115198992	2.144587233	0.419080704	0.379533727	0.35201285
0.126630778	0.772656991	0.30587201	0.349944976	0.267164458
0.040084825	1.368788933	0.236432456	0.416994425	0.339221614
0.113196401	1.957483301	0.415148675	0.442791674	0.29233612
0.053319239	1.130608932	0.183735206	0.338047996	0.357570594
0.117635875	1.633651214	0.537113914	0.573315028	0.715637057
0.133452663	2.189194292	0.322774833	0.332885976	0.466548833
0.195752142	0.85583228	0.298623581	0.321234779	0.34757591
0.070000062	1.511310402	0.225000482	0.36437604	0.35754581
0.041935039	1.407270827	0.256530096	0.325944898	0.249186877
0.160161578	1.121243794	0.318971626	0.46554741	0.286081875
0.106979035	1.932140491	0.354584161	0.468136124	0.391612847
0.127883319	1.291815468	0.240548549	0.497062786	0.508175948
0.082229508	0.941347957	0.283358424	0.446923558	0.409206226
0.091924931	1.301973424	0.245126778	0.389555287	0.239766167
0.188534441	1.0947489	0.261376257	0.424077545	0.261811432
0.093687475	1.138157331	0.153562728	0.339080565	0.30384343
0.06785505	1.727996146	0.189018585	0.315519265	0.297095776
0.124835486	1.171210181	0.28973382	0.580191072	0.375738278
0.071515511	1.849198375	0.133397173	0.457169163	0.46532158
0.117766409	1.015084621	0.220981883	0.455682225	0.408384496
0.074207047	1.436342694	0.356902027	0.385980535	0.318022336
0.072438476	3.944657386	0.442147611	0.503094039	0.43178948
0.169281943	1.365661562	0.176911532	0.421732468	0.352037251
0.260941805	0.882152509	0.196540699	0.353553391	0.319569139
0.091803948	1.555199866	0.337579687	0.520402573	0.359658598
0.096662492	1.163765191	0.151396106	0.353259435	0.357199013
0.213469225	1.362730239	0.212671704	0.408639339	0.276796959
0.154416626	1.398034536	0.210238676	0.371594229	0.401285241

Q9ULL4,PLXNB3,CARDIOMETABOLIC	Q16619,CTF1,CARDIOMETABOLIC	Q13158,FADD,CARDIOMETABOLIC	Q8NBP7,PCSK9,CARDIOMETABOLIC	Q8NHL6,LILRB1,CARDIOMETABOLIC
Q9ULL4	Q16619	Q13158	Q8NBP7	Q8NHL6
PLXNB3	CTF1	FADD	PCSK9	LILRB1
CARDIOMETABOLIC	CARDIOMETABOLIC	CARDIOMETABOLIC	CARDIOMETABOLIC	CARDIOMETABOLIC
0.531668906	1.406490687	0.621531752	0.325110037	0.413999233
0.594974606	0.710841591	0.802125565	0.262720376	0.36905302
0.569394317	0.714596127	0.751684937	0.284361883	0.448723921
0.501527252	0.749915527	0.770945083	0.259642778	0.422551766
0.527703698	0.822336061	0.938871747	0.331872272	0.551831608
0.553056968	0.611701619	0.866517025	0.279670502	0.423813074
0.498339211	0.705344516	0.855061444	0.318905305	0.344195466
0.414544821	0.70061802	0.581479405	0.235581801	0.305194316
0.420098634	0.676705491	0.646893445	0.324614646	0.319369844
0.444821985	0.826450318	0.709610863	0.251390145	0.351890873
0.39112455	0.543894974	0.675346593	0.377775226	0.318772703
0.684062477	0.744167866	0.771533123	0.271928638	0.557908331
0.445778827	0.495823996	0.76540753	0.255447726	0.318861098
0.391748593	0.70027816	0.789548612	0.207171866	0.231245949
0.376285604	0.573951207	0.645549666	0.226738298	0.222380162
0.381432585	0.774319028	1.01494391	0.219668514	0.325335464
0.47440792	0.633448835	1.030325368	0.2874534	0.410114882
0.552482242	1.155966829	0.906513242	0.285151396	0.296848762
0.51644979	0.640202404	0.744890362	0.337252256	0.372936007
0.48053053	1.252143578	0.946451183	0.238258568	0.339245128
0.605080921	0.575265557	0.7850737	0.206140517	0.231454417
0.371645746	72.57999237	0.652296594	0.262829661	0.338704721
0.342149798	1.159176297	0.470967692	0.219440238	0.401981218
0.38483182	0.580231289	0.54559412	0.273213372	0.252893174
0.588331016	0.590373553	0.946713631	0.171585554	0.354191133
0.558449991	0.41612821	0.671937988	0.388315177	0.303107194
0.54002571	0.638872524	0.783225695	0.292417184	0.348927691
0.563231528	0.644431982	0.92312633	0.254493374	0.341060598
0.580070437	0.973936758	0.762706553	0.391368623	0.326397067
0.404636958	0.659616778	0.914972317	0.339339199	0.294124731
0.495377415	1.478259963	0.782086453	0.341155174	0.452189689
0.56899978	0.508175948	0.675206174	0.249446097	0.344290911
0.391422882	0.403488651	0.635471796	0.23607219	0.328257527
0.429133966	0.662182138	0.7622309	0.20508587	0.306296333
0.48066378	0.814112457	1.014662547	0.276164542	0.423020651

Q15582,TGFBI,CARDIOMETABOLIC	P42574,CASP3,CARDIOMETABOLIC	P17931,LGALS3,CARDIOMETABOLIC	P20023,CR2,CARDIOMETABOLIC	Q07507,DPT,CARDIOMETABOLIC
Q15582	P42574	P17931	P20023	Q07507
TGFBI	CASP3	LGALS3	CR2	DPT
CARDIOMETABOLIC	CARDIOMETABOLIC	CARDIOMETABOLIC	CARDIOMETABOLIC	CARDIOMETABOLIC
0.368567304	0.688486371	0.398623868	0.862502175	0.428509769
0.321725011	0.636927028	0.423607489	0.94487802	0.398430501
0.268054823	0.910291175	0.241283305	1.525201653	0.355938526
0.302708271	0.809217823	0.378063375	0.697323497	0.337860594
0.319391982	1.174380567	0.352086057	1.115894007	0.317801976
0.333024449	0.786762445	0.157235621	0.496580667	0.357768928
0.328234775	0.684252165	0.314340485	0.840896415	0.300930033
0.356679449	0.508563561	0.291445898	0.712173175	0.338892591
0.386382056	0.78627179	0.321992725	0.577342766	0.30038819
0.230062859	0.698387673	0.206512355	0.620627703	0.204546393
0.263157789	0.542689914	0.26781339	0.71222254	0.293880186
0.345151103	1.058484395	0.564951921	0.886750412	0.688677286
0.248738201	0.65071603	0.246592359	0.641268301	0.371723036
0.19922906	0.775661984	0.33641175	0.559573676	0.234197893
0.173150685	0.623646324	0.263303755	0.49270636	0.156735078
0.269825761	0.751893378	0.296396435	0.46557968	0.222997586
0.305702446	1.256926248	0.440373634	1.162878203	0.253876718
0.272759245	0.790424735	0.430982143	1.230376625	0.314166226
0.32255118	0.721814485	0.36680876	0.816429377	0.41748608
0.289512993	0.813266448	0.296458075	1.030682513	0.344672954
0.242255277	0.679007775	0.293127454	0.467131293	0.255589416
0.251616773	0.87448164	0.31822079	0.319082192	0.413769727
0.303254298	0.618351898	0.314057363	0.479399397	0.303885554
0.285428243	0.60336378	0.319591291	0.598697852	0.229298687
0.30283419	0.689154806	0.34512718	0.806138707	0.317625798
0.342126083	0.953827313	0.402762146	0.859160755	0.242003531
0.360182502	0.738157203	0.393190399	0.960861234	0.317009944
0.25187852	1.158373096	0.401034984	0.937376154	0.275399911
0.386649968	0.78024548	0.421761701	0.555400353	0.35404386
0.30797817	0.605962323	0.360807192	0.705931449	0.275094652
0.293676555	0.573951207	0.302645331	0.932774336	0.263157789
0.352721152	0.56703119	0.353381887	1.06311672	0.357273298
0.237088896	0.720614706	0.28155721	0.413225159	0.226785452
0.229282794	0.648374834	0.230685623	1.129277464	0.223013044
0.353774017	1.067621304	0.414861016	0.864417389	0.273194435

O60635,TSPAN1,CARDIOMETABOLIC	O00533,CHL1,CARDIOMETABOLIC	P17676,CEBPB,CARDIOMETABOLIC	P04054,PLA2G1B,CARDIOMETABOLIC	Q13043,STK4,CARDIOMETABOLIC
O60635	O00533	P17676	P04054	Q13043
TSPAN1	CHL1	CEBPB	PLA2G1B	STK4
CARDIOMETABOLIC	CARDIOMETABOLIC	CARDIOMETABOLIC	CARDIOMETABOLIC	CARDIOMETABOLIC
0.84130452	0.748824737	0.84886086	0.365438363	0.92242275
0.771372704	0.48169772	0.768703957	0.395486403	1.068805991
0.860591204	0.48310209	0.591725511	0.378797838	1.052193469
0.924214738	0.582649428	0.913388164	0.402678402	1.395710764
0.76101669	0.87260461	0.868983085	0.495171436	1.219001583
0.842296455	0.795371074	0.684109894	0.428183171	1.650839656
0.618952241	0.394555457	0.71276579	0.431669779	0.984729921
0.853699358	0.471817227	0.636132852	0.334412342	1.027259021
0.775178252	0.465676505	1.08132485	0.266960833	0.876180493
0.642246933	0.386274943	0.862920765	0.187154287	1.320422841
0.636353357	0.610854208	0.708726058	0.414717261	1.092550526
0.78583591	0.614038066	0.698629758	0.530013128	1.069324705
0.691211919	0.460093825	0.525222272	0.323401887	1.765405993
0.660486052	0.360982299	0.756808396	0.213454429	1.16384586
0.698484497	0.338704721	0.805133543	0.224113273	0.871818667
0.754451466	0.368388518	0.700569459	0.332955205	1.220438841
0.849626106	0.524167569	0.864177755	0.378089581	1.138709703
0.81552443	0.469305736	0.508035071	0.340611724	0.842296455
0.669474031	0.550532638	0.729661885	0.228521213	0.861964286
0.727288667	0.460030047	0.597412778	0.314711107	1.265581135
0.739283695	0.556903784	0.944550607	0.18351883	1.372969903
0.687818583	0.398844973	0.786489822	0.275476279	1.155406087
0.776253622	0.377147299	0.74102802	0.237994477	0.993299025
0.98951947	0.35658057	0.937636086	0.254846421	1.024698867
0.591889595	0.597868456	0.488659011	0.329785529	1.441629062
0.973734255	0.544234379	0.60684501	0.314732922	0.895831934
1.012835586	0.540063143	0.96279463	0.434009943	1.161589244
0.772175133	0.494725442	0.847038823	0.464161896	1.097560145
0.730978055	0.46223551	0.599237577	0.414717261	1.127009753
0.848684362	0.408979377	1.052339344	0.234896969	1.363864198
0.671612041	0.345773689	0.885215121	0.456725739	1.115584659
0.611065951	0.494519734	0.808545014	0.325967492	0.937571096
0.6910682	0.308042219	0.952704032	0.344744634	0.954753362
0.676799309	0.477608362	0.74597542	0.370077672	1.051537281
0.967947027	0.41205248	1.053872254	0.348685917	0.844752137

Q9H1U4,MEGF9,CARDIOMETABOLIC	O14917,PCDH17,CARDIOMETABOLIC	O95998,IL18BP,CARDIOMETABOLIC	A6NI73,LILRA5,CARDIOMETABOLIC	P19021,PAM,CARDIOMETABOLIC
Q9H1U4	O14917	O95998	A6NI73	P19021
MEGF9	PCDH17	IL18BP	LILRA5	PAM
CARDIOMETABOLIC	CARDIOMETABOLIC	CARDIOMETABOLIC	CARDIOMETABOLIC	CARDIOMETABOLIC
0.482265663	0.448879464	0.409433201	0.450562766	0.39727227
0.434883236	0.863160051	0.606130355	0.484812903	0.508528311
0.455271798	0.723969086	0.563387711	0.404861398	0.444637027
0.413196517	0.615828269	0.623387011	0.402706315	0.372677597
0.468915541	0.727641635	0.488591273	0.455271798	0.467228441
0.353406382	0.742519078	0.521413557	0.327552939	0.451438073
0.403125235	0.592053724	0.491171923	0.262101951	0.343266268
0.308127638	0.48173111	0.538045468	0.338071429	0.372290317
0.415407739	0.367725212	0.445315582	0.359309753	0.277084901
0.302267967	0.519213572	0.382412079	0.378430428	0.25396472
0.38890778	0.356333493	0.403908385	0.241316756	0.305278946
0.570026146	0.580150857	0.549960535	0.369821242	0.561905723
0.385632888	0.380613859	0.341249775	0.306976474	0.373220465
0.330953401	0.404861398	0.346733708	0.171466661	0.311304859
0.253788746	0.324412204	0.347985718	0.231936214	0.194642681
0.346733708	0.422054146	0.373505139	0.305723636	0.272740339
0.428658305	0.467617232	0.426908857	0.571569168	0.398568611
0.39763041	0.412910211	0.411339066	0.304813774	0.381247558
0.433889627	0.402148432	0.388638303	0.537411836	0.351988451
0.419400359	0.456915725	0.498719321	0.347335072	0.334829837
0.284263348	0.496993883	0.44807123	0.29889279	0.340022
0.327462135	0.540475078	0.460444763	0.323334644	0.311196988
0.285725163	0.445531703	0.365818515	0.344314776	0.346109393
0.368414053	0.380693014	0.398596238	0.19144475	0.287154684
0.36912977	0.68339898	0.375816418	0.345773689	0.382200084
0.386569575	0.450000965	0.439306576	0.317713875	0.313426702
0.460189509	0.521016151	0.585199321	0.409461582	0.393817738
0.441076255	0.35137903	0.340824275	0.393244911	0.341960122
0.42581539	0.692794797	0.537598121	0.537113914	0.435064136
0.340588116	0.346589535	0.373712312	0.392945191	0.29233612
0.398596238	0.532369564	0.370976576	0.28631993	0.310442934
0.378351744	0.616597096	0.479532333	0.271853254	0.378194425
0.308319918	0.276816146	0.37397144	0.244787196	0.295903774
0.324614646	0.494862628	0.465805636	0.374568115	0.327416742
0.335015558	0.378037171	0.422668939	0.330861654	0.335062004

Q14393,GAS6,CARDIOMETABOLIC	P41218,MNDA,CARDIOMETABOLIC	P01130,LDLR,CARDIOMETABOLIC	P08581,MET,CARDIOMETABOLIC	Q9NR28,DIABLO,CARDIOMETABOLIC
Q14393	P41218	P01130	P08581	Q9NR28
GAS6	MNDA	LDLR	MET	DIABLO
CARDIOMETABOLIC	CARDIOMETABOLIC	CARDIOMETABOLIC	CARDIOMETABOLIC	CARDIOMETABOLIC
0.43587912	0.71976607	0.207819077	0.486833381	0.946123225
0.560660758	0.573156093	0.296540282	0.570421395	1.340340919
0.55351718	0.714398026	0.124818181	0.50902203	0.633844124
0.499307333	0.550608963	0.144265676	0.442975864	1.047245729
0.513914406	0.718221129	0.451375494	0.709512497	1.106880902
0.473455258	0.513451531	0.161052169	0.549579463	1.243408201
0.422141918	0.470054518	0.1147527	0.494348377	0.601359661
0.494862628	0.52445831	0.13553149	0.42199564	0.803628147
0.436211588	0.641268301	0.152269616	0.470771863	0.677315539
0.325764206	0.738617833	0.111244044	0.33401852	0.657562531
0.3747499	0.528582292	0.151396106	0.487441165	0.726633608
0.601317979	0.645818198	0.220217344	0.453728127	0.719965659
0.498235595	0.461115479	0.0848348	0.444544577	0.593245024
0.394829037	0.467941472	0.182693853	0.365641062	0.518135019
0.213188275	0.429282718	0.116483701	0.34006914	1.030253954
0.259139348	0.927230546	0.108142086	0.448257616	0.909219165
0.448164413	0.620025734	0.174451753	0.626288807	0.667805555
0.448381917	0.480097723	0.209293585	0.523622864	0.727540769
0.424106941	0.581116772	0.218423502	0.445624359	0.496821668
0.372006568	0.536853368	0.212303491	0.467746901	0.590537262
0.356481719	0.521377417	0.098714101	0.345605959	0.991991731
0.471817227	0.611277767	0.150684194	0.405901056	0.859220309
0.440495749	0.546805629	0.182150139	0.422756839	0.93322703
0.298292581	0.540962315	0.137309181	0.356654727	0.400868233
0.407253782	0.59139748	0.118085195	0.457676461	1.023563069
0.372961858	0.593820992	0.107745536	0.524312919	0.790041312
0.558256481	0.575664439	0.218529507	0.696260942	0.786980612
0.403684472	1.07937786	0.203189816	0.514948478	0.688295508
0.550990748	0.889520648	0.194885681	0.602694999	0.709709243
0.319170673	0.537300096	0.110766997	0.367037659	0.723216752
0.437817037	0.461467196	0.170305862	0.522028327	0.575106082
0.472242569	0.452283729	0.18351883	0.440007494	0.59139748
0.377775226	0.407564415	0.170684032	0.381961729	0.277989057
0.314994818	0.484342666	0.095788748	0.398623868	0.99212926
0.488557408	1.006327574	0.169634322	0.566599015	0.730319674

Q9Y286,SIGLEC7,CARDIOMETABOLIC	P09525,ANXA4,CARDIOMETABOLIC	Q05315,CLC,CARDIOMETABOLIC	P15090,FABP4,CARDIOMETABOLIC	P42785,PRCP,CARDIOMETABOLIC
Q9Y286	P09525	Q05315	P15090	P42785
SIGLEC7	ANXA4	CLC	FABP4	PRCP
CARDIOMETABOLIC	CARDIOMETABOLIC	CARDIOMETABOLIC	CARDIOMETABOLIC	CARDIOMETABOLIC
0.378010968	1.019456347	0.350017752	0.502257811	0.575664439
0.50768305	1.348728536	0.480131002	0.35623471	0.567424363
0.462011286	1.232083472	0.582447532	0.288271477	0.487880592
0.330357499	1.194991205	0.569315388	0.465999399	0.510046257
0.55198463	1.357827303	0.5030243	0.955415376	0.513914406
0.464773591	1.288595981	0.389744346	0.380824975	0.389717332
0.34820287	1.173729532	0.332747562	0.220415869	0.358687656
0.314100904	0.935882944	0.244041769	0.1701053	0.422317519
0.332540048	0.976302412	0.337884014	0.291062322	0.383500403
0.324344752	1.005909142	0.393817738	0.169728413	0.357347599
0.283358424	0.957868822	0.344935854	0.628942486	0.403600537
0.471849932	1.262514537	0.416301309	0.900501135	0.620971947
0.332932127	1.16384586	0.500624222	0.164550236	0.258672753
0.203965917	0.841246207	0.453791031	0.256210232	0.41339705
0.250450952	0.919422578	0.26960142	0.21452237	0.323805636
0.433168428	1.141238245	0.784801661	0.298851357	0.348637582
0.44807123	0.876605721	0.425579334	0.26681284	0.342102369
0.381168288	1.11211039	0.459201736	0.610854208	0.438910899
0.363467935	1.200803427	0.493697758	0.659433918	0.388934738
0.445315582	1.274737331	0.63423966	0.298189218	0.481464055
0.271025406	1.391460544	0.473258394	0.292396916	0.393135895
0.453130969	1.019385686	0.738720234	1.040733005	0.407649175
0.338751678	0.916178111	0.657243557	0.496236583	0.326578111
0.259211207	0.890445982	0.372755101	0.225562634	0.300804906
0.433739278	1.161669762	0.726230789	0.288031799	0.444267343
0.347865136	1.315125063	0.35370046	0.313535347	0.421557111
0.452722841	1.136344283	0.300471487	0.270443663	0.41085465
0.326804556	0.842763653	0.343909292	0.254352292	0.61796627
0.547260638	1.238505264	0.355839852	0.220232608	0.558488702
0.316504957	1.226459845	0.346853897	0.67694006	0.382385574
0.339127575	1.030396787	0.38646241	0.162104944	0.388261349
0.435094293	1.148061558	0.323155399	0.235271749	0.529352262
0.38979838	0.848037519	0.292417184	0.230669634	0.372625936
0.406605039	1.12482456	0.364805655	0.23324208	0.378115789
0.394856406	1.153965426	0.428688018	0.183480672	0.46885054

Q76M96,CCDC80,CARDIOMETABOLIC	Q99549,MPHOSPH8,CARDIOMETABOLIC	P23284,PPIB,CARDIOMETABOLIC	P35247,SFTPD,CARDIOMETABOLIC	P25445,FAS,CARDIOMETABOLIC
Q76M96	Q99549	P23284	P35247	P25445
CCDC80	MPHOSPH8	PPIB	SFTPD	FAS
CARDIOMETABOLIC	CARDIOMETABOLIC	CARDIOMETABOLIC	CARDIOMETABOLIC	CARDIOMETABOLIC
0.327575644	1.046810282	0.694621995	0.360157537	0.213750544
0.534106742	0.753771943	0.676658587	0.504665721	0.366707074
0.53968893	1.146312186	0.715686663	0.814056029	0.241233137
0.332194479	0.981595132	0.709758438	0.190967627	0.249532563
0.310356873	1.224845685	0.543970379	0.600651466	0.24400794
0.43569788	1.310211757	0.498201061	0.350090544	0.380561099
0.493116352	0.913134954	0.681459583	0.286300084	0.354313907
0.530049867	0.663606536	0.596874697	0.512775771	0.366402182
0.374827836	0.944092419	0.563934691	0.583659958	0.220141035
0.382120616	1.027543877	0.527850029	0.368720619	0.188756731
0.298934228	1.146391645	0.51652139	0.317713875	0.243737477
0.704709223	1.136108011	0.880198001	0.421148228	0.422200444
0.386355275	1.203136224	0.651257506	0.307019033	0.234311554
0.328507907	0.937506111	0.522173084	0.204901152	0.187608877
0.203372991	0.982752477	0.596378437	0.173751821	0.134679297
0.280524756	1.291994564	0.654515834	0.231261979	0.215849851
0.3075302	0.894528901	1.008562158	0.285744968	0.231229921
0.303717091	1.477952599	0.573633029	0.248359184	0.196826994
0.364249778	0.708922586	0.504490847	0.257135374	0.200573095
0.372238711	0.729156297	0.664527128	0.361007321	0.240082142
0.451031469	0.909471289	0.641623995	0.371413974	0.169399321
0.335852576	0.733820951	0.666418336	0.541975672	4.170441149
0.385018587	0.625681347	0.673430048	0.478105199	0.219881785
0.365387706	1.335703691	0.632308273	0.222041308	0.233646608
0.315388071	0.684441907	0.776845711	1.00583942	0.278915497
0.39112455	1.16199189	0.526133202	1.087789948	0.229409971
0.40410441	1.040011874	0.712864607	0.349847964	0.338305843
0.347913364	0.886996305	0.782900028	0.437059015	0.276068847
0.46664586	0.868802404	0.667574152	0.210107563	0.298851357
0.428301905	0.844869253	0.462139401	0.429997447	0.219805593
0.234360283	1.067991378	0.65094159	0.11528686	0.312061006
0.517058707	0.814394656	0.561399623	1.120855268	0.289252233
0.43178948	1.125916626	0.546616154	0.304982845	0.243872671
0.341581087	1.466726625	0.669891801	0.637899034	0.213572826
0.258870056	1.142821434	0.526169672	0.311196988	0.287294046

Q06418,TYRO3,CARDIOMETABOLIC	NTproBNP,NTproBNP,CARDIOMETABOLIC	P09237,MMP7,CARDIOMETABOLIC	O96017,CHEK2,CARDIOMETABOLIC
Q06418	NTproBNP	P09237	O96017
TYRO3	NTproBNP	MMP7	CHEK2
CARDIOMETABOLIC	CARDIOMETABOLIC	CARDIOMETABOLIC	CARDIOMETABOLIC
0.682215765	0.256707971	0.059730019	0.979148782
0.676095992	0.294349075	0.0889908	0.923894485
0.675440223	0.424871948	0.084447586	1.4037636
0.400063246	0.239583424	0.083014088	1.031325687
0.560155779	0.443959507	0.080475866	1.094597146
0.541337411	0.566795418	0.04674967	0.78747171
0.448475165	1.061128961	0.080164094	0.679949733
0.418645203	0.386649968	0.079439481	0.849920615
0.380007553	0.279166939	0.051331919	1.058557766
0.304391505	0.41085465	0.060862609	1.186325577
0.46823348	0.437756347	0.034068919	0.856960136
0.662090346	0.597951344	0.069170467	1.026049263
0.428420672	0.721214346	0.064515964	1.240137424
0.450625231	0.29816855	0.067409699	1.150929928
0.312905734	0.189714256	0.063153215	0.764559137
0.464129724	0.141022767	0.042685799	1.135084735
0.578624774	0.348420158	0.078487135	0.999584198
0.442760983	0.379060491	0.069579205	1.366703224
0.444914493	0.421878655	0.084394922	0.867779253
0.379744243	0.173547201	0.058707963	0.996056856
0.350794978	0.249255976	0.044842173	1.04181564
0.423842452	0.286359625	0.081412831	0.780299565
0.318595987	0.15430963	0.067181136	1.253793712
0.431131536	0.21745669	0.058253963	1.087036211
0.452597337	0.821595395	0.063280289	0.804966138
0.418326124	0.749811574	0.052073746	0.865316609
0.56616717	0.247654373	0.06609569	1.240309356
0.463711689	0.494177078	0.051821694	0.940043876
0.530196849	0.278258951	0.077966598	0.80068129
0.555901045	0.231518599	0.094286825	1.558221148
0.442147611	0.221918216	0.093616069	1.151408685
0.433588981	0.999168569	0.057631445	1.121010662
0.286756879	0.620971947	0.056222835	0.970567193
0.42513708	0.588575747	0.04793755	1.176906737
0.350770664	0.388530564	0.090759974	0.885828918

P36952,SERPINB5,CARDIOMETABOLIC	P51693,APLP1,CARDIOMETABOLIC	O95502,NPTXR,CARDIOMETABOLIC	P80370,DLK1,CARDIOMETABOLIC	Q9UHL4,DPP7,CARDIOMETABOLIC
P36952	P51693	O95502	P80370	Q9UHL4
SERPINB5	APLP1	NPTXR	DLK1	DPP7
CARDIOMETABOLIC	CARDIOMETABOLIC	CARDIOMETABOLIC	CARDIOMETABOLIC	CARDIOMETABOLIC
0.836478289	1.376591016	1.000901497	0.638916809	0.505611086
0.796916239	1.353035789	1.458211516	0.503128912	0.663192685
0.846510577	1.676322815	0.746078842	0.373039421	0.581801935
0.944092419	1.926390256	1.16295881	0.636000586	0.503687209
0.794104073	2.679195764	1.901449996	0.481397315	0.55186986
0.976167077	0.931611272	0.582245706	0.428212852	0.372832621
1.455384146	0.808432934	0.644208678	0.447760758	0.309926925
1.00556058	1.189207115	0.687151444	0.458660954	0.39088063
0.776953413	1.111879158	0.532258873	0.348396008	0.40502981
0.383287804	0.676846222	0.508105505	0.211921226	0.454074209
1.403082655	0.78046184	0.802793032	0.394118124	0.35060051
0.794324276	0.976843939	0.768703957	0.264749525	0.596998826
0.785890382	1.160945301	0.703245344	0.440556818	0.412624102
0.694285045	0.989793861	0.785999337	0.396309649	0.496787232
0.857494902	0.863519104	0.645415442	0.217803645	0.240698657
0.952373907	0.764665135	0.732956762	0.209453225	0.271401387
0.688772764	0.430027253	0.971644183	0.432388483	0.55144924
1.029326019	1.833626755	0.933679945	0.440556818	0.415407739
1.003680436	1.284582903	0.861128236	0.553363733	0.423372656
1.072144975	0.723617899	0.526863083	0.445408193	0.363997387
0.785128119	1.207062183	0.550532638	0.214329152	0.397162138
0.830642733	1.106957628	0.867839405	0.392564059	0.482499717
0.969625805	1.037060457	0.826049419	0.378797838	0.459902517
0.946976152	0.734533401	0.364123561	0.150820036	0.284914312
0.449284128	1.197810762	0.65879431	0.601609811	0.617324092
0.834393602	1.11984573	0.829779546	0.343885455	0.414659773
1.064812934	1.226799939	0.997991892	0.41838412	0.487914411
0.910543597	0.645191796	0.58394322	0.41116803	0.542727531
0.630819866	0.841712822	0.574110362	0.209366134	0.834335768
0.834509281	1.552722482	0.922230957	0.369513762	0.425992519
0.910480485	0.7944344	0.710250575	0.410997066	0.329237369
0.926459618	1.001664938	0.653382627	0.488828396	0.458311376
0.767266675	0.541037313	0.443067988	0.458470242	0.392482436
1.125136471	0.908085472	0.698290863	0.360307353	0.394911148
0.878856789	0.939262294	0.67952569	0.385980535	0.339550956

Q16270,IGFBP7,CARDIOMETABOLIC	Q03154,ACY1,CARDIOMETABOLIC	Q15067,ACOX1,CARDIOMETABOLIC	P54760,EPHB4,CARDIOMETABOLIC	P25815,S100P,CARDIOMETABOLIC
Q16270	Q03154	Q15067	P54760	P25815
IGFBP7	ACY1	ACOX1	EPHB4	S100P
CARDIOMETABOLIC	CARDIOMETABOLIC	CARDIOMETABOLIC	CARDIOMETABOLIC	CARDIOMETABOLIC
0.34193642	0.265760758	0.856663188	0.51015233	0.642514091
0.357124743	0.284401307	1.353598617	0.700472346	0.8560696
0.415666965	0.314079133	1.228672143	0.613612595	0.622135183
0.418413121	0.376285604	1.122332384	0.437968799	0.713161141
0.344458002	0.281420631	1.266722053	0.509728173	1.466523307
0.36180895	0.367827181	0.680326881	0.589964479	0.699889951
0.425490846	0.241266581	0.72286593	0.478105199	0.816203046
0.383979182	0.199173829	0.803516748	0.567306382	0.484745698
0.259426903	0.275743733	0.569039221	0.362059823	0.684536797
0.310830504	0.192749631	1.311120242	0.385552706	0.664711399
0.385472541	0.212465426	1.008282564	0.475461358	0.743291492
0.407394949	0.286856278	0.783008568	0.87569477	1.75625216
0.295514331	0.19225593	1.252230373	0.525222272	0.885215121
0.310356873	0.134530016	0.674738318	0.402427277	0.515770084
0.276662689	0.246284886	0.830124713	0.298437348	0.568802614
0.291486303	0.316680513	0.976437765	0.314471243	0.692746778
0.435184778	0.220935936	0.696502289	0.451939011	0.636662193
0.320367566	0.201926197	0.862083788	0.3842987	0.70730286
0.395102807	0.257849294	0.725727578	0.476153948	1.129590609
0.328781266	0.275075585	1.017197626	0.544196657	0.77840884
0.226942702	0.264712825	0.741593241	0.437877735	1.048771219
0.284421021	0.388530564	0.917194749	0.498477399	0.615572207
0.269938002	0.231213894	0.897447842	0.401646998	0.741541839
0.295493848	0.198622367	0.618351898	0.461275317	0.910291175
0.316987971	0.300055234	0.912755271	0.57327529	0.549312871
0.345342549	0.334945901	0.584510157	0.490423494	0.626723068
0.412538308	0.229823782	0.762389418	0.620326645	0.698581334
0.298644281	0.295289098	0.713606173	0.503163787	1.085831318
0.380429229	0.125060665	1.282447707	0.621790293	0.88154126
0.216044439	0.22510968	1.006257823	0.393708564	0.563348661
0.33008283	0.118003373	0.815750573	0.481397315	0.480397317
0.336201951	0.376207366	1.181402033	0.57990963	0.518278697
0.257688489	0.253525014	0.783551497	0.414200155	0.362034728
0.258726548	0.23725329	0.705540106	0.452158346	0.465515142
0.336855089	0.304813774	0.925047915	0.449252987	0.480497224

Q9BWV1,BOC,CARDIOMETABOLIC	P14555,PLA2G2A,CARDIOMETABOLIC	Q9Y275,TNFSF13B,CARDIOMETABOLIC	P00797,REN,CARDIOMETABOLIC	P08319,ADH4,CARDIOMETABOLIC
Q9BWV1	P14555	Q9Y275	P00797	P08319
BOC	PLA2G2A	TNFSF13B	REN	ADH4
CARDIOMETABOLIC	CARDIOMETABOLIC	CARDIOMETABOLIC	CARDIOMETABOLIC	CARDIOMETABOLIC
0.733312481	1.394646993	0.590373553	0.372238711	0.110882224
0.672403901	0.782140665	0.606214388	0.447202451	0.133619271
0.742570548	0.724722203	0.562802249	0.631388549	0.194265282
0.474901428	6.877098456	0.669520437	0.772549886	0.252647884
0.675206174	0.936402051	0.675533865	0.736470682	0.313491884
0.815694031	1.521084183	0.529425651	0.434280777	0.151008326
0.571727662	0.689728268	0.465708784	0.456440907	0.154224086
0.51359391	0.56923647	0.546957257	0.339174591	0.122105492
0.441106829	1.106574052	0.469435874	0.303359417	0.19840221
0.418645203	0.824162085	0.485552769	0.497993908	0.089939583
0.44590244	0.774802224	0.502048971	0.241768803	0.109887562
0.989931086	1.531876509	0.604703569	0.30384343	0.139728673
0.524676471	0.735450424	0.625811467	0.242860539	0.08459405
0.506839195	0.667666703	0.386944887	0.248273124	0.087080892
0.327053827	0.603238327	0.325222731	0.307210621	0.187154287
0.359907981	0.74298243	0.3323557	0.50547092	0.389852421
0.57271925	0.676752398	0.687627906	0.355100673	0.186455079
0.614336071	1.31777129	0.564286602	0.338916082	0.399315229
0.523296313	0.897323438	0.513095757	0.395760628	0.137166492
0.505365821	0.969760233	0.552865327	0.341841628	0.14203319
0.536592948	1.068805991	0.393572139	0.320811997	0.145259055
0.327484834	5.235763747	0.699792933	0.400035517	0.266905326
0.511426918	1.335888872	0.503303313	0.311240132	0.115254901
0.591233532	1.055114816	0.35201285	0.266129436	0.227809529
0.49830467	0.812252396	0.561983625	0.420623103	0.208815397
0.47207893	1.275532802	0.468493196	0.278799523	0.081046854
0.557676352	0.548627939	0.58422662	0.27431397	0.093227539
0.667527881	0.957736042	0.49393736	0.307423637	0.158504972
0.528362507	0.757228177	0.582649428	0.339362721	0.116742356
0.405535467	0.567149113	0.409915941	0.569512732	0.132733092
0.39801646	1.355570363	0.413827091	0.349363308	0.067878571
0.709266641	0.471849932	0.577743088	0.438576374	0.316066492
0.520041982	0.608276848	0.398679133	0.253226448	0.108412273
0.385659619	0.475296604	0.413196517	0.282280231	0.092423274
0.441963766	0.703928108	0.478934411	0.364300278	0.11220431

Q8NHS0,DNAJB8,CARDIOMETABOLIC	P05556,ITGB1,CARDIOMETABOLIC	P02786,TFRC,CARDIOMETABOLIC	P62736,ACTA2,CARDIOMETABOLIC	P55259,GP2,CARDIOMETABOLIC
Q8NHS0	P05556	P02786	P62736	P55259
DNAJB8	ITGB1	TFRC	ACTA2	GP2
CARDIOMETABOLIC	CARDIOMETABOLIC	CARDIOMETABOLIC	CARDIOMETABOLIC	CARDIOMETABOLIC
0.890384263	0.605416542	0.837812898	0.542802774	0.31837523
0.806641759	0.576702827	0.900501135	0.478237777	0.320611927
0.792509426	0.579266846	0.771479646	0.453382308	0.449564493
0.668129655	0.595387154	0.964999433	0.386837618	0.369283319
0.775608221	0.625291149	0.859696895	1.5888688	0.548361808
0.8560696	0.605416542	0.691499446	0.419516657	0.508493064
0.77981294	0.477045903	0.94881584	0.539913427	0.53972634
0.848155091	0.510966283	0.757700707	0.530527706	0.181733967
1.761250358	0.533034198	0.825191005	0.434070114	0.270856384
0.770998522	0.38799232	0.560427634	0.563817436	0.488625141
0.976167077	0.535998177	0.814225325	0.461947242	0.247894815
0.885031065	0.636618064	1.058190961	1.405029089	0.336575017
0.860829844	0.447295454	0.788837477	0.469338267	0.471294255
0.86160588	0.39253685	0.713606173	0.540063143	0.159995141
1.03900313	0.386783994	0.631476084	0.383792919	0.23023834
0.798685817	0.335433806	0.782249101	0.490253556	0.286002567
0.818639395	0.588086387	0.707008762	1.057824284	0.344625175
0.769236967	0.636265146	0.663836564	1.787818152	0.314057363
0.882213658	0.5829322	0.789658074	0.690589354	0.336481712
0.759014841	0.561516375	0.68491649	0.464129724	0.191670472
0.938286231	0.580673862	0.658657332	0.638164384	0.324164947
0.903376945	0.447884921	1.046012433	0.601026289	0.193378593
0.659845423	0.551678629	0.69260274	0.499272725	0.484074163
0.841829517	0.370257278	0.754137764	0.681034599	0.241099406
0.831622098	0.494142826	0.807368945	0.387481678	0.413540348
0.726130119	0.571489937	0.773997065	0.440037994	0.219333791
0.976302412	0.480863724	0.752414731	0.615316251	0.203119408
1.043767221	0.42670177	0.759541132	0.506101972	0.193767699
4.182309997	0.493355672	0.721514353	0.456599125	0.199394843
1.083575741	0.568605516	0.857732683	0.717574237	0.282417227
0.72597914	0.343052194	0.796971478	0.397189668	0.339668656
0.618737766	0.586539429	0.799849238	0.431969094	0.199546932
0.954422527	0.391395751	0.784910465	0.425372891	0.397079559
0.908463213	0.403936382	0.725526392	0.477178187	0.508986748
0.742004582	0.503687209	0.878856789	0.386141093	0.3139703

Q14162,SCARF1,CARDIOMETABOLIC	Q8TDL5,BPIFB1,CARDIOMETABOLIC	P48357,LEPR,CARDIOMETABOLIC	Q03167,TGFBR3,CARDIOMETABOLIC	P39060,COL18A1,CARDIOMETABOLIC
Q14162	Q8TDL5	P48357	Q03167	P39060
SCARF1	BPIFB1	LEPR	TGFBR3	COL18A1
CARDIOMETABOLIC	CARDIOMETABOLIC	CARDIOMETABOLIC	CARDIOMETABOLIC	CARDIOMETABOLIC
0.602903914	0.626592758	0.655832817	0.318331096	0.288251496
0.720564759	0.354313907	0.539053362	0.359160351	0.3900957
0.532517189	0.413999233	0.664573191	0.381353276	0.283672853
0.442025039	1.057897609	0.770891647	0.377644322	0.297260567
0.577582926	0.221733706	0.778948579	0.303527681	0.301222199
0.401786223	0.576063598	0.615145672	0.42443043	0.243433564
0.480597151	0.405338748	0.486968379	0.331688293	0.292396916
0.540737382	0.728196645	0.41847113	0.354707072	0.308683441
0.322037366	0.660074148	0.544158938	0.297528546	0.227430871
0.327734624	0.235402248	0.623387011	0.236498018	0.202276412
0.378010968	0.255713459	0.443559639	0.274390036	0.244600626
0.96306161	0.786871521	0.517273791	0.578424273	0.45231508
0.394883776	0.362009635	0.517094548	0.275094652	0.199311934
0.391287248	0.320611927	0.4063233	0.279554214	0.239616639
0.326600748	0.367063101	0.461339268	0.159673854	0.164561642
0.346926031	0.572798651	0.495892737	0.21209757	0.275075585
0.795481343	0.877821798	0.588738957	0.291647983	0.247654373
0.49103576	0.839149637	0.623905745	0.282515123	0.22047699
0.437362066	0.396199784	0.736317553	0.290256444	0.215595654
0.468980551	0.269395937	0.496030247	0.269507999	0.314144451
0.314340485	0.299348926	0.618523365	0.268594189	0.218347815
0.441902501	0.421498675	0.520582962	0.248496941	0.272098329
0.450562766	0.477509056	0.442270218	0.280116719	0.247037164
0.325245274	0.425490846	0.496030247	0.260598376	0.225765978
0.433348615	0.342600699	0.641046093	0.279748054	0.242709082
0.561477455	0.87575547	0.63745703	0.352061653	0.333139886
0.579708683	0.425549836	0.540175458	0.346517471	0.333001366
0.526680518	0.782086453	0.450843929	0.285863831	0.292315858
0.453005352	0.657562531	0.633975942	0.39433673	0.332770627
0.345725758	0.196159621	0.471228924	0.308490935	0.278838176
0.424989764	0.362486708	0.525404332	0.322015045	0.320145581
0.505856469	0.277526992	0.493355672	0.384272063	0.24686599
0.434973676	0.467682062	0.327031158	0.254352292	0.227446636
0.365565037	0.276375187	0.652206173	0.243214307	0.221518639
0.566481207	1.133983776	0.495102795	0.281674331	0.281225632

Q76LX8,ADAMTS13,CARDIOMETABOLIC	Q9UKL0,RCOR1,CARDIOMETABOLIC	P31949,S100A11,CARDIOMETABOLIC	P51161,FABP6,CARDIOMETABOLIC
Q76LX8	Q9UKL0	P31949	P51161
ADAMTS13	RCOR1	S100A11	FABP6
CARDIOMETABOLIC	CARDIOMETABOLIC	CARDIOMETABOLIC	CARDIOMETABOLIC
0.255908505	0.947173091	0.414861016	0.699356515
0.268259283	1.05270412	0.482332524	0.659205416
0.30369604	1.227055071	0.512775771	0.657198002
0.320923202	0.968819629	0.50923377	0.580070437
0.291223766	0.824562068	0.749707635	0.880381052
0.292843139	0.889582307	0.589555689	0.665356752
0.273763118	0.935688353	0.436332548	0.679054842
0.249532563	1.205640679	0.359334659	0.734686159
0.190359701	0.886135976	0.38969032	0.616169852
0.20656962	1.127244132	0.536741744	0.693707795
0.255553986	1.011362366	0.401702682	0.596295767
0.314514841	1.006467091	0.692650749	0.699501958
0.257777813	0.980575078	0.394254739	0.570896056
0.245602985	0.854705908	0.297569796	0.622609718
0.230413955	0.950066234	0.283142457	0.414889773
0.247568557	0.968819629	0.482901215	0.556363623
0.35507606	1.252838107	0.38947429	0.688009314
0.193445624	1.09179349	0.53562678	0.683588485
0.291243953	3.466548632	0.512029913	0.639182582
0.278008327	1.463882745	0.437301439	0.77598464
0.266757363	0.993299025	0.662273942	0.537039459
0.217140389	0.92562517	0.551831608	1.374969865
0.228046511	0.777492142	0.415551734	0.570658676
0.258905945	0.87175824	0.376781491	0.813097351
0.300804906	0.903251719	0.407536166	0.384085658
0.267702033	0.939848419	0.44288376	0.691595315
0.315169537	0.992541961	0.412252458	0.557908331
0.234035615	1.215626474	0.447822835	0.633361027
0.294145119	0.913578117	0.465934802	0.797689946
0.288371401	0.991029563	0.457644739	0.883253827
0.265650254	0.625507895	0.349314879	0.899315974
0.283220971	0.733058379	0.412138173	0.582649428
0.21358763	1.106113937	0.280991813	0.658292196
0.209947425	0.959530121	0.387965427	0.496580667
0.293391708	0.836014575	0.443528895	0.647027977

Q9NZK5,ADA2,CARDIOMETABOLIC	Q86VZ4,LRP11,CARDIOMETABOLIC	P36222,CHI3L1,CARDIOMETABOLIC	P13598,ICAM2,CARDIOMETABOLIC	O75791,GRAP2,CARDIOMETABOLIC
Q9NZK5	Q86VZ4	P36222	P13598	O75791
ADA2	LRP11	CHI3L1	ICAM2	GRAP2
CARDIOMETABOLIC	CARDIOMETABOLIC	CARDIOMETABOLIC	CARDIOMETABOLIC	CARDIOMETABOLIC
0.344625175	0.328348552	0.429610155	0.817448643	0.832025702
0.465837924	0.498961359	0.378692827	0.771426173	0.919741281
0.626723068	0.325493356	0.284914312	0.788892157	0.943961549
0.635163538	0.291931137	0.29371727	0.419225971	0.721564367
0.608108222	0.373246335	0.374023287	0.853640186	0.922294884
0.403656492	0.398209626	0.46047668	0.496752798	1.139736247
0.755864742	0.374308574	0.33118288	0.711482413	0.564364834
0.535998177	0.325561047	0.465741066	0.515305537	0.734380675
0.55091437	0.241266581	0.381882311	0.589555689	0.883008971
0.601776636	0.237401343	0.335062004	0.437938442	0.825877665
0.449346416	0.289071844	0.484007061	0.619682014	0.685296394
0.40598547	0.471555668	0.569591689	1.0856808	0.852634892
0.358662794	0.407875286	0.249169606	0.524603741	0.848155091
0.431370672	0.280933388	0.362662631	0.395568651	0.623559874
0.561321802	0.170211451	0.253086069	0.521955964	0.812308699
0.511710592	0.236301387	0.311693505	0.59526336	1.027472656
0.574707587	0.314296911	0.342126083	0.628506687	1.187888973
0.516485589	0.318971626	0.389609295	0.584267117	0.772871247
0.596833326	0.284440736	0.45936091	0.856544437	0.778246992
0.565304466	0.25458159	0.348130471	0.716828548	0.663744543
0.486158955	0.286697256	0.297487303	0.315038489	0.68724671
0.430564118	0.292863438	0.364653968	0.455145588	1.114657127
0.457137476	0.247843272	0.327916409	0.593903319	0.591807547
0.341841628	0.287015389	0.328416838	0.5483238	0.769450274
0.368592852	0.386060806	0.360082652	0.44723345	0.969155454
0.479465861	0.256014956	0.239234938	0.777114992	0.795426207
0.396611936	0.341155174	0.4557454	0.676189725	0.804854553
0.158164749	0.400562703	0.320856474	0.546313129	0.897012503
0.52949905	0.489778042	0.460093825	0.697371833	0.90331433
0.330311705	0.324187417	0.341604764	0.523441422	0.721164357
0.367725212	0.254123201	0.29816855	0.739129981	0.797192477
0.46532158	0.388880824	0.292457725	0.667157827	1.067251359
0.48326955	0.272286998	0.394364064	0.443774907	0.810284248
0.394364064	0.252858118	0.3405173	0.552635444	0.917767103
0.540437616	0.295780736	0.341439057	0.634635443	0.87436042

P22004,BMP6,CARDIOMETABOLIC	Q9UHD0,IL19,CARDIOMETABOLIC	Q15165,PON2,CARDIOMETABOLIC	Q9BYF1,ACE2,CARDIOMETABOLIC	P35590,TIE1,CARDIOMETABOLIC
P22004	Q9UHD0	Q15165	Q9BYF1	P35590
BMP6	IL19	PON2	ACE2	TIE1
CARDIOMETABOLIC	CARDIOMETABOLIC	CARDIOMETABOLIC	CARDIOMETABOLIC	CARDIOMETABOLIC
0.541712768	0.399093863	0.774050716	0.323469143	0.46992421
0.56413017	0.427412202	0.897572263	0.374308574	0.433348615
0.584510157	0.429312475	0.683635869	0.296334807	0.52453102
0.519537575	0.561944673	1.190196681	0.344052349	0.42401876
0.460668226	0.521341279	1.121476974	0.306636215	0.731484906
0.457962064	0.372032355	0.762970933	0.329785529	0.474901428
0.513273613	0.597040208	0.780137323	0.287632779	0.411766966
0.513166892	1.781385801	0.838277609	0.256387885	0.388692183
0.507190631	0.333972218	0.631738762	0.291324714	0.314514841
0.43696814	0.45948829	0.784801661	0.252035699	0.353896647
0.454483556	0.273649286	0.485317235	0.299182978	0.345797657
0.745768621	0.73417709	0.72733908	0.379375916	0.512171897
0.651302649	0.359907981	0.880869374	0.328553451	0.302708271
0.378089581	0.258744482	0.571965487	0.30065899	0.348130471
0.30891889	0.180904475	0.582084296	0.262920766	0.242574533
0.295248165	0.361332768	0.411738426	0.328621779	0.296150002
1.187806638	0.876544962	0.902438173	0.36296441	0.386569575
0.736011391	0.479332943	0.77975889	0.280330379	0.408526055
0.453979797	0.532627934	0.694140687	0.28155721	0.323177799
0.67156549	0.390203872	0.75116409	0.308704838	0.355889186
0.352427889	0.622782366	0.567778451	0.256299044	0.308234446
0.318286969	0.470478271	0.58931055	0.307253213	0.299494206
0.354264793	0.288431373	0.762812294	0.264914736	0.301117821
0.546843532	0.374101071	0.847097537	0.366199062	0.264236194
0.457232545	0.318154625	0.588738957	0.317801976	0.154288239
0.42637655	0.626158587	0.464354976	0.381776445	0.380402861
0.679996865	0.385205445	0.829664522	0.244295636	0.394118124
2.792195582	0.349290667	0.609331826	0.327598351	0.136616155
0.557908331	0.512775771	0.701492702	0.254705144	0.439794053
0.429818653	0.235549145	0.506488002	0.247929183	0.302729254
0.47168643	0.623257395	0.473520897	0.28839139	0.393708564
0.346853897	0.362210431	0.665771953	0.260779072	0.397713103
0.447574578	0.325448236	0.646714113	0.271947487	0.338329293
0.396749415	1.866324693	0.556363623	0.275419001	0.354731659
0.631782552	0.558643569	0.740155344	0.278664282	0.375113737

P04080,CSTB,CARDIOMETABOLIC	Q9BUD6,SPON2,CARDIOMETABOLIC	Q8WX77,IGFBPL1,CARDIOMETABOLIC	Q99988,GDF15,CARDIOMETABOLIC	Q13867,BLMH,CARDIOMETABOLIC
P04080	Q9BUD6	Q8WX77	Q99988	Q13867
CSTB	SPON2	IGFBPL1	GDF15	BLMH
CARDIOMETABOLIC	CARDIOMETABOLIC	CARDIOMETABOLIC	CARDIOMETABOLIC	CARDIOMETABOLIC
0.609500792	0.242456864	0.46082791	0.250225374	0.725626978
0.349097033	0.30384343	0.529535753	0.290497973	0.643851552
0.440221039	0.305236628	0.637236142	0.224751087	0.581680965
0.548665969	0.251703992	0.489167344	0.307935478	0.452534598
0.696936926	0.23266079	0.553555548	0.375009747	0.431071773
0.713457798	0.280991813	0.597329964	0.247431314	0.509445598
0.467228441	0.219592396	0.42581539	0.250520401	0.312385631
0.470543498	0.2946757	0.538082764	0.217758358	0.376755375
0.431819411	0.197510328	0.375972748	0.188221065	0.691787092
0.422581056	0.189582802	0.445655248	0.215252217	0.416099367
0.523586571	0.162882103	0.615913647	0.217592389	0.544989372
1.002012148	0.376885971	0.691883001	0.342102369	0.846275907
0.335457057	0.207401754	0.516127712	0.202346528	0.441167983
0.402064816	0.171764048	0.436665362	0.17276705	0.389366319
0.349532861	0.097436087	0.459679425	0.189241447	0.301535549
0.510081612	0.169399321	0.392210483	0.192950141	0.380719402
0.547146851	0.160840207	0.465708784	0.234262835	0.63248361
0.5538242	0.207028315	0.40983071	0.226502676	0.49267221
0.579869435	0.205726565	0.466839972	0.239168617	0.534032704
0.537374586	0.183302707	0.5465025	0.222303104	0.614932516
0.454105684	0.272400263	0.570263263	0.231534647	0.641001661
0.59423274	0.213484022	0.497821347	0.223973508	0.564482202
0.484476972	0.151921715	0.403628514	0.189714256	0.429639934
0.373893683	0.142151379	0.478072061	0.254087974	0.406238816
0.417138969	0.232564049	0.43419048	0.195711441	0.619639062
0.579748867	0.292619943	0.480164284	0.246489825	0.574866952
0.293107137	0.218529507	0.450531536	0.267034861	0.500554825
0.693900158	0.20601196	0.359085674	0.209801951	0.546237399
0.441229146	0.310787417	0.535033081	0.225094077	0.646355598
0.453193791	0.252000762	0.461019602	0.248049508	0.318198734
0.418703244	0.200211951	0.308191718	0.203866976	0.275571768
0.344959764	0.257402862	0.439002177	0.226958433	0.523513991
0.437574327	0.178946471	0.347046287	0.182466055	0.449564493
0.3739196	0.136549885	0.342957093	0.204929559	0.27308084
0.622350836	0.177673448	0.395541233	0.216719369	0.437574327

Q14956,GPNMB,CARDIOMETABOLIC	P42830,CXCL5,CARDIOMETABOLIC	O15467,CCL16,CARDIOMETABOLIC	P05451,REG1A,CARDIOMETABOLIC	O95544,NADK,CARDIOMETABOLIC
Q14956	P42830	O15467	P05451	O95544
GPNMB	CXCL5	CCL16	REG1A	NADK
CARDIOMETABOLIC	CARDIOMETABOLIC	CARDIOMETABOLIC	CARDIOMETABOLIC	CARDIOMETABOLIC
0.559922865	0.195575831	0.260742923	0.307700779	0.440221039
0.557405831	0.156691628	0.191166284	0.290638957	0.29876851
0.545064928	0.223523745	0.26212012	0.39052857	0.499653546
0.458374915	0.155210708	0.235777834	0.358513661	0.431370672
0.51192345	0.222056699	0.287075079	0.332309628	1.149973009
0.653926321	0.218438642	0.278027598	0.470021938	0.469305736
0.439032608	0.243248026	0.23768125	0.407338476	0.221887454
0.490015741	0.098755164	0.290216208	0.388422856	0.210384453
0.439397937	0.182782518	0.140262378	0.274237924	0.232967401
0.529866198	0.45546118	0.213055323	0.335829297	0.393081399
0.358936365	0.186765515	0.207934348	0.33877516	0.417717647
0.644744739	0.247757391	0.409773899	0.330151476	0.367801686
0.582286066	0.598117154	0.158570906	0.217622556	0.349629785
0.413769727	0.269788358	0.164847053	0.168357512	0.193499266
0.306763768	0.569789128	0.070394182	0.16520165	0.215520947
0.290336931	0.214805077	0.21108559	0.26781339	0.68382544
0.414171446	2.253863274	0.180716482	0.429848447	0.38958229
0.510399917	0.258565196	0.198801425	0.269881876	0.242843706
0.397189668	0.123595661	0.304856033	0.256619018	0.481530805
0.424048151	0.198250993	0.338212058	0.309690708	0.315935071
0.568250912	0.200364663	0.150339915	0.377251881	0.380139277
0.393272169	0.235336989	0.21614929	0.40000779	0.69260274
0.435275282	0.206154806	0.23306431	0.453225205	0.456377635
0.346349381	0.087352936	0.255376911	0.303401474	0.231936214
0.517991382	0.157901852	0.100293499	0.316044585	0.282769809
0.540212901	0.883927531	0.349532861	0.312039376	0.354264793
0.414832261	0.153509516	0.150569347	0.315213231	0.282378079
0.26533741	0.582286066	0.314166226	0.276107121	0.415407739
0.321992725	0.342648197	0.349508634	0.430862666	0.414631032
0.465741066	0.11322779	0.165488171	0.411111034	0.285903463
0.323805636	0.215685336	0.318507666	0.285428243	0.257956553
0.539539317	0.143448024	0.285487602	0.299058577	0.277642437
0.321101209	0.174681654	0.122537905	0.743343015	0.349532861
0.462780506	0.172360369	0.091810311	0.290679251	0.306976474
0.330724081	0.444482955	0.34077703	0.289954815	0.583498155

Q13332,PTPRS,CARDIOMETABOLIC	Q9BXJ1,C1QTNF1,CARDIOMETABOLIC	Q06141,REG3A,CARDIOMETABOLIC	P19971,TYMP,CARDIOMETABOLIC	P80188,LCN2,CARDIOMETABOLIC
Q13332	Q9BXJ1	Q06141	P19971	P80188
PTPRS	C1QTNF1	REG3A	TYMP	LCN2
CARDIOMETABOLIC	CARDIOMETABOLIC	CARDIOMETABOLIC	CARDIOMETABOLIC	CARDIOMETABOLIC
0.429639934	0.524167569	0.331228794	0.353161505	0.218393224
0.681932099	0.468071231	0.221518639	0.421381827	0.485182695
0.615444215	0.538381223	0.270106451	0.488896167	0.440831738
0.407592667	0.454704126	0.288371401	0.389528286	0.319613444
0.536927797	0.487846776	0.43669563	0.307146745	0.458947171
0.604284566	0.53894128	0.284736629	0.264126324	0.449502174
0.590455402	0.460700159	0.349847964	0.261394375	0.458692747
0.630601278	0.442025039	0.388207528	0.247740218	0.493560895
0.436635096	0.344744634	0.261720711	0.318706423	0.550380019
0.334621025	0.414200155	0.269414611	0.534662352	0.374075141
0.417283563	0.391450014	0.262047455	0.307871452	0.438576374
0.665771953	0.54329211	0.345773689	0.737543476	0.291284331
0.580553127	0.348058087	0.209583929	0.355741206	0.295842249
0.427056838	0.343313858	0.146127539	0.204532215	0.295678244
0.257117551	0.326306583	0.169940309	0.238870401	0.258977739
0.427649275	0.302981162	0.368516214	0.412338192	0.444113398
0.476682313	0.579507806	0.210457379	0.354313907	0.414918532
0.471621044	0.459838766	0.248221502	0.409262958	0.311693505
0.412281034	0.451907686	0.219820829	0.381432585	0.383606747
0.558527414	0.309390328	0.317978252	0.27524724	0.462684284
0.385499261	0.470902406	0.215565768	0.368541758	0.400673778
0.416041688	0.491308124	0.251826149	0.456219494	0.332263564
0.416416748	0.405310653	0.317427715	0.310701261	0.35963367
0.414631032	0.332609205	0.25107669	0.206054803	0.351062548
0.501770653	0.350211897	0.257956553	0.30268729	0.347961598
0.413339745	0.540287796	0.299598021	0.256814755	0.446613882
0.645236519	0.341249775	0.260779072	0.256085949	0.512349433
0.479532333	0.326238737	0.282926654	0.284657694	0.45891536
0.483872884	0.627635996	0.380640242	0.364275027	0.502815141
0.300346551	0.356951508	0.198072432	0.235810522	0.467552411
0.369744348	0.340493698	0.237351982	0.243146883	0.406633224
0.603071097	0.484510555	0.289232184	0.387159514	0.380719402
0.450000965	0.384698471	0.323424304	0.25368322	0.279573592
0.519213572	0.370822323	0.22575033	0.252805543	0.354117489
0.464870248	0.443375206	0.407394949	0.328872436	0.52898547

Q9NPY3,CD93,CARDIOMETABOLIC	Q9BQB4,SOST,CARDIOMETABOLIC	P00915,CA1,CARDIOMETABOLIC	Q53H82,LACTB2,CARDIOMETABOLIC	Q12805,EFEMP1,CARDIOMETABOLIC
Q9NPY3	Q9BQB4	P00915	Q53H82	Q12805
CD93	SOST	CA1	LACTB2	EFEMP1
CARDIOMETABOLIC	CARDIOMETABOLIC	CARDIOMETABOLIC	CARDIOMETABOLIC	CARDIOMETABOLIC
0.793058942	0.478735269	0.865256632	0.889952351	0.258959789
1.132098902	0.637103647	0.853936087	0.888657871	0.268929514
0.959330613	0.573036921	1.061643949	1.054091423	0.300117635
0.547791961	0.350649117	0.83052759	0.812252396	0.304307121
0.807648806	0.668175968	0.750071484	0.862024035	0.255961725
0.814056029	0.589596555	0.885460589	1.071550617	0.292153808
0.609036248	0.475692109	1.195074038	0.824619224	0.22084407
0.826679491	0.407140882	0.558875951	0.722365051	0.24556894
0.47976506	0.408158102	0.687103816	0.629465843	0.234149198
0.417457143	0.39801646	1.287524602	0.879953992	0.197004433
0.778462797	0.349363308	0.643360827	0.722264917	0.211891849
1.429291322	0.87230224	1.534214286	0.927873476	0.372780939
0.700715153	0.346325375	0.43696814	0.914401706	0.226832615
0.634283624	0.355987873	0.905131932	0.670635147	0.189084106
0.352818961	0.311477531	0.614975141	0.679007775	0.132613541
0.343980813	0.328485137	1.358674624	0.923446317	0.144816711
0.736828107	0.319746395	2.924520293	1.243925428	0.307253213
0.685058929	0.616041736	0.87448164	0.891557654	0.314645671
0.726482525	0.524785586	0.601359661	0.769983801	0.293229062
0.689537061	0.491853304	0.763288309	0.908967111	0.205455805
0.879283316	0.521305144	0.333417099	1.022215945	0.231021656
0.653337339	0.414372452	1.09497657	0.878004355	0.255129212
0.616127143	0.599279114	0.487339815	0.682925448	0.194251817
0.460604369	0.463005103	0.72401927	0.672077728	0.171051185
0.769290288	0.495755265	0.654016981	0.933097667	0.270499906
0.725425819	0.508563561	1.657604701	0.940891323	0.265650254
0.59238212	0.360882227	1.275090812	0.946451183	0.272004043
0.743755326	0.295760235	1.43415405	0.767639045	0.203274338
0.646490018	0.477045903	1.676439012	1.058851301	0.289412673
0.554900112	0.398044049	0.602235643	0.660486052	0.247774565
0.36296441	0.356037227	1.079527504	0.81966142	0.171942727
0.876605721	0.389123496	0.826564896	0.70120102	0.248410834
0.471130945	0.318861098	1.009121578	0.615999037	0.168977141
0.703391594	0.28155721	0.755183946	0.903815372	0.235532818
0.599445293	0.609543041	2.686075767	0.793938961	0.252035699

Q13508,ART3,CARDIOMETABOLIC	Q06033,ITIH3,CARDIOMETABOLIC	P01222,TSHB,CARDIOMETABOLIC	P09467,FBP1,CARDIOMETABOLIC	P13501,CCL5,CARDIOMETABOLIC
Q13508	Q06033	P01222	P09467	P13501
ART3	ITIH3	TSHB	FBP1	CCL5
CARDIOMETABOLIC	CARDIOMETABOLIC	CARDIOMETABOLIC	CARDIOMETABOLIC	CARDIOMETABOLIC
0.31837523	0.481630947	0.440068496	0.189845802	0.417746602
0.244922972	0.284953812	0.521630452	0.305173163	0.404636958
0.414315012	0.503128912	0.5813988	0.229696376	0.443037278
0.344267047	0.531042783	0.544083507	0.55144924	0.28751318
0.356531141	0.375425877	0.585564501	0.604871252	0.364527611
0.352452318	0.261249467	0.365109218	0.465128098	0.306699985
0.331803268	0.309519026	0.611108308	0.319325573	0.757543164
0.373453364	0.302939163	0.274694513	0.616212563	0.250920109
0.225828582	0.451125268	0.326103086	0.618995145	0.650851357
0.23266079	0.381459025	0.128987217	0.231021656	1.051464397
0.298809931	0.262847879	0.641312752	0.537151145	0.906576079
0.395733197	0.419632988	0.791521257	0.327439438	0.673850287
0.283476294	0.338352745	0.388046111	0.569315388	0.490253556
0.310550544	0.265889738	0.221349804	0.322774833	0.252770499
0.190214614	0.273744143	0.23988253	0.325448236	0.987190215
0.191179535	0.486192654	0.36432553	0.500450749	1.02023394
0.398955572	0.516628809	0.334551449	0.409660302	7.553786458
0.255678012	0.482098551	0.852103156	0.711482413	0.238043972
0.300971753	0.465644228	0.514912786	0.195223685	0.419022611
0.305787216	0.301995718	0.818809644	0.487846776	0.202234354
0.2769121	0.338235502	0.442423523	0.393272169	0.254228909
0.229553129	0.349726737	0.567935894	0.352159279	1.25989194
0.246080117	0.30891889	0.354215684	0.193257994	0.394118124
0.267999089	0.210165825	0.311326438	0.382597672	0.174596918
0.283005108	0.398044049	0.448723921	0.292072817	0.164687162
0.302855182	0.378876615	0.413827091	0.493971599	1.030611074
0.311650299	0.309433221	0.623862501	0.241166262	0.261340025
0.230445899	0.303506643	0.273573425	0.463101392	1.966322554
0.297404834	0.328439603	0.393326692	0.28029152	1.639436474
0.305914416	0.374568115	0.609627547	0.260923719	0.206455105
0.508035071	0.274104896	0.78154454	0.29763168	1.364053283
0.286776756	0.316614668	0.422698237	0.216824547	0.614208337
0.279496089	0.329123284	0.308426792	0.095636159	0.605164809
0.333370881	0.265834453	0.667435348	0.257046273	0.475098975
0.309068815	0.446613882	0.459775023	0.315650513	0.735450424

Q9UKP3,ITGB1BP2,CARDIOMETABOLIC	P03950,ANG,CARDIOMETABOLIC	P18827,SDC1,CARDIOMETABOLIC	P07711,CTSL,CARDIOMETABOLIC	Q92558,WASF1,CARDIOMETABOLIC
Q9UKP3	P03950	P18827	P07711	Q92558
ITGB1BP2	ANG	SDC1	CTSL	WASF1
CARDIOMETABOLIC	CARDIOMETABOLIC	CARDIOMETABOLIC	CARDIOMETABOLIC	CARDIOMETABOLIC
3.190096354	0.358463964	0.426140182	0.355125288	0.901687858
0.88871947	0.350211897	0.490967693	0.341178822	0.775554462
1.115739322	0.344983675	0.497062786	0.398071641	0.870550563
0.60996569	0.332309628	0.317515736	0.379165604	1.064370182
1.083801088	0.242087417	0.378141999	0.286062046	0.939197192
0.698048895	0.31273227	0.506312497	0.350527612	0.626288807
1.204554776	0.270875159	0.336995212	0.251738888	0.891372279
0.858743989	0.325899716	0.44217826	0.246746239	0.909156145
0.398900269	0.176189518	0.365565037	0.276068847	0.78008325
0.636044672	0.186674918	0.342862019	0.241116118	1.00765376
0.489269074	0.189057895	0.349654021	0.220736942	0.842822071
0.78132788	0.494999852	0.488625141	0.543894974	0.660577621
1.234220361	0.266627963	0.431639859	0.30333839	0.852398524
0.624122012	0.231598851	0.292012089	0.235843215	1.059952783
0.577102706	0.178748123	0.243805064	0.190425686	0.956541853
0.664665326	0.289914621	0.313231237	0.332470905	0.626462475
0.884969721	0.18306146	0.389771362	0.284776104	0.647701055
1.51887169	0.235222831	0.500554825	0.312884045	0.665264521
0.700812299	0.248927927	0.358836861	0.206727182	0.972048363
0.427945802	0.380745793	0.315475528	0.312342328	0.748824737
0.608234687	0.226094844	0.349484409	0.219455449	1.40133317
0.813379198	0.386944887	0.329968452	0.350989554	0.593368399
0.548742035	0.237483634	0.286458887	0.278896165	1.065477406
0.832544908	0.223771778	0.236350529	0.173330807	0.866036659
0.927037754	0.271363766	0.68155406	0.233824823	0.875937598
1.004445996	0.346181372	0.559573676	0.358687656	0.880381052
1.035910957	0.342054947	0.386810805	0.236366912	1.171047828
1.08192463	0.376833727	0.403404757	0.299473448	0.736266517
1.036270039	0.326397067	0.706029319	0.291082498	0.590537262
0.887365273	0.291163214	0.485182695	0.259013644	1.120932962
0.327280602	0.279942028	0.315256932	0.280252666	0.94927632
0.371028008	0.3459415	0.455871777	0.246883102	0.782249101
0.756651039	0.284736629	0.340611724	0.215222378	0.954422527
0.575385193	0.346541491	0.370616752	0.19527782	1.008003047
0.63971446	0.193257994	0.470869767	0.234815574	3.005697874

Q92820,GGH,CARDIOMETABOLIC	Q92520,FAM3C,CARDIOMETABOLIC	P08118,MSMB,CARDIOMETABOLIC	P32942,ICAM3,CARDIOMETABOLIC	Q15485,FCN2,CARDIOMETABOLIC
Q92820	Q92520	P08118	P32942	Q15485
GGH	FAM3C	MSMB	ICAM3	FCN2
CARDIOMETABOLIC	CARDIOMETABOLIC	CARDIOMETABOLIC	CARDIOMETABOLIC	CARDIOMETABOLIC
0.462171435	0.311175418	0.269713567	0.487677731	0.63630925
0.710940141	0.495617832	0.358265245	0.477409771	0.694670145
0.750331484	0.483236053	0.333417099	0.607981782	0.640246781
0.489913856	0.277104107	0.365337056	0.346950079	0.738669032
0.600651466	0.492535631	0.398402885	0.594644774	0.579507806
0.320767526	0.635075491	0.569591689	0.397354889	0.545934585
0.386274943	0.267053371	0.831276308	0.530013128	0.352476749
0.487204715	0.286399325	0.845103533	0.317956212	0.364022619
0.405198293	0.220813457	0.239899158	0.432478405	0.522390295
0.293513751	0.249117798	0.391558562	0.359160351	0.585645683
0.425933468	0.312623904	0.396859432	0.332401777	0.560816227
0.581640648	0.549084465	0.911617168	0.519933854	0.717077025
0.392482436	0.23346853	0.466354841	0.318507666	0.56373928
0.383500403	0.212907695	0.64743174	0.297466684	0.224470848
0.244922972	0.145460566	0.439976996	0.315716157	0.382332567
0.328872436	0.205441565	0.52268005	0.40287383	0.577342766
0.584996541	0.323895426	0.483437067	0.448288688	0.511249701
0.532960309	0.355765865	0.478702087	0.480264141	0.561477455
0.41402793	0.289192091	0.410797697	0.559418551	0.77292482
0.452722841	0.304201675	0.717822973	0.392346436	0.219379405
0.387830992	0.263943309	0.522136891	0.30155645	0.424106941
0.613485011	0.333602036	0.379770566	0.25201823	0.811520812
0.354264793	0.412767132	0.247499926	0.283181711	0.377749042
0.340493698	0.239517006	0.632790568	0.306253874	0.407056229
0.349654021	0.414889773	0.546881438	0.484007061	0.438880477
0.381908782	0.311261706	0.477343593	0.480131002	0.831852705
0.717126731	0.351768938	0.375764323	0.484846509	0.62135945
0.675393406	0.254722799	0.528728867	0.482399394	0.875209316
0.563153453	0.298437348	0.94710744	0.463037197	0.592546385
0.366199062	0.252998371	0.253244001	0.245466832	0.74571693
0.323133	0.280194395	1.298098626	0.400479417	0.911806754
0.535886731	0.34276697	0.806753591	0.395020656	0.66296288
0.379849545	0.185359762	0.464934697	0.337556288	0.398651499
0.402427277	0.268073404	0.69140359	0.374646012	0.422903381
0.479399397	0.341723176	0.583336398	0.4399465	0.281362117

P10646,TFPI,CARDIOMETABOLIC	Q13541,EIF4EBP1,CARDIOMETABOLIC	P49747,COMP,CARDIOMETABOLIC	P10145,CXCL8,CARDIOMETABOLIC	P58546,MTPN,CARDIOMETABOLIC
P10646	Q13541	P49747	P10145	P58546
TFPI	EIF4EBP1	COMP	CXCL8	MTPN
CARDIOMETABOLIC	CARDIOMETABOLIC	CARDIOMETABOLIC	CARDIOMETABOLIC	CARDIOMETABOLIC
0.335550079	1.109415664	0.603196516	0.319635599	0.935753212
0.303759198	1.161508732	0.516342408	0.312537238	0.916876928
0.24028192	1.19731271	0.554208215	0.306742505	0.74241615
0.351549561	0.80068129	0.534736478	0.321747312	0.952704032
0.290095536	1.043477867	0.520150133	0.691691197	0.89001404
0.343004641	1.266195348	0.751632836	0.550723471	0.675440223
0.269115986	1.072665309	0.470152274	0.356284098	1.050663001
0.290135754	0.654198338	0.406210659	0.349969233	0.891681259
0.265061676	0.886750412	0.431729626	0.274523203	1.266195348
0.217788548	0.888226796	0.336178648	0.432988316	0.869766471
0.215058342	0.703245344	0.538679848	0.204560571	0.971711535
0.349944976	1.669712832	0.901437892	0.601985232	1.011712938
0.223771778	0.70383053	0.39112455	0.305384766	1.11211039
0.27431397	0.842588424	0.365793159	0.341297086	1.094369555
0.167670408	0.761174956	0.280369244	0.384165535	1.106037269
0.212362362	1.217734821	0.230573721	0.250694109	1.041382451
0.317361714	1.627547896	0.509728173	0.419982174	1.062232812
0.308747637	0.772175133	0.595015848	0.396254713	0.761755544
0.331251754	0.776092221	0.497890364	0.295064037	1.17609125
0.22144188	0.796695317	0.377932371	0.332516999	0.801569767
0.240748715	0.620541671	0.427619634	0.402120558	1.008701984
0.233161259	1.279340241	0.22664402	0.695923196	0.862502175
0.210617906	0.767160317	0.230046913	0.369257723	1.279428921
0.226974165	0.636132852	0.312905734	0.328599001	1.180501603
0.252402832	0.847508651	0.424106941	0.389852421	0.836014575
0.330243026	1.09179349	0.453256621	0.504385952	0.729763045
0.300617312	0.797911144	0.259858832	0.517273791	1.108032348
0.262083785	2.710389142	0.373815942	0.641535053	1.13115764
0.323245009	1.492364555	0.517381366	0.516915368	1.116899983
0.285685556	0.939327401	0.345797657	1.241341445	1.012976005
0.307786103	0.897572263	0.285013073	0.299452691	0.94219658
0.318529744	0.78561806	0.480863724	0.251930902	0.864956809
0.192362569	0.586783415	0.37051401	0.340116287	0.997922719
0.194858666	0.671286251	0.30149375	0.590987696	0.790479525
0.355716549	2.200757219	0.249066	0.40779048	1.16360387

P19957,PI3,CARDIOMETABOLIC	P10721,KIT,CARDIOMETABOLIC	Q9UGM5,FETUB,CARDIOMETABOLIC	P01589,IL2RA,CARDIOMETABOLIC	Q9H2A7,CXCL16,CARDIOMETABOLIC
P19957	P10721	Q9UGM5	P01589	Q9H2A7
PI3	KIT	FETUB	IL2RA	CXCL16
CARDIOMETABOLIC	CARDIOMETABOLIC	CARDIOMETABOLIC	CARDIOMETABOLIC	CARDIOMETABOLIC
0.160628523	0.526425033	0.507331273	0.660348722	0.409433201
0.238671796	0.376129144	0.516091938	0.893103945	0.374646012
0.2249537	0.572679553	0.459551993	0.935169641	0.368643954
0.151375119	0.565618024	0.400312897	0.468623108	0.469956784
0.16381053	0.529939658	0.41549413	0.636838738	0.423930597
0.199381022	0.449533333	0.320367566	0.769930432	0.36208492
0.141581042	0.460157612	0.436665362	0.641935388	0.32653284
0.204518039	0.402622583	0.296211591	0.722465199	0.301640071
0.157814317	0.463069294	0.319790725	0.275609973	0.26110464
0.211701002	0.317471722	0.249930695	0.550380019	0.292295597
0.161835498	0.417891407	0.24084886	0.323312233	0.276988887
0.318066426	0.580875143	0.571727662	0.624468195	0.519897816
0.200489696	0.471588355	0.268277878	0.617837781	0.293371372
0.198553542	0.408158102	0.225797278	0.43708931	0.277430825
0.152692383	0.369232129	0.258654823	0.264217879	0.212656963
0.268036244	0.316395284	0.295678244	0.572997202	0.262756799
0.114569902	0.625507895	0.356951508	0.657699282	0.40595733
0.220446427	0.523513991	0.460412849	0.48524996	0.370334279
0.164504619	0.569315388	0.44534645	0.319591291	0.418616186
0.325944898	0.42637655	0.287313961	0.544951597	0.312667246
0.156604763	0.438667582	0.26508005	0.357892943	0.245364766
0.185321221	0.396227248	0.558449991	0.674551267	0.41302471
0.158878961	0.452597337	0.307381022	0.431909214	0.381855842
0.354215684	0.437999158	0.229983139	0.386596371	0.316987971
0.144886994	0.486057871	0.197250382	0.878491359	0.328621779
0.137566396	0.573156093	0.364073086	0.579507806	0.326713959
0.237961486	0.379875876	0.421878655	0.697613566	0.357050489
0.198581069	0.416878825	0.350794978	0.598614861	0.398458119
0.2967459	0.43718021	0.357149498	0.71946679	0.340092713
0.142832877	0.411824053	0.294145119	0.47813834	0.326012684
0.327099169	0.571291908	0.236350529	0.195223685	0.275037454
0.251059287	0.413798408	0.364123561	0.719317197	0.35384759
0.157083113	0.449845033	0.183951838	0.465999399	0.343075974
0.400063246	0.43669563	0.154834621	0.451563255	0.30434931
0.198305968	0.504630741	0.413483023	0.484409814	0.350211897

Q16820,MEP1B,CARDIOMETABOLIC	P04070,PROC,CARDIOMETABOLIC	P40225,THPO,CARDIOMETABOLIC	P78380,OLR1,CARDIOMETABOLIC	P24158,PRTN3,CARDIOMETABOLIC
Q16820	P04070	P40225	P78380	P24158
MEP1B	PROC	THPO	OLR1	PRTN3
CARDIOMETABOLIC	CARDIOMETABOLIC	CARDIOMETABOLIC	CARDIOMETABOLIC	CARDIOMETABOLIC
0.124127958	0.325019909	0.38724003	0.475033117	0.323962785
0.339433297	0.265502988	0.44630442	0.271269734	0.372651766
0.366351391	0.198443471	0.77630743	0.448692819	0.397657972
0.249066	0.309218813	0.308854659	0.245551919	0.638562616
0.392863489	0.308426792	0.416936621	0.352818961	0.470967692
0.538381223	0.214864642	0.482968164	0.311024471	0.37221291
0.233484713	0.25685036	0.478569381	0.314951153	0.430355258
0.160405999	0.162780524	0.233922088	0.26811057	0.291688416
0.141355508	0.248945182	0.449782676	0.433258512	0.472963252
0.16530474	0.22708432	0.307061598	0.292964955	0.382571153
0.179904104	0.183251892	0.433078363	0.41549413	0.3408479
0.433829481	0.329716959	0.485182695	0.593286146	0.517668343
0.193767699	0.179480623	0.35401932	0.283495944	0.2997642
0.215655438	0.171229122	0.334481889	0.190003777	0.18439865
0.336225255	0.167333708	0.295780736	0.260309524	0.349363308
0.122240987	0.237269736	0.301055212	0.514270748	0.400507177
0.279089548	0.251198543	0.574428805	0.531963808	0.496752798
0.182390185	0.265521391	0.372832621	0.293493407	0.2880917
0.277469288	0.308704838	0.50149249	0.31930344	0.570975204
0.219318589	0.208381629	0.412795744	0.330953401	0.41612821
0.166685441	0.182630547	0.391911551	0.343242476	0.409149502
0.380508345	0.192642777	0.347310998	0.344649063	0.256387885
0.425431865	0.136030305	0.283338784	0.2913853	0.262957217
0.14869211	0.196854282	0.549122526	0.22642419	0.341439057
0.336528361	0.158263448	0.22510968	0.563231528	0.28813164
0.294696126	0.204333832	0.433709215	0.296972243	0.485923126
0.289412673	0.336341802	0.502397085	0.359135457	0.344147754
0.312710594	0.324164947	0.444791154	0.449377563	0.363871257
0.358314914	0.256885969	0.380086582	0.317868068	0.557058212
0.305490622	0.283063964	0.33844657	0.296601952	0.27905086
0.113133649	0.242087417	0.330861654	0.178253215	0.31281899
0.254722799	0.219318589	0.313014197	0.265374196	0.218287285
0.168696272	0.227100061	0.348879323	0.173114683	0.272438028
0.395650915	0.221595425	0.43724082	0.221795193	0.271363766
0.164310889	0.302372743	0.382809888	0.454389058	0.359384477

Q13275,SEMA3F,CARDIOMETABOLIC	P19022,CDH2,CARDIOMETABOLIC	Q8WVQ1,CANT1,CARDIOMETABOLIC	Q8N1Q1,CA13,CARDIOMETABOLIC	P04746,AMY2A,CARDIOMETABOLIC
Q13275	P19022	Q8WVQ1	Q8N1Q1	P04746
SEMA3F	CDH2	CANT1	CA13	AMY2A
CARDIOMETABOLIC	CARDIOMETABOLIC	CARDIOMETABOLIC	CARDIOMETABOLIC	CARDIOMETABOLIC
0.401452165	0.254246532	0.420856411	0.480697098	0.475000191
0.462973011	0.28753311	0.556132286	0.491989694	0.266166332
0.62161792	0.458216082	0.454074209	1.093990341	0.323424304
0.528728867	0.397685537	0.429818653	0.60634046	0.410968579
0.580553127	0.398679133	0.5030243	0.654289036	0.512278412
0.497545372	0.289573202	0.567227742	0.737236804	0.556132286
0.435245112	0.213276956	0.461723158	0.716580157	0.409575124
0.409404822	0.297487303	0.453413735	0.428123817	0.350673423
0.339645113	0.217290951	0.343456668	0.458629163	0.466193243
0.354510435	0.306997753	0.380877772	0.757805754	0.243045782
0.312602235	0.274066899	0.308790441	0.431669779	0.455776991
0.508528311	0.405310653	0.51666462	0.612635129	0.50916318
0.30834129	0.293208737	0.366275219	0.516593	0.492535631
0.338681244	0.26811057	0.337930858	0.351695797	0.239699699
0.244126362	0.220767545	0.242171333	0.412395358	0.296560838
0.350794978	0.276796959	0.368695062	0.519069635	0.361934365
0.376703149	0.278529106	0.509763506	0.371285274	0.727944315
0.443344474	0.347022233	0.416416748	0.575464964	0.569315388
0.492160234	0.259516829	0.389852421	0.623214195	0.28631993
0.479931363	0.305766021	0.371105169	0.391205891	0.338352745
0.479100426	0.406098049	0.460317118	0.848096303	0.294512342
0.508281631	0.236990314	0.443928735	0.542802774	0.340470097
0.341439057	0.204149792	0.321056698	0.42164478	0.428985265
0.349702496	0.201088154	0.349000256	0.488930056	0.403181123
0.461627156	0.227052842	0.490661507	0.579748867	0.580915408
0.396447024	0.280583096	0.533477748	0.689298127	0.510329166
0.441504485	0.227352063	0.468298395	0.465515142	0.255997211
0.352354611	0.203542222	0.425756364	0.344625175	0.488185043
0.544800525	0.402148432	0.533921667	0.61595634	0.378902877
0.413655021	0.263943309	0.367292158	0.699792933	0.362285759
0.34231585	0.210530331	0.297239963	0.329328666	0.437847385
0.407931834	0.203626891	0.435788491	0.464001058	0.434943527
0.268985442	0.139593145	0.362335986	0.38209413	0.256263515
0.410285479	0.228204635	0.359434302	0.527338049	0.352379036
0.33464422	0.242305658	0.431609941	0.522064513	0.313296379

P05107,ITGB2,CARDIOMETABOLIC	P02144,MB,CARDIOMETABOLIC	Q6GTS8,PM20D1,CARDIOMETABOLIC	P33151,CDH5,CARDIOMETABOLIC	P07858,CTSB,CARDIOMETABOLIC
P05107	P02144	Q6GTS8	P33151	P07858
ITGB2	MB	PM20D1	CDH5	CTSB
CARDIOMETABOLIC	CARDIOMETABOLIC	CARDIOMETABOLIC	CARDIOMETABOLIC	CARDIOMETABOLIC
1.147663739	0.213617242	0.039716987	0.603405604	0.317493729
0.885767519	0.097957516	0.125095344	0.782357551	0.401897637
0.881235794	0.117684808	0.282671826	0.772817678	0.303191245
0.713606173	0.107186863	0.052425045	0.527996401	0.672031145
0.888042113	0.298540797	0.065752976	0.96132756	0.297920644
0.774372701	0.241065985	0.484443392	0.66328463	0.334806629
0.625811467	0.113377007	0.051517272	0.505085664	0.242911046
0.545216074	0.114023255	0.037951481	0.524640105	0.24635318
0.74442582	0.073709787	0.077847796	0.399093863	0.311391183
0.564834455	0.061477402	0.178129702	0.472602773	0.259390941
0.562880276	0.071026441	0.043840255	0.519537575	0.434853093
1.190691773	0.224984887	0.063963824	0.931288456	0.383261238
0.55041817	0.072267961	0.907078931	0.457074107	0.298478723
0.465031388	0.082452097	0.082058694	0.49909972	0.2639982
0.444298138	0.050467555	0.136237899	0.324637148	0.217020014
0.59234106	0.065298787	0.272645831	0.318176679	0.235728811
0.900813279	0.088474162	0.103729633	0.518638065	0.710398283
0.898755127	0.075457076	0.174101435	0.599445293	0.318949517
0.950527321	0.100571958	0.127599979	0.640957232	0.307508885
0.759435845	0.18716726	0.062703944	0.494040082	0.303044171
0.576502992	0.078541557	0.270406175	0.404048393	0.244838103
0.49806295	0.149861323	0.079820327	0.420069516	0.273308077
0.697033548	0.133055495	0.071510554	0.479233279	0.285744968
0.429223211	0.075692806	0.510223057	0.355839852	0.21697489
0.72502367	0.065023274	0.060542833	0.611744021	0.351915265
0.593820992	0.075107459	0.097476618	0.498442849	0.353357393
0.638872524	0.076760079	0.105696536	0.601359661	0.447512535
0.627418511	0.067414372	0.227320547	0.449969774	0.256814755
0.68216848	0.138331345	0.121961694	0.612592666	0.596626515
0.415955183	0.095450727	0.073755784	0.432268616	0.255023129
0.592998352	0.134978357	0.137757235	0.497028333	0.4659671
0.67245051	0.107082898	0.113573645	0.512313921	0.336878439
0.668314926	0.108615357	0.052425045	0.352867875	0.219775123
0.4399465	0.059933233	0.296889917	0.481497429	0.300992616
0.519789718	0.049240868	0.138975261	0.52355028	0.316110311

P08833,IGFBP1,CARDIOMETABOLIC	Q15846,CLUL1,CARDIOMETABOLIC	P41236,PPP1R2,CARDIOMETABOLIC	P27487,DPP4,CARDIOMETABOLIC	O14793,MSTN,CARDIOMETABOLIC
P08833	Q15846	P41236	P27487	O14793
IGFBP1	CLUL1	PPP1R2	DPP4	MSTN
CARDIOMETABOLIC	CARDIOMETABOLIC	CARDIOMETABOLIC	CARDIOMETABOLIC	CARDIOMETABOLIC
0.75178915	0.707498993	0.466290195	0.878674055	0.339715747
0.22642419	0.845748137	0.586946128	0.921336453	0.615188313
0.769236967	0.982343847	0.622998243	0.86357896	0.509692843
0.689059276	1.09497657	0.778894589	0.907141807	0.431012017
5.731846601	0.621445595	0.996540263	0.891681259	0.389339332
0.882947767	1.663128913	0.876970367	0.810621306	0.673663482
0.277142525	0.975558302	0.572481112	0.705980382	0.833353207
0.934975198	1.04630249	0.611023596	0.703099123	0.520366503
0.211451691	0.582851394	0.479665306	0.648284956	0.36905302
0.1002379	0.40615435	0.649859615	0.683778042	0.228647968
0.120624929	0.375191747	0.49875389	0.581277915	0.449377563
0.365337056	1.123733553	0.885951728	1.193335743	0.663192685
0.052494133	0.738515446	0.491069798	0.810003474	0.321078952
0.275629078	0.569315388	0.412795744	0.634767425	0.382465097
0.073337759	0.461115479	0.414889773	0.607518396	0.26434611
0.066800374	0.615700225	0.488963947	0.525185868	0.242742731
0.336062157	1.375923252	0.525586455	0.799849238	0.521811267
0.692410736	0.636397468	0.736828107	1.035408452	0.273801072
0.137566396	0.698145672	0.633756261	0.76731986	0.459902517
0.238572556	0.473980628	0.573792096	0.752571208	0.479698555
0.15965172	0.49806295	0.697371833	0.797192477	0.341368064
0.573156093	0.562802249	0.664987902	0.553056968	0.27108177
0.738873863	0.567857167	0.386971709	0.688247801	0.246097175
0.064784837	0.436907568	0.52355028	0.678208138	0.639404144
0.137910098	0.915606748	0.714794283	0.739642485	0.303485606
0.901500377	1.100988958	0.490933663	0.849449449	0.304497017
0.138734644	0.662779093	0.62420854	0.768863821	0.619123875
0.336761706	0.622566563	0.472570016	0.647745952	0.224113273
0.524131238	0.587230986	0.57164841	0.69399636	0.257813551
0.227289036	0.736879182	0.6279406	0.627505496	0.205270754
0.466516496	0.423519411	0.757858283	0.43455178	0.297982601
0.21953152	0.423372656	0.497821347	0.842471624	0.310421416
1.029825572	0.375582045	0.406520497	0.568566105	0.212303491
0.079931058	0.523296313	0.679196062	0.608445522	0.310120328
0.175336711	0.741850302	0.474736868	0.736011391	0.407423189

Q16663,CCL15,CARDIOMETABOLIC	P04085,PDGFA,CARDIOMETABOLIC	O00161,SNAP23,CARDIOMETABOLIC	P01034,CST3,CARDIOMETABOLIC	Q9NY25,CLEC5A,CARDIOMETABOLIC
Q16663	P04085	O00161	P01034	Q9NY25
CCL15	PDGFA	SNAP23	CST3	CLEC5A
CARDIOMETABOLIC	CARDIOMETABOLIC	CARDIOMETABOLIC	CARDIOMETABOLIC	CARDIOMETABOLIC
0.557676352	0.453036753	0.46904557	0.248169891	0.455934979
0.288011835	0.335131686	0.409177863	0.309926925	0.476153948
0.26266575	0.353039129	0.622782366	0.259498841	0.511675124
0.305003986	0.2731187	0.437847385	0.295452887	0.417428208
0.236908193	0.360357306	0.843172664	0.30418059	0.510647625
0.258977739	0.243399819	0.616340714	0.297941295	0.416936621
0.259588792	0.40528256	0.455998189	0.237072463	0.336691686
0.268948156	0.280194395	0.391993055	0.335154916	0.34077703
0.165167301	0.47083713	0.484846509	0.194656173	0.34231585
0.149539653	0.916051111	0.274961208	0.176397253	0.417630795
0.370000724	0.451406782	0.273763118	0.23759889	0.256601232
0.970096385	0.668037039	0.458692747	0.495308746	0.575903901
0.165499642	0.399315229	0.401786223	0.20770387	0.354092944
0.117619568	0.315125848	0.489540458	0.199699137	0.26105035
0.287094978	0.435607289	0.327325976	0.143438081	0.267887654
0.178587127	0.612422843	0.536407011	0.157541083	0.382359069
0.293147773	4.177963823	0.514342046	0.227888496	0.397520179
0.217185546	0.29816855	0.465482876	0.225484473	0.386301718
0.267646372	0.309948408	0.381194709	0.255748911	0.521377417
0.545178283	0.344076198	0.26425451	0.271702548	0.313426702
0.132292208	0.28753311	0.44028207	0.186998681	0.285744968
0.220003747	0.53067482	0.749707635	0.269060031	0.316834205
0.191604055	0.377173442	0.515341257	0.200976678	0.322238326
0.192589373	0.261303798	0.354756248	0.19900823	0.221810567
0.207631897	0.239102314	0.40983071	0.228679667	0.469435874
0.18307415	0.598490395	0.397327347	0.241383673	0.422346793
0.229664536	0.301786463	0.479465861	0.219775123	0.369257723
0.291203581	1.239192227	0.711383787	0.229919383	0.294206291
0.244821133	0.464419354	0.472602773	0.335782745	0.325019909
0.196854282	0.268631427	0.570935629	0.166016668	0.462491899
0.273478628	0.634151742	0.321212513	0.264291146	0.246626546
0.344816329	0.36759779	0.384751805	0.289974914	0.347335072
0.304243849	0.52507667	0.352770053	0.198223512	0.206512355
0.220889998	0.260634505	0.478105199	0.219121052	0.29889279
0.223880379	0.48350409	0.606298433	0.221979753	0.36946254

Q9HBB8,CDHR5,CARDIOMETABOLIC	O43854,EDIL3,CARDIOMETABOLIC	P46531,NOTCH1,CARDIOMETABOLIC	Q9UKJ0,PILRB,CARDIOMETABOLIC	Q9HD89,RETN,CARDIOMETABOLIC
Q9HBB8	O43854	P46531	Q9UKJ0	Q9HD89
CDHR5	EDIL3	NOTCH1	PILRB	RETN
CARDIOMETABOLIC	CARDIOMETABOLIC	CARDIOMETABOLIC	CARDIOMETABOLIC	CARDIOMETABOLIC
0.432028981	1.508902932	0.544573996	0.415522931	0.173354837
0.608150374	0.835030038	0.489065635	0.505050656	0.318441441
0.607644739	1.80800667	0.494931236	0.554169801	0.30729581
0.288691393	1.133669413	0.424724725	0.489710149	0.245909606
0.543668822	1.164652856	0.56424749	0.424989764	0.419255031
0.420943935	0.888657871	0.439215234	0.339927739	0.382226577
0.205726565	0.889458994	0.492433221	0.434732543	0.188926895
0.315256932	1.069398827	0.385419107	0.518925739	0.209903772
0.357298064	1.117984356	0.406464145	0.324502163	0.294798278
0.392428031	1.394646993	0.3739196	0.373479251	0.326691313
0.207660683	1.138709703	0.364856232	0.196350068	0.224035615
0.517381366	1.457908321	0.667712984	0.526425033	0.259750782
0.610684867	1.575380147	0.425844907	0.291910903	0.216179256
0.195142511	1.259280785	0.347768701	0.276796959	0.184079388
0.108939571	0.856485068	0.316680513	0.170164265	0.201353157
0.322193658	0.761069442	0.380139277	0.184603268	0.335271092
0.293391708	0.711926397	0.483738745	0.361984543	0.241166262
0.315606757	0.740719899	0.453665231	0.361934365	0.187206184
0.440740079	1.342293352	0.466516496	0.403656492	0.190861762
0.324952331	1.108185964	0.444482955	0.361533189	0.220018997
0.321034444	1.80025341	0.39713461	0.434943527	0.174572716
0.357199013	0.804408371	0.362110019	0.325493356	0.174016981
0.327961871	1.07467468	0.359833148	0.218211645	0.189084106
0.383048772	1.091188239	0.353528885	0.226738298	0.169740178
0.240048862	0.959463613	0.488218883	0.520222246	0.153349992
0.271307343	0.79940583	0.424813053	0.19783917	0.181645811
0.548665969	0.614038066	0.448786132	0.502710595	0.356803086
0.45584018	1.06984367	0.479366169	0.3778276	0.374438322
0.415897524	1.082299661	0.473914924	0.205498533	0.41065535
0.312299031	0.846275907	0.367470413	0.234571558	0.415407739
0.368337452	0.938026119	0.429550602	0.4430987	0.328712905
0.355297637	0.856306986	0.463358262	0.24235605	0.283869548
0.295555301	0.846099947	0.358041817	0.28269142	0.178710958
0.34191272	0.918594468	0.350649117	0.266018779	0.228442028
0.400590469	0.886258829	0.464902471	0.21997325	0.261086542

Q13822,ENPP2,CARDIOMETABOLIC	Q96LA6,FCRL1,CARDIOMETABOLIC	P05362,ICAM1,CARDIOMETABOLIC	O14786,NRP1,CARDIOMETABOLIC	O15031,PLXNB2,CARDIOMETABOLIC
Q13822	Q96LA6	P05362	O14786	O15031
ENPP2	FCRL1	ICAM1	NRP1	PLXNB2
CARDIOMETABOLIC	CARDIOMETABOLIC	CARDIOMETABOLIC	CARDIOMETABOLIC	CARDIOMETABOLIC
0.763658749	0.614080629	0.526972653	0.441933132	0.472471758
0.605794338	0.761069442	0.536035331	0.401702682	0.513095757
0.659159725	1.068657834	0.568329694	0.657243557	0.479499096
0.676986983	0.980779004	0.664204775	0.710496772	0.479731807
0.905194673	0.763394131	0.54381958	0.587068193	0.56413017
0.575225684	0.759646434	0.518709968	0.594892131	0.409518349
0.621574834	1.036126391	0.576143463	0.577703043	0.537002236
0.534069722	0.743652227	0.478602554	0.611616825	0.411396094
0.471130945	0.482499717	0.478934411	0.435667681	0.377644322
0.527484278	0.707008762	0.368771738	0.429788862	0.277623192
0.557212682	0.415062357	0.511710592	0.410683816	0.351890873
0.756703488	0.850568894	0.604075173	0.741541839	0.661127303
0.670774616	0.842004588	0.415839872	0.52166661	0.396831925
0.517776	0.466613515	0.314623862	0.460923746	0.356506429
0.424283359	0.32766648	0.370642443	0.289312387	0.276490152
0.418616186	0.550570799	0.433468782	0.480064447	0.422434626
0.748046573	0.763182502	0.68382544	0.655287538	0.47440792
0.730471555	0.851985038	0.404973665	0.450562766	0.463294031
0.704806923	0.565147752	0.678866594	0.485754747	0.492194349
0.516413994	0.72502367	0.488117371	0.595057093	0.385686352
0.496752798	0.737901421	0.441076255	0.48260006	0.370231615
0.577142709	0.401730527	0.4315202	0.438576374	0.444051835
0.561360711	0.485620086	0.413225159	0.403432719	0.427560357
0.470608734	0.441596303	0.357199013	0.43178948	0.315738042
0.656515055	0.682830781	0.363417551	0.507788631	0.395705768
0.640779545	0.581237625	0.522100701	0.528069602	0.394555457
0.560855102	0.562841261	0.476814495	0.570777354	0.461051559
0.524967494	0.76212524	0.461467196	0.508739846	0.365590377
0.559457328	0.688581822	0.545178283	0.46616093	0.643048742
0.484275526	0.383739718	0.454200122	0.446923558	0.378194425
0.360707169	0.391070333	0.323962785	0.326736606	0.394172764
0.739539956	0.895707754	0.537896311	0.654379746	0.516485589
0.488185043	0.412652704	0.364148801	0.424165739	0.348710086
0.484745698	0.459934397	0.281635285	0.506347593	0.33079286
0.594727215	0.585808081	0.50986952	0.414487357	0.551334582

P09417,QDPR,CARDIOMETABOLIC	Q8WTU2,SSC4D,CARDIOMETABOLIC	Q99650,OSMR,CARDIOMETABOLIC	P14543,NID1,CARDIOMETABOLIC	Q9UBP4,DKK3,CARDIOMETABOLIC
P09417	Q8WTU2	Q99650	P14543	Q9UBP4
QDPR	SSC4D	OSMR	NID1	DKK3
CARDIOMETABOLIC	CARDIOMETABOLIC	CARDIOMETABOLIC	CARDIOMETABOLIC	CARDIOMETABOLIC
0.439733089	0.74298243	0.8005703	0.44031259	0.541975672
0.433619037	1.444730102	0.762600827	0.453005352	0.41116803
0.410626887	0.494725442	0.605248708	0.521124504	0.590619134
0.321479801	0.199201443	0.771586603	0.45015695	0.48333655
0.420331651	0.561282895	0.803906711	0.373945519	0.477608362
0.525695759	0.270649945	0.696019678	0.468590626	0.451219087
0.327212553	0.490797566	0.669613259	0.46852567	0.43431088
0.353602407	0.548133798	0.609374063	0.410826173	0.497890364
0.335294332	0.903690086	0.720365004	0.398540985	0.452095668
0.371516966	0.411652816	0.66840758	0.464194071	0.319591291
0.291668199	0.12284406	0.624165274	0.272268125	0.480463919
0.59295725	0.730623468	0.674037144	0.93789609	0.897696702
0.253384468	0.905069195	0.662457589	0.441167983	0.554746283
0.298623581	0.846510577	0.594521134	0.324727169	0.306020456
0.260905633	0.644431982	0.58422662	0.295821743	0.284736629
0.30587201	1.026689546	0.567542368	0.352525616	0.287852171
0.600693101	0.445562586	0.759067454	0.578143688	0.45936091
0.414544821	0.908274323	0.649139297	0.335829297	0.464709164
0.302184172	0.775178252	0.662228038	0.36966747	0.367827181
0.303927685	1.01995111	0.623214195	0.450781433	0.335782745
0.282417227	0.659205416	0.630382767	0.320434191	0.3637956
0.516235049	0.286419178	0.653337339	0.542689914	0.489336906
0.294839148	0.160851356	0.595593535	0.330861654	0.419400359
0.254511015	1.949899814	0.533034198	0.394200087	0.326397067
0.443713391	0.1960237	0.554977043	0.318529744	0.438515578
0.441963766	0.677597285	0.750487527	0.421674008	0.421206615
0.36128268	1.332744289	0.723768387	0.403041416	0.430474594
0.621101088	0.309626316	0.578344092	0.475692109	0.446366295
0.478635729	1.403860905	0.502815141	0.578785225	0.434883236
0.281050249	1.640004758	0.730370297	0.347214716	0.380666627
0.300325733	1.364147835	0.59737137	0.374775877	0.370616752
0.414343731	0.243012091	0.68060988	0.601526416	0.376912096
0.289914621	0.402204185	0.599112982	0.371955001	0.368107743
0.315563008	1.296569912	0.595799988	0.364729804	0.278374699
0.443867197	0.530013128	0.658931317	0.451907686	0.310895147

P78324,SIRPA,CARDIOMETABOLIC	Q9UMF0,ICAM5,CARDIOMETABOLIC	O95445,APOM,CARDIOMETABOLIC	P30530,AXL,CARDIOMETABOLIC	Q15113,PCOLCE,CARDIOMETABOLIC
P78324	Q9UMF0	O95445	P30530	Q15113
SIRPA	ICAM5	APOM	AXL	PCOLCE
CARDIOMETABOLIC	CARDIOMETABOLIC	CARDIOMETABOLIC	CARDIOMETABOLIC	CARDIOMETABOLIC
0.490117648	0.306147753	0.427856822	0.428955531	0.331205836
0.545858908	0.482232236	0.310163323	0.507999858	0.315935071
0.533108097	0.530638037	0.361307723	0.631126016	0.284559056
0.392591271	0.570144692	0.46554741	0.587149584	0.209947425
0.778516758	0.552941975	0.360157537	0.577342766	0.231550696
0.667851846	0.689489267	0.255023129	0.537002236	0.326691313
0.656014678	0.473225591	0.263139549	0.642157904	0.24989605
0.685201398	0.374827836	0.213676477	0.595428425	0.277719426
0.342814491	0.412138173	0.310227826	0.480997066	0.194399983
0.370026372	0.224844578	0.265447783	0.391205891	0.159618525
0.17736583	0.380640242	0.26350459	0.43431088	0.200531391
0.73417709	0.363593925	0.446428179	0.650355297	0.477575258
0.675299783	0.480264141	0.279012178	0.412481123	0.279961432
0.30665747	0.265723919	0.264309466	0.393272169	0.125651518
0.326035282	0.474506581	0.294880024	0.272494686	0.113345577
0.314493041	0.42164478	0.277757929	0.379638971	0.159110395
0.427975466	0.843464936	0.336808394	0.551525693	0.346445423
0.741901725	0.492296709	0.39608995	0.560388789	0.169059149
0.663238656	0.512349433	0.284243645	0.476451081	0.316417215
0.497648845	0.32273009	0.293737629	0.444359735	0.211539649
0.356012549	0.311132283	0.286498601	0.527118781	0.185000362
0.601651513	0.404048393	0.299701872	0.485923126	0.205213849
0.580834881	0.376624824	0.321234779	0.458597374	0.236694814
0.540437616	0.611404892	0.278799523	0.377618146	0.209206561
0.749811574	0.498270131	0.230461873	0.494896931	0.254652185
0.403628514	0.489235162	0.377932371	0.544951597	0.295002687
0.527996401	0.416099367	0.354117489	0.518817842	0.266572525
0.605122863	0.449252987	0.313361534	0.397713103	0.241618027
0.656879205	0.388342094	0.277238592	0.560699621	0.332586151
0.306041669	0.488557408	0.395048038	0.455366479	0.206412179
0.626115187	0.631519856	0.239865903	0.389339332	0.194642681
0.588943034	0.587190283	0.32506497	0.494999852	0.312342328
0.399176861	0.415551734	0.274884983	0.468298395	0.196826994
0.589065514	0.403600537	0.287015389	0.458057304	0.229282794
0.557599047	0.556170836	0.417804518	0.506839195	0.197100044

P21964,COMT,CARDIOMETABOLIC	Q9Y5K6,CD2AP,CARDIOMETABOLIC	Q8TE57,ADAMTS16,CARDIOMETABOLIC	P10644,PRKAR1A,CARDIOMETABOLIC
P21964	Q9Y5K6	Q8TE57	P10644
COMT	CD2AP	ADAMTS16	PRKAR1A
CARDIOMETABOLIC	CARDIOMETABOLIC	CARDIOMETABOLIC	CARDIOMETABOLIC
0.363467935	0.334737016	0.42806447	0.187050535
0.44813335	0.431430477	0.395020656	0.313752748
0.236579996	0.566913292	0.604158922	0.198746313
0.315366211	0.345725758	0.396831925	0.239932418
0.420681418	0.399204531	0.441259731	0.261394375
0.354166583	0.508916192	0.485653748	0.284736629
0.310421416	0.555323364	0.422200444	0.162915977
0.302603378	0.376416037	0.371568473	0.19504785
0.408724322	0.436453542	0.565226104	0.201046343
0.409404822	0.509763506	0.441320907	0.225719036
0.386194628	0.358463964	0.349314879	0.14058358
0.312623904	0.674925422	0.487542537	0.272740339
0.259444885	0.332701436	0.481597564	0.254599237
0.40351662	0.400340645	0.429818653	0.469631147
0.272967293	0.43077308	0.351866483	0.154770241
0.46888304	0.575824069	0.401869781	0.373790032
0.783225695	0.951977908	0.443867197	0.633580571
0.378115789	0.380798579	0.441779996	0.326849864
0.507507131	0.296047382	0.534069722	0.250694109
0.348927691	0.389366319	0.555246385	0.151648171
0.344219325	0.345318612	0.536369832	0.250069324
0.383846128	0.465902507	0.461787171	0.324817215
0.32139068	0.244804164	0.334922685	0.212362362
0.496236583	0.219866544	0.511533277	0.282809012
0.259193241	0.341510064	0.50215338	0.163992302
0.413339745	0.456282744	0.395048038	0.94461608
0.437695665	0.429669715	0.36319091	0.24130003
0.607560507	0.850038447	0.428094142	0.14450587
0.324884766	0.624424911	0.477145113	0.238324636
0.306190197	0.535441179	0.384165535	0.127387886
0.337907435	0.451531956	0.353357393	0.170849745
0.222657791	0.393653988	0.363216085	0.188979284
0.396419545	0.439184791	0.444976176	0.180478639
0.295924285	0.35157393	0.502292626	0.170553941
0.475922973	1.525730339	0.430146499	0.258045969

P09093,CELA3A,CARDIOMETABOLIC	P01375,TNF,CARDIOMETABOLIC	P08571,CD14,CARDIOMETABOLIC	P13987,CD59,CARDIOMETABOLIC	Q92823,NRCAM,CARDIOMETABOLIC
P09093	P01375	P08571	P13987	Q92823
CELA3A	TNF	CD14	CD59	NRCAM
CARDIOMETABOLIC	CARDIOMETABOLIC	CARDIOMETABOLIC	CARDIOMETABOLIC	CARDIOMETABOLIC
0.534069722	0.757805754	0.432868283	0.343909292	0.718768954
0.68179031	0.806809513	0.309325998	0.339550956	0.721314335
0.61459162	0.628637395	0.275457185	0.371516966	0.682546859
0.575544746	0.808488972	0.309411774	0.316790285	0.613187418
0.666187413	0.833873242	0.32021216	0.420186	0.939718138
0.385900281	0.525914435	0.316483019	0.382491608	0.633800191
0.610642539	0.621230256	0.203514007	0.292133558	0.468395785
0.350527612	0.597040208	0.260851385	0.299784978	0.560000492
0.341439057	0.589719171	0.166396847	0.279631734	0.455050953
0.172324532	0.680138281	0.181721371	0.25709973	0.52719186
0.396035044	0.486260059	0.159695991	0.280194395	0.439002177
0.639492791	0.601401345	0.673990424	0.531632055	0.695730272
0.317141812	0.571489937	0.209947425	0.269414611	0.631957744
0.376703149	0.714447546	0.150256572	0.256885969	0.549084465
0.234197893	0.68767557	0.135109404	0.185436867	0.44537732
0.6056264	0.563973781	0.188038502	0.320811997	0.393845037
0.529352262	0.88779593	0.245807357	0.324120011	1.41705917
0.668268603	0.586986814	0.323155399	0.292741665	0.714794283
0.347431387	0.76955695	0.267943366	0.316395284	0.65533296
0.364022619	0.51788368	0.246455657	0.37579037	0.708922586
0.270424918	0.700958044	0.207631897	0.224004559	0.675768028
0.292153808	0.79940583	0.326351822	0.295125401	0.540699902
0.397354889	0.807424909	0.222010528	0.268817693	0.394391401
0.444945333	0.682594171	0.177574952	0.257831422	0.43915435
0.356827819	0.665080096	0.230717605	0.315650513	0.686009281
0.382730293	0.528765517	0.303485606	0.350163351	0.769290288
0.705637922	0.71946679	0.266313967	0.389420301	0.53056448
0.368311921	0.593450663	0.186843205	0.245007871	0.567857167
0.372574283	0.579829243	0.284322465	0.3747499	0.595882589
0.328986434	0.881480158	0.218468926	0.300700673	0.549503281
0.616554358	0.593327271	0.200865264	0.398044049	0.386542781
0.44881724	0.586376829	0.185655505	0.271476646	0.698145672
0.529022138	0.594685993	0.182744514	0.248824422	0.372290317
0.353945711	0.556595056	0.238969765	0.274409056	0.576662855
0.475922973	0.769023718	0.304644796	0.289312387	0.505681183

P17936,IGFBP3,CARDIOMETABOLIC	Q6WN34,CHRDL2,CARDIOMETABOLIC	Q9NWQ8,PAG1,CARDIOMETABOLIC	P13591,NCAM1,CARDIOMETABOLIC
P17936	Q6WN34	Q9NWQ8	P13591
IGFBP3	CHRDL2	PAG1	NCAM1
CARDIOMETABOLIC	CARDIOMETABOLIC	CARDIOMETABOLIC	CARDIOMETABOLIC
0.485283596	0.251930902	2.137611982	0.643360827
0.487441165	0.473619373	1.489058052	0.649859615
0.372729264	0.377461132	1.362541338	0.586946128
0.269004088	0.243231166	1.347233577	0.592012688
0.449471018	0.280933388	2.382704432	0.653201496
0.568250912	0.310529019	0.984456934	0.725375539
0.408724322	0.280058477	1.506499297	0.615103035
0.381644155	0.20495797	1.686345328	0.451406782
0.237121766	0.223043962	1.601473583	0.545518489
0.251948365	0.143826358	1.52467315	0.555554364
0.284677426	0.157617541	1.621354992	0.432928295
0.619381415	0.516557194	2.334478012	0.903001319
0.328895232	0.259588792	1.256752013	0.585645683
0.259732779	0.177808969	0.822678132	0.401563487
0.258888	0.109499789	0.905257419	0.415148675
0.256921584	0.158923018	1.835406981	0.338141736
0.375035742	0.476021948	1.653702823	0.642202417
0.296560838	0.302729254	1.462665627	0.642157904
0.310313851	0.225625182	1.381753244	0.566834706
0.528069602	0.278954166	1.825130977	0.656287564
0.350211897	0.194656173	1.109031236	0.473652203
0.36948815	0.212229925	1.202052577	0.50260607
0.325493356	0.201213638	1.278187957	0.515591362
0.324187417	0.245994847	0.720414937	0.435214944
0.385499261	0.231647015	1.120078619	0.410058032
0.375920631	0.216689327	1.545956794	0.4555559
0.422200444	0.185231325	1.316766922	0.664757475
0.417399275	0.227635899	1.548852757	0.397933703
0.455366479	0.263614202	0.894342909	0.619467286
0.304919432	0.210895468	1.072590961	0.343528095
0.347022233	0.151301689	0.863938187	0.502849995
0.41575341	0.354756248	1.286721653	0.46143521
0.244685413	0.185758483	1.662091723	0.410114882
0.324479671	0.168883466	1.129042661	0.405366845
0.246489825	0.114887998	2.120787394	0.504525817

P12318,FCGR2A,CARDIOMETABOLIC	P40818,USP8,CARDIOMETABOLIC	P46379,BAG6,CARDIOMETABOLIC	P01033,TIMP1,CARDIOMETABOLIC	Q16620,NTRK2,CARDIOMETABOLIC
P12318	P40818	P46379	P01033	Q16620
FCGR2A	USP8	BAG6	TIMP1	NTRK2
CARDIOMETABOLIC	CARDIOMETABOLIC	CARDIOMETABOLIC	CARDIOMETABOLIC	CARDIOMETABOLIC
0.294634852	0.318640157	0.766097543	0.319170673	0.592094764
0.476120945	0.301786463	0.586824089	0.330655316	0.556479328
0.39069102	0.253437164	0.599569957	0.33641175	0.478072061
0.185090147	0.145854318	0.527520842	0.393626703	0.475263661
0.242507286	0.174282546	0.757490657	0.327870953	0.61869488
0.318551823	0.106210625	0.587841859	0.345222883	0.518099106
0.397189668	0.146919724	0.326329202	0.338071429	0.377225733
0.367495885	0.245432805	0.625507895	0.287194495	0.451813724
0.183442522	0.261430614	0.504001524	0.269190612	0.393653988
0.160428237	0.199298119	0.551640391	0.307807438	0.501944584
0.23258017	0.170412138	0.413282448	0.314493041	0.405001737
0.243653018	0.406266975	0.982888725	0.718022023	0.70383053
0.265208699	0.160639657	0.372393553	0.240365209	0.55856613
0.254563944	0.168824946	0.486057871	0.224860164	0.306211421
0.205740825	0.17685023	0.404468709	0.198718763	0.284716893
0.311628697	0.231807637	0.399425958	0.216824547	0.40906443
0.172372317	0.253753566	1.141000956	0.57125231	0.508422577
0.386060806	0.150558911	0.666695549	0.289192091	0.634811426
0.241065985	0.145581607	0.673476728	0.290719551	0.337158763
0.173896403	0.263139549	0.379928541	0.278066143	0.448755025
0.264419412	0.176936059	0.654606576	0.306891374	0.462427788
0.262410981	0.176801203	0.539053362	0.358165926	0.422786144
0.120091005	0.14847583	0.619123875	0.253314225	0.339174591
0.156442023	0.156398654	0.453350883	0.267331176	0.416359025
0.217065146	0.106993866	0.557251307	0.273099769	0.524749212
0.179443305	0.180441114	0.58192293	0.320367566	0.499618914
0.475659138	0.220553414	0.491171923	0.314471243	0.454168641
0.216239202	0.252262908	0.599320655	0.294430697	0.441198564
0.391368623	0.185694115	0.585158759	0.346517471	0.525987347
0.304497017	0.122741923	0.694621995	0.279631734	0.507155476
0.171063041	0.178934068	0.304033036	0.334412342	0.321903462
0.36938572	0.184181492	0.337065296	0.27100662	0.459329071
0.239982315	0.296478625	0.395815496	0.271740217	0.348492618
0.24779174	0.142793281	0.309047393	0.288411381	0.393244911
0.200003895	0.249221424	0.645594414	0.29641698	0.426613049

P21549,AGXT,CARDIOMETABOLIC	Q99969,RARRES2,CARDIOMETABOLIC	P55082,MFAP3,CARDIOMETABOLIC	Q6EMK4,VASN,CARDIOMETABOLIC	Q9Y5X1,SNX9,CARDIOMETABOLIC
P21549	Q99969	P55082	Q6EMK4	Q9Y5X1
AGXT	RARRES2	MFAP3	VASN	SNX9
CARDIOMETABOLIC	CARDIOMETABOLIC	CARDIOMETABOLIC	CARDIOMETABOLIC	CARDIOMETABOLIC
0.347022233	0.21073473	0.896080346	0.603949572	0.807984768
0.309090239	0.259876845	0.838335716	0.686627717	0.840022572
0.472864912	0.194359563	1.015647659	0.620025734	0.84844909
0.428450369	0.23338763	0.795757084	0.598739352	0.757595675
0.395157583	0.213854282	0.827654183	0.538306593	0.888657871
0.440495749	0.224455289	0.541487523	0.598656355	0.970096385
0.294941349	0.159364257	1.050298932	0.580110646	0.785890382
0.243382948	0.111637993	0.810789887	0.669798941	0.756860856
0.429639934	0.171039329	1.27041513	0.386221397	0.672870138
0.266868328	0.15616034	0.919932556	0.391287248	0.835782815
0.274085897	0.187556868	0.841246207	0.499618914	0.664573191
0.391314371	0.271815569	0.950659101	0.806418142	1.235846875
0.260075067	0.120566416	0.562100499	0.637722196	0.738157203
0.211202673	0.152946606	0.950066234	0.354903818	0.676471003
0.343885455	0.152216853	0.87436042	0.343337656	0.653065681
0.31983506	0.156822014	0.991098259	0.371439719	0.831679744
0.344434127	0.250920109	0.781057139	0.486293765	1.13233434
0.353137026	0.17731666	0.729510172	0.403181123	0.623257395
0.294757413	0.217200601	1.000277297	0.456884055	0.695537401
0.361984543	0.16536204	0.922103118	0.476186954	0.633536656
0.312927423	0.175519107	0.890754641	0.458597374	0.725224717
0.660989839	0.237664775	0.905320169	0.473455258	1.059585495
0.243399819	0.165281826	1.049352943	0.45021936	0.724822678
0.288571354	0.171775954	0.765725921	0.409291327	0.686532536
0.39023092	0.146462172	0.655105879	0.494142826	0.675299783
0.354559584	0.19214935	0.585645683	0.520546879	0.924919685
0.282143301	0.166801018	1.271824848	0.626071789	0.83052759
0.33143549	0.219394612	1.245737416	0.484476972	1.234904948
0.297260567	0.163051543	1.025196174	0.562217396	1.096799638
0.306508715	0.163526913	0.670356296	0.56241228	0.614847274
0.261575622	0.170553941	0.679714121	0.443774907	0.679054842
0.464161896	0.203415286	0.954951918	0.56550042	0.683967652
0.278799523	0.135794787	0.731839911	0.397052036	0.631913941
0.342268398	0.135456356	0.749447851	0.564834455	0.60684501
0.223678733	0.247757391	0.808713164	0.504385952	1.051755965

P52789,HK2,CARDIOMETABOLIC	Q96KN2,CNDP1,CARDIOMETABOLIC	P21246,PTN,CARDIOMETABOLIC	Q12864,CDH17,CARDIOMETABOLIC	A1L4H1,SSC5D,CARDIOMETABOLIC
P52789	Q96KN2	P21246	Q12864	A1L4H1
HK2	CNDP1	PTN	CDH17	SSC5D
CARDIOMETABOLIC	CARDIOMETABOLIC	CARDIOMETABOLIC	CARDIOMETABOLIC	CARDIOMETABOLIC
1.043188594	0.148249588	0.929225081	0.431430477	0.326351822
0.742004582	0.152713552	0.827138026	0.665172302	0.295145858
0.853107824	0.085342016	1.130530567	0.560544184	0.188208019
0.698387673	0.153967739	0.87751762	0.510223057	0.302288919
0.898942037	0.15238576	0.653609111	0.483035123	0.397906122
0.627592493	0.230877582	0.818696141	0.791027634	0.216614241
1.079452679	0.094706024	1.125760552	0.772871247	0.172539669
0.788782801	0.057919783	1.16191135	0.480797067	0.195101936
1.220438841	0.170932662	1.140447473	0.397795814	0.223802801
0.809610553	0.101131196	1.101981497	0.458216082	0.107030954
0.574110362	0.12947092	1.300890923	0.330037074	0.17330678
0.929547182	0.244465028	1.93790789	0.430952271	0.282378079
0.789658074	0.137785884	1.667862088	0.457898581	0.206727182
1.438634393	0.102735023	1.478259963	0.355297637	0.129184062
0.977318022	0.110452655	0.683635869	0.324727169	0.144716367
0.981527096	0.129731435	0.911427623	0.297239963	0.187180234
0.721314335	0.100998096	0.888473099	0.363442742	0.202486833
0.839731493	0.123415885	1.734355926	0.372419366	0.184628861
1.229438867	0.185784236	1.449042591	0.287553041	0.185346914
0.804798767	0.107618649	1.901449996	0.569157562	0.246712035
0.859756486	0.113306301	0.634943445	0.544800525	0.20381046
0.799793799	0.249948019	0.898256889	0.238721432	0.223229562
0.723016261	0.094797972	0.813717543	0.386623169	0.149301442
1.877222946	0.093544718	0.722214855	0.232257969	0.192736271
0.732347359	0.192389237	1.097560145	0.370848027	0.123758541
0.866637158	0.160305963	1.373255433	0.439367481	0.304560342
1.453065765	0.13794834	1.301883181	0.660898213	0.196826994
1.093611258	0.177648819	1.231827294	0.46364741	0.356531141
0.824219213	0.192562676	1.541890165	0.40595733	0.324030158
1.406880703	0.253577738	1.072442278	0.465257077	0.404721108
0.812365006	0.153477598	1.556062491	0.337486103	0.192589373
0.581560021	0.120775523	1.149255842	0.427086441	0.315409933
1.031111251	0.110047631	1.11211039	0.287931992	0.176862489
1.053288025	0.088161953	0.925753497	0.393326692	0.120282611
0.688057004	0.067996297	1.068139443	0.276241121	0.210063877

P34998,CRHR1,CARDIOMETABOLIC	P00750,PLAT,CARDIOMETABOLIC	P00740,F9,CARDIOMETABOLIC	Q9NRD8,DUOX2,CARDIOMETABOLIC	P35443,THBS4,CARDIOMETABOLIC
P34998	P00750	P00740	Q9NRD8	P35443
CRHR1	PLAT	F9	DUOX2	THBS4
CARDIOMETABOLIC	CARDIOMETABOLIC	CARDIOMETABOLIC	CARDIOMETABOLIC	CARDIOMETABOLIC
0.62994597	0.120207599	0.329374324	0.683683257	0.987669319
0.588738957	0.170897121	0.397024516	0.82857259	1.278187957
0.600901322	0.136502568	0.292680797	0.813548353	1.674580813
0.703001659	0.107283491	0.374801855	0.410740753	0.763288309
1.408637114	0.187219161	0.373660508	0.934262586	0.792344646
1.01395948	0.121270454	0.17256359	0.542276291	1.43246511
1.083500636	0.126192668	0.471915349	0.652341809	0.574627921
0.775070796	0.144175707	0.321791919	0.485754747	1.289489477
0.731079397	0.151679708	0.335550079	0.633624489	1.110800707
0.849390572	0.08748018	0.340022	0.69011084	0.617409677
1.011292266	0.119244944	0.256387885	0.661127303	0.708971727
0.928452495	0.241969984	0.429878243	1.121010662	1.797759458
0.750695635	0.112235424	0.260399756	0.529425651	1.340248017
0.583134264	0.096789878	0.252665396	0.466322517	1.165056564
1.133983776	0.105338155	0.185900171	0.506839195	0.409007726
0.847332435	0.133018609	0.307935478	0.531521517	0.335573338
1.165702788	0.051331919	0.321948091	0.713804054	0.658839976
0.767798688	0.117237011	0.328553451	1.011642814	1.175602229
0.740565887	0.156800276	0.429104222	0.732093591	0.859458569
0.752623374	0.111359767	0.318242849	0.94160899	1.070214514
0.950988632	0.107648491	0.302498522	0.754190038	0.968953945
0.963595793	0.137986593	0.368541758	0.717375311	0.870188587
0.744374222	0.086960256	0.235728811	0.650535639	0.854883658
0.648599582	0.065661886	0.278973502	0.798685817	0.851276671
1.050298932	0.084934824	0.309090239	0.633361027	0.979556083
0.35072204	0.137556861	0.448848351	0.685391402	0.836594257
0.628724549	0.161163841	0.402176308	0.916876928	0.40598547
0.472209837	0.130815011	0.356605288	0.740976657	1.04710056
0.78736255	0.178339725	0.534810613	0.40522638	1.219846824
0.886688949	0.179717151	0.404805276	0.675206174	0.759804415
0.906073506	0.121463942	0.326057882	0.570500478	0.235467524
0.814846378	0.135128136	0.37353103	0.562061538	1.238505264
25.06495954	0.092231284	0.27814325	0.493047997	0.830642733
0.679714121	0.077245784	0.314689293	0.880198001	0.765725921
0.852280365	0.131944215	0.430564118	0.645594414	0.441290318

P35218,CA5A,CARDIOMETABOLIC	P07911,UMOD,CARDIOMETABOLIC	P23141,CES1,CARDIOMETABOLIC	Q969D9,TSLP,CARDIOMETABOLIC	O43186,CRX,CARDIOMETABOLIC
P35218	P07911	P23141	Q969D9	O43186
CA5A	UMOD	CES1	TSLP	CRX
CARDIOMETABOLIC	CARDIOMETABOLIC	CARDIOMETABOLIC	CARDIOMETABOLIC	CARDIOMETABOLIC
0.129902401	0.459870641	0.424754165	0.881113637	0.901125531
0.153286229	0.134176135	0.313426702	0.959796196	0.465805636
0.131962507	0.424312769	0.182693853	0.885521966	0.305638883
0.158790884	0.374308574	0.308020868	1.000207966	0.414717261
0.105857838	0.158307334	0.267980513	0.714744739	0.823419774
0.275629078	0.320856474	0.28813164	0.625941615	0.592751782
0.191697045	0.207977592	0.248772686	1.070140335	0.366707074
0.159751347	0.204674035	0.181935629	0.571371111	0.409773899
0.251007086	0.328986434	0.185359762	0.535144349	0.570935629
0.14520872	0.149404965	0.271439014	0.849626106	0.55179336
0.13055232	0.273592389	0.272173781	0.87496669	0.248118291
0.101862858	0.345079338	0.168054374	1.009261482	0.485081815
0.129776404	0.297879346	0.266554049	0.883988802	0.389447294
0.084599914	0.2714202	0.41549413	1.482672538	0.317801976
0.084313064	0.16617785	0.157530163	0.880869374	6.779129357
0.169164647	0.262520138	0.152406887	0.574309368	1.132962414
0.069704712	0.214864642	0.219105864	0.909408252	0.504525817
0.137090452	0.49297965	0.392264858	1.18846548	0.480963727
0.176690944	0.241149546	0.503477775	0.979080915	0.495755265
0.212332925	0.256707971	0.239899158	0.666603132	0.468590626
0.12477493	0.207675078	0.216179256	0.929225081	0.39077227
0.15519995	0.205812142	0.488218883	0.708726058	0.408752653
0.083135012	0.275533569	0.199782206	1.439731715	0.298830643
0.236416069	0.096021415	0.308940304	0.785999337	0.533514727
0.128300616	0.251686545	0.180066287	0.65071603	0.397961287
0.121961694	0.107775413	0.215296982	0.748098426	0.349629785
0.144255677	0.163390952	0.225828582	0.823876502	0.467325608
0.249221424	0.274028908	0.278451892	0.833064439	0.478768453
0.100599846	0.257956553	0.276260269	0.67604913	0.486597226
0.127017572	0.478370391	0.493253092	1.088619663	0.423196617
0.140612816	0.408441114	0.259642778	0.525622887	0.310163323
0.141679212	0.156811145	0.374905787	1.050007767	0.367699724
0.099552406	0.215939639	0.246267815	1.03011114	0.375738278
0.161981393	0.299847323	0.259211207	0.67703391	0.384351978
0.063385647	0.204546393	0.513522715	1.196897825	0.593697523

P07451,CA3,CARDIOMETABOLIC	Q13361,MFAP5,CARDIOMETABOLIC	Q99674,CGREF1,CARDIOMETABOLIC	Q13105,ZBTB17,CARDIOMETABOLIC	P15529,CD46,CARDIOMETABOLIC
P07451	Q13361	Q99674	Q13105	P15529
CA3	MFAP5	CGREF1	ZBTB17	CD46
CARDIOMETABOLIC	CARDIOMETABOLIC	CARDIOMETABOLIC	CARDIOMETABOLIC	CARDIOMETABOLIC
0.382226577	0.111436982	1.022215945	0.708529585	0.448848351
0.321524371	0.20554127	1.212596867	0.761333254	0.541412462
0.287951951	0.131734032	1.139736247	0.898007874	0.480330725
0.227635899	0.156583055	0.528655574	0.977927894	0.386355275
0.34820287	0.131023727	0.750175473	0.612720064	0.456029798
0.361583312	0.194669666	0.802236771	0.677973129	0.431460383
0.282867827	0.13288959	0.544309832	0.775124522	0.435124453
0.157268321	0.181620631	1.148141138	0.669566847	0.403796413
0.205826408	0.126543034	0.586498775	0.730876727	0.333209168
0.283908904	0.117188264	0.634987457	0.588657346	0.270668706
0.235108728	0.135983169	0.5253315	0.641535053	0.328621779
0.48169772	0.258350217	1.337927555	1.031397176	0.612720064
0.179281683	0.10137684	0.996540263	0.732245841	0.334041673
0.245245743	0.172073877	0.62772301	0.577663001	0.330769932
0.152681799	0.049700356	0.494416913	0.524676471	0.250798391
0.260363659	0.109948513	0.416301309	0.539277594	0.296643073
0.688534095	0.102777759	0.549808075	0.839673289	0.417862442
0.341770552	0.116008305	0.64478943	0.600068873	0.379165604
0.20161851	0.179654876	0.522607596	0.594191553	0.405901056
0.318264908	0.095636159	0.859816082	0.620842833	0.357347599
0.143457967	0.129686481	0.771265776	0.669474031	0.389393309
0.377042746	0.128603338	0.64478943	0.73189064	0.33263226
0.242288864	0.135343734	0.860770178	0.632439771	0.299162241
0.230989632	0.145510988	0.715190759	0.580030231	0.322372369
0.174258387	0.133851016	0.788454824	0.791521257	0.347046287
0.347335072	0.110444999	0.527703698	0.682215765	0.454105684
0.395897812	0.159596398	0.742364692	0.743600683	0.499757457
0.314079133	0.163685678	0.63860688	0.718569696	0.313187817
0.367088545	0.184756881	0.994263396	0.758331207	0.354117489
0.18622259	0.179020908	0.53056448	0.518314622	0.380930577
0.31057207	0.299431935	0.49768334	0.49134218	0.281947802
0.191524386	0.139013799	1.094217853	0.704758071	0.399869182
0.248015123	0.141179253	0.605206757	0.76731986	0.322595898
0.158548925	0.090039384	0.503791959	0.77292482	0.355963199
0.501840218	0.105615977	0.462844666	0.760805721	0.351013884

P21980,TGM2,CARDIOMETABOLIC	Q6UWL2,SUSD1,CARDIOMETABOLIC	P17516,AKR1C4,CARDIOMETABOLIC	Q96H15,TIMD4,CARDIOMETABOLIC
P21980	Q6UWL2	P17516	Q96H15
TGM2	SUSD1	AKR1C4	TIMD4
CARDIOMETABOLIC	CARDIOMETABOLIC	CARDIOMETABOLIC	CARDIOMETABOLIC
1.824877978	0.643628449	0.93770108	0.453068156
2.85561206	0.767905135	0.528216034	0.474967268
3.3603232	0.847743663	0.990892187	0.728701568
1.894871479	0.501875004	0.816655771	0.431221196
2.221755408	0.815467904	0.909912675	0.519609603
3.673104649	0.745923715	1.090432147	0.471294255
3.594522165	0.794104073	0.979352412	0.243754372
1.600031159	0.703684188	0.7801914	0.492740513
2.118143012	0.717922491	1.034977927	0.422639643
3.005281225	0.743394541	0.873088619	0.431310876
1.854204028	0.750227473	1.019032455	0.35998283
1.672724673	0.897447842	0.825419828	0.567935894
1.663359488	0.879039561	2.188587403	0.482867744
2.447824287	0.826622192	1.08259978	0.312927423
1.978353177	0.671146676	0.853817714	0.396556957
2.432938749	0.610007971	0.926202785	0.358339752
4.885817583	0.70305039	2.459730064	0.690397909
2.409777591	0.769290288	0.710053679	0.72956074
1.571671819	0.784040454	0.974004269	0.362863789
1.604139936	0.81292829	1.183615094	0.376912096
1.596264804	0.622178307	0.99529769	0.646758942
4.266934605	0.573672792	0.789275023	0.314340485
2.144438587	0.691259832	0.547867907	0.321524371
1.773623757	1.021224461	0.61839476	0.234084287
2.427211793	0.643450022	1.284850052	0.470902406
4.788586956	0.824276346	1.026120386	0.65788166
3.026184609	0.79487505	0.811802113	0.321346129
7.959070746	0.784801661	1.379074122	0.423871831
4.999209546	0.842413231	1.060761265	0.443129415
1.927859615	0.681034599	1.335518536	0.607055363
2.469809896	0.613229922	0.758331207	0.535998177
2.830976945	0.80446413	0.762759422	0.55064713
2.02300516	0.648105238	0.634635443	0.343718641
1.872025387	0.737952571	1.578550046	0.48310209
8.744646107	0.616383437	1.763326951	0.661402315

Q9UM47,NOTCH3,CARDIOMETABOLIC	P08236,GUSB,CARDIOMETABOLIC	P12830,CDH1,CARDIOMETABOLIC	O15354,GPR37,CARDIOMETABOLIC	P31483,TIA1,CARDIOMETABOLIC
Q9UM47	P08236	P12830	O15354	P31483
NOTCH3	GUSB	CDH1	GPR37	TIA1
CARDIOMETABOLIC	CARDIOMETABOLIC	CARDIOMETABOLIC	CARDIOMETABOLIC	CARDIOMETABOLIC
0.417254641	0.390149782	0.310550544	0.368031205	0.986916546
0.405114043	0.288951647	0.359683529	0.50754231	0.964665048
0.462876749	0.586946128	0.387266872	0.25815331	0.768171317
0.334853047	0.252070641	0.315497395	0.228252094	0.879222371
0.344052349	0.371259539	0.257206677	0.400118711	1.03950738
0.391233008	0.304856033	0.36128268	0.427234483	0.762336575
0.413769727	0.299556491	0.331091069	0.270162624	1.027900059
0.45040664	0.271721382	0.358886609	0.246523998	0.902062938
0.288111669	0.180804188	0.249515268	0.218181397	1.410591254
0.230621672	0.225922521	0.234068062	0.254352292	1.112418776
0.257385021	0.267869086	0.321992725	0.283633531	0.840197268
0.734737085	0.31914855	0.427975466	0.559185943	0.925047915
0.406774177	0.642781361	0.266701899	0.273061912	0.944419673
0.337299013	0.347046287	0.293269715	0.251459855	1.072367945
0.244855074	0.148157134	0.209076092	0.221073806	1.227225189
0.238622171	0.165901634	0.192762991	0.185642637	0.96346222
0.436514051	0.254158432	0.316263726	0.183798895	1.044635763
0.359808207	0.201311291	0.232048777	0.319015848	0.947041794
0.389420301	0.601776636	0.226235934	0.214864642	1.067399322
0.381723524	0.179879165	0.257688489	0.307402329	0.925753497
0.361984543	0.289212137	0.26186588	0.142664669	1.058484395
0.290981634	0.283103208	0.294736982	0.221027839	0.798630458
0.380218333	0.251459855	0.260490019	0.287592907	0.730826068
0.33143549	0.493868891	0.230126655	0.539128096	1.32106367
0.404328555	0.36846513	0.262775012	0.212701189	0.689871707
0.341391726	0.198099892	0.283771184	0.125460055	0.833295446
0.35623471	0.290679251	0.319945926	0.228854093	0.970096385
0.277738677	0.666787979	0.262410981	0.193365189	1.017761836
0.380772185	0.174548516	0.379323327	0.337252256	0.995780728
0.240999156	0.245688119	0.387266872	0.212068169	1.107878753
0.252700426	0.157202929	0.353406382	0.420156876	0.896142459
0.371208075	0.346757742	0.345103258	0.25566029	0.846510577
0.254528657	0.333001366	0.267999089	0.184373089	1.315489743
0.271608399	0.171217254	0.242793213	0.238754528	0.810789887
0.32261826	0.228521213	0.290336931	0.292234822	0.971644183

O75326,SEMA7A,CARDIOMETABOLIC	Q07654,TFF3,CARDIOMETABOLIC	P09619,PDGFRB,CARDIOMETABOLIC	Q12884,FAP,CARDIOMETABOLIC	P18428,LBP,CARDIOMETABOLIC
O75326	Q07654	P09619	Q12884	P18428
SEMA7A	TFF3	PDGFRB	FAP	LBP
CARDIOMETABOLIC	CARDIOMETABOLIC	CARDIOMETABOLIC	CARDIOMETABOLIC	CARDIOMETABOLIC
0.525987347	0.528765517	0.631738762	1.0238469	0.335201381
0.427234483	0.485990494	0.584591193	0.991716731	0.384885173
0.406520497	0.51419946	0.830354904	1.173648178	0.269844465
0.365185149	0.424960307	0.557599047	0.86862176	0.564912763
0.566559743	0.482265663	0.613612595	0.986642952	0.274827828
0.372264513	0.466193243	0.381882311	0.933550518	0.186661979
0.464515937	0.499134316	0.361508131	0.805915229	0.163799175
0.425785876	0.493595108	0.364856232	0.958599438	0.181343885
0.283830198	0.369846877	0.332056352	0.683209528	0.376677039
0.308298548	0.25201823	0.514805724	0.701055225	0.250537767
0.331757273	0.439824538	0.551067137	0.8362464	0.127167331
0.496511831	0.68060988	1.051974694	1.821971005	0.490355512
0.302960161	0.377958568	0.627810037	1.157089131	0.094817686
0.327484834	0.387535398	0.486327474	0.845162113	0.121548163
0.308191718	0.311089154	0.549922416	0.658885645	0.179132622
0.38209413	0.370026372	0.43419048	0.567188427	0.301911999
0.394446079	0.501006074	0.547146851	0.805133543	0.314580249
0.379954877	0.543405096	0.56848729	0.929611615	0.261412494
0.424106941	0.410086456	0.636838738	0.94828985	0.206626901
0.37618129	0.434491543	0.498235595	0.954687186	0.191166284
0.309325998	0.383979182	0.439641658	0.927809163	0.368082228
0.303717091	0.486361185	0.309969893	0.953959551	0.354780839
0.271759053	0.548856155	0.443344474	0.933550518	0.201954192
0.288631367	0.363266441	0.567935894	0.76164995	0.198333461
0.433859553	0.463743832	0.394719583	1.061202515	0.160884807
0.353259435	0.587719634	0.369872514	0.837058292	0.32139068
0.384458558	0.452660084	0.544536251	0.67633035	0.269171953
0.251181132	0.417978315	0.789439165	1.014381261	0.225359473
0.474835597	0.547033086	0.3898254	0.95210989	0.287254221
0.220354765	0.489642265	0.455871777	0.626896856	0.283378066
0.327257917	0.434491543	0.631038529	0.489845944	0.315060326
0.49520576	0.506733811	0.389420301	1.037060457	0.21697489
0.285448028	0.540962315	0.45081268	0.78154454	0.240681974
0.298851357	0.372910158	0.414976056	0.811745845	0.162622637
0.33844657	0.469566047	0.610304021	0.830700311	0.625247808

O75594,PGLYRP1,CARDIOMETABOLIC	P07478,PRSS2,CARDIOMETABOLIC	P31997,CEACAM8,CARDIOMETABOLIC	Q12794,HYAL1,CARDIOMETABOLIC	Q9UBR2,CTSZ,CARDIOMETABOLIC
O75594	P07478	P31997	Q12794	Q9UBR2
PGLYRP1	PRSS2	CEACAM8	HYAL1	CTSZ
CARDIOMETABOLIC	CARDIOMETABOLIC	CARDIOMETABOLIC	CARDIOMETABOLIC	CARDIOMETABOLIC
0.321346129	0.304243849	0.389528286	0.459201736	0.543744195
0.409631907	0.287592907	0.379323327	0.447481517	0.556247943
0.447574578	0.307508885	0.725073926	0.44714046	0.436604831
0.298395979	0.316768328	0.168205875	0.43569788	0.540737382
0.623300597	0.329991324	0.496752798	0.47880164	0.455524325
0.315847487	0.289031773	0.377487297	0.39280903	0.422317519
0.534477085	0.347238784	0.355273011	0.482934689	0.368184296
0.567345706	0.295965311	0.400146446	0.38947429	0.47883483
0.488286569	0.236629197	0.313535347	0.438728399	0.351452105
0.287015389	0.134231949	0.431221196	0.359907981	0.329397155
0.333833352	0.249083265	0.456947397	0.321635822	0.523187508
0.441994401	0.394145443	0.633009914	0.568093381	0.613867842
0.274999328	0.243788166	0.410114882	0.357967372	0.451250364
0.210267823	0.363593925	0.179729608	0.346637586	0.385713087
0.30120132	0.150277404	0.31910431	0.315847487	0.323043421
0.726029463	0.30648747	0.587271691	0.297755487	0.310077339
0.452158346	0.278316819	0.387777231	0.501979377	0.455398044
0.316746372	0.313296379	0.272702532	0.453445164	0.388207528
0.493492478	0.220370039	0.53950192	0.372755101	0.484745698
0.472864912	0.275476279	0.608909615	0.44288376	0.257938673
0.259193241	0.210953949	0.379086767	0.342007531	0.470673978
0.283456646	0.280330379	0.362612358	0.39172144	0.523913303
0.337977708	0.235125025	0.377434969	0.308469552	0.369744348
0.319635599	0.256245753	0.513629511	0.390420307	0.325651325
0.319037961	0.209889223	0.398099234	0.360982299	0.382809888
0.38483182	0.244566719	0.266664928	0.49875389	0.365691754
0.38286296	0.304856033	0.486024181	0.476550167	0.402232065
0.461403227	0.244719336	0.569512732	0.461883207	0.444082615
0.668268603	0.292924344	0.610388633	0.544234379	0.490083677
0.311089154	0.23023834	0.291203581	0.422639643	0.478569381
0.337579687	0.274675474	0.211305174	0.406295136	0.366325999
0.232097035	0.325877127	0.294267476	0.434732543	0.392047401
0.285329338	0.271514283	0.334365986	0.361909278	0.346085403
0.258135417	0.310163323	0.218090676	0.320012464	0.343790122
0.466096311	0.261086542	0.5121009	0.512029913	0.618351898

P43121,MCAM,CARDIOMETABOLIC	Q01638,IL1RL1,CARDIOMETABOLIC	Q12912,LRMP,CARDIOMETABOLIC	Q96N03,VSTM2L,CARDIOMETABOLIC	Q8N423,LILRB2,CARDIOMETABOLIC
P43121	Q01638	Q12912	Q96N03	Q8N423
MCAM	IL1RL1	LRMP	VSTM2L	LILRB2
CARDIOMETABOLIC	CARDIOMETABOLIC	CARDIOMETABOLIC	CARDIOMETABOLIC	CARDIOMETABOLIC
0.446026088	0.217607472	0.439824538	0.671518942	0.504281079
0.455303356	0.58422662	0.573434257	0.501527252	0.469631147
0.355642588	0.30513086	0.956740781	0.87502734	0.580714113
0.384751805	0.243281749	0.514520334	0.552980304	0.259822811
0.510258424	0.288191562	0.856781955	0.811520812	0.594273931
0.368695062	0.294328673	0.703684188	0.792124991	0.530748392
0.38667677	0.536183972	0.800237421	0.645594414	0.277046491
0.311952873	0.385205445	0.72733908	0.681979368	0.412509715
0.328576225	0.205313443	0.444945333	0.627418511	0.497372966
0.225031676	0.291344908	0.547791961	0.645370706	0.376912096
0.413196517	0.225734682	0.674785089	0.60869862	0.335759471
0.492740513	0.287174589	0.758962232	0.650084878	0.363442742
0.333093706	0.239982315	0.764347185	0.631038529	0.364881522
0.298127218	0.179617522	1.00235948	0.578905593	0.294757413
0.264841296	0.203429386	0.351232926	0.48169772	0.207516794
0.295883264	0.20974379	0.615273602	0.418152183	0.370822323
0.492023797	0.204972178	0.764347185	0.666834199	0.577582926
0.367674238	0.195291356	0.548589913	0.592423182	0.362587225
0.433288545	0.414976056	0.673150035	0.592751782	0.531263683
0.32983125	0.171407246	0.825362616	0.714199981	0.390636862
0.336738364	0.129857388	0.818696141	0.736113431	0.141120551
0.36645298	0.212951973	0.625768091	0.550380019	0.340139863
0.313383255	0.172217064	0.458216082	0.483906425	0.42637655
0.284342174	0.146523096	0.528802169	0.887426783	0.311801549
0.32050083	0.292741665	0.509763506	0.26067064	0.517776
0.38844978	0.343909292	0.447264451	0.502362263	0.456440907
0.382518121	0.658018477	0.77819305	0.744838732	0.410598425
0.325673898	0.200100961	0.517560708	0.25535921	0.437422701
0.339362721	0.292863438	0.734024438	0.566324167	0.420186
0.450000965	0.286756879	0.713062283	0.698823486	0.421498675
0.280000246	0.21793956	0.490389502	0.531337337	0.470869767
0.385232146	0.243991027	0.466031701	0.521811267	0.421878655
0.302414664	0.231807637	0.705295627	0.66840758	0.331251754
0.30063815	0.167206171	0.543066208	0.825191005	0.336645013
0.282613052	0.157923744	0.65469733	0.660074148	0.533034198

P04792,HSPB1,CARDIOMETABOLIC	P20711,DDC,CARDIOMETABOLIC	Q99523,SORT1,CARDIOMETABOLIC	O95684,FGFR1OP,CARDIOMETABOLIC	P00533,EGFR,CARDIOMETABOLIC
P04792	P20711	Q99523	O95684	P00533
HSPB1	DDC	SORT1	FGFR1OP	EGFR
CARDIOMETABOLIC	CARDIOMETABOLIC	CARDIOMETABOLIC	CARDIOMETABOLIC	CARDIOMETABOLIC
0.903564816	0.2379285	0.27559087	0.765195344	0.500520131
0.126376489	0.335062004	0.45429458	0.939913567	0.404328555
0.467811749	0.450437861	0.325448236	0.709610863	0.441351498
0.583659958	0.376103073	0.287134781	0.751580739	0.444144183
0.79526082	0.379402213	0.410940094	1.158292806	0.519285555
0.480430617	0.460317118	0.233808616	0.665356752	0.504246126
0.658018477	0.28138162	0.282123745	0.770998522	0.448506252
0.13918735	0.272286998	0.252665396	0.710496772	0.341889021
0.420506498	0.216329152	0.291486303	1.511729484	0.442454191
0.467131293	0.176055231	0.265006564	0.665679664	0.33631849
0.148249588	0.380693014	0.171027473	0.699792933	0.397740672
0.906324758	0.343361455	0.45565064	1.061423209	0.550990748
0.175348865	0.149840549	0.223756268	0.72502367	0.371182346
0.326170905	0.16007279	0.193606595	0.976234742	0.315541136
0.175239511	0.187387939	0.230621672	0.892361391	0.302897169
0.299535728	0.239201775	0.281596245	0.876909582	0.342648197
0.996747509	0.462491899	0.633536656	0.641046093	0.484208396
0.188390745	0.50768305	0.267757706	0.945598728	0.467098915
0.657516954	0.242658617	0.31837523	1.234305914	0.440862295
0.238837288	0.241668275	0.290739703	0.840139032	0.412795744
0.515734335	0.314972985	0.282006437	1.075867193	0.279263707
0.774587433	0.22147258	0.297796768	1.104964485	0.407903559
0.31024933	0.368490671	0.257563488	0.574627921	0.386837618
0.144025882	0.162679008	0.283633531	1.142583816	0.37288431
0.210428206	0.535552532	0.259912874	0.913894794	0.333278464
0.113392726	0.322819582	0.349896466	0.694188803	0.460317118
0.373298082	0.271476646	0.372548459	1.089978743	0.435395982
0.349290667	0.308726237	0.272419145	0.88785747	0.411510173
0.383846128	0.378850354	0.315147692	0.755812351	0.43283828
0.445130419	0.180241109	0.293046193	1.16384586	0.347551819
0.389123496	0.216239202	0.226801172	0.95323247	0.385125352
0.192816444	0.36938572	0.327870953	0.806362247	0.412481123
0.271439014	0.39829244	0.249083265	0.589433107	0.325222731
0.192229279	0.187882161	0.262101951	0.366351391	0.362059823
1.706215648	0.274085897	0.372032355	1.032398535	0.424371595

Q9NRV9,HEBP1,CARDIOMETABOLIC	P05121,SERPINE1,CARDIOMETABOLIC	Q7Z739,YTHDF3,INFLAMMATION	P01583,IL1A,INFLAMMATION	Q9UHX3,ADGRE2,INFLAMMATION
Q9NRV9	P05121	Q7Z739	P01583	Q9UHX3
HEBP1	SERPINE1	YTHDF3	IL1A	ADGRE2
CARDIOMETABOLIC	CARDIOMETABOLIC	INFLAMMATION	INFLAMMATION	INFLAMMATION
0.663652535	0.112640691	0.762653688	0.826908727	0.378876615
0.903752727	0.159033213	0.718171347	0.743343015	0.39001459
0.866997657	0.076595317	0.437271128	2.756237103	0.429848447
0.658383461	0.040193332	1.614961755	1.207899145	0.431819411
1.110107969	0.072770624	1.29145735	1.210665238	0.634943445
1.033257618	0.062008079	1.108339602	1.003680436	0.486361185
0.681459583	0.083014088	0.73989887	0.64698313	0.470641355
1.176906737	0.109644093	1.340898467	0.663146718	0.422405346
0.936791571	0.309240247	1.299989526	0.875452009	0.483437067
0.951648035	0.325877127	0.705197859	2.118583513	0.292315858
0.671425856	0.092943643	0.639226888	0.563036361	0.255058485
0.894404902	0.238986329	0.830412462	1.011292266	0.51652139
1.547886834	0.063985996	1.147902414	5.712807827	0.271627226
1.119224928	0.056195562	0.588943034	0.902187999	0.253947117
0.737850276	0.104480094	1.751025386	1.014521894	0.271401387
0.58406466	0.183607895	0.375868521	0.833468742	0.328530678
1.32656926	1.202719321	0.963261894	1.03634187	0.532258873
1.311756556	0.166569943	1.938445266	3.640659964	0.423372656
0.648689504	0.102053672	1.146391645	1.061643949	0.345007589
0.671239723	0.05109054	0.363770385	0.74649267	0.438394013
1.263652691	0.070423464	0.519429552	0.952571968	0.449439865
0.865796576	0.19145802	0.795205698	0.882091365	0.325425679
0.812590273	0.055444955	0.966070245	0.662228038	0.343361455
1.501807602	0.029147178	0.740360587	1.209742504	0.270499906
0.640646313	0.036701937	1.219255094	0.80179204	0.35858822
0.551678629	0.198017522	0.444267343	0.835087919	0.331688293
1.497960934	0.055157466	1.180992661	0.942523177	0.332955205
0.756546152	0.499411172	0.609162906	1.133119486	0.275457185
1.088619663	0.211481006	0.680940194	0.839556894	0.555785461
1.025480458	0.176813459	1.011081996	0.924278801	0.351768938
0.603280142	0.168147589	1.350131566	0.678866594	0.242003531
1.073706736	0.15621447	1.173729532	0.824504916	0.394555457
0.794985251	0.167670408	0.652613167	0.931675848	0.284795844
1.279340241	0.051872007	1.300620438	3.38674649	0.350649117
0.99151053	0.109272328	0.437392382	0.748720935	0.371491215

Q16552,IL17A,INFLAMMATION	P01579,IFNG,INFLAMMATION	O00626,CCL22,INFLAMMATION	Q05084,ICA1,INFLAMMATION	P19878,NCF2,INFLAMMATION
Q16552	P01579	O00626	Q05084	P19878
IL17A	IFNG	CCL22	ICA1	NCF2
INFLAMMATION	INFLAMMATION	INFLAMMATION	INFLAMMATION	INFLAMMATION
0.664342907	0.155727971	0.229505399	0.91376811	0.239965682
0.536630143	0.373505139	0.788564134	1.032541666	0.185013186
0.758015892	0.559690048	0.696116174	1.131236049	0.301055212
0.786871521	2.149945368	0.409688698	0.9961259	0.305808413
0.612720064	6.575041498	0.588249462	0.677785181	0.399010883
0.814056029	0.42889607	0.308811846	0.772817678	0.282299798
0.753040832	0.011621177	0.375217755	0.644253333	0.197113706
0.571767293	0.007568903	0.689107039	0.668963777	0.153839726
0.448692819	0.246643641	0.715042054	0.969693017	0.271815569
0.67858432	0.42443043	0.600276877	0.60336378	0.192069454
0.666418336	1.705387988	0.175373176	1.174950518	0.286379474
0.524240239	3.596266662	0.575106082	0.83133393	0.302226066
0.743600683	0.497269551	0.190346507	0.97258753	0.26201113
0.742673497	0.137881423	0.412624102	0.841362837	0.221196429
0.729105758	0.216839577	0.262410981	0.547943862	0.164550236
0.521413557	0.100544077	0.581116772	0.760542092	0.46274843
0.709020871	0.489879899	0.380904173	1.217566019	0.348468463
0.803405365	0.156572202	0.392346436	0.835493205	0.217803645
0.647745952	1.093914514	0.548399819	0.385793302	0.342434508
0.55953489	0.455334917	0.369641848	0.798021765	0.259013644
0.64967946	1.640914421	0.471490301	0.729105758	0.245500863
0.457708186	0.829089641	0.288811481	0.914655267	0.429908041
0.446026088	0.469175635	0.486664687	0.958732338	0.27804687
0.763976412	0.260435857	0.22945768	0.369001862	0.255341511
0.656697105	0.564599595	0.303044171	0.906387582	0.255270725
0.617923437	0.675065783	0.323536414	0.748409616	0.205398849
0.564286602	0.546123824	0.565186927	0.909534331	0.241802321
0.561127296	0.723216752	0.41612821	0.635251596	0.338610825
0.831276308	1.137211032	0.274275944	0.932645035	0.288871544
0.641935388	0.133684119	0.498995946	0.872423175	0.233484713
0.621144141	0.413683695	0.211833109	0.655060472	0.208280546
0.623041428	0.286498601	0.281128184	0.359409388	0.170849745
0.73153561	1.176498923	0.387750353	0.812759264	0.175239511
0.661356472	0.428658305	0.227462402	0.685296394	0.216749414
0.752727717		0.478469875	0.897634481	0.688629552

P40933,IL15,INFLAMMATION	P05113,IL5,INFLAMMATION	Q9NQ30,ESM1,INFLAMMATION	Q96DB9,FXYD5,INFLAMMATION	O43598,DNPH1,INFLAMMATION
P40933	P05113	Q9NQ30	Q96DB9	O43598
IL15	IL5	ESM1	FXYD5	DNPH1
INFLAMMATION	INFLAMMATION	INFLAMMATION	INFLAMMATION	INFLAMMATION
0.336295178	0.004594106	1.428597993	1.389821881	1.265317992
0.266295508	0.001982175	1.340991414	1.012554807	0.961127679
0.343980813	0.006668139	1.777192552	1.437537907	1.102210671
0.47355372	0.01660199	1.412254404	1.445932295	0.744477421
0.265227082	0.005583943	1.073037131	1.335703691	0.839324152
0.320656376	0.004745908	1.410200209	1.587877921	0.839964348
0.302498522	0.021254321	1.280138584	0.79526082	0.798907291
0.32483973	0.002501657	1.538367298	1.257187646	0.581036218
0.266868328	0.003163857	1.95152237	1.359899465	0.944485138
0.227714805	0.005131157	1.107955548	2.032844694	1.191434797
0.278567721	0.001737827	0.82262111	1.237732885	0.694910941
0.415263795	0.003400824	2.656082576	1.300530289	2.116528624
0.320434191	0.007502567	0.940956543	1.257797785	0.610430943
0.217200601	0.004654687	0.681459583	1.134612765	0.929547182
0.197455574	0.004311782	0.738720234	1.32840955	0.970298132
0.193633436	0.004996384	0.807201075	1.49557473	1.500142968
0.322238326	0.003394465	1.453065765	2.019922583	2.893669191
0.403376796	0.002993606	1.002081605	1.726080799	0.869585627
0.361007321	0.014522161	0.966740106	1.182303151	0.6265059
0.297075184	0.004749199	1.091112606	0.86585659	0.718320702
0.288491357	0.003743491	1.292532001	0.69260274	0.608403349
0.432568345	0.003635577	1.388666341	1.017056622	1.641028165
0.268724544	0.004537143	1.505246747	1.181811547	0.817561973
0.261648157	0.002239201	0.810340415	1.030753957	0.777761647
0.302729254	0.01099659	1.510263204	1.157089131	0.795150581
0.264382758	0.003611215	1.53485248	1.128416761	1.352848231
0.306147753	0.001602122	1.256839128	1.450851639	1.207480591
0.22521893	0.004318662	0.779542724	1.597593092	2.352834754
0.285725163	0.007366525	1.390978384	1.25840822	1.588978936
0.297838054	0.009979616	1.047608739	1.35162974	0.829319546
0.257367181	0.004743606	0.888288365	1.067695309	0.840546769
0.298230559	0.005595179	1.072070662	0.831737394	0.943569048
0.184718466	0.002573237	0.767107143	0.880442077	0.873451801
0.284105763	0.011899131	0.591602478	1.281381441	0.763923459
0.351549561	0.002807302	0.994608041	0.614889894	2.868506923

P19801,AOC1,INFLAMMATION	O75077,ADAM23,INFLAMMATION	Q13478,IL18R1,INFLAMMATION	P46109,CRKL,INFLAMMATION	O75888,TNFSF13,INFLAMMATION
P19801	O75077	Q13478	P46109	O75888
AOC1	ADAM23	IL18R1	CRKL	TNFSF13
INFLAMMATION	INFLAMMATION	INFLAMMATION	INFLAMMATION	INFLAMMATION
0.308319918	0.467228441	0.331849269	0.63630925	0.367190337
0.208989157	0.324142478	0.563075389	0.564873607	0.326691313
0.393217654	0.532738703	0.494965543	0.560544184	0.447419488
0.292843139	0.452283729	0.429223211	0.522317881	0.478768453
0.350892253	0.408186394	0.660119902	0.56750303	0.383739718
0.346157377	0.498857614	0.345797657	0.644610682	0.344672954
0.287413553	0.303296341	0.372238711	0.547564188	0.408497739
0.240115427	0.393845037	0.388422856	0.486125258	0.368082228
0.286637645	0.306763768	0.437817037	0.494794031	0.332586151
0.267220019	0.248221502	0.293676555	0.462652214	0.312472255
0.258601043	0.319945926	0.255447726	0.442454191	0.313644028
0.241802321	0.752310432	0.444729497	0.849213964	0.480897056
0.367572311	0.307508885	0.366402182	0.429937841	0.337486103
0.246216611	0.481664332	0.361533189	0.531411001	0.297590422
0.350479022	0.182200649	0.203457589	0.367929179	0.255376911
0.328121037	0.173619392	0.261902185	0.620627703	0.255465433
0.237516559	0.424577552	0.322417063	1.074972684	0.47440792
0.508140725	0.541938107	0.319369844	0.486057871	0.346950079
0.2967459	0.524276578	0.428153493	0.58743454	0.407818747
0.251634214	0.319812892	0.319591291	0.446273486	0.381247558
0.224175419	0.333671414	0.308597868	0.395486403	0.295616766
0.332516999	0.2731187	0.464161896	0.655060472	0.394746943
0.345222883	0.271928638	0.308790441	0.586336186	0.368695062
0.243264887	0.307594156	0.283181711	0.332816762	0.288871544
0.230493824	0.528912142	0.236334147	0.414976056	0.366199062
0.244481974	0.318154625	0.392074576	0.552750373	0.321346129
0.350284729	0.259175275	0.35060051	0.621574834	0.359907981
0.225562634	0.314035595	0.490695518	0.887488297	0.293961679
0.334412342	0.560155779	0.40666141	0.614847274	0.354657903
0.78138204	0.288491357	0.240581898	0.478171483	0.30063815
0.245160762	0.339550956	0.340446498	0.501562016	0.293900557
0.214819967	0.412566904	0.393081399	0.496890547	0.325877127
0.340092713	0.383022222	0.248893421	0.504840655	0.283849873
0.333393989	0.381935255	0.454578073	0.387427965	0.26999414
0.184373089	0.314732922	0.387427965	1.008003047	0.443928735

P01730,CD4,INFLAMMATION	P19474,TRIM21,INFLAMMATION	Q7L8A9,VASH1,INFLAMMATION	P22301,IL10,INFLAMMATION	O95715,CXCL14,INFLAMMATION
P01730	P19474	Q7L8A9	P22301	O95715
CD4	TRIM21	VASH1	IL10	CXCL14
INFLAMMATION	INFLAMMATION	INFLAMMATION	INFLAMMATION	INFLAMMATION
0.388261349	0.921975296	1.115739322	0.002215273	0.245041838
0.482800809	0.705491204	1.043550198	0.003881688	0.251303035
0.513665114	0.811127156	1.376972741	0.0064245	0.218272155
0.415897524	0.681601304	1.233963739	0.003861026	0.393190399
0.766575609	1.463476926	1.186325577	0.00493717	0.355963199
0.59106963	0.912122816	1.499103508	0.001614832	0.234929534
0.407338476	0.527850029	0.981050971	0.001562206	0.217321076
0.385258849	0.741696055	1.03469101	0.003195373	0.195535166
0.312017748	0.476385035	1.019385686	0.003601466	0.615444215
0.688629552	0.840372	1.33062127	0.003422343	0.186312968
0.341841628	0.697323497	1.270150983	0.003456673	0.18922833
0.515305537	1.167077203	0.821652345	0.008971718	0.246028951
0.400118711	0.705344516	1.855746953	0.002033245	0.223647727
0.271119352	0.400174183	0.982003451	0.002619124	0.242725906
0.25278802	0.856960136	1.1845179	0.001751249	0.218574954
0.26986317	0.755026926	0.930578654	0.000852803	0.372419366
0.599071456	0.859518144	1.245651071	0.005338684	0.219470661
0.688772764	0.795371074	1.359899465	0.002959563	0.284184544
0.395349362	0.748928554	0.951120476	0.00352466	0.235434883
0.404973665	0.818809644	1.182631	0.000791458	0.161007523
0.340210601	0.800958833	1.064296408	0.003626013	0.329328666
0.365514362	0.820969198	1.146948012	0.006522768	0.198870336
0.32653284	0.633361027	1.127009753	0.003262062	0.192122714
0.309175949	0.466710555	1.002984979	0.002991739	0.259193241
0.442116965	0.666556928	1.040444493	0.002588801	0.220263141
0.33743932	0.579066122	1.056505297	0.001698763	0.20285208
0.55632506	0.530343871	1.181647724	0.002885626	0.314602055
0.279515463	1.01832636	0.978470323	0.002736979	0.344028502
0.38286296	0.792454496	1.114888938	0.002103635	0.239982315
0.366097544	0.469826502	1.209742504	0.001960042	0.272079469
0.353210467	0.479565573	0.884172642	0.002912553	0.210034758
0.376363858	0.644119378	1.09414201	0.005415461	0.215655438
0.243197449	0.563778357	0.982752477	0.003134172	0.179704694
0.337626488	0.651528413	1.366419056	0.001908295	0.25458159
0.397492625	0.857613785	0.983706609	0.002279764	0.259319032

Q03405,PLAUR,INFLAMMATION	P24394,IL4R,INFLAMMATION	Q96LC7,SIGLEC10,INFLAMMATION	O95750,FGF19,INFLAMMATION	Q8N6P7,IL22RA1,INFLAMMATION
Q03405	P24394	Q96LC7	O95750	Q8N6P7
PLAUR	IL4R	SIGLEC10	FGF19	IL22RA1
INFLAMMATION	INFLAMMATION	INFLAMMATION	INFLAMMATION	INFLAMMATION
0.409972771	0.536332655	0.561516375	0.076362069	0.951845945
0.354780839	0.498546508	0.748461493	0.376807608	0.788072356
0.451250364	0.659525342	0.871335362	0.614676826	0.631563631
0.357248535	0.445222991	0.669242049	0.513736328	0.938611473
0.393735855	0.571450326	0.805747661	0.14809553	1.407270827
0.415782228	0.495171436	0.489201252	0.177710398	0.592628536
0.352965725	0.49393736	0.5069446	0.167589074	0.772175133
0.32653284	0.481430684	0.658931317	0.165064296	0.65071603
0.377565801	0.351476466	0.427441829	0.127679605	2.069668918
0.314842019	0.313470155	0.565343651	0.13131467	0.794104073
0.298189218	0.378115789	0.432628316	0.191378411	0.667435348
0.585118201	0.830067175	0.648374834	0.317515736	0.638031696
0.310615128	0.421323415	0.667065346	0.114561961	0.692458732
0.271138145	0.413196517	0.33708866	0.187439901	0.550952558
0.260129153	0.272702532	0.372522637	0.095636159	0.837870973
0.288211539	0.240215309	0.50145773	0.255624851	0.629596751
0.365159837	0.39172144	0.45751787	0.132191379	0.818923163
0.381353276	0.395102807	0.57990963	0.406745982	0.890631165
0.326261351	0.390826446	1.377163643	0.185539723	0.829722032
0.346781779	0.448972815	0.634107788	0.590373553	0.672683604
0.352696704	0.303885554	0.45394833	0.104010422	0.669798941
0.347214716	0.40906443	0.564482202	0.183467954	0.895831934
0.35323495	0.34642141	0.470804495	0.226439885	0.569710144
0.237072463	0.366580005	0.378692827	0.233306758	0.697033548
0.398485739	0.588127151	0.604075173	0.211774384	0.526315577
0.335782745	0.394418739	0.432898288	0.115952031	0.515591362
0.294389883	0.444298138	0.656424049	0.41879032	0.945402117
0.35294126	0.287892079	0.429491058	0.119592598	1.133355136
0.41529258	0.513985655	0.564443077	0.069767551	0.657516954
0.34006914	0.379060491	0.474605262	0.168135935	0.870128272
0.238341156	0.368950711	0.407677432	0.153477598	0.838103312
0.339386245	0.510010905	0.629378587	0.082081448	0.933679945
0.290518109	0.359160351	0.484611317	0.268240689	0.640291161
0.351208581	0.424253951	0.523223774	0.197578792	0.783225695
0.333024449	0.327552939	0.535404066	0.262975445	0.699695928

P01133,EGF,INFLAMMATION	O60575,SPINK4,INFLAMMATION	P11274,BCR,INFLAMMATION	O76038,SCGN,INFLAMMATION	Q9UPV0,CEP164,INFLAMMATION
P01133	O60575	P11274	O76038	Q9UPV0
EGF	SPINK4	BCR	SCGN	CEP164
INFLAMMATION	INFLAMMATION	INFLAMMATION	INFLAMMATION	INFLAMMATION
0.311002913	0.45031299	0.939978719	0.542727531	0.819150248
0.153562728	0.620627703	0.745200217	0.578023479	0.740206649
0.208006425	0.753093031	0.87448164	0.894033007	0.701298234
0.14033045	0.605164809	0.826966046	0.78024548	0.844752137
0.201492773	1.054456807	0.864717025	0.713062283	0.804854553
0.169117751	0.954488685	1.185750108	0.785236968	0.634327591
0.19548096	0.707155796	0.891434066	0.709955252	0.890075733
0.124965347	0.603572927	1.027330228	0.697371833	0.565304466
0.180666384	0.450000965	0.772121612	0.624424911	0.831045862
0.46577335	0.509586866	0.75126823	0.670914114	0.883559992
0.133480417	0.671751713	0.718519891	0.935493802	0.631126016
0.25417605	0.850038447	1.007863318	0.537300096	0.586417475
0.198470983	0.65492427	1.087413015	0.3747499	0.66494181
0.167577458	0.425756364	0.747994724	0.449533333	0.776845711
0.170790543	0.494416913	0.909030118	0.577783136	0.752206146
0.242204907	0.425431865	1.156688184	0.751372385	0.568881472
1.229694548	0.556749399	1.019456347	0.843698827	0.977995682
0.184769688	0.859875682	0.880381052	0.913704775	2.844549132
0.165821157	0.422200444	0.644834125	0.901312935	1.006536856
0.13956412	0.49806295	1.060761265	0.544121221	0.497890364
0.118085195	0.673150035	0.737134609	0.501666324	0.455808584
0.236744038	0.662273942	1.228416674	0.640069292	0.725828193
0.143080602	0.782411782	0.896204577	0.590823862	0.852575794
0.111514251	0.829664522	1.002845945	0.479200062	0.610134832
0.126341455	0.522281678	0.817505306	0.829147112	0.605962323
0.204390493	0.483537606	0.831968032	0.613867842	0.662228038
0.15063198	0.570381858	0.973194451	0.786816981	1.061128961
0.387052185	0.479731807	1.076986376	0.830930662	0.95013209
0.252000762	0.417312488	1.147981983	0.796640096	0.866216765
0.15859289	0.39096192	1.114657127	0.801458653	0.5488942
0.198347209	0.673570098	0.768171317	0.552061156	0.689632658
0.178909264	0.440984545	0.844459419	0.648329893	0.708185888
0.199616101	0.477078971	0.724923167	0.689107039	0.44500702
0.175300255	0.613655128	0.903502188	1.0856808	0.73805488
0.22976007	0.461275317	1.290025872	0.633756261	0.51505557

Q9BY76,ANGPTL4,INFLAMMATION	O43915,VEGFD,INFLAMMATION	P50452,SERPINB8,INFLAMMATION	Q16651,PRSS8,INFLAMMATION	O00253,AGRP,INFLAMMATION
Q9BY76	O43915	P50452	Q16651	O00253
ANGPTL4	VEGFD	SERPINB8	PRSS8	AGRP
INFLAMMATION	INFLAMMATION	INFLAMMATION	INFLAMMATION	INFLAMMATION
0.375764323	0.480930391	0.46885054	0.336132047	0.129542734
0.23346853	0.330586565	0.618480494	0.361708649	0.461947242
0.309690708	0.384965216	0.380271046	0.280349811	0.325042439
0.19549451	0.324794701	0.511568735	0.140340178	0.429252964
0.285092106	0.255058485	0.731586318	0.219105864	0.634943445
0.337649892	0.611447273	0.396172323	0.264401085	0.530454164
0.272986214	0.3304033	0.26605566	0.28651846	0.206999616
0.229696376	0.305469448	0.258224895	0.347262854	0.219090677
0.19533197	0.206126229	0.258618969	0.207747065	0.130651899
0.205441565	0.175021007	0.258063856	0.190808851	0.202571062
0.120057713	0.266221686	0.402817984	0.149902879	0.4466758
0.742930932	0.348227007	0.442362195	0.430086872	0.128942521
0.152702967	0.331343609	0.398347659	0.224984887	0.244041769
0.139061986	0.251372721	0.243602357	0.210530331	0.160250415
0.127697307	0.22268866	0.302163227	0.129893397	0.091975919
0.22636142	0.378168211	0.292924344	0.188887613	0.105967958
0.23338763	0.271551926	0.522390295	0.304222761	0.131087316
0.129479894	0.346253366	0.372290317	0.20424887	0.38979838
0.247157056	0.313839751	0.688104699	0.252875645	0.465353834
0.293961679	0.285487602	0.235826868	0.232145304	0.410427698
0.193646858	0.498132001	0.374360468	0.239301277	0.096154621
0.374620045	0.206211972	0.435909334	0.357917751	0.102322832
0.243382948	0.288151613	0.39559607	0.196350068	0.146553568
0.189911609	0.246148354	0.214790189	0.234815574	0.296396435
0.211481006	0.328621779	0.443651884	0.286617777	0.178673799
0.158351232	0.433048345	0.278992839	0.194966749	0.13187107
0.344290911	0.290538247	0.437938442	0.27302406	0.148146865
0.225765978	0.372187111	0.254123201	0.286478743	0.119957894
0.187712939	0.269134641	0.232032693	0.338681244	0.355765865
0.191657186	0.221626147	0.290497973	0.314405857	0.208569485
0.158120903	0.244634537	0.319325573	0.212612747	0.401340875
0.275571768	0.59828301	0.510435297	0.311801549	0.229092161
0.188782901	0.288951647	0.295493848	0.271853254	0.122622872
0.274770685	0.245892562	0.35150083	0.245790319	0.117115181
0.108645475	0.297054593	0.479133635	0.218984399	0.295903774

O00468,AGRN,INFLAMMATION	Q99538,LGMN,INFLAMMATION	Q9HCB6,SPON1,INFLAMMATION	Q15389,ANGPT1,INFLAMMATION	O75475,PSIP1,INFLAMMATION
O00468	Q99538	Q9HCB6	Q15389	O75475
AGRN	LGMN	SPON1	ANGPT1	PSIP1
INFLAMMATION	INFLAMMATION	INFLAMMATION	INFLAMMATION	INFLAMMATION
0.358787119	0.405423045	0.327167195	0.199588431	0.466225558
0.309905443	0.353602407	0.324502163	0.105075629	0.60617237
0.44028207	0.393872337	0.393708564	0.133711921	0.658383461
0.353063601	0.50006932	0.229362271	0.065985827	0.835493205
0.363467935	0.350236173	0.34035212	0.180766595	0.560932858
0.469631147	0.310873598	0.171264732	0.079263473	0.553056968
0.341889021	0.341155174	0.334041673	0.177796644	0.701152418
0.323446723	0.317779948	0.306126533	0.07129279	0.486226355
0.303611849	0.303969821	0.273706196	0.270331212	0.449190711
0.141856091	0.296684199	0.267201498	0.999653486	0.790479525
0.323783192	0.365033304	0.245875519	0.235924966	0.554438751
0.596998826	0.511356024	0.542050811	0.585564501	0.934651216
0.268594189	0.322148995	0.273289133	0.164026407	0.562139462
0.272173781	0.249411519	0.241182979	0.102166916	0.515519891
0.194009607	0.281576727	0.162949858	0.273421766	0.585239885
0.309948408	0.209801951	0.164174276	0.441443284	0.590046271
0.375321801	0.607307883	0.310163323	5.754138713	0.813717543
0.305321269	0.274028908	0.264108017	0.089089548	0.755864742
0.316922062	0.527484278	0.282006437	0.08739533	0.749863549
0.315606757	0.404945596	0.210355289	0.077411945	0.821937158
0.262920766	0.364098323	0.322461762	0.055923559	0.824733549
0.327757341	0.483537606	0.208468311	0.297363607	0.699259571
0.30418059	0.238986329	0.302267967	0.146006044	0.713210575
0.267238542	0.241902905	0.226047834	0.050369702	0.524894723
0.317449718	0.346974129	0.224190958	0.068076468	0.712321282
0.381855842	0.361783872	0.262811443	0.429818653	0.567227742
0.299245198	0.371877663	0.245058824	0.064627859	0.596709231
0.286558183	0.481030408	0.207186226	1.160543018	0.55118174
0.374308574	0.41754396	0.261738853	0.310787417	0.56616717
0.307019033	0.317405713	0.251651657	0.063804412	0.457327633
0.263486326	0.299100038	0.18485936	0.413970538	0.816995479
0.322484114	0.383686524	0.267664924	0.160751043	0.477343593
0.264584417	0.378246857	0.242372849	0.402120558	0.452785606
0.277392368	0.241099406	0.224066675	0.110360821	0.547526235
0.329511335	0.352086057	0.291526715	0.200865264	0.708627815

Q9UN19,DAPP1,INFLAMMATION	P52564,MAP2K6,INFLAMMATION	P34896,SHMT1,INFLAMMATION	Q13241,KLRD1,INFLAMMATION	P35613,BSG,INFLAMMATION
Q9UN19	P52564	P34896	Q13241	P35613
DAPP1	MAP2K6	SHMT1	KLRD1	BSG
INFLAMMATION	INFLAMMATION	INFLAMMATION	INFLAMMATION	INFLAMMATION
0.703099123	0.688820507	0.123510021	0.389285361	0.775446954
0.694429432	0.576423077	0.097476618	0.604033303	0.790917982
0.601860066	0.500207987	0.129408115	0.362788342	0.983706609
1.368030125	0.449439865	0.257831422	0.47813834	0.773085562
0.688486371	0.714051482	0.166028176	0.589024684	0.744116286
1.011853201	0.723317018	0.148733342	0.278085418	0.708529585
1.006188077	0.390636862	0.120591489	0.368541758	0.562334318
0.997093015	0.583659958	0.067798633	0.287353793	0.730117214
0.627548993	0.480930391	0.172707184	0.374075141	0.421732468
0.934392112	0.503687209	0.246233678	0.376337772	0.379691603
0.554169801	0.403628514	0.110437344	0.26067064	0.532074438
0.704806923	0.915987617	0.189503973	0.609458546	1.124980504
0.840954704	0.54566976	0.100502271	0.461275317	0.76418826
0.641090529	0.35201285	0.118906542	0.297281172	0.533034198
0.806585849	0.49768334	0.102443475	0.205156959	0.444482955
7.714646226	0.595552253	0.246729136	0.428183171	0.396721915
0.736521732	0.54699517	0.428539472	0.385632888	0.668037039
0.918021597	0.854054476	0.085046755	0.3323557	0.684679157
0.878917709	0.685676508	0.141277145	0.329534176	0.687341989
1.358297971	0.664020644	0.156659048	0.352183689	0.642024385
0.513522715	0.656242075	0.102251931	0.346757742	0.559030925
1.04746352	0.455966583	0.237879029	0.333995368	0.509763506
0.735756353	0.473685035	0.09097412	0.250833162	0.559496108
1.006467091	0.463261919	0.157814317	0.196636085	0.497097241
0.93789609	0.487745342	0.104176372	0.623430222	0.673570098
0.599777788	0.439794053	0.123930226	0.317405713	0.634855429
1.097408001	0.403348837	0.124343242	0.511568735	0.499376557
0.647701055	0.607602622	0.29569874	0.372264513	0.489879899
0.866456965	0.51630662	0.216824547	0.4466758	0.73825954
0.636397468	0.354535009	0.119170578	0.218317548	0.611320139
0.643093316	0.418007288	0.05250505	0.37131101	0.447636629
0.730117214	0.757228177	0.126648334	0.31355708	0.607771108
0.669288439	0.288891568	0.158362209	0.447202451	0.511001701
1.160462578	0.486597226	0.088984631	0.475494316	0.561438538
0.600901322	0.543066208	0.406435972	0.451939011	0.666880422

Q6ZUJ8,PIK3AP1,INFLAMMATION	P14210,HGF,INFLAMMATION	P09874,PARP1,INFLAMMATION	P22304,IDS,INFLAMMATION	Q9HD26,GOPC,INFLAMMATION
Q6ZUJ8	P14210	P09874	P22304	Q9HD26
PIK3AP1	HGF	PARP1	IDS	GOPC
INFLAMMATION	INFLAMMATION	INFLAMMATION	INFLAMMATION	INFLAMMATION
1.825510542	0.363417551	1.52118962	0.799184219	0.742879438
1.901713612	0.378876615	1.432266542	0.772764112	0.771051966
2.885857374	0.558527414	1.421387582	0.834393602	0.832718049
2.214222191	0.369872514	1.407173286	0.954753362	0.732042848
2.708323361	0.454105684	0.950659101	0.84950833	0.629291342
1.581068638	0.400534939	1.484317786	0.644834125	0.676189725
1.772271952	0.344744634	1.025551542	0.57271925	0.850922709
0.841246207	0.262483747	0.826163942	0.645683918	0.638739688
1.697133436	0.32483973	1.036557394	0.614378655	0.778732639
1.690675755	0.31983506	1.582823072	0.55632506	0.449097314
1.366040257	0.33457464	1.374207631	0.645862965	0.849331699
3.183910981	0.624468195	1.270943591	0.783388579	0.812365006
1.832991378	0.258278597	1.45195828	0.737594601	0.979420298
1.645128185	0.271326149	6.487668032	0.721114372	0.373220465
1.121943481	0.260490019	1.060687741	0.580633614	0.690445765
2.512458907	0.431340773	1.340340919	0.517058707	0.851866936
4.476279123	0.597288562	1.244097885	0.747735534	0.692074858
2.091879863	0.264217879	1.590521639	0.880930433	0.811014717
1.912023162	0.350066278	1.285028182	0.7275912	0.682830781
1.931872658	0.335875856	1.46886316	0.650580732	0.87502734
1.837061596	0.318198734	1.046665173	0.643271644	0.578704994
3.179720595	0.345198954	1.40288816	0.570421395	0.877943498
1.125916626	0.306232647	0.928645581	0.646669288	0.611235398
1.040011874	0.259912874	0.973126997	0.592587459	0.599154511
2.177994031	0.263705579	1.441928871	0.63820862	0.642113395
2.383530357	0.287692596	1.176172774	0.68060988	0.850156295
2.862746652	0.28210419	1.240051468	0.556517901	0.700084029
4.707953268	0.409546736	1.080276034	0.62563798	1.097560145
3.145523194	0.44070953	1.423458073	0.714645661	1.8303252
1.268743121	0.228806509	1.267073312	0.624295079	0.898381423
1.821213426	0.272324748	1.171778594	0.500763044	0.631476084
2.076998249	0.365311734	1.043550198	0.682878113	0.761755544
1.715465367	0.246729136	1.135163416	0.641090529	0.636662193
1.976708313	0.220415869	1.269358868	0.615785585	0.730724761
5.61155264	0.382306067	1.611942185	0.66595657	1.186819059

Q7KYR7,BTN2A1,INFLAMMATION	P47712,PLA2G4A,INFLAMMATION	Q9BXN2,CLEC7A,INFLAMMATION	P51671,CCL11,INFLAMMATION	P29965,CD40LG,INFLAMMATION
Q7KYR7	P47712	Q9BXN2	P51671	P29965
BTN2A1	PLA2G4A	CLEC7A	CCL11	CD40LG
INFLAMMATION	INFLAMMATION	INFLAMMATION	INFLAMMATION	INFLAMMATION
0.223740759	0.719367058	0.386569575	0.087595466	0.288571354
0.665080096	0.693275172	0.622911883	0.104335354	0.250781008
0.563700206	0.662916929	0.518961709	0.098899018	2.352671674
0.381961729	0.746027129	0.45031299	0.097909998	0.177574952
0.448848351	0.782628742	0.420244254	0.107238883	0.204702411
0.473586546	0.592300004	0.397052036	0.089958287	0.123381671
0.492057903	0.620326645	0.237500096	0.119228414	0.225062874
0.546010273	0.53972634	0.396117405	0.08374228	0.165236006
0.373790032	0.620326645	0.327371356	0.096649093	0.220232608
0.340871527	0.734940826	0.350989554	0.140905517	0.299390428
0.317934174	0.675768028	0.354117489	0.106424336	0.16631613
0.335992282	0.873088619	0.629073284	0.161365045	0.352990191
0.420098634	0.558411284	0.384645144	0.063605706	0.178055635
0.3013475	0.528142813	0.163447589	0.068797502	0.160239308
0.26226551	0.489336906	0.141993815	0.068308078	0.192656131
0.302918165	0.568014632	0.327394048	0.082406389	0.250450952
0.438242103	0.793113915	0.196608827	0.142111972	2.087534441
0.377618146	0.673896996	0.207286778	0.143199663	0.24954986
0.375087737	0.724270239	0.386891248	0.113573645	0.200406332
0.425697346	0.644566002	0.471490301	0.083319616	0.180028847
0.408894341	0.590291716	0.220782848	0.084476859	0.146889176
0.337743521	1.081699673	0.324682155	0.063521994	0.198223512
0.368669507	0.640513108	0.332701436	0.093629048	0.172360369
0.340942416	0.47237352	0.25	0.089976995	0.15442733
0.422346793	0.518135019	0.352086057	0.097943937	0.184500931
0.458629163	0.596667872	0.23338763	0.113597265	0.227178782
0.618094786	0.662319849	0.535107257	0.114808392	0.201744325
0.395048038	0.620326645	0.320012464	0.081882559	0.467811749
0.475988954	0.723617899	0.380060237	0.089213138	0.393544859
0.381670609	0.669752516	0.264767877	0.099386933	0.106571974
0.339104069	0.482332524	0.325019909	0.087838669	0.167240944
0.44217826	0.658064089	0.35130597	0.075902962	0.178649032
0.309218813	0.537039459	0.382147103	0.100369998	0.231326107
0.347527729	0.61869488	0.354092944	0.091721261	0.17685023
0.402176308	0.70275806	0.289512993	0.098311228	0.205128521

P07148,FABP1,INFLAMMATION	Q14118,DAG1,INFLAMMATION	Q8WU39,MZB1,INFLAMMATION	O43291,SPINT2,INFLAMMATION	Q9H4D0,CLSTN2,INFLAMMATION
P07148	Q14118	Q8WU39	O43291	Q9H4D0
FABP1	DAG1	MZB1	SPINT2	CLSTN2
INFLAMMATION	INFLAMMATION	INFLAMMATION	INFLAMMATION	INFLAMMATION
0.101912294	0.299494206	0.151921715	0.249670972	0.297404834
0.076925195	0.405451147	0.260941805	0.44220891	0.43875881
0.161981393	0.386649968	0.710644531	0.425756364	0.622393975
0.088615323	0.283712181	0.151092086	0.39166714	0.383792919
0.304138424	0.37618129	0.269395937	0.465999399	1.849582945
0.151385612	0.339621571	0.147869868	0.335340817	0.48801588
0.086857847	0.368669507	0.368873998	0.424813053	0.599777788
0.088027616	0.349024448	0.204943764	0.40114619	0.362034728
0.103600294	0.299639557	0.225656462	0.347672292	0.257795681
0.027206591	0.238324636	0.160962888	0.327167195	0.240115427
0.08135642	0.264456071	0.14123798	0.369872514	0.384751805
0.157847137	0.422668939	0.23607219	0.56550042	0.473619373
0.042715397	0.303443537	0.147521793	0.347551819	0.38966331
0.065926395	0.237302631	0.236285008	0.303653941	0.315825595
0.03156129	0.230015024	0.097382072	0.317625798	0.285329338
0.049377582	0.264602757	0.259858832	0.318971626	0.227304791
0.072649667	0.406238816	0.206412179	0.848802023	0.556942387
0.204036618	0.319082192	0.351988451	0.355617937	0.378719077
0.086365564	0.336878439	0.344171609	0.346685644	0.352696704
0.108909371	0.278896165	0.171466661	0.326736606	0.389285361
0.087704824	0.354805431	0.146767048	0.324457181	0.374931774
0.12947092	0.208555028	0.148784898	0.365008003	0.225672104
0.117424064	0.284086071	0.202500868	0.381379711	0.266646445
0.06648165	0.224253126	0.222380162	0.307040315	0.319037961
0.161970165	0.343266268	0.171359728	0.440587357	0.305278946
0.100022744	0.305427104	0.290477837	0.425343408	0.436816725
0.064847735	0.298085892	0.217035057	0.461787171	0.328189275
0.060358468	0.243501067	0.269414611	0.433078363	0.229346373
0.061082376	0.36645298	0.491376238	0.418239144	0.284440736
0.13651203	0.309133091	0.106956792	0.451500659	0.307551518
0.077723787	0.180754065	0.201088154	0.414544821	0.305046271
0.137499664	0.340658946	0.192722912	0.435064136	0.350479022
0.115823508	0.205527023	0.188442986	0.351842094	0.320434191
0.077739951	0.249948019	0.215969576	0.403740439	0.252035699
0.09594823	0.340446498	0.236908193	0.41091161	0.312840674

Q99895,CTRC,INFLAMMATION	O60449,LY75,INFLAMMATION	Q9NRJ3,CCL28,INFLAMMATION	Q9Y6N7,ROBO1,INFLAMMATION	P02778,CXCL10,INFLAMMATION
Q99895	O60449	Q9NRJ3	Q9Y6N7	P02778
CTRC	LY75	CCL28	ROBO1	CXCL10
INFLAMMATION	INFLAMMATION	INFLAMMATION	INFLAMMATION	INFLAMMATION
0.356852553	0.683635869	0.962461008	0.769610293	0.124076345
0.265834453	0.595676108	0.897696702	0.708529585	0.222056699
0.421586332	0.471000338	0.970567193	0.805021936	0.219668514
0.304433705	0.2200495	0.896328826	0.490015741	0.214834858
0.362536963	0.551372798	0.851807891	0.773407146	0.298416663
0.393954249	0.51652139	1.112573	0.672730233	0.161242057
0.485956809	0.336365116	0.932774336	0.538605176	0.181017364
0.238341156	0.303212261	0.955879059	0.592587459	0.172181256
0.309540481	0.340210601	0.914972317	0.530932367	0.227430871
0.185565446	0.463069294	1.001664938	0.402315716	0.380112928
0.244329505	0.430206134	0.892485107	0.544951597	0.14839352
0.484309095	0.742107452	0.88441782	0.836362336	0.773782497
0.13107823	0.598946896	1.041021598	0.544045795	0.106697627
0.186300054	0.308897479	0.87151657	0.436332548	0.189057895
0.190941155	0.136464727	0.94461608	0.347648194	0.105133912
0.480930391	0.283103208	0.852339442	0.408695992	0.191166284
0.397052036	0.463743832	0.959330613	0.593450663	0.246900215
0.327484834	0.440740079	1.162314108	0.578223841	0.157104891
0.28628024	0.465611953	0.867177963	0.64042432	0.26135814
0.210311552	0.490967693	0.89001404	0.651438098	0.176568513
0.139438417	0.225296999	0.917576279	0.640291161	0.426553912
0.164550236	0.395486403	1.005909142	0.513273613	0.290820324
0.251041886	0.517704226	0.895087111	0.625854847	0.184321977
0.281303615	0.222904864	0.946910515	0.417312488	0.100085161
0.092814885	0.373401596	1.053872254	0.608487698	0.207143147
0.26508005	0.194953235	0.909345219	0.556672222	0.150110833
0.302477555	0.443836432	0.962327592	0.492774668	0.18206178
0.236744038	0.334157463	0.827195361	0.459647564	0.149291093
0.185411162	0.471261588	0.847919965	0.596378437	0.177882933
0.167426523	0.367470413	0.930965752	0.378482893	0.130516128
0.227809529	0.386649968	0.798575103	0.355002232	0.200865264
0.148259864	0.547108927	0.888596276	0.691787092	0.163209846
0.368899567	0.200253588	0.709856838	0.462973011	0.199851458
0.223539239	0.369923793	1.05709131	0.399952341	0.11517504
0.24212098	0.349944976	0.825934912	0.482666967	0.168895173

O15455,TLR3,INFLAMMATION	Q08174,PCDH1,INFLAMMATION	B1AKI9,ISM1,INFLAMMATION	P10147,CCL3,INFLAMMATION	Q8TAD2,IL17D,INFLAMMATION
O15455	Q08174	B1AKI9	P10147	Q8TAD2
TLR3	PCDH1	ISM1	CCL3	IL17D
INFLAMMATION	INFLAMMATION	INFLAMMATION	INFLAMMATION	INFLAMMATION
0.885644734	0.655969208	0.875937598	0.288431373	0.454830214
0.580633614	0.655560121	0.815467904	0.403684472	0.402009082
0.567385033	0.747010279	0.960927838	0.331274716	0.545556303
0.503198665	0.681317893	0.505856469	0.401897637	0.362486708
1.009541348	0.667065346	0.64936431	0.421381827	0.467098915
0.380402861	0.639537118	0.752206146	0.440617897	0.42566784
0.421206615	0.553939376	0.402817984	0.35507606	0.492808826
0.347407306	0.513380356	0.852812211	0.402399384	0.336062157
0.361107427	0.468623108	0.552367369	0.33641175	0.473980628
0.199533101	0.478370391	0.284815586	0.320878715	0.443990281
0.243214307	0.470706604	0.563153453	0.298996396	0.481898094
0.718619505	0.748461493	1.044853011	0.55091437	0.642246933
0.412281034	0.551067137	0.587638164	0.286022392	0.446459124
0.30067983	0.49342407	0.462684284	0.275705509	0.526388545
0.197250382	0.412423944	0.299951261	0.263632475	0.313709256
0.593491799	0.441198564	0.213410047	0.266184782	0.347768701
0.726784723	0.566402681	0.59526336	0.260020991	0.494622578
0.806529943	0.805412629	0.53968893	0.338235502	0.622480263
0.503547577	0.534662352	0.413683695	0.375712234	0.552520539
0.203626891	0.596295767	0.406999803	0.439123911	0.382359069
0.35128162	0.550189305	0.70921748	0.308961719	0.337977708
0.358414274	0.506874327	0.567817808	0.432868283	0.313665769
0.172791002	0.451939011	0.458819941	0.308619259	0.520763414
0.547716026	0.419749351	0.432538363	0.289773988	0.531779475
0.411339066	0.500034659	0.605584422	0.501909793	0.503128912
0.288791463	0.550494479	0.447791795	0.473980628	0.432388483
0.438029518	0.500450749	0.371182346	1.67144977	0.393353957
0.526023807	0.477310507	0.476517136	0.277104107	0.47237352
0.820912295	0.662503509	0.560777356	0.403712455	0.508704584
0.398900269	0.481030408	0.556942387	0.519861781	0.485149066
0.139874027	0.5538242	0.314689293	0.508986748	0.453728127
0.55179336	0.611871242	0.712963438	0.262975445	0.521919786
0.14389616	0.507225788	0.355790526	0.334273293	0.36759779
0.529168834	0.622393975	0.287114879	0.308576478	0.317647815
0.450032158	0.501388218	0.399232203	0.257278	0.461595159

Q15166,PON3,INFLAMMATION	O95644,NFATC1,INFLAMMATION	O43597,SPRY2,INFLAMMATION	Q92484,SMPDL3A,INFLAMMATION	Q92844,TANK,INFLAMMATION
Q15166	O95644	O43597	Q92484	Q92844
PON3	NFATC1	SPRY2	SMPDL3A	TANK
INFLAMMATION	INFLAMMATION	INFLAMMATION	INFLAMMATION	INFLAMMATION
0.862502175	1.042610287	0.962994858	0.287732482	0.669798941
0.74913623	1.008632068	0.877882646	0.446057005	0.54536726
0.752466886	0.862681546	0.842004588	0.418413121	0.714298997
0.713556711	0.899440654	0.578384181	0.401925495	1.002637431
0.964130272	0.899128986	1.004445996	0.536109647	0.99833783
0.70588252	0.901312935	0.789493887	0.217909349	0.647476618
0.5121009	0.743446071	0.858565436	0.200059355	0.783062844
0.348396008	0.925561012	0.73825954	0.430892532	0.39713461
0.763288309	0.716927929	0.883621238	0.308897479	1.079228237
0.499792099	0.925176162	0.650220073	0.093973644	0.860054507
0.555978115	0.879100493	0.635868347	0.683446351	0.926459618
0.764029368	0.865316609	0.56120509	0.36437604	1.112341671
0.642514091	0.878917709	0.713210575	0.301431063	1.215121015
0.414343731	0.759014841	0.458089055	0.450656467	0.559884055
0.371336748	0.937636086	0.641801915	0.338728199	0.500450749
0.58394322	1.218832606	0.421820174	0.231743375	0.748980467
0.855950932	0.988970916	1.038427144	0.229807852	1.038283197
0.90802253	0.826679491	0.896453092	0.445717033	0.680279726
0.701298234	0.771586603	0.64698313	0.330380399	0.658839976
0.66429686	0.855417128	0.164641507	0.281108698	0.893413525
0.576702827	0.795757084	0.595882589	0.24854862	0.730167824
0.48454414	1.118992216	0.892546971	0.221165767	0.754190038
0.426494783	0.586539429	0.578344092	0.209047109	0.64546018
0.692506731	0.737594601	0.798796546	0.226722582	0.848684362
0.555400353	0.833179934	0.670356296	0.276528484	0.497304021
0.575305433	0.686580125	0.583498155	0.393081399	0.504595764
0.82262111	0.800015579	0.741387656	0.374542153	0.861964286
0.568566105	0.696454013	0.785999337	0.265834453	0.980982972
0.775661984	0.895956131	0.765089273	0.482332524	1.101141598
0.487610129	0.875816175	0.444267343	0.355125288	0.808096786
0.604326453	0.712469421	0.650175005	0.297961947	1.384053777
0.636000586	0.838161407	0.900688409	0.247071413	0.678631358
0.399066201	0.515091272	0.783714449	0.337556288	0.572084436
0.534143765	1.164652856	0.869887055	0.314079133	0.832544908
0.677597285	0.954753362	0.742827947	0.679243142	0.63785482

Q8NHJ6,LILRB4,INFLAMMATION	P14784,IL2RB,INFLAMMATION	Q9NZN5,ARHGEF12,INFLAMMATION	Q9Y6Q6,TNFRSF11A,INFLAMMATION	Q9NQ76,MEPE,INFLAMMATION
Q8NHJ6	P14784	Q9NZN5	Q9Y6Q6	Q9NQ76
LILRB4	IL2RB	ARHGEF12	TNFRSF11A	MEPE
INFLAMMATION	INFLAMMATION	INFLAMMATION	INFLAMMATION	INFLAMMATION
0.521594297	1.058998099	1.203052832	0.462427788	1.090734521
0.584267117	0.837638698	1.162797601	0.396144863	1.465811918
0.554361895	0.833526516	1.263827883	0.359160351	1.548745403
0.535701039	0.865676559	1.73411551	0.267423842	0.715537856
0.662916929	1.276859689	0.989725257	0.472766593	1.160140874
0.487441165	0.901437892	1.385301498	0.393872337	1.358203825
0.381089034	0.925240293	0.923894485	0.316044585	1.36074808
0.351817707	0.789822296	0.803461054	0.36966747	1.584030374
0.331136971	1.259106223	1.282181057	0.23230627	0.770090551
0.387669731	1.129982163	2.52013322	0.273668255	1.060981867
0.375894575	1.001803808	0.88871947	0.328030076	0.8019032
0.443621133	0.894652918	1.167724548	0.643360827	2.058937447
0.249688278	1.054749205	1.162233545	0.301431063	1.345833558
0.25222794	0.954621014	1.106497353	0.266868328	1.172184772
0.159474759	0.92242275	0.999168569	0.245126778	0.654470468
0.459998161	1.110646728	1.279872414	0.269283922	0.912945093
0.235108728	0.984115806	1.714276709	0.292701085	1.133355136
0.379244457	1.081025084	0.942131274	0.342149798	0.827195361
0.381855842	1.045722457	0.860292998	0.328644558	1.107801963
0.367827181	0.760542092	1.000138639	0.316285649	1.302063673
0.415666965	1.003889167	0.972048363	0.210238676	1.329791444
0.450593997	0.813773948	1.424346349	0.34191272	1.253098654
0.271288538	0.895087111	0.922742493	0.28449989	1.24616923
0.251756337	1.061570364	0.804742985	0.254123201	1.18640781
0.562256368	1.075792622	1.247811491	0.429044739	1.187395047
0.383048772	0.627548993	1.047826606	0.189333289	0.956740781
0.300784056	1.298188607	1.134219606	0.287015389	1.138394029
0.473258394	0.849213964	1.251189231	0.265945033	0.789986552
0.473422441	1.046665173	1.095432053	0.355963199	1.769571432
0.465095859	1.341084368	1.346486719	0.282182417	0.89428092
0.42401876	1.013537874	1.29020472	0.373608711	1.053288025
0.338681244	0.683967652	1.169182386	0.351232926	1.287524602
0.329991324	0.863160051	0.86904332	0.275973185	1.246428391
0.263486326	1.19731271	1.03089686	0.296869338	0.882458293
0.392564059	0.883192607	1.532195087	0.295002687	1.186572292

Q03431,PTH1R,INFLAMMATION	O94992,HEXIM1,INFLAMMATION	Q6UXH1,CRELD2,INFLAMMATION	P43234,CTSO,INFLAMMATION	O14788,TNFSF11,INFLAMMATION
Q03431	O94992	Q6UXH1	P43234	O14788
PTH1R	HEXIM1	CRELD2	CTSO	TNFSF11
INFLAMMATION	INFLAMMATION	INFLAMMATION	INFLAMMATION	INFLAMMATION
0.445500822	0.606214388	0.384405265	0.477906402	0.729459608
0.476979775	0.760015107	0.748254005	0.461595159	0.632439771
0.535515412	1.122099026	0.482499717	0.460700159	1.287703104
0.39044737	0.520943928	0.407592667	0.329305839	0.649499356
0.501006074	0.852103156	0.443928735	0.686437369	0.441504485
0.441198564	0.751216158	0.467746901	0.351793322	1.022074245
0.411652816	0.603405604	0.577422808	0.265392591	0.630994791
0.467098915	0.694044465	0.51359391	0.30174463	0.976031761
0.345270744	0.608825208	0.410569966	0.324907286	0.379981214
0.372600109	0.980439151	0.320456403	0.285309561	0.955018112
0.347407306	0.583295965	0.242188119	0.290618812	0.524931108
0.667296573	1.157891442	0.583255536	0.688009314	1.139578257
0.519609603	0.767479436	0.32653284	0.341983826	1.249975658
0.314035595	0.419807545	0.332102388	0.303653941	1.004028346
0.261394375	0.47519778	0.235385931	0.177747355	0.537672653
0.368746178	0.751893378	0.324052619	0.266092546	0.819604608
0.341391726	0.968148327	0.431400574	0.415839872	0.434732543
0.592628536	0.710447526	0.430713366	0.242440058	0.837812898
0.330151476	0.61021942	0.551219947	0.42581539	0.44217826
0.499030535	0.68700857	0.379139323	0.330907524	1.218156929
0.351842094	0.515376979	0.355543996	0.23483185	0.398126829
0.395294558	0.573116366	0.289874433	0.449533333	0.64743174
0.357967372	0.579990028	0.322931482	0.279321785	0.457771642
0.337345775	0.569710144	0.306423745	0.245875519	0.611659221
0.472733825	0.63016433	0.419545737	0.362461583	0.792949009
0.417688695	0.522933718	0.4118526	0.316351425	0.636794597
0.36680876	0.841187898	0.680657058	0.322104339	0.405507359
0.322528823	0.776361241	0.281225632	0.402399384	0.790589117
0.471719125	0.935818075	0.535366956	0.521594297	0.449720327
0.371028008	0.620025734	0.547526235	0.232918962	0.535998177
0.333093706	0.748409616	0.480031172	0.366580005	0.586295545
0.461243345	0.457042427	0.423079298	0.49373198	0.630208012
0.294185899	0.646848608	0.446366295	0.239185195	0.383394089
0.600276877	0.49627098	0.31214754	0.353823064	0.788017733
0.409149502	1.015858879	0.541262371	0.361332768	0.628724549

Q29980_Q29983,MICB_MICA,INFLAMMATION	Q96LA5,FCRL2,INFLAMMATION	P42701,IL12RB1,INFLAMMATION	P26951,IL3RA,INFLAMMATION	Q12866,MERTK,INFLAMMATION
Q29980_Q29983	Q96LA5	P42701	P26951	Q12866
MICB_MICA	FCRL2	IL12RB1	IL3RA	MERTK
INFLAMMATION	INFLAMMATION	INFLAMMATION	INFLAMMATION	INFLAMMATION
0.395788061	0.371465466	0.714893381	1.143455325	0.51584159
0.565030245	0.363518326	1.31494276	0.998683887	0.416157055
0.664250816	0.363644333	0.915670215	1.172266024	0.456662427
0.035904289	0.445068715	1.225949881	1.126306907	0.463005103
0.580311732	0.336295178	1.505351087	1.453770968	0.589188019
0.735807354	0.292498271	1.144168872	0.96339544	0.39044737
0.288751431	0.445778827	0.990136957	1.109569472	0.319480548
0.317163795	0.295370981	0.849331699	0.97440943	0.297157562
0.431340773	0.238242054	0.83098826	1.103357258	0.309905443
0.615785585	0.21851436	0.900875721	1.04710056	0.246694935
0.575744249	0.349435963	0.835782815	1.031540168	0.323312233
0.488963947	0.371980784	0.979284531	1.270767413	0.576423077
0.046723754	0.217110289	0.560311108	0.90645041	0.274618362
0.447109467	0.213602435	0.631388549	0.985207831	0.242692259
0.354830026	0.184475355	0.890507705	1.029040669	0.242658617
0.888473099	0.2085984	0.994056666	0.954687186	0.316241805
0.045342256	0.28651846	0.860292998	1.087036211	0.357273298
0.517488964	0.588453369	1.078555189	1.304683618	0.413081971
0.62676651	0.32376075	0.950922716	1.064739129	0.398817328
0.036481276	0.268556957	0.8272527	1.181893467	0.420419065
0.634151742	0.158757868	0.554669384	0.949671195	0.179343828
0.704220925	0.119038487	0.900376308	1.045577499	0.357496247
0.44590244	0.213276956	0.991991731	0.997093015	0.292356384
0.030881786	0.183417093	0.744322628	1.120078619	0.25605045
0.060371021	0.374152936	0.657106902	1.126697322	0.315650513
0.44723345	0.328599001	0.696357471	0.930643159	0.329032044
0.393872337	0.272362503	1.044201401	1.309757751	0.39253685
0.031642338	0.339386245	1.002498446	1.173648178	0.319037961
0.060765657	0.371362488	0.912945093	1.01395948	0.378666579
0.701638589	0.182504001	0.947632774	1.290473039	0.32005683
0.593532938	0.185488288	0.686484951	1.061055411	0.379191886
0.505611086	0.336691686	0.99806107	1.061496784	0.372316124
0.32371587	0.233112779	0.753040832	0.898568255	0.234392774
0.437362066	0.152967811	1.322529589	1.162878203	0.318485589
0.832602618	0.4144299	1.116358191	1.071922052	0.324389719

P78556,CCL20,INFLAMMATION	Q13261,IL15RA,INFLAMMATION	P29460,IL12B,INFLAMMATION	P12034,FGF5,INFLAMMATION	P28827,PTPRM,INFLAMMATION
P78556	Q13261	P29460	P12034	P28827
CCL20	IL15RA	IL12B	FGF5	PTPRM
INFLAMMATION	INFLAMMATION	INFLAMMATION	INFLAMMATION	INFLAMMATION
0.127114455	0.807424909	0.578504465	0.498339211	0.822336061
0.138178015	0.593862154	1.078480432	0.839498702	0.716331853
0.107536625	0.925881843	0.971846252	0.487170946	1.021578452
0.186636104	0.711482413	0.862621751	0.540100579	0.417312488
0.360857214	1.32317144	1.005142466	0.497062786	1.079527504
0.120583131	0.765672846	0.854172881	0.336085452	0.75126823
0.106823429	0.860710516	0.574747424	0.662411672	0.505505958
0.081254977	0.478105199	0.555015513	0.382067646	0.303780254
0.125913074	1.049498424	0.743033932	0.61839476	0.352354611
0.15161664	0.857911063	0.63542775	0.476913656	0.685438912
0.114165607	0.386355275	0.54329211	0.707204814	0.579066122
0.129767409	0.740155344	0.839324152	0.573156093	0.877395979
0.149270398	0.615230956	0.360182502	0.361458018	1.095356126
0.078047704	0.629073284	0.765885165	0.33877516	0.297404834
0.071883278	0.540512542	0.603154707	0.451219087	0.489506526
0.089939583	0.772282187	0.668824685	0.473258394	0.385739823
0.163844597	0.883192607	1.122565791	0.531079593	0.554977043
0.167751782	0.892856358	0.616939105	0.518278697	0.854824404
0.135484527	0.425844907	0.75917269	0.426022048	0.4118526
0.090565162	1.028684092	1.068880078	0.29259966	0.560349948
0.100788295	0.541975672	0.749759603	0.379139323	0.326510208
0.138667346	0.869224052	0.782845763	0.513878786	0.496890547
0.129794396	0.848625538	0.667712984	0.378509129	0.386489199
0.105184935	0.561243991	0.297446065	0.470315244	0.355371527
0.069593675	0.643405423	0.668592928	0.477972659	0.437665327
0.142565816	1.008632068	0.679808356	0.405563578	0.469859069
0.085076235	0.395458991	0.672310693	0.604242682	0.626983769
0.139322483	0.837406488	0.596213108	0.628071191	0.386248169
0.118216228	0.615401557	0.58394322	0.494622578	0.67562752
0.054093535	0.834856416	0.283142457	0.458152565	0.46557968
0.147409356	0.683067473	0.428539472	0.426790509	0.43970261
0.168801543	0.635383707	0.689537061	0.486765897	0.638695415
0.175300255	0.575345312	0.542013241	0.385098658	0.339433297
0.207574338	0.759962428	0.578183763	0.406858772	0.876059037
0.114015352	0.861904541	0.643137893	0.571291908	0.44070953

P78410,BTN3A2,INFLAMMATION	Q9P0M4,IL17C,INFLAMMATION	P13232,IL7,INFLAMMATION	P28838,LAP3,INFLAMMATION	Q7Z6M3,MILR1,INFLAMMATION
P78410	Q9P0M4	P13232	P28838	Q7Z6M3
BTN3A2	IL17C	IL7	LAP3	MILR1
INFLAMMATION	INFLAMMATION	INFLAMMATION	INFLAMMATION	INFLAMMATION
0.502257811	0.403488651	0.545858908	0.363745171	0.677456398
0.432148782	0.434340985	0.480397317	0.322417063	0.96072804
0.621962714	0.35167142	0.237269736	0.659205416	1.265844333
0.704806923	0.500624222	0.408526055	0.734686159	0.754608366
0.527996401	0.376363858	0.634151742	0.311412768	0.807760778
0.568960341	0.268203506	0.482867744	0.585726876	0.706127202
0.473356816	0.268687293	0.43431088	0.407875286	0.564873607
0.512775771	0.538082764	0.578023479	0.366757913	0.570856486
0.39727227	0.40666141	0.479034013	0.529866198	0.665725807
0.365590377	0.526534511	0.477310507	0.739386189	0.553363733
0.302498522	0.21807556	0.519537575	0.542765152	0.422844758
0.623732786	0.538381223	0.536035331	0.458851745	0.802236771
0.465902507	0.589473965	0.585889297	0.302876175	0.572481112
0.372625936	0.285190929	0.493218904	0.237846055	0.506277404
0.225828582	0.292721374	0.487542537	0.28847136	0.446118846
0.401925495	0.226879789	0.454326071	0.380165627	0.323738309
0.443590385	0.550494479	1.145200337	0.43696814	0.832429501
0.459615705	0.320167772	0.509798841	0.507471955	0.678114124
0.385125352	0.249048737	0.460891798	0.463808125	0.900251498
0.536146808	0.37192922	0.379402213	0.321568947	0.733312481
0.39978604	0.346589535	0.46554741	0.422200444	0.498719321
0.382677239	0.812252396	0.619080962	1.031325687	0.639847499
0.294226684	0.428005132	0.4575813	0.313274664	0.510399917
0.364982703	0.517776	0.369872514	0.412910211	0.343980813
0.453005352	0.368567304	0.524312919	0.4181232	0.659479628
0.265392591	0.314275127	0.417862442	0.369001862	0.56663829
0.374412369	0.584550674	0.49338987	0.36389648	0.833699861
0.376572617	0.334876258	0.655287538	0.326464947	0.584915449
0.473258394	0.419632988	0.98241194	0.588575747	0.496787232
0.318198734	0.26072485	0.651167229	0.531300509	0.700084029
0.400979393	0.36478037	0.538306593	0.437392382	0.403684472
0.415004821	0.380455599	0.359708461	0.266295508	1.12015626
0.298395979	0.242456864	0.407988389	0.474736868	0.620111694
0.472275303	0.339057062	0.354535009	0.420302517	0.711877052
0.401257427	0.211686328	0.50596167	0.344076198	0.55290365

O60934,NBN,INFLAMMATION	Q3KPI0,CEACAM21,INFLAMMATION	Q9HBG7,LY9,INFLAMMATION	Q01344,IL5RA,INFLAMMATION	O00182,LGALS9,INFLAMMATION
O60934	Q3KPI0	Q9HBG7	Q01344	O00182
NBN	CEACAM21	LY9	IL5RA	LGALS9
INFLAMMATION	INFLAMMATION	INFLAMMATION	INFLAMMATION	INFLAMMATION
0.332401777	0.235418565	0.376129144	0.156236127	0.456472546
0.397052036	0.335573338	0.470967692	0.190122344	0.50278029
0.439976996	0.309497573	0.608614242	0.29259966	0.411025555
0.397795814	0.286637645	0.329534176	0.307658125	0.481530805
0.464419354	0.706274052	0.567267061	0.21953152	0.586133012
0.271495464	0.712025098	0.317075871	0.237664775	0.411766966
0.373893683	0.278683598	0.350381862	0.314645671	0.403432719
0.225734682	0.272286998	0.294369479	0.360982299	0.384591825
0.434130293	0.456314372	0.397492625	0.289131961	0.335968994
0.374801855	0.256405658	0.311888011	0.166951389	0.486125258
0.317075871	0.455271798	0.328872436	0.217321076	0.33940977
0.341960122	0.377225733	0.480630464	0.391504284	0.681081807
0.328394074	0.19994845	0.324659651	0.292518546	0.339904178
0.23733553	0.407112663	0.317581769	0.206942232	0.246848879
0.940109037	0.15106067	0.260471964	0.241484082	0.211671656
0.375165742	0.239168617	0.395568651	0.212465426	0.327394048
0.302205118	0.575544746	0.327598351	0.30587201	0.428717734
0.424783608	0.212774918	0.512562558	0.365235778	0.465644228
0.320167772	0.493800431	0.349339092	0.274770685	0.400646006
0.305956828	0.292356384	0.337322393	0.271401387	0.388557496
1.746419325	0.235500169	0.207574338	0.267294119	0.48424196
0.366275219	0.646400402	0.271119352	0.160283742	0.489642265
0.326374444	0.476252972	0.287473325	0.137109458	0.350114811
0.248738201	0.394036178	0.228220454	0.246506911	0.243686798
0.229282794	0.256441205	0.372936007	0.386596371	0.43708931
0.326397067	0.462107369	0.358737384	0.234848128	0.387320563
0.463069294	0.33420379	0.385071966	0.174113503	0.410257041
0.40666141	0.99986138	0.290759856	0.201646462	0.372729264
0.293574792	0.578384181	0.400423902	0.266221686	0.416734371
0.354142035	0.620756772	0.361032345	0.174645334	0.420069516
0.330265917	1.09186917	0.281713382	0.171324098	0.283103208
0.329283014	0.207934348	0.402901756	0.259552808	0.418674222
0.305215472	0.164824202	0.273478628	0.317075871	0.306041669
0.394172764	0.222395577	0.311520714	0.203598664	0.293452723
0.476616235	0.161096829	0.350357576	0.237401343	0.361057371

Q9NRM6,IL17RB,INFLAMMATION	P02745,C1QA,INFLAMMATION	P19876,CXCL3,INFLAMMATION	Q92583,CCL17,INFLAMMATION	Q9HCU5,PREB,INFLAMMATION
Q9NRM6	P02745	P19876	Q92583	Q9HCU5
IL17RB	C1QA	CXCL3	CCL17	PREB
INFLAMMATION	INFLAMMATION	INFLAMMATION	INFLAMMATION	INFLAMMATION
0.236104918	0.511214265	0.245398783	0.126938359	0.678396203
0.451782408	0.79901805	0.186351715	0.103621839	0.602402641
0.359733395	0.520871715	0.221119781	0.207775867	0.647835755
0.244821133	0.719267339	0.094640401	0.16996387	0.852398524
0.445068715	0.792949009	0.104147492	0.196241218	0.921719706
0.576822762	0.60344743	0.219775123	0.136143499	0.730978055
0.364957405	0.543555781	0.122164753	0.148465539	0.872120869
0.35072204	0.55118174	0.082899086	0.166951389	0.536927797
0.402678402	0.47539545	0.275399911	0.254334662	1.092777739
0.219592396	0.380086582	0.529278883	0.375217755	0.757963352
0.185809993	0.447543556	0.352770053	0.109052897	0.837987135
0.513558311	0.805691813	0.290981634	0.23027026	0.677127773
0.352672258	0.449657987	0.304518124	0.091733977	0.646221206
0.136068026	0.349823715	0.101559704	0.175957632	0.644834125
0.211965298	0.392455233	0.496339783	0.135353115	0.445161274
0.29619106	0.521196753	0.133369437	0.266775854	0.623992243
0.379796891	0.552443949	2.816102801	0.571014783	0.605920323
0.441351498	0.393572139	0.119816624	0.150997859	0.978673812
0.379428512	0.710693791	0.155382938	0.125390504	0.645907734
0.266535573	0.666048897	0.151490581	0.125756075	0.578704994
0.381802909	0.485754747	0.127343745	0.122529412	0.819207029
0.247105667	0.532000682	0.203909373	0.213084861	0.811520812
0.257135374	0.484275526	0.2659266	0.201940194	0.752049746
0.328462369	0.444667848	0.094804543	0.117489196	0.67633035
0.406943384	0.476385035	0.146198458	0.122130886	0.634327591
0.309626316	0.64403009	0.267702033	0.277065695	0.752101876
0.486192654	0.533810652	0.122240987	0.157508326	0.988628225
0.228394529	0.324254837	0.391857224	0.358836861	0.767373048
0.487711535	0.659845423	0.469728814	0.1376427	0.668222284
0.179791909	0.462876749	0.065303313	0.145480732	0.80068129
0.299909681	0.690732973	0.144265676	0.104538046	0.641846403
0.379586345	0.54566976	0.096669192	0.094038804	0.588494159
0.331366577	0.474112061	0.197866599	0.250728865	0.596750593
0.737390124	0.458947171	0.14389616	0.162352331	0.857197769
0.30665747	0.663238656	0.286955713	0.2561037	0.490661507

Q9UHF4,IL20RA,INFLAMMATION	Q9NZV1,CRIM1,INFLAMMATION	P15260,IFNGR1,INFLAMMATION	P25116,F2R,INFLAMMATION	Q0Z7S8,FABP9,INFLAMMATION
Q9UHF4	Q9NZV1	P15260	P25116	Q0Z7S8
IL20RA	CRIM1	IFNGR1	F2R	FABP9
INFLAMMATION	INFLAMMATION	INFLAMMATION	INFLAMMATION	INFLAMMATION
0.725727578	0.362310871	0.440801183	0.401396516	0.811464564
0.658611679	0.404356582	0.505926601	0.598075697	0.711235874
0.445192132	0.456314372	0.520691225	0.480430617	0.559108429
0.761069442	0.345103258	0.412910211	0.329374324	0.816882227
0.937376154	0.332678376	0.447016503	0.421148228	0.835203695
1.031897734	0.341865324	0.47539545	0.428183171	0.627592493
0.8933516	0.451531956	0.451125268	0.461275317	0.531263683
0.505435884	0.377095019	0.43906304	0.460317118	0.911553982
0.719616414	0.363241262	0.371542718	0.321568947	0.51015233
0.851925985	0.314384065	0.401758374	0.309368883	0.53211132
0.662825035	0.325042439	0.386435623	0.342292124	0.530711604
0.664757475	0.552750373	0.70588252	0.78067826	1.933882311
0.77981294	0.295064037	0.424136339	0.357843331	0.533958677
0.655969208	0.297157562	0.332885976	0.321123466	0.963996625
0.431012017	0.228727225	0.305363599	0.244346442	0.508704584
0.455429611	0.305384766	0.314079133	0.278451892	0.534884759
0.74406471	0.280524756	0.396831925	0.528692219	0.677785181
0.979148782	0.348782606	0.414286295	0.343456668	0.700763725
0.548361808	0.281635285	0.375009747	0.304222761	0.923574343
0.502118575	0.316834205	0.347503641	0.332332663	0.801625329
0.659845423	0.369155357	0.407084445	0.372548459	0.408271283
0.779921053	0.328712905	0.376650931	0.341178822	0.416359025
0.798464405	0.363216085	0.393490306	0.362738052	0.371619986
0.60663473	0.279631734	0.333255364	0.302918165	0.481330584
0.702952933	0.367088545	0.35040615	0.36645298	0.76785191
0.608108222	0.339574493	0.431819411	0.335317574	0.42670177
0.772335719	0.327530236	0.489269074	0.473783546	0.858922578
0.867658962	0.253279111	0.343290062	0.401841926	0.387481678
0.709955252	0.39023092	0.462427788	0.440740079	0.66595657
0.689107039	0.378640333	0.415955183	0.360457232	0.608023926
0.859756486	0.326827209	0.403600537	0.321747312	0.967209285
0.651347796	0.373039421	0.413999233	0.41754396	1.483906303
0.625898229	0.259768788	0.357298064	0.330586565	0.581680965
0.747735534	0.296704765	0.360532195	0.34699818	0.768544126
0.821139932	0.362034728	0.416416748	0.323738309	0.666741762

P53634,CTSC,INFLAMMATION	Q07325,CXCL9,INFLAMMATION	Q9GZT9,EGLN1,INFLAMMATION	Q5ZPR3,CD276,INFLAMMATION	Q6UWV6,ENPP7,INFLAMMATION
P53634	Q07325	Q9GZT9	Q5ZPR3	Q6UWV6
CTSC	CXCL9	EGLN1	CD276	ENPP7
INFLAMMATION	INFLAMMATION	INFLAMMATION	INFLAMMATION	INFLAMMATION
0.490627498	0.240465195	0.354387593	0.687199075	0.309561938
0.397906122	0.240215309	0.505260744	1.036270039	0.255837562
0.511604195	0.281479157	0.50662845	1.147584192	0.077928778
0.547640102	0.264291146	0.526863083	0.72920684	0.178327364
0.448630621	0.406464145	0.615742903	0.61327243	0.12342444
0.532738703	0.237846055	0.602862125	0.900189099	0.288691393
0.368158777	0.154341721	0.382041164	1.034834459	0.29441029
0.423842452	0.157224723	0.370925151	0.827367389	0.102856153
0.383420665	0.18439865	0.334737016	0.497959391	0.167449735
0.522933718	0.283299508	0.445500822	0.572441432	0.10819457
0.43077308	0.222518933	0.472177107	0.556865184	0.235092432
0.557328564	0.544423029	0.709512497	1.450650522	0.260742923
0.407282011	0.205413086	0.45009455	1.143455325	0.064870214
0.383340943	0.167647166	0.331045173	0.802570482	0.06743774
0.402343603	0.13302783	0.240265265	0.362587225	0.293676555
0.346157377	0.261014164	0.610304021	0.438454791	0.179070551
0.373764123	0.289051808	0.477277424	0.620111694	0.218650719
0.424606982	0.202753679	0.50375704	0.654152994	0.162555018
0.363593925	0.210384453	0.444606209	0.624901193	0.10309884
0.434040027	0.27100662	0.466031701	0.805412629	0.13439953
0.417312488	0.321301584	0.332747562	0.937636086	0.081644528
1.504412293	0.230461873	0.588290237	0.73163703	0.125008665
0.321479801	0.212391804	0.363619128	0.472799364	0.088676768
0.370026372	0.116193398	0.415638154	0.761227718	0.077632255
0.411567224	0.259570799	0.308362664	0.790698723	0.116265906
0.529205514	0.231438374	0.431340773	0.62711416	0.048724798
0.424077545	0.288671383	0.514449012	0.932386486	0.214566983
0.380033894	0.19185656	0.478204629	0.835609037	0.095285467
0.487542537	0.734126202	0.468753056	0.662595357	0.434612026
0.401869781	0.166801018	0.376363858	0.474802685	0.128460792
0.419196914	0.245024854	0.383925955	0.496787232	0.222626926
0.464194071	0.167194582	0.418587171	1.116822568	0.104226931
0.34077703	1.141317352	0.35060051	0.480397317	0.121987058
0.359808207	0.252595352	0.368771738	0.986984956	0.186882062
0.418442125	0.222673225	0.699501958	0.583215109	0.187323006

Q8WV07,LTO1,INFLAMMATION	P01584,IL1B,INFLAMMATION	O43707,ACTN4,INFLAMMATION	P55773,CCL23,INFLAMMATION	P29350,PTPN6,INFLAMMATION
Q8WV07	P01584	O43707	P55773	P29350
LTO1	IL1B	ACTN4	CCL23	PTPN6
INFLAMMATION	INFLAMMATION	INFLAMMATION	INFLAMMATION	INFLAMMATION
0.667527881	0.594438721	0.959264119	0.105637942	0.573116366
0.690685097	0.479465861	0.802626113	0.180491149	0.578384181
0.666741762	0.800348366	0.78736255	0.259480854	0.672543738
1.084026481	0.807480878	1.025054062	0.189320166	0.913578117
0.850568894	0.800736791	1.540074348	0.222102879	1.448038539
0.815694031	0.587923357	1.049207481	0.211583642	0.560932858
1.116126075	0.681129017	0.98677974	0.211642314	0.609120684
0.721764454	0.539614118	0.892175849	0.233419986	0.640646313
20.64470645	0.481297222	1.098701895	0.254828757	0.790808345
0.954224081	0.635339667	1.247984486	0.172467927	0.618437625
0.841129594	0.384112282	0.912692006	0.107305803	0.584550674
0.707498993	1.004028346	0.805524291	0.208555028	0.672870138
0.697178507	0.8861974	1.005142466	0.10256426	0.636750459
0.696550569	0.325470795	0.867117857	0.166581489	0.537672653
4.773673847	0.683683257	1.064812934	0.107134868	0.703245344
0.871214578	0.544611745	0.794214167	0.19185656	0.808825283
0.938481363	0.560816227	0.839033314	0.174875489	0.847156255
1.212680921	0.940043876	1.16812932	0.184475355	0.622221435
0.427027238	0.679619899	0.816316204	0.187348976	0.827138026
0.598324482	0.462395736	0.936272247	0.135775963	0.719167634
0.559224704	0.593944487	0.892732591	0.173390889	0.63248361
0.801403102	0.611574433	0.864776965	0.150329495	0.841537811
0.655605562	0.285408459	0.902062938	0.145763357	0.643762301
0.895956131	0.534514133	0.920570426	0.163901391	0.561399623
0.708431369	0.618480494	0.956011581	0.206126229	0.485418164
0.602152161	0.513095757	0.678725443	0.256139206	0.529278883
1.343689688	0.657334677	1.00291546	0.179991415	0.759014841
0.917321908	0.61869488	1.114966219	0.15621447	0.897696702
0.468330857	0.701589956	0.903063912	0.149373901	0.721214346
0.966271155	0.492501492	1.152366799	0.22047699	0.670681634
4.954364453	0.713606173	0.983911186	0.30155645	0.616939105
0.867899561	0.647072827	0.846334569	0.194480849	0.543781886
1.125604499	0.814451108	1.147027516	0.124093547	0.462075339
0.710201346	0.836536271	1.049352943	0.150569347	0.510293793
1.534958872	0.566913292	0.926074395	0.230829577	1.056651769

P78362,SRPK2,INFLAMMATION	P09341,CXCL1,INFLAMMATION	Q03403,TFF2,INFLAMMATION	Q9H008,LHPP,INFLAMMATION	Q9H3U7,SMOC2,INFLAMMATION
P78362	P09341	Q03403	Q9H008	Q9H3U7
SRPK2	CXCL1	TFF2	LHPP	SMOC2
INFLAMMATION	INFLAMMATION	INFLAMMATION	INFLAMMATION	INFLAMMATION
0.979216654	0.557058212	0.381644155	0.675206174	0.454515059
1.045505028	0.619381415	0.278567721	0.798409062	0.503338201
0.852221291	0.885092413	0.565304466	0.695730272	0.570026146
2.021603406	0.61339997	0.380139277	0.441535089	0.332332663
0.825705946	1.206393031	0.391097441	0.824962245	0.539352359
1.088619663	0.984729921	0.269433286	0.598656355	0.489201252
0.788181613	0.681459583	0.463518877	0.692074858	0.320079015
0.821253774	0.856663188	0.378614088	0.398568611	0.426287897
0.697516863	0.615230956	0.242944723	0.620412647	0.341202471
1.035193167	0.718320702	0.214150953	0.838568184	0.27177789
0.86160588	0.575584641	0.368516214	0.575026361	0.293229062
1.028826708	0.816825607	0.500416061	1.102287073	0.773782497
1.146550579	0.541112322	0.267257067	0.326374444	0.371002291
0.710053679	0.664481068	0.259696774	0.628027658	0.355913855
0.682263055	0.951845945	0.256885969	0.430295602	0.192029518
0.905194673	0.543141498	0.172850898	0.644610682	0.237730679
0.688486371	1.878915257	0.311931251	1.741463216	0.420856411
0.770838214	0.697033548	0.5488942	0.635383707	0.377644322
0.797358265	0.664342907	0.322886718	0.439306576	0.305427104
0.710940141	0.635868347	0.382385574	0.415407739	0.338258947
0.937051341	0.901375411	0.312125904	0.483638165	0.377696678
0.991373087	1.255968255	0.295186777	1.659444064	0.43283828
0.889643971	1.27570964	0.325764206	0.727994774	0.454105684
0.753719697	0.718968266	0.244210985	0.447760758	0.269395937
0.838684443	0.741490441	0.310959802	0.446706762	0.429848447
0.890878134	0.731839911	0.390366187	0.870007655	0.353724977
0.952902162	1.168939287	0.264988196	0.931223906	0.373220465
0.731484906	0.984525173	0.241233137	1.372589287	0.357050489
0.678960712	1.00430676	0.336971854	0.825705946	0.627244578
0.960395135	0.686104389	0.270237539	0.505190705	0.36478037
0.793168891	0.670495707	0.414918532	0.335247853	0.296252657
0.77271055	0.594562344	1.57385213	0.821083017	0.365235778
0.689345907	0.444513765	0.199450134	0.367827181	0.380983388
0.935493802	0.973599276	0.410200171	0.460636297	0.292214566
0.778462797	0.816146473	0.28618104	1.174299167	0.281732909

Q9UII2,ATP5IF1,INFLAMMATION	Q5R372,RABGAP1L,INFLAMMATION	Q13459,MYO9B,INFLAMMATION	Q12918,KLRB1,INFLAMMATION	O43639,NCK2,INFLAMMATION
Q9UII2	Q5R372	Q13459	Q12918	O43639
ATP5IF1	RABGAP1L	MYO9B	KLRB1	NCK2
INFLAMMATION	INFLAMMATION	INFLAMMATION	INFLAMMATION	INFLAMMATION
0.539876004	1.287078458	0.873512347	0.61839476	0.993781093
0.379823217	1.044346168	1.156527844	0.721414337	0.854942916
0.454074209	1.295761322	0.769236967	0.665172302	1.003124032
0.499307333	0.913958143	0.761386028	0.553325378	0.939067001
0.40522638	1.195736912	0.872544127	0.802570482	0.828859801
0.346085403	1.228076132	0.968148327	0.640246781	0.774909642
0.35167142	0.933938852	0.460891798	0.518709968	0.927616251
0.319126429	0.725325261	0.710693791	0.588208689	0.95013209
0.464644746	1.318410831	0.841246207	0.533773653	1.026974244
0.312320679	1.027543877	0.79454454	0.454798689	0.784638483
0.492740513	0.757910816	0.640513108	0.462716356	0.882458293
0.544687249	1.18640781	0.564638732	0.988217151	0.854409741
0.376781491	1.047173142	0.569118113	0.348879323	0.827195361
0.35507606	1.164975811	0.862322841	0.370052021	0.843406473
0.332493951	1.108570099	0.667389086	0.382041164	0.918594468
0.475066045	1.053069023	0.504141282	0.491989694	0.756179163
0.412709914	2.474264957	0.814620486	0.513914406	0.986095993
0.436211588	1.678764662	1.146232732	0.547716026	0.897012503
0.422141918	0.933615229	0.987258644	0.472832137	0.917512679
0.287473325	0.767053973	0.506733811	0.562646229	0.873633449
0.434642152	0.893537387	0.733770088	0.416647723	0.995780728
0.53329289	1.239793633	0.60336378	0.400562703	0.813773948
0.496787232	0.747165632	0.705589012	0.392237669	0.902375623
0.435456345	0.937311183	1.01995111	0.32894083	0.865496566
0.43455178	0.76286517	0.654788096	0.483571123	0.723166624
0.325019909	1.078031998	0.483202559	0.650175005	0.956541853
0.286896048	0.95561407	1.136186763	0.616511623	0.944812528
0.416705486	1.653817453	0.889458994	0.428777171	0.812590273
0.405535467	1.092550526	0.816203046	0.674691551	0.942065973
0.394911148	0.775877073	0.749188157	0.348058087	1.006257823
0.478171483	0.661723307	0.733973561	0.570698233	0.83058516
0.342505723	1.207396898	1.026903062	0.426760927	0.825362616
0.452377788	0.977182546	5.35133928	0.487711535	0.815072332
0.374827836	0.849567216	0.992198031	0.483068605	0.795205698
0.50575129	1.274030663	0.409404822	0.529022138	0.973059547

Q9Y266,NUDC,INFLAMMATION	Q9HC38,GLOD4,INFLAMMATION	P20809,IL11,INFLAMMATION	O14836,TNFRSF13B,INFLAMMATION	O75462,CRLF1,INFLAMMATION
Q9Y266	Q9HC38	P20809	O14836	O75462
NUDC	GLOD4	IL11	TNFRSF13B	CRLF1
INFLAMMATION	INFLAMMATION	INFLAMMATION	INFLAMMATION	INFLAMMATION
0.496959435	0.69011084	1.25989194	0.307082882	0.345749723
0.501562016	0.588738957	0.640823962	0.366834187	0.465902507
0.423548769	0.716778863	0.867478556	0.382756823	0.358936365
0.59139748	0.577823186	0.891619454	0.293432383	0.308490935
0.793168891	0.654833484	1.449946833	0.381882311	0.382571153
0.441504485	0.666418336	0.802347993	0.258350217	0.446211623
0.352770053	0.65747138	0.678255149	0.380983388	0.36180895
0.373039421	0.473192791	0.770090551	0.383234673	0.373091139
0.315497395	0.512491506	0.789329733	0.242288864	0.342434508
0.38734741	0.637280314	1.396678532	0.25396472	0.30477152
0.418819349	0.521196753	0.649004326	0.282515123	0.279418607
0.832025702	1.087639159	0.959264119	0.339174591	0.516485589
0.39033913	0.39069102	0.803182644	0.249584457	0.309325998
0.303990891	0.511533277	0.762812294	0.167635546	0.268948156
0.232531811	0.412795744	0.864417389	0.19946396	0.248514167
0.369898153	0.639758804	1.197146739	0.339692201	0.260760997
0.612465294	1.326845141	0.680091139	0.292761957	0.424754165
0.391233008	0.583579051	1.597814581	0.285645954	0.377932371
0.532480279	0.516163489	0.966673099	0.263376768	0.365818515
0.352281349	0.424106941	1.135163416	0.273554463	0.301180444
0.376077005	0.465741066	1.018608739	0.219866544	0.351817707
0.572600169	0.70285549	1.245910123	0.247637207	0.392156114
0.425490846	0.561633151	0.844283837	0.270574915	0.336038864
0.252542832	0.391477148	0.834277938	0.185925944	0.319126429
0.35697625	0.49768334	0.399564413	0.276087983	0.385125352
0.334690614	0.730572827	1.082674823	0.328257527	0.306211421
0.324884766	0.627375023	1.012414447	0.300804906	0.383234673
0.506523111	1.005351502	0.982956856	0.218832664	0.296663636
0.318154625	0.841421157	0.580110646	0.424312769	0.373582816
0.352183689	0.39025797	0.624468195	0.234555299	0.34455352
0.267664924	0.458883552	1.429984986	0.257171023	0.268705918
0.361658509	0.497786842	1.203970464	0.341533737	0.379402213
0.380587478	0.420156876	1.35434942	0.225703391	0.419080704
0.277565468	0.417659744	1.060173217	0.27190979	0.320856474
0.441994401	1.179356592	0.706176149	0.308961719	0.308042219

P09919,CSF3,INFLAMMATION	Q14242,SELPLG,INFLAMMATION	P42768,WAS,INFLAMMATION	Q9UNK0,STX8,INFLAMMATION	Q92609,TBC1D5,INFLAMMATION
P09919	Q14242	P42768	Q9UNK0	Q92609
CSF3	SELPLG	WAS	STX8	TBC1D5
INFLAMMATION	INFLAMMATION	INFLAMMATION	INFLAMMATION	INFLAMMATION
0.557212682	1.066437931	1.074525708	0.84188787	0.715835501
0.67245051	0.893475454	1.052193469	0.739181216	0.67604913
0.971240172	0.817675319	0.990068329	0.666418336	0.905947906
0.75612675	0.463872427	1.017620755	0.776468875	0.701541327
0.870188587	0.988217151	1.267600382	0.795205698	0.975693553
0.846686622	1.032255425	0.910859222	0.741490441	0.644610682
0.520258307	0.98173122	1.201469479	0.705637922	0.671612041
0.483705216	0.85862495	0.65469733	0.730623468	0.763552891
0.596833326	0.785563607	1.204304322	0.607518396	0.645907734
0.75236258	1.019173732	1.053142018	0.699356515	0.657106902
0.394829037	0.93212801	1.101065275	0.779866995	0.781869643
0.805468458	1.033186	0.715637057	0.739488696	1.099844832
0.666603132	1.294145654	1.216806698	0.850745783	0.859935286
0.542464262	0.689776078	1.098397312	0.638252859	0.636706325
0.435667681	0.829722032	0.577182715	0.777330484	0.720015565
0.513095757	0.837929052	0.411225034	0.749811574	0.765619776
0.84077985	0.947173091	0.743446071	0.654651951	0.768544126
0.615828269	1.086734863	0.734838948	0.882458293	0.832083375
0.726079789	0.803962436	1.421486108	0.693611633	0.600693101
0.501006074	0.936661713	0.748098426	0.776953413	0.647521499
1.146312186	0.833815444	0.82393361	0.586214273	0.733770088
0.69964743	0.905885113	0.63366841	0.746285727	0.797026722
0.659159725	0.43615112	0.806194586	0.601484723	0.621962714
0.60023527	0.793993994	0.767213494	0.640291161	0.596089142
0.630688704	0.99212926	1.0138892	0.863519104	0.869645904
0.344290911	1.006815966	0.570935629	0.708824315	0.457676461
0.742673497	0.86357896	1.348635052	0.917321908	0.684015063
0.578905593	0.802848679	1.074227828	0.797026722	0.658200944
0.573633029	0.79603292	0.98684814	0.700375246	0.877456798
0.999168569	1.113961985	1.362919167	0.764824159	1.130687303
0.40410441	0.857732683	0.707498993	0.764241232	0.759646434
0.975625925	1.04478059	1.148698355	0.665818102	0.732093591
0.482666967	0.818752891	1.006536856	0.646221206	0.662733155
0.819775057	1.023279317	0.834740688	0.872725587	0.829779546
0.623343803	0.970432653	1.494642033	0.595799988	0.667250321

Q96P31,FCRL3,INFLAMMATION	Q04759,PRKCQ,INFLAMMATION	P56470,LGALS4,INFLAMMATION	O00585,CCL21,INFLAMMATION	Q13651,IL10RA,INFLAMMATION
Q96P31	Q04759	P56470	O00585	Q13651
FCRL3	PRKCQ	LGALS4	CCL21	IL10RA
INFLAMMATION	INFLAMMATION	INFLAMMATION	INFLAMMATION	INFLAMMATION
0.557560398	0.769610293	0.349339092	0.734686159	0.849861705
0.495480436	0.922550634	0.293168093	0.824562068	0.847273704
0.513024632	0.706861759	0.399398273	0.824790717	0.737594601
0.685153905	0.829204586	0.29610895	0.766363099	1.003193566
0.673430048	0.874784765	0.625941615	0.953100333	1.146948012
0.569907625	0.896204577	0.288411381	0.706323009	0.735654363
0.531153222	0.831506819	0.336948498	0.70730286	0.941739534
0.574428805	0.462139401	0.292538822	0.803182644	0.647880661
0.695682049	0.952241889	0.299556491	0.608023926	0.960927838
0.60667678	0.872423175	0.167996141	0.533958677	0.77981294
0.481030408	0.943045968	0.268873598	0.597992792	0.678396203
0.444544577	0.56405197	0.436665362	0.75859407	0.936921447
0.466322517	1.04630249	0.210209533	0.923318309	0.831622098
0.508281631	0.701589956	0.214106426	0.540662425	0.682972787
0.344601288	0.825362616	0.146614531	0.374957764	0.674598025
0.606424523	1.419812086	0.212377083	0.790863161	0.809442217
0.542464262	0.715289912	0.38969032	0.579226695	0.705051233
0.749292024	1.131236049	0.419865746	0.700423794	0.982820599
0.444945333	0.787089719	0.306933921	0.647656162	0.82857259
0.406295136	0.733363312	0.272929454	1.063043033	0.770945083
0.537411836	1.036844828	0.249342377	0.783714449	0.736623843
0.415955183	0.776791866	0.42199564	0.593203905	0.609881137
0.294818712	0.95972967	0.433919703	0.457422742	0.821481505
0.321725011	0.871939535	0.179493064	0.496477417	0.889458994
0.49103576	1.086207703	0.364881522	0.63971446	0.776092221
0.352476749	0.644074732	0.314340485	0.949473737	0.612380394
0.4430987	0.885337846	0.22256521	0.817052111	0.93400359
0.74597542	0.825992164	0.279942028	0.673896996	0.943634454
0.591192552	0.869706186	0.389366319	0.905445681	1.276682691
0.485586427	0.834277938	0.39829244	0.537225615	1.186736798
0.408073237	0.759593782	0.199284305	0.627070693	0.872846581
0.576662855	1.002984979	0.400118711	0.926909248	0.771533123
0.479332943	0.994883843	0.421615555	0.502501567	0.768224564
0.395513817	1.083951345	0.376155216	0.484846509	0.813830356
0.492945481	0.779488692	0.26072485	0.447822835	0.717474767

O95760,IL33,INFLAMMATION	Q13291,SLAMF1,INFLAMMATION	P42702,LIFR,INFLAMMATION	P05231,IL6,INFLAMMATION	Q6UXB4,CLEC4G,INFLAMMATION
O95760	Q13291	P42702	P05231	Q6UXB4
IL33	SLAMF1	LIFR	IL6	CLEC4G
INFLAMMATION	INFLAMMATION	INFLAMMATION	INFLAMMATION	INFLAMMATION
1.176662032	0.265043304	0.579347155	0.530196849	0.46274843
0.800903317	0.345797657	0.440678984	0.623214195	0.564991082
0.900501135	0.944092419	0.615870957	0.731738463	0.493492478
0.946648012	0.362888942	0.589719171	0.550074908	0.410313919
1.539540692	0.542501864	0.774104371	1.261727185	0.495377415
0.858446422	0.345725758	0.659342508	0.438971749	0.474342158
1.074600191	0.507120324	0.505646133	0.43080294	0.307700779
0.622998243	0.426760927	0.437756347	0.261938495	0.371182346
1.383957845	0.342220953	0.462491899	0.373712312	0.684015063
0.895521516	0.999792077	0.403824403	0.707646129	0.339456826
0.628201808	0.316022679	0.476814495	0.301431063	0.248290333
0.653654418	0.354903818	0.683446351	0.983433905	0.470478271
0.614463832	0.223989033	0.588086387	0.392101754	0.342648197
0.459934397	0.259301058	0.30036737	0.291082498	0.180942097
0.745510202	0.301472853	0.342434508	0.303107194	0.341344403
0.746440929	0.755183946	0.454515059	0.264108017	0.546616154
0.988422666	0.319635599	0.552061156	0.431939153	0.321903462
1.209994089	0.485822092	0.601026289	0.826679491	0.380218333
0.515126977	0.683162173	0.485822092	1.481337116	0.476979775
0.64166847	0.596957447	0.515519891	0.535366956	0.442147611
0.682073917	0.595717398	0.371208075	0.920251435	0.261122739
1.055553718	0.325290366	0.34992072	1.153485605	0.448941696
0.467163673	0.449689156	0.401479993	0.757438154	0.314798375
0.761597158	0.129830388	0.331458464	0.365134527	0.215431333
0.591192552	0.509657515	0.453382308	0.474375038	0.452785606
0.418036263	0.385900281	0.615145672	0.324569648	0.428658305
1.207396898	0.784040454	0.539015999	0.377749042	0.818752891
0.897758928	0.654379746	0.291203581	0.356852553	0.833295446
1.217312859	0.480031172	0.421557111	0.542276291	0.385499261
1.433458363	0.757490657	0.542501864	0.661906801	0.55953489
0.932386486	0.603698449	0.394282067	0.407253782	0.430176315
0.761069442	0.419691166	0.394172764	0.521955964	0.264639441
0.452660084	0.848390282	0.382624192	0.273383864	0.329694106
1.426520026	0.434792813	0.448164413	0.420827241	0.315453661
0.521702771	0.320545264	0.529682592	0.316658563	0.87175824

P01591,JCHAIN,INFLAMMATION	P13725,OSM,INFLAMMATION	Q9NYY1,IL20,INFLAMMATION	Q8IVG5,SAMD9L,INFLAMMATION	P11684,SCGB1A1,INFLAMMATION
P01591	P13725	Q9NYY1	Q8IVG5	P11684
JCHAIN	OSM	IL20	SAMD9L	SCGB1A1
INFLAMMATION	INFLAMMATION	INFLAMMATION	INFLAMMATION	INFLAMMATION
0.367444943	0.70061802	0.488185043	0.938286231	0.236793273
0.454011265	0.413569013	0.322774833	0.850450988	0.247929183
0.205983403	0.905257419	0.316724418	0.827539453	0.260002969
0.369744348	0.34512718	0.752779894	0.864057963	0.127812425
0.220752243	0.59423274	0.511037123	0.780353653	0.242540907
0.361834029	0.516951199	0.423783699	0.842822071	0.328599001
0.448755025	0.487677731	0.419225971	0.804742985	0.335526821
0.435335628	0.263723859	0.379717922	0.616853585	0.438789224
0.284401307	0.31992375	0.635603952	0.761755544	0.144927171
0.696357471	0.665033997	0.632308273	0.663376587	0.289031773
0.267683478	0.308854659	0.438910899	0.638739688	0.241802321
0.21915143	1.627999211	0.572798651	1.107034359	0.332770627
0.29278225	0.244075603	0.344267047	0.79366385	0.159253833
0.210136692	0.327348665	0.437362066	0.609923412	0.166569943
0.509127889	0.505225723	0.308362664	0.515698588	0.198388458
0.493082173	0.873330724	0.35539616	0.913008376	0.237302631
0.397354889	1.016492803	0.245279744	0.876848802	0.236744038
1.059365184	0.151469581	0.570856486	0.723567744	0.239500405
0.658337827	0.385980535	0.579748867	0.762812294	0.173042702
0.55091437	0.435818698	0.26960142	0.861247622	0.248479718
0.298851357	0.532332665	0.41339705	0.699356515	0.17689927
0.224938108	0.78561806	0.451312925	1.067325338	0.20656962
0.175567778	0.446582926	0.294287873	0.729459608	0.251390145
0.523949619	0.199339566	0.272004043	0.77196107	0.25396472
0.291304522	0.318551823	0.328667338	0.758015892	0.283712181
0.252385337	0.394364064	0.515627102	0.846980113	0.281440138
0.535478294	0.464226247	0.649319301	0.865256632	0.28618104
1.733754948	0.464934697	0.446087924	0.801292012	0.314732922
0.659479628	0.473061612	0.445315582	0.661264794	0.240248612
0.397547733	0.24696868	0.52624262	4.002218686	0.149684838
0.594397519	0.241065985	0.340493698	0.70803864	0.267497998
0.539240216	0.358961245	0.421790937	0.723567744	0.228267916
0.458819941	0.261376257	0.385873533	0.543932675	0.182617889
0.219942757	0.255146897	0.574548266	0.549427109	0.224066675
0.185141472	0.850804754	0.476318999	0.728045237	0.353823064

P78310,CXADR,INFLAMMATION	P35625,TIMP3,INFLAMMATION	O14867,BACH1,INFLAMMATION	Q9UKU9,ANGPTL2,INFLAMMATION	Q9NWZ3,IRAK4,INFLAMMATION
P78310	P35625	O14867	Q9UKU9	Q9NWZ3
CXADR	TIMP3	BACH1	ANGPTL2	IRAK4
INFLAMMATION	INFLAMMATION	INFLAMMATION	INFLAMMATION	INFLAMMATION
0.666418336	0.380824975	0.902438173	0.39763041	0.925817668
0.551640391	0.544762764	0.865076726	0.513451531	0.605962323
0.584348119	0.620068712	1.160060462	0.458533804	0.593450663
0.504316034	0.521702771	1.43435288	0.610981245	0.289432734
0.577703043	0.971576836	1.197644722	0.696647138	0.609120684
0.523187508	0.438029518	0.761174956	0.522643822	0.625551254
0.333602036	0.379560035	0.762812294	0.486260059	0.600401714
0.377696678	0.398513361	0.71227191	0.621058038	0.745303531
0.324209889	0.405170207	0.865976632	0.555708418	0.523078726
0.275667291	0.508175948	0.997991892	0.340375712	0.663330607
0.263669024	0.415695778	0.843874287	0.398623868	0.695200006
0.532664855	0.601401345	0.894652918	0.558063038	0.733566672
0.414573556	0.39801646	1.175439268	0.284223944	0.673383371
0.270143898	0.429729284	0.665818102	0.234068062	0.445995173
0.260147185	0.343575722	0.677362489	0.288591357	0.648240022
0.304539232	0.491444362	0.725928821	0.267405306	0.388019214
0.384671806	1.153565561	1.097560145	0.352623371	0.90909313
0.570896056	0.486496051	0.823819397	0.409660302	0.58204395
0.331573359	0.54733651	1.764671934	0.462459842	0.642068889
0.484745698	0.353063601	0.770998522	0.388422856	0.580271509
0.383925955	0.403796413	0.680185426	0.380244688	0.552788689
0.544838289	0.282632642	0.779704843	0.40418845	0.876970367
0.344696845	0.483604643	0.993987766	0.307317111	0.340116287
0.382200084	0.32687252	0.918785504	0.261756996	0.586417475
0.387965427	0.295739735	0.903752727	0.269807059	0.656697105
0.398458119	0.521196753	0.83225642	0.432868283	0.705002364
0.376885971	0.37837797	1.022712047	0.36645298	0.611489656
0.444113398	0.696067924	0.797579371	0.424665849	0.800736791
0.330311705	0.471261588	0.65715245	0.72718785	0.635119513
0.408271283	0.511568735	0.85174885	0.543970379	0.975287857
0.400396148	0.589269704	0.964999433	0.344219325	0.554900112
0.506347593	0.371002291	0.877335165	0.46664586	0.381723524
0.337907435	0.471588355	0.833468742	0.310701261	0.480031172
0.35963367	0.354092944	0.92665229	0.290196093	0.596006512
0.420448208	0.489845944	0.96339544	0.480297432	0.645907734

Q6GTX8,LAIR1,INFLAMMATION	Q96AX2,RAB37,INFLAMMATION	P29279,CCN2,INFLAMMATION	P21860,ERBB3,INFLAMMATION	P09326,CD48,INFLAMMATION
Q6GTX8	Q96AX2	P29279	P21860	P09326
LAIR1	RAB37	CCN2	ERBB3	CD48
INFLAMMATION	INFLAMMATION	INFLAMMATION	INFLAMMATION	INFLAMMATION
0.339433297	0.603907711	0.621876498	0.460604369	0.47371787
0.321145726	1.328962135	0.704318557	0.372806779	0.39066394
0.50389673	1.207899145	0.669798941	0.357892943	0.418964526
0.34231585	1.6545054	0.457454449	0.413339745	0.442730294
0.362361102	2.371828999	0.531926936	0.474769776	0.444513765
0.34642141	0.985549337	0.572957487	0.332955205	0.338822127
0.266332427	1.185010629	0.469566047	0.39975833	0.388530564
0.284125456	0.824504916	0.763658749	0.31057207	0.356852553
0.226377111	1.093914514	0.548970298	0.395980145	0.307828775
0.205512778	0.867899561	0.578704994	0.275954057	0.378850354
0.273042986	0.612168196	0.499792099	0.337509496	0.283142457
0.649184293	1.059805853	1.143217574	0.457137476	0.510258424
0.195643624	0.870067961	0.470152274	0.317339717	0.255979468
0.254987777	0.874239216	0.517668343	0.26312131	0.264767877
0.197496638	0.907456253	0.328644558	0.309948408	0.196731516
0.269377265	0.697807012	0.466807615	0.295227701	0.410086456
0.339974866	1.822223601	1.31084763	0.406745982	0.344267047
0.343694817	2.62242225	0.553248677	0.438302861	0.466613515
0.274409056	0.747528247	0.452942556	0.381379711	0.408582693
0.367903677	0.743806881	0.469891638	0.328599001	0.298147883
0.277161735	0.808488972	0.5318532	0.33008283	0.262192805
0.367776193	1.282003322	0.829664522	0.330517829	0.298561491
0.281186649	0.858684467	0.635207565	0.29889279	0.301974786
0.193338385	0.726633608	0.385793302	0.30283419	0.264657786
0.386355275	0.785454712	0.452816992	0.322215991	0.359085674
0.304328215	1.069472955	0.477873278	0.455303356	0.424048151
0.265631842	1.052558194	0.391857224	0.349242248	0.349944976
0.19484516	0.943176711	0.608108222	0.353945711	0.245960747
0.275667291	0.762759422	0.653065681	0.372135518	0.326080483
0.355346895	0.897261243	0.388611365	0.408611015	0.316395284
0.234734207	0.725124186	0.379481116	0.274789732	0.320411981
0.232338477	1.217228484	0.570105174	0.328507907	0.343099755
0.182807859	1.347887417	0.46364741	0.288671383	0.342600699
0.205498533	1.146789023	0.391830064	0.283712181	0.355593289
0.253560162	1.046882844	0.438302861	0.505435884	0.443805669

P05412,JUN,INFLAMMATION	O75563,SKAP2,INFLAMMATION	Q9NQ25,SLAMF7,INFLAMMATION	Q9Y6K9,IKBKG,INFLAMMATION	Q99983,OMD,INFLAMMATION
P05412	O75563	Q9NQ25	Q9Y6K9	Q99983
JUN	SKAP2	SLAMF7	IKBKG	OMD
INFLAMMATION	INFLAMMATION	INFLAMMATION	INFLAMMATION	INFLAMMATION
0.33662168	0.360757177	0.410940094	0.665172302	2.26451171
0.437908088	0.479465861	0.475824018	0.758331207	2.382209014
0.443344474	0.485822092	0.861307322	1.041238095	3.134423241
0.503442878	0.393353957	0.346325375	0.770464292	1.848301356
0.569157562	0.442270218	0.686104389	0.807760778	1.905408064
0.461147442	0.336271869	0.45875634	0.871395761	2.354302987
0.438059881	0.411824053	0.590864816	0.59563482	2.302976995
0.735960377	0.417630795	0.441290318	0.40228783	2.533970976
0.539128096	0.558527414	0.46836332	0.83294896	2.237053873
0.534106742	0.459010799	0.396859432	0.870369556	1.765038925
0.410285479	0.424518697	0.408724322	0.5488942	1.776946197
0.408809322	0.488896167	0.484309095	1.02129525	3.448096139
0.629727685	0.350430439	0.472996037	0.682263055	2.775408248
0.332424818	0.297796768	0.378037171	0.594315124	2.091299952
0.340234183	0.398789685	0.446551972	0.535849588	1.586007947
0.324682155	0.543631139	0.345079338	0.938481363	1.209490971
0.614932516	0.515126977	0.320745293	1.085229372	2.272216022
0.511533277	0.40570416	0.665264521	0.748202141	2.226534552
0.466904695	0.4499074	0.537598121	0.720664657	1.778301568
1.148141138	0.376233443	0.41754396	0.755602825	2.043015288
3.950951126	0.382438587	0.395952699	0.449720327	2.66013599
0.650355297	0.38616786	0.335178148	0.672730233	1.900922876
0.472340779	0.46577335	0.355199142	0.661769176	2.374460901
0.416532219	0.360307353	0.332701436	0.450562766	1.788685817
0.436211588	0.360232437	0.525732198	0.474144925	2.398612323
0.32585454	0.394746943	0.510966283	0.627157629	1.965641197
0.381670609	0.404636958	0.516843713	0.68164855	1.580959051
0.379770566	0.508316864	0.395486403	1.106574052	1.831086569
0.424548123	0.487779151	0.40801667	0.544272104	2.391473823
0.440099	0.440892854	0.426642621	0.574827106	1.35999373
0.426022048	0.411196531	0.283751515	0.521269011	0.958333695
0.392074576	0.316680513	0.476285984	0.61595634	2.425193734
0.433979861	0.328849641	0.400784883	0.434280777	2.15531689
0.314907495	0.346709675	0.479166848	0.61668258	1.562114279
0.476120945	0.589188019	0.375035742	0.976302412	1.876182281

O15169,AXIN1,INFLAMMATION	P21709,EPHA1,INFLAMMATION	P23582,NPPC,INFLAMMATION	P20849,COL9A1,INFLAMMATION	O94856,NFASC,INFLAMMATION
O15169	P21709	P23582	P20849	O94856
AXIN1	EPHA1	NPPC	COL9A1	NFASC
INFLAMMATION	INFLAMMATION	INFLAMMATION	INFLAMMATION	INFLAMMATION
1.25840822	0.354928419	0.779542724	83.4187622	1.029682818
0.935493802	0.372755101	1.15765069	67.06555126	0.660944025
1.288238756	0.306402506	0.994194481	142.3599973	0.759225314
1.235761215	0.134660628	0.83398885	70.11251104	1.117209696
1.030182544	0.254669837	1.217650417	77.10753804	1.0138892
1.18558574	0.26230187	1.986047324	69.17122015	0.889089155
1.172266024	0.284361883	0.760173164	55.24990198	0.649634429
1.0428994	0.441106829	1.034332476	98.12167794	1.349570179
0.914211581	0.176568513	0.479465861	59.94219462	0.445840629
1.271913007	0.139428752	0.682783453	34.94950216	0.560311108
1.479695171	0.226330042	0.656833675	83.08407084	0.660714999
1.093611258	0.591643486	1.804000827	134.9237022	1.190196681
1.452662945	0.176997391	1.289132004	96.91177084	0.704562698
0.886996305	0.182795188	0.835319487	50.46110872	0.87109381
1.1076484	0.109895179	0.579708683	62.2283462	0.631344786
1.002845945	0.151921715	0.738566638	25.29008534	0.736879182
1.023563069	0.150955999	0.626201991	47.37650931	0.73933494
1.26190211	0.14455596	0.727692073	72.69075576	0.839265976
0.665126197	0.22147258	0.833179934	81.18267652	0.607392079
0.930127244	0.239268105	1.100531165	82.91722906	1.177641159
1.011222171	0.161074498	1.20689486	104.3509555	1.276859689
1.07780785	0.234149198	0.945467649	91.56980213	0.758962232
0.876848802	0.221610785	0.908589161	107.0024485	0.535107257
0.803906711	0.19797635	1.099692372	38.48435066	0.513309192
1.230120802	0.298809931	0.907770808	111.1296518	0.5893514
0.853107824	0.243281749	0.70705777	79.48991885	0.935040008
0.953100333	0.411909698	0.678067123	18.7536564	0.467941472
0.816768991	0.272570248	1.115739322	106.2412341	0.424401012
1.246774022	0.358339752	1.131785061	101.4763695	0.698387673
0.987806249	0.341770552	0.473947775	69.68614776	0.537002236
0.888842681	0.229744145	0.769823704	6.366497948	0.407762215
1.005142466	0.27283488	1.129590609	109.6452877	0.957470538
0.861307322	0.226455581	0.738106039	49.13230437	0.6341957
1.104198847	0.228806509	0.87751762	49.55298291	0.865796576
1.062674674	0.318838997	0.554938576	73.71058994	0.622135183

Q8WTT0,CLEC4C,INFLAMMATION	O95971,CD160,INFLAMMATION	P43489,TNFRSF4,INFLAMMATION	P28845,HSD11B1,INFLAMMATION	O43736,ITM2A,INFLAMMATION
Q8WTT0	O95971	P43489	P28845	O43736
CLEC4C	CD160	TNFRSF4	HSD11B1	ITM2A
INFLAMMATION	INFLAMMATION	INFLAMMATION	INFLAMMATION	INFLAMMATION
0.630688704	0.38947429	0.65646955	0.308020868	1.240567298
1.1498136	0.564169274	0.836014575	0.465418351	1.147584192
0.855891604	0.541487523	0.875087995	0.475692109	1.819446967
0.764294207	0.363972158	0.543518106	0.306147753	1.030468211
1.464694719	0.593121675	0.660302952	0.543066208	1.521716916
0.485384519	0.467325608	0.709758438	0.339974866	1.475291457
0.662916929	0.504176227	0.492160234	0.417659744	1.058264312
0.691643254	0.528912142	0.679714121	0.362310871	1.268655181
0.819320604	0.34075341	0.445068715	0.35370046	1.04131027
0.529058808	0.286657514	0.458883552	0.313079294	0.876423455
0.828228068	0.428509769	0.568881472	0.463165596	0.855891604
0.652658404	0.576343173	0.974612074	0.372755101	1.526788262
1.853818497	0.268985442	0.538605176	0.772978397	1.20196926
0.502501567	0.327734624	0.631082271	0.338071429	0.821538448
0.599777788	0.222349336	0.30727451	0.470478271	0.987053371
0.927873476	0.270031572	0.347214716	0.304750396	0.737236804
0.744580635	0.356531141	0.696985235	0.468753056	1.641938397
0.546199538	0.328963631	0.576942721	0.39680442	1.503161478
0.911427623	0.253788746	0.559457328	0.409404822	0.924086623
0.742004582	0.481931498	0.611193032	0.340706175	1.103127846
0.572838356	0.380297405	0.610092542	0.432148782	1.0181852
0.78067826	0.343099755	0.585767477	0.247654373	1.249802386
0.610642539	0.331803268	0.479100426	0.293025881	1.081025084
0.410484599	0.298085892	0.366681656	0.4499074	0.597164372
0.891990345	0.433799411	0.743291492	0.459551993	2.411615655
0.752310432	0.506558221	0.649814571	0.365235778	1.244270365
1.001664938	0.528545655	0.460093825	0.56276324	0.950461438
0.470576115	0.305003986	0.670263371	0.246250746	0.787307977
0.90463016	0.626592758	0.501944584	0.521594297	1.610713609
0.573911425	0.336528361	0.408327885	0.323513989	0.963328665
0.621316382	0.620197665	0.412138173	0.338212058	1.177967716
0.682736127	0.335619861	0.709955252	0.469143116	1.086885526
0.670867612	0.36298957	0.437877735	0.256939393	0.61668258
0.357347599	0.42065226	0.522752514	0.310077339	0.874663502
0.696888619	0.433739278	0.473619373	0.282221538	1.00486382

Q9BZW8,CD244,INFLAMMATION	P20783,NTF3,INFLAMMATION	O00273,DFFA,INFLAMMATION	P60568,IL2,INFLAMMATION	O60880,SH2D1A,INFLAMMATION
Q9BZW8	P20783	O00273	P60568	O60880
CD244	NTF3	DFFA	IL2	SH2D1A
INFLAMMATION	INFLAMMATION	INFLAMMATION	INFLAMMATION	INFLAMMATION
0.479831575	0.890939887	0.926074395	0.976099416	0.647790852
0.704855778	0.937181253	0.660989839	0.719018103	0.555169417
0.640735131	0.750955852	0.981867326	0.885951728	0.655878278
0.658702988	0.619682014	0.873875706	0.504560789	0.59357408
0.613570064	1.082899982	1.291636397	0.431460383	0.638651146
0.502640909	0.903815372	0.77362161	0.799627503	0.519033657
0.555939579	0.540587478	0.622566563	0.732804364	0.581076494
0.512171897	0.659982648	0.423813074	0.512846862	0.535366956
0.528618932	0.499618914	0.566716849	0.957536907	0.574229757
0.424960307	0.444544577	0.726986259	1.032541666	0.644566002
0.42281545	0.579226695	0.575145947	0.912628745	0.591602478
0.641579522	1.093687064	1.347140197	0.672264093	0.870671256
0.425638336	0.676095992	0.61552954	0.415033588	0.659982648
0.341013321	0.417370344	0.600776381	0.46532158	0.377696678
0.377330337	0.554900112	0.394172764	0.473127197	1.201552761
0.460732093	0.489269074	0.796143281	0.491682871	0.766947644
0.427145651	0.736725968	1.056505297	0.585970524	0.606214388
0.492945481	0.763605818	0.895583591	0.507612675	0.620799801
0.602778557	0.732296599	0.918276162	0.741696055	0.537113914
0.597785579	0.723367156	0.579829243	0.647566384	0.626245397
0.357199013	0.57164841	0.649049313	1.056578531	0.491171923
0.435395982	0.78583591	0.994745933	1.018679346	0.63745703
0.460125718	0.462267551	0.785563607	0.530160099	0.552214241
0.344625175	0.599196042	0.595676108	0.74675143	0.493526686
0.451813724	0.626549327	0.659708226	0.714398026	0.744116286
0.514877096	0.771533123	0.630994791	0.631826345	0.70383053
0.679337312	0.927937794	0.546957257	0.542727531	0.619724968
0.384671806	0.670914114	1.037060457	0.929547182	0.620154678
0.479166848	0.527886618	0.630776142	0.593697523	0.562217396
0.336388432	0.561633151	0.472996037	1.08417677	0.465934802
0.459169907	0.568211525	0.476748399	0.628376007	0.537039459
0.424401012	0.667898139	0.619467286	0.508070287	0.467098915
0.329146098	0.529609167	0.565226104	0.768597399	0.501666324
0.498995946	0.649814571	0.482733884	0.878491359	0.556093739
0.467034166	0.598614861	1.054968557	1.089374498	0.858863044

P57771,RGS8,INFLAMMATION	P50995,ANXA11,INFLAMMATION	Q5KU26,COLEC12,INFLAMMATION	P01374,LTA,INFLAMMATION	P09603,CSF1,INFLAMMATION
P57771	P50995	Q5KU26	P01374	P09603
RGS8	ANXA11	COLEC12	LTA	CSF1
INFLAMMATION	INFLAMMATION	INFLAMMATION	INFLAMMATION	INFLAMMATION
0.829434522	0.777114992	0.59737137	0.94461608	0.367852678
0.738413073	0.74706206	0.725878505	0.828974713	0.420914758
0.877213549	0.862442393	0.677785181	1.345180714	0.436907568
1.176009733	1.292532001	0.431460383	0.63327323	0.426199262
1.085605549	0.813435579	0.555323364	1.214952575	0.374360468
0.774694821	0.984525173	0.711482413	0.863938187	0.366961344
0.790096075	1.037419937	0.413540348	0.605710363	0.379638971
0.767373048	0.692218786	0.619596114	0.767373048	0.338212058
1.045577499	0.909597378	0.46364741	0.792344646	0.304729273
0.740052743	0.896080346	0.355494711	0.650039819	0.309862484
0.706959758	0.914782074	0.526644013	0.896080346	0.316197968
0.816768991	0.868260586	1.038787098	0.991647993	0.633888061
1.076986376	1.063190412	0.56178889	0.839033314	0.289312387
0.660806599	0.779704843	0.516951199	0.379902207	0.275170935
0.713853533	0.640468712	0.326578111	0.672170904	0.219866544
0.626592758	1.032541666	0.391341496	0.952902162	0.257206677
0.760542092	0.961660789	0.514377699	0.535441179	0.407310243
0.971105539	0.797911144	0.511107972	1.194825555	0.341723176
0.791576123	0.606802948	0.509339673	0.726130119	0.352525616
0.670263371	0.685106415	0.593162789	0.858030002	0.338305843
0.761491585	0.79901805	0.669010148	0.566481207	0.349048641
0.769236967	0.638872524	0.662733155	0.773782497	0.324412204
0.742004582	0.781707075	0.642825917	0.705246741	0.314405857
1.154285418	0.73163703	0.376964351	0.671612041	0.277507756
0.956608158	0.870429888	0.58931055	0.884847047	0.326035282
0.850450988	0.804352616	0.503094039	0.985002984	0.293798717
0.975490684	0.919805035	0.451876363	0.940043876	0.350916576
1.010101311	0.718619505	0.521775099	0.562607231	0.27308084
0.918721821	0.68136512	0.642870475	0.439489316	0.338282394
0.881052565	0.680232575	0.496546248	0.642959602	0.291223766
0.932063402	0.789986552	0.311823162	0.704318557	0.301807382
0.760963943	0.731282123	0.590168981	0.813210078	0.364199286
0.678255149	0.781165424	0.460955696	0.558256481	0.294655275
1.043260904	0.816429377	0.410370804	0.807984768	0.267980513
0.784420965	0.939783276	0.507577492	0.769450274	0.330059951

O14773,TPP1,INFLAMMATION	P18564,ITGB6,INFLAMMATION	P27540,ARNT,INFLAMMATION	O00300,TNFRSF11B,INFLAMMATION	Q9HB29,IL1RL2,INFLAMMATION
O14773	P18564	P27540	O00300	Q9HB29
TPP1	ITGB6	ARNT	TNFRSF11B	IL1RL2
INFLAMMATION	INFLAMMATION	INFLAMMATION	INFLAMMATION	INFLAMMATION
0.456282744	0.592217899	0.395678341	0.271025406	0.457295935
0.353479879	0.621230256	0.431580025	0.326895178	0.366757913
0.390041625	0.576303226	0.285151396	0.312515576	0.541449991
0.386542781	0.484880117	2.458707304	0.333093706	0.517776
0.399952341	0.51788368	0.866156725	0.376024873	0.789275023
0.326442319	0.716431164	0.607897504	0.271966338	0.319037961
0.316812244	0.528033	0.414401175	0.319480548	0.312840674
0.30919738	0.294145119	0.321101209	0.213706101	0.283751515
0.316000775	0.415235012	0.332678376	0.246216611	0.41884838
0.296129475	0.394801671	0.447016503	0.166281549	0.290316807
0.334087984	0.383979182	0.354436725	0.24227207	0.251459855
0.60336378	0.606130355	0.324254837	0.377618146	0.428272219
0.283279872	0.358787119	0.511214265	0.190135523	0.372083933
0.2582965	0.462011286	0.336971854	0.159530038	0.297652311
3.453118874	0.252612862	0.288331427	0.206440795	0.182998027
0.27302406	0.306997753	0.225938182	0.208714104	0.297652311
0.458374915	0.776253622	0.931998798	0.389123496	0.371774571
0.350867932	0.527082245	1.075196241	0.263669024	0.598822361
0.4765832	0.363719959	0.393081399	0.261412494	0.367750702
0.314754738	0.544423029	0.416792147	0.209860129	0.411824053
0.306360032	0.494074328	0.35379854	0.34484023	0.330151476
0.468136124	0.452001667	0.33503878	0.251459855	0.329488496
0.33457464	0.512527031	0.45081268	0.238820734	0.511249701
0.267201498	0.485485462	0.298872073	0.158406122	0.265410987
4.003883508	0.331412518	0.316790285	0.247740218	0.501353466
0.313057593	0.275743733	0.316307573	0.343147322	0.396639428
0.334690614	0.410826173	0.431879278	0.286876163	0.439976996
0.366656241	0.556787991	0.428569177	0.216029464	0.310012867
0.438637177	0.382014684	0.32585454	0.262101951	0.289974914
0.269227932	0.759435845	0.380112928	0.284874817	0.452597337
0.282006437	0.435245112	0.376990481	0.152957208	0.320101202
0.393108646	0.406407801	0.34642141	0.355642588	0.274713554
0.266960833	0.590291716	0.445593471	0.328690121	0.326035282
0.246421493	0.520330435	0.265024934	0.186287141	0.459902517
0.394036178	0.365894593	0.394118124	0.229123922	0.488625141

P26022,PTX3,INFLAMMATION	Q9UJA9,ENPP5,INFLAMMATION	P15291,B4GALT1,INFLAMMATION	P33241,LSP1,INFLAMMATION	P13747,HLA-E,INFLAMMATION
P26022	Q9UJA9	P15291	P33241	P13747
PTX3	ENPP5	B4GALT1	LSP1	HLA-E
INFLAMMATION	INFLAMMATION	INFLAMMATION	INFLAMMATION	INFLAMMATION
1.531770331	0.592505315	0.319524841	0.554092982	0.745510202
0.930127244	0.254987777	0.467098915	0.586498775	0.78154454
1.36717697	0.626115187	0.384325338	0.574866952	0.979691888
1.209490971	0.699792933	0.410569966	0.567070495	0.842471624
1.558653238	0.485687412	0.35150083	1.059071506	1.055626886
2.166400535	0.46852567	0.364982703	0.733414147	0.912692006
1.45730212	0.677315539	0.315694274	0.520294369	0.746596163
1.708819482	0.476087944	0.386060806	0.370693828	0.811577065
1.73616011	0.353970246	0.240715342	0.441076255	0.728499557
1.215710738	0.484376239	0.299452691	0.651889797	0.777707739
1.0604672	0.463936738	0.246370257	0.687199075	0.810846089
2.44206229	0.49427985	0.511994423	0.787253406	0.903376945
2.275210456	0.463551007	0.260706779	0.596626515	0.728398572
0.891125172	0.411082539	0.239865903	0.361608376	0.637280314
1.277745048	0.317053894	0.138715413	0.395952699	0.734584317
1.825890186	0.377618146	0.195819996	0.643851552	0.687770909
1.42899414	0.946582398	0.297054593	0.495755265	0.713309454
1.067103417	0.553709047	0.308319918	0.695971435	0.828170661
1.055261097	0.549693757	0.293168093	0.603907711	0.718071794
1.641369443	0.464676954	0.227793739	0.471817227	0.733770088
1.415488472	0.38969032	0.285230468	0.44319085	0.721464343
2.041316662	0.319945926	0.281713382	0.599902521	0.824162085
1.635917506	0.425726854	0.26312131	0.519537575	0.775339462
1.378978535	0.49134218	0.206269154	0.326419692	0.715042054
0.733922687	0.570737792	0.29711637	0.427530722	0.807145126
1.980960344	0.774265358	0.25278802	0.463679548	0.77781556
1.21369002	0.279592972	0.380244688	0.746440929	0.85027416
0.4695335	0.440617897	0.304286029	0.611913656	0.572679553
2.318352594	0.507050027	0.412652704	0.47853621	0.786435308
0.821083017	0.691307748	0.262738587	0.490899635	1.124200997
0.706274052	0.510576839	0.260075067	0.344720739	0.757963352
1.956940646	0.469468414	0.328052814	0.370334279	0.874118029
1.28155909	0.419255031	0.258099634	0.311218559	0.644208678
1.059291757	0.433228482	0.256690178	0.47237352	0.828113259
1.587437729	0.394309398	0.306721244	0.684015063	0.666141238

P32970,CD70,INFLAMMATION	Q9H0P0,NT5C3A,INFLAMMATION	P50591,TNFSF10,INFLAMMATION	P54317,PNLIPRP2,INFLAMMATION	Q9UIB8,CD84,INFLAMMATION
P32970	Q9H0P0	P50591	P54317	Q9UIB8
CD70	NT5C3A	TNFSF10	PNLIPRP2	CD84
INFLAMMATION	INFLAMMATION	INFLAMMATION	INFLAMMATION	INFLAMMATION
0.166546853	0.819093471	0.42637655	0.69457385	0.358662794
0.302142283	0.576103529	0.557521752	0.003409793	0.469273208
0.423402003	0.996540263	0.534958914	0.284184544	0.408497739
0.494040082	0.540999813	0.490117648	0.002634418	0.304117344
0.218393224	0.741233505	0.539240216	0.862263072	0.347792807
0.5562865	1.229694548	0.436998429	0.40000779	0.302960161
0.222349336	0.752727717	0.609120684	0.663974619	0.300138438
0.442853062	0.556093739	0.456377635	0.292761957	0.376077005
0.229680455	0.894404902	0.470347845	0.185552584	0.225140889
0.401786223	0.702368476	0.372367741	0.566010217	0.276509317
0.164356452	0.943896121	0.328439603	0.490049708	0.228711371
0.525440751	1.483083679	0.68179031	0.003479269	0.582891795
0.292843139	0.783931771	0.523768064	0.164402028	0.518099106
0.509410287	0.510718421	0.391205891	0.220859378	0.211202673
0.189556522	0.747735534	0.360532195	0.001463562	0.307423637
0.270125174	0.745200217	0.4575813	0.273383864	0.221825942
0.298313258	1.111647973	0.415465331	0.353504381	0.355002232
0.439245679	0.755131602	0.389852421	0.270818838	0.310421416
0.382226577	0.390149782	0.496202187	0.156996032	0.264914736
0.399647508	0.542953292	0.540175458	0.126586898	0.387079015
0.247431314	0.652658404	0.448661719	0.182453407	0.250485674
0.490321524	0.914274952	0.493595108	0.00506578	0.254793433
0.303969821	0.616896343	0.376207366	0.002922057	0.278857504
0.257028457	0.68060988	0.419196914	0.240581898	0.191564216
0.538381223	1.13084406	0.447357466	0.001747248	0.358662794
0.36155825	0.935169641	0.458565588	0.208526118	0.302729254
0.237533023	0.912881814	0.495102795	0.002199513	0.338798643
0.289834251	1.24323584	0.391639993	0.002888428	0.284657694
0.501979377	1.255620075	0.697855382	0.153925056	0.36698678
0.364653968	1.678182947	0.374308574	0.388019214	0.243467313
0.345366487	0.973059547	0.432238654	0.135456356	0.232257969
0.562568235	0.978470323	0.50024266	0.473947775	0.322015045
0.293391708	0.621273318	0.399481334	0.363770385	0.230557739
0.280719269	0.620928906	0.371877663	0.235418565	0.350916576
0.254793433	1.264616545	0.496993883	0.705100105	0.300825756

P00813,ADA,INFLAMMATION	P24387,CRHBP,INFLAMMATION	Q14005,IL16,INFLAMMATION	P10145,CXCL8,INFLAMMATION	Q8TEU8,WFIKKN2,INFLAMMATION
P00813	P24387	Q14005	P10145	Q8TEU8
ADA	CRHBP	IL16	CXCL8	WFIKKN2
INFLAMMATION	INFLAMMATION	INFLAMMATION	INFLAMMATION	INFLAMMATION
0.974949907	0.330380399	0.283869548	0.261249467	0.619896816
1.389243991	0.415811049	0.414976056	0.254793433	0.582851394
1.189207115	0.38844978	0.312688919	0.354215684	0.66494181
0.926331193	0.322774833	0.345222883	0.323828081	0.436514051
1.110107969	0.394446079	0.553670668	0.550189305	0.657927263
1.15885495	0.314863843	0.306636215	0.490423494	0.568093381
0.916559218	0.381221132	0.159496868	0.312840674	0.492603915
0.966539099	0.263322006	0.205114303	0.32894083	0.566520474
0.834624977	0.29508449	0.190465288	0.219988498	0.477707688
0.797855838	0.260201286	0.196977124	0.357000995	0.377670499
0.791905398	0.364098323	0.217245771	0.254228909	0.441841244
1.449645356	0.5121009	0.398927919	0.622135183	0.669195663
1.079527504	0.341770552	0.182833203	0.251948365	0.416445613
1.043839571	0.260526133	0.148537592	0.351744556	0.39433673
0.717226153	0.184577679	0.1357101	0.331849269	0.286339777
0.82410496	0.253911915	0.230302184	0.189740558	0.345725758
0.860770178	0.373582816	0.280116719	0.487103414	0.724973416
1.08658422	0.376520416	0.2874534	0.327167195	0.708922586
0.907456253	0.351622672	0.391802905	0.262592934	0.470184863
1.187395047	0.367878177	0.275113721	0.308683441	0.324547152
0.946254394	0.280855508	0.191923064	0.319436262	0.422200444
0.857376037	0.297569796	0.217065146	0.637236142	0.243433564
0.779704843	0.232693046	0.291405498	0.386489199	0.375295787
0.805412629	0.268259283	0.143467911	0.282299798	0.353970246
1.138788635	0.355100673	0.226879789	0.37845666	0.622609718
1.121088367	0.373246335	0.178649032	0.377696678	0.617067407
0.817731998	0.396941965	0.16670855	0.456124636	0.541449991
0.95627668	0.362738052	0.220813457	0.602444398	0.360807192
0.929031874	0.425225494	0.173450992	0.462299594	0.446335357
0.655151289	0.371156618	0.170672201	1.253619912	0.412452532
0.853817714	0.279864422	0.25493476	0.271627226	0.456694082
1.182385105	0.418674222	0.12267388	0.217170493	0.506207224
0.832429501	0.350357576	0.182326984	0.328189275	0.343670995
0.673943709	0.242137763	0.119468319	0.547488285	0.404636958
0.786162798	0.372936007	0.279689888	0.378430428	0.566599015

Q9UKX5,ITGA11,INFLAMMATION	P51617,IRAK1,INFLAMMATION	P30048,PRDX3,INFLAMMATION	Q8TD46,CD200R1,INFLAMMATION	Q8N608,DPP10,INFLAMMATION
Q9UKX5	P51617	P30048	Q8TD46	Q8N608
ITGA11	IRAK1	PRDX3	CD200R1	DPP10
INFLAMMATION	INFLAMMATION	INFLAMMATION	INFLAMMATION	INFLAMMATION
2.186767744	0.644431982	0.471522984	0.548209791	0.691547378
1.29154687	0.599362198	0.697130184	0.551678629	0.731484906
1.599365863	0.649949711	1.013608129	0.715984371	0.604661656
1.466828294	0.700472346	2.683656458	0.37428263	0.977453516
1.442928687	0.782411782	0.453728127	0.456061408	0.834451439
2.777717728	0.631476084	0.692026889	0.562178428	0.578745108
0.782357551	0.834740688	0.599154511	0.483236053	0.777330484
0.971509494	0.476186954	0.491240019	0.413511684	0.579106261
1.245392072	0.814846378	0.741182128	0.495995866	0.485754747
0.997300377	0.995504678	0.916305129	0.266923827	0.671518942
1.274295617	0.918785504	0.737287907	0.439824538	0.624468195
1.342479446	0.819207029	0.537188379	0.610938896	0.573076642
2.315301381	0.62876813	0.897821158	0.444513765	0.773031978
0.804854553	0.79366385	0.430176315	0.290578527	0.436786448
1.016915638	0.576982713	0.54231388	0.396941965	0.540362701
0.612125765	0.531742616	0.542539469	0.451782408	1.029254674
1.465100875	0.675112577	0.555361857	0.535515412	0.818752891
2.097251677	1.061717539	0.724069457	0.503477775	0.963929808
1.276594202	0.640646313	0.870007655	0.557058212	0.531779475
1.111725029	0.656560563	0.611277767	0.764718139	0.629422213
1.50305729	0.538045468	0.299805759	0.313448428	0.643182474
0.958665886	0.968215436	0.507085174	0.513131323	0.640291161
1.128964405	0.631826345	0.827941075	0.398817328	0.52007803
0.90463016	0.658109704	0.534699414	0.32261826	0.425638336
1.595932905	0.614336071	0.680798612	0.347816915	0.524749212
1.204137381	0.58922886	0.992473165	0.528802169	0.608614242
0.753615216	0.77598464	0.829894586	0.472733825	0.690828736
0.844225317	0.995573683	0.96908828	0.362662631	0.430027253
1.120311557	0.80865711	0.903189112	0.435094293	0.542614686
1.080950156	0.910354274	0.504350992	0.416387885	0.689202576
0.707548035	0.552635444	0.773299937	0.395239763	0.577983415
0.946319986	0.784149153	0.509586866	0.47237352	0.590619134
1.180747106	0.551563923	0.490389502	0.31822079	0.768011596
1.028256363	0.509904862	0.647297124	0.407564415	0.556595056
0.965467767	0.848037519	0.516127712	0.4659671	0.658657332

O95633,FSTL3,INFLAMMATION	Q14116,IL18,INFLAMMATION	Q6UB28,METAP1D,INFLAMMATION	Q08334,IL10RB,INFLAMMATION	P24001,IL32,INFLAMMATION
O95633	Q14116	Q6UB28	Q08334	P24001
FSTL3	IL18	METAP1D	IL10RB	IL32
INFLAMMATION	INFLAMMATION	INFLAMMATION	INFLAMMATION	INFLAMMATION
0.256156961	0.276470988	0.442055679	0.36394693	0.557096825
0.319635599	0.218257026	0.364401297	0.526023807	0.483705216
0.286995496	0.338939574	0.58666141	0.505646133	0.552482242
0.259588792	0.229489492	0.683209528	0.365058607	0.428955531
0.251355297	0.296663636	0.554515618	0.35026045	0.616127143
0.3747499	0.261611887	0.68037404	0.458883552	0.722465199
0.317031918	0.324412204	0.393408491	0.439641658	0.352623371
0.3139703	0.242322454	0.540550009	0.523876989	0.495858365
0.261158941	0.256494536	0.412338192	0.310636659	0.479133635
0.21698993	0.192429248	0.431131536	0.289011739	0.299639557
0.280563648	0.210880851	0.543631139	0.378194425	0.432418455
0.417717647	0.3028132	0.517811891	0.725224717	0.851571752
0.203372991	0.160139377	0.475857	0.363719959	0.570421395
0.226565485	0.146097156	0.474144925	0.420623103	0.39172144
0.146097156	0.214700879	0.46461254	0.263322006	0.335247853
0.195955776	0.397602849	0.652613167	0.293432383	0.432418455
0.302016652	0.244583672	0.529572459	0.377173442	0.63630925
0.228743079	0.207963176	0.46888304	0.444082615	0.563543937
0.240398533	0.244973908	0.562724234	0.341794242	0.481063751
0.219060307	0.160606257	0.380086582	0.467876606	0.453256621
0.276566822	0.261158941	0.366046796	0.410883129	0.339598031
0.261738853	0.449221848	0.612295506	0.341202471	0.400923809
0.210866234	0.373298082	0.429193461	0.413253803	0.379191886
0.192976891	0.141649754	0.48454414	0.332470905	0.377225733
0.225000482	0.165671805	0.415407739	0.503931659	0.881846831
0.239102314	0.140388824	0.475692109	0.382597672	0.539651523
0.303254298	0.219836066	0.535255641	0.434642152	0.316197968
0.186493855	0.223415317	0.439550247	0.390041625	0.536407011
0.289152003	0.227052842	0.458883552	0.420244254	0.411510173
0.270069009	0.235549145	0.461115479	0.445192132	0.431580025
0.254758114	0.261847729	0.403964382	0.352427889	0.332563099
0.252000762	0.300471487	0.459201736	0.513736328	0.545896745
0.231310073	0.230015024	0.607855369	0.329922712	0.434822952
0.241182979	0.174475939	0.632834431	0.449003937	0.41647448
0.229473585	0.419953064	0.665356752	0.390285021	0.405394944

P01135,TGFA,INFLAMMATION	Q9HCM2,PLXNA4,INFLAMMATION	Q99616,CCL13,INFLAMMATION	Q12765,SCRN1,INFLAMMATION	P03956,MMP1,INFLAMMATION
P01135	Q9HCM2	Q99616	Q12765	P03956
TGFA	PLXNA4	CCL13	SCRN1	MMP1
INFLAMMATION	INFLAMMATION	INFLAMMATION	INFLAMMATION	INFLAMMATION
0.353381887	0.380824975	0.051200439	0.766256865	0.346373389
0.386944887	0.499030535	0.061481663	0.738464258	0.201912201
0.495823996	0.430534275	0.101827561	0.844752137	0.184949076
0.349629785	0.372961858	0.066288389	0.532258873	0.070501609
0.483604643	0.373764123	0.089293564	0.651483254	0.16935236
0.496374188	0.498961359	0.055237813	0.598117154	0.09431297
0.34719065	0.395102807	0.061588295	0.501075533	0.138484844
0.352647814	0.333139886	0.040644389	0.497131698	0.161824281
0.299431935	0.251686545	0.091106639	0.745251872	0.430086872
0.268966798	0.365185149	0.11255484	0.814112457	0.204234713
0.309798057	0.294696126	0.058981241	0.492262587	0.226094844
0.527374602	0.489506526	0.124394965	0.916876928	0.366961344
0.385312261	0.46143521	0.087686588	0.50986952	0.112687547
0.294451106	0.261158941	0.117529922	0.488354264	0.07065816
0.251826149	0.306423745	0.028799748	0.553478814	0.297136965
0.255465433	0.524240239	0.081176165	0.550952558	0.490321524
0.430474594	0.33662168	0.19241591	0.698678185	1.65175533
0.393544859	0.409944355	0.080721679	1.014100054	0.052164061
0.369872514	0.382809888	0.115911852	0.540100579	0.149674463
0.303485606	0.237434256	0.075415245	1.168291269	0.151953309
0.2946757	0.377775226	0.090176792	0.515162684	0.129282598
0.283260237	0.329237369	0.135071949	0.677691227	0.298354615
0.324097546	0.180341084	0.098679896	0.713210575	0.175373176
0.235125025	0.40114619	0.071669346	0.562724234	0.20919206
0.387320563	0.388234438	0.092237678	0.790150842	0.126411533
0.360557186	0.30333839	0.101608993	0.884356519	0.197305079
0.425196021	0.349896466	0.096495134	0.648914361	0.100928114
0.243315478	0.370026372	0.089846119	1.021224461	0.190412487
0.347527729	0.484913727	0.073459862	0.862741345	0.159474759
0.337135394	0.39280903	0.108172074	0.737390124	0.312645574
0.23379241	0.282946265	0.110621215	0.421119037	0.394774306
0.373790032	0.367572311	0.071386743	0.508140725	0.402399384
0.249446097	0.260815226	0.134101753	0.652658404	0.533034198
0.371877663	0.348154602	0.071287849	0.724370651	0.156528797
0.336178648	0.260345613	0.08589393	3.261193399	0.095015059

P14317,HCLS1,INFLAMMATION	P20340,RAB6A,INFLAMMATION	Q9BYZ8,REG4,INFLAMMATION	Q9Y258,CCL26,INFLAMMATION	P22466,GAL,INFLAMMATION
P14317	P20340	Q9BYZ8	Q9Y258	P22466
HCLS1	RAB6A	REG4	CCL26	GAL
INFLAMMATION	INFLAMMATION	INFLAMMATION	INFLAMMATION	INFLAMMATION
0.33118288	0.437726005	0.1865585	1.372303895	0.229060404
0.248927927	0.513487122	0.159618525	1.667630889	0.173571261
0.26811057	0.48801588	0.281811033	1.415390361	0.364148801
0.44260756	0.449876215	0.156496251	1.570256237	0.199049617
0.591561472	0.384085658	0.441351498	1.80325072	0.133656323
0.288191562	0.367241244	0.216839577	2.694653926	0.458438464
0.192096082	0.264474402	0.257438548	0.573871646	0.325922306
0.209221062	0.411111034	0.2383742	0.716878237	0.291001804
0.303128205	0.250885327	0.196118835	1.057897609	0.09634142
0.326148297	0.521811267	0.141797107	0.704220925	0.1971957
0.246438574	0.398734405	0.149487836	0.376572617	0.070982146
0.442668923	0.520402573	0.287035285	1.060834794	0.33631849
0.162037541	0.492535631	0.083591497	0.770571108	0.301849225
0.164026407	0.325696473	0.112033337	0.476087944	0.131615381
0.16292727	0.388315177	0.12309124	0.527740277	0.196867927
0.42443043	0.285131631	0.271232131	0.353161505	0.206555302
0.331688293	0.481898094	0.180992272	0.822108092	0.142378183
0.371697271	0.529022138	0.140165189	1.623154129	0.22084407
0.351135557	1.344434994	0.224237582	0.99854545	0.170011
0.248015123	0.345390426	0.166270024	1.792284918	0.157770568
0.1770342	0.30945467	0.180979727	1.356040249	0.147001217
0.282671826	0.332010322	0.118832388	0.847919965	0.082755557
0.235353302	0.457676461	0.244041769	1.136580603	0.164675747
0.179219559	0.280252666	0.160661928	3.695834105	0.404889462
0.177193796	0.422581056	0.197469261	7.4014093	0.176189518
0.263468063	0.33010571	0.224579788	1.078330933	0.118232618
0.282006437	0.352696704	0.15793469	0.660669203	0.173078689
0.453696678	0.352208102	0.135212459	0.81056512	0.161813065
0.299972052	0.390420307	0.159629589	0.562061538	0.24760288
0.211627644	0.419982174	0.180729009	1.280582323	0.193512679
0.200851341	0.228632119	0.273497585	0.823077395	0.133073942
0.153052657	0.200739996	0.188234112	1.541142217	0.33376394
0.165637358	0.2744471	0.177464209	0.571331508	0.177292081
0.184949076	0.33464422	0.168848352	0.37298771	0.338165175
0.588412582	0.311779937	0.203697475	0.736828107	0.117098946

Q9Y2J8,PADI2,INFLAMMATION	Q9UMR7,CLEC4A,INFLAMMATION	Q6UXK5,LRRN1,INFLAMMATION	Q16363,LAMA4,INFLAMMATION	P35225,IL13,INFLAMMATION
Q9Y2J8	Q9UMR7	Q6UXK5	Q16363	P35225
PADI2	CLEC4A	LRRN1	LAMA4	IL13
INFLAMMATION	INFLAMMATION	INFLAMMATION	INFLAMMATION	INFLAMMATION
0.228965161	0.72865106	0.690924512	0.629422213	0.603280142
0.482767345	0.488794515	0.634855429	0.677315539	0.429133966
0.417515019	0.717226153	0.917512679	0.802514854	0.481430684
0.776630354	0.780299565	0.936661713	0.545556303	0.714645661
0.496202187	0.683541104	0.888288365	0.510753822	0.636353357
0.604619746	0.951384219	0.492057903	0.766947644	0.596006512
0.260417806	0.44664484	0.524022259	0.384805147	0.624987829
0.542013241	0.475000191	0.481197149	0.676846222	0.469175635
0.458184322	0.651257506	0.373168729	0.46461254	0.694670145
0.563387711	0.47371787	0.397382432	0.391531422	0.632922166
0.535107257	0.520294369	0.530343871	0.558101721	0.469403336
0.516235049	0.659936903	0.933097667	1.025551542	0.569078666
0.574428805	0.862561961	0.511391469	0.536332655	0.561166192
0.474703963	0.340163441	0.500416061	0.52471284	0.557908331
0.482800809	0.394282067	0.328030076	0.337299013	0.629291342
0.528399131	0.326012684	0.480597151	0.314820196	0.523151245
0.5121009	1.121632455	0.522354087	0.909912675	3.280464252
0.632352102	0.601943507	0.837929052	0.806529943	0.870067961
0.372780939	0.764082329	0.55194637	0.513950029	0.574468622
0.735807354	0.451000207	0.532664855	0.535329848	0.634591455
0.352501182	0.553747429	0.555323364	0.674457761	0.697130184
0.559961677	0.560272272	0.697516863	0.581036218	0.539576716
0.375946689	0.687723238	0.678678399	0.565853307	0.552022892
0.326736606	0.465450612	0.340635334	0.355445432	0.591971654
0.470021938	0.462043312	0.942457848	0.590128075	0.447450501
0.344672954	0.819207029	0.415724593	0.542652298	0.601651513
0.428598884	0.381802909	0.502327443	0.434612026	0.748565259
0.370899441	0.379375916	0.903878022	0.72693587	0.923318309
0.37659872	0.549579463	0.644521326	0.719167634	0.642959602
0.380956982	0.74406471	0.446180695	0.448910579	0.73163703
0.462684284	0.418413121	0.394200087	0.326555475	0.865976632
0.363090226	0.392101754	0.652884637	0.744993633	0.45565064
0.674457761	0.280174974	0.602068691	0.465870215	0.519501565
0.600568204	0.434762677	0.69457385	0.554361895	0.837116315
0.285804394	0.471915349	0.68164855	0.408356189	0.743497605

Q96PD4,IL17F,INFLAMMATION	P32456,GBP2,INFLAMMATION	Q96PL1,SCGB3A2,INFLAMMATION	P48023,FASLG,INFLAMMATION	Q9UQV4,LAMP3,INFLAMMATION
Q96PD4	P32456	Q96PL1	P48023	Q9UQV4
IL17F	GBP2	SCGB3A2	FASLG	LAMP3
INFLAMMATION	INFLAMMATION	INFLAMMATION	INFLAMMATION	INFLAMMATION
0.78192384	0.658839976	0.520041982	0.518530228	0.178537619
0.621014991	0.5562865	0.324367234	0.733058379	0.243382948
0.985139544	1.043477867	0.612550206	0.518853805	0.427738212
0.833699861	0.803349679	0.359608743	0.622911883	0.339315679
0.820684721	0.592710697	0.321479801	0.795095467	0.279515463
0.781165424	0.491137879	0.310550544	0.655923741	0.166696995
0.74913623	0.457137476	0.172324532	0.518566171	0.2233379
0.595098341	0.213854282	0.29461443	0.406971593	0.328530678
1.183451022	0.658246568	0.549617559	0.70514898	0.222287696
0.666279772	0.873330724	0.435969768	0.54231388	0.218302416
0.863279719	0.897634481	0.275915804	0.509657515	0.195806424
0.618480494	0.515805835	0.357149498	0.54536726	0.251791241
0.886750412	0.971576836	0.497235084	0.557212682	0.245637035
1.03289958	0.805468458	0.316548837	0.390203872	0.18772595
0.584267117	0.615913647	0.681081807	0.409262958	0.125668938
0.624424911	0.469175635	0.331665303	0.664757475	0.229219232
0.823476851	0.26170257	1.070882357	0.732195088	0.459711289
0.646624466	0.583538602	0.369411325	0.829779546	0.319502694
0.886074556	0.392564059	0.465611953	0.732195088	0.23940082
0.650896472	0.33489947	0.145510988	0.457295935	0.298416663
0.900313901	0.87436042	0.161141501	0.455019412	0.165327658
0.798575103	0.446923558	0.177562644	0.298520104	0.167821563
0.618866442	0.748409616	0.222580637	0.302624354	0.131834512
0.776361241	0.49944579	0.374049213	0.450500309	0.167833196
0.681223448	0.569749635	0.343028417	0.608023926	0.583983697
0.595015848	0.70120102	0.303569762	0.751007906	0.226942702
0.850568894	0.596791958	0.303002164	0.652794135	0.174391303
0.840255508	0.55186986	0.206240561	0.356012549	0.188521373
1.336629852	0.563543937	0.179904104	0.529388955	0.299992846
0.49909972	0.901562866	0.318706423	0.30367499	0.138408073
0.782249101	0.378876615	0.258995691	0.637236142	0.184373089
0.899191311	0.94487802	0.181117769	0.365767805	0.305215472
0.722515278	0.625421188	0.166154814	0.461851193	0.186493855
0.999514915	0.712420038	0.270218808	0.442546206	0.245926652
0.655015068	0.487170946	0.353039129	0.460317118	0.26105035

Q9NR12,PDLIM7,INFLAMMATION	P20273,CD22,INFLAMMATION	P51888,PRELP,INFLAMMATION	Q12968,NFATC3,INFLAMMATION	O76036,NCR1,INFLAMMATION
Q9NR12	P20273	P51888	Q12968	O76036
PDLIM7	CD22	PRELP	NFATC3	NCR1
INFLAMMATION	INFLAMMATION	INFLAMMATION	INFLAMMATION	INFLAMMATION
0.667990736	1.379456535	0.191179535	0.356555855	0.425933468
0.51419946	1.501807602	0.212068169	0.491887398	0.877578447
0.606887075	2.051103083	0.206985269	0.593245024	0.690397909
0.558604848	1.268479321	0.186907971	1.076837085	0.570816918
0.650445461	1.562872405	0.11811794	0.611828832	0.8362464
0.570263263	1.424741317	0.208107375	0.514092546	0.674364268
0.554515618	1.513302078	0.197894031	0.551563923	0.677879149
0.656151107	1.200636972	0.181419319	0.38966331	0.708431369
0.694140687	0.988491181	0.177895263	0.617538077	0.46076403
0.466839972	1.261377409	0.153435051	0.486597226	0.386944887
0.595222101	0.876970367	0.158504972	0.53972634	0.312884045
0.776953413	1.276948198	0.235255442	0.372058143	0.695055458
0.5562865	1.203386436	0.137957902	0.650310219	0.514627337
0.483738745	1.135793059	0.142151379	0.298354615	0.391531422
0.532332665	0.8005703	0.106675442	0.433528877	0.263742139
0.603823998	0.786544339	0.187258096	0.483068605	0.443651884
0.66429686	1.219170585	0.286677384	0.520727318	0.576622885
0.579146403	1.836297742	0.21067631	0.580794622	0.52123288
0.554707832	1.287703104	0.184577679	0.345605959	0.467293217
0.50024266	1.519924856	0.148157134	0.534365955	0.49373198
0.608276848	0.908967111	0.144265676	0.419662076	0.383766318
0.631388549	0.845748137	0.162735398	0.540063143	0.369360117
0.604997045	1.102516311	0.149001628	0.592094764	0.294634852
0.523151245	1.003610869	0.135644269	0.413225159	0.399010883
0.612550206	1.340991414	0.144305681	0.402259947	0.544800525
0.624641358	0.962928111	0.206069086	0.412767132	0.488760635
0.525513598	1.053215019	0.227983291	0.622307699	0.849449449
0.579789053	1.088846059	0.1376427	0.581963267	0.421557111
0.575704343	1.074376758	0.192522638	0.422112659	0.583215109
0.61796627	0.715785885	0.201157858	0.596047826	0.293025881
0.59691607	0.601943507	0.209583929	0.375529982	0.438819639
0.543894974	1.455585919	0.158900988	0.417949344	0.475560238
0.575106082	0.698290863	0.175021007	0.473028823	0.426849679
0.524349263	0.987190215	0.148054475	0.495034164	0.531742616
0.602862125	1.275974944	0.176360576	0.752675543	0.426731348

Q9BXJ7,AMN,INFLAMMATION	P16422,EPCAM,INFLAMMATION	Q9Y478,PRKAB1,INFLAMMATION	P24071,FCAR,INFLAMMATION	Q92956,TNFRSF14,INFLAMMATION
Q9BXJ7	P16422	Q9Y478	P24071	Q92956
AMN	EPCAM	PRKAB1	FCAR	TNFRSF14
INFLAMMATION	INFLAMMATION	INFLAMMATION	INFLAMMATION	INFLAMMATION
0.507260947	0.269209271	0.81197094	0.475692109	0.369898153
0.783117124	0.170199653	0.460987648	0.975625925	0.517704226
0.344529638	0.251494717	0.531484676	0.971980988	0.468980551
0.4499074	0.189885283	0.73769686	0.704611536	0.363644333
0.86106855	0.195453862	0.635383707	0.836072525	0.433378654
0.531411001	0.814676953	0.69399636	0.692218786	0.394993276
0.430713366	0.190848533	0.470021938	0.879710051	0.410826173
0.413540348	0.185051662	0.904442067	0.55413139	0.484812903
0.694285045	0.248565849	1.063780134	0.817108746	0.313296379
0.49120597	0.180528685	0.899814799	0.609205132	0.291668199
0.390149782	0.231181843	0.718071794	0.467909038	0.28406638
0.463679548	0.182921936	0.585199321	0.608234687	0.682594171
0.625117805	0.128158405	0.785563607	0.475131908	0.333787076
0.467001795	0.292964955	0.779650799	0.494588294	0.311974496
0.582487906	0.161521711	0.823990723	0.634107788	0.212185798
0.3515983	0.132944869	0.713507253	0.510187692	0.281401125
0.517488964	0.305490622	0.688343219	0.683209528	0.465128098
0.587312399	0.418761292	0.834567127	0.700423794	0.332194479
0.589637424	0.120391046	0.572957487	0.489099536	0.262501942
0.332516999	0.292985262	0.775554462	0.467228441	0.366148299
0.402566772	0.247225592	0.650580732	0.668222284	0.387562261
0.490321524	0.948552809	0.707253835	0.572798651	0.354485863
0.386944887	0.228743079	0.574986504	0.498995946	0.350965226
0.790041312	0.204206402	0.759330572	0.584712768	0.266387815
0.300263288	0.289232184	0.612847489	0.765195344	0.394254739
0.40410441	0.197359791	0.351525195	0.607139524	0.341865324
0.476946715	0.173595325	0.716679503	0.856188285	0.462844666
0.59106963	0.554285049	1.147584192	0.933162346	0.33178027
0.537859028	0.369693094	0.775446954	0.77840884	0.443836432
0.717425037	0.311261706	0.912818541	0.495034164	0.36389648
0.371671508	0.383447243	1.005909142	0.396062496	0.331297679
0.457898581	0.326306583	0.805635968	0.689680461	0.415609345
0.544876056	0.152576005	0.584753299	0.35045473	0.348855141
0.652658404	0.633317127	0.811858385	0.640601908	0.351939659
0.398844973	0.211393072	0.626332219	0.409773899	0.385606159

O00175,CCL24,INFLAMMATION	P01375,TNF,INFLAMMATION	Q9NP70,AMBN,INFLAMMATION	P19256,CD58,INFLAMMATION	Q14210,LY6D,INFLAMMATION
O00175	P01375	Q9NP70	P19256	Q14210
CCL24	TNF	AMBN	CD58	LY6D
INFLAMMATION	INFLAMMATION	INFLAMMATION	INFLAMMATION	INFLAMMATION
0.035080181	0.554438751	3.231715057	0.472668294	0.333440211
0.060258142	0.55856613	1.28422679	0.542727531	0.270125174
0.152904206	0.735348476	1.304774055	0.460189509	0.386274943
0.050373193	0.711778371	2.092604979	0.387965427	0.280874976
0.14014576	0.996747509	2.068808345	0.465902507	0.320700832
0.068369657	0.742724977	1.299539062	0.45429458	0.312190816
0.35013908	0.618737766	0.93789609	0.395815496	0.438424401
0.147839122	0.642024385	1.33403822	0.370488329	0.366351391
0.128282831	0.554823192	1.572216614	0.322685353	0.320900958
0.051879198	0.826966046	1.570800541	0.332263564	0.791301831
0.300450661	0.47976506	1.22858698	0.368669507	0.313404978
0.057899713	0.607223698	1.385301498	0.680562706	0.648644541
0.078177648	0.335643126	1.2796063	0.392754579	0.293961679
0.189819485	0.461467196	2.247778692	0.329123284	0.343670995
0.050797454	0.445778827	1.449143035	0.262483747	0.19489919
0.227999095	0.481931498	1.07758375	0.267442379	0.291769301
0.076749438	0.75917269	2.853831192	0.515198394	0.34108424
0.073597471	0.748565259	4.609147701	0.383075323	0.291082498
0.052991325	0.568054006	2.184798154	0.412709914	0.370154635
0.263723859	0.611828832	1.322437921	0.403656492	0.311823162
0.109317782	0.574667753	1.062159186	0.304286029	0.292093063
0.067428392	0.880625179	1.299989526	0.28628024	5.998492591
0.088750558	0.659845423	1.85214879	0.367699724	0.447078477
0.064677154	0.524022259	2.490438871	0.305046271	0.295207238
0.137490134	0.849331699	1.436243138	0.334597831	0.234165428
0.401925495	0.540212901	1.045794944	0.376990481	0.289533061
0.061643817	0.507718242	1.087036211	0.532185091	0.300096833
0.040638755	0.692218786	1.931337103	0.302205118	0.242104198
0.297693577	0.752623374	1.294414792	0.466193243	0.373427479
0.068883392	0.543367431	1.595601074	0.355100673	0.393135895
0.177267504	0.365311734	0.971038229	0.293452723	0.360157537
0.071918164	0.593286146	2.152181873	0.328234775	0.260363659
0.149373901	0.578584669	1.168939287	0.298023913	0.367648754
0.081616237	0.590905773	1.332929059	0.321078952	0.30510971
0.346829856	0.597785579	1.414409628	0.357620167	0.281264621

Q8WXD2,SCG3,INFLAMMATION	Q07065,CKAP4,INFLAMMATION	P13693,TPT1,INFLAMMATION	P30044,PRDX5,INFLAMMATION	P19883,FST,INFLAMMATION
Q8WXD2	Q07065	P13693	P30044	P19883
SCG3	CKAP4	TPT1	PRDX5	FST
INFLAMMATION	INFLAMMATION	INFLAMMATION	INFLAMMATION	INFLAMMATION
0.846099947	0.707548035	1.136029265	0.341960122	0.250398877
0.783877434	0.80068129	1.017409168	0.342576953	0.263742139
0.836536271	1.034404173	0.776199818	0.281635285	0.249117798
0.953562892	0.748824737	0.954290225	0.385258849	0.267479457
1.134534122	0.740719899	1.138946515	0.385472541	0.574309368
0.52011408	0.739539956	0.826221209	0.358066635	0.318485589
0.395404172	0.528655574	1.005769703	0.221564707	0.176887009
0.615358903	0.768544126	0.875330654	0.286737003	0.247620043
0.460125718	0.443590385	1.06836158	0.222488088	0.256957203
0.389879445	0.416965522	0.910796088	0.252893174	0.265668669
0.647611271	0.501805434	0.924150678	0.243113178	0.21726083
0.764930193	1.324547884	1.483289292	0.551258156	0.495274415
0.533366826	0.775877073	0.601776636	0.238473357	0.134697969
0.55632506	0.544272104	0.573195822	0.214284589	0.133083166
0.512775771	0.378771582	1.148698355	0.197209369	0.076145363
0.488862281	0.370282943	0.758488914	0.280660901	0.129452973
0.914338327	0.627027229	1.246860445	0.29278225	0.213055323
0.925304428	0.623516654	1.337649369	0.276451825	0.234750478
0.532517189	0.651302649	0.954621014	0.353039129	0.303254298
0.496374188	0.724069457	1.048335138	0.235745151	0.115622975
0.801292012	0.620971947	0.815411382	0.567385033	0.161611302
0.347720493	0.600068873	0.904442067	0.297054593	0.386221397
0.50215338	0.66213624	0.697033548	0.359235044	0.169693122
0.38890778	0.520727318	1.542959292	0.250520401	0.159950787
0.59225895	0.621747195	0.712518807	0.253331784	0.111978992
0.507155476	0.598407433	1.070214514	0.267739147	0.239384227
0.567424363	0.413511684	0.708578698	0.265245467	0.112305462
0.495720903	0.596502463	0.519105616	0.288551353	0.179070551
0.406915178	0.688916005	0.768863821	0.340635334	0.211598309
0.806362247	0.531005975	1.531133418	0.237516559	0.232435124
0.311455942	0.453853944	1.143851685	0.283417353	0.175908853
0.733007569	0.724069457	0.656424049	0.282182417	0.139893419
0.355100673	0.579708683	0.845103533	0.197510328	0.185835754
0.701589956	0.459265399	1.38578169	0.283122831	0.094025768
0.717723468	0.588412582	1.152766248	0.318463514	0.209554877

O95866,MPIG6B,INFLAMMATION	P16455,MGMT,INFLAMMATION	Q15109,AGER,INFLAMMATION	P08727,KRT19,INFLAMMATION	P15692,VEGFA,INFLAMMATION
O95866	P16455	Q15109	P08727	P15692
MPIG6B	MGMT	AGER	KRT19	VEGFA
INFLAMMATION	INFLAMMATION	INFLAMMATION	INFLAMMATION	INFLAMMATION
0.154662999	0.604284566	0.626245397	0.39452811	0.33929216
0.118470519	0.306912647	0.479831575	0.29259966	0.41838412
0.105572062	0.426406105	0.677597285	0.786980612	0.398182025
0.093999702	0.444945333	0.499792099	0.444729497	0.395048038
0.121978603	0.548133798	0.40000779	0.6816958	0.40615435
0.111012959	0.663330607	0.539801167	0.695585614	0.311499122
0.088125295	0.584712768	0.499792099	0.438120614	0.333278464
0.099228613	0.83398885	0.495240086	0.424342181	0.258959789
0.167496169	0.533921667	0.420593949	0.31085205	0.293107137
0.208136227	0.645594414	0.344458002	0.359235044	0.416387885
0.137575931	0.621101088	0.422317519	0.493526686	0.316263726
0.212038773	1.128416761	0.858148959	0.558024357	0.542088385
0.0778424	0.559573676	0.468460723	0.456852388	0.305787216
0.091245676	0.28210419	0.415436534	0.345605959	0.222858517
0.108984887	0.332724498	0.369001862	0.315519265	0.175203075
0.122105492	0.673010072	0.309133091	0.331642315	0.303296341
0.710349044	0.280972336	0.517632462	0.653065681	0.646086842
0.101362787	0.926395403	0.454830214	0.769823704	0.288351414
0.104270286	0.855654333	0.384938533	0.353308411	0.378797838
0.101919358	0.474769776	0.53288643	0.56097174	0.287652717
0.111862625	0.595799988	0.488083538	0.304687031	0.269414611
0.1234929	0.608487698	0.251477285	0.772496338	0.420186
0.100189276	0.374594079	0.39433673	0.383287804	0.269060031
0.084447586	0.49427985	0.324637148	0.239716314	0.340281353
0.090955204	0.612592666	0.591028662	0.566402681	0.282711015
0.094745419	0.439976996	0.630688704	0.383553571	0.286876163
0.114681136	0.307508885	0.505015649	0.3672667	0.251181132
0.257777813	0.989450884	0.441718757	0.355963199	0.419255031
0.096221293	0.605248708	0.669752516	0.506874327	0.410257041
0.095292072	0.333047533	0.376546516	0.479166848	0.261503108
0.114221014	0.656151107	0.39066394	0.490899635	0.309175949
0.105681884	0.739950157	0.645057647	0.414114034	0.29498224
0.101173264	0.447481517	0.413225159	0.53170576	0.275037454
0.119063243	0.580231289	0.678490255	0.513024632	0.295903774
0.158790884	0.423959982	0.445809727	0.309154519	0.344028502

Q6ZMH5,SLC39A5,INFLAMMATION	Q4KMG0,CDON,INFLAMMATION	Q15661,TPSAB1,INFLAMMATION	Q14773,ICAM4,INFLAMMATION	P49771,FLT3LG,INFLAMMATION
Q6ZMH5	Q4KMG0	Q15661	Q14773	P49771
SLC39A5	CDON	TPSAB1	ICAM4	FLT3LG
INFLAMMATION	INFLAMMATION	INFLAMMATION	INFLAMMATION	INFLAMMATION
0.305914416	0.635119513	0.290256444	0.38291604	0.337462711
0.349460185	0.991922974	0.17343897	0.223694238	0.176556275
0.460061935	0.88902753	0.391205891	0.328257527	0.257260167
0.199740667	0.471719125	0.17343897	0.475988954	0.427767861
0.238390723	0.517130392	0.358141101	0.668083346	0.600693101
0.355223763	0.916178111	0.195725007	0.447947015	0.228457863
0.274504176	0.740565887	0.177759676	0.138648124	0.248393616
0.222812179	0.862203306	0.220339492	0.145662357	0.299058577
0.206784507	0.534402996	0.114023255	0.506804065	0.129363274
0.233808616	0.438272481	0.156236127	0.291728856	0.254334662
0.223601225	0.55091437	0.184539301	0.192056141	0.23768125
0.323357057	1.242632765	0.30283419	0.507331273	0.36846513
0.292315858	0.84434236	0.198470983	0.446273486	0.212981496
0.179679784	0.571291908	0.289312387	0.204787562	0.283948265
0.19814109	0.326442319	0.136313467	0.1301277	0.173270746
0.183340828	0.370026372	0.658337827	0.384645144	0.163504245
0.197332433	0.485889446	0.146980839	0.401730527	0.337766933
0.230302184	0.45565064	0.213484022	0.291042148	0.337860594
0.23759889	0.552635444	0.319990283	0.346229366	0.374723926
0.220034248	0.823476851	0.275705509	0.710496772	0.208410519
0.186364632	0.693323228	0.344625175	0.220522841	0.219045123
0.228663817	0.513344773	0.22581293	0.196499834	0.264089711
0.181633221	0.597495602	0.143906135	0.22131912	0.200976678
0.240715342	0.555246385	0.21283392	0.599528399	0.219029941
0.206326352	0.630295383	0.129551713	0.288691393	0.232274068
0.206069086	0.606424523	0.173475039	0.238126486	0.252280395
0.33508523	0.692650749	0.122988898	0.201604535	0.145359775
0.209467743	0.844752137	0.093993187	0.368235341	0.252140539
0.216119327	0.85174885	0.123672788	0.27100662	0.345605959
1.001317847	0.457327633	0.189609085	0.213735729	0.222318514
0.202304456	0.398762044	0.294491929	0.381882311	0.222010528
0.213735729	1.182057323	0.228378699	0.529095481	0.358811989
0.217065146	0.652794135	0.299473448	0.205199625	0.485048192
0.196813352	0.840022572	0.148784898	0.148383235	0.210953949
0.206999616	0.47519778	0.241433872	0.349218042	0.19816856

Q8N8S7,ENAH,INFLAMMATION	P42575,CASP2,INFLAMMATION	Q5T4W7,ARTN,INFLAMMATION	Q16719,KYNU,INFLAMMATION	Q16698,DECR1,INFLAMMATION
Q8N8S7	P42575	Q5T4W7	Q16719	Q16698
ENAH	CASP2	ARTN	KYNU	DECR1
INFLAMMATION	INFLAMMATION	INFLAMMATION	INFLAMMATION	INFLAMMATION
1.225185332	0.384645144	0.844517954	0.401758374	0.486563499
1.079303045	0.388665242	0.633624489	0.291506508	0.439855025
1.448540478	0.650896472	0.748565259	0.367037659	0.476021948
1.555415478	0.457454449	1.159256648	0.547033086	0.578865468
1.95125185	0.70329409	0.91092236	0.344267047	0.542577076
1.202302563	0.575345312	0.798187726	0.431400574	0.274352
0.870188587	0.526023807	1.255794153	0.255943984	0.489235162
1.00242896	0.577062705	0.71227191	0.256939393	0.443436675
1.029183334	0.491648791	0.777599933	0.303254298	0.799960128
0.916495689	0.550303725	0.656151107	0.285428243	0.5069446
1.094293701	0.581116772	0.614762044	0.322998641	0.680515534
2.690547916	0.515591362	0.777492142	0.53155836	0.56703119
0.810846089	0.513950029	0.640335544	0.190214614	0.556865184
0.803628147	0.383792919	0.736777035	0.194265282	0.311348018
0.553056968	0.487711535	0.5488942	0.253859121	0.422200444
0.505015649	0.591930623	0.680279726	0.318861098	0.498028428
1.391364098	0.525914435	0.799904681	0.386435623	0.638297101
0.838568184	0.882641814	1.13981525	0.353504381	0.496443005
1.605363498	0.585239885	0.77292482	0.391911551	0.536630143
0.663192685	0.450063353	0.522426506	0.438971749	0.415724593
1.594937621	0.60566838	0.720315074	0.297507924	0.331550377
1.759298152	0.525040275	0.760542092	0.441565695	0.754765299
1.437338636	0.44596426	0.596461118	0.317493729	0.450562766
0.707744236	0.411282046	0.557830994	0.191736911	0.466354841
1.013116443	0.613485011	0.865376591	0.312234097	0.413110605
0.653654418	2.859573524	0.659982648	0.171692628	0.683967652
0.521955964	0.657927263	1.002220533	0.173511116	0.901750361
1.051245773	0.426613049	1.179356592	0.27783495	0.525659321
0.857257187	0.668592928	0.780786493	0.263669024	0.575464964
0.802069968	0.481998312	0.936921447	0.244481974	0.521775099
0.286478743	0.381564802	0.736164456	0.183595169	0.467746901
0.898817426	0.568881472	0.632001549	0.412223884	0.37079662
0.791301831	0.623732786	0.800903317	0.266129436	0.397906122
1.497441871	0.614208337	0.877395979	0.255058485	0.596709231
1.106957628	0.490321524	0.703440352	0.295145858	1.20280269

Q9NZC2,TREM2,INFLAMMATION	Q13574,DGKZ,INFLAMMATION	P18510,IL1RN,INFLAMMATION	P12532,CKMT1A_CKMT1B,INFLAMMATION	P40259,CD79B,INFLAMMATION
Q9NZC2	Q13574	P18510	P12532	P40259
TREM2	DGKZ	IL1RN	CKMT1A_CKMT1B	CD79B
INFLAMMATION	INFLAMMATION	INFLAMMATION	INFLAMMATION	INFLAMMATION
0.435667681	1.015929296	0.308213081	0.497510886	0.623257395
0.249670972	1.336907825	0.250138668	0.357397141	1.153565561
0.287214402	0.89328968	0.323513989	0.64478943	1.457908321
0.257385021	1.41362553	0.345725758	0.528033	0.682026641
0.198058703	0.811352079	0.39978604	0.495927111	0.940956543
0.144165714	1.066142293	0.299660327	0.418964526	0.791850509
0.166442988	0.922550634	0.240982452	0.448319762	0.749395905
0.202711522	0.961860781	0.265135177	0.470478271	0.687389634
0.290719551	1.455081539	0.312992502	0.413741047	0.410769224
0.290860643	1.048698526	0.81552443	0.365235778	0.534032704
0.297672943	1.04441856	0.322394715	0.509798841	0.455050953
0.671007129	0.983774797	0.552941975	0.677973129	0.909849607
0.227462402	1.551754145	0.209351622	0.284776104	0.791905398
0.190016947	1.124668637	0.208468311	0.230509801	0.438029518
0.280563648	0.985139544	0.24400794	0.204986386	0.263395025
0.242255277	0.958532996	0.319347708	0.406830571	0.337509496
0.334250124	1.462057448	0.310012867	0.816599166	0.698145672
0.329009238	1.177396301	0.314754738	0.342671948	0.620412647
0.317493729	0.744941995	0.402092686	0.701589956	0.712518807
0.173210705	1.032470098	0.241685027	0.241902905	0.642246933
0.20064262	1.281470262	0.357298064	0.448102289	0.526753536
0.365337056	0.753406299	3.041957506	0.443313745	0.43452166
0.323133	1.259717294	0.403600537	0.477575258	0.480597151
0.202978665	0.851099672	0.21975989	0.329009238	0.453476596
0.26067064	1.246687605	0.354928419	0.418500137	0.592423182
0.307807438	1.043839571	0.323872976	0.489133439	0.50382688
0.300742361	1.271207904	0.184194259	0.392564059	0.774050716
0.300159243	0.901187995	0.251756337	0.418297129	0.471130945
0.564286602	1.091717815	0.411681351	0.482165389	0.57383187
0.775608221	1.203887014	0.623084615	0.472668294	0.325944898
0.168988854	0.718121569	0.155103162	0.223926938	0.444421341
0.223013044	0.949605371	0.175982027	0.568960341	0.590168981
0.201534677	1.260153954	0.223663229	0.499826743	0.392346436
0.304708151	0.816542566	0.197578792	0.448755025	0.424489272
0.270762529	0.788290886	0.35460874	0.581519712	0.602778557

Q96KG7,MEGF10,INFLAMMATION	P05112,IL4,INFLAMMATION	Q8TCS8,PNPT1,INFLAMMATION	Q6UXB2,CXCL17,INFLAMMATION	P80162,CXCL6,INFLAMMATION
Q96KG7	P05112	Q8TCS8	Q6UXB2	P80162
MEGF10	IL4	PNPT1	CXCL17	CXCL6
INFLAMMATION	INFLAMMATION	INFLAMMATION	INFLAMMATION	INFLAMMATION
0.740052743	1.808508025	0.788181613	0.372625936	0.08035324
0.970701751	0.149778245	0.573354768	0.316592723	0.329328666
1.023492124	1.741463216	0.320745293	0.388234438	0.357644956
0.795315945	0.335829297	0.759699091	0.375191747	0.434009943
1.085379827	0.31930344	0.479100426	0.420885584	0.448381917
1.287167674	1.387030969	0.434009943	0.365210462	0.45015695
0.733464985	0.879527139	0.710595275	0.291223766	0.226895515
0.724621742	0.584267117	0.536667341	0.328439603	0.268240689
0.543668822	1.128651433	0.33854042	0.181306179	0.233743799
0.554323471	0.238605632	0.700326702	0.210165825	0.447574578
0.563504876	1.992942381	0.769290288	0.348420158	0.173607358
1.185750108	1.417452117	0.683730648	0.301953856	0.631826345
0.732296599	1.623829321	0.504595764	0.324907286	0.165660322
0.522354087	1.325833857	0.276988887	0.315759928	0.395075421
0.359011012	1.109877153	1.200720197	0.184987539	0.092686305
0.3021004	1.306493548	0.579347155	0.19409031	0.091867603
0.827768928	1.89763168	0.627505496	0.31085205	0.355248386
0.829894586	1.137526378	1.759542059	0.470641355	0.246046006
0.631913941	1.72500435	0.504805663	0.412624102	0.465418351
0.785236968	1.673072542	0.483839346	0.487779151	0.394555457
0.625898229	0.76583208	0.66891741	0.293107137	0.238489888
0.499965344	0.976776232	0.683351612	0.192002899	0.262174632
0.729004689	1.831721286	0.535255641	0.331366577	0.311736718
0.519393549	0.436665362	0.458629163	0.250312111	0.461595159
0.598034243	0.750799711	0.665771953	0.295268631	0.322819582
0.581076494	0.463518877	0.977995682	0.229632699	0.353185985
0.537002236	0.203077175	0.678631358	0.187335991	0.332309628
0.607771108	1.759664026	0.478768453	0.252700426	0.217471764
0.868080055	1.165379631	0.63745703	0.238936639	0.293290043
0.657425809	0.737747995	0.768224564	0.496477417	0.203669238
0.456187873	0.797413536	0.471915349	0.303359417	0.391422882
0.8217093	1.048843917	0.500450749	0.322148995	0.26833367
0.426968043	1.924788596	0.690685097	0.196241218	0.182112266
0.561477455	1.599476726	0.464934697	0.169458041	0.310529019
0.688725023	0.940043876	0.486968379	0.26359593	0.432748283

Q9Y3P8,SIT1,INFLAMMATION	P45984,MAPK9,INFLAMMATION	P48061,CXCL12,INFLAMMATION	P68106,FKBP1B,INFLAMMATION	P30203,CD6,INFLAMMATION
Q9Y3P8	P45984	P48061	P68106	P30203
SIT1	MAPK9	CXCL12	FKBP1B	CD6
INFLAMMATION	INFLAMMATION	INFLAMMATION	INFLAMMATION	INFLAMMATION
2.059651144	0.53950192	0.70290421	1.723092319	0.483537606
2.404938498	0.454105684	0.855713645	1.807756044	0.368771738
2.002080523	0.445531703	1.726559437	1.837825767	0.323985241
2.887057818	0.558721019	0.914782074	4.571919495	0.546578266
3.635364441	0.543367431	1.269446857	2.584168243	0.348903506
1.380317353	0.571886201	0.699938466	1.845101267	0.401953356
2.457344285	0.426760927	0.97063447	2.283425942	0.259372962
1.41558659	0.397768242	0.933550518	1.936162444	0.311218559
2.081321739	0.318573904	0.916749831	1.974927921	0.34687794
2.740425662	0.35257449	0.826908727	2.22792397	0.369564991
2.205491194	0.356630006	0.525914435	2.162200036	0.430385089
2.411782822	0.796584879	1.394163729	1.727397372	0.534365955
1.52414483	0.508669325	0.672497123	1.91149311	0.317779948
1.240309356	0.263340259	0.826736794	1.984946331	0.274085897
1.117364585	0.389393309	0.370745221	2.306011967	0.301932927
1.975749441	0.484678503	0.4430987	2.372980097	0.266147884
2.363459266	0.392319242	1.045070305	1.76210513	0.511994423
2.00319102	0.412824357	0.858505927	2.215296799	0.360432248
2.521531066	0.454735645	0.956740781	2.04570767	0.337415932
2.326240083	0.442331534	0.799960128	1.943692504	0.503373091
1.501391271	0.506277404	0.723467443	1.782003289	0.231261979
2.502551919	0.415378946	0.746027129	2.276788058	0.318595987
1.983295985	0.369590608	0.546805629	2.706634348	0.466225558
1.327581104	0.461307291	0.897385638	1.851635336	0.315869381
1.812272625	0.507296109	0.602694999	1.879436276	0.597909898
1.477030892	0.395020656	0.468071231	1.994739013	0.427678919
2.109059798	0.469500956	0.99660934	2.126823039	0.285092106
2.489058262	0.411510173	1.038427144	2.153375627	0.329123284
1.185914499	0.452973953	0.978877342	1.859480991	0.363140564
1.584908991	0.252525328	0.403488651	2.123729469	0.36946254
1.275355988	0.384032416	0.877943498	2.393463823	0.257956553
1.802001235	0.456694082	0.459679425	2.053521426	0.336668349
2.088113309	0.393708564	0.698532914	2.338040628	0.223183148
1.847276724	0.269078682	0.477442864	1.682725673	0.383314373
2.636456147	0.419284092	0.530711604	1.929998866	0.38724003

P23229,ITGA6,INFLAMMATION	Q03426,MVK,INFLAMMATION	Q8WXI8,CLEC4D,INFLAMMATION	P49763,PGF,INFLAMMATION	O43508,TNFSF12,INFLAMMATION
P23229	Q03426	Q8WXI8	P49763	O43508
ITGA6	MVK	CLEC4D	PGF	TNFSF12
INFLAMMATION	INFLAMMATION	INFLAMMATION	INFLAMMATION	INFLAMMATION
0.954157941	0.412223884	0.328963631	0.45021936	0.464934697
0.682357643	0.273876996	0.521919786	0.578344092	0.582528282
0.815072332	0.616127143	0.569197015	0.453979797	0.530895567
0.924791473	0.545858908	0.155944005	0.36948815	0.383846128
0.847861193	0.974747193	0.466710555	0.462716356	0.584834368
1.043767221	0.505190705	0.347816915	0.494794031	0.452911162
0.859935286	0.290256444	0.255695735	0.38858443	0.42791614
1.009541348	0.187478882	0.381538355	0.495411753	0.366834187
0.89925364	0.432028981	0.518242774	0.349169633	0.407112663
1.124746595	0.290659104	0.381882311	0.317009944	0.290336931
0.954819543	0.431909214	0.321368404	0.353137026	0.432718288
1.121943481	0.316834205	0.562451265	0.854172881	0.684015063
1.010661586	0.407649175	0.189714256	0.33743932	0.424901399
0.727692073	0.426968043	0.280077889	0.353332901	0.40522638
0.869887055	0.209991087	0.146665352	0.227525476	0.331527396
0.901125531	0.380376494	0.365362381	0.288591357	0.430683512
1.404055535	0.502362263	0.450531536	0.475428403	0.691978923
0.947961255	0.548399819	0.274466124	0.299909681	0.41039925
1.039435329	0.622393975	0.190914687	0.406886974	0.45584018
0.810228085	0.532443372	0.252893174	0.413167878	0.528618932
0.862801147	0.403992384	0.247997933	0.397547733	0.322439412
0.928774328	0.58406466	0.240032223	0.456820722	0.315366211
0.753876445	0.421323415	0.322819582	0.327462135	0.391233008
1.319050683	0.430385089	0.212878182	0.417572903	0.384405265
1.089525528	0.249722895	0.403488651	0.387669731	0.519609603
1.003054503	0.236055827	0.355716549	0.371877663	0.507471955
1.044129025	0.413053339	0.510293793	0.376964351	0.457422742
1.022499402	0.386837618	0.466937059	0.441994401	0.428628594
0.943765278	0.364906815	0.633448835	0.463775978	0.473192791
1.178539408	0.534958914	0.211847792	0.305236628	0.438424401
0.958333695	0.357521027	0.388046111	0.318353162	0.403404757
0.940239373	0.712963438	0.237236846	0.480097723	0.475758059
0.802125565	0.318706423	0.195264285	0.358463964	0.314645671
2.444094386	0.3441239	0.302918165	0.35130597	0.427945802
0.797855838	0.35128162	0.546199538	0.347768701	0.468298395

P10144,GZMB,INFLAMMATION	P36941,LTBR,INFLAMMATION	Q9UNE0,EDAR,INFLAMMATION	P27930,IL1R2,INFLAMMATION	Q8NDB2,BANK1,INFLAMMATION
P10144	P36941	Q9UNE0	P27930	Q8NDB2
GZMB	LTBR	EDAR	IL1R2	BANK1
INFLAMMATION	INFLAMMATION	INFLAMMATION	INFLAMMATION	INFLAMMATION
0.164379239	0.363972158	0.436181353	0.424401012	1.05270412
0.254881753	0.604661656	0.483135577	0.563856519	1.042538022
0.342339579	0.520980038	0.50145773	0.655741906	1.117829381
0.300263288	0.34596548	0.270800067	0.43669563	0.838451942
0.410058032	0.354166583	0.326080483	0.52355028	1.529860386
0.327099169	0.389447294	0.369308916	0.437756347	1.138236225
0.25458159	0.365742453	0.355420795	0.429937841	1.022499402
0.125460055	0.556363623	1.272618501	0.336295178	1.041165924
0.283692517	0.282593463	0.382253072	0.378325519	0.970163629
0.276835334	0.302142283	0.349557089	0.338587355	1.609820687
0.223461779	0.379691603	0.386864432	0.37030861	0.925112036
0.351330322	0.74406471	0.453256621	0.57506622	1.487820004
0.099070545	0.311132283	0.351695797	0.331297679	1.294953236
0.366935909	0.306211421	0.271551926	0.343147322	0.96620418
0.185797114	0.177451909	0.401869781	0.347070343	0.800903317
0.800625793	0.250242719	0.431131536	0.364578149	1.956533754
0.150266987	0.456725739	0.413225159	0.384991901	2.159803408
0.268817693	0.380112928	0.482365958	0.386355275	1.23387821
0.295493848	0.376624824	0.278181812	0.436574569	1.602584024
0.19924287	0.321502085	0.473225591	0.439123911	1.159738869
0.22879065	0.409972771	0.283397709	0.3304033	0.820457211
0.213336097	0.356086587	0.477674577	0.397713103	1.380508719
0.142773487	0.387911647	0.366910475	0.446211623	0.970499921
0.232951254	0.351257272	0.439245679	0.330930461	0.872483649
0.344911945	0.337930858	0.352281349	0.405535467	0.919741281
0.253753566	0.326555475	0.355716549	0.388799967	1.215289478
0.330586565	0.464741376	0.523223774	0.598117154	1.385493555
0.305215472	0.380719402	0.353137026	0.228663817	1.393197704
0.416763258	0.444390537	0.430862666	0.529792748	1.082074627
0.227399344	0.312688919	0.342434508	0.479898098	1.18304094
0.187439901	0.307061598	0.439276127	0.437908088	0.980982972
0.220095263	0.433348615	0.300450661	0.40934807	0.954621014
0.281069731	0.352501182	0.358712519	0.296622512	0.773460757
0.191683758	0.281050249	0.44590244	0.360332329	1.118992216
0.274142897	0.311002913	0.407169104	0.466904695	1.910433447

Q8IYS5,OSCAR,INFLAMMATION	O15444,CCL25,INFLAMMATION	Q99435,NELL2,INFLAMMATION	P55145,MANF,INFLAMMATION	P58294,PROK1,INFLAMMATION
Q8IYS5	O15444	Q99435	P55145	P58294
OSCAR	CCL25	NELL2	MANF	PROK1
INFLAMMATION	INFLAMMATION	INFLAMMATION	INFLAMMATION	INFLAMMATION
0.297301779	0.27198519	1.178702799	0.635339667	0.193848302
0.329032044	0.229903447	1.036701101	0.509057313	0.432868283
0.369616227	0.354879219	0.982207674	0.533773653	0.310916697
0.433438737	0.329465658	1.008701984	0.511426918	0.294206291
0.38111545	0.676986983	1.129042661	0.893847117	0.217140389
0.275820196	0.158066111	0.5465025	0.562490252	0.320634151
0.329739814	0.144957311	0.608023926	0.668500248	0.248893421
0.348516774	0.126964758	0.764824159	0.534069722	0.325267819
0.304518124	0.146117411	0.879527139	0.647745952	0.191060308
0.333602036	0.159596398	0.613442489	0.672543738	0.22540634
0.329191731	0.270931492	0.597992792	0.620326645	0.276490152
0.361508131	0.275113721	1.204387801	0.723166624	0.461371246
0.304222761	0.141003219	0.672683604	0.562802249	0.308747637
0.245177756	0.107969819	0.868742185	0.55064713	0.223926938
0.273649286	0.103063114	0.706078259	0.461595159	0.18322649
0.311607098	0.130344354	0.501006074	0.664342907	0.278954166
0.396337121	0.230350079	0.99633306	1.03469101	0.283083585
0.397299808	0.195223685	1.184846363	0.673990424	0.191179535
0.257402862	0.255394613	0.936596791	0.693082982	0.269041382
0.429878243	0.277392368	0.652206173	0.616939105	0.260237361
0.310206323	0.245994847	0.774533744	0.520222246	0.18230171
0.310744336	0.310356873	0.810228085	2.3690358	0.30149375
0.291445898	0.294145119	0.902625849	0.61339997	0.195386135
0.238390723	0.235092432	0.483638165	0.511604195	0.270256271
0.363593925	0.138034423	0.96989468	0.586498775	0.334111142
0.273364916	0.146198458	0.674738318	0.524785586	0.152058672
0.293595142	0.21564049	0.838103312	0.65094159	0.382624192
0.266923827	0.169987433	0.916495689	0.446861606	0.333116795
0.333972218	0.474868512	0.792399569	0.68136512	0.320478616
0.322126666	0.170885276	0.845806762	0.896577376	0.30477152
0.207631897	0.351939659	0.503617388	0.551258156	0.543329769
0.281030769	0.278606341	0.906010704	0.600318486	0.343028417
0.281713382	0.303002164	0.536407011	0.426465222	0.230318148
0.268594189	0.178624267	0.709709243	0.662687219	0.200990609
0.347648194	0.314253344	0.792949009	0.6341957	0.295493848

P30613,PKLR,INFLAMMATION	Q9UJU6,DBNL,INFLAMMATION	O76096,CST7,INFLAMMATION	Q8IU57,IFNLR1,INFLAMMATION	P09038,FGF2,INFLAMMATION
P30613	Q9UJU6	O76096	Q8IU57	P09038
PKLR	DBNL	CST7	IFNLR1	FGF2
INFLAMMATION	INFLAMMATION	INFLAMMATION	INFLAMMATION	INFLAMMATION
2.020202623	0.379297035	0.239982315	0.647252259	0.905633983
1.510367891	0.5893514	0.323401887	0.753824192	0.815807118
2.221601413	0.78905622	0.36180895	0.705393409	1.017973496
1.561681229	0.776791866	0.342862019	0.548171793	1.080425803
1.897237119	0.829779546	0.328234775	1.62079317	1.080875233
1.530815061	0.716431164	0.235402248	0.63055757	0.943896121
1.733514615	0.68577157	0.288491357	0.772121612	1.0060486
0.673943709	0.620455652	0.279903222	0.555400353	0.755236293
1.816422736	0.809778924	0.318772703	0.501666324	0.826393035
1.592838503	0.734788015	0.481964904	0.496236583	0.931223906
1.057531033	0.540400157	0.257028457	0.491682871	0.990960873
3.013625195	1.060026256	0.726180452	0.568526696	1.051683065
0.934845592	0.566677568	0.22298213	0.730522189	1.001942697
1.356228249	0.736215485	0.35045473	0.397823388	0.760120474
1.182794959	0.352525616	0.273308077	0.259552808	0.80446413
1.72476523	0.76731986	0.721814485	0.581116772	0.789001528
5.558516898	1.306493548	0.662503509	0.51630662	0.819150248
1.382232207	0.711334479	0.322461762	0.436060434	0.899627707
1.787322532	0.722264917	0.407733952	0.730876727	0.752779894
1.10030234	0.406407801	0.311434354	0.628245353	0.864357475
1.160462578	0.564638732	0.265945033	0.378063375	1.009681309
2.32366163	0.645057647	0.35150083	0.733464985	1.179274848
1.483186482	0.587801115	0.341036959	0.588004866	0.732144338
0.796253658	0.558179096	0.209409675	0.441565695	0.876484206
1.048407806	0.563895604	0.296150002	0.621488672	0.998891579
1.608482233	0.579949827	0.464934697	0.443713391	0.953562892
1.584030374	0.633009914	0.301640071	0.616725327	1.021436841
4.636062449	1.017056622	0.472635533	0.814676953	1.070882357
2.784271696	0.972992102	0.515627102	0.655423815	0.789877044
1.00242896	0.363165736	0.292417184	0.550303725	0.841421157
1.291188827	0.626071789	0.190967627	0.389015623	0.914274952
1.41705917	0.41359768	0.281889178	0.534773544	0.792729187
1.095507985	0.358811989	0.237500096	0.465128098	0.828170661
0.916432165	0.568842042	0.208064105	0.580311732	0.857911063
4.349350574	1.149096533	0.627810037	0.568920905	0.792289726

Q9UHC6,CNTNAP2,INFLAMMATION	Q12778,FOXO1,INFLAMMATION	P55957,BID,INFLAMMATION	Q6DN72,FCRL6,INFLAMMATION	P30838,ALDH3A1,INFLAMMATION
Q9UHC6	Q12778	P55957	Q6DN72	P30838
CNTNAP2	FOXO1	BID	FCRL6	ALDH3A1
INFLAMMATION	INFLAMMATION	INFLAMMATION	INFLAMMATION	INFLAMMATION
1.363297101	0.961660789	0.680326881	0.441902501	1.149096533
1.730153452	1.119923354	0.437938442	0.692410736	0.684584247
1.374874563	0.994125571	0.426760927	0.325786787	0.915924128
1.626194702	1.103357258	0.473652203	0.369283319	1.21959319
1.782373885	1.197146739	0.342600699	0.316395284	1.463172637
0.561866776	1.042827115	0.680279726	0.772978397	0.827711553
0.955945318	0.977114815	0.393272169	0.402427277	0.809330012
0.633185446	1.921322899	0.34004557	0.351817707	1.071847755
0.878917709	0.734788015	0.479166848	0.267609271	0.552252519
0.637412846	0.966338134	0.539427135	0.422844758	0.981118975
0.688963758	0.919295128	0.567070495	0.364628693	1.196234308
0.987737781	1.24065329	0.622092061	0.540587478	2.482166685
1.589309389	2.043015288	0.462716356	0.270481157	0.933679945
1.194742739	0.880808319	0.567345706	0.368541758	0.936661713
0.852398524	1.064812934	0.485451812	0.271533104	0.833064439
0.838975159	0.961260928	0.324097546	0.204489688	0.489676206
1.030682513	1.384533537	0.652748888	0.332493951	0.834567127
1.234134814	1.137841811	0.556556477	0.544309832	0.904379378
1.21959319	1.202719321	0.438910899	0.224751087	0.733617521
0.980439151	3.135075094	0.754190038	0.396035044	0.804519893
0.865076726	0.691116103	0.459870641	0.247499926	0.845572287
1.247119749	2.037923646	0.52898547	0.39433673	0.8456309
1.209994089	1.099235117	0.46047668	0.386141093	0.669381229
0.568093381	0.658702988	0.364275027	0.308148997	0.563856519
1.033186	1.068880078	0.581116772	0.461051559	0.85862495
0.701346846	1.033830736	0.496993883	0.415695778	0.755812351
1.059585495	1.322162957	0.29629373	0.731130073	1.087940759
0.903063912	1.171778594	0.427767861	0.300159243	0.729307937
0.817505306	0.678396203	0.477045903	0.584267117	1.012976005
0.94828985	1.225270258	0.646758942	0.392020227	1.119380096
0.792509426	1.003680436	0.420827241	0.40649232	0.774372701
1.066290101	0.761861153	0.570184213	0.301619164	0.634283624
0.850097369	0.832775771	0.519465557	0.298085892	0.69341935
1.274560627	0.787799278	0.309690708	0.417891407	0.795701928
1.179438342	1.221031145	0.537002236	0.558798479	0.904065998

Q9BZZ2,SIGLEC1,INFLAMMATION	Q9Y3D6,FIS1,INFLAMMATION	Q13007,IL24,INFLAMMATION	P63241,EIF5A,INFLAMMATION	P37235,HPCAL1,INFLAMMATION
Q9BZZ2	Q9Y3D6	Q13007	P63241	P37235
SIGLEC1	FIS1	IL24	EIF5A	HPCAL1
INFLAMMATION	INFLAMMATION	INFLAMMATION	INFLAMMATION	INFLAMMATION
0.359459217	0.220798152	0.837464534	1.335888872	0.8783087
0.462844666	0.218817496	0.481564183	0.953034271	0.998130253
0.477972659	0.237056031	0.925946023	0.966740106	0.983093132
0.452189689	0.168089324	0.903690086	1.325558187	0.744838732
0.298602883	0.18595172	0.873512347	0.672357295	0.927873476
0.346277367	0.174609021	0.991373087	1.319965297	0.805245166
0.408129811	0.138082271	0.823305632	0.931159361	0.603823998
0.361608376	0.145017609	0.786380798	1.229268443	0.684963966
0.375347818	0.155415252	0.711383787	1.111725029	0.623343803
0.39280903	0.126753721	0.677597285	1.439931319	0.531263683
0.310658191	0.130461859	0.79603292	1.36197479	0.644566002
0.464709164	0.308170357	0.554707832	1.644444137	1.464288675
0.289713737	0.121615583	1.029040669	1.191682575	0.680091139
0.29259966	0.130778746	0.642825917	0.935882944	0.572084436
0.269657488	0.130353389	0.913578117	1.331636204	0.414832261
0.448910579	0.191192787	0.523187508	1.039939788	0.732905959
0.43419048	0.32789368	1.01150258	1.581726323	0.994952806
0.419255031	0.202739626	1.079153433	1.279340241	0.983570248
0.351988451	0.189215214	0.926010206	1.169020314	1.040733005
0.495411753	0.1185198	0.870550563	0.785944857	0.67007756
0.440160015	0.152745311	1.079078634	1.266634254	0.706616822
0.542426663	0.199712979	1.117054829	0.738566638	0.87977103
0.408752653	0.177267504	0.835666959	0.908211368	0.795371074
0.242810043	0.127493889	0.637943252	1.148618736	0.366097544
0.413282448	0.133943827	0.607981782	1.342386396	0.716431164
0.243281749	0.178872065	0.79874118	0.931159361	0.561749952
0.403097293	0.1537651	1.060687741	1.169830898	0.579628324
0.3075302	0.191126536	0.742519078	0.800903317	0.786380798
0.476285984	0.145874539	0.598200076	0.739591218	0.708431369
0.289192091	0.244736299	0.936791571	1.092777739	0.508281631
0.320545264	0.126595673	0.66213624	0.942784536	0.527301498
0.534625294	0.172420115	0.776415056	1.383190629	0.672730233
0.418442125	0.114704985	0.779921053	1.028256363	0.484174834
0.352330189	0.099607625	1.103969259	1.641938397	0.661998567
0.358687656	0.142951732	0.896328826	1.570038569	0.79603292

P80098,CCL7,INFLAMMATION	Q9Y5A7,NUB1,INFLAMMATION	P36959,GMPR,INFLAMMATION	P01588,EPO,INFLAMMATION	Q12933,TRAF2,INFLAMMATION
P80098	Q9Y5A7	P36959	P01588	Q12933
CCL7	NUB1	GMPR	EPO	TRAF2
INFLAMMATION	INFLAMMATION	INFLAMMATION	INFLAMMATION	INFLAMMATION
0.287872124	0.81298464	1.616529684	0.429669715	0.784856061
0.314057363	0.747994724	1.666359868	0.408526055	0.742570548
0.280855508	0.714794283	2.016145838	0.601359661	1.016351897
0.34476853	0.819945543	1.503265673	0.489710149	0.803349679
0.398126829	1.116590355	0.891125172	0.799960128	1.025907032
0.271570749	0.603531092	1.742429156	0.436332548	1.033114388
0.33662168	0.654470468	1.515506458	0.454137161	0.764718139
0.270800067	0.6341957	0.98746396	0.437938442	0.541862983
0.259301058	0.811577065	1.352004544	0.226282983	1.064075117
0.425726854	0.808825283	1.550786413	0.40615435	0.886135976
0.297281172	0.839906128	1.176335837	0.489438671	0.732093591
0.50782383	1.23738976	2.400275491	0.374127002	1.041887855
0.446397236	0.883498751	1.26988689	0.306529961	0.935104822
0.302519491	0.616255277	1.690675755	0.301911999	0.590660074
0.264859654	0.718270914	1.43296165	0.366885044	0.833699861
0.220721642	0.811633321	1.757835417	0.273516543	0.842705239
0.464419354	1.132569827	4.218411983	0.608909615	1.301071277
0.272645831	1.092247649	2.353324064	1.011081996	0.876423455
0.271138145	0.643316234	1.395323845	0.36642758	0.716729182
0.218756835	0.973734255	1.443528909	0.335178148	0.709512497
0.345007589	0.746958502	1.124045161	0.340399306	0.596171783
0.354289349	0.929869394	1.840885632	0.666002732	1.046447548
0.28255429	0.674037144	1.270062945	0.388853869	0.792784137
0.338024565	0.706127202	1.301071277	0.328507907	0.613952948
0.51132058	0.988422666	1.066364014	0.357793727	0.849626106
0.341155174	0.905885113	2.04755187	0.295780736	0.898692832
0.362662631	1.036772962	1.70385197	0.372910158	0.793003974
0.34075341	1.118139352	2.934673492	0.351964054	1.205139371
0.33081579	0.753197439	2.173168439	0.319458404	0.899378312
0.572798651	0.755498083	1.178539408	0.39763041	0.909849607
0.214047071	0.872846581	1.614402149	0.264126324	0.688390933
0.252280395	0.75691332	1.49350286	0.352305768	0.645325974
0.275132791	0.932257239	1.497545669	0.302855182	0.588779767
0.311823162	0.478469875	1.251622935	0.328007339	0.773407146
0.440434687	0.890939887	3.435690312	0.420681418	1.241513544

P0DMV8,HSPA1A,INFLAMMATION	Q96SB3,PPP1R9B,INFLAMMATION	O14904,WNT9A,INFLAMMATION	Q99685,MGLL,INFLAMMATION	O60542,PSPN,INFLAMMATION
P0DMV8	Q96SB3	O14904	Q99685	O60542
HSPA1A	PPP1R9B	WNT9A	MGLL	PSPN
INFLAMMATION	INFLAMMATION	INFLAMMATION	INFLAMMATION	INFLAMMATION
0.430295602	0.791576123	0.344649063	0.427560357	0.316834205
0.398955572	0.93770108	0.383686524	0.35370046	0.111483337
0.420448208	1.142187895	0.333509555	0.443375206	0.230334113
0.308769038	1.024059826	0.47711204	0.413741047	0.070913298
0.452722841	1.509739877	0.447357466	0.536518566	0.29078001
0.339621571	1.147186538	0.439367481	0.348178735	0.04336876
0.360757177	0.769023718	0.401118386	0.326759254	0.057284951
0.310270836	0.806865439	0.294043193	0.342909553	0.081933656
0.288831501	1.17047977	0.437665327	0.412824357	0.121514468
0.285507391	1.218241368	0.331803268	0.336225255	0.060170494
0.292904041	0.791960291	0.293127454	0.388746071	0.341675806
0.606298433	1.25422832	0.296047382	0.529976392	0.268389475
0.243264887	0.928774328	0.446799662	0.332079369	0.05704325
0.268017666	0.648105238	0.347166585	0.346349381	0.070751277
0.272551355	0.592464247	0.349557089	0.474934347	0.061345443
0.581882596	0.934910392	0.314602055	0.42566784	0.099704332
0.383101877	1.268303484	0.341060598	0.500867185	0.179592624
0.354461293	0.805412629	0.621273318	0.491785124	0.261684431
0.367954683	0.903189112	0.459297234	0.434853093	0.105843164
0.329991324	1.159015612	0.328507907	0.270856384	0.214760415
0.303927685	0.76530143	0.304644796	0.417688695	0.122402082
0.396199784	0.923574343	0.31024933	0.514912786	0.129776404
0.366554596	0.912818541	0.36093226	0.454137161	0.160461601
0.293961679	0.940826108	0.324547152	0.321836531	0.038393343
0.289452796	0.872544127	0.283594213	0.327961871	0.179804371
0.311779937	0.618609117	0.408865999	0.403852395	0.519681642
0.355051449	0.735297508	0.345294677	0.306062883	0.119833236
0.412681308	1.389629225	0.324457181	0.509622189	0.031884521
0.379454813	0.85583228	0.507718242	0.364275027	0.297322387
0.255695735	0.799738363	0.447636629	0.39713461	0.19548096
0.311499122	0.785781442	0.407282011	0.25368322	0.51192345
0.303212261	0.892856358	0.33008283	0.281186649	0.128327298
0.259031598	0.7018818	0.421206615	0.342078657	0.124343242
0.333532673	0.867298188	0.322327682	0.283515595	0.052515969
0.43642329	1.204304322	0.392999668	0.494691152	0.137680868

Q13219,PAPPA,INFLAMMATION	Q969V3,NCLN,INFLAMMATION	Q04637,EIF4G1,INFLAMMATION	Q01151,CD83,INFLAMMATION	P01903,HLA-DRA,INFLAMMATION
Q13219	Q969V3	Q04637	Q01151	P01903
PAPPA	NCLN	EIF4G1	CD83	HLA-DRA
INFLAMMATION	INFLAMMATION	INFLAMMATION	INFLAMMATION	INFLAMMATION
0.770410889	0.659205416	0.482131969	0.564560462	1.425333973
1.145914973	1.088846059	0.464676954	0.737799134	0.730370297
1.172103525	0.526753536	0.562802249	0.718420289	1.372113667
0.672497123	0.840954704	0.72271563	0.543066208	1.127009753
1.334593146	0.57990963	0.690063007	0.785672517	0.940565291
1.745935182	0.589678296	0.388826917	0.465934802	1.323446614
0.947567091	0.715835501	0.415724593	0.521485846	0.772121612
0.87151657	1.458009379	0.48173111	0.687199075	0.763605818
0.826908727	0.893103945	0.463711689	0.467163673	1.082975046
0.382385574	0.798077082	0.501735874	0.428598884	1.226459845
0.711531731	0.793553833	0.394063491	0.488659011	0.619896816
0.627897076	0.651934984	0.89925364	0.76302382	1.425926876
0.619123875	1.26918291	0.451187811	0.576343173	1.186078913
0.970096385	0.463615273	0.347142522	0.434822952	0.871637397
0.629771336	0.520474721	0.406915178	0.328553451	0.837987135
0.919805035	0.702806773	0.554823192	0.471359595	1.195156877
0.683683257	1.125526481	0.902125466	0.738566638	0.572401755
0.733515827	1.179520097	0.367343079	0.480164284	1.21942413
0.868200405	0.826335756	0.500936625	0.544951597	0.583619503
1.208066607	2.65718744	0.321346129	0.484376239	1.38520548
0.911743554	0.70578467	0.425166549	0.433318579	1.09065892
0.734075318	1.209155676	0.59691607	0.381961729	0.704465031
0.916876928	0.870671256	0.486867127	0.403684472	1.054091423
0.462427788	0.948684315	0.373013565	0.352672258	0.596171783
1.239965517	0.848566718	0.401619159	0.633053792	1.208569132
0.600776381	0.680326881	0.474177792	0.461819181	1.21959319
1.194825555	0.978877342	0.433408694	0.49768334	0.943503647
0.614719434	0.805635968	0.715042054	0.368976285	0.827023368
0.658474739	0.907519155	0.489845944	0.487069652	1.279340241
0.503094039	0.897323438	0.391287248	0.433438737	0.612040913
1.04630249	0.813379198	0.347913364	0.389258379	1.115739322
0.503338201	0.805524291	0.391341496	0.58406466	0.77362161
0.748254005	0.986027644	0.475000191	0.363644333	0.868200405
0.753093031	0.805803513	0.464194071	0.362813489	0.776791866
0.607813237	0.611362514	0.73907875	0.489065635	0.918658142

O00241,SIRPB1,INFLAMMATION	Q06520,SULT2A1,INFLAMMATION	Q9BT73,PSMG3,INFLAMMATION	P12872,MLN,INFLAMMATION	P25942,CD40,INFLAMMATION
O00241	Q06520	Q9BT73	P12872	P25942
SIRPB1	SULT2A1	PSMG3	MLN	CD40
INFLAMMATION	INFLAMMATION	INFLAMMATION	INFLAMMATION	INFLAMMATION
0.205128521	0.722365051	1.810891363	0.246523998	0.402845906
0.474539472	0.614123196	2.080023748	0.196513455	0.449221848
0.354412158	0.604619746	3.004656359	0.314188003	0.356605288
0.301723715	0.653110949	1.610601967	0.181570282	0.343956971
0.279670502	0.595098341	1.745209219	0.54231388	0.38533897
0.426140182	0.866216765	2.01084238	0.373815942	0.35998283
0.526972653	0.703342841	1.972875614	0.238704886	0.278413293
0.531153222	0.754765299	1.487613762	0.324682155	0.316110311
0.237220402	0.753928701	1.5636309	0.129076655	0.305511798
0.342862019	0.792454496	2.064224667	0.29889279	0.220538127
0.278393995	0.606718833	1.374207631	0.300846609	0.27308084
0.486630955	0.77271055	3.366851289	0.208931221	0.537262854
0.340470097	0.86934456	1.438235574	0.181406745	0.269246594
0.237780119	0.697130184	2.009031243	0.176018625	0.218650719
0.138638514	0.773299937	2.070673371	0.163062845	0.232129213
0.369283319	0.697903755	2.54823795	0.125138706	0.292761957
0.382518121	0.758751832	4.189563671	0.126657113	0.371439719
0.311844777	0.745045273	1.956126947	0.467455196	0.366021424
0.261086542	0.685961732	1.267073312	0.195318431	0.238953201
0.216344148	0.814846378	1.453770968	0.244990889	0.300076033
0.320878715	0.543141498	1.348261183	0.166569943	0.321903462
0.372032355	0.83613048	2.634264116	0.266591003	0.449003937
0.389555287	0.504071398	1.416666332	0.173294768	0.351403387
0.282221538	0.694092575	1.440530294	0.175994226	0.247963555
0.33028881	0.881113637	1.894608812	0.28048587	0.319901575
0.331688293	0.614676826	2.784464694	0.192776353	0.398762044
0.431460383	0.813491964	2.492856781	0.228331214	0.353112549
0.340281353	0.664803554	3.959449885	0.187751976	0.389258379
0.389150469	0.691020301	3.341277948	0.231181843	0.342339579
0.306955197	0.701055225	1.613730878	0.215565768	0.327939139
0.257688489	0.701833151	1.737123108	0.254352292	0.220966566
0.334412342	0.705833593	1.647410398	0.115952031	0.343052194
0.256192474	0.596419776	1.706688777	0.222072091	0.297363607
0.238341156	0.816599166	1.542531552	0.166720105	0.302939163
0.386515989	0.711285175	4.281451551	0.115230937	0.330059951

O00339,MATN2,INFLAMMATION	Q9C035,TRIM5,INFLAMMATION	P41217,CD200,INFLAMMATION	O60884,DNAJA2,INFLAMMATION	Q96RJ3,TNFRSF13C,INFLAMMATION
O00339	Q9C035	P41217	O60884	Q96RJ3
MATN2	TRIM5	CD200	DNAJA2	TNFRSF13C
INFLAMMATION	INFLAMMATION	INFLAMMATION	INFLAMMATION	INFLAMMATION
0.649139297	0.800847805	0.498581065	0.391558562	0.734533401
0.618566239	1.109646384	0.549465193	0.382677239	0.578344092
0.906513242	1.080725402	0.556363623	0.517202086	0.984934711
0.614719434	1.350880445	0.372238711	0.430057062	0.622652875
0.78905622	1.025764821	0.542163539	0.820400343	0.843757309
0.802403609	0.811014717	0.382306067	0.378404198	0.523006217
0.818015452	0.688247801	0.393244911	0.445655248	0.686247075
0.840605033	0.803572445	0.385499261	0.422874069	0.615614876
0.597247163	0.966472106	0.29711637	0.517596584	0.387401112
0.550570799	0.935493802	0.294573591	0.338258947	0.64069072
0.603112901	0.914972317	0.362260648	0.404244486	0.454609583
0.982275758	0.964999433	0.775446954	0.645236519	0.836304366
0.898194629	1.221708416	0.453885404	0.364148801	0.610388633
0.559108429	0.862322841	0.257617053	0.479399397	0.307359717
0.37845666	0.726532882	0.313296379	0.252507825	0.241266581
0.441841244	0.918148871	0.293168093	0.466581173	0.404636958
0.723216752	0.827998466	0.423284627	0.723969086	0.659342508
0.612295506	0.928452495	0.461659154	0.496959435	0.940826108
0.680185426	1.177559534	0.408809322	0.545707584	0.515234106
0.582366793	0.925368567	0.347238784	0.375816418	0.692074858
0.925817668	0.538120062	0.320145581	0.291183397	0.42065226
0.50662845	1.35698051	0.353357393	0.445593471	0.516593
0.531963808	0.808432934	0.349508634	0.497304021	0.563700206
0.459138081	0.912502237	0.331159925	0.340658946	0.206226266
0.423578128	0.940500099	0.375920631	0.225781628	0.768703957
0.753406299	0.7005209	0.422962012	0.338071429	0.610430943
0.402510968	0.869887055	0.342933322	0.339386245	0.590537262
0.450250568	0.752258287	0.233905874	0.640513108	0.699211103
0.764400168	0.75917269	0.377722859	1.034547582	0.46852567
0.430623811	0.965802431	0.353455378	0.356852553	0.451907686
0.47168643	0.640468712	0.275457185	0.381908782	0.449346416
0.713457798	0.495892737	0.361357815	0.376859848	0.585361595
0.504490847	0.728146172	0.236990314	0.285072346	0.404160434
0.533884659	0.774694821	0.351890873	0.312992502	0.602402641
0.692698762	0.846569255	0.35150083	0.548399819	0.8217093

Q8NFT8,DNER,INFLAMMATION	O95379,TNFAIP8,INFLAMMATION	P09238,MMP10,INFLAMMATION	P01137,TGFB1,INFLAMMATION	Q9UDT6,CLIP2,INFLAMMATION
Q8NFT8	O95379	P09238	P01137	Q9UDT6
DNER	TNFAIP8	MMP10	TGFB1	CLIP2
INFLAMMATION	INFLAMMATION	INFLAMMATION	INFLAMMATION	INFLAMMATION
0.52380437	1.111185748	0.266554049	0.348178735	0.473291199
0.340493698	0.928452495	0.189793173	0.493321476	0.445624359
0.354215684	1.079527504	0.339174591	0.456124636	0.2906994
0.461755164	0.609543041	0.362536963	0.473914924	0.270893935
0.489676206	0.781273725	0.18911032	0.401007187	0.692362744
0.497476403	0.774802224	0.167159818	0.33503878	0.560272272
0.380007553	0.694670145	0.220095263	0.286896048	0.851807891
0.292498271	0.298023913	0.495617832	0.298375296	0.728297602
0.338001136	1.118682008	0.176727689	0.214403446	1.682142586
0.29738422	1.179847175	0.272362503	0.29761105	0.363694749
0.350649117	0.530527706	0.121649306	0.282769809	0.667204073
0.470380448	0.875330654	0.321412958	0.453916866	0.452440506
0.342173515	0.664066672	0.194953235	0.330999284	0.424813053
0.268780429	0.624468195	0.138034423	0.198787645	0.290578527
0.282867827	0.757700707	0.147276587	0.232032693	0.474572366
0.338329293	0.728146172	0.361783872	0.257456393	0.36971872
0.580231289	1.062085566	0.275399911	0.645639164	0.714893381
0.407394949	0.597412778	0.25518227	0.359484133	0.717126731
0.532406467	0.631913941	0.208121801	0.364451817	0.562880276
0.434642152	0.591684497	0.157573846	0.31837523	0.884908382
0.316285649	0.694477568	0.397409978	0.306933921	0.770731361
0.298499413	0.798409062	0.636706325	0.278799523	0.948750075
0.350867932	0.399453645	0.279651117	0.26624014	0.548742035
0.319502694	0.69193096	0.126701016	0.242591348	0.814451108
0.336178648	0.866637158	0.295289098	0.29617053	0.284125456
0.35072204	0.389933497	0.296684199	0.33690179	0.581358502
0.330472012	0.794599615	0.153222492	0.275992315	0.486496051
0.280777649	0.810340415	0.156225298	0.302309873	0.27100662
0.406351465	0.524676471	0.401340875	0.392509642	0.232870533
0.337766933	0.885951728	0.163311693	0.248807176	0.387481678
0.297755487	0.753719697	0.14704198	0.381855842	1.236703796
0.360307353	0.942327205	0.206698525	0.363493129	0.402371492
0.276605165	0.720015565	0.450625231	0.279476716	0.296992829
0.312515576	0.735603373	0.242591348	0.225938182	0.54178787
0.40584479	0.740976657	0.448412997	0.349993492	0.622437118

O43561,LAT,INFLAMMATION	O43521-2,BCL2L11,INFLAMMATION	Q14435,GALNT3,INFLAMMATION	P01127,PDGFB,INFLAMMATION	Q15517,CDSN,INFLAMMATION
O43561	O43521-2	Q14435	P01127	Q15517
LAT	BCL2L11	GALNT3	PDGFB	CDSN
INFLAMMATION	INFLAMMATION	INFLAMMATION	INFLAMMATION	INFLAMMATION
0.622825535	0.494142826	0.685153905	0.24779174	0.364502345
0.514520334	0.70740092	0.822849221	0.149270398	0.332447861
0.497993908	0.591151575	0.683446351	0.162588824	0.570223736
0.495137114	0.678161129	0.766947644	0.056531547	0.55259714
0.661035657	0.674551267	0.580110646	0.12673615	0.374178871
0.42699764	0.871154192	0.699308041	0.030659968	0.547450337
0.562529242	0.631476084	2.948129709	0.163108062	0.398844973
0.487542537	0.617195736	0.445161274	0.056144947	0.432748283
0.800903317	0.477641468	0.827195361	0.389879445	0.454987874
0.89328968	0.568329694	0.663974619	1.194742739	0.362939252
0.652477474	0.568802614	0.620240656	0.280641448	0.471588355
1.168210292	0.767266675	0.566795418	0.684441907	0.650625828
0.498926775	0.874239216	0.565853307	0.134931585	0.466581173
0.385900281	0.484443392	0.395870372	0.081030003	0.533588693
0.595345886	0.550876185	0.216029464	0.372755101	0.32302103
1.027045431	0.489472598	0.426968043	0.437786691	0.316548837
3.620025852	0.946976152	0.400201922	5.554665363	0.312645574
0.457739913	0.476748399	0.704416203	0.109363256	0.356210019
0.921655819	0.617794957	0.644431982	0.114450843	0.734686159
0.452346433	0.683778042	0.57271925	0.081221191	0.373686409
0.339786397	0.531484676	0.603238327	0.065981254	0.297404834
0.81490286	0.632395935	0.87417862	0.382253072	0.330563651
0.627331539	0.570737792	0.638783964	0.284953812	0.287035285
0.462716356	0.589596555	0.368107743	0.029390628	0.365463695
0.633975942	0.360182502	0.551640391	0.041642486	0.440251554
0.777276606	0.71400199	0.411253539	0.506523111	0.306402506
0.546350998	0.727439918	0.734380675	0.043474101	0.325673898
1.778671394	0.277565468	0.535775308	1.265756594	0.373790032
0.774319028	0.619939786	0.463840275	0.356753626	0.43455178
0.344028502	0.614719434	0.509480912	0.025862401	0.465224829
0.670681634	0.661035657	0.554016174	0.523223774	0.517022869
0.514021283	0.586051762	0.616169852	0.19189646	0.344147754
0.787853886	0.52007803	0.481097097	0.457613018	0.542125961
0.649949711	0.610854208	0.81253395	0.113565773	0.529425651
0.92678076	0.64470005	0.612380394	0.345175028	0.42889607

Q9BU40,CHRDL1,INFLAMMATION	Q99748,NRTN,INFLAMMATION	P12544,GZMA,INFLAMMATION	P13236,CCL4,INFLAMMATION	Q13232,NME3,INFLAMMATION
Q9BU40	Q99748	P12544	P13236	Q13232
CHRDL1	NRTN	GZMA	CCL4	NME3
INFLAMMATION	INFLAMMATION	INFLAMMATION	INFLAMMATION	INFLAMMATION
0.238986329	1.102516311	0.293880186	0.224704356	0.445068715
0.4118526	0.972992102	0.379928541	0.198457226	0.526972653
0.425992519	1.133747995	0.333232265	0.289894527	0.564521331
0.318485589	1.344528187	0.412081042	1.804501072	0.403740439
0.418094219	1.203970464	0.414458628	0.565657231	0.491682871
0.384751805	0.946844883	0.348927691	0.420885584	0.402985546
0.26851973	0.940304547	0.335829297	0.335224617	0.393490306
0.334621025	0.761702745	0.241551045	0.246284886	0.390474435
0.295268631	0.749967509	0.383447243	0.327462135	0.514413354
0.292052573	1.074823671	0.343385256	0.382279568	0.300304917
0.216599227	0.996402123	0.335015558	0.271269734	0.408809322
0.619767926	1.026191514	0.496167794	0.363165736	0.62676651
0.212627485	0.961260928	0.325267819	0.260959893	0.45584018
0.210471967	0.546843532	0.301974786	0.215103067	0.407733952
0.159640654	0.493082173	0.24313003	0.26230187	0.329283014
0.245654062	0.700084029	0.570816918	0.30776477	0.384192164
0.268296474	1.231912681	0.459010799	0.42625835	0.535812447
0.285448028	1.084477409	0.380244688	0.243248026	0.533255927
0.288251496	1.560599129	0.422610348	0.35030901	0.502397085
0.29761105	1.025125116	0.359210145	0.20264128	0.442822367
0.249186877	1.065403555	0.208092951	0.340139863	0.457295935
0.244939949	1.652900636	0.320678603	0.481530805	0.365742453
0.291425697	1.172672371	0.275476279	0.643271644	0.449035061
0.204901152	1.234562607	0.299618788	0.177956927	0.418529146
0.226486977	1.007793461	0.379954877	0.387642861	0.447016503
0.332010322	0.704904637	0.468493196	0.382306067	0.399592109
0.298830643	1.1640879	0.352965725	0.583174685	0.471326924
0.277392368	1.169993082	0.27268363	0.276260269	0.448226546
0.351744556	0.754451466	0.448164413	0.311779937	0.552175966
0.295411931	2.041882714	0.197756908	0.332240534	0.385232146
0.220583992	1.023208391	0.298830643	0.325470795	0.301870148
0.296848762	0.914528478	0.265576611	0.321012193	0.467617232
0.25458159	0.990136957	0.336925143	0.272513574	0.381988206
0.165637358	1.189042267	0.304159506	0.187180234	0.397465074
0.341794242	1.032971178	0.412423944	0.243501067	0.33645839
