# Supplementary material for: Putative Concussion Biomarkers Identified in Adolescent Male Athletes Using Targeted Plasma Proteomics
Source: Front Neurol. 2021 Dec 20;12:787480. doi: 10.3389/fneur.2021.787480 (PMC8721148; doi:10.3389/fneur.2021.787480)
Supplement: Supplementary file 2 [file Data_Sheet_2.PDF]

Table 2: Plasma protein expression between concussion patients and control subjects.

Protein	Concussion Patients (n=11)	Control Subjects (n=24)	Change	Fold-Change	P-value	AUC
ATOX1	0.50 (0.47, 0.52)	0.57 (0.52, 0.73)	↓	0.88	0.003	0.814
SPARC	0.41 (0.35, 0.55)	0.65 (0.50, 0.80)	↓	0.63	0.004	0.811
CD34	0.55 (0.53, 0.62)	0.69 (0.62, 0.84)	↓	0.80	0.006	0.792
PQBP1	0.47 (0.40, 0.62)	0.64 (0.54, 0.82)	↓	0.73	0.008	0.784
IGFBPL1	0.43 (0.35, 0.46)	0.48 (0.44, 0.55)	↓	0.90	0.008	0.784
NT5C3A	0.98 (0.91, 1.26)	0.75 (0.59, 0.91)	↑	1.31	0.009	0.780
COL4A1	0.39 (0.34, 0.49)	0.55 (0.44, 0.66)	↓	0.71	0.009	0.777
SPINT2	0.43 (0.41, 0.44)	0.35 (0.33, 0.42)	↑	1.23	0.009	0.777
CRTAC1	0.17 (0.13, 0.22)	0.22 (0.21, 0.31)	↓	0.77	0.011	0.773
S100P	0.55 (0.48, 0.70)	0.71 (0.64, 0.88)	↓	0.77	0.011	0.773
HAVCR1	0.16 (0.12, 0.20)	0.11 (0.09, 0.15)	↑	1.45	0.012	0.769
APBB1IP	0.46 (0.37, 0.53)	0.54 (0.46, 0.60)	↓	0.85	0.013	0.765
KYNU	0.26 (0.18, 0.30)	0.32 (0.26, 0.40)	↓	0.81	0.013	0.765
SPINK6	0.25 (0.14, 0.26)	0.32 (0.21, 0.50)	↓	0.78	0.014	0.761
JUN	0.42 (0.38, 0.44)	0.47 (0.41, 0.60)	↓	0.89	0.014	0.761
TRIM5	0.76 (0.70, 0.87)	0.92 (0.81, 1.07)	↓	0.83	0.014	0.761
PPP3R1	0.54 (0.44, 0.59)	0.66 (0.57, 0.71)	↓	0.82	0.016	0.758
IL6	0.41 (0.32, 0.51)	0.58 (0.44, 0.99)	↓	0.71	0.016	0.758
BAIAP2	0.40 (0.30, 0.55)	0.53 (0.45, 0.80)	↓	0.75	0.016	0.758
MPO	0.30 (0.27, 0.37)	0.37 (0.34, 0.44)	↓	0.81	0.017	0.754
ANKRD54	0.81 (0.67, 0.92)	0.63 (0.58, 0.75)	↑	1.29	0.017	0.754
MSTN	0.30 (0.22, 0.31)	0.41 (0.29, 0.52)	↓	0.73	0.017	0.754
DRAXIN	0.82 (0.74, 1.09)	1.25 (0.95, 1.46)	↓	0.66	0.019	0.750
CDH2	0.23 (0.20, 0.26)	0.28 (0.24, 0.34)	↓	0.82	0.019	0.750
PLA2G10	0.47 (0.38, 0.65)	0.72 (0.52, 0.86)	↓	0.65	0.021	0.746
LAMP2	0.49 (0.44, 0.54)	0.57 (0.48, 0.71)	↓	0.86	0.021	0.746
HSPB6	0.13 (0.12, 0.16)	0.19 (0.14, 0.25)	↓	0.68	0.021	0.746

WIF1	0.73 (0.56, 0.89)	0.95 (0.80, 1.07)	↓	0.77	0.021	0.746
PPY	0.13 (0.11, 0.15)	0.18 (0.13, 0.27)	↓	0.72	0.021	0.746
L1CAM	0.56 (0.50, 0.56)	0.63 (0.52, 0.67)	↓	0.89	0.021	0.746
PON2	0.61 (0.51, 0.70)	0.76 (0.60, 0.84)	↓	0.80	0.021	0.746
EDIL3	0.89 (0.85, 0.96)	1.11 (0.86, 1.38)	↓	0.80	0.021	0.746
SPINK4	0.48 (0.44, 0.57)	0.64 (0.50, 0.78)	↓	0.75	0.021	0.746
IL16	0.18 (0.17, 0.23)	0.25 (0.19, 0.31)	↓	0.72	0.021	0.746
CXCL8	0.41 (0.33, 0.58)	0.31 (0.27, 0.38)	↑	1.32	0.023	0.742
CPE	0.34 (0.29, 0.38)	0.43 (0.33, 0.53)	↓	0.79	0.023	0.742
ADA2	0.40 (0.37, 0.48)	0.51 (0.43, 0.59)	↓	0.78	0.023	0.742
EPHA1	0.27 (0.23, 0.34)	0.22 (0.15, 0.28)	↑	1.23	0.023	0.742
EPO	0.35 (0.30, 0.37)	0.41 (0.34, 0.49)	↓	0.85	0.023	0.742
LIF	0.51 (0.45, 0.62)	0.62 (0.56, 0.70)	↓	0.82	0.025	0.739
LBR	0.46 (0.40, 0.48)	0.53 (0.46, 0.57)	↓	0.87	0.025	0.739
SETMAR	0.34 (0.32, 0.39)	0.41 (0.35, 0.46)	↓	0.83	0.025	0.739
SCLY	0.27 (0.23, 0.33)	0.35 (0.29, 0.39)	↓	0.77	0.025	0.739
CD209	0.17 (0.16, 0.22)	0.23 (0.20, 0.31)	↓	0.74	0.025	0.739
FBP1	0.28 (0.24, 0.32)	0.39 (0.31, 0.55)	↓	0.72	0.025	0.739
TMSB10	0.20 (0.18, 0.21)	0.28 (0.19, 0.33)	↓	0.71	0.028	0.735
FCRL5	0.31 (0.27, 0.39)	0.25 (0.22, 0.33)	↑	1.24	0.030	0.731
RHOC	0.42 (0.37, 0.45)	0.47 (0.41, 0.58)	↓	0.89	0.030	0.731
ATP6V1D	1.15 (1.06, 1.31)	1.02 (0.98, 1.15)	↑	1.13	0.030	0.731
TGM2	2.83 (2.02, 5.00)	2.13 (1.70, 2.97)	↑	1.33	0.030	0.731
CCN2	0.45 (0.39, 0.57)	0.54 (0.47, 0.66)	↓	0.83	0.030	0.731
SCGB3A2	0.27 (0.18, 0.32)	0.34 (0.28, 0.49)	↓	0.79	0.030	0.731
HNMT	0.17 (0.16, 0.19)	0.23 (0.17, 0.30)	↓	0.74	0.033	0.727
TNXB	0.58 (0.51, 0.68)	0.70 (0.60, 0.79)	↓	0.83	0.033	0.727
TXNDC5	0.48 (0.41, 0.57)	0.55 (0.48, 0.68)	↓	0.87	0.033	0.727
SMOC1	0.11 (0.10, 0.13)	0.14 (0.12, 0.17)	↓	0.79	0.033	0.727

FMR1	0.82 (0.65, 0.90)	0.96 (0.84, 1.06)	↓	0.85	0.033	0.727
RABEPK	0.83 (0.66, 0.95)	1.01 (0.81, 1.06)	↓	0.82	0.033	0.727
LPL	0.62 (0.48, 0.80)	0.77 (0.64, 0.91)	↓	0.81	0.033	0.727
DNMBP	0.30 (0.26, 0.33)	0.36 (0.32, 0.44)	↓	0.83	0.036	0.723
SNCG	0.16 (0.14, 0.19)	0.23 (0.17, 0.33)	↓	0.70	0.036	0.723
CNTN5	0.57 (0.45, 0.63)	0.76 (0.53, 1.05)	↓	0.75	0.036	0.723
CDH15	0.45 (0.42, 0.62)	0.60 (0.46, 0.81)	↓	0.75	0.036	0.723
EPS8L2	0.29 (0.28, 0.33)	0.40 (0.28, 0.47)	↓	0.73	0.036	0.723
CCL14	0.55 (0.48, 0.60)	0.45 (0.38, 0.50)	↑	1.22	0.036	0.723
DCTPP1	0.36 (0.27, 0.37)	0.42 (0.34, 0.48)	↓	0.86	0.036	0.723
ANGPTL1	0.41 (0.39, 0.58)	0.53 (0.48, 0.67)	↓	0.77	0.036	0.723
CLC	0.35 (0.32, 0.39)	0.46 (0.36, 0.55)	↓	0.76	0.036	0.723
IL12B	0.60 (0.54, 0.67)	0.75 (0.58, 0.86)	↓	0.80	0.036	0.723
RAB6A	0.33 (0.27, 0.39)	0.40 (0.33, 0.49)	↓	0.83	0.036	0.723
CLEC4A	0.43 (0.38, 0.55)	0.58 (0.47, 0.73)	↓	0.74	0.036	0.723
CLEC11A	0.58 (0.47, 0.64)	0.73 (0.56, 0.90)	↓	0.79	0.039	0.720
FABP5	0.31 (0.25, 0.34)	0.37 (0.32, 0.43)	↓	0.84	0.039	0.720
LYPD1	1.06 (0.89, 1.14)	1.13 (1.06, 1.32)	↓	0.94	0.039	0.720
ANXA3	0.20 (0.18, 0.29)	0.25 (0.23, 0.30)	↓	0.80	0.039	0.720
STX16	0.64 (0.61, 0.66)	0.56 (0.45, 0.63)	↑	1.14	0.039	0.720
VWF	0.21 (0.18, 0.34)	0.30 (0.24, 0.43)	↓	0.70	0.039	0.720
PLA2G2A	0.70 (0.55, 0.96)	0.92 (0.73, 1.33)	↓	0.76	0.039	0.720
NCAM1	0.46 (0.41, 0.50)	0.58 (0.46, 0.64)	↓	0.79	0.039	0.720
ATXN10	0.12 (0.06, 0.14)	0.15 (0.12, 0.17)	↓	0.80	0.043	0.716
TEK	0.38 (0.36, 0.44)	0.44 (0.39, 0.51)	↓	0.86	0.043	0.716
CES2	0.43 (0.39, 0.49)	0.34 (0.30, 0.44)	↑	1.26	0.043	0.716
CLEC1A	0.62 (0.56, 0.76)	0.76 (0.68, 0.91)	↓	0.82	0.043	0.716
NUDC	0.35 (0.32, 0.38)	0.41 (0.36, 0.52)	↓	0.85	0.043	0.716
FST	0.18 (0.11, 0.21)	0.24 (0.16, 0.27)	↓	0.75	0.043	0.716

NEFL	0.44 (0.39, 0.55)	0.37 (0.29, 0.43)	↑	1.19	0.047	0.712
ENO2	0.78 (0.70, 0.84)	0.87 (0.79, 0.95)	↓	0.90	0.047	0.712
NOS1	0.09 (0.06, 0.13)	0.12 (0.09, 0.15)	↓	0.75	0.047	0.712
MIA	0.71 (0.53, 0.80)	0.79 (0.67, 0.93)	↓	0.90	0.047	0.712
ACTA2	0.44 (0.40, 0.51)	0.54 (0.46, 0.67)	↓	0.81	0.047	0.712
TYMP	0.26 (0.25, 0.33)	0.35 (0.27, 0.41)	↓	0.74	0.047	0.712
SDC1	0.46 (0.37, 0.56)	0.35 (0.32, 0.44)	↑	1.31	0.047	0.712

Data are presented as median (IQRs). AUC, area under the curve.
